# Niaoduqing alleviates podocyte injury in high glucose model *via* regulating multiple targets and AGE/RAGE pathway: Network pharmacology and experimental validation

**DOI:** 10.3389/fphar.2023.1047184

**Published:** 2023-02-27

**Authors:** Yipeng Fang, Yunfei Zhang, Chenxi Jia, Chunhong Ren, Xutao Zhao, Xin Zhang

**Affiliations:** ^1^ Laboratory of Molecular Cardiology, The First Affiliated Hospital of Shantou University Medical College, Shantou, Guangdong, China; ^2^ Laboratory of Medical Molecular Imaging, The First Affiliated Hospital of Shantou University Medical College, Shantou, Guangdong, China; ^3^ Shantou University Medical College, Shantou, Guangdong, China; ^4^ Tianjin Hospital of Tianjin University, Tianjin, China; ^5^ International Medical Service Center, The First Affiliated Hospital of Shantou University Medical College, Shantou, Guangdong, China; ^6^ Jinan Municipal Hospital of Traditional Chinese Medicine, Jinan, Shandong, China

**Keywords:** Niaoduqing particles, uremic clearance granule, diabetic nephropathy, podocyte injury, proteinuria, network pharmacology, AGE/RAGE signaling

## Abstract

**Purpose:** The aim of present study was to explore the pharmacological mechanisms of Niaoduqing granules on the treatment of podocyte injury in diabetic nephropathy (DN) *via* network pharmacology and experimental validation.

**Methods:** Active ingredients and related targets of Niaoduqing, as well as related genes of podocyte injury, proteinuria and DN, were obtained from public databases. Gene ontology (GO), Kyoto Encyclopedia of Genes and Genomes (KEGG) and protein-protein interaction (PPI) network analysis were performed to investigate the potential mechanisms. High glucose (HG) -induced MPC5 cell injury model was treated with the major core active ingredients of Niaoduqing and used to validate the predicted targets and signaling pathways.

**Results:** Totally, 16 potential therapeutic targets were identified by intersecting the targets of Niaoduqing and disease, in which 7 of them were considered as the core targets *via* PPI network analysis. KEGG enrichment analysis showed that AGE-RAGE signaling pathway was identified as the most crucial signaling pathway. The results of *in vitro* experiments revealed that the treatment of Niaoduqing active ingredients significantly protected MPC5 cells from HG-induced apoptosis. Moreover, Niaoduqing could significantly attenuate the HG-induced activation of AGE-RAGE signaling pathway, whereas inhibited the over-expression of VEGF-A, ICAM-1, PTGS-2 and ACE in HG-induced MPC5 cells.

**Conclusion:** Niaoduqing might protect against podocyte injury in DN through regulating the activity of AGE/RAGE pathway and expression of multiple genes. Further clinical and animal experimental studies are necessary to confirm present findings.

## 1 Introduction

Diabetic nephropathy (DN) is one of the most common complications of diabetes mellitus, which involves the entire kidneys (Anders et al., 2018). In China, the prevalence of total diabetes in adults was 11.2% (Li et al., 2020a). DN develops in approximately 20%–40% of patients with diabetes and consequently has become the leading cause of chronic kidney disease (CKD) and end-stage renal disease (ESRD) (Zhang et al., 2016; American Diabetes Association, 2020). Over the years, lifestyle change, risk factor control, proteinuria inhibition and interstitial fibrosis prevention are the primary modes of treatment for DN. However, current management approaches cannot stop the progression of renal failure (Yang et al., 2019a).

The pathogenesis of DN is complex and multifactorial, among which podocyte injury plays the key role. In diabetes, declining insulin sensitivity, oxidative stress and inflammatory reaction cause permanent functional and/or structural change of podocytes, that is regarded as one of the major causes of proteinuria. Podocyte damage, including dysfunction, shedding and apoptosis, is considered as the early pathological change underlying various glomerular diseases, including DN (Cao et al., 2014; Ni et al., 2018; Podgórski et al., 2019). Proteinuria is one of the early clinical manifestations of diabetic kidney disease, and persistent proteinuria accelerates the progression of renal disease (Brinkkoetter et al., 2019; Yang et al., 2019b). Thus, podocyte might be a potential therapeutic target for DN (Ni et al., 2018), and controlling proteinuria represents an effective method delaying the progression of diabetic kidney damage. Angiotensin converting enzyme inhibitors (ACEI) and angiotensin receptor blocker (ARB) are widely used in patients with proteinuria, in order to reduce albuminuria and decrease the risk of cardiovascular diseases through inhibiting the activity of renin-angiotensin system (RAS); however, whether ACEI or ARB can prevent the progression towards ESRD are still uncertain (Marre et al., 2004). According to the theory of traditional Chinese medicine (TCM), Chinese compound medicines treat diverse diseases through “multi-component, multi-targets and multi-pathways” method (Zhang et al., 2020). The complex mechanisms of DN suggest that a combination of medicines may play better therapeutic activities in DN. Niaoduqing granules, consisted of 9 herbal medicines, are commonly used in ESRD. As Li et al. (2022a) reported, Niaoduqing granules can effectively improve renal function, inhibit renal fibrosis and decrease the level of inflammatory responses through regulating MAPK/NF-κB signaling pathway in the ESRD model induced by 5/6 nephrectomy. TGF-β is considered as one of the crucial targets for the anti-fibrosis of Niaoduqing (Miao et al., 2010; Lu et al., 2013; Huang et al., 2014; Wu et al., 2016). As Huang YR et al. reported, Niaoduqing granules ameliorate tubule-interstitial fibrosis and renal dysfunction in the renal failure model induced by adenine and unilateral ureteral obstruction though promoting extracellular matrix degradation and maintaining MMP-2/TIMP-1 balance or regulating TGF-beta1/Smad signaling pathway in kidney tissue (Huang et al., 2014). In addition, Niaoduqing can also treat tubule-interstitial fibrosis *via* inhibiting tubular epithelial-to-mesenchymal transition (EMT) and regulating TGF-beta1/Smad pathway (Lu et al., 2013). The interaction between Niaoduqing and TGF-β1 may be related to the methylation/demethylation regulation of TGF-β1 promoter (Miao et al., 2010). What’s more, Niaoduqing granules can ameliorate CKD-related anemia though erythropoietin (EPO) receptor signaling pathway (Wang et al., 2017). Except for ameliorating renal function and fibrosis, Niaoduqing also has good effects on managing uremic pruritus (Lu et al., 2021). Niaoduqing also regulate the amino acid, lipid and energy metabolisms in the chronic renal failure rat model (Zhu et al., 2018). The therapeutic effects of Niaoduqing on DN have been evaluated in some Chinese articles. As Wu et al. (2009) found in an intervention research including 76 DN patients without dialysis or kidney transplant, Niaoduqing exposure can effectively improve the clinical symptoms (92.10% vs. 65.78%, *p* < 0.05), and reduce the level of blood urea nitrogen (BUN, 15.9 ± 1.75 mmol/L vs. 16.9 ± 1.34 mmol/L, *p* < 0.05), blood creatinine (Scr, 383.2 ± 74.58 μmol/L vs. 425.74 ± 86.32 μmol/L, *p* < 0.05) and urine protein (0.81 ± 0.67 g/24 h vs. 1.38 ± 0.45 g/24 h, *p* < 0.05). Compared with using ACEI/ARB alone, the better renal function is observed in patients received combination therapy with Niaoduqing (Li et al., 2016; Wei and Ruan, 2018). What’s more, a recent network meta-analysis reported that Niaoduqing has a better effect on controlling proteinuria in patients with early stage DN, compared with other six kinds of TCM (Zhao et al., 2022). Although some studies have proved the positive role of Niaoduqing in the treatment of diabetes nephropathy and proteinuria, the underline mechanisms of Niaoduqing for early stage DN are still unclear and need to be further explored.

**FIGURE 1:**
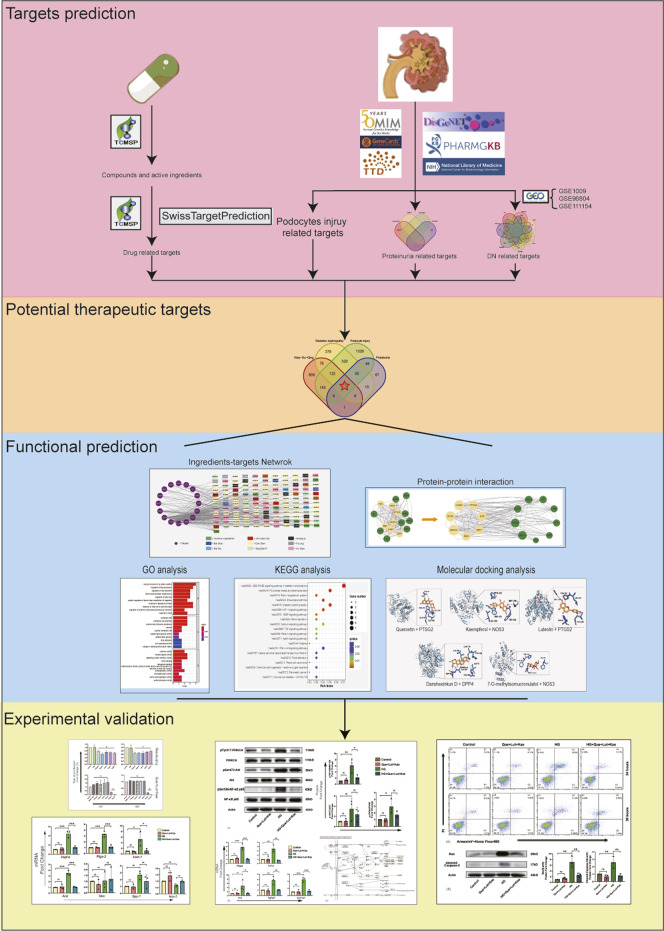
Flow chart of present study.

Network pharmacology is an analytic tool for systematic pharmacology based on the “network target, multi-component” strategy, which has been widely applied to analyze the active ingredients and core potential therapeutic targets of drugs to disease, especially in Chinese compound medicines (Hopkins, 2007; Kibble et al., 2015). In the present study, we explored the core ingredients and potential mechanisms of Niaoduqing granules on the treatment of podocyte damage and proteinuria in DN through network pharmacology followed by experimental validation, so as to search for novel and effective therapeutic strategies for podocyte protection and proteinuria reduction in DN. The flow chart of our study was shown in Figure 1.

## 2 Materials and methods

### 2.1 Screen the active ingredients and targets of Niaoduqing granules

Niaoduqing granules consist of nine components, including Bai Shao, Bai Shu, Che Qian Cao, Da Huang, Dan Shen, Fu Ling, Huang Qi, Ku Shen and Sang Bai Ye. The active ingredients of the above night components were screened through the Traditional Chinese Medicine Systems Pharmacology Database and Analysis Platform (TCMSP, https://tcmsp-e.com/tcmsp.php) according to the condition of oral bioavailability (OB) ≥ 30% and drug-like properties (DL) ≥ 0.18 (Ru et al., 2014). Related targets of the active ingredients, defined as Niaoduqing-related targets, were selected through TCMSP database and Swiss Target Prediction website (http://www.swisstargetprediction.ch/) (Ru et al., 2014; Daina et al., 2019). In Swiss Target Prediction website, only the top 100 predicted targets with probability greater than 0 were included. SMILE strings, which should be used in the Swiss Target Prediction website, were obtained through Pubchem website (https://pubchem.ncbi.nlm.nih.gov/). The conversions from protein names to the unique entry gene IDs were performed through the uniprot database (https://www.uniprot.org/).

### 2.2 Screen differentially expressed genes related to DN from GEO database

DN related databases (GSE1009, GSE96804 and GSE111154) were obtained from Gene Expression Omnibus (GEO) database. After removing duplicate and missing data, we screened the differentially expressed genes (DEGs) according to the following criteria: │log FC│≥1 and *p*-value < 0.05. Volcano plots and heat maps were used to represent the DEGs.

### 2.3 Collect related targets and potential therapeutic targets

Disease targets of DN, proteinuria and podocyte injury were attained by searching GeneCards database (https://www.genecards.org/) (Stelzer et al., 2016), the Online Mendelian Inheritance in Man database (OMIM, https://omim.org/) (Amberger and Hamosh, 2017), Therapeutic target database (TTD, http://db.idrblab.net/ttd/) (Li et al., 2018), DisGeNET database (https://www.disgenet.org/home/) (Piñero et al., 2015), NCBI (https://www.ncbi.nlm.nih.gov/) and PharmgKB database (https://www.pharmgkb.org/) (Whirl-Carrillo et al., 2021) with “diabetes nephropathy,” “proteinuria” and “podocyte injury” as keywords and “*Homo sapiens*” as the organism.

All disease targets obtained from the above databases and the DEGs of DN obtained from GSE1009, GSE96804 and GSE111154 datasets were pooled together, and those targets appearing in at least two databases and datasets were defined as DN-related targets in present study. Similar protocol was applied to screen the proteinuria-related genes: only the overlapping targets appearing in at least two databases were identified as proteinuria-related genes.

Niaoduqing-related targets were intersected with the DN-related targets, the proteinuria-related targets and the podocyte injury-related targets to identify the potential therapeutic targets of Niaoduqing on the treatment of podocyte injury and proteinuria in DN. “venn” and “VennDiagram” packages from R language were used to create the Venn diagram to depict the intersections between different databases and datasets. Cytoscape 3.6.1 software was used to construct the relationship network among night components, active ingredients and the potential therapeutic targets.

### 2.4 The analysis of PPI network, GO and KEGG

The protein-protein interactions (PPI) results among potential therapeutic targets were obtained from the SRTING database (https://cn.string-db.org/, Version: 11.5), with the minimum required interaction score set at “median confidence (0.400)” level. Cytoscape 3.6.1 and its CytoNCA plugin were used to further analyze the original PPI network. Three topological parameters, including betweenness centrality, closeness centrality and degree value, were calculated and considered as the evidence for the screen of core targets. The higher the values were, the more important the targets were (Azuaje et al., 2011). Nodes with all three parameters higher than the median were used to build the sub-network and considered as the core targets.

Gene Ontology (GO) and Kyoto Encyclopedia of Genes and Genomes (KEGG) pathway enrichment analysis were performed using “Cluster profiler” package from R language to investigate the probable molecular mechanisms of Niaoduqing on the treatment of podocyte injury and proteinuria in DN. The top 10 enriched enters of molecular function (MF), biological process (BP) and cellular components (CC) in GO analysis were presented in bar chart. The top 20 enriched pathways were shown in bubble chart.

### 2.5 Molecular docking

The core ingredients and core targets were used in the molecular docking analysis. Firstly, the three-dimensional structure of core active ingredients was obtained from Pubchem website and translated into PDB format files using PyMOL software. Secondly, the 3D structure of the core target was obtained through the protein docking bank database (PDB, https://www.rcsb.org/). The water molecules, metal ions and small molecule ligands were removed, and the active pockets were identified by PyMOL software. Thirdly, AutoDock Vina 1.1.2 (Trott and Olson, 2010) was used to convert the ingredients and targets into PDBQT format files and perform molecular docking simulation. This binding energy estimated the stability of the target and the ingredient complexes. The first representative binding pose, with the lowest binding energy in our docking result, was visualized by PyMOL software.

### 2.6 Experimental validation *in vitro*

#### 2.6.1 Drugs and reagents

Niaoduqing Granules were obtained from KangCheng Pharmaceutical Industry, China (No. Z20073256). The lyophilized powder of quercetin (Que, Q4951) was acquired from Sigma (St. Louis, United States). The lyophilized powders of kaempferol (Kae, S2314) and luteolin (Lut, S2320) were obtained from Selleck (Shanghai, China). Anti-PI3K antibody (110kD, AF5112) was purchased from Affinity Biosciences (Ohio, United States). Anti-phospho-PI3KCA antibody (p-PI3K^Tyr317^, 110kD, bs-5570R) was obtained from Bioss Biotech (Beijing, China). Anti-AKT antibody (60 kD, 4691S), anti-phospho-AKT antibody (p-AKT^Ser473^, 60kD, 4060S), anti-caspase-3 antibody (17kD, 9662) and anti-Bax antibody (20kD, 2772S) was acquired from Cell Signaling Technology (CST, Danvers, MA, United States). Anti-NF-κB antibody (65kD, A2547) and anti-phosphor-NF-κB p65/RelA antibody (p-NF-κB^Ser536^, 65kD, AP0124) was purchased from ABclonal Biotech (Wuhan, China). Goat anti-mouse IgG second antibody (C1308), goat anti-rabbit IgG second antibody (C1309) and anti-actin antibody (42kD, C1313) was acquired from Pulilai Biotech (Beijing, China). Annexin V—Alexa Flour 488/PI Apoptosis Kit (FXP022) was purchased from 4A Biotech (Suzhou, China). CCK-8 (CK04) was obtained from Dojindo Laboratorise (Shanghai, China). Fetal bovine serum (FBS) was purchased from Zeta Life (California, United States).

#### 2.6.2 Cell culture

The conditionally immortalized mouse podocyte cell line Mouse Podocyte Clone 5 (MPC5) cells were purchased from Jennio Biotech (Guangzhou, China). MPC5 cells were maintained in RPMI 1640 medium supplemented with 15% FBS, 2 mM L-Glutamin, 100 IU/mL penicillin-streptomycin, and 5 U/mL recombinant mouse interferon-γ (IFN-γ, Yeasen Biotech, Shanghai, China, 91212ES60) at 33°C in a humidified atmosphere with 5% CO_2_. To induce differentiation, MPC5 cells were shifted from 33°C to 37°C and cultured without IFN-γ for 14 days. To establish high glucose (HG) model, extra glucose (Sigma, St. Louis, United States, G7021) was added to growth medium and differentiated MPC5 cells were cultured under high glucose condition (44 mM). Since the solution of Niaoduqing granules showed toxic effect on the survival and proliferation of MPC5 cells, the mixture of three major active ingredients of Niaoduqing (Que + Lut + Kae) were used as an alternative for the *in vitro* experiments. MPC5 cells were randomly divided into four groups: the control group, Que + Lut + Kae (1 μg/mL) group, HG group and HG + Que + Lut + Kae group.

#### 2.6.3 CCK-8 assay for cell viability

MPC5 cells were re-plated in 96-well plates (5,000 cells per well) and cultured at 37°C overnight. Then, growth medium was removed and 100 μL of culture medium with different concentrations of Niaoduqing granules (0.0625, 0.25, 1, 4, 16 μg/mL) and Que + Lut + Kae mixture (0.0625, 0.25, 1, 4, 16, 64 μg/mL) was added. Twenty four and 48 h after treatment, 100 μL of basic medium and 10 μL CCK-8 solution were added to each well and incubated for another 2 h. The optical density (OD) value was measured at 490 nm. The cell viability was calculated using the following formula: Cell viability% = [(OD_value of experimental group_) − (OD_value of cell-free group_)]/[(OD_value of control group_)-(OD_value of cell-free group_)] × 100%.

#### 2.6.4 Real time quantitative PCR (RT-qPCR) analysis

Total RNA was extracted from each group of MPC5 cells 48 h after treatment using Trizol method (Accurate Biotechnology, Changsha, China). The cDNA was synthesized using Evo M-MLV RT kit (Accurate Biotechnology, AG11734). The mRNA was quantified using the 2× SYBR Green Pro Taq HS Premix II (Accurate Biotechnology, AG11736), with *β-actin* gene as the internal control. The differences of the gene expression were analyzed using the delta-delta Ct method (2^−△△CT^). The primer sequences for RT-qPCR are shown in Table 1.

**TABLE 1:** Primer sequences of RT-PCR.

Gene name	Forward primer sequences (5′-3′)	Reverse primer sequences (5′-3′)
*Ace*	CCA​ACA​AGA​TTG​CCA​AGC​TCA	AGT​GGC​TGC​AGC​TCC​TGG​TA
*β-actin*	ACC​AAC​TGG​GAC​GAC​ATG​GAG​AAG	TAC​GAC​CAG​AGG​CAT​ACA​GGG​ACA
*Col1a1*	TGG​CCT​TGG​AGG​AAA​CTT​TG	CTT​GGA​AAC​CTT​GTG​GAC​CAG
*Icam-1*	GCC​TTG​GTA​GAG​GTG​ACT​GAG	GAC​CGG​AGC​TGA​AAA​GTT​GTA
*Il-6*	TTA​TAT​CCA​GTT​TGG​TAG​CAT​CCA​T	AGG​CTT​AAT​TAC​ACA​TGT​TCT​CTG​G
*Nos-3*	ATT​TCC​TGT​CCC​CTG​CCT​TCC​GC	GGT​TGC​CTT​CAC​ACG​CTT​CGC​C
*Ptgs-2*	TTC​AAC​ACA​CTC​TAT​CAC​TGG​C	AGA​AGC​GTT​TGC​GGT​ACT​CAT
*Rage*	CAGGGTCACAGAAACCGG	ATT​CAG​CTC​TGC​ACG​TTC​CT
*Ren*	GAG​GCC​TTC​CTT​GAC​CAA​TC	TGT​GAA​TCC​CAC​AAG​CAA​GG
*Spp-1*	TGG​GCT​CTT​AGC​TTA​GTC​TGT​TG	CAG​AAG​CAA​AGT​GCA​GAA​GC
*Tgfβ1*	CCA​CCT​GCA​AGA​CCA​TCG​AC	CTG​GCG​AGC​CTT​AGT​TTG​GAC
*Tnf-α*	CCC​TCA​CAC​TCA​GAT​CAT​CTT​CT	GCT​ACG​ACG​TGG​GCT​ACA​G
*Vegf-α*	CTT​TTC​GTC​CAA​CTT​CTG​GGC​TCT​T	CCT​TCT​CTT​CCT​CCC​CTC​TCT​TCT​C
*Wt-1*	TAC​AGA​TGC​ATA​GCC​GGA​AGC​ACA	TCA​CAC​CTG​TGT​GTC​TCC​TTT​GGT

#### 2.6.5 Western blot

Protein was extracted from different groups of MPC5 cells using RIPA lysis buffer (Beyotime Biotech, Beijing, China, P0013K) containing 1:100 protease inhibitors and 1:100 phosphatase inhibitors. The protein was quantified using bicinchoninic acid kit (BCA, Beyotime Biotech, Beijing, China, P0012) according to the manufacturer’s instruction. After adding 5 × protein loading buffer, all samples were denatured by boiling at 100°C for 10 min and separated by Sodium dodecyl sulfate-polyacrylamide gel electrophoresis (SDS-PAGE). The electrophoresis was performed at a constant voltage of 60 V for 60 min initially and then switched to 120 V. The gel was further blotted to PVDF membrane for 120 min at a constant current of 200 mA. After blocking with 5% skimmed milk, membranes were incubated with primary antibody at a concentration of 1:1000 at 4°C overnight and further incubated with secondary antibody at a concentration of 1:5000 for 1 h at room temperature. The protein blots were visualized using enhanced chemiluminescence reagent (NCM Biotech, Suzhou, China, P10100). Quantitative analysis was completed using ImageJ.

#### 2.6.6 Flow cytometric analysis

Apoptosis was determined using an Annexin V/Alexa Fluor 488/propidium iodide Apoptosis Detection Kit (FXP022-100, 4A Biotech Co., Ltd.) according to the manufacturers’ instructions. Briefly, MPCs cells were washed twice with cold phosphate-buffered saline (PBS) and then re-suspended in 100 µL of 1 × binding buffer. The cell suspension was incubated with AnnexinV-Alexa Flour 488 (5 µL) for 5 min in dark at room temperature, then 10 µL of PI solution and 400 µL of PBS was added. Samples were measured on Accuri C6 flow cytometer (BD Biosciences) and data were analyzed by FlowJo 8.0 software (Tree Star, Ashland, OR).

### 2.7 Statistical analysis

Data shown in present study repeated at least three times. All data were showed as mean and standard deviation of the mean (SD) and analyzed by SPSS23.0 (SPSS, Armonk, New York, United States). Student’s *t*-test and Bonferroni test in ANOVA were used to make comparisons between two and multiple groups. *p* < 0.05 was considered as a significant difference (*p < 0.05, **p < 0.01, ***p < 0.001, NS = non-statistically significant). GraphPad Prism 8 (GraphPad Software, United States) was used to visualize the results.

## 3 Results

### 3.1 Collection of active ingredients and predicted targets

A total of 206 active ingredients were identified, including 32 from Sang Bai Ye, 13 from Bai Shao, 8 from Bai Shu, 10 from Che Qian Cao, 16 from Da Huang, 67 from Dan Shen, 16 from Fu Ling, 20 from Huang Qi and 45 from Ku Shen (Supplementary Table S1). There were 11 common ingredients discovered in at least two herb compounds, in which 6 ingredients (hederagenin, sitosterol, formononetin, baicalin, gallic acid-3-O-(6′-O-galloyl)-glucoside, (24S)-24-Propylcholesta-5-Ene-3beta-Ol) were common to two herbs and 5 ingredients (quercetin, luteolin, kaempferol, beta-sitosterol, mairin) were common to three herbs (Supplementary Table S1). Among the active ingredients, 142 got predicted targets from TCMSP database and 133 obtained targets information from Swiss Targets Prediction website. 28 active ingredients had no target information (Supplementary Table S2). Finally, after eliminating the duplicates, we identified 1,022 predicted targets of Niaoduqing (Supplementary Table S3), and the relationship between active ingredients and predicted targets was shown in Supplementary Table S4.

### 3.2 Potential therapeutic targets of Niaoduqing on the treatment of podocyte injury and proteinuria in DN

**FIGURE 2:**
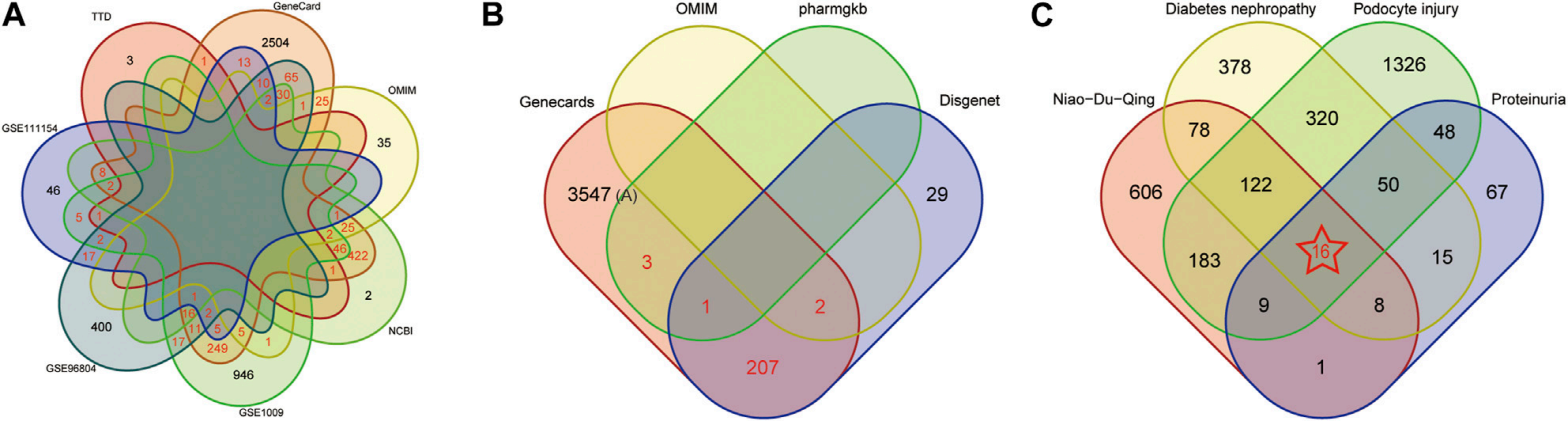
Screening Potential Therapeutic Targets of Niaoduqing on the treatment of podocyte injury and proteinuria in DN. **(A)** The Venn diagram of disease targets in 4 databases and 3 GEO datasets. Overlapping targets in at least two databases and dataset were considered as DN-related targets (marked in red color). **(B)** The Venn diagram of disease targets in 4 databases. Overlapping targets in at least two databases and dataset were considered as proteinuria-related targets (marked in red color). **(C)** The Venn diagram of potential therapeutic targets of Niaoduqing on the treatment of podocyte injury and proteinuria in DN. 16 overlapping targets was identified as the potential therapeutic targets (marked in red color with star).

1325, 574 and 115 DEGs were detected in the GSE1009 (Baelde et al., 2004), GSE96804 (Pan et al., 2018) and GSE111154 (Sircar et al., 2018), and the DEGs were further presented by volcano map and heat map, shown in Supplementary Figures S1–S3. The green and red nodes indicated downregulated and upregulated DEGs in the volcano map. In the heat map, red color nodes represented the high expression, while blue color nodes represented the down expression. We further obtained 3448, 95, 559 and 6 targets in “GeneCards,” “OMIM,” “NCBI” and “TTD” database using “diabetes nephropathy” as the keyword and “*homo sapiens*” as organism. After taking the intersection of targets appearing in at least two databases and datasets using Venn diagram, 986 overlapping targets was identified as DN-related targets (shown in Figure 2A). 3760, 239, 4 and 2 targets associated with proteinuria were found in “GeneCards,” “DisGeNET,” “OMIM” and “PharmgKB”. 213 overlapping targets, which appeared in at least two databases, were considered as proteinuria related-targets shown in Figure 2B. 2072 and 3 targets were found using “podocyte injury” as the keyword and “*homo sapiens*” as organism in “GeneCards” and “OMIM” database. After removing 1 duplicating gene, 2074 unique targets were defined as the related targets of podocyte injury.

Further taking the intersection of targets of Niaoduqing, DN-related targets, proteinuria-related targets and podocyte injury-related targets, we obtained 16 potential therapeutic targets of Niaoduqing on the treatment of podocyte injury and proteinuria in DN (shown in Figure 2C). The detail information about targets of DN, proteinuria, podocyte injury, and the overlapping potential therapeutic targets was shown in Supplementary Tables S5–S8.

### 3.3 Construction of the compound-ingredients-therapeutic targets network

**FIGURE 3:**
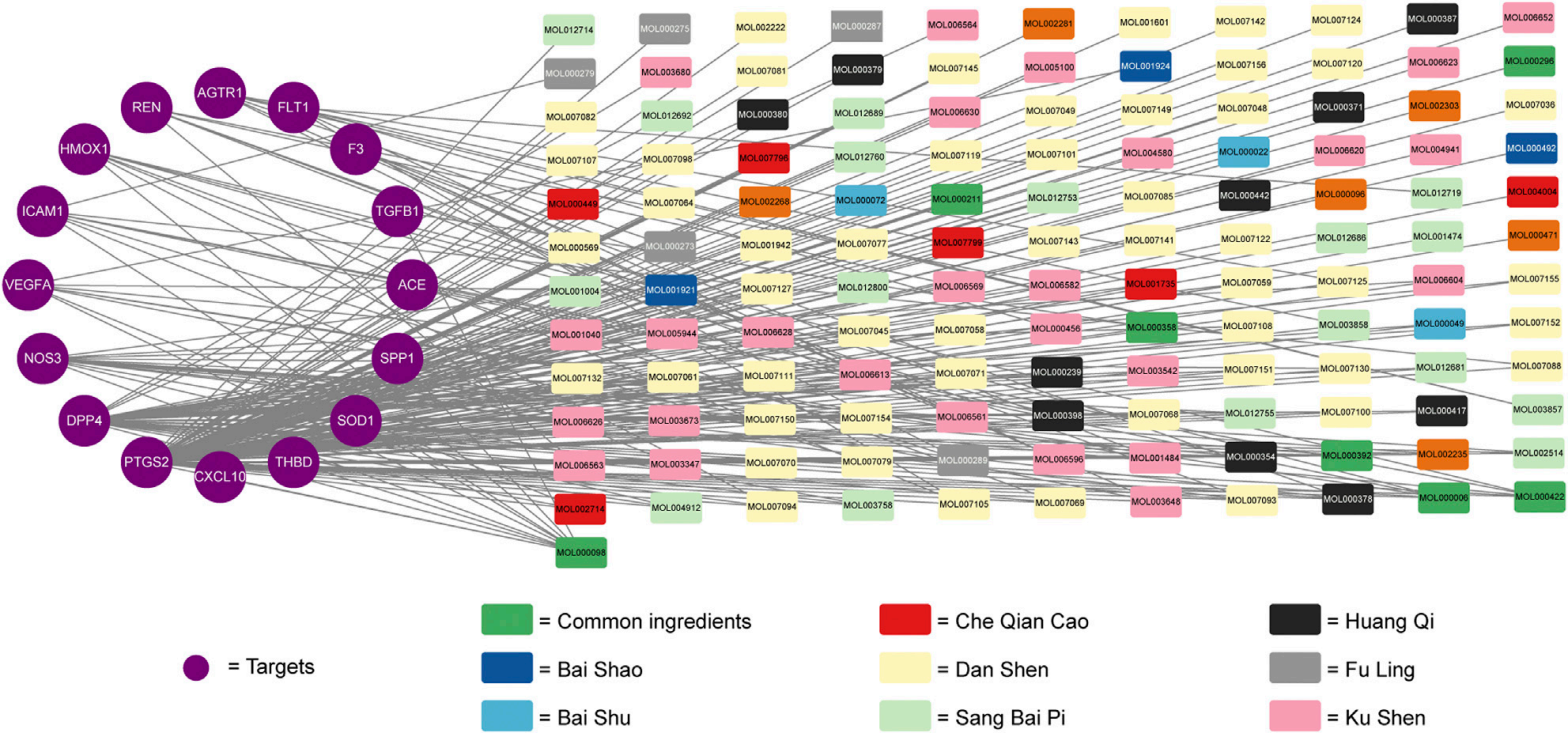
The compound-ingredients-therapeutic targets network of Niaoduqing on the treatment of podocyte injury and proteinuria in DN. The purple circle represented the potential therapeutic targets, while the colorful square represented the active ingredients from different compounds.

Cytoscape 3.6.1 software was used to construct the compound-ingredients-therapeutic targets network. The network was constructed by 133 active ingredients and 16 potential therapeutic targets, with 149 nodes and 234 edges. Different color was used to represent different compounds of active ingredients (Shown in Figure 3). The higher the topological parameters were, the more nodes connected to it. The data about topological parameters of the nodes was shown in Supplementary Table S9. In all of the active ingredients, quercetin had the highest topological parameters, following by luteolin, kaempferol, 7-O-methylisomucronulatol and danshexinkun D, which were considered as the core ingredients of Niaoduqing on the treatment of podocyte injury and proteinuria in DN.

### 3.4 PPI network and core targets screening

**FIGURE 4:**
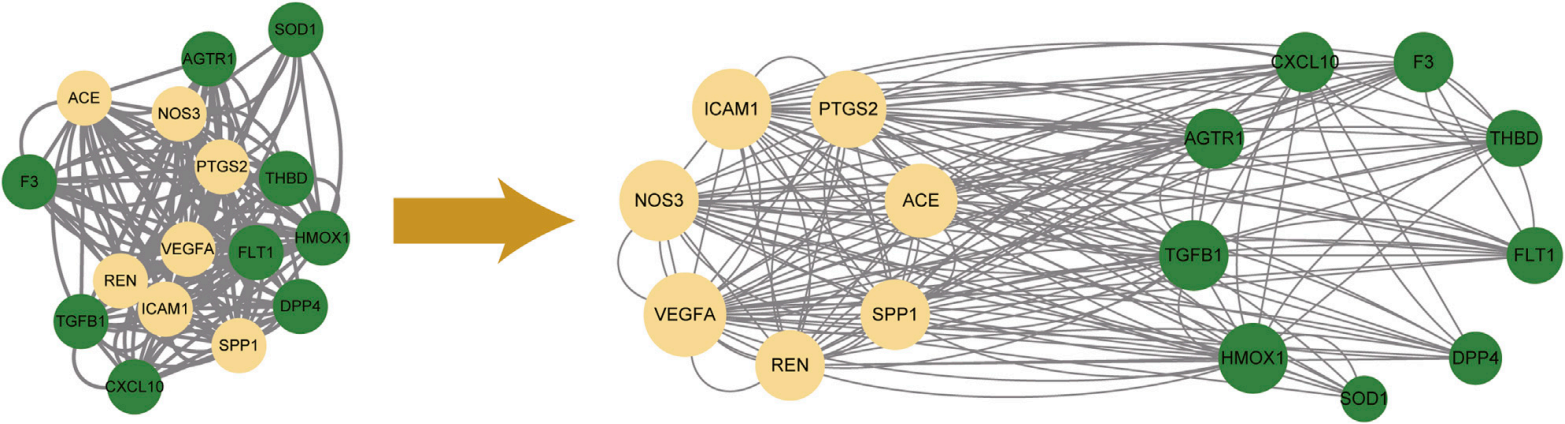
The protein-protein interaction network among 16 potential therapeutic targets. 7 core targets with the highest topological parameters were determined. Yellow nodes represented the selected targets (core targets), and green ones represented the remaining targets.

Importing 16 potential therapeutic targets into STRING database, we established an active ingredients-disease co-expression targets PPI network, which contained 16 nodes and 160 edges. We further screened targets with all three parameters higher than the median to construct the sub-network. We found that VEGFA, NOS3, ICAM1, PTGS2, ACE, SPP1 and REN were the core targets with the highest topological parameters (shown in Figure 4). The data about topological parameters of present network was shown in Supplementary Table S10.

### 3.5 GO and KEGG pathway analyses of potential therapeutic targets

**FIGURE 5:**
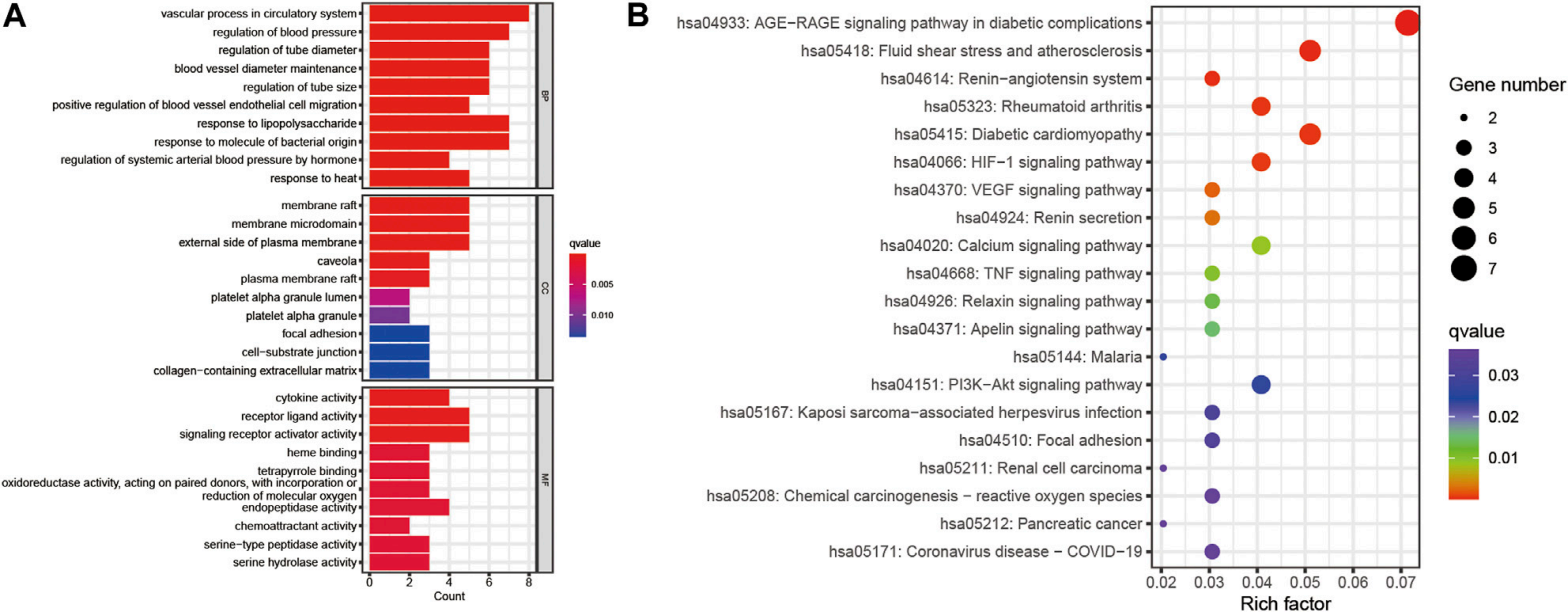
GO and KEGG enrichment of the 16 potential therapeutic targets. **(A)** The bar plot of top 10 GO enriched terms of BP, CC and MF function. **(B)** The bubble chart of top 20 KEGG enrichment pathways. The redder the bar, the smaller the *p*-value; the larger the bar, the greater the number of genes enriched in this processes and pathways.

GO analysis and KEGG enrichment analyses were performed based on the above 16 potential therapeutic targets of Niaoduqing on the treatment of podocyte injury and proteinuria in DN. The top 10 GO enrichment terms of MF, BP and CC were shown in Figure 5A. In addition, KEGG analysis was carried out to determine the key pathways of the overlapping genes (Supplementary Table S11), and the top 20 enriched signaling pathways were shown in Figure 5B. In all of them, AGE-RAGE signaling pathway in diabetic complications (hsa04933) exhibited the most significant enrichment, following by fluid shear stress and atherosclerosis (hsa05418), renin-angiotensin system (hsa04614), rheumatoid arthritis (hsa05323), diabetic cardiomyopathy (hsa05415) and HIF-1 signaling pathway (hsa04066). In addition to this, several inflammation signaling pathways and vascular barrier associated pathways were enriched, including VEGF signaling pathway (hsa04370), TNF signaling pathway (hsa04668), PI3K-Akt signaling pathway (hsa04151) and Focal adhesion (has 04510).

### 3.6 Molecular docking analysis

**FIGURE 6:**
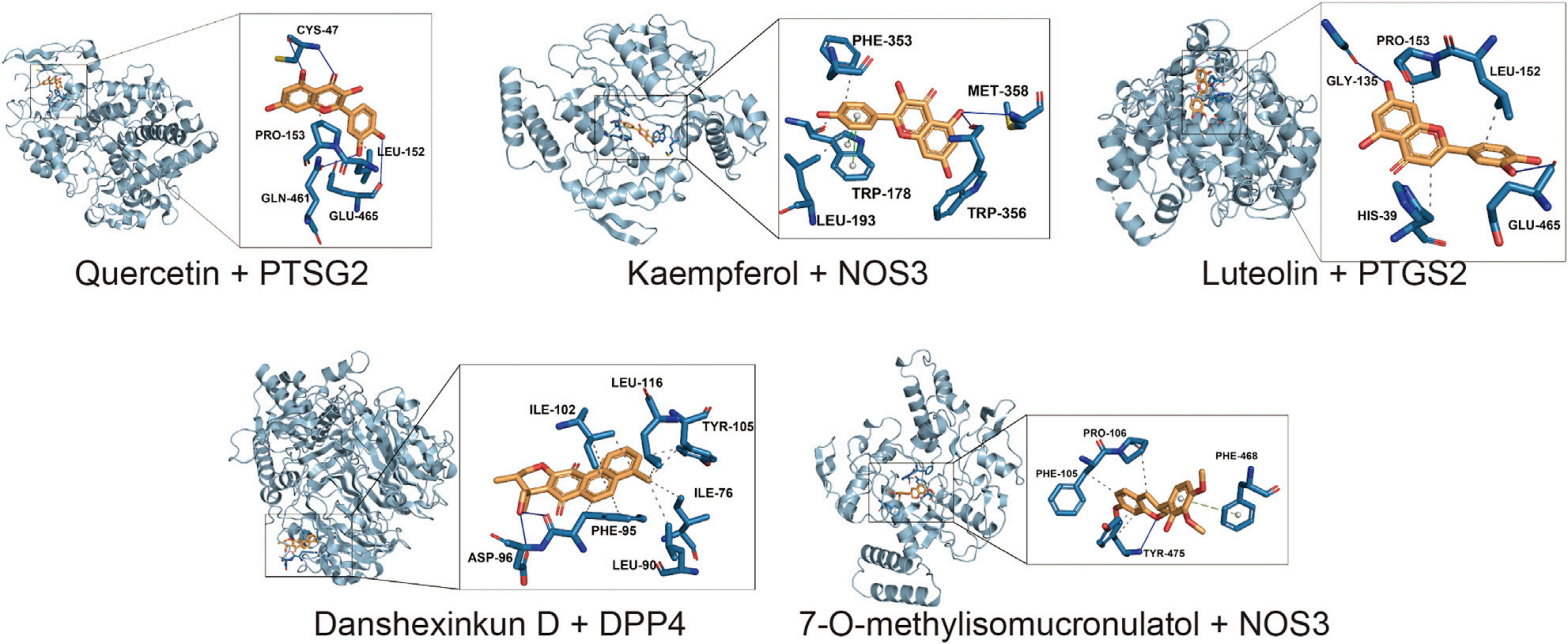
The result of molecular docking analysis between the core ingredients and the potential targets. The most binding results with the lowest binding energy of each core ingredients were shown.

The potential therapeutic targets of Niaoduqing were further docked with the top five ingredients through molecule docking analysis. We acquired their docking methods and binding energies, and found that the binding energies of all molecular docking were less than −5.5 (Shown in Table 2). PTSG2 and DPP4 interacted with all five core ingredients, while NOS3 interacted with four of them. The most binding results with the lowest binding energy of each core ingredients were shown in Figure 6.

**TABLE 2:** The binding energies results of molecular docking analysis.

Number	Ingredient	Symbol	PDB identifier	Binding energies (kcal/mol)
1	Quercetin	PTGS2	5ikv	−9.3
2	Kaempferol	NOS3	6pp1	−9.2
3	Luteolin	PTGS2	5ikv	−9.1
4	Danshexinkun d	DPP4	6b1o	−8.7
5	Danshexinkun d	NOS3	6pp1	−8.4
6	Danshexinkun d	REN	4s1g	−8.3
7	Quercetin	NOS3	6pp1	−8.1
8	Luteolin	HMOX1	1n45	−8.1
9	Quercetin	HMOX1	1n45	−8
10	Danshexinkun d	PTGS2	5ikv	−7.9
11	Luteolin	DPP4	6b1o	−7.8
12	Quercetin	DPP4	6b1o	−7.7
13	7-O-methylisomucronulatol	NOS3	6pp1	−7.6
14	Kaempferol	ICAM1	5mza	−7.6
15	Kaempferol	HMOX1	1n45	−7.5
16	Kaempferol	DPP4	6b1o	−7.5
17	7-O-methylisomucronulatol	PTGS2	5ikv	−7.2
18	Kaempferol	PTGS2	5ikv	−7
19	Quercetin	ICAM1	5mza	−7
20	Luteolin	ICAM1	5mza	−6.9
21	7-O-methylisomucronulatol	DPP4	6b1o	−6.8
22	Quercetin	CXCL10	1o7z	−6.8
23	Quercetin	SOD1	6fon	−6.7
24	Quercetin	TGFB1	4kv5	−6.2
25	Quercetin	SPP1	predicted	−6.2
26	Quercetin	THBD	1dx5	−6.1
27	Luteolin	VEGFA	1bj1	−5.9
28	7-O-methylisomucronulatol	F3	6r2w	−5.9
29	Quercetin	VEGFA	1bj1	−5.7

### 3.7 CCK-8 assay for cytotoxicity analysis

**FIGURE 7:**
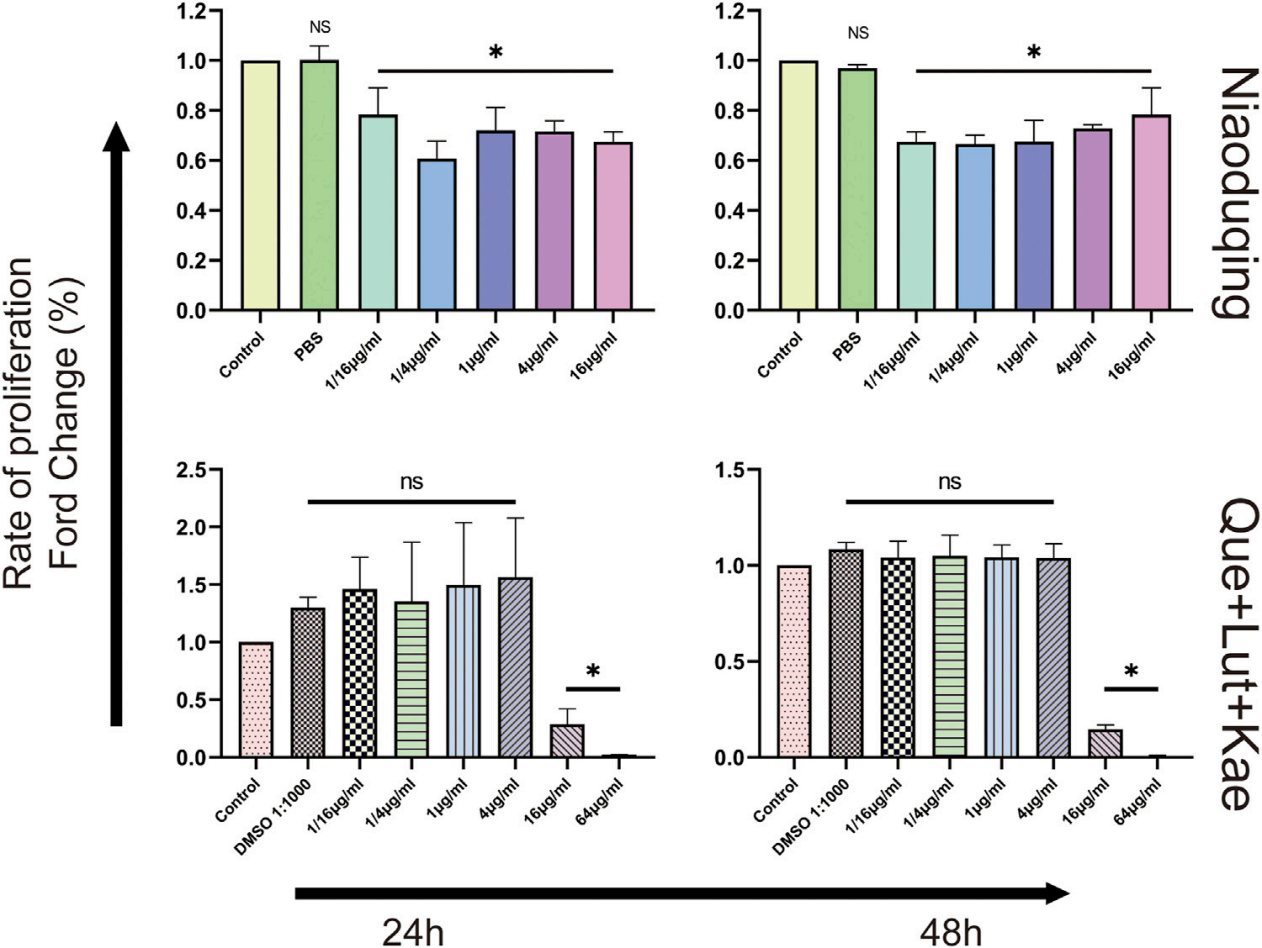
The cytotoxicity of the crude Niaoduqing granules solution and its purified core ingredients was detected by CCK-8 assay. The crude Niaoduqing solution showed obvious cytotoxic effect on MPC5 cells at all concentrations. No cytotoxicity of Que + Lut + Kae mixture was observed at the concentrations ranged from 1/16 μg/mL to 4 μg/mL. The data were represented visually with bar graphs. Data were presented as mean ± SD (*n* = 3 per group) of the representative data from three independent experiments. *p < 0.05, NS, non-statistically significant, compared with control group; analyzed by S-N-K test in ANOVA.

To determine the cytotoxicity and appropriate concentration of the crude Niaoduqing granules solution and its purified core active ingredients, differentiated MPC5 cells were exposed to drugs at different concentrations and CCK-8 assays were performed to detect the cell viability. As shown in Figure 7, the cell viability significantly decreased in Niaoduqing solution exposure group from the lowest to the highest concentration (1/16 to 64 μg/mL); therefore, the crude extract of Niaoduqing was not applicable for the *in vitro* experiment. In contrast, the mixture of three major purified Niaoduqing active ingredients (Que + Lut + Kae) did not show significant cytotoxicity up to 4 μg/mL (*p* > 0.05). Therefore, 1 μg/mL of Que + Lut + Kae was considered as a safe concentration and further used in our study.

### 3.8 *In vitro* validation of the predicted core targets

**FIGURE 8:**
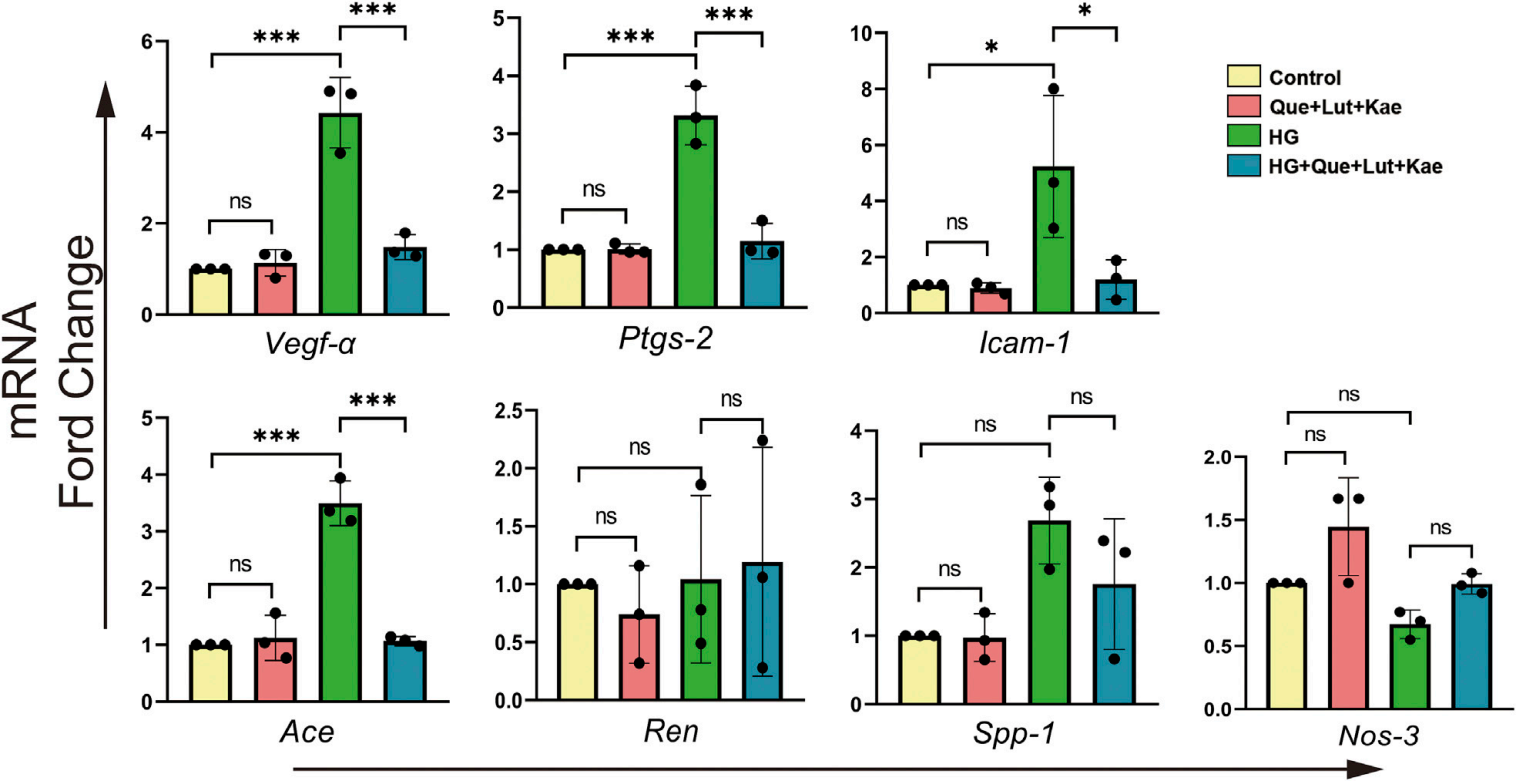
The effects of three major ingredients of Niaoduqing (Que + Lut + Kae) on predicted target genes. The treatment of Que + Lut + Kae mixture significantly attenuated the upregulation of the mRNA expression levels of *Vegf-α*, *Ptgs-2*, *Icam-1* and *Ace* in HG-induced MPC5 cells. The data were represented visually with bar graphs. Data were presented as mean ± SD (*n* = 3 per group) of the representative data from three independent experiments; *p < 0.05, **p < 0.01, ***p < 0.001, NS, non-statistically significant.

To validate the potential therapeutic targets of Niaoduqing in HG-induced podocytes injury model, RT-qPCR was performed to detect the relative expression levels of the above predicted targets. As shown in Figure 8, HG exposure significantly upregulated the expression levels of *Vegf-α* (***p < 0.001), *Ptgs-2* (***p < 0.001), *Icam-1* (*p = 0.026) and *Ace* (***p < 0.001) expression, but had no effect on *Ren* (*p* = 1.000), *Spp-1* (*p* = 0.052) and *Nos-3* (*p* = 0.524) expression. Compared with HG group, the upregulation of mRNA expression levels of *Vegf-α* (***p < 0.001), *Ptgs-2* (***p < 0.001), *Icam-1* (*p = 0.034) and *Ace* (***p < 0.001) were significantly attenuated in HG + Que + Lut + Kae group. There was no statistical difference of expression levels for all target genes between control and Que + Lut + Kae group (all *p* > 0.05).

### 3.9 Niaoduqing ingredients attenuate high glucose induced activation of AGE/RAGE signaling pathway

**FIGURE 9:**
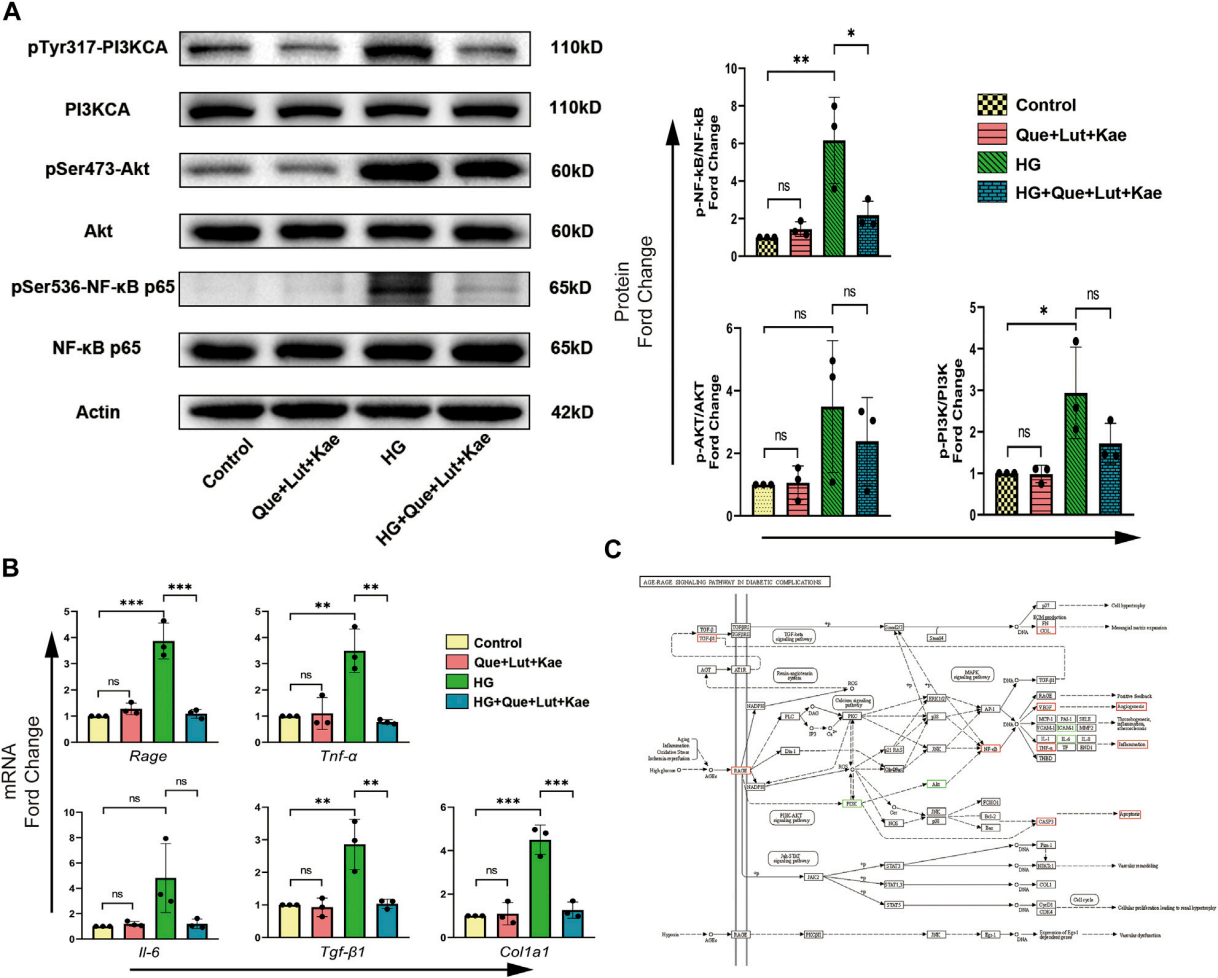
Three major ingredients of Niaoduqing (Que + Lut + Kae) participated in the regulation of AGE/RAGE signaling pathway in diabetic complications (hsa04933). **(A)** The protein expression and phosphorylation levels of PI3K, AKT and NF-κB in MPC5 cells were detected by Western blot. **(B)** The mRNA expression levels of *Rage, Tnf-α, Il-6, Tgf-β1* and *Col1a1* in MPC5 cells were determined by RT-qPCR. **(C)** The detail of AGE/RAGE signaling pathway in diabetic complications (hsa04933). Boxes represented the detected sites. Red boxes indicated the positive results, while green boxes indicated the inconclusive results. Data represented the mean ± SD of triplicate independent experiments; *p < 0.05, **p < 0.01, ***p < 0.001, NS, non-statistically significant.

To further investigate the potential mechanisms and evaluate the results of our network pharmacology analysis, the activity of AGE/RAGE signaling pathway in diabetic complications (hsa04933) was detected, which was the most enriched signaling pathway in KEGG analysis. As shown in Figures 9A, B, compared with the control group, the phosphorylation of PI3KCA (Tyr317, *p = 0.028) and NF-κB (Ser536, **p = 0.005), as well as the mRNA levels of target genes including *Rage* (***p < 0.001), *Tnf-α* (**p = 0.002), *Tgf-β1* (**p = 0.004), *Col1a1* (***p < 0.001) significantly increased in HG group. Strikingly, Niaoduqing ingredients treatment significantly reduced HG-induced the increase of phosphorylation of NF-κB (Ser536, *p = 0.024) and upregulation of the expression of *Rage* (***p < 0.001), *Tnf-α* (**p = 0.001), *Tgf-β1* (**p = 0.004), and *Col1a1* (***p < 0.001). Although Que + Lut + Kae treatment also inhibited HG-induced phosphorylation of PI3KCA (Tyr317, *p* = 0.244) and AKT (Ser473, *p* = 1.000), but with no statistical significance. The details of AGE/RAGE signaling pathway were shown in Figure 9C. Our results indicated that the therapeutic effect of Niaoduqing might be through the regulation of ARG/RAGE signaling pathway.

### 3.10 Niaoduqing ingredients protect against HG-induced MPC5 cell apoptosis

**FIGURE 10:**
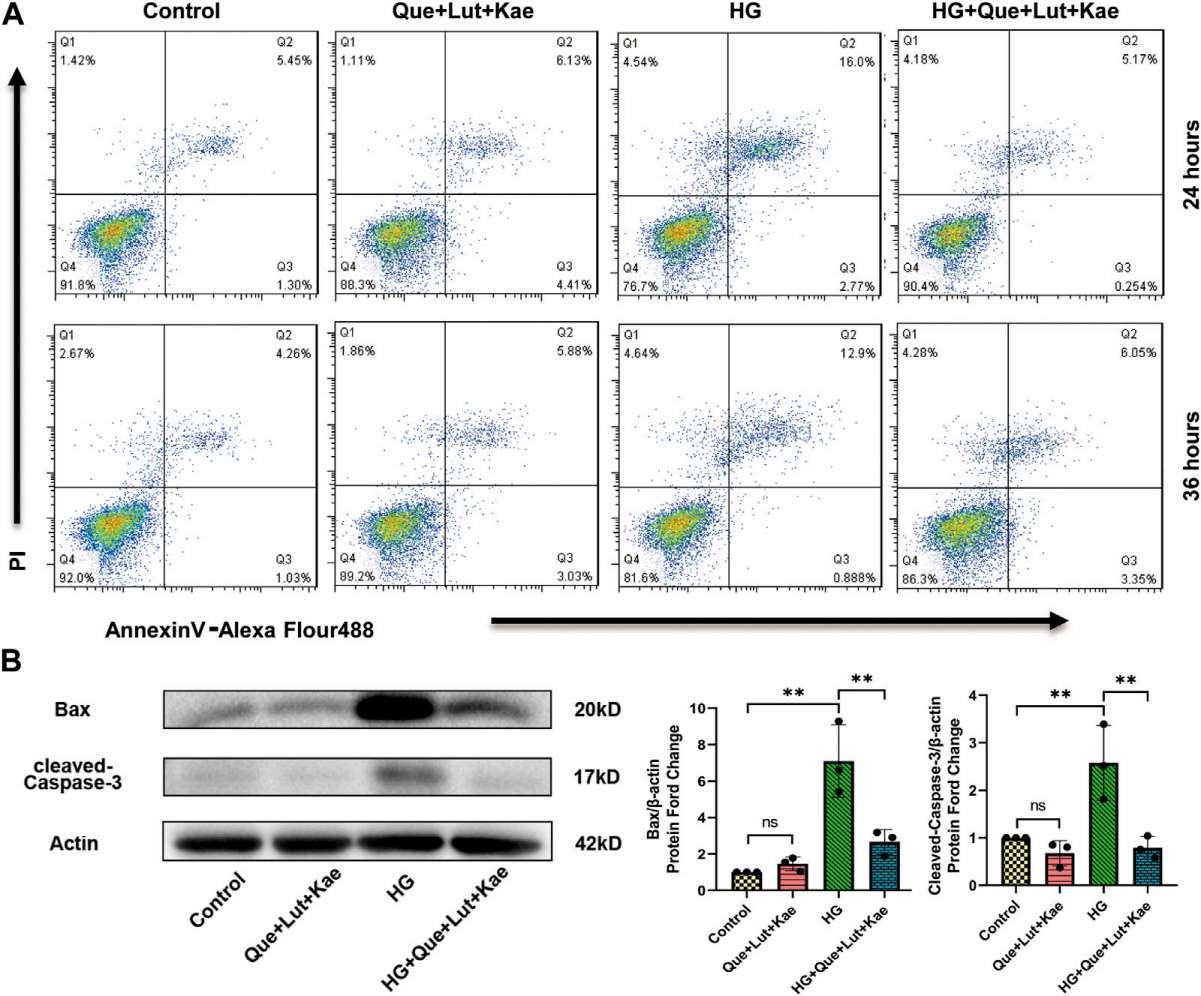
Three major ingredients of Niaoduqing (Que + Lut + Kae) reduced HG-induced MPC5 cells apoptosis rate. **(A)** Annexin V/propidium iodide staining and Flow cytometry were performed to determine the apoptosis rate of MPC5 cells. Q2 and Q3 indicated the early and late apoptosis, respectively. **(B)** The protein levels of Bax and cleaved-Caspase-3 in MPC5 cells were detected by Western blot. Data represented the mean ± SD of triplicate independent experiments; *p < 0.05, **p < 0.01, ***p < 0.001, NS, non-statistically significant.

Previous study showed that Niaoduqing had better effect on controlling proteinuria in patients with early stage DN (Zhao et al., 2022). Since podocyte damage is the major cause of proteinuria, Niaoduqing might be able to protect podocyte during early stage DN. Western blot and flow cytometry analysis were performed to further determine the protective effect of Niaoduqing against HG-induced MPC5 cell apoptosis. Flow cytometry data also showed that Que + Lut + Kae treatment significantly lowered the increase of HG-induced apoptosis rate of MPC5 cells (Figure 10A). As shown in Figure 10B, compared with the control group, Bax (**p = 0.001) and cleaved-Caspase-3 (**p = 0.012) protein levels significantly increased in HG-induced group, while Que + Lut + Kae treatment reduced HG-induced increase of protein levels of Bax (**p = 0.006) and cleaved-Caspase-3 (**p = 0.006). Our data suggested that Niaoduqing might reduce proteinuria through the protection of podocyte from high glucose induced apoptosis.

## 4 Discussions

Diabetic nephropathy poses a significant threat to the global public health and places enormous economic burden due to high morbidity, high mortality but poor control rate worldwide. To date, we still do not have effective treatment approach to stop or delay the progression of DN (Waanders et al., 2013). Podocyte is the major component of glomerular filtration barrier, and its injury would lead to the leakage of protein (proteinuria). Podocyte injury is considered as the major contributor to DN development, especially in the early stage. Several pathological processes, including persistent proteinuria inflammatory reaction, oxidative stress, vascular endothelial barrier injury and tissue fibrosis are all involved in the development of DN. Due to the complex mechanisms of DN development, treatment simply focusing on single target or pathway might be difficult to achieve satisfactory therapeutic results. Niaoduqing granule, a common clinically used TCM in CKD and ESKD, could treat diseases through a “multi-component, multi-targets and multi-pathways” way. However, the therapeutic effect and the underlying mechanisms of Niaoduqing on the treatment of DN and podocytes injury are still uncertain, especially of the early-stage DN.

In our network pharmacology analysis, 138 active components were considered as potential effective materials of Niaoduqing in podocytes protection and proteinuria reduction. Among the active components, various flavonoids were obtained, including quercetin, luteolin, kaempferol and so on. Flavonoids consisted of a large group of polyphenolic compounds of plant secondary metabolites that can be found widely in vegetables and fruits, and have numerous biological functions in the treatment of various diseases (Seo et al., 2019). In this study, quercetin, luteolin and kaempferol were determined as the most three core ingredient due to its highest topological parameters and the most related overlapped targets. Additionally, with the good docking score, all of them could be considered for the subsequent analysis of Niaoduqing. Due to the cytotoxic injury of the crude extract of Niaoduqing to MPC5 cells, the mixture produced by mixing the purified quercetin, luteolin and kaempferol on a 1:1:1 scale was used in the *in vitro* experiment. Although only three core ingredients could not fully represent Niaoduqing compound, they might be considered as one of the best alternative methods for clarifying the therapeutic effect of Niaoduqing *in vitro* experiment. Quercetin is an effectively ingredient in alleviating diabetes and related complications (Yan et al., 2022). It inhibits inflammation, oxidative stress, fibrosis, hyperglycemia and dyslipidemia to stop the progression of DN in a time-dependent and dose-dependent manner (Li et al., 2022b). Luteolin is considered as a potential medicine for kidney intervention in DN, which has anti-inflammatory, anti-oxidative stress and anti-fibrosis properties (Zhang et al., 2021). It also delays apoptosis, deletion, fusion and mitochondrial membrane potential collapse of podocytes, and maintains the normal filtration function of basement membrane through regulating the Nphs2 and NLRP3 inflammasome (Yu et al., 2019; Xiong et al., 2020). Kaempferol also has various biological functions. Except for anti-inflammation and anti-oxidative stress, it can enhance the release of insulin and GLP-1 to inhibit fibrosis of kidney in DN model (Sharma et al., 2020; Luo et al., 2021). Except for them, some other ingredients have been reported to correlate with podocyte protection. Wang et al. (2022) found that paeoniflorin can restore autophagy and inhibit apoptosis to protect podocyte from injury *via* inhibiting VEGFR2-PI3K-AKT activity. As Ertürküner et al. (2014) reported, mesangial matrix and podocyte has less damage and micro-albuminuria level decreased in the catechin-treated group compared with the untreated diabetic group, and catechin exposure even has the better protective effect on podocyte structure compared with ACEI. Xu et al. (2016) reported that matrine inhibits podocyte damage caused by adriamycin and improves renal function by maintaining the Th17/Treg balance. In addition, rhein and pachymic acid can ameliorate podocyte damage *via* regulating Wnt/β-catenin signaling pathway in DN mice (Duan et al., 2016; Chen et al., 2017). Multiple compounds and ingredients of Niaoduqing involved in the treatment of podocyte injury and proteinuria in DN, and flavonoids were considered as the most predominant effective constituents.

With the help of network pharmacology and experimental verification, we firstly identify some therapeutic targets of Niaoduqing in improving DN. Seven core potential targets with the higher topological parameters were screened out through PPI network construction and four of which were confirmed by *in vitro* cell experiment, including VEGF-A, ICAM1, PTGS2 and ACE. Our therapeutic targets mainly concentrate on the molecular process of vascular endothelial barrier, inflammatory reaction and RAS. All of those processes are involved in the pathogenesis of DN. Podocytes can produce VEGF-A, which is an important angiogenic factor and can induce vascular hyper-permeability and inflammation through interaction with endothelial VEGF receptor-2 (Tufro and Veron, 2012). The maintenance of normal VEGF-A levels is crucial for normal kidney structure and function, and either overexpression or insufficient of VEGF-A leads to kidney injury (Sivaskandarajah et al., 2012; Locatelli et al., 2022). ICAM-1 is one of the trans-membrane glycoprotein of the immunoglobulin supergene family, which is widely expressed on endothelium, epithelium, macrophage, and so on. ICAM-1 expression would be upregulated under high glucose condition, which mediates the infiltration of inflammatory cells into renal glomeruli and results in kidney damage (Miyatake et al., 1998; Galkina and Ley, 2006). Inhibition of ICAM-1 expression effectively blocks inflammatory cell infiltration into the glomeruli and alleviates kidney injury (Miyatake et al., 1998; Chen et al., 2016). PTGS-2, also known as COX-2, is one of the key enzymes in catalyzing the conversion of arachidonic acid into prostaglandin and leukotriene, which exacerbates local inflammatory reaction. ACE is the core compounds of RAS. The activation of RAS has been recognized as one of the key potential mechanisms of kidney injury, including DN. The most commonly used antihypertensive drugs, ACEI and ARB, are recommended and widely used in DN patients to inhibit the RAS and improve outcomes (Liu et al., 2020). In the present study, the increased mRNA expression levels of VEGF-A, ICAM-1, PTGS-2 and ACE in HG-induced group indicated that these genes may contribute to the development of DN and podocytes injury. The significant decrease of the expression levels after drug treatment suggested that the four hub targets may be the potential therapeutic targets of Niaoduqing in the management of DN and podocytes damage.

In KEGG pathway enrichment analysis, the potential molecular mechanism of Niaoduqing’s treatment of podocytes injury and proteinuria in DN was most enriched in AGE-RAGE signaling pathways (has04933). Two of the four hub targets (VEGF-A and ICAM1) were involved in this pathway. AGE/RAGE pathway has been demonstrated to be involved in the development of DN (Pathomthongtaweechai and Chutipongtanate, 2020). In the present study, some representative indicators of AGE/RAGE pathway were detected to evaluate its activity, including Rage, PI3K/AKT, NF-κB, VEGF-A, ICAM-1, IL-6, TNF-A, Caspase-3, TGF-B1, COL-1A1. Binding to their receptors RAGE, AGEs can activate downstream signaling pathways a, including TGF-β, p21-RAS and MAPK, and lead to indirect kidney injury (Wautier et al., 2001; Yeh et al., 2001). The upregulation of RAGE expression could be considered as the evidence of pathway activation. The downstream signal molecules of AGE/RAGE pathway, including PI3K/AKT and NF-κB, were detected. Although we observed obvious differences in the phosphorylation level of PI3K^Tyr317^ and AKT^Ser473^, no statistical differences were obtained due to some fluctuating individual values and low number of replicates. NF-κB is a crucial transcription factor involved in the regulation of inflammation, immune response and stress responses. The upregulation and activation of NF-κB is observed in preclinical DN models and kidney tissues of patients with DN (Opazo-Ríos et al., 2020). Targeting NF-κB is confirmed to be an effective method for DN (Opazo-Ríos et al., 2020). In present study, Niaoduqing effectively inhibited the activation of NF-κB in HG-induced injury model. What’s more, some phenotypes mediated by AGE/RAGE pathway were detected in our study. The increase of VEGF-A, ICAM-1, TNF-A and cleaved-Caspase-3 indicated the vascular barrier dysfunction, imbalance of inflammatory reaction and podocytes apoptosis in the HG-induced group. Niaoduqing alleviating those abnormal changes revealed its protective effect on podocytes in DN development. In sum, the administration of Niaoduqing effectively ameliorated podocytes damage caused by HG through partially regulating AGE/RAGE pathway.

Fibrosis of renal tissues is another crucial pathological feature of DN, especially in the end stage. The activation of myofibroblastic and inflammatory cells, extracellular matrix (ECM) expansion and collagens accumulation are identified as the key links of fibrosis development, in which EMT and endothelial to mesenchymal transition (EndMT) are the main sources of matrix-producing myofibroblasts (Srivastava et al., 2021). Inflammatory cytokines are the key profibrotic factors, including tumor necrosis factor-α (TNF-α) and interleukin-6 (IL-6) (Zheng et al., 2016). Many classical pathways have been reported to be closely related to kidney fibrosis, including Wnt signaling pathway and transforming growth factor β (TGF-β) signaling pathway. The loss of glucocorticoid receptor can promote fibrogenesis in kidney tissues *via* activating Wnt signaling pathway and interfering with metabolism of fatty acids (Srivastava et al., 2021). Fibroblast Growth Factor Receptor 1 (FGFR1), the endothelial receptor of fibroblast growth factor (FGF), is essential for combating EndMT, and the activation of FGFR1 signaling pathway has been reported to inhibit TGFβ signaling and TGFβ-induced EndMT (Woo et al., 2021). The deficiency of FGFR1 in endothelium can lead to serious fibrosis associated with EndMT (Li et al., 2020b), Compared with the control mice. Sirtuin-3 (SIRT3), one of the NAD-dependent mitochondrial deacetylases, also plays a crucial role in blocking tissues fibrosis *via* regulating TGF-β/Smad signaling pathway (Srivastava et al., 2018). The loss of SIRT3 can leads to induction of abnormal glycolysis and defective metabolism of kidney tissues, which is responsible for the progression of kidneys fibrosis in diabetes (Srivastava et al., 2018). In present study, we found that the expression of profibrotic factors (TNF-A) and fibrotic markers (TGF-β1 and COL-1A1) significantly increased after high glucose exposure, but the increase was inhibited by Niaoduqing ingredients treatment. These data suggested a therapeutic potential of Niaoduqing in alleviating podocytes fibrosis and inhibiting EndMT.

Recently, there are many potential drugs that have been proven to be effective against DN. As the commonly used anti-hypertension drugs, both ACEI and ARB show good effect on inhibiting kidney fibrosis, but their therapeutic effects are not completely consistent (Srivastava et al., 2020). The author found that DPP-4 and TGF-β signaling are identified as the downstream signals of ACEI in the treatment of kidney fibrosis, but both of them are not regulated by ARB. The anti-fibrotic effects of ACEI but no ARB, partly depend on N-acetyl-seryl-aspartyl-lysyl-proline (AcSDKP), which controls the metabolic switch between glucose and fatty acid metabolism. Another commonly used drug, sodium-dependent glucose transporters 2 inhibitor (SGLT-2i) is considered as a protector of kidney tissues in many kinds of kidney diseases. The application of SGLT-2i can reduce the progression of DN through promoting ketone body induced mechanistic target of rapamycin complex 1 (mTORC1) signaling inhibition (Tomita et al., 2020) The protective effect of SGLT-2i is also related to the inhibition of EMT and aberrant glycolysis (Li et al., 2020c). Compared with the individual application, the combination of SGLT-2i, ACEI and endothelin receptor antagonism can enhance their cardiac and renal protective effects in Type 2 diabetic model (Vergara et al., 2022). Tsuprykov O showed that dipeptidyl peptidase-4 (DPP-4) inhibitor, Linagliptin, has the comparable efficacy to ARB in preventing CKD progression in the 5/6 nephrectomy rats models. However, there may be differences in the underline mechanism of them (Tsuprykov et al., 2016). In addition, due to Warburg effect, which represents the abnormal shift of energy metabolism from mitochondrial oxidative phosphorylation to aerobic glycolysis, promotes fibrogenesis in kidney tissues, inhibiting glycolysis is considered as a potential anti-fibrotic method (Wei et al., 2019). As Wei et al. (2019) reported, both dichloroacetate and shikonin, two glycolysis inhibitors, effectively inhibited the process of renal interstitial fibrosis, and dichloroacetate was recommended because of its higher anti-fibrosis efficiency and lower toxicity. Those drugs are of concern and warrant further research, especially the comparison between Niaoduqing and those drugs.

There were some limitations in this current study. Firstly, whether the mixture of the purified quercetin, luteolin and kaempferol could fully substitutes for Niaoduqing granules is still unclear. Collecting animal serum containing Niaoduqing *via* serologic pharmacology method as previously described is an optional method to solve this problem (Lu et al., 2013). Secondly, missing data of animal experiment is another notable limitation. Further animal experimental validation using Niaoduqing is warranted. Thirdly, some of the disease therapeutic targets and pathways may be missed, because we only pay attention to the top predicted targets and pathways. Other predicted targets and pathways need to be further confirmed by both *in vitro* and *in vivo* experiments in the future. Due to the above limitations of present study, our results should be interpreted with caution.

## 5 Conclusion

In present study, we found that the active ingredients of Niaoduqing, including quercetin, luteolin and kaempferol, could ameliorate the podocyte injury in DN through multi-ingredients, multi-targets and multi-pathways method using network pharmacology method and experimental verification. VEGFA, ICAM1, PTGS2, ACE may be the major targets, and AGE/RAGE signaling pathway in diabetic complications (hsa04933) might be one of the core signaling pathways. Further evidence of *in vivo* experiment and clinical data are necessary to confirm our findings.

## Data Availability

The datasets presented in this study can be found in online repositories. The names of the repository/repositories and accession number(s) can be found in the article/Supplementary Material.

## Glossary

ACEI: Angiotensin Converting Enzyme Inhibitors
AGE-RAGE: Advanced Glycation End products—Receptor of Advanced Glycation End products
ARB: Angiotensin Receptor Blocker
AcSDKP: N-acetyl-seryl-aspartyl-lysyl-proline
BP: Biological Process
CC: Cellular Components
CKD: Chronic Kidney Disease
DEGs: Differentially Expressed Genes
DL: Drug-like Properties
DN: Diabetic Nephropathy
DPP-4: Dipeptidyl Peptidase-4
ECM: Extracellular Matrix
EMT: Epithelial to Mesenchymal Transition
EndMT: Endothelial to Mesenchymal Transition
ESRD: End-Stage Renal Disease
FGF(R): Fibroblast Growth Factor (Receptor)
GEO: Gene Expression Omnibus
GO: Gene Ontology
IL: Interleukin
Kae: Kaempferol
KEGG: Kyoto Encyclopedia of Genes and Genomes
Lut: Luteolin
MF: Molecular Function
MMP: Matrix Metalloproteinases
MPC5: Mouse Podocyte Clone 5 cells
OB: Oral Bioavailability
OMIM: Online Mendelian Inheritance in Man database
Que: Quercetin
PDB: Protein Docking Bank database
PPI: Protein-Protein Interaction
RAS: Renin-Angiotensin System
ROS: Reactive Oxygen Species
SGLT-2i: Sodium-Dependent Glucose Transporters 2 Inhibitor
SIRT3: Sirtuin-3
TCMSP: Traditional Chinese Medicine Systems Pharmacology Database and Analysis Platform
TIMP: Tissue Inhibitors of Metalloproteinases
TNF: Tumor Necrosis Factor
TTD: Therapeutic Target Database
