# Supplementary material for: Niaoduqing alleviates podocyte injury in high glucose model via regulating multiple targets and AGE/RAGE pathway: Network pharmacology and experimental validation
Source: Front Pharmacol. 2023 Feb 27;14:1047184. doi: 10.3389/fphar.2023.1047184 (PMC10009170; doi:10.3389/fphar.2023.1047184)
Supplement: Supplementary file 1 [file Table5.pdf]

Table S5 The targets list of DN related targets

	TTD	GeneCard	OMIM	NCBI	GSE1009	GSE96804	GSE111154	Overlapping
1	HTR2A	INS	PTPN22	VEGFA	HSPA12A	FOS	REG1A	CFH
2	D2R	KCNJ11	RFH1	IL6	PLCE1	MIR4521	PMP22	ACE
3	BDKRB2	GCK	CFHR5	PPARG	PLA2R1	DUSP1	CTGF	TCF7L2
4	COX	ABCC8	SLC41A1	HLA-DRB1	CDS1	MIR4256	PLG	SPINK1
5	SGLT2	HNF1A	MIA3	CDKN2A	HOXD1	SNORD3D	COL8A1	SERPINF1
6	CTGF	HNF1B	KLF11	IL10	BMP2	SNORD3B-1	FLNA	SLC12A3
7		HNF4A	HNFJ3	HMGA2	CLIC5	SNORD3B-2	FMO2	LPL
8		INSR	IL1RN	TCF7L2	TNNC1	SNORD3C	MT1A	GSTA1
9		PDX1	GPD2	HLA-DQA1	FAM153A	PDK4	CP	EGR1
10		NEUROD1	IDDM19	FTO	TSPAN8	CXCR2	LINC00924	ITGB6
11		ACE	IDDM7	INS	MME	MIR548D1	ADAMTSL3	DUSP1
12		PPARG	NEUROD1	KCNQ1	GMDS	HIST2H2AA4	PLGLA	HSPA1A
13		PAX4	CTLA4	IGF2	FGF1	HSD17B14	SPON1	COL3A1
14		WFS1	IDDM13	IL2RA	PAK1	SNORD3A	TAGLN	CHI3L1
15		IL6	IGAN2	HNF4A	FOXC1	LOC642799	ABCC9	NPHS1
16		TCF7L2	IRS1	HNF1A	THSD7A	HSPA1A	FHL5	POSTN
17		SLC2A2	CAPN10	HLA-B	SEMA5A	VTRNA1-3	SVEP1	UMOD
18		IRS1	PPARG	IAPP	C1orf21	NPIPA3	ACTA2	ANXA1
19		AQP2	CCR5	HNF1B	PTPRO	MIR1256	HGF	TYRO3
20		AVPR2	APPL1	CDKN2B	CRIM1	MIR548AD	SYNPO2	HBA1
21		KLF11	SLC2A2	SUMO4	IQGAP2	NPIPB5	ALOX5AP	CALD1
22		SLC30A8	IGF2BP2	ACE	F2R	MIR548D2	VSIG4	CD69
23		RETN	WFS1	ADIPOQ	ST3GAL6	PFN1P2	SRGN	PLG
24		VEGFA	IDDM23	IL2	EVI2A	NPIPB3	CD69	FBN1
25		ENPP1	IDDM18	INSR	LOC103344931	NPIPB11	SRPX2	CYP2B6
26		AVP	YIPF5	RETN	EXPH5	MIR548AI	ART4	SLC7A8
27		INS-IGF2	SPINK1	CDKAL1	MAP1B	MIR1254-1	C1S	BDKRB2
28		BLK	NIDDM4	PTEN	RABGAP1L	HSPA1B	AEBP1	CTGF
29		IGF2BP2	HFE	NLRP3	MTX2	NPIPA5	SAMHD1	SOD2
30		AKT2	ZFP57	NFKB1	FUT3	LOC613037	DIO1	AQP2
31		IRS2	IDDM1	PON1	PAM	LOC440354	LRP1	SPRY2
32		GLIS3	MBNP	IGF1	XPNPEP2	DANCR	C3AR1	IAPP
33		REN	PBCA	CCR5	F3	SLC7A5P2	C1orf162	LIPC
34		CEL	ITPR3	APOE	CEP152	CXCR1	IRAK3	UCP3
35		ALB	HMGA1	TNF	KBTBD11	NPIPL3	RASSF2	EPO
36		SLC2A4	VEGF	SPRY2	PFN2	SMG1P1	TLR4	PON1
37		MTNR1B	IDDM15	LEP	MGAT5	MORN2	CALD1	NEUROD1
38		PTPN22	IGAN1	SOD2	ARL1	BLVRB	CYP2B6	IL6
39		PON1	ENPP1	TP53	VEGFA	RNA5SP195	ITGA1	HNF1A
40		LIPC	TNDM1	TGFB1	KLK7	ZFP36	LPAR1	RETN
41		LMNA	IDDM21	MTHFR	TYRO3	LOC595101	HMGCS2	HNF4A

42	HFE	IDDM8	POU5F1	MYO1B	G6PC	PARM1	INSR
43	FOXP3	SUMO4	FOXP3	TGFBR3	LOC100506060	ANGPTL1	SUMO4
44	ADIPOQ	SOD2	HLA-DQB1	FAT1	LOC100190986	LYVE1	IRS2
45	UMOD	IL6	HFE	CDC42EP3	NEBL-AS1	MOXD1	INS
46	ZFP57	GCK	HLA-A	GMFB	MIR548AJ2	FMO3	IL1RN
47	SOD2	PON1	PEA15	PLA2G15	LOC101060449	CD163	HNF1B
48	PTPN1	EPO	MMP9	COL4A5	MIR54802	APOD	AVP
49	CTLA4	PAX4	IL1RN	PPEF1	SPDYE3	NEGR1	PPARG
50	EPO	BLK	TNFRSF11B	FAM50B	HIST1H2BD	RNU5D-1	IL2RA
51	CAPN10	SLC30A8	HIF1A	CR1	CKB	NPY1R	HFE
52	TNF	MAFA	APOB	ANXA2P1	LOC728734	NEXN	ENPP1
53	MIR21	GLIS3	TLR4	ADORA2B	MIR548AA2	FMOD	CCR5
54	APPL1	HNP1	NOS3	DGCR14	MIR1285-2	GUCY1A2	PTPN22
55	IL1RN	CEL	VDR	PRKG2	PCBD1	STEAP4	IRS1
56	XPNPEP3	IL2RA	CFH	ZNF264	S100A8	EDNRB	WFS1
57	MIR29A	IDDM24	CRP	HYAL1	ECH1	HSPA4L	ITPR3
58	TGFB1	IDDM17	LPL	SBSPON	SPDYE8P	TIMP3	DNAJC3
59	GPD2	TCF7L2	IL1B	PPAP2C	JUN	PDGFRA	SLC2A2
60	HLA-DQA1	CD151	LPP	DPYSL3	MIR548A3	LTF	LEP
61	HLA-DQB1	INS	CDH13	NPHS1	RNU7-29P	FSTL1	SDC2
62	LEP	KCNJ11	LPA	EGR2	SNORD66	LOC101060026	AGT
63	APOA1	ABCC8	SIRT1	FRY	RNA5SP312	LUM	TNNT2
64	AGT	MAPK8IP1	CCL2	GAP43	RNA5SP315	PLN	PODXL
65	SLC19A2	IDDM4	RASGRP1	SRGAP2	RNA5SP313	ADH1C	VEGFA
66	NLRP3	UCP3	STAT3	COLQ	SPDYE1	RGS2	APOL1
67	HYMAI	MTNR1B	NEUROD1	GPC4	ANXA1	ALPL	MME
68	AGTR1	NEDE	UMOD	TDGF1	NFIL3	CYP1B1	PROC
69	MT-TL1	IAPP	IL18	SYCP2	RNU7-53P	NDNF	ELMO1
70	HLA-DRB1	AQP2	EGFR	ERBB4	MIR297	FBLN5	CASR
71	MIR29C	NIDDM2	AGER	CLIP3	SAT2	C7	ATP6AP2
72	MC4R	HNF1A	ENPP1	TUSC3	DOCK9	MRC1	ERBB4
73	RFX6	PDX1	ELMO1	GAS1	LUST	DCN	AIF1
74	IL2RA	TBC1D4	AGT	PALLD	SPDYE6	ARHGDIB	VDR
75	PTF1A	SPRY2	AKT1	UGCG	S100A12	ADH1B	PECAM1
76	AGER	DNAJC3	PTGS2	PIN4	RNA5SP311	HIST1H2AC	NOS3
77	AKR1B1	IRS2	LCN2	RHOBTB2	RNA5SP314	F13A1	XBP1
78	ALMS1	COL4A1	IFNG	PREPL	RNA5SP317	KLRB1	PTPRO
79	SPINK1	IDDM11	EPO	KDM4A	SPDYE2	PCDH18	HLA-DRB1
80	SUMO4	LIPC	SERPINE1	AL109706	PRINS	FCER1G	LCN2
81	APOE	IDDM3	BDNF	PHACTR1	KIAA1191	GPR34	GC
82	CRP	FOXC2	AGTR1	GPNMB	S100A9	C1R	WNT5A
83	PLAGL1	HNF1B	AVP	GABPB1	MIR548F1	RBMS3	BMP7
84	MT-TE	ACE	NPPB	KANK1	MIR548T	NID1	TIMP1
85	NOS3	IDDM6	NFE2L2	DAG1	RNU7-47P	FBN1	STAT1

86	CCL2	IER3IP1	ACE2	CEP57	RNU6-38	SLAMF6	COL4A2
87	MTHFR	INSR	PTPN1	SLC22A4	MIR103A2	SPINK1	NAMPT
88	GCG	RETN	GSTM1	AIF1	RNU7-48P	COL1A2	TGFBR3
89	B2M	AKT2	ST6GAL1	LPL	RNU7-45P	SLC6A19	PDCD1
90	COL4A1	AVP	CXCL8	CEP112	RNA5SP187	SLC7A8	F5
91	CCR5	T2D3	MMP2	CRYGA	MED11	GLIPR1	ADM
92	IGF1	HNF4A	KCNQ1OT1	APOD	RNU7-7P	FRZB	XYLT1
93	IAPP	XPNPEP3	MTOR	LEPROT	LINC00342	LAMA2	PTEN
94	ITPR3	IDDMX	GSTT1	SERINC5	SPDYE5	TMEM252	ALOX5
95	STAT3	AVPR2	MBL2	GLUL	VNN2	ATP12A	GJA1
96	HFE-AS1		CST3	NTNG1	HNRNPU-AS1	IGLC7	BDNF
97	MAFB		LIPC	LOX	GUSBP3	HP	MYH9
98	SLC2A1		SLC2A1	NFASC	ANKRD36	LAPTM5	C4A
99	SERPINE1		CDK2	PTPRD	GSTA3	A2M	XRCC3
100	MIR192		SOD1	EIF3M	ERRFI1	AGMAT	CD2AP
101	TP53		IL17A	SECTM1	AKR7A3	PRELP	IGF2
102	CAT		SPP1	TLE4	MIR548X	CFH	RARRES2
103	LPL		ICAM1	HMGN1	SNRK-AS1	IGHA1	CDH1
104	APOB		HLA-DPB1	PPIP5K1	LOC100996472	STK17B	MSTN
105	LEPR		TLR2	DYNC1I1	CDH10	C1QC	ITGB3
106	ICAM1		REN	IGLL1	RNU6-8	MGP	MMP2
107	DNAJC3		EDN1	HOXC4	ANKRD36B	ABO	MMP7
108	GATA6		AQP2	SLC38A3	SMA5	SLC9A4	HES1
109	WT1		IRS2	LCN1	MIR548AC	MPEG1	SELL
110	MIR155		GSTP1	XYLT1	PTGS2	IGJ	MIR21
111	GAD2		ITGB6	ENPEP	LOC100506123	HCLS1	ALB
112	PTPRN		MIR21	DNAJB9	MIR548H4	ASS1	TREM1
113	MT-TK		IL4	UBXN2B	KIRREL-IT1	FXYD4	SLC4A4
114	CDKAL1		HMOX1	PTGER4	PCDHGA10	CA8	ESM1
115	IL10		MIR146A	CAPN3	MIR644A	IGHV3-33	VEGFC
116	IFIH1		C3	WDR62	MIR30A		S100A8
117	MIR377		GHRL	NXT2	RNU7-25P		KLK1
118	MIR17		CDKN2B-AS1	FXYD2	JUNB		ADAMTS5
119	CD79A		KL	USP46	BTG2		S100A9
120	DPP4		PVT1	GGT1	HBA1		CUBN
121	CAV1		TNFSF13	CHRNE	RNA5SP310		IGF1
122	NAGLU		TNFRSF1A	MYL9	RNA5SP316		GDF15
123	UCP3		ERBB4	DPF1	ACAP2-IT1		C3
124	CST3		SYK	THY1	MIR103B2		MIOX
125	MT-ND1		PLG	C1R	LOC727944		FN1
126	MLKL		MIF	ARHGEF15	EMP1		ZFP36
127	MYH9		IGF1R	OR2F1	RNU7-35P		EGF
128	PIK3R1		APOA1	TRAM2	RNU7-11P		PTGS2
129	LCN2		CYP2C19	ASPH	MIR548H2		APOH

130	NPHS1	SORBS1	BDNF	MIR548H3	CCL2
131	CISD2	PDCD1	KLHL9	GUSBP2	EPHX2
132	FTO	FGF23	SPOCK1	RNU6-83P	ANGPT2
133	RBP4	ABCB1	DCAF8	CA2	FABP1
134	MIR145	F2	HLA-DRB1	GUSBP9	VCAM1
135	HP	MPO	TENM1	LOC100996862	TGFBI
136	NPPA	CTNNB1	HMGCS2	FCGR3B	HP
137	CP	ALB	HTRA1	ETFB	MGP
138	VDR	CDH1	GJA1	NAIP	ALOX5AP
139	ADRB3	TNFSF10	BEX4	GOLGA8A	DCN
140	KCNQ1	CCL5	WASL	RNU6-3	TIMP3
141	IL1B	NOS2	MPP6	LOC100272216	CD163
142	CCN2	BCL2	CLEC16A	MAOA	TLR4
143	EDN1	RBP4	CBLB	TAS2R31	HGF
144	IL18	GDF15	FARP2	RNU7-24P	TCF21
145	IGF2	FLT1	MIPEP	RNU5B-1	NT5E
146	GHRL	ABCA1	COL4A4	HIST1H1E	CDH11
147	CDKN2A	HP	SIX1	GDF15	CR1
148	GAD1	CCN2	TAF4	MIR503	HBB
149	SPRY2	CYBA	WNT5A	OCLM	CXCR2
150	MAFA	HBA1	CDK14	AGAP6	PTGDS
151	NEUROG3	NAMPT	MFHAS1	PER3	SLC22A6
152	MMP9	HLA-E	SNCB	ATP13A3	DAO
153	MEN1	PPARGC1A	HIPK2	RNA5SP229	DPEP1
154	COL4A5	DPP4	CHEK1	SLC6A6	LOX
155	HMOX1	CXCL10	GEMIN2	MIR450B	ASS1
156	AKT1	CHI3L1	GULP1	MIR5047	APOD
157	FBN1	FGF21	TSPYL4	HBB	CYP1B1
158	AMBP	ITGB3	GABRD	RNA5SP320	FLNA
159	TLR4	F5	TCL1A	RNU7-13P	CP
160	APOC3	MAPK14	SERPINB8	SMCO3	SAMHD1
161	SLC5A2	FNDC5	TCF21	A1CF	COL1A2
162	NPPB	KDR	YWHAE	MIR548W	C1QC
163	IGFBP1	CD36	LOC101928625	DCXR	EDNRB
164	UCP2	TAP1	FAM98A	TREM1	SLC6A19
165	SOD1	MAPK1	CDKN1C	AGAP7	AEBP1
166	VCAM1	TNFRSF1B	IGFBP6	GUCY1A3	COL8A1
167	CETP	FN1	TNNT2	RNA5SP166	LUM
168	MPO	TLR9	ATP6AP2	MIR548AN	LYVE1
169	HAMP	MIR155	ZNF674	FPR1	ADH1B
170	PPARA	HAVCR1	COL4A3	SMA4	ALPL
171	CASR	PARP1	GYG1	RPL23AP32	VSIG4
172	TTR	VWF	CILP	ANXA9	HTR2A
173	FLT1	PTX3	THBS4	RPL36AP33	BLK

174	NPHS2	TIMP1	TUBB2A	AQP9	SLC30A8
175	CXCL8	APOL1	HSPB11	THRB-IT1	AKT2
176	CTNNB1	FOXO1	LPAR4	RNU6-80	GLIS3
177	CD40LG	NOTCH1	CLDN4	MIR548C	SLC41A1
178	GLP1R	S100A9	HS2ST1	RNU6-43	MIA3
179	SELE	CD4	CSF2	FLJ45340	KCNJ11
180	HHEX	FGF2	CD47	RNU7-40P	ABCC8
181	PCBD1	IL1A	GDPD5	GOLGA8B	GPD2
182	GGT1	HDAC9	HSPA2	SNORD105B	ZFP57
183	IL4	IFNL3	CTDSPL	CACNA1C-AS4	APPL1
184	VWF	OSM	ZNF101	RNU7-61P	MTNR1B
185	IGFBP3	CD40LG	HOXB1	RNA5SP20	GCK
186	PPARGC1A	TAP2	GPR162	RNU7-62P	MAFA
187	COL4A4	ANGPT2	MT3	HES1	CEL
188	COL4A3	MMP1	MCM6	FPR2	IGF2BP2
189	NAMPT	APOA5	MUC3A	FN1	FOXC2
190	IFNG	HSPB1	DST	ALPL	CTLA4
191	ADRB2	LTA	TOB1	SLIT3	PDX1
192	NOS2	CMIP	MAG	MIR3911	XPNPEP3
193	SST	APOC3	CORO2B	LOC399753	COL4A1
194	TNFRSF11B	RHOA	SEPT5-GP1BB	ALDH2	KLF11
195	IGF1R	STAT1	CREB3L2	RNU7-57P	PAX4
196	PRKCB	CDKN1A	OAS1	CYP2B6	AVPR2
197	MIA3	AHSG	BAG2	RNU6-21P	CAPN10
198	WRN	IL6R	TMED5	LRP2BP	HMGA1
199	GCKR	CNR1	PLOD2	TAS2R30	MEFV
200	AIRE	HGF	FAM168A	NR4A1	XDH
201	CNDP1	CAT	MYLK	GPR18	EDN1
202	HBA1	RELA	ZNF202	HIST2H2BE	IRAK1
203	FABP1	XRCC1	PDGFA	TMEM150C	NOS2
204	CFTR	GC	ARHGEF18	CRYL1	TRIB3
205	CDKN1C	SELE	CCDC69	MIR3671	SERPINA12
206	ACE2	SERPINF1	KIF11	MIR1299	MIR200B
207	PVT1	VCAM1	C1S	ESM1	SLC22A2
208	APOA5	FABP4	ZSCAN12	SNORA70G	PRKCB
209	MMP2	PLA2G7	ADORA1	LPL	C1QTNF3
210	IL2	UCP2	CHI3L1	NAP1L2	FOXP3
211	HIF1A	SHBG	ADAMTS3	ECHS1	APOE
212	MT-CO1	GPX1	TMED10	AK4P3	HSPD1
213	AOC3	CAV1	DDN	ANKS4B	SHBG
214	LPA	CLU	ITGA3	HBA2	MIR200C
215	FGF2	AKR1B1	CD55	RNA5SP343	BCL2
216	SPP1	SP3	ATP2A2	RNU7-10P	COL18A1
217	CCL5	SERPINA1	PODXL	CYB5A	CARD8

218	CDKN2B	SELP	CXCL3	GSTA5	CELA1
219	SHBG	MIR29A	PLSCR1	FAM180A	UNC13B
220	GH1	NPY	SLC26A4	MIR612	KDM6A
221	MIR140	TLR3	DPEP1	FXYD1	IL6R
222	G6PC2	RB1	CDH15	ANGPT2	ENHO
223	GHR	FCRL3	PLCG2	TPPP3	GSTP1
224	SIRT1	TNFSF11	OTC	LTBP1	LTBR
225	CEP290	HPSE	SULF1	SNORA22	NFE2L2
226	UCP1	HAVCR2	HSP90B1	PTGDS	IL23R
227	SLC12A3	YAP1	MUSK	C9orf66	KLF4
228	AR	RAC1	AP4S1	G0S2	PPARGC1A
229	KAT6B	LGALS1	PRKAR2B	RNU7-28P	CYP3A5
230	FGA	MIR34A	F5	CTHRC1	C5
231	THBD	CD163	PAMR1	RERG-IT1	CMA1
232	GSTM1	CD14	SUB1	PLK2	TNFRSF1A
233	HGF	ADAMTS13	GRIP1	ACMSD	OSM
234	HMGCR	EGF	CYP2C8	RNU6-60	B2M
235	ABCA1	MMP3	IGFBP2	SNORD7	OLR1
236	MIF	B2M	FAM49A	TPM1	AGER
237	APOA2	TGFBR3	CYP17A1	ASPN	TCF19
238	BMP2	THBS1	OR2H1	KLF7-IT1	IL18
239	BDNF	ADM	KDM5A	ALB	NPY
240	C3	MUC1	FAM20B	C14orf164	ADD1
241	FABP2	NOD2	BTC	ECM1	TRPC1
242	MME	ANGPTL4	TXNRD2	MIR570	GPX4
243	MIR146A	GJA1	CTSH	ALDH4A1	CMKLR1
244	MTOR	RBMS1	SMTN	SLC7A7	CDKN2B
245	PON2	ITLN1	DDX17	SNORA47	PSMB8
246	PTEN	CASP8	DDAH1	ZNF638-IT1	XRCC1
247	FN1	GNB3	LOC101930075	CMBL	ALPK1
248	PNPLA2	FBN1	ZNF629	MAGI2-AS1	FOXO1
249	FOXC2	DNMT1	PRKCI	PEPD	MMP1
250	BGLAP	PSMB8	ALOX5	LOC100303749	HLA-DQA1
251	IL1R1	PLAUR	ESRRB	MIR3120	SLC22A3
252	PLAT	CYP11B2	PARVA	VEGFC	MIR152
253	ADIPOR1	PROC	MXRA7	MIR186	UCP2
254	CXCL10	MALAT1	GTF2IRD2	VASN	MIR770
255	DIANPH	TCF19	MYH9	EGR1	IDO1
256	G6PC1	PRKCB	MYO5A	FBN1	ALOX12
257	IL17A	HLA-DPA1	CYP4F12	RNU6-45P	MMP3
258	CYP3A4	HSPD1	AC002310.7	FBP1	TRAF6
259	SELP	TNFSF13B	DPP6	BCL6B	OGN
260	KL	CASR	ACIN1	SMIM2-AS1	APELA
261	CLU	GRN	OPTN	HPD	MMP8

262	TFR2	CD40	ZNF185	LOX	MIR34A
263	ESR1	ADAMTS5	SLCO1A2	LOC100287497	NTRK2
264	APRT	PIK3R1	LSM3	BRE-AS1	IL10
265	GNAS	FABP2	ALDH1A3	PDZK1IP1	TLR10
266	MIR375	TXNIP	GAB2	ENAH	SH3YL1
267	NOD2	PRKCA	ING1	MIR548A2	TAP2
268	LTA	OLR1	CA5A	CTH	EGFR
269	CYBA	TIMP3	RCAN1	RNA5SP450	SOD1
270	ANGPT2	RARRES2	SLIT2	ZBTB20-AS2	CREB1
271	JAZF1	CX3CL1	ANXA1	RNF138P1	PDE5A
272	HAVCR1	ENG	PRKACG	RGS2	THBS1
273	MBL2	IL22	LRIG1	PRODH2	ACSL1
274	ADM	IL33	ZNF507	ADAMTS1	MIR155
275	F2	SMAD3	ATP10B	PI3	CDK2
276	MAPK8	NOTCH3	HTR6	MIR604	MIR22
277	TIMP1	BTNL2	ZBTB18	ACY3	CXCL10
278	PARP1	ADAM17	CD69	CRYAA	ARG2
279	AHSG	SOX2	TRIM44	CCND2	MIR140
280	TFRC	SLC22A3	RYR3	TGFBI	CDKN1A
281	PAPPA	TGM2	TCN2	LUM	PIK3R1
282	GFPT1	CX3CR1	44756	MIR30E	MIR199A1
283	HMGB1	PKM	APPBP2	SCARNA7	OIP5-AS1
284	ICA1	CYP3A5	SPOCK2	PCK1	HPSE
285	HLA-B	CD69	MEGF9	RNF152	LCAT
286	FGF21	IL21	SLN	EGF	MIR196A2
287	LMX1B	MEFV	PDLIM5	AK4	ST6GAL1
288	NFE2L2	TWIST1	ATXN10	GSTA2	PSMA6
289	HLA-A	BSG	RHBDF1	FRY-AS1	CX3CR1
290	NFKB1	PAPPA	HNRNPC	NPL	HLA-DRB3
291	LEPQTL1	TNNT2	AGT	SNORA12	FABP2
292	ACP1	GAS6	HPN	MIR548K	MIR483
293	FABP4	POSTN	AHNAK2	MIR421	TWIST1
294	CPE	S100A8	SACM1L	RNA5SP82	IL1R1
295	CD36	IDO1	FKTN	ASPA	RBMS1
296	SELL	MME	PDE4B	PCDH18	GNB3
297	KDR	MYD88	GPRC5A	EPHX2	HECW1
298	PTGS2	TRAF6	CDH1	COL15A1	DNASE1
299	PCSK1	CHGA	SCNN1A	CLEC4E	LPP
300	FGF23	SAA1	RAI14	MMP2	BID
301	CXCL12	CAMP	PLS3	RASD1	MIR146A
302	SERPINF1	EGR1	ARF6	CTXN3	CD4
303	STAT1	MYH9	SLC22A6	IGHJ1	ITLN1
304	MIR126	KLF4	ABLIM3	GIPC2	MBL2
305	TNFRSF1A	HSPA1A	AOC4P	RNU5E-2P	AKR1B10

306	NPY	TGFBR1	DLGAP5	LOC100996266	EXT2
307	RARRES2	ALOX5	TIPRL	ALDH6A1	NOS1
308	PRL	ADIPOR1	INHBC	ACY1	ABCG1
309	ATM	SPARC	CSF1R	SORD	SMAD3
310	DLK1	MIR27A	FCGR2A	MIR95	MIR204
311	TAB2	SLC12A3	COCH	MIR3916	MIR377
312	HLA-DPB1	TET2	CCL14	FABP1	SOD3
313	NPHP1	NPHS2	SPAG6	SELL	MIR192
314	GPT	CREB1	EIF3J	RNU4-2	MIR223
315	BMP6	FGA	NACC2	RNU6-82P	SKP2
316	MEG3	MIR223	B3GNT1	DPP6	PTHLH
317	LRP5	UCP1	PCK2	LINC00948	TNFRSF11B
318	IFT172	NPHS1	RP11-255C15.3	DPEP1	MIR27A
319	CAMK1D	ADAM10	G3BP2	PRKAR2B	PKLR
320	PRKAA2	MMP7	PCDHGB4	NAPSA	CD36
321	GNB3	CYBB	ATG4B	ADAMTS5	MIR320A
322	MMP3	GLO1	MXRA8	SYT11	C1QTNF5
323	MIR122	SERPINA12	TOP3B	NT5E	SLC9C1
324	PTX3	PON2	FLRT2	HRSP12	STAT4
325	SAA1	ANGPTL8	ADCY7	SNORA48	CD40
326	MT-ATP6	NOX4	RNF13	LOC100505664	TAP1
327	MIR143	TGFBI	ENO1	RNU5D-2P	CYP11B2
328	CYP27B1	UCP3	FGFBP1	FMN2	MIR23A
329	IGFBP2	SELL	CD200	KHK	FCRL3
330	GAPDH	SMAD2	HMCES	IGFBP6	MERTK
331	GPX1	NPPA	DBF4	MIR4263	NEAT1
332	TG	WNT5A	ZNF710	L3MBTL3	SERPINA1
333	THBS1	TFRC	TLK1	PDE1A	IFNL3
334	MTTP	KLF15	INPP5K	NID2	SELE
335	ADIPOR2	SPINK1	WHAMM	AQP7P3	ABCB1
336	LCAT	VEGFC	CRISP3	SFRP2	CNR1
337	TH	NEAT1	COL3A1	RASSF9	KLF6
338	CFH	SCARB1	MYC	SLC12A2	THBD
339	KNG1	THBD	PAWR	ABAT	IL17A
340	PRSS1	CPB2	GPR15	COL1A2	SLC22A1
341	CYP19A1	CASP9	KIF5B	RNA5SP160	KCNQ1OT1
342	ANGPTL8	IFNA1	UMOD	FABP5P1	MAPK14
343	FNDC5	SHC1	XBP1	RNA5SP260	MPO
344	TREX1	IL7	IQCJ-SCHIP1	SNORD75	PDGFB
345	APOC2	STAT4	CHRNA1	MIOX	HLA-A
346	BBS2	IL23R	PTPRS	PBLD	STAT3
347	IL6R	ITGAM	C1orf61	TGFB2	ORAI1
348	C4A	APOH	LGALS8	LOC728290	APAF1
349	TF	ETS1	KCNN3	LOC645638	TGFBR1

350	ARL6	XRCC3	COL9A3	SNORD14D	MCAM
351	SOCS3	SGK1	MAT1A	SNORD63	CAMP
352	SERPINB7	ANXA1	SRSF10	MRO	PTX3
353	PEA15	NOS1	NRIP1	MOXD1	CASC2
354	IGF2R	MIR150	MTDH	CCL21	SCARB1
355	MFF-DT	TLR7	CA4	FABP5P7	HAVCR1
356	TNFRSF1B	BMP7	SEMA3C	SNORD41	TXNIP
357	UTS2	SKP2	INPP5D	APOH	TLR3
358	PECAM1	MCAM	NOS2P1	GSTA1	ITGAM
359	ITLN1	CXCR3	SDC2	PVALB	MIR423
360	SORD	FABP1	CALD1	SLC7A8	PRKAA2
361	OLR1	MIR17	VSNL1	SLC12A3	PTPN1
362	PRKAR1A	JAG1	MSX2P1	FOLH1B	KIF6
363	SERPINA1	PTHLH	DLAT	TNNI1	LPA
364	ANGPTL4	AQP5	WWP1	APLN	LY75
365	MKKS	JAK1	RP1-40E16.11	CTSL2	MIR23B
366	MT-ND4	LCAT	WT1	EGOT	FGA
367	SLC41A1	HLA-DRB3	PHLPP2	FABP3	SYK
368	HPRT1	XBP1	GAL3ST1	MIR3975	IFNG
369	F3	S100A4	CITED2	EHHADH	FGF2
370	TPO	TRPC6	FANCL	RNA5SP268	CLU
371	APLN	PECAM1	OPRM1	RNY3P6	NTN1
372	PCK1	MIR200C	UGP2	TM4SF5	CX3CL1
373	MLXIPL	HSD11B2	SHOC2	ARHGAP19	IGF1R
374	CNR1	NRP1	DAPK2	AEBP1	CAV1
375	APOA4	MMP8	CCDC22	NR4A2	TNFRSF1B
376	SLC30A9	DDIT3	KIFAP3	MARCKS	LRRC7
377	FOXO1	ORAI1	GTF2B	F2RL2	CXCL1
378	TAP2	SMAD7	GFRA2	SNORA45	RELA
379	SGK1	SLC5A2	HOXD13	GLYATL1	HIF1A
380	CYP2C9	IL1R1	APOL1	IGHG4	MIR133B
381	NOS1	NTRK2	CCHCR1	INHBA	TNFRSF11A
382	PRKCD	UCHL1	LOC100996724	LRRC2	SPON2
383	FOXA2	CNDP1	TM4SF5	COL8A1	ADAMTS13
384	TXNIP	PRRC2A	FASTKD2	HSD17B2	MIR17
385	TNFSF11	ESM1	CYP1B1	PCOLCE2	NOX1
386	GIP	ARG1	GPR45	ITGA11	TLR9
387	COG2	MIR16-1	RAMP2	ANKRD10-IT1	IL33
388	GYS1	CXCL16	PANK3	PTH1R	PGC
389	GSK3B	PDGFB	USP9X	SYTL2	HLA-B
390	MIR223	NR1H3	CNTF	FUT6	KLF15
391	APOM	CYP24A1	SOCS7	AGMAT	JAK1
392	SOX2	MGP	NPTN	MFAP4	FABP4
393	CDKN2B-AS1	DCN	S100A1	GBA3	AKT1

394	MGAM	MSTN	RAD50	NPHS1	MIR130B
395	SOCS1	TRPV4	UBR2	RHCG	PON2
396	ATRIP	MIR192	CKAP5	PTN	MIR342
397	NR1H2	IRAK1	CCL19	COL6A3	MALAT1
398	CDKN3	ACTN4	TCF7L2	WDR72	MLXIPL
399	SERPINC1	MIR204	CXADR	FPR3	MIR217
400	MIR27A	TNFSF12	ZNF423	HAO2	SPARC
401	SREBF1	ATF6	CASR	ABCC2	APOA1
402	SEC61A1	COL3A1	IMPA2	CPXM1	MCF2L2
403	GC	XDH	RAG1	TNC	CDKN2A
404	MEFV	CXCL1	MTMR6	PPBP	NOTCH3
405	GDF15	MTRR	PCNXL4	POSTN	NOTCH1
406	CYP1A1	TNFRSF11A	CCL3	SLC6A13	CDKAL1
407	FGFR1	MIR196A2	SMYD5	ABCC9	GSTT1
408	SHH	MDK	RNF11	GLYAT	CD40LG
409	PLA2G7	TRIB3	ADM	CDH11	IL7
410	MALAT1	TREM1	ODC1	RBP5	PRSS1
411	PKHD1	ATF4	CACNG3	ADH1B	MIR216A
412	CYP1A2	CD86	CLEC3B	SLC36A2	ACTN4
413	PCK2	HDAC4	TIMP1	FBLN5	TKT
414	SOD3	ZFP36	LPHN3	LOC100505985	HSPB1
415	HSPA4	DUSP1	DDX51	PIPOX	KIRREL1
416	CD2AP	NOX1	SLC25A14	DDC	SHC1
417	PRPS1	C4A	NAMPT	SLC6A19	HSD11B2
418	GSTP1	MIR137	HOXA10-HOXA9	CCL2	CYP2C19
419	BBS1	SLC22A1	FUT2	AFM	TLR1
420	ALDH2	C5	LOC101930112	DPYS	MTRR
421	TSC1	CXCL5	INPP1	SAMHD1	MIR520H
422	HSPD1	LY75	LOXL1	USP2	KL
423	SERPINA12	SOD3	ARHGAP19	LOC101060277	MIR9-1
424	AGTR2	ICOS	GRIP2	EHD3	GHRL
425	APOL1	PRSS1	BCAR3	FAM151A	CST3
426	PCSK9	MIR182	LINC00837	THBS2	CRP
427	MUC1	COL18A1	HERC2P2	WDR49	MT-ND2
428	PDGFB	MERTK	AKR1C3	PRELP	MIR182
429	MT-ND5	ADD1	BACH1	HGD	TNFSF12
430	CDC123	C1QTNF3	CTNNBIP1	LINC00052	NPPA
431	LGALS3	FDX1	ADAMTS2	PAH	MIR424
432	MT-TS2	MIR140	OSER1	SNORD14E	TNF
433	AHI1	EPHX2	SOX15	SVEP1	IL1A
434	PPARD	MIR200B	GNAT2	RNU7-75P	CNDP2
435	GFPT2	BDKRB2	YBX3	RGS4	SGK1
436	OGA	ABCG1	DGCR11	ALDOB	SELP
437	BCL2	MIR23A	TLL1	ANGPTL3	SPP1

438	PTPN2	TLR1	HIST1H1T	TCF21	FDX1
439	FOS	GSTA1	HSP90AA1	CD248	F2
440	SLC30A10	ACACB	FCN2	PMP22	CTNNB1
441	MT-CO3	MIR22	NUP160	PROM1	GALNT2
442	NR3C1	NTN1	LOC102723551	ABI3BP	ACE2
443	HK2	CD80	HSPA5	C7	SELENOS
444	ONECUT1	CXCL9	GPR125	CFH	TGFB1
445	TAP1	PODXL	ITFG2	MIR4275	CHIT1
446	TTC21B	LRP2	PPP1R13B	IGLC7	CD86
447	MT-ND6	KLF6	EID1	CDH6	TNFSF13
448	POLD1	PSMD9	EFR3A	HPGD	SLC2A1
449	GIPR	MIR499A	MECP2	OLFML2B	KCNQ1
450	MT-CO2	PRKAA2	POU1F1	PLGLA	BSG
451	MAPK1	PKLR	VPS13D	GPR34	FN3K
452	CLCNKB	TRPC1	RDX	DAO	CD14
453	LHX1	ALOX5AP	PADI2	COL14A1	IL19
454	SP1	BID	DNAJC24	ACADSB	PSMD9
455	MIR34A	MIR424	EIF2B1	SNORD59B	GFPT1
456	TSPAN8	KLK1	GADD45A	COL3A1	HMOX1
457	MAPK10	MIR29C	TUT1	CR1	GLO1
458	CREB1	MIR199A1	RAD23B	VTRNA2-1	FLT1
459	ELMO1	EXT2	SLK	EDNRB	SMAD2
460	F8	CMA1	GSK3B	APOM	ANGPTL4
461	VTN	MIR27B	UBE3A	FMO3	HDAC4
462	BRAF	MIR152	HNRNPA0	MT1H	GSTM1
463	SCARB1	GPX4	B4GALNT1	SCARNA2	MAPK1
464	PIK3C2A	APAF1	TRAIP	MT1G	FTO
465	TLR2	MIR181A1	SH3BP1	VCAN	TNFSF11
466	SNRPN	HES1	IMPG1	VSIG4	CXCR3
467	IFT140	CD74	GJB1	PLG	TLR7
468	SURF1	MLXIPL	CAND2	MS4A6A	NPHS2
469	CD28	ALOX12	LOC101930303	MNDA	IL4
470	FASLG	CHIT1	TJP1	SNORD99	DEFA1
471	TIMP3	MIR133B	ATXN7L1	RNA5SP217	HLA-DPB1
472	HSD11B1	MIR130B	LANCL1	XPNPEP2	CD74
473	ATF6	DNASE1	DDX1	CYP4A22	TRPV4
474	ABCG5	AMBP	PPM1F	PPP1R3C	AKR1B1
475	SLC2A9	MIR9-1	ADCY1	NAT8B	LRP2
476	GPX3	IRAK4	CCL25	SLC2A2	CYBB
477	ABCG1	AKR1B10	DCAF4	C1QC	TRPC6
478	SLC2A3	ATP6AP2	PEX14	TYRO3	PLA2G7
479	MMP1	CUBN	CXCR2	SERPINF1	RB1
480	CRYAA	MIR320A	HYAL2	MMP7	SAA1
481	ARG1	SLC22A2	PRODH	DEFB1	MIR9-2

482	POLG	KDM6A	PNPLA2	KNG1	ADIPOR1
483	PTPN3	DEFA1	RPH3A	PSAT1	SP3
484	DDIT3	SDC2	GLOD4	VCAN-AS1	FFAR2
485	PIK3CA	SELENOS	CACNB2	C3	CCL5
486	PDCD1	PSMA6	CRIP1	CCL11	BTNL2
487	ST3GAL4	GSK3A	SSBP2	SLC13A3	TP53
488	CHI3L1	MIR217	PTPN21	TNFRSF12A	CASP9
489	CCK	SUV39H1	EMC2	TMEM207	CAT
490	ARAP1	MMP10	CYR61	LOC100131825	ADAM17
491	PRKACA	CALD1	EXOC3	CCL19	CNDP1
492	HBB	SLC4A4	DKK1	RNU6-79P	ACACB
493	ATRIP-TREX1	HECW1	SDC4	KCNJ15	MIR150
494	XBP1	MIR423	DRD3	CALB1	AHSG
495	PPY	PDE5A	NEK7	SLC7A9	POU5F1
496	BBS10	IGFBP4	MTHFD2	BHMT	NFKB1
497	GAST	MIR23B	HOXC5	TMEM174	ICOS
498	INPPL1	CMKLR1	TPX2	CPA3	SIRT1
499	ISL1	GFPT1	HMHA1	IGKV1D-16	MIR27B
500	EGF	ARG2	ASS1	DAPL1	IL1B
501	CDK4	GALNT2	CYP4F2	LYVE1	REN
502	TRAF6	COL4A2	UTF1	CCL18	FGF21
503	ADRB1	AIF1	CD24	PLVAP	NOD2
504	TGIF1	CARD8	SLC6A10P	ACSM2A	IL21
505	PDE11A	MIR483	SCAF11	BHMT2	TLR2
506	SLC22A1	TLR10	ATP8A1	CTSC	GPX1
507	CALCA	IL19	GPR65	METTL7B	GAS6
508	PLA2R1	DACH1	SLC12A3	CYP4A11	MMP10
509	IL6-AS1	CD2AP	LFNG	IGKV1D-33	HAVCR2
510	FRMD3	PGC	HIVEP2	ACSM2B	PLAUR
511	LRP2	MIR377	CBX2	PLN	MIR29A
512	IL1A	LTBR	NFE2L3	COLEC12	SOX2
513	SLC17A5	TYRO3	PRDX6	FOXQ1	CYBA
514	MYD88	CASC2	GHR	ITGB6	RAC1
515	MT-TQ	MIR216A	CDC14A	NAT8	FRMD3
516	ANXA5	MIR9-2	KIAA0195	IGKV3-7	NRP1
517	NKX6-1	MIR194-1	GAGE1	SNORD78	CDH13
518	ELN	MT-ND2	IFNAR1	IGKC	NLRP3
519	ALOX5AP	MIR342	S100B	CLDN8	MIR9-3
520	GJA1	MIR196B	EPS15	UGT2B7	AMBP
521	DDAH2	ACSL1	HSPA13	IGHV3-21	TGM2
522	NPHP3	ENHO	TNPO2	SLC47A1	NR1H3
523	CYP2D6	TKT	PNMT	CHI3L1	MTHFR
524	SCT	SLC19A3	CHD3	IGKV1-6	DACH1
525	ACACB	C1QTNF5	BPHL	AGXT2	CMIP

526	PSMB8	OIP5-AS1	PIP	SST	UCHL1
527	BAIAP2L1	MIR9-3	STX11	IGHA1	DPP4
528	MMP8	IL36A	PECAM1	IGKV1D-39	APOC3
529	NOX1	PTPRO	APCS	SLC22A8	NPPB
530	H6PD	DEFA3	ACSL3	IGHJ5	LGALS1
531	ACTN4	XYLT1	MUC3A	MS4A4A	HLA-E
532	REG1A	KIF6	SLC6A7	KLK1	ABCA1
533	FOXO3	FFAR2	GP1BA	CLDN1	TNFSF10
534	USF1	PARL	SLC1A7	TMEM52B	MUC1
535	FABP5	GSTK1	CMAHP	SLC4A4	MIR196B
536	CD163	OGN	ADAMTSL2	UMOD	CDKN2B-AS1
537	IL15	VNN1	ALPI	MIR21	CASP8
538	ALOX5	APELA	DNAH7	IGKV1D-27	APOB
539	RHO	DACT1	CDK1	IGKV1-9	MIR137
540	KLF14	FN3K	PROC	VCAM1	JAG1
541	IKBKB	SPON2	ACR	NELL1	CHGA
542	KCNJ10	UNC13B	DOC2B	IGKV3D-15	PAPPA
543	PAX6	MIR802	PHYHIP	KRT19	RHOA
544	BCHE	ALPK1	DLX4	FMO1	HLA-DQB1
545	PNLIP	CNDP2	PMCH	IGJ	CXCL8
546	PF4	NSA2	CLIP1	IGKV1D-42	HDAC9
547	PGF	LRRC7	LRRC23	APOC1	CPB2
548	MT-TW	MIOX	SLC20A2	IGKV1-17	MTOR
549	CASP9	CELA1	TOMM20	IGKV3-11	SLC19A3
550	PDE5A	KIRREL1	RPN2	SLC5A12	CCN2
551	SHC1	MIR770	PHGDH	SLC22A6	HLA-DPA1
552	TRPC6	NEWENTRY	CRYM	IGKV1-5	APOA5
553	DRD2	FRMD3	CPS1	ATP6V1G3	SMAD7
554	HSPA5	MIR520H	METAP1	IGKV3D-7	IL22
555	MT-TF	SH3YL1	CD27	C19orf77	ARG1
556	BMP7	SLC9C1	DNAH17	IGHV3-30	ADAM10
557	LYZ	MCF2L2	HSD17B3	SLC17A1	NOX4
558	GLA	ND5	MATN1	IGLV6-57	VWF
559	STX1A		LOC101930306	OR51E1	ANGPTL8
560	MT-TS1		FLNC	DEFA1B	AGTR1
561	FASN		44756	IGF1	TFRC
562	TLR9		ARHGAP32	TMEM27	ATF4
563	LDHA		MSTN	SLC27A2	HMGA2
564	CYP2C19		LOC100508689	CUBN	PARP1
565	TRIB3		SMARCA1	SLC34A1	DEFA3
566	GSR		UNG	IGKV2D-29	ENG
567	GSTT1		VIM	PLCG2	KDR
568	EP300		CBX1	RNASE6	MYD88
569	MAPK14		TATDN2	SLC13A1	MIR802

570	CPT2	FYN	IGKV1-27	RBP4
571	TGFB2	DCTD	IGKV3D-20	S100A4
572	MT-TH	CD2AP	IGKV2-28	CD80
573	CPT1A	FERMT2	IGHV3-23	DNMT1
574	APOA1-AS	STX7	IGKV2D-28	RASGRP1
575	CYP21A2	BIRC5		SUV39H1
576	ITGA2	LTC4S		SORBS1
577	BBIP1	NOTCH4		PEA15
578	LRP1	TNP1		GSTK1
579	KRAS	INHBB		UCP1
580	BTNL2	SF3B3		IL2
581	ANGPT1	CACNA1A		VNN1
582	SDHB	SLC6A3		DDIT3
583	PLG	NEBL		FNDC5
584	PODXL	JRKL		TNFSF13B
585	UBD	GTF2A2		TET2
586	NCF1	LAIR1		IL36A
587	CARS1	ATP9A		MDK
588	SMAD3	PTPN11		ICAM1
589	SH2B3	ADRB1		CXCL9
590	SIRT6	IRS1		ETS1
591	TMEM67	COG7		MIR181A1
592	SLC12A1	FBXL2		PARL
593	ADAMTS9	ZNF35		GRN
594	IL13	RHEB		MIR194-1
595	SIM1	GP2		MIR16-1
596	MKS1	SART3		NSA2
597	RAC1	ZIC2		PRRC2A
598	HNF4G	MORC3		MIR29C
599	PSMD9	LPAL2		AQP5
600	HMGA2	PHTF2		ATF6
601	SLC5A1	MYO1D		SERPINE1
602	TNNI3	MTIF2		IFNA1
603	BAZ1B	ITPR3		PKM
604	PBX1	NBN		CXCL5
605	SMAD4	NFE2L1		YAP1
606	LRBA	ACBD3		SLC5A2
607	C1QTNF3	COX6A2		MIF
608	MIR20A	HOXB7		GSK3A
609	ACHE	AVL9		IGFBP4
610	PLA2G1B	TIE1		LTA
611	PAX2	EZH2		MIR499A
612	ATP2A2	TRIL		PVT1
613	PRSS2	PTGDS		IRAK4

614	ENHO	MIR7110	CXCL16
615	AP4B1-AS1	RAD1	CYP24A1
616	TGFBI	TBCA	PRKCA
617	EDNRA	OPLAH	DACT1
618	CLCNKA	SNCG	FGF23
619	ICOSLG	KLK6	MMP9
620	TNFRSF25	ESYT1	ADIPOQ
621	INF2	PDIA6	GP1BA
622	TLR3	COL4A2	COL4A5
623	SCGB1A1	CSF3R	HLA-DRB4
624	CTNS	TOM1L2	ATXN10
625	CYP11B2	ADAM8	ITGA3
626	TIMP2	IL2RG	CDK1
627	MIR483	PLOD1	DRD3
628	RPGRIP1L	MTSS1	EMX2
629	PTCH1	CPNE6	F7
630	FABP3	CALU	RAG1
631	CD63	ZNF460	MAPK10
632	CASP1	GRAP2	NCAM1
633	PRKAB1	VPS26A	FGF1
634	CS	PPP2CB	UFL1
635	SLC22A2	PROZ	PARG
636	CD4	EGR1	FXYD2
637	PKLR	MAGI2	ATP7B
638	FGF8	KMT2D	RCAN1
639	NR1H3	INSL3	CASP1
640	NTS	PPFIA4	NCF1
641	CBS	ID2	FOXN2
642	CCR6	CHPF	IGFBP2
643	NOTCH3	KIFC3	CLDN10
644	MFN2	CEBPA	ESRRB
645	PDP1	SYNJ2	MYC
646	KEAP1	TSC22D3	ARSA
647	ADD1	ARC	SOCS3
648	MIR125A	NFATC4	GSK3B
649	TBL2	PSIP1	TRIP10
650	CASP3	GABBR2	SLC25A24
651	GFAP	STK4	ZFYVE9
652	CRH	NECAP1	TCTN3
653	ANGPTL3	ALG8	AK1
654	IRAK1	IGFBP7	FCGR2A
655	NPHP4	MCL1	KMT2D
656	MIR181A2	PDCD1	AXL
657	ABCA4	F8A1	BBS9

658	BCL7B	POM121L1P	THSD7A
659	RPS27A	IFT140	VPS4B
660	BSND	F7	STAB1
661	ALAD	RSBN1	CD28
662	CELA3B	GSTA4	HTRA1
663	ELANE	PRSS2	IFT88
664	CD34	AZGP1	FAM50B
665	F5	ICAM2	ODC1
666	JAK1	FOSL2	ADORA2B
667	SDCCAG8	MORC2	FAT1
668	GAS5	RARRES2	DMD
669	BBS7	PRKAR1B	OTC
670	NQO1	ABHD14A-ACY1	ABCB11
671	MSTN	GGT1	SOCS5
672	PTPRC	HNRNPDL	MAPKBP1
673	PPP1R3B	ZCCHC11	APOA4
674	OGG1	CCL8	SLIT2
675	FKBP6	COL11A2	GALNS
676	MIR210	ACE	HOXB7
677	PCSK2	ACTB	MT2A
678	MIR221	UAP1	NDUFA5
679	MIR423	GNE	UGCG
680	MIR15A	EIF4EBP1	UNC5B
681	TTC8	SYN1	ACVRL1
682	GTF2I	CACNA1S	MECP2
683	NOX4	BTG3	BMPR1A
684	PTGDS	RIT2	CD55
685	SELENOS	STAT1	ITGB4
686	OFD1	ANXA10	SKP1
687	HLA-DMA	CAMSAP2	KIFAP3
688	CLIP2	TOX4	DDAH1
689	SMARCAL1	PTPN7	CHEK1
690	KCNQ1OT1	AP3B1	PARVA
691	XDH	ITGB3	HSPB11
692	STYX	RCVRN	IGFBP7
693	L1CAM	BAX	CORO2B
694	PRTN3	CERS6	COL4A3
695	G6PD	ISLR	ELN
696	CFB	ZNF516	VIM
697	COL4A6	KRT85	CD27
698	CYB5R4	LINC00894	SLC26A4
699	AZGP1	FASLG	GPRC5A
700	SCARB2	MAPKBP1	KCNJ10
701	SYNPO	DUSP14	TRIAP1

702	MMP14	MYOF	S100A1
703	PPIA	RP11-15P13.1	MCL1
704	BBS4	CXCR5	HNRNPC
705	CD40	TAOK2	CXCR5
706	CCL3	FER	PDPN
707	ABCG8	GRIK2	FASLG
708	VPS33B	NAB1	ASGR2
709	TKT	NINL	OPLAH
710	KCNJ1	LTN1	COL4A4
711	EIF4H	ATG5	NDST2
712	SUFU	DLG5	NUP160
713	PLA2G2A	ITIH4	CCL8
714	GTF2IRD2	GPR3	SLC12A1
715	VPS37D	PCSK1	GTF2IRD2
716	RFC2	RASL10A	AZGP1
717	SRC	RRAS2	PRODH
718	KCNK16	NT5E	SACM1L
719	NKX6-2	BAI1	APCS
720	ITGAM	IGLJ3	WRN
721	NKX2-2	HIPK1	CEBPA
722	LIMK1	MANBA	CDKN1C
723	ALOX12	GALNS	IL7R
724	CCR2	POU2F2	ACTB
725	WDR19	LIMK1	CBY1
726	DUSP2	ZCCHC24	HIPK2
727	SMAD2	TRIM29	HPX
728	ZMPSTE24	RGS4	CKAP5
729	S100B	ATP10D	HPS5
730	TCF4	LUC7L3	SIX1
731	GREM1	DNAJC22	MORC2
732	ADA	VDR	PRSS8
733	MIR93	FMO1	F3
734	MGP	MDFIC	RECQL4
735	STUB1	FAM53B	PTPN13
736	IGF2-AS	TTC22	TRAF3IP1
737	GP1BA	OSMR	INSL3
738	BBS9	CELSR3	PAK1
739	SSBP1	HNRNPC	PCSK1
740	MIR217	SMARCA2	ARF6
741	PTHLH	H2AFX	PALLD
742	TNFSF10	CPQ	BIRC5
743	HLA-DMB	WNT11	SLC1A7
744	IL2RB	HBB	GHR
745	TERT	SAC3D1	GATA6

746	GATA3	KLF12	FOXA2
747	MST1	NEDD9	IFT140
748	NRP1	ZSWIM8	PCK2
749	HLA-DPA1	TEAD4	LIF
750	NOTCH2	SLC17A4	WDR62
751	PIK3CG	NDST2	PLA2R1
752	CFAP418	ZFYVE9	IGLL1
753	ABCB1	EP400	RPE65
754	CEP19	PDPN	LCN1
755	GTF2IRD1	ANKRD17	ADRB1
756	DNAJC30	EN2	FYN
757	BUD23	CFH	THY1
758	METTL27	ITGB5	CRCP
759	TMEM270	PCMT1	CCND1
760	ANGPTL6	CHST2	S100B
761	CFP	MAFF	BMP2
762	LOC105371046	SKP1	PTPN14
763	PTH	HCAR3	FASTKD2
764	CSN2	DAGLA	GAL3ST1
765	PROM1	IFNA14	BIRC2
766	CD59	ATP2B3	DAG1
767	PKD2	VILL	SLC9A3R2
768	MIR130A	MUC7	GP2
769	KLF4	LINC00893	WWP1
770	ADA2	NCF1	SMARCA1
771	CD14	PSMA2	RECQL5
772	AQP5	RECQL4	FGFBP1
773	PIGR	DGAT1	MYO1E
774	MNX1	SASH3	MIPEP
775	MIR25	PCMTD2	XCL1
776	TXN	ACVRL1	INPP5K
777	EGFR	RAB21	GGT1
778	RPE65	CSNK1A1	CRH
779	ROBO2	EPHA1	PRSS2
780	MEST	GPR135	CA4
781	VIPAS39	TNXA	AASS
782	HPSE	HNRNPA3	THBS4
783	CASP8	C4A	PDGFA
784	MT-TL2	SF3A2	SFI1
785	POU5F1	PRSS8	HSD3B2
786	INVS	CYP4F2	WNT11
787	ADAMTS13	RNF41	F2R
788	MEG8	CCDC64	ZNF423
789	ZFAND3	DNAJC3	TERT

790	CSF2	ASGR2	ENO1
791	PRKCZ	NRXN1	CDKN2C
792	CDK5RAP3	CYFIP2	CD47
793	MIR26B	IFRD1	WHAMM
794	MT-TV	ZNF529	AGXT
795	CTNS-AS1	MEP1B	UBE3A
796	THSD7A	GRIN2C	LIMK1
797	TRIM32	IFNA16	MEP1B
798	MET	HSPA1A	ITIH4
799	FTH1	RGS10	EPHB4
800	C1GALT1	KRT35	PDLIM5
801	C2CD4B	B4GALT2	RYR3
802	TYK2	DMD	ZNF189
803	MIR216A	PDIA3	PRDX6
804	CEP104	TRBC1	CRYM
805	BDKRB1	44756	PTPN11
806	MT-TT	DCTN5	YWHAE
807	FAS	VSIG4	NEUROG3
808	MERTK	HBE1	CDKN1B
809	PDGFD	TAPBP	FOXC1
810	ARL3	HAPLN1	TSPAN8
811	ENSA	EMX2	MUC7
812	TRB	FABP6	TNNI3
813	C2CD4A	MSH2	CXCL11
814	ERN1	ABHD3	AFP
815	POLR1C	TLE2	USP9X
816	OTX2	IARS2	PFN2
817	LIG4	FGF3	WT1
818	MIP	PALM	SDC4
819	EBF3	MMRN1	FCER1A
820	PCNT	PRRG2	ATG5
821	IFT88	KRT8	SRGAP2
822	APP	HGFAC	STX11
823	ABCB4	KRT20	GALC
824	CDH5	EFNA2	SLCO1A2
825	DNMT3A	GRIN2A	TJP1
826	FABP12	KCNK2	KPNA1
827	CISD1	FMO6P	ERVW-1
828	ESR2	PTPRM	HSP90AA1
829	TUG1	GLUD2	FERMT2
830	RHOA	CDKL5	CD34
831	MIR23A	AKR1A1	TUBB2A
832	CCND1	BRMS1	CD24
833	SLC5A4	LOC101929889	TG

834	NUP107	NCR3	OPTN
835	CC2D2A	SAMD4A	CHRNA1
836	TRPV4	MFGE8	TAT
837	CDH23	DPY19L2P2	CBL
838	POSTN	SLCO2B1	CLCN5
839	RTL1	LTK	MAGI2
840	KIRREL2	AQP8	ADORA1
841	AQP1	C17orf75	IFIT1
842	NR5A1	MYO1E	HSPA5
843	STAT5B	SLC16A7	PRRG2
844	JUN	LRP4	DDN
845	TULP1	MUC2	EIF2A
846	WDPCP	MEF2B	HSP90B1
847	CUBN	ADAM18	ALPI
848	H19	PRSS22	DKK1
849	ITGB2	AKR1C1	NOTCH4
850	PHLPP1	RNMT	INPP5D
851	COL8A1	HAO1	MAT1A
852	GALNT2	ATP4B	RNASEH2A
853	IL18BP	PNLIPRP2	BACH1
854	UFM1	FCER1A	SCNN1A
855	DDRGK1	CEBPE	IARS2
856	UFL1	CDKN2C	CCL25
857	HSD11B2	PRKRIR	CEP152
858	FOXM1	ACADM	CFHR3
859	ATP6V1B1	GPR56	LTC4S
860	COMT	MAP3K8	SMARCA2
861	SLC22A11	TDO2	RAMP2
862	TTC21B-AS1	CRH	SYNPO
863	TNXB	GABRE	CCL3
864	TRPV1	EPHB4	ATP2A2
865	ITGB3	NR1I3	SPINT2
866	COQ2	XRCC3	BAX
867	FOXA1	HIRA	HIPK1
868	IL12B	SPINT1	PNPLA2
869	MAPT	EIF2A	CSF2
870	FCGR2A	GAS2L1	SFRP2
871	TGM2	WASF3	APOM
872	TSC22D1	TMEM5	CCL11
873	LOC111365141	RP11-79P5.2	CXCR1
874	CLTRN	KCNS3	IGKC
875	ZNHIT3	FAM131B	APLN
876	NELL1	SAG	KNG1
877	NR3C2	BIRC2	SLC7A7

878	VANGL2	LSS	VCAN
879	SERPINA3	PLEKHO2	S100A12
880	KLKB1	CRTC1	SLC13A3
881	KIF7	HMX1	HSD17B14
882	GLIS2	RAMP1	APOC1
883	TEK	PITX3	TGFB2
884	HSPB1	CPNE1	CALB1
885	PSTPIP1	CRLF1	ANGPTL3
886	PRRC2A	TMEM30B	KHK
887	SNHG6	TNNI3	SLC22A8
888	MIR19A	LIF	FABP3
889	BDKRB2	BMP7	SLIT3
890	CD8A	MAP2K4	TNFRSF12A
891	MT-ND2	GZMH	FOS
892	MICA	MYL5	SLC7A9
893	AGMO	NAALAD2	LINC00342
894	APOC1	CYP2A13	JUN
895	AKR1B10	XPO1	PTH1R
896	RPGR	SS18	ALDH2
897	MT-TI	NOL4	SLC6A6
898	SAMHD1	TRIM22	LTBP1
899	SLC37A4	TAT	ABCC2
900	NGF	BBS9	NAIP
901	AQP3	MYLPF	SST
902	PI4KA	DSG2	FCGR3B
903	MT-TP	FBP1	PCBD1
904	GRB10	EI24	ETFB
905	ENG	DAO	CLDN1
906	NAT2	HOXA2	PLVAP
907	MT-TN	NDUFA5	ERRFI1
908	FCAR	LDB2	NAT8
909	RDH12	TNNI1	DEFB1
910	KCNK5	GRK5	MIR30A
911	BAD	CLDN10	SLC34A1
912	BMP4	ATXN1	MIR103A2
913	XYLT1	RECQL5	PCK1
914	PALLD	CETN1	CYP4A11
915	MT-TA	CBL	FAM151A
916	MCF2L2	CNIH1	MFAP4
917	BAX	MAL	TNC
918	PKD1	BST1	CRYAA
919	ABCG2	CD70	NELL1
920	MIR181A1	ASMT	THBS2
921	NFKBIA	LCN2	CA2

922	NEU1	TSPYL5	SORD
923	CD55	LIPT1	DDC
924	CHGA	TAZ	PROM1
925	SNHG18	44756	CCL21
926	TWNK	WFS1	HSPA1B
927	PRKCSH	IFT88	CCND2
928	RBP1	KCNJ10	ALDOB
929	PDGFA	MAGEB2	MIR95
930	CCKAR	ABI1	LRP2BP
931	OSM	SMG7-AS1	MIR186
932	NEAT1	ELN	FMN2
933	TGFBR2	SOX5	PEPD
934	SI	DYRK1A	CTSC
935	XYLT2	APOA4	A2M
936	PRPF8	ST8SIA3	LRP1
937	HLA-C	AASS	LAMA2
938	GSK3A	CCDC6	NID1
939	LRPAP1	OSBPL2	ITGA1
940	HLA-DRB3	GATA2	LPAR1
941	MAPK3	PAPSS1	NPY1R
942	IQCB1	NUCB1	FMOD
943	MIAT	KIR3DL1	REG1A
944	FCGR3B	SARAF	C3AR1
945	AHR	FGF9	TAGLN
946	COQ4	AMOT	LTF
947	ZDHHC24	PTPN22	ACTA2
948	MIR199A1	SERINC1	CCL19
949	SLC22A3	CLEC10A	RGS4
950	NR2F2	FAM214B	NFIL3
951	TNFRSF10A	PCDHA9	DPP6
952	FXYD2	FXYD1	FPR2
953	HDAC9	C9	DPYS
954	MASP1	MCF2L	PRKAR2B
955	GAS6	CELP	PLCG2
956	DNM1L	UNC5B	ACSM2A
957	GANAB	CYP2B6	IGFBP6
958	PRDX1	FOXA2	FBP1
959	PPIG	HPX	TNNI1
960	MIR23B	FOXN2	FXYD1
961	IL33	HNRNPA1	XPNPEP2
962	MIR200B	SNX17	ARHGAP19
963	STIL	KRT18	FMO1
964	ATF4	BFSP2	TM4SF5
965	COL8A2	RFTN1	HMGCS2

966	VWA2	ABHD2	MRC1
967	CYP24A1	NDN	C1S
968	MIR22	CFHR3	C1R
969	NR5A2	LCK	ATP12A
970	KCNN4	MIR6872	IGLC7
971	CNDP2	HAP1	PCDH18
972	NRAS	FIP1L1	FMO3
973	TGFBR1	CYP51A1	PLN
974	NDUFS4	PTPLB	C7
975	ALDOB	KPNA1	IGJ
976	IL7	CDH12	RGS2
977	MIR106B	C6orf62	GPR34
978	GNAS-AS1	DHODH	IGHA1
979	C12orf29	AXL	AGMAT
980	JAK2	CNNM2	FBLN5
981	HSPA1A	GJA8	SVEP1
982	GZMB	PPL	ABCC9
983	EXT1	PI15	PLGLA
984	GCC1	APOBEC1	PRELP
985	MT-ATP8	HINFP	PMP22
986	PRKCA	DIAPH2	MOXD1
987	DGKE	FOLR2	
988	FGB	HCRT	
989	DCN	ITGAD	
990	AQP11	KRT83	
991	TMEM231	GJA9-MYCBP	
992	SLC22A8	STARD8	
993	BBS12	PTPN14	
994	MAP2K1	HSD17B4	
995	IFNA1	SULT2A1	
996	MIA2	ABCC3	
997	STAT4	LOC100288974	
998	SNORD116@	MT2A	
999	TMEM216	RPA3	
1000	H2AC18	TRMT1	
1001	GLO1	SLC6A5	
1002	CD274	TSPAN1	
1003	BBS5	IGF2	
1004	ROBO1	CCNF	
1005	IL7R	SFI1	
1006	INPP5E	CFH	
1007	PTPRO	FGF18	
1008	MIR34C	PAPOLA	
1009	CD38	KBTBD2	

1010	CDH1	PNMA2
1011	CHIT1	BSN
1012	DYNC2H1	SOCS3
1013	COL4A2	DOK2
1014	RPL36A-HNRNPH2	WRN
1015	PRCD	RABGGTB
1016	ITGB1	SGMS1
1017	SOX3	KCNJ5
1018	MT-ND3	TLN2
1019	MAF	CRYGD
1020	SLC26A4	TBX10
1021	MIR20B	ERVH-1
1022	PDGFRB	CCNB2
1023	CTSB	FLNA
1024	HSPG2	MBOAT2
1025	TIMM44	APOOL
1026	SLC9A1	ARSA
1027	PRKAG2	YWHAZ
1028	NSA2	SLC25A24
1029	CSF3	HPS5
1030	EPHB4	TLX2
1031	MED13L	AKAP5
1032	IMPDH1	SUMO2
1033	GHSR	CTGF
1034	MIR196A2	PPM1H
1035	SUCNR1	SERPINI1
1036	SORBS1	DTX2
1037	ERBB2	CXCL11
1038	APOH	ATP7B
1039	COL1A2	ADD3
1040	ROM1	PRKG1
1041	NOTCH1	ZNRF4
1042	HLA-E	KIAA0485
1043	SYK	CRCP
1044	SDC3	ZER1
1045	CRB1	TMEM259
1046	EIF2S1	DDX10
1047	ERBB4	SIK1
1048	CA4	SET
1049	CCR1	PAPPA2
1050	TBX21	CASP1
1051	EPHX2	SFTPB
1052	S100A8	HAL
1053	MX2	ZNF189

1054	SLC9A3	C16orf80
1055	SLC19A3	PRKACB
1056	CXCL9	HBA1
1057	IL5	INSM1
1058	IGFBP7	KIAA0101
1059	NUDC	CP
1060	CNTN1	SLC12A1
1061	TBX4	VCX3A
1062	TMEM237	TREH
1063	TNNT2	PGAM2
1064	IRF3	FNBP1L
1065	BMP3	CLCN5
1066	MMP7	STK16
1067	NID1	FADS2
1068	NLRP1	KIF2A
1069	ADH1B	LOC101929857
1070	HSPA1B	RLN1
1071	NSD1	COL17A1
1072	UCN3	SRSF7
1073	KCNJ3	S1PR2
1074	LOC108251801	GRAMD1B
1075	SAA4	TRIAP1
1076	ADAR	REPS2
1077	IFT80	ACTR3
1078	LAMA5	TNNT1
1079	WNT5A	SOCS5
1080	HTR2A	PSMA3
1081	GSN	PSMA7
1082	SMAD1	RPL39
1083	RELA	HNRNPA1
1084	GBA	THOC1
1085	S100A9	UBQLN2
1086	NDUFV2	CD34
1087	CXCR4	GALR2
1088	ITGA2B	KIF3C
1089	AGRN	ORC1
1090	CXCR3	AMHR2
1091	PTGS1	ITGB6
1092	CX3CR1	PSPH
1093	RNASEH2A	WIPI1
1094	PDSS2	TMEM97
1095	SPI1	PVRL2
1096	PLUT	SOAT2
1097	SIRT3	ACSL6

1098	IL4R	SEC61G
1099	NDUFS2	RSAD2
1100	CRX	OTUD4
1101	FMR1	INHA
1102	LOC109623489	MTCL1
1103	EXT2	PRMT3
1104	SCNN1A	ITGB4
1105	PRODH	PVR
1106	SLPI	RAP2A
1107	RNASEH2B	STC2
1108	MIR10A	CD164
1109	CD44	ADAM22
1110	PROX1	SEMA3E
1111	PLAUR	GALC
1112	TET2	TRIM37
1113	EXOSC4	SPTLC1
1114	MIR10B	PPP1CB
1115	NFAT5	SSH1
1116	ZFYVE9	SLC7A8
1117	CEP120	NUDCD3
1118	SLC26A3	CXCL2
1119	TP63	BST2
1120	IL21	TCTN3
1121	TMEM138	GSTA1
1122	TOMM40	MKLN1
1123	CHAT	GNRH2
1124	SNCA	STAB1
1125	TRIM28	CCBL2
1126	SYVN1	MROH7
1127	SPATA7	AANAT
1128	DMD	RP11-138P22.1
1129	WDR20	GCHFR
1130	DNAJB11	RNASEH2A
1131	HRAS	TBC1D9B
1132	ACADS	DPYS
1133	JAG1	ATP2A3
1134	AFP	BLOC1S1-RDH5
1135	RP2	SERPINF1
1136	VNN1	FAM91A1
1137	CMA1	CCND1
1138	SDK1	TRIP10
1139	GNRH1	HMGA1
1140	STAT5A	REV3L
1141	NDUFAF2	IFIT1

1142	RNASEH2C	LOC101928061
1143	MIR148B	PKMYT1
1144	MIR34B	MBTPS2
1145	NAGA	NEUROG3
1146	CX3CL1	ICMT
1147	ARG2	XCL1
1148	IGHMBP2	MYL12A
1149	FAM161A	PEX12
1150	PCARE	RPE65
1151	MTR	TMEM123
1152	PRKN	VGLL1
1153	HSPA1L	AK1
1154	NNAT	GK2
1155	CD46	BMPR1A
1156	BAMBI	DOCK10
1157	ACSM3	RGR
1158	LIMK2	ZNF780A
1159	ENTPD1	E2F2
1160	DNASE1	PNLIPRP1
1161	COL1A1	MOS
1162	CFAP47	NDST2
1163	FCN3	GDE1
1164	UROD	GC
1165	SPARC	EPHA5
1166	NLRP5	PTEN
1167	MIR200A	ORC3
1168	LBR	AOC1
1169	CD226	UFL1
1170	NEK8	SERPINA5
1171	SMAD7	MCFD2
1172	SH2B1	SEL1L
1173	PLAU	PARG
1174	SMURF1	DNAJA1
1175	LAP3	CEACAM5
1176	RNLS	ITM2A
1177	F7	SLC9A3R2
1178	GDNF	PAEP
1179	IL23R	MRPS31
1180	PARK7	KLRAP1
1181	PAFAH1B1	SS18L1
1182	AXL	PTPN13
1183	RPS6KB1	FOXF2
1184	CSF1	DUSP1
1185	IFNGR1	NEO1

1186	DZIP1L	LEP
1187	MT-CYB	RAB40A
1188	NTRK2	SLC20A1
1189	ATP6AP2	PCDHGA12
1190	TNFRSF11A	MPZL2
1191	UNC13B	NCAM1
1192	SP3	ICK
1193	KIF12	LGALS2
1194	CDKN1A	SLC10A1
1195	LAMB2	VPS4B
1196	TRPC1	P2RY11
1197	SLIT2	CHSY1
1198	SP6	GAGE1
1199	TBC1D3G	FOXH1
1200	TSC2	ATP12A
1201	NUP133	TRAF3IP1
1202	HTRA1	MIR3658
1203	DDX58	MAPKAPK5-AS1
1204	AGPAT1	URB1
1205	MT-RNR1	CDH11
1206	CDK2	IL7R
1207	GCLC	AKAP9
1208	ARMC5	TLL2
1209	CCL11	CDKN1B
1210	SLC11A2	CD28
1211	LIF	TTC39A
1212	VIM	DHX29
1213	IGFBP4	NAPG
1214	ANPEP	ANKRD40
1215	CYCS	LOC102724200
1216	CDK5	FUT3
1217	CYP2R1	XYLB
1218	EDA2R	ALPL
1219	FOXP1	PPP2R2B
1220	SDC1	GPR171
1221	LPP	DOLK
1222	DRD4	IFI44
1223	HBEGF	BRINP3
1224	MMP12	KLHL23
1225	NDUFS7	VIP
1226	SCG5	POSTN
1227	IFT27	MC1R
1228	CXCR6	RNF114
1229	MIR150	TAL1

1230	FAT1	ERC1
1231	RECK	AQR
1232	MIR141	B4GALT4
1233	SDC4	RASA3
1234	NDUFS1	44756
1235	CASP7	GATA6
1236	ALPP	CBY1
1237	IARS2	MYL3
1238	ADAM10	TERT
1239	CPOX	GEM
1240	SCNN1B	MIR4680
1241	SNAI1	SYNPO
1242	BSG	LLGL1
1243	SULT1A1	NFIL3
1244	HPX	H2AFB1
1245	MCAM	TSN
1246	SOX17	SPRYD7
1247	DLC1	MAP7
1248	MIR342	CDH4
1249	TRAF3IP1	FAM153A
1250	ARMC9	NTSR2
1251	LRRC7	MYF5
1252	MYC	HMGB3
1253	MIR146B	ALDH3B1
1254	MAP3K7	IGHG1
1255	ETS1	LETMD1
1256	OPTN	MAPK10
1257	CCL4	CITED1
1258	PKM	ACSM2A
1259	FGF1	TIPARP
1260	APEX1	PMP2
1261	MIR9-1	NOS3
1262	IL1RAPL2	CSDC2
1263	RP1	MFAP5
1264	TCTN2	ATP2B1
1265	SDC2	CHRND
1266	PROC	ST5
1267	HAVCR2	VPRBP
1268	HCFC1	CDR2
1269	HLA-G	TLR5
1270	CDKN1B	SYN3
1271	SASH1	ADAM28
1272	NES	TG
1273	XRCC1	PLD2

1274	PSMA6	HCK
1275	IL12A	MYH11
1276	GRP	HCN4
1277	EDNRB	IL1RL2
1278	AIF1	LOC101929524
1279	ZMIZ1	LSR
1280	MIOX	OR5I1
1281	HAS2	LOC100509646
1282	MIR182	SPINT2
1283	UPB1	DCHS1
1284	NDUFA1	MCAT
1285	NDUFB3	ERVW-1
1286	RASGRF1	PIN1P1
1287	CDH13	EIF4A2
1288	MPZ	ITIH1
1289	SERPINB2	RAB40B
1290	FCGR3A	FXYD3
1291	YAP1	NCBP2
1292	ADORA1	AFP
1293	SRD5A1	ABCB11
1294	CALD1	FPR2
1295	TFAM	SRRD
1296	SLC26A9	NUPR1
1297	C5	RAPGEF1
1298	NPY1R	MRC1
1299	RET	S1PR4
1300	PSMB9	ELMO1
1301	WDR35	FETUB
1302	TAC1	FOLH1
1303	CD86	C14orf132
1304	GRN	PDE2A
1305	P2RX7	N4BP1
1306	ADD2	ROS1
1307	NR1I2	GPSM3
1308	CGA	PRICKLE3
1309	NDUFS3	HERC3
1310	MSRB3	C2orf27A
1311	SKIL	KCNB2
1312	FOXP2	FRMPD1
1313	VEGFC	SUPT7L
1314	MIR30A	UGT1A1
1315	MIR26A1	AGXT
1316	PTPN11	MDN1
1317	SHROOM3	SLC18A1

1318	XIST	NUTF2
1319	MIR204	BFSP1
1320	RB1	NEFH
1321	MIR130B	HSD3B2
1322	CCN3	HLA-DRB4
1323	TWIST1	ARHGAP5
1324	FHL2	DLG2
1325	SLC34A1	IFRD2
1326	THG1L	
1327	SERPINF2	
1328	RAF1	
1329	S100A4	
1330	LAMB1	
1331	CTBP1	
1332	WDR73	
1333	CPVL	
1334	AGXT	
1335	NTRK1	
1336	BCAR1	
1337	DACT1	
1338	GRB2	
1339	SLC7A7	
1340	S100A12	
1341	SCNN1G	
1342	RYR1	
1343	PROS1	
1344	DOCK8	
1345	B9D2	
1346	ATP6V1A	
1347	CHN2	
1348	WWTR1	
1349	HSP90AA1	
1350	FAM50B	
1351	TBK1	
1352	CD80	
1353	DNMT1	
1354	PMPCA	
1355	SEC63	
1356	TYMP	
1357	ST6GALNAC2	
1358	NFATC3	
1359	PCNA	
1360	ARL13B	
1361	TNFSF12	

1362	TJP1
1363	NPM1
1364	HAS3
1365	USH1C
1366	MIR199B
1367	HDAC4
1368	MIR27B
1369	PSMA5
1370	PSRC1
1371	CYP2J2
1372	MAP3K1
1373	ACTL7A
1374	CLCN5
1375	POC1A
1376	ACSL4
1377	OCRL
1378	AEBP1
1379	PEG10
1380	MMADHC
1381	ATP7B
1382	NLRP7
1383	CNGB3
1384	IFNB1
1385	ZNF423
1386	TBX18
1387	CASC2
1388	NKX2-1
1389	FGFR3
1390	MYH6
1391	IFT74
1392	KMT2C
1393	ELMO2
1394	CPLANE1
1395	NLRP2
1396	ITGA4
1397	GP6
1398	ICOS
1399	AUH
1400	KIRREL1
1401	B9D1
1402	SLC22A6
1403	CLDN19
1404	CISH
1405	MIR200C

1406	RXRA
1407	ADCY3
1408	DDR1
1409	CDC42
1410	GRK2
1411	ITGAL
1412	DDC
1413	CALR
1414	SEMA3A
1415	RGS1
1416	DRD5
1417	DRD3
1418	DNMT3L
1419	PEG3
1420	KHDC3L
1421	OOEP
1422	PARD6G-AS1
1423	SRSF6
1424	MTHFD1L
1425	FLVCR1
1426	MIR16-1
1427	CYLD
1428	DKK1
1429	KLHDC7A
1430	CAMP
1431	EFEMP1
1432	GPR161
1433	NTN1
1434	CPB2
1435	ITGA1
1436	TBXAS1
1437	CEBPA
1438	GDAP1
1439	ZEB1
1440	GFER
1441	CXCL16
1442	GFND1
1443	CADM1
1444	IGHE
1445	SLC4A4
1446	MIR196A1
1447	MIPEP
1448	IL10RA
1449	BECN1

1450	LTF
1451	NDUFA5
1452	DARS2
1453	GJA4
1454	CD69
1455	CCND2
1456	F2RL1
1457	CNTNAP2
1458	MDM2
1459	TNFRSF18
1460	PGM3
1461	SLC22A12
1462	KIAA0586
1463	HLA-DRB4
1464	CMKLR1
1465	PAGR1
1466	HPSE2
1467	IL22
1468	MIR424
1469	LRG1
1470	MIR497
1471	KITLG
1472	HTT
1473	MIR455
1474	IL9
1475	CYP3A5
1476	EGR1
1477	TNFRSF6B
1478	AFF3
1479	TNFSF13
1480	ROCK1
1481	ZNF365
1482	CA2
1483	EFNB2
1484	FCRL3
1485	KIAA0753
1486	HYOU1
1487	TMEM126A
1488	MT-ND4L
1489	ZNF236
1490	CD19
1491	PAX8
1492	IRF5
1493	ANXA1

1494	SLC6A2
1495	PML
1496	MCTP2
1497	ACAD9
1498	NOTCH4
1499	TREM1
1500	YWHAE
1501	NF1
1502	SLC2A10
1503	CXCL5
1504	HOTAIR
1505	MVK
1506	ADCY10
1507	C2CD3
1508	ELAVL1
1509	MB
1510	CISD3
1511	RPS12
1512	ERRFI1
1513	MBP
1514	RASGRP1
1515	BTD
1516	ALAS2
1517	CYP4A11
1518	MTRR
1519	MDK
1520	TNFSF13B
1521	PRKCH
1522	C1QA
1523	LARS2
1524	SFTPD
1525	CCL20
1526	JAK3
1527	COX4I1
1528	DDOST
1529	PGR
1530	MIR142
1531	HDAC6
1532	ADCYAP1
1533	KLK3
1534	SLC35A5
1535	LIPA
1536	COX5A
1537	FAM20A

1538	GJB2
1539	ACVRL1
1540	TNFRSF8
1541	KCNH2
1542	ESRRA
1543	NCAM1
1544	BID
1545	ABCD4
1546	CCR3
1547	IL19
1548	ZEB2
1549	TLR1
1550	AKT3
1551	SPHK1
1552	MIR133B
1553	BCL2L11
1554	RPGRIP1
1555	HSPA8
1556	TGFBR3
1557	PEPD
1558	ISG15
1559	BCS1L
1560	CXCL1
1561	EZR
1562	GSTK1
1563	CASQ1
1564	MCL1
1565	ITGA3
1566	NEDD4L
1567	ST6GAL1
1568	ANGPTL2
1569	MIR152
1570	TGFB3
1571	VEGFB
1572	LOX
1573	FFAR2
1574	INSL3
1575	RGMA
1576	FYN
1577	BIRC5
1578	UBE2D3
1579	KLRK1
1580	F12
1581	SLC5A5

1582	SMAD5
1583	NDUFB4
1584	CEP41
1585	IMMP2L
1586	RD3
1587	ENO2
1588	ATXN10
1589	MMP10
1590	TCTN3
1591	TCTN1
1592	BLOC1S1
1593	IL6ST
1594	ITGA8
1595	COL18A1
1596	ABCB7
1597	FN3K
1598	GCH1
1599	ANXA2
1600	NPNT
1601	ACP5
1602	IDO1
1603	ESRRB
1604	CLDN10
1605	TKTL1
1606	ACTL7B
1607	ANG
1608	ACSL1
1609	WAS
1610	ABCB11
1611	KLF6
1612	SEZ6L
1613	CBY1
1614	ATL1
1615	MIR30B
1616	SLC16A1
1617	SPTBN2
1618	CLDN16
1619	CDH2
1620	PREP
1621	TRIM31
1622	FCGRT
1623	DEFA3
1624	HMCN1
1625	UTS2R

1626	CYGB
1627	DIO3
1628	ATP1A2
1629	DYNC1H1
1630	FMN2
1631	ATF3
1632	CASP12
1633	NR0B1
1634	SERPINA4
1635	ITGB6
1636	MECP2
1637	KIF3A
1638	SIRT4
1639	TNFRSF10B
1640	CXCR1
1641	TNFRSF13B
1642	CFHR2
1643	DYNC2I1
1644	CSNK2B
1645	ESM1
1646	NPPC
1647	OAT
1648	ARAP1-AS2
1649	DUSP1
1650	C1QTNF6
1651	MIR193A
1652	MAPK9
1653	MIR675
1654	NDUFB6
1655	TPRKB
1656	WDR4
1657	CYBB
1658	NCALD
1659	DEFB1
1660	MIR29B1
1661	HES1
1662	MAP1LC3A
1663	MIR26A2
1664	PLEKHH2
1665	ADAMTSL1
1666	SSTR2
1667	IFT52
1668	NCS1
1669	MT-RNR2

1670	SPTAN1
1671	ITPR1
1672	COL7A1
1673	E2F1
1674	NDST2
1675	MIR32
1676	CMIP
1677	NRG3
1678	H3-3B
1679	UBE3C
1680	LTBP1
1681	MIR383
1682	LYVE1
1683	IL5RA
1684	ANKRD55
1685	GCLM
1686	FGFR2
1687	ALK
1688	CFHR1
1689	F10
1690	PIGF
1691	PLA2G4A
1692	APCS
1693	VDAC1
1694	SUGCT
1695	NEK9
1696	HIPK2
1697	KDM4C
1698	SCAF4
1699	MIR9-3
1700	NOSIP
1701	DYNC2I2
1702	SYN2
1703	GLI1
1704	FUS
1705	ANKS1B
1706	MAP3K5
1707	SSTR3
1708	TYRO3
1709	DES
1710	KLF10
1711	THY1
1712	ITGB4
1713	OSGEP

1714	TP53RK
1715	NDUFAF6
1716	CD68
1717	DACH1
1718	COQ5
1719	BCL2L1
1720	ITGAE
1721	SKP2
1722	PIBF1
1723	C20orf27
1724	EDN3
1725	CCNB1
1726	UNC119B
1727	HABP2
1728	DDAH1
1729	ACTB
1730	SLC2A12
1731	PROCR
1732	C4B
1733	SOAT1
1734	ROCK2
1735	CELA1
1736	RAB8A
1737	WDR45
1738	SMARCA4
1739	EVC2
1740	GSS
1741	THBS4
1742	CD24
1743	CYP11A1
1744	ARHGAP24
1745	SOX2-OT
1746	EXO1
1747	IGKC
1748	MSR1
1749	SLC30A7
1750	ADAM17
1751	IL11
1752	COQ9
1753	ILK
1754	MYO1E
1755	MAT1A
1756	STIM1
1757	ASIC5

1758	XIAP
1759	ZNF407
1760	ADSL
1761	PLCG1
1762	MRPL23
1763	PPOX
1764	KLK1
1765	KHK
1766	FLT3
1767	PDSS1
1768	RBMS1
1769	FREM2
1770	CFLAR
1771	ADNP
1772	GRHPR
1773	TRAP1
1774	COL5A1
1775	IRX3
1776	FRAS1
1777	SERPINA7
1778	RPSAP52
1779	RARA
1780	ACTA1
1781	MIR135A1
1782	FOXC1
1783	DGUOK
1784	CABIN1
1785	ZFP36
1786	LGALS1
1787	KDM6A
1788	TCF19
1789	PDE10A
1790	MT2A
1791	SQSTM1
1792	MOK
1793	MASP2
1794	CRHR1
1795	LOC110283621
1796	IL3
1797	RAPGEF5
1798	HOGA1
1799	LAMA2
1800	MX1
1801	VCL

1802	CR1
1803	CCL21
1804	ASS1
1805	CCR7
1806	NLRC4
1807	SCD5
1808	ABCC2
1809	SLC13A3
1810	DEFA1
1811	IPO8
1812	APLNR
1813	RNF5
1814	DCC
1815	RNF10
1816	MIR9-2
1817	CCN1
1818	MIR590
1819	HULC
1820	GSTA1
1821	TRMU
1822	VASH1
1823	HMBS
1824	SERPINB13
1825	CNKSR3
1826	FMOD
1827	NOX5
1828	BTK
1829	MIR100
1830	UCHL1
1831	ARAP1-AS1
1832	XRCC2
1833	STING1
1834	RENBP
1835	NCOA1
1836	TNC
1837	CERT1
1838	TSBP1
1839	ATP1A1
1840	RAG1
1841	MIR138-1
1842	CRB2
1843	INSRR
1844	KLF15
1845	TAGLN

1846	GALNS
1847	MIR149
1848	APAF1
1849	P2RX4
1850	RXRG
1851	ALCAM
1852	SHLD2
1853	AMPD1
1854	PTAFR
1855	TNFRSF10D
1856	EEF1A1
1857	FOXP4
1858	HSP90B1
1859	IFNA2
1860	IGFBP5
1861	LAMA1
1862	C1GALT1C1
1863	TLR7
1864	YY1
1865	TUBA1A
1866	DRD1
1867	GRAMD2B
1868	CGAS
1869	KCNMA1
1870	MIR30C1
1871	TYMS
1872	LECT2
1873	SRGAP2
1874	MIRLET7C
1875	LRP8
1876	MKI67
1877	GPX4
1878	SORT1
1879	HNRNPF
1880	CDK5RAP1
1881	GUSB
1882	NEFL
1883	TRIM13
1884	CHUK
1885	IFNL3
1886	ITGAX
1887	NFATC1
1888	IRAK4
1889	AHSP

1890	CNTLN
1891	NEK1
1892	TULP3
1893	NSD2
1894	MIRLET7B
1895	ERF
1896	SLC2A5
1897	GUCY2D
1898	MYO7A
1899	BLZF1
1900	CREBBP
1901	KCNH7
1902	SMAD6
1903	NFKB2
1904	KIF17
1905	MAP2K2
1906	MIR770
1907	PLEK
1908	ICAM3
1909	BIRC3
1910	SERPINH1
1911	THPO
1912	EPAS1
1913	MYOM2
1914	MIR335
1915	LCP1
1916	APOD
1917	SCAF8
1918	WNK1
1919	NORAD
1920	PCAT1
1921	TUBB1
1922	ATIC
1923	H19-ICR
1924	BHLHE40
1925	RECQL5
1926	IFT122
1927	NCOA3
1928	SMPD1
1929	LAMC1
1930	AREG
1931	SLC26A1
1932	GP2
1933	SFTA3

1934	MIR646HG
1935	PIGN
1936	VPS4B
1937	MBD2
1938	CDH20
1939	ALPK2
1940	NRP2
1941	NPR2
1942	MIR485
1943	MMP16
1944	FOSL1
1945	MIR215
1946	MIR320A
1947	UBE2D2
1948	FECH
1949	FBF1
1950	TRAF1
1951	PTPN13
1952	ORAI1
1953	RARB
1954	PAX3
1955	APELA
1956	TBXT
1957	EGFL8
1958	SUOX
1959	PDGFC
1960	TEX14
1961	ULK1
1962	AIPL1
1963	COQ6
1964	PRICKLE1
1965	GPIHBP1
1966	PRKD1
1967	C1QTNF5
1968	MIR1207
1969	CR2
1970	TSPAN2
1971	LOC110673971
1972	TFB1M
1973	XRCC3
1974	SOX9
1975	ERCC1
1976	CD209
1977	CD74

1978	CHD9
1979	LSM2
1980	BMPR2
1981	MCM10
1982	TRIM68
1983	ZNF7
1984	COMMD5
1985	OR52I1
1986	ZFP36L1
1987	LINC00667
1988	LINC00342
1989	PKD1L1
1990	LOC106099062
1991	LOC107133510
1992	F9
1993	TRIM25
1994	POLR2I
1995	ALAS1
1996	CTSG
1997	SLC15A2
1998	MIR137
1999	NAIP
2000	OGN
2001	COQ8B
2002	CCBE1
2003	ACVR1
2004	IRF8
2005	SOX6
2006	MIR214
2007	TMSB4X
2008	LOC110006319
2009	MIR199A2
2010	MTMR3
2011	F2R
2012	MUC16
2013	LCA5
2014	SAFB
2015	INPP5D
2016	MIRLET7A3
2017	VHL
2018	PIK3R2
2019	LY75
2020	NOD1
2021	FLNA

2022	GTPBP3
2023	MTHFS
2024	CEP164
2025	STC1
2026	MIRLET7A2
2027	SPON2
2028	IFI27
2029	PKD2L1
2030	CSTB
2031	SPPL3
2032	CARD8
2033	CDK1
2034	A2M
2035	CXCR2
2036	XCL1
2037	STX8
2038	PCSK1N
2039	PODN
2040	HAX1
2041	STAT6
2042	GPRC5B
2043	OTC
2044	ZNRF3
2045	CCND3
2046	TTC39C
2047	SLC12A5
2048	ERCC6
2049	RPTOR
2050	ITGA6
2051	MR1
2052	BTRC
2053	EHMT2
2054	ZRANB3
2055	KMT5A
2056	HNRNPH2
2057	ZAP70
2058	CPT1C
2059	IL1RL1
2060	MT-TR
2061	ESRRG
2062	LAMP1
2063	TNFSF4
2064	NT5E
2065	CETN3

2066	CD58
2067	TRA
2068	IL36A
2069	SALL1
2070	WNT9B
2071	XK
2072	PTER
2073	STK19
2074	ITIH4
2075	FIG4
2076	MFF
2077	KCNK1
2078	POU3F4
2079	IQGAP1
2080	S100A1
2081	ENO1
2082	CNSN
2083	MFN1
2084	PREX1
2085	ACAA1
2086	PTK2
2087	LGALSL
2088	NPR3
2089	AGTRAP
2090	SLC3A1
2091	TLR10
2092	CCL28
2093	SLC26A8
2094	VPS11
2095	F2RL3
2096	LUM
2097	MTNR1A
2098	MIR196B
2099	DNASE1L3
2100	IFIT1
2101	MYL2
2102	UHRF1
2103	RREB1
2104	UBC
2105	CORO2B
2106	C1QB
2107	ECE1
2108	MTERF1
2109	MST1R

2110	EYA1
2111	GATM
2112	USP9X
2113	PBX3
2114	NAV3
2115	PARL
2116	CCNL1
2117	KIF1A
2118	SAA2
2119	MIR499A
2120	AURKA
2121	SLC3A2
2122	COL3A1
2123	FAH
2124	C3AR1
2125	RAMP2
2126	DNMT3B
2127	SUV39H1
2128	TMPO
2129	PITX2
2130	TMEM70
2131	ALPI
2132	GPANK1
2133	LCN1
2134	ELAC2
2135	SETD7
2136	SLC7A1
2137	NDUFB8
2138	KCNV1
2139	PKD1L3
2140	LY86-AS1
2141	SMARCA1
2142	METTL14
2143	PSD3
2144	RHOD
2145	STAT2
2146	SORCS3
2147	TUBD1
2148	PTK2B
2149	TNFAIP8
2150	MTO1
2151	FLNB
2152	HSPB2
2153	MIR107

2154	LEKR1
2155	GALNT17
2156	ARSB
2157	ETFB
2158	CDH22
2159	CALB1
2160	MAPKBP1
2161	RAP1B
2162	ATG5
2163	CARD9
2164	IGES
2165	MIR17HG
2166	MUC20
2167	MIR103A1
2168	SH3YL1
2169	ARRDC4
2170	SOCS6
2171	MIR494
2172	HSD3B2
2173	RICTOR
2174	EMX2
2175	AASS
2176	CCL25
2177	CALCR
2178	SPG21
2179	SHPK
2180	SLC15A1
2181	GPHA2
2182	PRMT1
2183	RBFOX1
2184	CYP2A6
2185	AIM2
2186	GIPC1
2187	PNKP
2188	STAC
2189	SLC6A6
2190	HLTF
2191	MIR378A
2192	HECW1
2193	PFN2
2194	CYP1B1
2195	RGN
2196	MIR194-1
2197	NOX3

2198	TFF1
2199	NLRP12
2200	PRPSAP1
2201	SLC4A1
2202	CEP83
2203	ERVFRD-1
2204	PKD1P1
2205	ST2
2206	MRE11
2207	PKD1L2
2208	PSAP
2209	EPRS1
2210	RAB38
2211	MEP1B
2212	PLSCR3
2213	FBXW7
2214	TXNRD1
2215	CASC15
2216	RARS1
2217	REL
2218	MAGI2
2219	CYP11B1
2220	ASL
2221	NCOR1
2222	VPS39
2223	PTPN6
2224	ACTN1
2225	TRPM5
2226	RYR3
2227	MIR186
2228	PDPK1
2229	DXO
2230	SNORA33
2231	E2F3
2232	TSHZ2
2233	DGKQ
2234	IHH
2235	PLVAP
2236	HORMAD2
2237	TNFSF12-TNFSF13
2238	C5orf64
2239	SMIM23
2240	PDE6C
2241	EIF2A

2242	TACR1
2243	OIP5-AS1
2244	GRIA1
2245	FCER2
2246	VEGFD
2247	STAB1
2248	PRPS1L1
2249	RLN2
2250	LCOR
2251	THBS2
2252	HDAC3
2253	RAB7B
2254	IGSF21
2255	VEPH1
2256	HSD17B1
2257	MIR218-1
2258	ACKR1
2259	MIR106A
2260	MIR422A
2261	MIR183
2262	BMPR1A
2263	FBLN1
2264	STEAP1B
2265	ABTB3
2266	SNHG16
2267	ANKRD26
2268	CLDN1
2269	PYCARD
2270	FCAMR
2271	ACTC1
2272	H3C14
2273	CAPN6
2274	TCF21
2275	MPC2
2276	WNK4
2277	ITGAV
2278	CDK7
2279	COX10
2280	MIR520H
2281	UBE4A
2282	KCNE1
2283	IFT81
2284	TRIM56
2285	UNC5B

2286	CCL26
2287	MIR135A2
2288	SRY
2289	MYH7B
2290	PFKP
2291	NDST1
2292	VAV3
2293	PICK1
2294	CHEK1
2295	FUCA1
2296	CEP135
2297	NPR1
2298	CD81
2299	MRC2
2300	MIR4490
2301	RNASEH1
2302	ADH1A
2303	METTL3
2304	RECQL4
2305	MMUT
2306	TOR2A
2307	NIPSNAP2
2308	CSF2RA
2309	AXDND1
2310	SUV39H2
2311	NDFIP2
2312	CYP2B6
2313	AOX1
2314	SOSTDC1
2315	IFT20
2316	CEP97
2317	ASCL2
2318	SKP1
2319	WDR62
2320	IPPK
2321	PXN
2322	KRT18P34
2323	ADAMTS9-AS2
2324	SOX11
2325	RNF185
2326	COA3
2327	CLEC4A
2328	LAMA4
2329	MPC1

2330	HAS1
2331	MIR1228
2332	SPINT2
2333	MIR802
2334	FCER1A
2335	HLA-DPB2
2336	LAMP3
2337	IMMP1L
2338	PHEX
2339	TFF3
2340	TSPYL2
2341	CHRNA1
2342	LTC4S
2343	PTH1R
2344	PARG
2345	SMARCB1
2346	RTN1
2347	NCOA2
2348	S1PR1
2349	STK39
2350	ATP5F1B
2351	TCF3
2352	SFRP1
2353	CD47
2354	SIX5
2355	TNFRSF12A
2356	SLCO1A2
2357	TNR
2358	CALB2
2359	GLI3
2360	CTCF
2361	KLRA1P
2362	ADORA2B
2363	SESN2
2364	ABCA12
2365	CROCC
2366	MCPH1
2367	PTGR3
2368	RAD52
2369	RAMP3
2370	MMAB
2371	LINC01619
2372	DUOX2
2373	UCA1

2374	GSDMD
2375	STIM2
2376	RNF166
2377	FKBP3
2378	UBE2V1
2379	ACTA2
2380	CHD7
2381	PPME1
2382	GPR158
2383	WNT1
2384	GADD45G
2385	MNT
2386	GCM1
2387	DHFR
2388	PAK1
2389	ALS2
2390	KISS1
2391	GPRC5A
2392	WTAP
2393	KIF6
2394	IGBP1
2395	MPRIP
2396	MSN
2397	PRCP
2398	GNG2
2399	SNCAIP
2400	LHX3
2401	NCOR2
2402	GNA11
2403	DCX
2404	IDH1
2405	SCLT1
2406	PPP3CA
2407	SLC9C1
2408	GPR55
2409	SRSF2
2410	FOXN2
2411	SNHG5
2412	MSC-AS1
2413	DAG1
2414	PKD2L2
2415	PKDREJ
2416	ITGA5
2417	TALDO1

2418	MPL
2419	MMP24
2420	VLDLR
2421	JCHAIN
2422	HDAC5
2423	HRG
2424	TGFBRAP1
2425	DDN
2426	MIR488
2427	PRNP
2428	MYT1L
2429	CREB5
2430	CCDC68
2431	PTPN14
2432	SPOP
2433	ACKR2
2434	SLC25A24
2435	DCAF5
2436	RPS15AP30
2437	HSALNG0080722
2438	TENM2
2439	DLX6-AS1
2440	AK3
2441	SLIT3
2442	SLC5A6
2443	SCNN1D
2444	SLC1A7
2445	MFAP3
2446	HORMAD2-AS1
2447	TLR8
2448	LAMP2
2449	PPT2-EGFL8
2450	DMRT1
2451	CLEC12A
2452	PFKFB2
2453	CCNG2
2454	LRP2BP
2455	CEP89
2456	PTGIR
2457	CARM1
2458	ADAMTS5
2459	ZIC3
2460	FBXL4
2461	DSTYK

2462	BGN
2463	PTPRU
2464	AGO2
2465	TET3
2466	SFRP2
2467	FDX1
2468	MTMR2
2469	SBF2
2470	GLRA3
2471	PAX8-AS1
2472	YARS2
2473	FIS1
2474	OPLAH
2475	SMARCA2
2476	SLC9A3R2
2477	PSORS1C1
2478	ATP8B1
2479	COQ8A
2480	LIN7C
2481	C1QBP
2482	RAB11A
2483	DERL2
2484	TNFRSF10C
2485	CAP1
2486	SETD2
2487	EWSR1
2488	SHANK1
2489	CFHR3
2490	PTPRQ
2491	MYH2
2492	NUMB
2493	MAD2L2
2494	DUSP26
2495	IFT43
2496	INPP5B
2497	ZBTB16
2498	SEMA3G
2499	NCF2
2500	GAL3ST1
2501	ARL13A
2502	ACOXL
2503	KIAA0319L
2504	MUS81
2505	BAZ1A

2506	TIPIN
2507	TMEM107
2508	THRA
2509	MED25
2510	SLC25A11
2511	MIR29B2
2512	GRAP
2513	PLD1
2514	PLXNA1
2515	FSTL5
2516	EXD2
2517	TOMM22P3
2518	HSALNG0111532
2519	LOC124903691
2520	piR-39858-354
2521	CCL8
2522	PGA3
2523	OXCT1
2524	GBE1
2525	TFEB
2526	CD79B
2527	TEC
2528	FOXE3
2529	RAB3IP
2530	RND3
2531	ATF2
2532	TNS1
2533	CTTN
2534	MT-TG
2535	CTSC
2536	LGALS3BP
2537	ARF6
2538	PGC
2539	OIT3
2540	CDKN2C
2541	CEP162
2542	CXCL6
2543	PDE6D
2544	GPC3
2545	KERA
2546	RHOB
2547	EXOC7
2548	RBL1
2549	CABYR

2550	PRNCR1
2551	RPL37A
2552	VSTM2B
2553	NLRC5
2554	CYP20A1
2555	GABRR1
2556	GABRR2
2557	LILRB3
2558	ACTG2
2559	ARSA
2560	UNC119
2561	CTCFL
2562	ALPK1
2563	CRKL
2564	BRD4
2565	MRPS15
2566	OSCP1
2567	MAP3K21
2568	LINC00917
2569	LINC02774
2570	LOC100506023
2571	LOC101928236
2572	LOC100131080
2573	LOC729200
2574	BBOX1
2575	MIR19B1
2576	COX15
2577	MRRF
2578	SEPTIN9
2579	RAB7A
2580	ASGR1
2581	PARP12
2582	ARHGAP45
2583	C6orf15
2584	MUCL3
2585	LHB
2586	CXCR5
2587	CFL1
2588	NTN4
2589	WT1-AS
2590	POC5
2591	INPP5K
2592	RNASE3
2593	CES2

2594	SLC26A6
2595	MT-TM
2596	SIX2
2597	SMPD3
2598	SLC7A6
2599	TXN2
2600	ODC1
2601	COL5A2
2602	DNM3
2603	ACAD11
2604	BIRC2
2605	SLC35D3
2606	ZYX
2607	FIBP
2608	LYN
2609	MYH10
2610	NAP1L4
2611	ABCF1
2612	KMT2D
2613	POLI
2614	PDIA3P1
2615	SOCS5
2616	CALCRL
2617	BACH1
2618	IDH2
2619	RIPK1
2620	CBL
2621	IFT46
2622	PDLIM5
2623	SMPD2
2624	MIR103A2
2625	MORC2
2626	REPS1
2627	CGNL1
2628	PIK3IP1
2629	UST
2630	RAD51B
2631	TAF8
2632	TBC1D5
2633	MED20
2634	METTL23
2635	SFI1
2636	TPRG1
2637	SUSD6

2638	CFAP44
2639	TPRG1-AS1
2640	ENSG00000267546
2641	RNU6-921P
2642	ENSG00000253369
2643	ENSG00000256569
2644	ENSG00000124593
2645	ENSG00000272386
2646	GSTM5P1
2647	RN7SL865P
2648	MN297907-007
2649	HSALNG0031478
2650	lnc-LPP-2
2651	WHAMM
2652	WNT11
2653	C1D
2654	DVL1
2655	TSLP
2656	P3H4
2657	MT-TC
2658	UBE3A
2659	TRDN
2660	TRPA1
2661	VAV1
2662	CSRP3
2663	EVC
2664	NUP62
2665	TRIAP1
2666	PRDM15
2667	MAP3K14
2668	GALC
2669	ERVW-1
2670	STARD5
2671	COX4I2
2672	OSGEPL1
2673	YRDC
2674	COL6A1
2675	PPAT
2676	ALDH16A1
2677	WEE1
2678	TUBA8
2679	TXK
2680	SLC7A8
2681	SMG6

2682	POU2AF1
2683	MIR374A
2684	MMAA
2685	MYOD1
2686	CRBN
2687	HNRNPC
2688	TRAF5
2689	PRICKLE2
2690	DGCR5
2691	RAB27A
2692	GCA
2693	LTA4H
2694	TNFRSF19
2695	ZNF175
2696	PABPC4L
2697	SCN1A-AS1
2698	PDPN
2699	HCP5
2700	INCENP
2701	ELK1
2702	ASGR2
2703	IL36G
2704	PRDM9
2705	DZANK1
2706	CCAT1
2707	CETN2
2708	RNU4ATAC
2709	UPK3A
2710	TUBG1
2711	CDCA7
2712	DUP2Q31.1
2713	RCAN1
2714	MITF
2715	UQCRC2
2716	COX6B1
2717	COX7C
2718	NNT-AS1
2719	NEU2
2720	SYT1
2721	SAP30BP
2722	MUC7
2723	GUCY1A1
2724	TRIM47
2725	TRIM65

2726	C19orf81
2727	ADM5
2728	ANXA7
2729	MYCT1
2730	MIR133A1
2731	TUBB4B
2732	VTCN1
2733	SH2D4A
2734	H2AX
2735	GP9
2736	IFT57
2737	DISC1
2738	UBA52
2739	RARS2
2740	MRTFA
2741	ORAI3
2742	TRIT1
2743	CEP63
2744	PRDX6
2745	LALBA
2746	USP14
2747	SMN1
2748	M6PR
2749	TBX3
2750	NBEAL2
2751	AAGAB
2752	BLOC1S3
2753	PGA5
2754	PGA4
2755	CREM
2756	DCDC2
2757	KPNA1
2758	MIR500A
2759	TMEM147
2760	MEOX2
2761	DYSF
2762	LATS2
2763	CCL23
2764	STK25
2765	TNFSF14
2766	SLC66A1
2767	HPS5
2768	ANXA11
2769	UNC13D

2770	MATN3
2771	CNPY3
2772	COLEC11
2773	DIAPH3
2774	PUM2
2775	REV1
2776	SUCLG2
2777	WWC1
2778	CDK3
2779	FUT9
2780	KDM4D
2781	LSAMP
2782	NCR2
2783	BCAS1
2784	CRCP
2785	GATC
2786	MAGOH
2787	PTOV1
2788	SRP68
2789	ZHX2
2790	CCDC80
2791	DCLK3
2792	SYT3
2793	TAPT1
2794	ZNF473
2795	CABP1
2796	EPYC
2797	UBAC1
2798	ALLC
2799	CTU1
2800	DYDC1
2801	MFSD11
2802	POP5
2803	TSGA10
2804	TSPAN14
2805	UNK
2806	DYDC2
2807	TBC1D21
2808	ANKRD29
2809	C19orf48
2810	MITD1
2811	AMTN
2812	SPINK4
2813	ABRACL

2814	KRTAP3-2
2815	KRTAP3-3
2816	NEPRO
2817	PLAC9
2818	CD200R1L
2819	TBC1D31
2820	IGSF22
2821	LYG1
2822	FBLL1
2823	PLEKHD1
2824	PRXL2A
2825	SMIM13
2826	TMEM61
2827	CZIB
2828	EFCAB8
2829	SCGB2B2
2830	TRABD2B
2831	TMEM250
2832	HAND2-AS1
2833	NUTM2B-AS1
2834	CASC19
2835	CASC9
2836	FOXP4-AS1
2837	LINC00857
2838	B4GALT1-AS1
2839	LINC00484
2840	LINC01191
2841	MBL1P
2842	LINC01151
2843	LINC01249
2844	LINC01266
2845	LINC01276
2846	MGC32805
2847	SMILR
2848	PTOV1-AS2
2849	INSYN1-AS1
2850	LINC02042
2851	LINC02607
2852	LOC729296
2853	LRP8-DT
2854	TBC1D27P
2855	LINC02304
2856	LOC100996664
2857	RNU6ATAC2P

2858	EIF5AP4
2859	ENSG00000111780
2860	ENSG00000230537
2861	ENSG00000235192
2862	ENSG00000258623
2863	ENSG00000266980
2864	ENSG00000267543
2865	ENSG00000268047
2866	LINC02114
2867	LINC02511
2868	LINC02720
2869	LOC100130698
2870	LOC124900247
2871	MAP3K4-AS1
2872	RNU6-1128P
2873	RNU6-41P
2874	RPL24P4
2875	RPL31P52
2876	COTL1P1
2877	ENSG00000232762
2878	ENSG00000249697
2879	ENSG00000254303
2880	ENSG00000264924
2881	ENSG00000267078
2882	ENSG00000267342
2883	ENSG00000267801
2884	LINC01738
2885	LINC02463
2886	PCNPP3
2887	SLC7A15P
2888	ENSG00000265204
2889	ENSG00000273306
2890	HMGN1P17
2891	RNA5SP86
2892	RNU6-1200P
2893	RPL26P31
2894	RPS4XP8
2895	SMC3P1
2896	ENSG00000273381
2897	HMGB1P13
2898	HMGB1P50
2899	LOC105370224
2900	RN7SL72P
2901	RNU7-88P

2902	ARL6IP1P3
2903	ENSG00000234382
2904	ENSG00000283504
2905	ENSG00000288623
2906	HSALNG0041935
2907	HSALNG0094521
2908	HSALNG0134953
2909	NONHSAG023555.2
2910	NPM1P48
2911	RF00017-3505
2912	RF00017-3510
2913	RF00017-4696
2914	RF00017-4882
2915	RF00017-6356
2916	RF00066-124
2917	RN7SKP82
2918	lnc-ABRACL-1
2919	lnc-DERL1-5
2920	lnc-FOXP4-2
2921	lnc-PRCD-5
2922	lnc-PUM2-4
2923	lnc-SHANK1-2
2924	lnc-ST6GALNAC2-27
2925	ENSG00000225416
2926	FJ601684-412
2927	HSALNG0017279
2928	HSALNG0025132
2929	HSALNG0025134
2930	HSALNG0028060
2931	HSALNG0041937
2932	HSALNG0041938
2933	HSALNG0062432
2934	HSALNG0062433
2935	HSALNG0068150
2936	HSALNG0072709
2937	HSALNG0075224
2938	HSALNG0075225
2939	HSALNG0092595
2940	HSALNG0092596
2941	HSALNG0094527
2942	HSALNG0097461-002
2943	HSALNG0102100
2944	LOC124903333
2945	LOC124906344

2946	LOC124909415
2947	MN298114-039
2948	RF00017-3019
2949	RF00066-054
2950	RF00951-072
2951	RF00994-964
2952	RF01045-145
2953	RNU4-37P
2954	hsa-miR-5095-098
2955	lnc-GAP43-16
2956	lnc-SHANK1-3
2957	piR-38959-011
2958	piR-39701-037
2959	piR-57461-057
2960	tRX-Lys-NNN-6-1
2961	AB458446
2962	HSALNG0003684
2963	HSALNG0050138
2964	HSALNG0061757
2965	HSALNG0097472
2966	LOC105378977
2967	LOC107983974
2968	LOC107984625
2969	LOC107986400
2970	LOC124901129
2971	RF00017-6284
2972	piR-30175-020
2973	piR-39476-002
2974	piR-56229-002
2975	ENSG00000279359
2976	LOC105378728
2977	piR-30060-002
2978	piR-32532-002
2979	piR-34000-002
2980	piR-34665-002
2981	piR-41669
2982	piR-42730
2983	piR-47386-002
2984	piR-50948-002
2985	piR-51705-002
2986	piR-53819-002
2987	piR-57133-322
2988	piR-61185-002
2989	LRRC8A

2990	LGALS9
2991	MIR218-2
2992	KAT5
2993	CASP4
2994	MYO15A
2995	CHPT1
2996	USF2
2997	ZCCHC14
2998	C16orf95
2999	SHB
3000	NAP1L5
3001	FAM135A
3002	SAGE1
3003	S100A6
3004	SLC6A19
3005	ANKFY1
3006	PTGER2
3007	TNFAIP1
3008	CCL24
3009	UBR3
3010	CFAP36
3011	PRSS8
3012	MIRLET7E
3013	WARS2
3014	TSFM
3015	COX5B
3016	MRPL44
3017	ADI1
3018	COX6C
3019	FASTKD2
3020	PITPNM1
3021	MRPL18
3022	GPR22
3023	OR4L1
3024	TRMT61B
3025	MRM2
3026	MT-TD
3027	TRL-AAG2-3
3028	CCNE1
3029	SLC44A3
3030	GALNT18
3031	BARX2
3032	TMEM45B
3033	RBM43

3034	RPS27P20
3035	HLA-S
3036	APOL3
3037	PDCD4
3038	MTM1
3039	FCGR1A
3040	OTOF
3041	HSD17B14
3042	SH3TC2
3043	SLC22A18
3044	THYN1
3045	TPSAB1
3046	TCP1
3047	DNAAF9
3048	MEX3C
3049	SMPDL3B
3050	LPAR1
3051	GLB1
3052	SV2B
3053	CRYM
3054	CUL5
3055	RNR1
3056	ARRB1
3057	ACTR2
3058	CD2
3059	WNT4
3060	LTBR
3061	FAM186B
3062	MFAP4
3063	JAML
3064	TTC28-AS1
3065	RELN
3066	SLC7A5
3067	IRF4
3068	TRPV6
3069	TMC1
3070	CLUAP1
3071	STRC
3072	ASB7
3073	TM2D3
3074	AP2B1
3075	KLHL3
3076	POMGNT2
3077	ARF3

3078	SLC7A2
3079	GPER1
3080	GTPBP4
3081	SOX18
3082	C5AR1
3083	SIRPA
3084	C1QC
3085	FAM151A
3086	PPP2CA
3087	LIN28A
3088	MIR544A
3089	PIWIL1
3090	CRISP2
3091	CRLS1
3092	MPP7
3093	AK1
3094	MIR490
3095	FSHB
3096	CCNG1
3097	MORN1
3098	CEP250
3099	P2RX5
3100	TAX1BP3
3101	TAT
3102	ADK
3103	TIA1
3104	PDLIM1
3105	GREM2
3106	TMEM212
3107	ARX
3108	CHRM2
3109	SLC25A21
3110	MATN2
3111	PRDM1
3112	IGHM
3113	INTS11
3114	PCM1
3115	MIR302A
3116	MIR95
3117	VPS45
3118	DNAJC13
3119	ANKS6
3120	UPK1A
3121	FCGR2B

3122	SKI
3123	IGHV4-38-2
3124	TAS2R43
3125	MBL3P
3126	AZU1
3127	MS4A1
3128	PELP1
3129	PHLDA2
3130	TSPAN32
3131	TSSC4
3132	SLC22A18AS
3133	ADAM19
3134	MRPL3
3135	NUP160
3136	NUP85
3137	NME7
3138	CAMKV
3139	ALPK3
3140	SLC16A12
3141	TMEM235
3142	MAX
3143	ARRB2
3144	MRPL12
3145	AGA
3146	PGAM5
3147	NAT8
3148	BBC3
3149	PITRM1
3150	MYH14
3151	SH3KBP1
3152	HOXB7
3153	CDK5RAP2
3154	ANKS3
3155	RBM48
3156	GLIS1
3157	NME3
3158	CXCL11
3159	H2BC12L
3160	ABCA3
3161	BAP1
3162	GRK1
3163	UROS
3164	KIF3B
3165	LRRC56

3166	CTAG1B
3167	TP73-AS1
3168	LGR4
3169	UTP4
3170	ZDBF2
3171	GBA2
3172	RB1CC1
3173	WIPI2
3174	GFRA1
3175	SIX1
3176	EEF1D
3177	LDB1
3178	ING2
3179	TXNDC15
3180	KPNA4
3181	DEFA5
3182	DEFA6
3183	FGFBP1
3184	PAX7
3185	CD27
3186	DNAH11
3187	SLC34A3
3188	CCT5
3189	CXCL13
3190	LINC00472
3191	MIR3137
3192	MIR4270
3193	MRPL19
3194	PTPRJ
3195	STX11
3196	ARFRP1
3197	RNY5
3198	FUT4
3199	HEXD
3200	SPAG5-AS1
3201	UGCG
3202	BMI1
3203	SIK2
3204	COL20A1
3205	SNRNP70
3206	DLGAP4
3207	GJB6
3208	AP1S1
3209	MSTO1

3210	SFTPC
3211	AP2A2
3212	SPRY1
3213	FAM170B
3214	CTSA
3215	EFTUD2
3216	VPS33A
3217	PPM1A
3218	GAK
3219	ANLN
3220	LMBRD1
3221	NEDD4
3222	KIF23
3223	HUWE1
3224	HNRNPA2B1
3225	DIAPH1
3226	ANXA3
3227	MIR744
3228	SCN7A
3229	KDM5C
3230	SLC26A5
3231	HERC1
3232	RCOR1
3233	MAPRE3
3234	ARID4A
3235	MYO5C
3236	DMRT2
3237	GREB1
3238	COMMD9
3239	DMRT3
3240	DCDC1
3241	MYEOV
3242	PRRG2
3243	SEPTIN2
3244	KIFBP
3245	EIPR1
3246	C11orf21
3247	SLC26A10P
3248	WASH3P
3249	CAPN8
3250	BMPR1B
3251	LOXL4
3252	TUBB
3253	CHIA

3254	STMN1
3255	ORAI2
3256	SLC7A9
3257	NRIR
3258	SNTB1
3259	DGCR2
3260	SYNJ1
3261	CENPJ
3262	DNAI1
3263	DNAH5
3264	HPS6
3265	BLOC1S6
3266	CEP152
3267	LYST
3268	VPS16
3269	VPS8
3270	WDR81
3271	VPS18
3272	BLOC1S4
3273	BHLHA9
3274	CMKLR2
3275	CA3
3276	MIR98
3277	MIR510
3278	RIPK2
3279	TNFRSF17
3280	SH2D4B
3281	APOL4
3282	AICDA
3283	CDH11
3284	CD84
3285	DKK3
3286	GOLGA2
3287	H1-5
3288	ZNF543
3289	DEFA1A3
3290	H3C1
3291	SSPOP
3292	C4BPB
3293	UPK1B
3294	CRABP2
3295	TUBB2A
3296	FOSB
3297	HMGB2

3298	CD5L
3299	PHLDB1
3300	MIR18B
3301	SH2D1A
3302	TGM1
3303	GNAI3
3304	A4GALT
3305	TRNT1
3306	MIR193B
3307	PIP5K1C
3308	ZBED5
3309	GALNT10
3310	ATP6V0A4
3311	MIR31HG
3312	IGKV3-20
3313	MIR630
3314	INPP4A
3315	MYO1C
3316	ADGRG1
3317	BOD1
3318	ABCB6
3319	SNRPA
3320	HSPB11
3321	CYS1
3322	FAR2
3323	DPEP1
3324	BAIAP2
3325	H2AC16
3326	H2AC17
3327	JAG2
3328	LEFTY2
3329	ARL2
3330	PACS1
3331	RCC1
3332	DECR1
3333	KIFAP3
3334	CNGA2
3335	TBCK
3336	SACM1L
3337	POC1B
3338	ZNF189
3339	TECTB
3340	LAMA5-AS1
3341	CLCN2

3342	VAV2
3343	EPHA2
3344	RARRES1
3345	SEPTIN7
3346	DCAF1
3347	FCN1
3348	MTMR7
3349	CEP128
3350	SMURF2
3351	RAB34
3352	CCT2
3353	DERL1
3354	PAK2
3355	VCAN
3356	EEF1G
3357	SPAG5
3358	MIR769
3359	MIR1297
3360	ADCK2
3361	UTRN
3362	MIR4767
3363	CASP10
3364	RNPC3
3365	CLCN4
3366	SLC6A8
3367	TRARG1
3368	TNS2
3369	HNRNPL
3370	PAQR3
3371	YBX1
3372	SCAI
3373	GRIN1
3374	SPTB
3375	PAX5
3376	HMMR
3377	H1-4
3378	TFPT
3379	RIPK3
3380	IGLL1
3381	PAFAH1B3
3382	SPA17
3383	MIR135B
3384	GZMA
3385	SLC25A10

3386	SDCBP
3387	SNTB2
3388	CD33
3389	SPTBN1
3390	SGCE
3391	CDK20
3392	UBE2S
3393	WWP1
3394	CKAP5
3395	CEP170
3396	OSBPL5
3397	TRIP10
3398	PMF1
3399	CNTRL
3400	EOGT
3401	ADAMTSL5
3402	SNRPD3
3403	WASF5P
3404	KDM3B
3405	SNTA1
3406	LIMS1
3407	AZIN2
3408	ID1
3409	S100A16
3410	CARNS1
3411	CDK9
3412	GPR146
3413	CUX1
3414	WNT9A
3415	ACYP2
3416	TMEM30A
3417	ANKRD1
3418	CCT4
3419	B4GALT1
3420	HIPK3
3421	MEP1A
3422	CCT3
3423	DUSP13
3424	GZF1
3425	GAMT
3426	MARK2
3427	CCT8
3428	SERPINB4
3429	DEFT1P2

3430	KRIT1
3431	SGK2
3432	HIPK1
3433	VAMP1
3434	LRRC55
3435	AMPD3
3436	DAO
3437	KPNA2
3438	PARVA
3439	NLN
3440	SGK3
3441	FERMT2
3442	SMOX
3443	EFHD2
3444	GLIPR2
3445	GPR182
3446	GSDME
3447	MIR4709
