# Supplementary material for: Niaoduqing alleviates podocyte injury in high glucose model via regulating multiple targets and AGE/RAGE pathway: Network pharmacology and experimental validation
Source: Front Pharmacol. 2023 Feb 27;14:1047184. doi: 10.3389/fphar.2023.1047184 (PMC10009170; doi:10.3389/fphar.2023.1047184)
Supplement: Supplementary file 2 [file Table4.pdf]

Table S4 The relationship between predicted targets and active ingredients

Ingredients	Symbol Name	UniproID
MOL000006	PFKFB3	Q16875
MOL000006	MMP2	P08253
MOL000006	ACHE	P22303
MOL000006	ADORA1	P30542
MOL000006	ADORA2A	P29274
MOL000006	ADCY2	Q08462
MOL000006	AKR1A1	P14550
MOL000006	AKR1B10	O60218
MOL000006	AKR1C1	Q04828
MOL000006	AKR1C2	P52895
MOL000006	AKR1C4	P17516
MOL000006	AKR1C3	P42330
MOL000006	AKR1B1	P15121
MOL000006	ALK	Q9UM73
MOL000006	AMY1A	P04745
MOL000006	APP	P05067
MOL000006	AR	P10275
MOL000006	ALOX12	P18054
MOL000006	ALOX15	P16050
MOL000006	ALOX5	P09917
MOL000006	ARG1	P05089
MOL000006	AHR	P35869
MOL000006	ABCG2	Q9UNQ0
MOL000006	XIAP	P98170
MOL000006	BIRC5	O15392
MOL000006	BCL2L1	Q07817
MOL000006	BACE1	P56817
MOL000006	CAMK2B	Q13554
MOL000006	CA1	P00915
MOL000006	CA2	P00918
MOL000006	CA3	P07451
MOL000006	CA4	P22748
MOL000006	CA9	Q16790
MOL000006	CA5A	P35218
MOL000006	CA6	P23280
MOL000006	CA7	P43166
MOL000006	CA12	O43570
MOL000006	CA13	Q8N1Q1
MOL000006	CA14	Q9ULX7
MOL000006	CSNK2A1	P68400
MOL000006	CASP3	P42574
MOL000006	CASP7	P55210

MOL000006	CASP9	P55211
MOL000006	CD40LG	P29965
MOL000006	CDK4	P11802
MOL000006	TP53	P04637
MOL000006	CDK1	P06493
MOL000006	CCNB3	Q8WWL7
MOL000006	CDK2	P24941
MOL000006	CDK5R1	Q15078
MOL000006	CDK6	Q00534
MOL000006	CDKN1A	P38936
MOL000006	PTGS2	P35354
MOL000006	CFTR	P13569
MOL000006	CYP19A1	P11511
MOL000006	CYP1B1	Q16678
MOL000006	DAPK1	P53355
MOL000006	DPP4	P27487
MOL000006	TOP1	P11387
MOL000006	TOP2A	P11388
MOL000006	DRD4	P21917
MOL000006	MDM2	Q00987
MOL000006	EGFR	P00533
MOL000006	HSD17B1	P14061
MOL000006	HSD17B2	P37059
MOL000006	ESR1	P03372
MOL000006	ESR2	Q92731
MOL000006	ESRRA	P11474
MOL000006	PTK2	Q05397
MOL000006	GRK6	P43250
MOL000006	CCND1	P24385
MOL000006	CCNB1	P14635
MOL000006	GSTP1	P09211
MOL000006	GSK3B	P49841
MOL000006	GLO1	Q04760
MOL000006	GPR35	Q9HC97
MOL000006	HSP90AB1	P08238
MOL000006	HMOX1	P09601
MOL000006	MET	P08581
MOL000006	MCL1	Q07820
MOL000006	INSR	P06213
MOL000006	IGF1R	P08069
MOL000006	ICAM1	P05362
MOL000006	IFNG	P01579
MOL000006	IL10	P22301
MOL000006	IL2	P60568

MOL000006	IL4	P05112
MOL000006	IL6	P05231
MOL000006	CXCR1	P25024
MOL000006	MMP1	P03956
MOL000006	NUF2	Q9BZD4
MOL000006	PYGL	P06737
MOL000006	CD38	P28907
MOL000006	KDM4E	B2RXH2
MOL000006	MMP12	P39900
MOL000006	MMP13	P45452
MOL000006	MMP3	P08254
MOL000006	MMP9	P14780
MOL000006	MAPK1	P28482
MOL000006	MAOA	P21397
MOL000006	PRKACA	P17612
MOL000006	ABCC1	P33527
MOL000006	MPO	P05164
MOL000006	NOX4	Q9NPH5
MOL000006	NFKBIA	P25963
MOL000006	NUAK1	O60285
MOL000006	NCOA2	Q15596
MOL000006	PPARG	P37231
MOL000006	ABCB1	P08183
MOL000006	PIK3CG	P48736
MOL000006	PLA2G1B	P04054
MOL000006	PIK3R1	P27986
MOL000006	PLG	P00747
MOL000006	PARP1	P09874
MOL000006	PCNA	P12004
MOL000006	PTGES	O14684
MOL000006	PTGS1	P23219
MOL000006	PKN1	Q16512
MOL000006	AKT1	P31749
MOL000006	ERBB2	P04626
MOL000006	PTPRS	Q13332
MOL000006	RB1	P06400
MOL000006	AURKB	Q96GD4
MOL000006	NEK2	P51955
MOL000006	NEK6	Q9HC98
MOL000006	PIM1	P11309
MOL000006	PLK1	P53350
MOL000006	SLC2A4	P14672
MOL000006	TNKS	O95271
MOL000006	TNKS2	Q9H2K2

MOL000006	TERT	O14746
MOL000006	F2	P00734
MOL000006	FOS	P01100
MOL000006	RELA	Q04206
MOL000006	TTR	P02766
MOL000006	PRSS1	P07477
MOL000006	TNF	P01375
MOL000006	TYR	P14679
MOL000006	FLT3	P36888
MOL000006	AXL	P30530
MOL000006	SRC	P12931
MOL000006	SYK	P43405
MOL000006	VEGFA	P15692
MOL000006	KDR	P35968
MOL000006	AVPR2	P30518
MOL000006	XDH	P47989
MOL000006	CDK5	Q00535
MOL000006	CCNB2	O95067
MOL000022	PTGS2	P35354
MOL000028	HSD11B1	P28845
MOL000028	HSD11B2	P80365
MOL000028	ACHE	P22303
MOL000028	SCD	O00767
MOL000028	ADORA3	P0DMS8
MOL000028	AKR1B10	O60218
MOL000028	FAAH	O00519
MOL000028	AR	P10275
MOL000028	ALOX5	P09917
MOL000028	BCHE	P06276
MOL000028	CNR1	P21554
MOL000028	CES2	O00748
MOL000028	CD81	P60033
MOL000028	SERPINA6	P08185
MOL000028	CYP17A1	P05093
MOL000028	CYP19A1	P11511
MOL000028	CYP2C19	P33261
MOL000028	CYP51A1	Q16850
MOL000028	POLB	P06746
MOL000028	CDC25A	P30304
MOL000028	CDC25B	P30305
MOL000028	ESR1	P03372
MOL000028	ESR2	Q92731
MOL000028	FABP4	P15090
MOL000028	FABP5	Q01469

MOL000028	FABP3	P05413
MOL000028	FABP1	P07148
MOL000028	HMGCR	P04035
MOL000028	ACP1	P24666
MOL000028	NR1H3	Q13133
MOL000028	MAPK3	P27361
MOL000028	CHRM2	P08172
MOL000028	NPC1L1	Q9UHC9
MOL000028	NOS2	P35228
MOL000028	SLC6A2	P23975
MOL000028	RORA	P35398
MOL000028	RORC	P51449
MOL000028	NR1I3	Q14994
MOL000028	PPARA	Q07869
MOL000028	PPARD	Q03181
MOL000028	PPARG	P37231
MOL000028	PDE4D	Q08499
MOL000028	PLA2G1B	P04054
MOL000028	PREP	P48147
MOL000028	PTGES	O14684
MOL000028	FNTA	P49354
MOL000028	PRKCH	P24723
MOL000028	PTPN1	P18031
MOL000028	PTPN6	P29350
MOL000028	PTPN11	Q06124
MOL000028	PTPRF	P10586
MOL000028	SLC6A4	P31645
MOL000028	SQLE	Q14534
MOL000028	SREBF2	Q12772
MOL000028	PTPN2	P17706
MOL000028	TERT	O14746
MOL000028	SHBG	P04278
MOL000028	UGT2B7	P16662
MOL000028	FNTB	P49356
MOL000033	HSD11B1	P28845
MOL000033	HSD11B2	P80365
MOL000033	ACHE	P22303
MOL000033	AR	P10275
MOL000033	DHCR7	Q9UBM7
MOL000033	BCHE	P06276
MOL000033	CES2	O00748
MOL000033	SERPINA6	P08185
MOL000033	CYP17A1	P05093
MOL000033	CYP19A1	P11511

MOL000033	CYP2C19	P33261
MOL000033	CYP51A1	Q16850
MOL000033	POLB	P06746
MOL000033	ESR1	P03372
MOL000033	ESR2	Q92731
MOL000033	G6PD	P11413
MOL000033	GLRA1	P23415
MOL000033	HMGCR	P04035
MOL000033	NR1H3	Q13133
MOL000033	NR1H2	P55055
MOL000033	CHRM2	P08172
MOL000033	NPC1L1	Q9UHC9
MOL000033	NOS2	P35228
MOL000033	SLC6A2	P23975
MOL000033	RORA	P35398
MOL000033	RORC	P51449
MOL000033	NR1I3	Q14994
MOL000033	PPARA	Q07869
MOL000033	PPARD	Q03181
MOL000033	PPARG	P37231
MOL000033	PGR	P06401
MOL000033	PREP	P48147
MOL000033	PTGES	O14684
MOL000033	PTGER1	P34995
MOL000033	PTGER2	P43116
MOL000033	PTPN1	P18031
MOL000033	PTPN6	P29350
MOL000033	SLC6A4	P31645
MOL000033	SQLE	Q14534
MOL000033	FDFT1	P37268
MOL000033	SREBF2	Q12772
MOL000033	PTPN2	P17706
MOL000033	SHBG	P04278
MOL000033	TBXAS1	P24557
MOL000033	UGT2B7	P16662
MOL000033	VDR	P11473
MOL000049	ACHE	P22303
MOL000049	ADRA1A	P35348
MOL000049	AR	P10275
MOL000049	ADRB2	P07550
MOL000049	DPP4	P27487
MOL000049	GABRA1	P14867
MOL000049	CHRM1	P11229
MOL000049	CHRM2	P08172

MOL000049	CHRM3	P20309
MOL000049	OPRM1	P35372
MOL000049	CHRNA7	P36544
MOL000049	NOS1	P29475
MOL000049	PTGS2	P35354
MOL000049	RXRA	P19793
MOL000049	SCN5A	Q14524
MOL000049	F2	P00734
MOL000072	GABRA1	P14867
MOL000072	CHRNA7	P36544
MOL000072	NCOA1	Q15788
MOL000072	NCOA2	Q15596
MOL000072	PTGS2	P35354
MOL000096	DPEP1	P16444
MOL000096	CALM1	P62158
MOL000096	ESR1	P03372
MOL000096	FASN	P49327
MOL000096	HSP90AB1	P08238
MOL000096	KLF7	O75840
MOL000096	PRKACA	P17612
MOL000096	NCOA2	Q15596
MOL000096	PPARG	P37231
MOL000096	PTGS1	P23219
MOL000096	PTGS2	P35354
MOL000098	PSMD3	O43242
MOL000098	MMP2	P08253
MOL000098	HSPA5	P11021
MOL000098	ACHE	P22303
MOL000098	ACACA	Q13085
MOL000098	AHSA1	O95433
MOL000098	ADORA1	P30542
MOL000098	ADORA2A	P29274
MOL000098	AKR1A1	P14550
MOL000098	AKR1B10	O60218
MOL000098	AKR1C1	Q04828
MOL000098	AKR1C2	P52895
MOL000098	AKR1C4	P17516
MOL000098	AKR1C3	P42330
MOL000098	AKR1B1	P15121
MOL000098	ALK	Q9UM73
MOL000098	MAOB	P27338
MOL000098	AR	P10275
MOL000098	BAX	Q07812
MOL000098	BCL2	P10415

MOL000098	ALOX12	P18054
MOL000098	ALOX15	P16050
MOL000098	ALOX5	P09917
MOL000098	ARG1	P05089
MOL000098	AHR	P35869
MOL000098	ABCG2	Q9UNQ0
MOL000098	BIRC5	O15392
MOL000098	BCL2L1	Q07817
MOL000098	APP	P05067
MOL000098	ADRB2	P07550
MOL000098	BACE1	P56817
MOL000098	CAMK2B	Q13554
MOL000098	CA1	P00915
MOL000098	CA2	P00918
MOL000098	CA3	P07451
MOL000098	CA4	P22748
MOL000098	CA9	Q16790
MOL000098	CA5A	P35218
MOL000098	CA6	P23280
MOL000098	CA7	P43166
MOL000098	CA12	O43570
MOL000098	CA13	Q8N1Q1
MOL000098	CA14	Q9ULX7
MOL000098	CSNK2A1	P68400
MOL000098	CASP3	P42574
MOL000098	CASP8	Q14790
MOL000098	CASP9	P55211
MOL000098	CTSD	P07339
MOL000098	CAV1	Q03135
MOL000098	CXCL2	P19875
MOL000098	CD40LG	P29965
MOL000098	CDK1	P06493
MOL000098	TP53	P04637
MOL000098	CLDN4	O14493
MOL000098	F7	P08709
MOL000098	F10	P00742
MOL000098	COL1A1	P02452
MOL000098	COL3A1	P02461
MOL000098	CRP	P02741
MOL000098	CXCL10	P02778
MOL000098	CXCL11	O14625
MOL000098	CCNB3	Q8WWL7
MOL000098	CDK2	P24941
MOL000098	CDK5R1	Q15078

MOL000098	CDK6	Q00534
MOL000098	CDKN1A	P38936
MOL000098	CDKN2A	P42771
MOL000098	CYP19A1	P11511
MOL000098	CYP1A2	P05177
MOL000098	CYP1B1	Q16678
MOL000098	CYP3A4	P08684
MOL000098	DCAF5	Q96JK2
MOL000098	DAPK1	P53355
MOL000098	DPP4	P27487
MOL000098	TOP1	P11387
MOL000098	TOP2A	P11388
MOL000098	APEX1	P27695
MOL000098	MPG	P29372
MOL000098	DRD4	P21917
MOL000098	DUOX2	Q9NRD8
MOL000098	EGFR	P00533
MOL000098	SELE	P16581
MOL000098	HSD17B1	P14061
MOL000098	HSD17B2	P37059
MOL000098	ESR2	Q92731
MOL000098	SULT1E1	P49888
MOL000098	ESRRA	P11474
MOL000098	ELK1	P19419
MOL000098	EIF6	P56537
MOL000098	PTK2	Q05397
MOL000098	CCND1	P24385
MOL000098	CCNB1	P14635
MOL000098	GABRA1	P14867
MOL000098	GJA1	P17302
MOL000098	GSTM1	P09488
MOL000098	GSTM2	P28161
MOL000098	GSTP1	P09211
MOL000098	GSK3B	P49841
MOL000098	GLO1	Q04760
MOL000098	GPR35	Q9HC97
MOL000098	HSF1	Q00613
MOL000098	HSP90AB1	P08238
MOL000098	HMOX1	P09601
MOL000098	MET	P08581
MOL000098	HK2	P52789
MOL000098	NKX3-1	Q99801
MOL000098	HAS2	Q92819
MOL000098	HIF1A	Q16665

MOL000098	CHUK	O15111
MOL000098	INSR	P06213
MOL000098	IGF1R	P08069
MOL000098	IGF2	P01344
MOL000098	IGFBP3	P17936
MOL000098	ICAM1	P05362
MOL000098	IFNG	P01579
MOL000098	IRF1	P10914
MOL000098	IL1A	P01583
MOL000098	IL1B	P01584
MOL000098	IL10	P22301
MOL000098	IL2	P60568
MOL000098	IL6	P05231
MOL000098	CXCL8	P10145
MOL000098	CXCR1	P25024
MOL000098	MMP1	P03956
MOL000098	PYGL	P06737
MOL000098	CD38	P28907
MOL000098	KDM4E	B2RXH2
MOL000098	MGAM	O43451
MOL000098	MMP12	P39900
MOL000098	MMP13	P45452
MOL000098	MMP3	P08254
MOL000098	MMP9	P14780
MOL000098	MAPT	P10636
MOL000098	MAPK1	P28482
MOL000098	MAOA	P21397
MOL000098	PRKACA	P17612
MOL000098	ABCC1	P33527
MOL000098	MYC	P01106
MOL000098	MPO	P05164
MOL000098	MYLK	Q15746
MOL000098	NQO1	P15559
MOL000098	NOX4	Q9NPH5
MOL000098	POR	P16435
MOL000098	NCF1	P14598
MOL000098	NFKBIA	P25963
MOL000098	NOS3	P29474
MOL000098	NUAK1	O60285
MOL000098	NFE2L2	Q16236
MOL000098	NCOA2	Q15596
MOL000098	NR1I2	O75469
MOL000098	NR1I3	Q14994
MOL000098	ODC1	P11926

MOL000098	SPP1	P10451
MOL000098	PRXC1A	P00433
MOL000098	PPARG	P37231
MOL000098	PPARA	Q07869
MOL000098	PPARD	Q03181
MOL000098	ABCB1	P08183
MOL000098	PTEN	P60484
MOL000098	PIK3CG	P48736
MOL000098	PGK1	P00558
MOL000098	PLA2G1B	P04054
MOL000098	PIK3R1	P27986
MOL000098	SERPINE1	P05121
MOL000098	PARP1	P09874
MOL000098	KCNH2	Q12809
MOL000098	HERC1	Q15751
MOL000098	PCOLCE	Q15113
MOL000098	EGF	P01133
MOL000098	PTGER3	P43115
MOL000098	PTGS1	P23219
MOL000098	PTGS2	P35354
MOL000098	ACPP	P15309
MOL000098	RUNX1T1	Q06455
MOL000098	PRKCA	P17252
MOL000098	PRKCB	P05771
MOL000098	PKN1	Q16512
MOL000098	FOS	P01100
MOL000098	NPEPPS	P55786
MOL000098	AKT1	P31749
MOL000098	RAF1	P04049
MOL000098	RASSF1	Q9NS23
MOL000098	RASA1	P20936
MOL000098	ERBB2	P04626
MOL000098	ERBB3	P21860
MOL000098	PTPRS	Q13332
MOL000098	RB1	P06400
MOL000098	RXRA	P19793
MOL000098	RUNX2	Q13950
MOL000098	AURKA	O14965
MOL000098	AURKB	Q96GD4
MOL000098	CHEK2	O96017
MOL000098	NEK2	P51955
MOL000098	NEK6	Q9HC98
MOL000098	PIM1	P11309
MOL000098	PLK1	P53350

MOL000098	PON1	P27169
MOL000098	STAT1	P42224
MOL000098	SCN5A	Q14524
MOL000098	SLC2A4	P14672
MOL000098	SLC22A12	Q96S37
MOL000098	SOD1	P00441
MOL000098	TNKS	O95271
MOL000098	TNKS2	Q9H2K2
MOL000098	TERT	O14746
MOL000098	F2	P00734
MOL000098	THBD	P07204
MOL000098	PLAT	P00750
MOL000098	E2F1	Q01094
MOL000098	E2F2	Q14209
MOL000098	RELA	Q04206
MOL000098	TGFB1	P01137
MOL000098	TTR	P02766
MOL000098	PRSS1	P07477
MOL000098	TNF	P01375
MOL000098	DIO1	P49895
MOL000098	TYR	P14679
MOL000098	FLT3	P36888
MOL000098	AXL	P30530
MOL000098	SRC	P12931
MOL000098	SYK	P43405
MOL000098	PLAU	P00749
MOL000098	VCAM1	P19320
MOL000098	VEGFA	P15692
MOL000098	KDR	P35968
MOL000098	AVPR2	P30518
MOL000098	XDH	P47989
MOL000098	CDK5	Q00535
MOL000098	CCNB2	O95067
MOL000211	HSD11B1	P28845
MOL000211	HSD11B2	P80365
MOL000211	SCD	O00767
MOL000211	ADORA3	P0DMS8
MOL000211	AKR1B10	O60218
MOL000211	FAAH	O00519
MOL000211	AR	P10275
MOL000211	NR1H4	Q96RI1
MOL000211	CES2	O00748
MOL000211	CD81	P60033
MOL000211	SERPINA6	P08185

MOL000211	PTGS2	P35354
MOL000211	CYP17A1	P05093
MOL000211	CYP19A1	P11511
MOL000211	CYP51A1	Q16850
MOL000211	POLB	P06746
MOL000211	TOP1	P11387
MOL000211	TOP2A	P11388
MOL000211	SLC6A3	Q01959
MOL000211	CDC25A	P30304
MOL000211	CDC25B	P30305
MOL000211	CDC25C	P30307
MOL000211	HSD17B3	P37058
MOL000211	FABP5	Q01469
MOL000211	FABP2	P12104
MOL000211	FABP3	P05413
MOL000211	FABP1	P07148
MOL000211	FFAR1	O14842
MOL000211	GABRA2	P47869
MOL000211	GABBR1	Q9UBS5
MOL000211	G6PD	P11413
MOL000211	GPBAR1	Q8TDU6
MOL000211	HSF1	Q00613
MOL000211	ACP1	P24666
MOL000211	MAPK3	P27361
MOL000211	NPC1L1	Q9UHC9
MOL000211	RORC	P51449
MOL000211	PPARD	Q03181
MOL000211	PPARG	P37231
MOL000211	PDE4D	Q08499
MOL000211	PLA2G1B	P04054
MOL000211	PGR	P06401
MOL000211	PTGES	O14684
MOL000211	PRKCH	P24723
MOL000211	PTPN1	P18031
MOL000211	PTPN6	P29350
MOL000211	PTPN11	Q06124
MOL000211	PTPRF	P10586
MOL000211	SIGMAR1	Q99720
MOL000211	SAE1	Q9UBE0
MOL000211	PTPN2	P17706
MOL000211	TERT	O14746
MOL000211	SHBG	P04278
MOL000211	UGT2B7	P16662
MOL000211	VDR	P11473

MOL000211	UBA2	Q9UBT2
MOL000211	GABRB2	P47870
MOL000211	GABRG2	P18507
MOL000239	PFKFB3	Q16875
MOL000239	ACHE	P22303
MOL000239	ADORA1	P30542
MOL000239	ADORA2A	P29274
MOL000239	ADORA3	P0DMS8
MOL000239	AKR1B1	P15121
MOL000239	ALK	Q9UM73
MOL000239	AMY1A	P04745
MOL000239	AR	P10275
MOL000239	ALOX12	P18054
MOL000239	ALOX15	P16050
MOL000239	ALOX5	P09917
MOL000239	AHR	P35869
MOL000239	ABCG2	Q9UNQ0
MOL000239	APP	P05067
MOL000239	ST6GAL1	P15907
MOL000239	BACE1	P56817
MOL000239	BCHE	P06276
MOL000239	CALM1	P62158
MOL000239	CAMK2B	Q13554
MOL000239	CA1	P00915
MOL000239	CA2	P00918
MOL000239	CA4	P22748
MOL000239	CA9	Q16790
MOL000239	CA6	P23280
MOL000239	CA7	P43166
MOL000239	CA12	O43570
MOL000239	CSNK2A1	P68400
MOL000239	CDK2	P24941
MOL000239	CDK1	P06493
MOL000239	CCNB3	Q8WWL7
MOL000239	CDK5R1	Q15078
MOL000239	CDK6	Q00534
MOL000239	PTGS2	P35354
MOL000239	CYP19A1	P11511
MOL000239	CYP1A2	P05177
MOL000239	CYP1B1	Q16678
MOL000239	DAPK1	P53355
MOL000239	OPRD1	P41143
MOL000239	DPP4	P27487
MOL000239	TOP2A	P11388

MOL000239	APEX1	P27695
MOL000239	MPG	P29372
MOL000239	DRD4	P21917
MOL000239	HSP90B1	P14625
MOL000239	EGFR	P00533
MOL000239	HSD17B1	P14061
MOL000239	HSD17B2	P37059
MOL000239	ESR1	P03372
MOL000239	ESR2	Q92731
MOL000239	ESRRA	P11474
MOL000239	PTK2	Q05397
MOL000239	GRK6	P43250
MOL000239	GCGR	P47871
MOL000239	GSK3B	P49841
MOL000239	GLO1	Q04760
MOL000239	GPR35	Q9HC97
MOL000239	HSP90AB1	P08238
MOL000239	MET	P08581
MOL000239	MCL1	Q07820
MOL000239	INSR	P06213
MOL000239	IGF1R	P08069
MOL000239	CXCR1	P25024
MOL000239	KDM4E	B2RXH2
MOL000239	MMP2	P08253
MOL000239	MMP9	P14780
MOL000239	MAPT	P10636
MOL000239	MAOA	P21397
MOL000239	OPRM1	P35372
MOL000239	ABCC1	P33527
MOL000239	MYLK	Q15746
MOL000239	NOX4	Q9NPH5
MOL000239	NAE1	Q13564
MOL000239	NOS2	P35228
MOL000239	NUAK1	O60285
MOL000239	NCOA2	Q15596
MOL000239	ODC1	P11926
MOL000239	ABCB1	P08183
MOL000239	PDE5A	O76074
MOL000239	PLA2G2A	P14555
MOL000239	PIK3CG	P48736
MOL000239	PLG	P00747
MOL000239	PTGS1	P23219
MOL000239	PKN1	Q16512
MOL000239	PTPRS	Q13332

MOL000239	AKT1	P31749
MOL000239	AURKB	Q96GD4
MOL000239	CHEK1	O14757
MOL000239	NEK2	P51955
MOL000239	NEK6	Q9HC98
MOL000239	PIM1	P11309
MOL000239	PLK1	P53350
MOL000239	SCN5A	Q14524
MOL000239	SLC22A12	Q96S37
MOL000239	KIT	P10721
MOL000239	TERT	O14746
MOL000239	F2	P00734
MOL000239	PRSS1	P07477
MOL000239	TYR	P14679
MOL000239	LCK	P06239
MOL000239	FLT3	P36888
MOL000239	AXL	P30530
MOL000239	SRC	P12931
MOL000239	SYK	P43405
MOL000239	KDR	P35968
MOL000239	AVPR2	P30518
MOL000239	KCNA3	P22001
MOL000239	XDH	P47989
MOL000239	CDK5	Q00535
MOL000239	CCNB1	P14635
MOL000239	CCNB2	O95067
MOL000273	HSD11B1	P28845
MOL000273	HSD11B2	P80365
MOL000273	ALOX5AP	P20292
MOL000273	AKR1B10	O60218
MOL000273	AR	P10275
MOL000273	ALOX12	P18054
MOL000273	BACE1	P56817
MOL000273	SLC10A1	Q14973
MOL000273	BCHE	P06276
MOL000273	CES2	O00748
MOL000273	CD81	P60033
MOL000273	SERPINA6	P08185
MOL000273	CYSLTR1	Q9Y271
MOL000273	CYP17A1	P05093
MOL000273	CYP19A1	P11511
MOL000273	CYP51A1	Q16850
MOL000273	OPRD1	P41143
MOL000273	POLB	P06746

MOL000273	TOP2A	P11388
MOL000273	CDC25B	P30305
MOL000273	EDNRA	P25101
MOL000273	ESR1	P03372
MOL000273	ESR2	Q92731
MOL000273	FABP1	P07148
MOL000273	PTGDR2	Q9Y5Y4
MOL000273	NR3C1	P04150
MOL000273	G6PD	P11413
MOL000273	HMGCR	P04035
MOL000273	HAO1	Q9UJM8
MOL000273	SLC10A2	Q12908
MOL000273	ITGB1	P05556
MOL000273	ITGAV	P06756
MOL000273	OPRK1	P41145
MOL000273	ACP1	P24666
MOL000273	NR1H3	Q13133
MOL000273	MMP1	P03956
MOL000273	MMP10	P09238
MOL000273	MMP12	P39900
MOL000273	MMP13	P45452
MOL000273	MMP2	P08253
MOL000273	MMP3	P08254
MOL000273	MMP8	P22894
MOL000273	MMP9	P14780
MOL000273	NR3C2	P08235
MOL000273	NPC1L1	Q9UHC9
MOL000273	NOS2	P35228
MOL000273	NCOA2	Q15596
MOL000273	RORC	P51449
MOL000273	MDM2	Q00987
MOL000273	PPARA	Q07869
MOL000273	PPARD	Q03181
MOL000273	PPARG	P37231
MOL000273	PDE4D	Q08499
MOL000273	PLA2G1B	P04054
MOL000273	PGR	P06401
MOL000273	PTGES	O14684
MOL000273	PTGDR	Q13258
MOL000273	PTGER1	P34995
MOL000273	PTGER2	P43116
MOL000273	PTGER3	P43115
MOL000273	PTGER4	P35408
MOL000273	PTGFR	P43088

MOL000273	FNTA	P49354
MOL000273	PRKCH	P24723
MOL000273	PTPN1	P18031
MOL000273	PTPN11	Q06124
MOL000273	RASGRP3	Q8IV61
MOL000273	PTPRF	P10586
MOL000273	SIGMAR1	Q99720
MOL000273	PTPN2	P17706
MOL000273	SHBG	P04278
MOL000273	THRA	P10827
MOL000273	THRB	P10828
MOL000273	TNF	P01375
MOL000273	TRPM8	Q7Z2W7
MOL000273	AGTR1	P30556
MOL000273	JAK1	P23458
MOL000273	JAK2	O60674
MOL000273	JAK3	P52333
MOL000273	VDR	P11473
MOL000273	FNTB	P49356
MOL000273	ITGA4	P13612
MOL000273	ITGB3	P05106
MOL000275	HSD11B1	P28845
MOL000275	HSD11B2	P80365
MOL000275	ACHE	P22303
MOL000275	CES1	P23141
MOL000275	SCD	O00767
MOL000275	ADORA3	P0DMS8
MOL000275	AKR1B10	O60218
MOL000275	FAAH	O00519
MOL000275	AR	P10275
MOL000275	ALOX5	P09917
MOL000275	BACE1	P56817
MOL000275	NR1H4	Q96RI1
MOL000275	BCHE	P06276
MOL000275	CNR1	P21554
MOL000275	CES2	O00748
MOL000275	CTSD	P07339
MOL000275	CD81	P60033
MOL000275	SERPINA6	P08185
MOL000275	PTGS1	P23219
MOL000275	PTGS2	P35354
MOL000275	CYP17A1	P05093
MOL000275	CYP19A1	P11511
MOL000275	CYP2C19	P33261

MOL000275	CYP51A1	Q16850
MOL000275	POLB	P06746
MOL000275	TOP1	P11387
MOL000275	TOP2A	P11388
MOL000275	SLC6A3	Q01959
MOL000275	CDC25A	P30304
MOL000275	CDC25B	P30305
MOL000275	CDC25C	P30307
MOL000275	HSD17B3	P37058
MOL000275	ESR1	P03372
MOL000275	ESR2	Q92731
MOL000275	FABP4	P15090
MOL000275	FABP5	Q01469
MOL000275	FABP3	P05413
MOL000275	FABP1	P07148
MOL000275	FFAR1	O14842
MOL000275	NR3C1	P04150
MOL000275	G6PD	P11413
MOL000275	HMGCR	P04035
MOL000275	IDO1	P14902
MOL000275	LTB4R	Q15722
MOL000275	ACP1	P24666
MOL000275	NR1H3	Q13133
MOL000275	MAPK3	P27361
MOL000275	NR3C2	P08235
MOL000275	CHRM2	P08172
MOL000275	NPC1L1	Q9UHC9
MOL000275	NOS2	P35228
MOL000275	SLC6A2	P23975
MOL000275	RORA	P35398
MOL000275	RORC	P51449
MOL000275	NR1I3	Q14994
MOL000275	PPARA	Q07869
MOL000275	PPARD	Q03181
MOL000275	PPARG	P37231
MOL000275	PDE4D	Q08499
MOL000275	PLA2G1B	P04054
MOL000275	NR1I2	O75469
MOL000275	PGR	P06401
MOL000275	PREP	P48147
MOL000275	PTGES	O14684
MOL000275	PTGER1	P34995
MOL000275	PTGER2	P43116
MOL000275	PTGER3	P43115

MOL000275	PTGER4	P35408
MOL000275	PTGIR	P43119
MOL000275	FNTA	P49354
MOL000275	PRKCH	P24723
MOL000275	PTPN1	P18031
MOL000275	PTPN6	P29350
MOL000275	PTPN11	Q06124
MOL000275	PTPRF	P10586
MOL000275	SLC6A4	P31645
MOL000275	SIGMAR1	Q99720
MOL000275	SLC22A6	Q4U2R8
MOL000275	SRD5A1	P18405
MOL000275	SRD5A2	P31213
MOL000275	SREBF2	Q12772
MOL000275	SAE1	Q9UBE0
MOL000275	PTPN2	P17706
MOL000275	TERT	O14746
MOL000275	SHBG	P04278
MOL000275	TRPV1	Q8NER1
MOL000275	UBA2	Q9UBT2
MOL000275	FNTB	P49356
MOL000279	HSD11B1	P28845
MOL000279	PFKFB3	Q16875
MOL000279	ADORA1	P30542
MOL000279	ADORA2A	P29274
MOL000279	AKR1C3	P42330
MOL000279	ALK	Q9UM73
MOL000279	AMPD3	Q01432
MOL000279	AR	P10275
MOL000279	AURKAIP1	Q9NWT8
MOL000279	CNR2	P34972
MOL000279	CES2	O00748
MOL000279	CCR1	P32246
MOL000279	CDC7	O00311
MOL000279	CDK8	P49336
MOL000279	MAPK8	P45983
MOL000279	F11	P03951
MOL000279	CCNT1	O60563
MOL000279	CDK2	P24941
MOL000279	CCNE2	O96020
MOL000279	CCND1	P24385
MOL000279	CYP17A1	P05093
MOL000279	CYP19A1	P11511
MOL000279	CYP2C19	P33261

MOL000279	CYP51A1	Q16850
MOL000279	OPRD1	P41143
MOL000279	DGAT1	O75907
MOL000279	DRD2	P14416
MOL000279	ESR1	P03372
MOL000279	ESR2	Q92731
MOL000279	PSEN2	P49810
MOL000279	NR3C1	P04150
MOL000279	G6PD	P11413
MOL000279	GRIN2A	Q12879
MOL000279	GRIA2	P42262
MOL000279	GSK3B	P49841
MOL000279	MET	P08581
MOL000279	HMGCR	P04035
MOL000279	CHUK	O15111
MOL000279	IKBKB	O14920
MOL000279	ITGAL	P20701
MOL000279	OPRK1	P41145
MOL000279	LRRK2	Q5S007
MOL000279	PYGL	P06737
MOL000279	NR1H3	Q13133
MOL000279	NR1H2	P55055
MOL000279	CSF1R	P07333
MOL000279	MAPK14	Q16539
MOL000279	MTNR1A	P48039
MOL000279	MTNR1B	P49286
MOL000279	GRM1	Q13255
MOL000279	GRM2	Q14416
MOL000279	GRM5	P41594
MOL000279	NR3C2	P08235
MOL000279	MAP3K14	Q99558
MOL000279	MGLL	Q99685
MOL000279	OPRM1	P35372
MOL000279	CHRM1	P11229
MOL000279	CHRM2	P08172
MOL000279	NTRK1	P04629
MOL000279	TACR2	P21452
MOL000279	NPC1L1	Q9UHC9
MOL000279	OPRL1	P41146
MOL000279	SLC6A2	P23975
MOL000279	RORA	P35398
MOL000279	RORC	P51449
MOL000279	HCRTR1	O43613
MOL000279	HCRTR2	O43614

MOL000279	MDM2	Q00987
MOL000279	PDE2A	O00408
MOL000279	PDE4B	Q07343
MOL000279	PIK3CA	P42336
MOL000279	PDGFRB	P09619
MOL000279	PTGER1	P34995
MOL000279	PTGER3	P43115
MOL000279	PTGFR	P43088
MOL000279	PRKCA	P17252
MOL000279	PRKCB	P05771
MOL000279	PRKCE	Q02156
MOL000279	MDM4	O15151
MOL000279	PTPN1	P18031
MOL000279	ROCK1	Q13464
MOL000279	ROCK2	O75116
MOL000279	AKT1	P31749
MOL000279	AURKA	O14965
MOL000279	AURKB	Q96GD4
MOL000279	MTOR	P42345
MOL000279	HTR6	P50406
MOL000279	SLC6A4	P31645
MOL000279	SMO	Q99835
MOL000279	S1PR1	P21453
MOL000279	S1PR3	Q99500
MOL000279	KIT	P10721
MOL000279	SHBG	P04278
MOL000279	F10	P00742
MOL000279	ABL1	P00519
MOL000279	TRPV1	Q8NER1
MOL000279	KDR	P35968
MOL000279	AVPR1A	P37288
MOL000279	VDR	P11473
MOL000279	KCNA3	P22001
MOL000279	KCNA5	P22460
MOL000279	PSENEN	Q9NZ42
MOL000279	NCSTN	Q92542
MOL000279	APH1A	Q96BI3
MOL000279	PSEN1	P49768
MOL000279	APH1B	Q8WW43
MOL000279	GRIN1	Q05586
MOL000279	ICAM1	P05362
MOL000279	ITGB2	P05107
MOL000279	CDK4	P11802
MOL000279	CCNE1	P24864

MOL000282	ACHE	P22303
MOL000282	AR	P10275
MOL000282	BCHE	P06276
MOL000282	CYP17A1	P05093
MOL000282	CYP19A1	P11511
MOL000282	CYP2C19	P33261
MOL000282	CYP51A1	Q16850
MOL000282	ESR1	P03372
MOL000282	ESR2	Q92731
MOL000282	HMGCR	P04035
MOL000282	NR1H3	Q13133
MOL000282	CHRM2	P08172
MOL000282	NPC1L1	Q9UHC9
MOL000282	SLC6A2	P23975
MOL000282	RORA	P35398
MOL000282	RORC	P51449
MOL000282	NR1I3	Q14994
MOL000282	PGR	P06401
MOL000282	PTPN1	P18031
MOL000282	SLC6A4	P31645
MOL000282	SREBF2	Q12772
MOL000282	SHBG	P04278
MOL000283	HSD11B2	P80365
MOL000283	ADORA1	P30542
MOL000283	ADORA2A	P29274
MOL000283	AKR1C3	P42330
MOL000283	FAAH	O00519
MOL000283	DHCR7	Q9UBM7
MOL000283	BACE1	P56817
MOL000283	CNR1	P21554
MOL000283	CNR2	P34972
MOL000283	CTSD	P07339
MOL000283	MAPK8	P45983
MOL000283	FASN	P49327
MOL000283	GLRA1	P23415
MOL000283	HPGDS	O60760
MOL000283	NR1H2	P55055
MOL000283	NOS2	P35228
MOL000283	MDM2	Q00987
MOL000283	PDE4A	P27815
MOL000283	PDE4B	Q07343
MOL000283	PDE4C	Q08493
MOL000283	PDE4D	Q08499
MOL000283	PGR	P06401

MOL000283	SHH	Q15465
MOL000283	TRPV1	Q8NER1
MOL000287	HSD11B1	P28845
MOL000287	HSD11B2	P80365
MOL000287	ALOX5AP	P20292
MOL000287	ACHE	P22303
MOL000287	CES1	P23141
MOL000287	SCD	O00767
MOL000287	ADORA3	P0DMS8
MOL000287	AKR1B10	O60218
MOL000287	FAAH	O00519
MOL000287	AR	P10275
MOL000287	ALOX5	P09917
MOL000287	BACE1	P56817
MOL000287	NR1H4	Q96RI1
MOL000287	BCHE	P06276
MOL000287	CNR1	P21554
MOL000287	CES2	O00748
MOL000287	CTSD	P07339
MOL000287	CD81	P60033
MOL000287	SERPINA6	P08185
MOL000287	PTGS1	P23219
MOL000287	PTGS2	P35354
MOL000287	CYP17A1	P05093
MOL000287	CYP19A1	P11511
MOL000287	CYP2C19	P33261
MOL000287	CYP51A1	Q16850
MOL000287	POLB	P06746
MOL000287	TOP1	P11387
MOL000287	TOP2A	P11388
MOL000287	SLC6A3	Q01959
MOL000287	CDC25A	P30304
MOL000287	CDC25B	P30305
MOL000287	EDNRA	P25101
MOL000287	HSD17B3	P37058
MOL000287	ESR1	P03372
MOL000287	ESR2	Q92731
MOL000287	FABP4	P15090
MOL000287	FABP5	Q01469
MOL000287	FABP3	P05413
MOL000287	FABP1	P07148
MOL000287	FFAR1	O14842
MOL000287	NR3C1	P04150
MOL000287	G6PD	P11413

MOL000287	HMGCR	P04035
MOL000287	IDO1	P14902
MOL000287	LTB4R	Q15722
MOL000287	ACP1	P24666
MOL000287	NR1H3	Q13133
MOL000287	MAPK3	P27361
MOL000287	NR3C2	P08235
MOL000287	CHRM2	P08172
MOL000287	NPC1L1	Q9UHC9
MOL000287	NOS2	P35228
MOL000287	SLC6A2	P23975
MOL000287	RORA	P35398
MOL000287	RORC	P51449
MOL000287	NR1I3	Q14994
MOL000287	MDM2	Q00987
MOL000287	PPARA	Q07869
MOL000287	PPARD	Q03181
MOL000287	PPARG	P37231
MOL000287	PDE4D	Q08499
MOL000287	PLA2G1B	P04054
MOL000287	NR1I2	O75469
MOL000287	PGR	P06401
MOL000287	PREP	P48147
MOL000287	PTGES	O14684
MOL000287	PTGER1	P34995
MOL000287	PTGER2	P43116
MOL000287	PTGER3	P43115
MOL000287	PTGER4	P35408
MOL000287	PTGFR	P43088
MOL000287	PTGIR	P43119
MOL000287	FNTA	P49354
MOL000287	PRKCH	P24723
MOL000287	PTPN1	P18031
MOL000287	PTPN6	P29350
MOL000287	PTPN11	Q06124
MOL000287	PTPRF	P10586
MOL000287	SLC6A4	P31645
MOL000287	SIGMAR1	Q99720
MOL000287	SLC22A6	Q4U2R8
MOL000287	FDFT1	P37268
MOL000287	SRD5A2	P31213
MOL000287	SREBF2	Q12772
MOL000287	SAE1	Q9UBE0
MOL000287	PTPN2	P17706

MOL000287	TERT	O14746
MOL000287	SHBG	P04278
MOL000287	TNF	P01375
MOL000287	TLR9	Q9NR96
MOL000287	TRPV1	Q8NER1
MOL000287	UBA2	Q9UBT2
MOL000287	FNTB	P49356
MOL000289	HSD11B1	P28845
MOL000289	HSD11B2	P80365
MOL000289	ALOX5AP	P20292
MOL000289	ACHE	P22303
MOL000289	AKR1B10	O60218
MOL000289	AKR1C1	Q04828
MOL000289	AKR1C2	P52895
MOL000289	AKR1C3	P42330
MOL000289	FAAH	O00519
MOL000289	AR	P10275
MOL000289	ACE	P12821
MOL000289	ALOX5	P09917
MOL000289	CALCRL	Q16602
MOL000289	CASR	P41180
MOL000289	CNR1	P21554
MOL000289	CES2	O00748
MOL000289	CTRC	Q99895
MOL000289	SERPINA6	P08185
MOL000289	PTGS2	P35354
MOL000289	CYSLTR1	Q9Y271
MOL000289	CYP17A1	P05093
MOL000289	CYP19A1	P11511
MOL000289	CYP2C19	P33261
MOL000289	PLA2G4A	P47712
MOL000289	EPAS1	Q99814
MOL000289	EDNRB	P24530
MOL000289	EPHB4	P54760
MOL000289	EPHB6	O15197
MOL000289	EPHA1	P21709
MOL000289	EPHA2	P29317
MOL000289	EPHA3	P29320
MOL000289	EPHA4	P54764
MOL000289	EPHA5	P54756
MOL000289	EPHA6	Q9UF33
MOL000289	EPHA7	Q15375
MOL000289	EPHA8	P29322
MOL000289	EPHB1	P54762

MOL000289	EPHB2	P29323
MOL000289	EPHB3	P54753
MOL000289	HSD17B2	P37059
MOL000289	PTGDR2	Q9Y5Y4
MOL000289	NR3C1	P04150
MOL000289	GSK3B	P49841
MOL000289	HMGCR	P04035
MOL000289	IKBKB	O14920
MOL000289	ITGB1	P05556
MOL000289	IL1B	P01584
MOL000289	NR1H3	Q13133
MOL000289	NR3C2	P08235
MOL000289	NLRP3	Q96P20
MOL000289	NOS2	P35228
MOL000289	ABCB1	P08183
MOL000289	PLA2G2A	P14555
MOL000289	PGR	P06401
MOL000289	PREP	P48147
MOL000289	PTGES	O14684
MOL000289	PTGER2	P43116
MOL000289	PTGIR	P43119
MOL000289	FNTA	P49354
MOL000289	PTPN1	P18031
MOL000289	S1PR2	O95136
MOL000289	PTPN2	P17706
MOL000289	TERT	O14746
MOL000289	SHBG	P04278
MOL000289	F2	P00734
MOL000289	TBXA2R	P21731
MOL000289	TBXAS1	P24557
MOL000289	TNF	P01375
MOL000289	PRSS1	P07477
MOL000289	AGTR1	P30556
MOL000289	VDR	P11473
MOL000289	FNTB	P49356
MOL000289	ITGA4	P13612
MOL000290	HSD11B1	P28845
MOL000290	AR	P10275
MOL000290	BCL2	P10415
MOL000290	CCR1	P32246
MOL000290	MAPK10	P53779
MOL000290	PLA2G4A	P47712
MOL000290	TOP2A	P11388
MOL000290	EDNRA	P25101

MOL000290	EDNRB	P24530
MOL000290	ESR1	P03372
MOL000290	ESR2	Q92731
MOL000290	NR3C1	P04150
MOL000290	HMGCR	P04035
MOL000290	IMPDH1	P20839
MOL000290	IMPDH2	P12268
MOL000290	LTB4R	Q15722
MOL000290	NR1H3	Q13133
MOL000290	MMP1	P03956
MOL000290	MMP2	P08253
MOL000290	MMP3	P08254
MOL000290	GRM2	Q14416
MOL000290	NOS2	P35228
MOL000290	PPARA	Q07869
MOL000290	PPARD	Q03181
MOL000290	PPARG	P37231
MOL000290	PDE2A	O00408
MOL000290	PIK3CA	P42336
MOL000290	PGR	P06401
MOL000290	PTGDR	Q13258
MOL000290	PTGER1	P34995
MOL000290	PTGER2	P43116
MOL000290	PTGER3	P43115
MOL000290	PTGER4	P35408
MOL000290	PTGFR	P43088
MOL000290	FNTA	P49354
MOL000290	PTPN1	P18031
MOL000290	SHBG	P04278
MOL000290	TYMS	P04818
MOL000290	THRA	P10827
MOL000290	THRB	P10828
MOL000290	TNF	P01375
MOL000290	VDR	P11473
MOL000290	FNTB	P49356
MOL000291	HSD11B1	P28845
MOL000291	AR	P10275
MOL000291	CES2	O00748
MOL000291	CCR1	P32246
MOL000291	POLB	P06746
MOL000291	TOP1	P11387
MOL000291	TOP2A	P11388
MOL000291	ESR1	P03372
MOL000291	NR3C1	P04150

MOL000291	HAO1	Q9UJM8
MOL000291	IMPDH1	P20839
MOL000291	IMPDH2	P12268
MOL000291	LTB4R	Q15722
MOL000291	NR1H3	Q13133
MOL000291	PPARA	Q07869
MOL000291	PPARD	Q03181
MOL000291	PPARG	P37231
MOL000291	PDE2A	O00408
MOL000291	PGR	P06401
MOL000291	PTGDR	Q13258
MOL000291	PTGER1	P34995
MOL000291	PTGER2	P43116
MOL000291	PTGER3	P43115
MOL000291	PTGER4	P35408
MOL000291	PTGFR	P43088
MOL000291	FNTA	P49354
MOL000291	PTPN1	P18031
MOL000291	SHBG	P04278
MOL000291	TYMS	P04818
MOL000291	THRA	P10827
MOL000291	THRB	P10828
MOL000291	TNF	P01375
MOL000291	VDR	P11473
MOL000291	FNTB	P49356
MOL000292	HSD11B1	P28845
MOL000292	HSD11B2	P80365
MOL000292	ALOX5AP	P20292
MOL000292	FAAH	O00519
MOL000292	AR	P10275
MOL000292	ALOX12	P18054
MOL000292	ALOX15	P16050
MOL000292	ALOX5	P09917
MOL000292	ENPP2	Q13822
MOL000292	CES2	O00748
MOL000292	CPB1	P15086
MOL000292	SERPINA6	P08185
MOL000292	CYP17A1	P05093
MOL000292	CYP19A1	P11511
MOL000292	CYP51A1	Q16850
MOL000292	PLA2G4A	P47712
MOL000292	POLB	P06746
MOL000292	TOP1	P11387
MOL000292	TOP2A	P11388

MOL000292	SLC6A3	Q01959
MOL000292	CDC25A	P30304
MOL000292	ESR1	P03372
MOL000292	ESR2	Q92731
MOL000292	FABP4	P15090
MOL000292	FABP5	Q01469
MOL000292	FABP3	P05413
MOL000292	FABP1	P07148
MOL000292	FFAR1	O14842
MOL000292	FFAR2	O15552
MOL000292	PTGDR2	Q9Y5Y4
MOL000292	GCGR	P47871
MOL000292	NR3C1	P04150
MOL000292	G6PD	P11413
MOL000292	HMGCR	P04035
MOL000292	ITGA4	P13612
MOL000292	ITGAV	P06756
MOL000292	LTB4R	Q15722
MOL000292	NR1H3	Q13133
MOL000292	MMP2	P08253
MOL000292	MMP9	P14780
MOL000292	GRM2	Q14416
MOL000292	NR3C2	P08235
MOL000292	GYS1	P13807
MOL000292	NOS2	P35228
MOL000292	OXTR	P30559
MOL000292	PPARA	Q07869
MOL000292	PPARD	Q03181
MOL000292	PPARG	P37231
MOL000292	PDE10A	Q9Y233
MOL000292	PGR	P06401
MOL000292	PREP	P48147
MOL000292	PTGES	O14684
MOL000292	PTGER1	P34995
MOL000292	PTGER2	P43116
MOL000292	PTGER4	P35408
MOL000292	PTGIR	P43119
MOL000292	FNTA	P49354
MOL000292	PTPN1	P18031
MOL000292	PTPN6	P29350
MOL000292	PTPN11	Q06124
MOL000292	SIGMAR1	Q99720
MOL000292	SRD5A2	P31213
MOL000292	PTPN2	P17706

MOL000292	TERT	O14746
MOL000292	SHBG	P04278
MOL000292	TBXAS1	P24557
MOL000292	TNF	P01375
MOL000292	TRPM8	Q7Z2W7
MOL000292	AVPR1A	P37288
MOL000292	AVPR2	P30518
MOL000292	VDR	P11473
MOL000292	ITGB3	P05106
MOL000292	FNTB	P49356
MOL000296	HSD11B1	P28845
MOL000296	HSD11B2	P80365
MOL000296	ALOX5AP	P20292
MOL000296	SCD	O00767
MOL000296	ADH1B	P00325
MOL000296	ADH1C	P00326
MOL000296	AKR1B10	O60218
MOL000296	ADRA1B	P35368
MOL000296	FAAH	O00519
MOL000296	AR	P10275
MOL000296	ALOX5	P09917
MOL000296	BACE1	P56817
MOL000296	NR1H4	Q96RI1
MOL000296	SLC10A1	Q14973
MOL000296	BCHE	P06276
MOL000296	CES2	O00748
MOL000296	CD81	P60033
MOL000296	PDE3A	Q14432
MOL000296	SERPINA6	P08185
MOL000296	PTGS1	P23219
MOL000296	PTGS2	P35354
MOL000296	CYP17A1	P05093
MOL000296	CYP19A1	P11511
MOL000296	CYP51A1	Q16850
MOL000296	POLB	P06746
MOL000296	TOP1	P11387
MOL000296	TOP2A	P11388
MOL000296	SLC6A3	Q01959
MOL000296	CDC25A	P30304
MOL000296	CDC25B	P30305
MOL000296	ESR1	P03372
MOL000296	ESR2	Q92731
MOL000296	FABP4	P15090
MOL000296	FABP5	Q01469

MOL000296	FABP3	P05413
MOL000296	FABP1	P07148
MOL000296	FFAR1	O14842
MOL000296	PTGDR2	Q9Y5Y4
MOL000296	GABRA1	P14867
MOL000296	GABRA2	P47869
MOL000296	GABRA3	P34903
MOL000296	GABRA5	P31644
MOL000296	GABRA6	Q16445
MOL000296	NR3C1	P04150
MOL000296	G6PD	P11413
MOL000296	GRIA2	P42262
MOL000296	GRIK1	P39086
MOL000296	GRIK2	Q13002
MOL000296	GLUL	P15104
MOL000296	GPBAR1	Q8TDU6
MOL000296	HMGCR	P04035
MOL000296	IGHG1	P01857
MOL000296	SLC10A2	Q12908
MOL000296	IL6	P05231
MOL000296	LTB4R	Q15722
MOL000296	ACP1	P24666
MOL000296	NR1H3	Q13133
MOL000296	LYZ	P61626
MOL000296	NR3C2	P08235
MOL000296	CHRM1	P11229
MOL000296	CHRM2	P08172
MOL000296	CHRM3	P20309
MOL000296	NOS2	P35228
MOL000296	NCOA2	Q15596
MOL000296	RORA	P35398
MOL000296	RORC	P51449
MOL000296	PPARA	Q07869
MOL000296	PPARD	Q03181
MOL000296	PPARG	P37231
MOL000296	PDE4D	Q08499
MOL000296	PLA2G1B	P04054
MOL000296	PGR	P06401
MOL000296	PREP	P48147
MOL000296	PTGES	O14684
MOL000296	PTGDR	Q13258
MOL000296	PTGER1	P34995
MOL000296	PTGER2	P43116
MOL000296	PTGER4	P35408

MOL000296	PTGIR	P43119
MOL000296	FNTA	P49354
MOL000296	PRKCH	P24723
MOL000296	PTPN1	P18031
MOL000296	PTPN6	P29350
MOL000296	PTPN11	Q06124
MOL000296	PTPRF	P10586
MOL000296	RXRA	P19793
MOL000296	SIGMAR1	Q99720
MOL000296	SCN5A	Q14524
MOL000296	SLC6A2	P23975
MOL000296	SRD5A2	P31213
MOL000296	PTPN2	P17706
MOL000296	TERT	O14746
MOL000296	SHBG	P04278
MOL000296	TLR9	Q9NR96
MOL000296	FNTB	P49356
MOL000300	HSD11B1	P28845
MOL000300	HSD11B2	P80365
MOL000300	ALOX5AP	P20292
MOL000300	SCD	O00767
MOL000300	AKR1B10	O60218
MOL000300	FAAH	O00519
MOL000300	AR	P10275
MOL000300	BCL2L1	Q07817
MOL000300	ALOX12	P18054
MOL000300	ALOX5	P09917
MOL000300	BCHE	P06276
MOL000300	CES2	O00748
MOL000300	CD81	P60033
MOL000300	SERPINA6	P08185
MOL000300	PTGS1	P23219
MOL000300	CYSLTR1	Q9Y271
MOL000300	CYP17A1	P05093
MOL000300	CYP19A1	P11511
MOL000300	CYP51A1	Q16850
MOL000300	POLB	P06746
MOL000300	TOP1	P11387
MOL000300	TOP2A	P11388
MOL000300	CDC25A	P30304
MOL000300	CDC25B	P30305
MOL000300	EDNRA	P25101
MOL000300	ESR1	P03372
MOL000300	ESR2	Q92731

MOL000300	FABP4	P15090
MOL000300	FABP5	Q01469
MOL000300	FABP3	P05413
MOL000300	FABP1	P07148
MOL000300	PTGDR2	Q9Y5Y4
MOL000300	NR3C1	P04150
MOL000300	G6PD	P11413
MOL000300	HMGCR	P04035
MOL000300	HAO1	Q9UJM8
MOL000300	IKBKB	O14920
MOL000300	LTB4R	Q15722
MOL000300	ACP1	P24666
MOL000300	NR1H3	Q13133
MOL000300	MAPK3	P27361
MOL000300	NOS2	P35228
MOL000300	RORA	P35398
MOL000300	RORC	P51449
MOL000300	MDM2	Q00987
MOL000300	PPARA	Q07869
MOL000300	PPARD	Q03181
MOL000300	PPARG	P37231
MOL000300	PDE4D	Q08499
MOL000300	PLA2G1B	P04054
MOL000300	PLCG1	P19174
MOL000300	PGR	P06401
MOL000300	PREP	P48147
MOL000300	PTGES	O14684
MOL000300	PTGER1	P34995
MOL000300	PTGER2	P43116
MOL000300	FNTA	P49354
MOL000300	PRKCH	P24723
MOL000300	PTPN1	P18031
MOL000300	PTPN6	P29350
MOL000300	PTPN11	Q06124
MOL000300	RASGRP3	Q8IV61
MOL000300	PTPRF	P10586
MOL000300	SIGMAR1	Q99720
MOL000300	SRD5A2	P31213
MOL000300	SAE1	Q9UBE0
MOL000300	PTPN2	P17706
MOL000300	TERT	O14746
MOL000300	SHBG	P04278
MOL000300	TNF	P01375
MOL000300	VDR	P11473

MOL000300	FNTB	P49356
MOL000300	UBA2	Q9UBT2
MOL000354	ACHE	P22303
MOL000354	ADORA1	P30542
MOL000354	ADORA2A	P29274
MOL000354	AKR1A1	P14550
MOL000354	AKR1B10	O60218
MOL000354	AKR1C1	Q04828
MOL000354	AKR1C2	P52895
MOL000354	AKR1C4	P17516
MOL000354	AKR1C3	P42330
MOL000354	AKR1B1	P15121
MOL000354	ALK	Q9UM73
MOL000354	MAOB	P27338
MOL000354	AR	P10275
MOL000354	ALOX12	P18054
MOL000354	ALOX15	P16050
MOL000354	ALOX5	P09917
MOL000354	ARG1	P05089
MOL000354	AHR	P35869
MOL000354	ABCG2	Q9UNQ0
MOL000354	APP	P05067
MOL000354	BACE1	P56817
MOL000354	CALM1	P62158
MOL000354	CAMK2B	Q13554
MOL000354	CA1	P00915
MOL000354	CA2	P00918
MOL000354	CA3	P07451
MOL000354	CA4	P22748
MOL000354	CA9	Q16790
MOL000354	CA5A	P35218
MOL000354	CA6	P23280
MOL000354	CA7	P43166
MOL000354	CA12	O43570
MOL000354	CA13	Q8N1Q1
MOL000354	CA14	Q9ULX7
MOL000354	CSNK2A1	P68400
MOL000354	CDK2	P24941
MOL000354	F7	P08709
MOL000354	CCNA2	P20248
MOL000354	CDK1	P06493
MOL000354	CCNB3	Q8WWL7
MOL000354	CDK5R1	Q15078
MOL000354	CDK6	Q00534

MOL000354	CYP19A1	P11511
MOL000354	CYP1B1	Q16678
MOL000354	DAPK1	P53355
MOL000354	DPP4	P27487
MOL000354	TOP2A	P11388
MOL000354	APEX1	P27695
MOL000354	MPG	P29372
MOL000354	DRD4	P21917
MOL000354	EGFR	P00533
MOL000354	HSD17B1	P14061
MOL000354	HSD17B2	P37059
MOL000354	ESR1	P03372
MOL000354	ESR2	Q92731
MOL000354	ESRRA	P11474
MOL000354	PTK2	Q05397
MOL000354	GABRA1	P14867
MOL000354	GRIA2	P42262
MOL000354	PYGM	P11217
MOL000354	GSK3B	P49841
MOL000354	GLO1	Q04760
MOL000354	GPR35	Q9HC97
MOL000354	HSP90AB1	P08238
MOL000354	MET	P08581
MOL000354	MCL1	Q07820
MOL000354	INSR	P06213
MOL000354	IGF1R	P08069
MOL000354	CXCR1	P25024
MOL000354	PYGL	P06737
MOL000354	CD38	P28907
MOL000354	KDM4E	B2RXH2
MOL000354	MMP12	P39900
MOL000354	MMP13	P45452
MOL000354	MMP2	P08253
MOL000354	MMP3	P08254
MOL000354	MMP9	P14780
MOL000354	MAPT	P10636
MOL000354	MAPK14	Q16539
MOL000354	MAOA	P21397
MOL000354	PRKACA	P17612
MOL000354	PTPN1	P18031
MOL000354	ABCC1	P33527
MOL000354	MPO	P05164
MOL000354	MYLK	Q15746
MOL000354	NOX4	Q9NPH5

MOL000354	NCF1	P14598
MOL000354	NOS3	P29474
MOL000354	NOS1	P29475
MOL000354	NUAK1	O60285
MOL000354	NCOA1	Q15788
MOL000354	NCOA2	Q15596
MOL000354	OLR1	P78380
MOL000354	PPARD	Q03181
MOL000354	PPARG	P37231
MOL000354	ABCB1	P08183
MOL000354	PIK3CG	P48736
MOL000354	PLA2G1B	P04054
MOL000354	PIK3R1	P27986
MOL000354	PLG	P00747
MOL000354	PARP1	P09874
MOL000354	PTGS1	P23219
MOL000354	PTGS2	P35354
MOL000354	PKN1	Q16512
MOL000354	PIM1	P11309
MOL000354	PTPRS	Q13332
MOL000354	AKT1	P31749
MOL000354	AURKB	Q96GD4
MOL000354	CHEK1	O14757
MOL000354	NEK2	P51955
MOL000354	NEK6	Q9HC98
MOL000354	PLK1	P53350
MOL000354	SLC22A12	Q96S37
MOL000354	TNKS2	Q9H2K2
MOL000354	TERT	O14746
MOL000354	F2	P00734
MOL000354	RELA	Q04206
MOL000354	TTR	P02766
MOL000354	PRSS1	P07477
MOL000354	TYR	P14679
MOL000354	FLT3	P36888
MOL000354	AXL	P30530
MOL000354	SRC	P12931
MOL000354	SYK	P43405
MOL000354	KDR	P35968
MOL000354	AVPR2	P30518
MOL000354	XDH	P47989
MOL000354	CDK5	Q00535
MOL000354	CCNB1	P14635
MOL000354	CCNB2	O95067

MOL000358	HSD11B1	P28845
MOL000358	HTR2A	P28223
MOL000358	ACHE	P22303
MOL000358	ADRA1A	P35348
MOL000358	ADRA1B	P35368
MOL000358	AR	P10275
MOL000358	DHCR7	Q9UBM7
MOL000358	BAX	Q07812
MOL000358	BCL2	P10415
MOL000358	ADRB2	P07550
MOL000358	BCHE	P06276
MOL000358	CES2	O00748
MOL000358	CASP3	P42574
MOL000358	CASP8	Q14790
MOL000358	CASP9	P55211
MOL000358	PDE3A	Q14432
MOL000358	SERPINA6	P08185
MOL000358	CYP17A1	P05093
MOL000358	CYP19A1	P11511
MOL000358	CYP2C19	P33261
MOL000358	CYP51A1	Q16850
MOL000358	POLB	P06746
MOL000358	DRD1	P21728
MOL000358	ESR1	P03372
MOL000358	ESR2	Q92731
MOL000358	GABRA1	P14867
MOL000358	GABRA2	P47869
MOL000358	GABRA3	P34903
MOL000358	GABRA5	P31644
MOL000358	NR3C1	P04150
MOL000358	G6PD	P11413
MOL000358	GLRA1	P23415
MOL000358	HSP90AB1	P08238
MOL000358	HMGCR	P04035
MOL000358	NR1H3	Q13133
MOL000358	NR1H2	P55055
MOL000358	MAP2	P11137
MOL000358	PRKACA	P17612
MOL000358	CHRM1	P11229
MOL000358	CHRM2	P08172
MOL000358	CHRM3	P20309
MOL000358	CHRM4	P08173
MOL000358	OPRM1	P35372
MOL000358	CHRNA7	P36544

MOL000358	CHRNA2	Q15822
MOL000358	NPC1L1	Q9UHC9
MOL000358	NOS2	P35228
MOL000358	SLC6A2	P23975
MOL000358	NCOA2	Q15596
MOL000358	RORA	P35398
MOL000358	RORC	P51449
MOL000358	NR1I3	Q14994
MOL000358	PPARD	Q03181
MOL000358	PPARG	P37231
MOL000358	PIK3CG	P48736
MOL000358	KCNH2	Q12809
MOL000358	PGR	P06401
MOL000358	PTGES	O14684
MOL000358	PTGS1	P23219
MOL000358	PTGS2	P35354
MOL000358	PTGER1	P34995
MOL000358	PTGER2	P43116
MOL000358	PRKCA	P17252
MOL000358	PTPN1	P18031
MOL000358	PTPN6	P29350
MOL000358	SLC6A4	P31645
MOL000358	PON1	P27169
MOL000358	SCN5A	Q14524
MOL000358	SQLE	Q14534
MOL000358	FDFT1	P37268
MOL000358	SREBF2	Q12772
MOL000358	PTPN2	P17706
MOL000358	SHBG	P04278
MOL000358	TBXAS1	P24557
MOL000358	FOS	P01100
MOL000358	TGFB1	P01137
MOL000358	UGT2B7	P16662
MOL000358	VDR	P11473
MOL000359	HSD11B1	P28845
MOL000359	ACHE	P22303
MOL000359	AR	P10275
MOL000359	DHCR7	Q9UBM7
MOL000359	BCHE	P06276
MOL000359	CES2	O00748
MOL000359	SERPINA6	P08185
MOL000359	CYP17A1	P05093
MOL000359	CYP19A1	P11511
MOL000359	CYP2C19	P33261

MOL000359	CYP51A1	Q16850
MOL000359	POLB	P06746
MOL000359	ESR1	P03372
MOL000359	ESR2	Q92731
MOL000359	NR3C1	P04150
MOL000359	G6PD	P11413
MOL000359	GLRA1	P23415
MOL000359	HMGCR	P04035
MOL000359	NR1H3	Q13133
MOL000359	NR1H2	P55055
MOL000359	NR3C2	P08235
MOL000359	CHRM2	P08172
MOL000359	NPC1L1	Q9UHC9
MOL000359	NOS2	P35228
MOL000359	SLC6A2	P23975
MOL000359	NCOA2	Q15596
MOL000359	RORA	P35398
MOL000359	RORC	P51449
MOL000359	NR1I3	Q14994
MOL000359	PPARD	Q03181
MOL000359	PPARG	P37231
MOL000359	PGR	P06401
MOL000359	PTGES	O14684
MOL000359	PTGER1	P34995
MOL000359	PTGER2	P43116
MOL000359	PTPN1	P18031
MOL000359	PTPN6	P29350
MOL000359	SLC6A4	P31645
MOL000359	SQLE	Q14534
MOL000359	FDFT1	P37268
MOL000359	SREBF2	Q12772
MOL000359	PTPN2	P17706
MOL000359	SHBG	P04278
MOL000359	TBXAS1	P24557
MOL000359	UGT2B7	P16662
MOL000359	VDR	P11473
MOL000371	HTR3A,	P46098
MOL000371	ACHE	P22303
MOL000371	ADORA2A	P29274
MOL000371	ADRA1B	P35368
MOL000371	ADRA1D	P25100
MOL000371	ADRA2C	P18825
MOL000371	AR	P10275
MOL000371	ADRB1	P08588

MOL000371	ADRB2	P07550
MOL000371	CALM1	P62158
MOL000371	CNR1	P21554
MOL000371	CSNK1D	P48730
MOL000371	CCNA2	P20248
MOL000371	CCNC	P24863
MOL000371	CDK8	P49336
MOL000371	PDE3A	Q14432
MOL000371	CDK2	P24941
MOL000371	CCNE2	O96020
MOL000371	CDK4	P11802
MOL000371	CCND3	P30281
MOL000371	CCND1	P24385
MOL000371	CYP11B1	P15538
MOL000371	CYP11B2	P19099
MOL000371	CYP19A1	P11511
MOL000371	DHFR	P00374
MOL000371	MAP2K1	Q02750
MOL000371	CLK2	P49760
MOL000371	CLK4	Q9HAZ1
MOL000371	TTK	P33981
MOL000371	DYRK1B	Q9Y463
MOL000371	CLK1	P49759
MOL000371	HSD17B2	P37059
MOL000371	ESR1	P03372
MOL000371	SLC1A3	P43003
MOL000371	FGFR1	P11362
MOL000371	GABRA1	P14867
MOL000371	GABRA2	P47869
MOL000371	GABRA3	P34903
MOL000371	GABRA5	P31644
MOL000371	GABRB3	P28472
MOL000371	GABRG2	P18507
MOL000371	PGGT1B	P53609
MOL000371	GSK3B	P49841
MOL000371	MET	P08581
MOL000371	MCL1	Q07820
MOL000371	IKBKB	O14920
MOL000371	LRRK2	Q5S007
MOL000371	ELANE	P08246
MOL000371	SLC27A1	Q6PCB7
MOL000371	KDM5B	Q9UGL1
MOL000371	MAPKAPK2	P49137
MOL000371	MCHR1	Q99705

MOL000371	GRM1	Q13255
MOL000371	MARS	P56192
MOL000371	CHRM1	P11229
MOL000371	CHRM3	P20309
MOL000371	OPRM1	P35372
MOL000371	SIRT2	Q8IXJ6
MOL000371	NTRK1	P04629
MOL000371	NPBWR1	P48145
MOL000371	NOS2	P35228
MOL000371	NOS1	P29475
MOL000371	NCOA2	Q15596
MOL000371	HCRTR1	O43613
MOL000371	HCRTR2	O43614
MOL000371	P2RX7	Q99572
MOL000371	BRPF1	P55201
MOL000371	ABCB1	P08183
MOL000371	PDE10A	Q9Y233
MOL000371	PDE3B	Q13370
MOL000371	PDE5A	O76074
MOL000371	PGK1	P00558
MOL000371	PIK3CA	P42336
MOL000371	PIK3CB	P42338
MOL000371	PIK3CD	O00329
MOL000371	PIK3CG	P48736
MOL000371	PDGFRB	P09619
MOL000371	PARP1	P09874
MOL000371	PTGS1	P23219
MOL000371	PTGS2	P35354
MOL000371	PRKCB	P05771
MOL000371	TGM2	P21980
MOL000371	PTPN1	P18031
MOL000371	PTP4A3	O75365
MOL000371	MERTK	Q12866
MOL000371	PDK1	Q15118
MOL000371	NQO2	P16083
MOL000371	RGS4	P49798
MOL000371	RXRA	P19793
MOL000371	ROCK2	O75116
MOL000371	RPS6KB1	P23443
MOL000371	RPS6KA3	P51812
MOL000371	PPP1CA	P62136
MOL000371	AURKA	O14965
MOL000371	BRAF	P15056
MOL000371	CHEK1	O14757

MOL000371	MTOR	P42345
MOL000371	PIM3	Q86V86
MOL000371	PLK2	Q9NYY3
MOL000371	PLK3	Q9H4B4
MOL000371	SCN5A	Q14524
MOL000371	SCN9A	Q15858
MOL000371	KIT	P10721
MOL000371	F2	P00734
MOL000371	TBXAS1	P24557
MOL000371	PRSS1	P07477
MOL000371	TUBB1	Q9H4B7
MOL000371	TUBB3	Q13509
MOL000371	ABL1	P00519
MOL000371	FLT3	P36888
MOL000371	SRC	P12931
MOL000371	SYK	P43405
MOL000371	TDP2	O95551
MOL000371	PLAU	P00749
MOL000371	TRPV1	Q8NER1
MOL000371	KDR	P35968
MOL000371	CCNA1	P78396
MOL000371	FNTA	P49354
MOL000371	CCNE1	P24864
MOL000371	CCND2	P30279
MOL000371	GABRA6	Q16445
MOL000378	HTR2A	P28223
MOL000378	ALOX5AP	P20292
MOL000378	ADAMTS5	Q9UNA0
MOL000378	ALDH3A1	P30838
MOL000378	ALPL	P05186
MOL000378	ADRA1A	P35348
MOL000378	ADRA1B	P35368
MOL000378	ADRA1D	P25100
MOL000378	ADRA2C	P18825
MOL000378	FAAH	O00519
MOL000378	AR	P10275
MOL000378	ALOX12	P18054
MOL000378	ALOX15	P16050
MOL000378	ALOX15B	O15296
MOL000378	ADRB1	P08588
MOL000378	ADRB2	P07550
MOL000378	BACE1	P56817
MOL000378	KCNMA1	Q12791
MOL000378	CALM1	P62158

MOL000378	CA14	Q9ULX7
MOL000378	CDK9	P50750
MOL000378	CDK2	P24941
MOL000378	PDE3A	Q14432
MOL000378	F3	P13726
MOL000378	F10	P00742
MOL000378	CCNA2	P20248
MOL000378	CDK1	P06493
MOL000378	CDK4	P11802
MOL000378	CDK7	P50613
MOL000378	OPRD1	P41143
MOL000378	DPP4	P27487
MOL000378	DRD1	P21728
MOL000378	DRD2	P14416
MOL000378	CDC25A	P30304
MOL000378	CDC25B	P30305
MOL000378	CDC25C	P30307
MOL000378	CLK2	P49760
MOL000378	CLK3	P49761
MOL000378	CLK4	Q9HAZ1
MOL000378	DYRK1B	Q9Y463
MOL000378	CLK1	P49759
MOL000378	HSD17B3	P37058
MOL000378	ESR1	P03372
MOL000378	ESR2	Q92731
MOL000378	PTK2	Q05397
MOL000378	GABRA1	P14867
MOL000378	GSK3B	P49841
MOL000378	HSP90AB1	P08238
MOL000378	MET	P08581
MOL000378	IMPDH1	P20839
MOL000378	IMPDH2	P12268
MOL000378	RET	P07949
MOL000378	LDHB	P07195
MOL000378	MKNK1	Q9BUB5
MOL000378	MMP1	P03956
MOL000378	MAPK14	Q16539
MOL000378	MAOB	P27338
MOL000378	PRKACA	P17612
MOL000378	CHRM1	P11229
MOL000378	CHRM2	P08172
MOL000378	CHRM3	P20309
MOL000378	CHRM4	P08173
MOL000378	CHRM5	P08912

MOL000378	NDUFA4	O00483
MOL000378	NOX4	Q9NPH5
MOL000378	CHRNA7	P36544
MOL000378	NOS3	P29474
MOL000378	NOS1	P29475
MOL000378	NCOA2	Q15596
MOL000378	PPARG	P37231
MOL000378	PDE10A	Q9Y233
MOL000378	PDE2A	O00408
MOL000378	PIK3CA	P42336
MOL000378	KCNH2	Q12809
MOL000378	PTGS1	P23219
MOL000378	PTGS2	P35354
MOL000378	PIM1	P11309
MOL000378	RXRA	P19793
MOL000378	RXRB	P28702
MOL000378	AURKB	Q96GD4
MOL000378	BRAF	P15056
MOL000378	CHEK1	O14757
MOL000378	MTOR	P42345
MOL000378	PLK1	P53350
MOL000378	RAF1	P04049
MOL000378	WEE1	P30291
MOL000378	SIGMAR1	Q99720
MOL000378	SCN5A	Q14524
MOL000378	SLC6A3	Q01959
MOL000378	SLC6A4	P31645
MOL000378	KIT	P10721
MOL000378	TGFBR1	P36897
MOL000378	F2	P00734
MOL000378	TLR9	Q9NR96
MOL000378	TRPM8	Q7Z2W7
MOL000378	PRSS1	P07477
MOL000378	ABL1	P00519
MOL000378	SRC	P12931
MOL000378	SYK	P43405
MOL000378	KDR	P35968
MOL000378	CCNB1	P14635
MOL000378	CCNA1	P78396
MOL000378	CCNH	P51946
MOL000378	CCNT1	O60563
MOL000379	TOP2A	P11388
MOL000379	NCOA2	Q15596
MOL000379	PTGS2	P35354

MOL000380	HTR3A,	P46098
MOL000380	ACHE	P22303
MOL000380	ADRA1B	P35368
MOL000380	ADRA1D	P25100
MOL000380	ADRB2	P07550
MOL000380	CALM1	P62158
MOL000380	ESR1	P03372
MOL000380	GABRA1	P14867
MOL000380	HSP90AB1	P08238
MOL000380	CHRM1	P11229
MOL000380	CHRM3	P20309
MOL000380	CHRM4	P08173
MOL000380	CHRNA7	P36544
MOL000380	NOS2	P35228
MOL000380	NCOA1	Q15788
MOL000380	NCOA2	Q15596
MOL000380	PTGS1	P23219
MOL000380	PTGS2	P35354
MOL000380	RXRA	P19793
MOL000380	SCN5A	Q14524
MOL000380	F2	P00734
MOL000380	PRSS1	P07477
MOL000387	ABCG2	Q9UNQ0
MOL000387	KCNMA1	Q12791
MOL000387	TOP2A	P11388
MOL000387	EGFR	P00533
MOL000387	PTK2	Q05397
MOL000387	GSK3B	P49841
MOL000387	HSP90AB1	P08238
MOL000387	MET	P08581
MOL000387	OXTR	P30559
MOL000387	PDE8B	O95263
MOL000387	PTGS1	P23219
MOL000387	PTGS2	P35354
MOL000387	JUN	P05412
MOL000387	F2	P00734
MOL000387	JAK3	P52333
MOL000387	SYK	P43405
MOL000387	KDR	P35968
MOL000387	AVPR1A	P37288
MOL000387	AVPR2	P30518
MOL000392	HSD3B1	P14060
MOL000392	HSD3B2	P26439
MOL000392	PFKFB3	Q16875

MOL000392	ACHE	P22303
MOL000392	ADORA1	P30542
MOL000392	ADORA2A	P29274
MOL000392	ALDH2	P05091
MOL000392	ADRA1A	P35348
MOL000392	MAOB	P27338
MOL000392	AR	P10275
MOL000392	ALOX12	P18054
MOL000392	ALOX15	P16050
MOL000392	ATP5F1E	P56381
MOL000392	ABCG2	Q9UNQ0
MOL000392	ADRB2	P07550
MOL000392	DPEP1	P16444
MOL000392	BACE1	P56817
MOL000392	CALM1	P62158
MOL000392	PKIA	P61925
MOL000392	CA1	P00915
MOL000392	CA2	P00918
MOL000392	CA3	P07451
MOL000392	CA4	P22748
MOL000392	CA9	Q16790
MOL000392	CA5A	P35218
MOL000392	CA5B	Q9Y2D0
MOL000392	CA6	P23280
MOL000392	CA7	P43166
MOL000392	CA12	O43570
MOL000392	CA13	Q8N1Q1
MOL000392	CA14	Q9ULX7
MOL000392	CBR1	P16152
MOL000392	CDK2	P24941
MOL000392	PDE3A	Q14432
MOL000392	CCNA2	P20248
MOL000392	PTGS1	P23219
MOL000392	CYP19A1	P11511
MOL000392	DHODH	Q02127
MOL000392	DPP4	P27487
MOL000392	ERCC5	P28715
MOL000392	EGFR	P00533
MOL000392	HSD17B1	P14061
MOL000392	HSD17B2	P37059
MOL000392	ESR1	P03372
MOL000392	ESR2	Q92731
MOL000392	ESRRA	P11474
MOL000392	ESRRB	O95718

MOL000392	FEN1	P39748
MOL000392	GSK3B	P49841
MOL000392	HSP90AB1	P08238
MOL000392	IL2	P60568
MOL000392	IL4	P05112
MOL000392	MIF	P14174
MOL000392	MGAM	O43451
MOL000392	MAPK14	Q16539
MOL000392	MAOA	P21397
MOL000392	PRKACA	P17612
MOL000392	CHRM1	P11229
MOL000392	SIRT1	Q96EB6
MOL000392	MT-ND6	P03923
MOL000392	NOX4	Q9NPH5
MOL000392	NOS3	P29474
MOL000392	NOS1	P29475
MOL000392	SLC6A2	P23975
MOL000392	PPARG	P37231
MOL000392	PPARA	Q07869
MOL000392	ABCB1	P08183
MOL000392	PTGS2	P35354
MOL000392	PTPN1	P18031
MOL000392	PIM1	P11309
MOL000392	PTPRS	Q13332
MOL000392	RXRA	P19793
MOL000392	CHEK1	O14757
MOL000392	RAF1	P04049
MOL000392	HTR2A	P28223
MOL000392	HTR2C	P28335
MOL000392	PON1	P27169
MOL000392	SLC6A3	Q01959
MOL000392	SLC6A4	P31645
MOL000392	STS	P08842
MOL000392	TNKS	O95271
MOL000392	TNKS2	Q9H2K2
MOL000392	F2	P00734
MOL000392	TBXAS1	P24557
MOL000392	TLR9	Q9NR96
MOL000392	FOS	P01100
MOL000392	PRSS1	P07477
MOL000392	TYR	P14679
MOL000392	XDH	P47989
MOL000398	SOAT1	P35610
MOL000398	ADORA2A	P29274

MOL000398	ADORA2B	P29275
MOL000398	FAAH	O00519
MOL000398	ACE	P12821
MOL000398	ALOX12	P18054
MOL000398	ALOX15	P16050
MOL000398	GUSB	P08236
MOL000398	BDKRB2	P30411
MOL000398	C5AR1	P21730
MOL000398	CA9	Q16790
MOL000398	CA12	O43570
MOL000398	CASP3	P42574
MOL000398	CASP7	P55210
MOL000398	CTSH	P09668
MOL000398	CTSV	O60911
MOL000398	CTSF	Q9UBX1
MOL000398	CTSG	P08311
MOL000398	CTSK	P43235
MOL000398	CTSS	P25774
MOL000398	MAPK8	P45983
MOL000398	MAPK10	P53779
MOL000398	CRHR1	P34998
MOL000398	CREBBP	Q92793
MOL000398	CXCR3	P49682
MOL000398	CDK1	P06493
MOL000398	CCNB3	Q8WWL7
MOL000398	CDK2	P24941
MOL000398	CCNE2	O96020
MOL000398	CCND3	P30281
MOL000398	CDK7	P50613
MOL000398	CYP11B1	P15538
MOL000398	CYP11B2	P19099
MOL000398	CYP19A1	P11511
MOL000398	CYP3A4	P08684
MOL000398	CTSC	P53634
MOL000398	DNMT3A	Q9Y6K1
MOL000398	DRD4	P21917
MOL000398	CDC25A	P30304
MOL000398	ECE1	P42892
MOL000398	EPHX1	P07099
MOL000398	HSD17B3	P37058
MOL000398	ESR1	P03372
MOL000398	ESR2	Q92731
MOL000398	XPO1	O14980
MOL000398	FGFR3	P22607

MOL000398	GABRB3	P28472
MOL000398	GABRA2	P47869
MOL000398	GABRG2	P18507
MOL000398	PSEN2	P49810
MOL000398	PGGT1B	P53609
MOL000398	QPCT	Q16769
MOL000398	GSK3B	P49841
MOL000398	HMOX1	P09601
MOL000398	GCK	P35557
MOL000398	HRH1	P35367
MOL000398	EP300	Q09472
MOL000398	KAT2B	Q92831
MOL000398	MCL1	Q07820
MOL000398	INSR	P06213
MOL000398	CXCR2	P25025
MOL000398	RET	P07949
MOL000398	CSF1R	P07333
MOL000398	MIF	P14174
MOL000398	MST1R	Q04912
MOL000398	MKNK1	Q9BUB5
MOL000398	MC4R	P32245
MOL000398	GRM5	P41594
MOL000398	MAOB	P27338
MOL000398	MGLL	Q99685
MOL000398	CHRM3	P20309
MOL000398	MPO	P05164
MOL000398	SIRT2	Q8IXJ6
MOL000398	MME	P08473
MOL000398	HCRTR1	O43613
MOL000398	P2RX7	Q99572
MOL000398	PDE10A	Q9Y233
MOL000398	PDE4B	Q07343
MOL000398	PDE7A	Q13946
MOL000398	PIK3CA	P42336
MOL000398	PIK3CB	P42338
MOL000398	PARP1	P09874
MOL000398	PGR	P06401
MOL000398	FNTA	P49354
MOL000398	PTPN1	P18031
MOL000398	NQO2	P16083
MOL000398	RGS4	P49798
MOL000398	RPS6KA2	Q15349
MOL000398	AURKA	O14965
MOL000398	PLK3	Q9H4B4

MOL000398	TGFBR1	P36897
MOL000398	TBXAS1	P24557
MOL000398	TLR9	Q9NR96
MOL000398	JAK1	P23458
MOL000398	JAK2	O60674
MOL000398	FLT3	P36888
MOL000398	TYK2	P29597
MOL000398	CACNA1B	Q00975
MOL000398	FNTB	P49356
MOL000398	GABRA6	Q16445
MOL000398	GABRA1	P14867
MOL000398	GABRA3	P34903
MOL000398	CCNH	P51946
MOL000398	PSENEN	Q9NZ42
MOL000398	NCSTN	Q92542
MOL000398	APH1A	Q96BI3
MOL000398	PSEN1	P49768
MOL000398	APH1B	Q8WW43
MOL000398	CCND1	P24385
MOL000398	CDK4	P11802
MOL000398	CCND2	P30279
MOL000398	GABRA5	P31644
MOL000398	CCNB1	P14635
MOL000398	CCNB2	O95067
MOL000398	CCNE1	P24864
MOL000417	PFKFB3	Q16875
MOL000417	ACHE	P22303
MOL000417	ADORA1	P30542
MOL000417	ADORA2A	P29274
MOL000417	ALDH2	P05091
MOL000417	AR	P10275
MOL000417	ALOX12	P18054
MOL000417	ALOX15	P16050
MOL000417	ABCG2	Q9UNQ0
MOL000417	ADRB2	P07550
MOL000417	CALM1	P62158
MOL000417	CA1	P00915
MOL000417	CA2	P00918
MOL000417	CA4	P22748
MOL000417	CA5B	Q9Y2D0
MOL000417	CA7	P43166
MOL000417	CA12	O43570
MOL000417	CBR1	P16152
MOL000417	CDC7	O00311

MOL000417	CDK2	P24941
MOL000417	PDE3A	Q14432
MOL000417	CCNA2	P20248
MOL000417	PTGS1	P23219
MOL000417	CYP19A1	P11511
MOL000417	CYP1B1	Q16678
MOL000417	DPP4	P27487
MOL000417	CDC25B	P30305
MOL000417	DUSP3	P51452
MOL000417	EGFR	P00533
MOL000417	HSD17B1	P14061
MOL000417	HSD17B2	P37059
MOL000417	ESR1	P03372
MOL000417	ESR2	Q92731
MOL000417	ESRRA	P11474
MOL000417	ESRRB	O95718
MOL000417	GSK3B	P49841
MOL000417	HSP90AB1	P08238
MOL000417	IL2	P60568
MOL000417	MIF	P14174
MOL000417	MGAM	O43451
MOL000417	MAPK14	Q16539
MOL000417	MAOA	P21397
MOL000417	MAOB	P27338
MOL000417	PRKACA	P17612
MOL000417	ABCC1	P33527
MOL000417	NOX4	Q9NPH5
MOL000417	NOS2	P35228
MOL000417	SLC6A2	P23975
MOL000417	NCOA2	Q15596
MOL000417	PPARG	P37231
MOL000417	PPARA	Q07869
MOL000417	ABCB1	P08183
MOL000417	PTGS2	P35354
MOL000417	PTPN1	P18031
MOL000417	PIM1	P11309
MOL000417	PTPRS	Q13332
MOL000417	RXRA	P19793
MOL000417	CHEK1	O14757
MOL000417	HTR2A	P28223
MOL000417	HTR2C	P28335
MOL000417	PON1	P27169
MOL000417	STS	P08842
MOL000417	F10	P00742

MOL000417	TBXAS1	P24557
MOL000417	PLAT	P00750
MOL000417	TLR9	Q9NR96
MOL000417	PRSS1	P07477
MOL000417	TYR	P14679
MOL000417	PLAU	P00749
MOL000417	XDH	P47989
MOL000422	PSMD3	O43242
MOL000422	PFKFB3	Q16875
MOL000422	ACHE	P22303
MOL000422	AHSA1	O95433
MOL000422	ADORA1	P30542
MOL000422	ADORA2A	P29274
MOL000422	AKR1A1	P14550
MOL000422	AKR1B10	O60218
MOL000422	AKR1C1	Q04828
MOL000422	AKR1C2	P52895
MOL000422	AKR1C3	P42330
MOL000422	AKR1C4	P17516
MOL000422	AKR1B1	P15121
MOL000422	ALK	Q9UM73
MOL000422	ADRA1B	P35368
MOL000422	AMY1A	P04745
MOL000422	AR	P10275
MOL000422	SLPI	P03973
MOL000422	BAX	Q07812
MOL000422	BCL2	P10415
MOL000422	ALOX12	P18054
MOL000422	ALOX15	P16050
MOL000422	ALOX5	P09917
MOL000422	ARG1	P05089
MOL000422	AHR	P35869
MOL000422	ABCG2	Q9UNQ0
MOL000422	APP	P05067
MOL000422	BACE1	P56817
MOL000422	CALM1	P62158
MOL000422	CAMK2B	Q13554
MOL000422	CA1	P00915
MOL000422	CA2	P00918
MOL000422	CA3	P07451
MOL000422	CA4	P22748
MOL000422	CA9	Q16790
MOL000422	CA5A	P35218
MOL000422	CA6	P23280

MOL000422	CA7	P43166
MOL000422	CA12	O43570
MOL000422	CA13	Q8N1Q1
MOL000422	CA14	Q9ULX7
MOL000422	CSNK2A1	P68400
MOL000422	CASP3	P42574
MOL000422	CDK1	P06493
MOL000422	F7	P08709
MOL000422	CCNB3	Q8WWL7
MOL000422	CDK2	P24941
MOL000422	CDK5R1	Q15078
MOL000422	CDK6	Q00534
MOL000422	PTGS2	P35354
MOL000422	CFTR	P13569
MOL000422	CYP19A1	P11511
MOL000422	CYP1A2	P05177
MOL000422	CYP1B1	Q16678
MOL000422	CYP3A4	P08684
MOL000422	DAPK1	P53355
MOL000422	DPP4	P27487
MOL000422	TOP1	P11387
MOL000422	TOP2A	P11388
MOL000422	MPG	P29372
MOL000422	DRD4	P21917
MOL000422	EGFR	P00533
MOL000422	SELE	P16581
MOL000422	HSD17B1	P14061
MOL000422	HSD17B2	P37059
MOL000422	ESR1	P03372
MOL000422	ESR2	Q92731
MOL000422	ESRRA	P11474
MOL000422	PTK2	Q05397
MOL000422	GRK6	P43250
MOL000422	GABRA1	P14867
MOL000422	GABRA2	P47869
MOL000422	GSTM1	P09488
MOL000422	GSTM2	P28161
MOL000422	GSTP1	P09211
MOL000422	GSK3B	P49841
MOL000422	GLO1	Q04760
MOL000422	GPR35	Q9HC97
MOL000422	HSP90AB1	P08238
MOL000422	HMOX1	P09601
MOL000422	MET	P08581

MOL000422	HAS2	Q92819
MOL000422	IKBKB	O14920
MOL000422	INSR	P06213
MOL000422	IGF1R	P08069
MOL000422	ICAM1	P05362
MOL000422	CXCR1	P25024
MOL000422	MMP1	P03956
MOL000422	PYGL	P06737
MOL000422	CD38	P28907
MOL000422	MMP12	P39900
MOL000422	MMP13	P45452
MOL000422	MMP2	P08253
MOL000422	MMP3	P08254
MOL000422	MMP9	P14780
MOL000422	MAPT	P10636
MOL000422	MAPK8	P45983
MOL000422	MAOA	P21397
MOL000422	PRKACA	P17612
MOL000422	ABCC1	P33527
MOL000422	CHRM1	P11229
MOL000422	CHRM2	P08172
MOL000422	MPO	P05164
MOL000422	NOX4	Q9NPH5
MOL000422	NOS3	P29474
MOL000422	NOS1	P29475
MOL000422	NUAK1	O60285
MOL000422	NCOA2	Q15596
MOL000422	NR1I2	O75469
MOL000422	NR1I3	Q14994
MOL000422	PRXC1A	P00433
MOL000422	PPARG	P37231
MOL000422	ABCB1	P08183
MOL000422	PIK3CG	P48736
MOL000422	PLA2G1B	P04054
MOL000422	PIK3R1	P27986
MOL000422	PARP1	P09874
MOL000422	PGR	P06401
MOL000422	PTGS1	P23219
MOL000422	PKN1	Q16512
MOL000422	AKT1	P31749
MOL000422	PTPRS	Q13332
MOL000422	AURKB	Q96GD4
MOL000422	NEK2	P51955
MOL000422	NEK6	Q9HC98

MOL000422	PIM1	P11309
MOL000422	PLK1	P53350
MOL000422	PPP3CB	P16298
MOL000422	STAT1	P42224
MOL000422	SLC6A2	P23975
MOL000422	SLC2A4	P14672
MOL000422	SLC22A12	Q96S37
MOL000422	TNKS	O95271
MOL000422	TNKS2	Q9H2K2
MOL000422	TERT	O14746
MOL000422	F2	P00734
MOL000422	FOS	P01100
MOL000422	RELA	Q04206
MOL000422	TTR	P02766
MOL000422	PRSS1	P07477
MOL000422	TNF	P01375
MOL000422	DIO1	P49895
MOL000422	TYR	P14679
MOL000422	FLT3	P36888
MOL000422	AXL	P30530
MOL000422	SRC	P12931
MOL000422	SYK	P43405
MOL000422	VCAM1	P19320
MOL000422	KDR	P35968
MOL000422	AVPR2	P30518
MOL000422	XDH	P47989
MOL000422	CDK5	Q00535
MOL000422	CCNB1	P14635
MOL000422	CCNB2	O95067
MOL000433	CDK2	P24941
MOL000433	GSK3B	P49841
MOL000433	F2	P00734
MOL000439	TOP2A	P11388
MOL000442	HSP90AB1	P08238
MOL000442	PTGS2	P35354
MOL000442	RXRA	P19793
MOL000442	PRSS1	P07477
MOL000449	HSD11B1	P28845
MOL000449	HTR2A	P28223
MOL000449	ACHE	P22303
MOL000449	ADH1C	P00326
MOL000449	AKR1B1	P15121
MOL000449	ADRA1A	P35348
MOL000449	ADRA1B	P35368

MOL000449	ADRA2A	P08913
MOL000449	MAOA	P21397
MOL000449	MAOB	P27338
MOL000449	AR	P10275
MOL000449	DHCR7	Q9UBM7
MOL000449	ADRB1	P08588
MOL000449	ADRB2	P07550
MOL000449	BCHE	P06276
MOL000449	CTRB1	P17538
MOL000449	SERPINA6	P08185
MOL000449	CYP17A1	P05093
MOL000449	CYP19A1	P11511
MOL000449	CYP2C19	P33261
MOL000449	CYP51A1	Q16850
MOL000449	POLB	P06746
MOL000449	ESR1	P03372
MOL000449	ESR2	Q92731
MOL000449	GABRA1	P14867
MOL000449	GABRA3	P34903
MOL000449	G6PD	P11413
MOL000449	GLRA1	P23415
MOL000449	HMGCR	P04035
MOL000449	IGHG1	P01857
MOL000449	LTA4H	P09960
MOL000449	NR1H3	Q13133
MOL000449	NR1H2	P55055
MOL000449	NR3C2	P08235
MOL000449	PRKACA	P17612
MOL000449	CHRM1	P11229
MOL000449	CHRM2	P08172
MOL000449	CHRM3	P20309
MOL000449	CHRNA7	P36544
MOL000449	NPC1L1	Q9UHC9
MOL000449	NOS2	P35228
MOL000449	SLC6A2	P23975
MOL000449	NCOA1	Q15788
MOL000449	NCOA2	Q15596
MOL000449	RORA	P35398
MOL000449	RORC	P51449
MOL000449	NR1I3	Q14994
MOL000449	PPARA	Q07869
MOL000449	PPARD	Q03181
MOL000449	PPARG	P37231
MOL000449	PGR	P06401

MOL000449	PTGES	O14684
MOL000449	PTGS1	P23219
MOL000449	PTGS2	P35354
MOL000449	PTGER1	P34995
MOL000449	PTGER2	P43116
MOL000449	PTPN1	P18031
MOL000449	PTPN6	P29350
MOL000449	RXRA	P19793
MOL000449	SLC6A4	P31645
MOL000449	SCN5A	Q14524
MOL000449	SLC6A3	Q01959
MOL000449	SQLE	Q14534
MOL000449	FDFT1	P37268
MOL000449	SREBF2	Q12772
MOL000449	PTPN2	P17706
MOL000449	SHBG	P04278
MOL000449	TBXAS1	P24557
MOL000449	UGT2B7	P16662
MOL000449	PLAU	P00749
MOL000449	VDR	P11473
MOL000456	ADORA2B	P29275
MOL000456	ADRA1B	P35368
MOL000456	ADRA1D	P25100
MOL000456	AR	P10275
MOL000456	BAD	Q92934
MOL000456	ADRB2	P07550
MOL000456	CALM1	P62158
MOL000456	CSNK1A1	P48729
MOL000456	CSNK1D	P48730
MOL000456	CSNK1G1	Q9HCP0
MOL000456	CSNK1G2	P78368
MOL000456	COMT	P21964
MOL000456	CTSS	P25774
MOL000456	CDK2	P24941
MOL000456	PRKG2	Q13237
MOL000456	F10	P00742
MOL000456	CCNA2	P20248
MOL000456	CDK1	P06493
MOL000456	CCNB3	Q8WWL7
MOL000456	CCND3	P30281
MOL000456	CCND1	P24385
MOL000456	CDK5R1	Q15078
MOL000456	CDK7	P50613
MOL000456	LNPEP	Q9UIQ6

MOL000456	DAPK1	P53355
MOL000456	DAPK2	Q9UIK4
MOL000456	DAPK3	O43293
MOL000456	PRKDC	P78527
MOL000456	DSTYK	Q6XUX3
MOL000456	MAP2K1	Q02750
MOL000456	MAP2K2	P36507
MOL000456	MAP2K3	P46734
MOL000456	MAP2K4	P45985
MOL000456	EPHB4	P54760
MOL000456	EPHA2	P29317
MOL000456	EPHB2	P29323
MOL000456	EGFR	P00533
MOL000456	HSD17B2	P37059
MOL000456	HSD17B3	P37058
MOL000456	ESR1	P03372
MOL000456	ESR2	Q92731
MOL000456	FGFR3	P22607
MOL000456	GRK7	Q8WTQ7
MOL000456	GABRB3	P28472
MOL000456	GABRG2	P18507
MOL000456	NR3C1	P04150
MOL000456	GSK3B	P49841
MOL000456	HPGDS	O60760
MOL000456	EP300	Q09472
MOL000456	HDAC1	Q13547
MOL000456	HIPK4	Q8NE63
MOL000456	RET	P07949
MOL000456	LRRK2	Q5S007
MOL000456	LTA4H	P09960
MOL000456	CSF1R	P07333
MOL000456	MIF	P14174
MOL000456	MAPK14	Q16539
MOL000456	MAP3K9	P80192
MOL000456	CHRM1	P11229
MOL000456	CHRM3	P20309
MOL000456	MYLK	Q15746
MOL000456	NTRK1	P04629
MOL000456	CHRNA7	P36544
MOL000456	NOS2	P35228
MOL000456	NCOA2	Q15596
MOL000456	CASK	O14936
MOL000456	PPARG	P37231
MOL000456	PIK3C2G	O75747

MOL000456	PIP5K1C	O60331
MOL000456	PIP4K2C	Q8TBX8
MOL000456	PDE5A	O76074
MOL000456	PDE7A	Q13946
MOL000456	PHKG2	P15735
MOL000456	PIK3CB	P42338
MOL000456	PIK3CD	O00329
MOL000456	PIK3CG	P48736
MOL000456	PI4KB	Q9UBF8
MOL000456	PDGFRA	P16234
MOL000456	PDGFRB	P09619
MOL000456	PTGS1	P23219
MOL000456	PTGS2	P35354
MOL000456	PTPN1	P18031
MOL000456	PIM1	P11309
MOL000456	ERBB2	P04626
MOL000456	RXRA	P19793
MOL000456	RXRB	P28702
MOL000456	RPS6KA1	Q15418
MOL000456	RPS6KA3	P51812
MOL000456	RPS6KA4	O75676
MOL000456	AURKA	O14965
MOL000456	CHEK1	O14757
MOL000456	CHEK2	O96017
MOL000456	MTOR	P42345
MOL000456	PIM2	Q9P1W9
MOL000456	PIM3	Q86V86
MOL000456	PLK1	P53350
MOL000456	TAOK2	Q9UL54
MOL000456	WEE1	P30291
MOL000456	SCN5A	Q14524
MOL000456	S1PR1	P21453
MOL000456	S1PR3	Q99500
MOL000456	VCP	P55072
MOL000456	PRSS1	P07477
MOL000456	TUBB1	Q9H4B7
MOL000456	ABL1	P00519
MOL000456	BLK	P51451
MOL000456	CSK	P41240
MOL000456	FYN	P06241
MOL000456	HCK	P08631
MOL000456	JAK1	P23458
MOL000456	JAK2	O60674
MOL000456	JAK3	P52333

MOL000456	LCK	P06239
MOL000456	FLT3	P36888
MOL000456	YES1	P07947
MOL000456	FLT1	P17948
MOL000456	FLT4	P35916
MOL000456	CDK5	Q00535
MOL000456	CDK4	P11802
MOL000456	CCND2	P30279
MOL000456	GABRA3	P34903
MOL000456	GABRA1	P14867
MOL000456	GABRA5	P31644
MOL000456	GABRA6	Q16445
MOL000456	CCNB1	P14635
MOL000456	CCNB2	O95067
MOL000471	AKR1B1	P15121
MOL000471	BAX	Q07812
MOL000471	PKIA	P61925
MOL000471	CASP3	P42574
MOL000471	CDK1	P06493
MOL000471	TP53	P04637
MOL000471	CDKN1A	P38936
MOL000471	EIF6	P56537
MOL000471	FASN	P49327
MOL000471	CCNB1	P14635
MOL000471	HSP90AB1	P08238
MOL000471	IGHG1	P01857
MOL000471	IL1B	P01584
MOL000471	PRKACA	P17612
MOL000471	MYC	P01106
MOL000471	NCOA2	Q15596
MOL000471	PIK3CG	P48736
MOL000471	PCNA	P12004
MOL000471	PTGS1	P23219
MOL000471	PTGS2	P35354
MOL000471	PRKCA	P17252
MOL000471	PRKCD	Q05655
MOL000471	PRKCE	Q02156
MOL000471	TNF	P01375
MOL000492	DPEP1	P16444
MOL000492	CALM1	P62158
MOL000492	CAT	P04040
MOL000492	ESR1	P03372
MOL000492	HSP90AB1	P08238
MOL000492	HAS2	Q92819

MOL000492	PRKACA	P17612
MOL000492	NCOA2	Q15596
MOL000492	PTGS1	P23219
MOL000492	PTGS2	P35354
MOL000492	RXRA	P19793
MOL000569	AKR1B1	P15121
MOL000569	FUT7	Q11130
MOL000569	CA1	P00915
MOL000569	CA2	P00918
MOL000569	CA3	P07451
MOL000569	CA9	Q16790
MOL000569	CA5A	P35218
MOL000569	CA6	P23280
MOL000569	CA7	P43166
MOL000569	CA12	O43570
MOL000569	CA14	Q9ULX7
MOL000569	CSNK2A1	P68400
MOL000569	CSNK2A2	P19784
MOL000569	POLA1	P09884
MOL000569	POLB	P06746
MOL000569	HSP90AB1	P08238
MOL000569	MAOA	P21397
MOL000569	MAOB	P27338
MOL000569	SERPINE1	P05121
MOL000569	PTGS2	P35354
MOL000569	TUBB1	Q9H4B7
MOL001004	PFKFB3	Q16875
MOL001004	ACHE	P22303
MOL001004	ADORA1	P30542
MOL001004	ADORA2A	P29274
MOL001004	ADORA3	P0DMS8
MOL001004	AKR1A1	P14550
MOL001004	AKR1C1	Q04828
MOL001004	AKR1C2	P52895
MOL001004	AKR1C4	P17516
MOL001004	AKR1C3	P42330
MOL001004	AKR1B1	P15121
MOL001004	ALK	Q9UM73
MOL001004	AR	P10275
MOL001004	ALOX12	P18054
MOL001004	ALOX15	P16050
MOL001004	ALOX5	P09917
MOL001004	ARG1	P05089
MOL001004	AHR	P35869

MOL001004	ABCG2	Q9UNQ0
MOL001004	BACE1	P56817
MOL001004	BCHE	P06276
MOL001004	CAMK2B	Q13554
MOL001004	CA1	P00915
MOL001004	CA2	P00918
MOL001004	CA3	P07451
MOL001004	CA4	P22748
MOL001004	CA9	Q16790
MOL001004	CA5A	P35218
MOL001004	CA6	P23280
MOL001004	CA7	P43166
MOL001004	CA12	O43570
MOL001004	CA13	Q8N1Q1
MOL001004	CA14	Q9ULX7
MOL001004	CSNK2A1	P68400
MOL001004	CDK1	P06493
MOL001004	CCNB3	Q8WWL7
MOL001004	CDK2	P24941
MOL001004	CDK5R1	Q15078
MOL001004	CDK6	Q00534
MOL001004	PTGS1	P23219
MOL001004	CYP19A1	P11511
MOL001004	CYP1B1	Q16678
MOL001004	DAPK1	P53355
MOL001004	TOP2A	P11388
MOL001004	APEX1	P27695
MOL001004	MPG	P29372
MOL001004	DRD4	P21917
MOL001004	EGFR	P00533
MOL001004	HSD17B1	P14061
MOL001004	HSD17B2	P37059
MOL001004	ESR1	P03372
MOL001004	ESR2	Q92731
MOL001004	ESRRA	P11474
MOL001004	PTK2	Q05397
MOL001004	NR3C1	P04150
MOL001004	GSK3B	P49841
MOL001004	GLO1	Q04760
MOL001004	GPR35	Q9HC97
MOL001004	HSP90AB1	P08238
MOL001004	MET	P08581
MOL001004	INSR	P06213
MOL001004	IGF1R	P08069

MOL001004	CXCR1	P25024
MOL001004	PYGL	P06737
MOL001004	NR1H3	Q13133
MOL001004	CD38	P28907
MOL001004	KDM4E	B2RXH2
MOL001004	MMP13	P45452
MOL001004	MMP2	P08253
MOL001004	MMP3	P08254
MOL001004	MMP9	P14780
MOL001004	MAPT	P10636
MOL001004	NR3C2	P08235
MOL001004	MAOA	P21397
MOL001004	PRKACA	P17612
MOL001004	ABCC1	P33527
MOL001004	MPO	P05164
MOL001004	MYLK	Q15746
MOL001004	SIRT1	Q96EB6
MOL001004	NOX4	Q9NPH5
MOL001004	NOS3	P29474
MOL001004	NUAK1	O60285
MOL001004	NCOA1	Q15788
MOL001004	NCOA2	Q15596
MOL001004	PPARG	P37231
MOL001004	ABCB1	P08183
MOL001004	PDE4B	Q07343
MOL001004	PLA2G1B	P04054
MOL001004	PIK3CG	P48736
MOL001004	PIK3R1	P27986
MOL001004	PGR	P06401
MOL001004	PTGS2	P35354
MOL001004	PKN1	Q16512
MOL001004	PTPRS	Q13332
MOL001004	RXRA	P19793
MOL001004	AKT1	P31749
MOL001004	AURKB	Q96GD4
MOL001004	NEK2	P51955
MOL001004	NEK6	Q9HC98
MOL001004	PIM1	P11309
MOL001004	PLK1	P53350
MOL001004	SLC22A12	Q96S37
MOL001004	SHBG	P04278
MOL001004	F2	P00734
MOL001004	TYR	P14679
MOL001004	FLT3	P36888

MOL001004	AXL	P30530
MOL001004	SRC	P12931
MOL001004	SYK	P43405
MOL001004	KDR	P35968
MOL001004	AVPR2	P30518
MOL001004	XDH	P47989
MOL001004	CDK5	Q00535
MOL001004	CCNB1	P14635
MOL001004	CCNB2	O95067
MOL001040	ACHE	P22303
MOL001040	CES1	P23141
MOL001040	ADORA1	P30542
MOL001040	ADORA3	P0DMS8
MOL001040	AKR1C3	P42330
MOL001040	ALK	Q9UM73
MOL001040	ALOX12	P18054
MOL001040	ABCG2	Q9UNQ0
MOL001040	DPEP1	P16444
MOL001040	BACE1	P56817
MOL001040	BCHE	P06276
MOL001040	CA1	P00915
MOL001040	CA2	P00918
MOL001040	CA4	P22748
MOL001040	CA7	P43166
MOL001040	CA12	O43570
MOL001040	CBR1	P16152
MOL001040	CES2	O00748
MOL001040	CTSB	P07858
MOL001040	CTSK	P43235
MOL001040	CTSL	P07711
MOL001040	CISD1	Q9NZ45
MOL001040	PTGS1	P23219
MOL001040	CYP19A1	P11511
MOL001040	CYP1B1	Q16678
MOL001040	POLB	P06746
MOL001040	TOP1	P11387
MOL001040	DNM1	Q05193
MOL001040	EDNRA	P25101
MOL001040	HSD17B1	P14061
MOL001040	HSD17B2	P37059
MOL001040	ESR1	P03372
MOL001040	ESR2	Q92731
MOL001040	NR3C1	P04150
MOL001040	GSK3B	P49841

MOL001040	GRK2	P25098
MOL001040	PLA2G10	O15496
MOL001040	HSP90AB1	P08238
MOL001040	HSP90AA1	P07900
MOL001040	HNF4A	P41235
MOL001040	IGF1R	P08069
MOL001040	KLK1	P06870
MOL001040	KLK2	P20151
MOL001040	KDM1A	O60341
MOL001040	MMP12	P39900
MOL001040	MMP13	P45452
MOL001040	MMP2	P08253
MOL001040	GRM2	Q14416
MOL001040	GRM5	P41594
MOL001040	NR3C2	P08235
MOL001040	MAOA	P21397
MOL001040	MAOB	P27338
MOL001040	PRKACA	P17612
MOL001040	ABCC1	P33527
MOL001040	CHRNA7	P36544
MOL001040	PPARG	P37231
MOL001040	PLA2G1B	P04054
MOL001040	PLA2G2A	P14555
MOL001040	PLA2G5	P39877
MOL001040	PGF	P49763
MOL001040	SERPINE1	P05121
MOL001040	PDGFRA	P16234
MOL001040	PDGFRB	P09619
MOL001040	PGR	P06401
MOL001040	PTGS2	P35354
MOL001040	PTGER1	P34995
MOL001040	PTGER2	P43116
MOL001040	PTGER3	P43115
MOL001040	RXRA	P19793
MOL001040	RPS6KA5	O75582
MOL001040	BRAF	P15056
MOL001040	CHEK1	O14757
MOL001040	WEE1	P30291
MOL001040	ERN1	O75460
MOL001040	SLC5A2	P31639
MOL001040	TAS2R31	P59538
MOL001040	SHBG	P04278
MOL001040	SRC	P12931
MOL001040	TDP1	Q9NUW8

MOL001040	VEGFA	P15692
MOL001040	KDR	P35968
MOL001040	FLT4	P35916
MOL001474	HPGD	P15428
MOL001474	ACHE	P22303
MOL001474	SCD	O00767
MOL001474	BAD	Q92934
MOL001474	CA9	Q16790
MOL001474	CA12	O43570
MOL001474	CA14	Q9ULX7
MOL001474	CYP11B1	P15538
MOL001474	CYP11B2	P19099
MOL001474	CYP17A1	P05093
MOL001474	DHFR	P00374
MOL001474	HDAC1	Q13547
MOL001474	HDAC6	Q9UBN7
MOL001474	PRKACA	P17612
MOL001474	MME	P08473
MOL001474	PDE5A	O76074
MOL001474	PTGS1	P23219
MOL001474	PTGS2	P35354
MOL001474	PRKCE	Q02156
MOL001474	RXRA	P19793
MOL001474	CHEK2	O96017
MOL001474	PLK1	P53350
MOL001474	SCN9A	Q15858
MOL001474	TBXAS1	P24557
MOL001474	TDP2	O95551
MOL001484	HTR3A,	P46098
MOL001484	ADAM17	P78536
MOL001484	ADCY5	O95622
MOL001484	ADRA1B	P35368
MOL001484	ADRA1D	P25100
MOL001484	AR	P10275
MOL001484	ALOX15	P16050
MOL001484	ADRB2	P07550
MOL001484	CALM1	P62158
MOL001484	F3	P13726
MOL001484	LNPEP	Q9UIQ6
MOL001484	DAO	P14920
MOL001484	DYRK1B	Q9Y463
MOL001484	CLK1	P49759
MOL001484	HSD17B2	P37059
MOL001484	HSD17B3	P37058

MOL001484	ESRRA	P11474
MOL001484	ESRRB	O95718
MOL001484	EZR	P15311
MOL001484	HSP90AB1	P08238
MOL001484	IGHG1	P01857
MOL001484	IMPDH1	P20839
MOL001484	IMPDH2	P12268
MOL001484	RET	P07949
MOL001484	MIF	P14174
MOL001484	MAPKAPK2	P49137
MOL001484	PRKACA	P17612
MOL001484	CHRM1	P11229
MOL001484	CHRM3	P20309
MOL001484	OPRM1	P35372
MOL001484	PIK3CG	P48736
MOL001484	PGF	P49763
MOL001484	PARP1	P09874
MOL001484	PTGS1	P23219
MOL001484	PTGS2	P35354
MOL001484	PTPN1	P18031
MOL001484	RXRA	P19793
MOL001484	RPS6KA1	Q15418
MOL001484	RPS6KA3	P51812
MOL001484	CHEK1	O14757
MOL001484	CHEK2	O96017
MOL001484	TBK1	Q9UHD2
MOL001484	WEE1	P30291
MOL001484	ERN1	O75460
MOL001484	SCN5A	Q14524
MOL001484	TRPM8	Q7Z2W7
MOL001484	PRSS1	P07477
MOL001484	TUBB1	Q9H4B7
MOL001484	TUBB3	Q13509
MOL001484	LCK	P06239
MOL001484	SRC	P12931
MOL001484	VEGFA	P15692
MOL001601	HTR3A,	P46098
MOL001601	ACHE	P22303
MOL001601	CES1	P23141
MOL001601	ADORA3	P0DMS8
MOL001601	AKR1B1	P15121
MOL001601	ADRA1A	P35348
MOL001601	ADRA1B	P35368
MOL001601	CNR1	P21554

MOL001601	CA2	P00918
MOL001601	CES2	O00748
MOL001601	CASP3	P42574
MOL001601	CASP7	P55210
MOL001601	PDE3A	Q14432
MOL001601	MAPK8	P45983
MOL001601	OPRD1	P41143
MOL001601	TOP1	P11387
MOL001601	DUSP1	P28562
MOL001601	EGFR	P00533
MOL001601	GABRB3	P28472
MOL001601	GABRA2	P47869
MOL001601	IDO1	P14902
MOL001601	IDH1	O75874
MOL001601	PTPRC	P08575
MOL001601	KDM4E	B2RXH2
MOL001601	GRM1	Q13255
MOL001601	MALT1	Q9UDY8
MOL001601	CHRM1	P11229
MOL001601	CHRM2	P08172
MOL001601	CHRM3	P20309
MOL001601	CHRM4	P08173
MOL001601	CHRM5	P08912
MOL001601	PIK3CA	P42336
MOL001601	PTGS1	P23219
MOL001601	PTGS2	P35354
MOL001601	PBRM1	Q86U86
MOL001601	PTPN6	P29350
MOL001601	PTPN11	Q06124
MOL001601	ERBB2	P04626
MOL001601	RXRA	P19793
MOL001601	STAT3	P40763
MOL001601	SCN5A	Q14524
MOL001601	SMARCA4	P51532
MOL001601	TDP2	O95551
MOL001601	KDR	P35968
MOL001601	GABRA3	P34903
MOL001601	GABRG2	P18507
MOL001601	GABRA1	P14867
MOL001601	GABRA5	P31644
MOL001735	PFKFB3	Q16875
MOL001735	ACHE	P22303
MOL001735	ADORA1	P30542
MOL001735	ADORA2A	P29274

MOL001735	ADORA3	P0DMS8
MOL001735	AKR1B10	O60218
MOL001735	AKR1B1	P15121
MOL001735	ALK	Q9UM73
MOL001735	AMY1A	P04745
MOL001735	AR	P10275
MOL001735	ALOX12	P18054
MOL001735	ALOX15	P16050
MOL001735	ALOX5	P09917
MOL001735	ARG1	P05089
MOL001735	AHR	P35869
MOL001735	ABCG2	Q9UNQ0
MOL001735	APP	P05067
MOL001735	ST6GAL1	P15907
MOL001735	BACE1	P56817
MOL001735	CALM1	P62158
MOL001735	CA1	P00915
MOL001735	CA2	P00918
MOL001735	CA4	P22748
MOL001735	CA9	Q16790
MOL001735	CA7	P43166
MOL001735	CA12	O43570
MOL001735	CBR1	P16152
MOL001735	CSNK2A1	P68400
MOL001735	CDK1	P06493
MOL001735	CCNB3	Q8WWL7
MOL001735	CDK5R1	Q15078
MOL001735	CDK6	Q00534
MOL001735	PTGS2	P35354
MOL001735	CFTR	P13569
MOL001735	CYP19A1	P11511
MOL001735	CYP1A2	P05177
MOL001735	CYP1B1	Q16678
MOL001735	DAPK1	P53355
MOL001735	OPRD1	P41143
MOL001735	DPP4	P27487
MOL001735	TOP1	P11387
MOL001735	MPG	P29372
MOL001735	DRD2	P14416
MOL001735	EGFR	P00533
MOL001735	HSD17B1	P14061
MOL001735	HSD17B2	P37059
MOL001735	ESR1	P03372
MOL001735	ESR2	Q92731

MOL001735	ESRRA	P11474
MOL001735	PTK2	Q05397
MOL001735	GRK6	P43250
MOL001735	GSK3B	P49841
MOL001735	GLO1	Q04760
MOL001735	GPR35	Q9HC97
MOL001735	HSP90AB1	P08238
MOL001735	MET	P08581
MOL001735	IGHG1	P01857
MOL001735	MCL1	Q07820
MOL001735	IGF1R	P08069
MOL001735	CD38	P28907
MOL001735	KDM4E	B2RXH2
MOL001735	MMP12	P39900
MOL001735	MMP2	P08253
MOL001735	MMP9	P14780
MOL001735	MAOA	P21397
MOL001735	MAOB	P27338
MOL001735	PRKACA	P17612
MOL001735	OPRM1	P35372
MOL001735	ABCC1	P33527
MOL001735	NOX4	Q9NPH5
MOL001735	NAE1	Q13564
MOL001735	NOS2	P35228
MOL001735	NOS1	P29475
MOL001735	NCOA1	Q15788
MOL001735	NCOA2	Q15596
MOL001735	ODC1	P11926
MOL001735	ABCB1	P08183
MOL001735	PIK3CG	P48736
MOL001735	PLA2G2A	P14555
MOL001735	PLG	P00747
MOL001735	PARP1	P09874
MOL001735	PTGS1	P23219
MOL001735	PKN1	Q16512
MOL001735	PTPN1	P18031
MOL001735	PTPRS	Q13332
MOL001735	RHO	P08100
MOL001735	AKT1	P31749
MOL001735	AURKB	Q96GD4
MOL001735	NEK2	P51955
MOL001735	NEK6	Q9HC98
MOL001735	PIM1	P11309
MOL001735	PLK1	P53350

MOL001735	SIGMAR1	Q99720
MOL001735	SLC22A12	Q96S37
MOL001735	KIT	P10721
MOL001735	TNKS	O95271
MOL001735	TNKS2	Q9H2K2
MOL001735	TERT	O14746
MOL001735	F2	P00734
MOL001735	TTR	P02766
MOL001735	PRSS1	P07477
MOL001735	TYR	P14679
MOL001735	LCK	P06239
MOL001735	FLT3	P36888
MOL001735	AXL	P30530
MOL001735	SRC	P12931
MOL001735	SYK	P43405
MOL001735	KDR	P35968
MOL001735	XDH	P47989
MOL001735	CDK5	Q00535
MOL001735	CCNB1	P14635
MOL001735	CCNB2	O95067
MOL001771	HSD11B1	P28845
MOL001771	ACHE	P22303
MOL001771	AR	P10275
MOL001771	DHCR7	Q9UBM7
MOL001771	BCHE	P06276
MOL001771	CES2	O00748
MOL001771	SERPINA6	P08185
MOL001771	CYP17A1	P05093
MOL001771	CYP19A1	P11511
MOL001771	CYP2C19	P33261
MOL001771	CYP51A1	Q16850
MOL001771	POLB	P06746
MOL001771	ESR1	P03372
MOL001771	ESR2	Q92731
MOL001771	G6PD	P11413
MOL001771	GLRA1	P23415
MOL001771	HMGCR	P04035
MOL001771	NR1H3	Q13133
MOL001771	NR1H2	P55055
MOL001771	CHRM2	P08172
MOL001771	NPC1L1	Q9UHC9
MOL001771	NOS2	P35228
MOL001771	SLC6A2	P23975
MOL001771	NCOA2	Q15596

MOL001771	RORA	P35398
MOL001771	RORC	P51449
MOL001771	NR1I3	Q14994
MOL001771	PPARD	Q03181
MOL001771	PPARG	P37231
MOL001771	PGR	P06401
MOL001771	PTGES	O14684
MOL001771	PTGER1	P34995
MOL001771	PTGER2	P43116
MOL001771	PTPN1	P18031
MOL001771	PTPN6	P29350
MOL001771	SLC6A4	P31645
MOL001771	SQLE	Q14534
MOL001771	SREBF2	Q12772
MOL001771	PTPN2	P17706
MOL001771	SHBG	P04278
MOL001771	UGT2B7	P16662
MOL001771	VDR	P11473
MOL001918	GABRA1	P14867
MOL001919	AR	P10275
MOL001919	CYP19A1	P11511
MOL001919	ESR1	P03372
MOL001919	GABBR1	Q9UBS5
MOL001919	NR3C1	P04150
MOL001919	HAO1	Q9UJM8
MOL001919	NR3C2	P08235
MOL001919	CHRM1	P11229
MOL001919	PGR	P06401
MOL001919	SHBG	P04278
MOL001921	PFKFB3	Q16875
MOL001921	HSPA5	P11021
MOL001921	FGF1	P05230
MOL001921	ADORA2A	P29274
MOL001921	ADORA2B	P29275
MOL001921	ADORA3	P0DMS8
MOL001921	ADK	P55263
MOL001921	FGF2	P09038
MOL001921	GBA	P04062
MOL001921	F3	P13726
MOL001921	SLC6A3	Q01959
MOL001921	MAP2K1	Q02750
MOL001921	ECE1	P42892
MOL001921	EGFR	P00533
MOL001921	SLC29A1	Q99808

MOL001921	LGALS3	P17931
MOL001921	LGALS9	O00182
MOL001921	GLRA1	P23415
MOL001921	GLRA2	P23416
MOL001921	HSPA8	P11142
MOL001921	HSP90AA1	P07900
MOL001921	HPSE	Q9Y251
MOL001921	HK1	P19367
MOL001921	HK2	P52789
MOL001921	MME	P08473
MOL001921	LGALS7	P47929
MOL001921	ABCB1	P08183
MOL001921	PTAFR	P25105
MOL001921	PPM1A	P35813
MOL001921	SLC5A11	Q8WWX8
MOL001921	SLC28A2	O43868
MOL001921	SSTR1	P30872
MOL001921	SSTR2	P30874
MOL001921	SSTR3	P32745
MOL001921	SSTR4	P31391
MOL001921	SSTR5	P35346
MOL001921	FDFT1	P37268
MOL001921	VEGFA	P15692
MOL001924	FGF1	P05230
MOL001924	AKR1B1	P15121
MOL001924	AMY1A	P04745
MOL001924	FGF2	P09038
MOL001924	BACE1	P56817
MOL001924	LGALS3	P17931
MOL001924	LGALS9	O00182
MOL001924	HSP90AA1	P07900
MOL001924	HPSE	Q9Y251
MOL001924	IL6	P05231
MOL001924	LBP	P18428
MOL001924	CD14	P08571
MOL001924	SLC6A2	P23975
MOL001924	ABCB1	P08183
MOL001924	SERPINE1	P05121
MOL001924	PTAFR	P25105
MOL001924	PTPN1	P18031
MOL001924	SELP	P16109
MOL001924	SSTR1	P30872
MOL001924	SSTR2	P30874
MOL001924	SSTR3	P32745

MOL001924	SSTR4	P31391
MOL001924	SSTR5	P35346
MOL001924	SQLE	Q14534
MOL001924	PTPN2	P17706
MOL001924	F10	P00742
MOL001924	TNF	P01375
MOL001924	VEGFA	P15692
MOL001942	HSD11B1	P28845
MOL001942	ADORA2B	P29275
MOL001942	AKR1C1	Q04828
MOL001942	AKR1C3	P42330
MOL001942	ALOX15	P16050
MOL001942	ALOX5	P09917
MOL001942	BACE1	P56817
MOL001942	BRD4	O60885
MOL001942	CA1	P00915
MOL001942	CA4	P22748
MOL001942	CA9	Q16790
MOL001942	CA5B	Q9Y2D0
MOL001942	CA6	P23280
MOL001942	CA7	P43166
MOL001942	CA12	O43570
MOL001942	CA13	Q8N1Q1
MOL001942	CA14	Q9ULX7
MOL001942	CBR1	P16152
MOL001942	CCR9	P51686
MOL001942	CDK1	P06493
MOL001942	DNMT3A	Q9Y6K1
MOL001942	HPGDS	O60760
MOL001942	HMGCR	P04035
MOL001942	OPRK1	P41145
MOL001942	LIMK1	P53667
MOL001942	CD38	P28907
MOL001942	MTNR1A	P48039
MOL001942	METAP2	P50579
MOL001942	MAOA	P21397
MOL001942	MAOB	P27338
MOL001942	P2RX7	Q99572
MOL001942	PDE10A	Q9Y233
MOL001942	PDE7A	Q13946
MOL001942	PDE8B	O95263
MOL001942	PIK3CA	P42336
MOL001942	PIK3CB	P42338
MOL001942	PIK3CD	O00329

MOL001942	PIK3CG	P48736
MOL001942	PARP2	Q9UGN5
MOL001942	PARP1	P09874
MOL001942	PTGES	O14684
MOL001942	PTGS2	P35354
MOL001942	MTOR	P42345
MOL001942	ATM	Q13315
MOL001942	SRD5A1	P18405
MOL001942	JAK2	O60674
MOL001942	FLT3	P36888
MOL001942	KCNA3	P22001
MOL001942	KCNA5	P22460
MOL001942	CCNB1	P14635
MOL002222	HSD11B1	P28845
MOL002222	ACHE	P22303
MOL002222	ADRA1A	P35348
MOL002222	ADRA1B	P35368
MOL002222	ADRA1D	P25100
MOL002222	AR	P10275
MOL002222	ALOX15	P16050
MOL002222	ALOX5	P09917
MOL002222	ADRB2	P07550
MOL002222	CASR	P41180
MOL002222	CA2	P00918
MOL002222	CA12	O43570
MOL002222	CCR1	P32246
MOL002222	CDK4	P11802
MOL002222	PTGS2	P35354
MOL002222	CYP19A1	P11511
MOL002222	CYP24A1	Q07973
MOL002222	CYP2D6	P10635
MOL002222	DRD2	P14416
MOL002222	OPRD1	P41143
MOL002222	POLA1	P09884
MOL002222	ELAVL1	Q15717
MOL002222	HSD17B1	P14061
MOL002222	HSD17B2	P37059
MOL002222	ESR1	P03372
MOL002222	ESR2	Q92731
MOL002222	GABRB3	P28472
MOL002222	GABRA2	P47869
MOL002222	NR3C1	P04150
MOL002222	GPBAR1	Q8TDU6
MOL002222	HNF4A	P41235

MOL002222	KAT2B	Q92831
MOL002222	HDAC2	Q92769
MOL002222	LDLR	P01130
MOL002222	LRRK2	Q5S007
MOL002222	NR1H3	Q13133
MOL002222	NR1H2	P55055
MOL002222	MC4R	P32245
MOL002222	NR3C2	P08235
MOL002222	MAOA	P21397
MOL002222	CHRM1	P11229
MOL002222	CHRM2	P08172
MOL002222	CHRM3	P20309
MOL002222	CHRM4	P08173
MOL002222	CHRM5	P08912
MOL002222	NOX4	Q9NPH5
MOL002222	NOS2	P35228
MOL002222	PLA2G2A	P14555
MOL002222	PLA2G5	P39877
MOL002222	PRKCZ	Q05513
MOL002222	QRFPR	Q96P65
MOL002222	HTR2B	P41595
MOL002222	SLC6A4	P31645
MOL002222	SCN5A	Q14524
MOL002222	CYP27A1	Q02318
MOL002222	STS	P08842
MOL002222	SAE1	Q9UBE0
MOL002222	SHBG	P04278
MOL002222	TBXA2R	P21731
MOL002222	TSPO	P30536
MOL002222	SRC	P12931
MOL002222	KDR	P35968
MOL002222	GABRA3	P34903
MOL002222	GABRG2	P18507
MOL002222	GABRA1	P14867
MOL002222	GABRA5	P31644
MOL002235	PFKFB3	Q16875
MOL002235	ACHE	P22303
MOL002235	ADORA1	P30542
MOL002235	ADORA2A	P29274
MOL002235	ADORA3	P0DMS8
MOL002235	AKR1A1	P14550
MOL002235	AKR1B10	O60218
MOL002235	AKR1C1	Q04828
MOL002235	AKR1C2	P52895

MOL002235	AKR1C4	P17516
MOL002235	AKR1C3	P42330
MOL002235	AKR1B1	P15121
MOL002235	ALK	Q9UM73
MOL002235	AR	P10275
MOL002235	ALOX12	P18054
MOL002235	ALOX15	P16050
MOL002235	ALOX5	P09917
MOL002235	ARG1	P05089
MOL002235	AHR	P35869
MOL002235	ABCG2	Q9UNQ0
MOL002235	APP	P05067
MOL002235	ST6GAL1	P15907
MOL002235	BACE1	P56817
MOL002235	CALM1	P62158
MOL002235	CAMK2B	Q13554
MOL002235	CA1	P00915
MOL002235	CA2	P00918
MOL002235	CA3	P07451
MOL002235	CA4	P22748
MOL002235	CA9	Q16790
MOL002235	CA5A	P35218
MOL002235	CA6	P23280
MOL002235	CA7	P43166
MOL002235	CA12	O43570
MOL002235	CA13	Q8N1Q1
MOL002235	CA14	Q9ULX7
MOL002235	CSNK2A1	P68400
MOL002235	F7	P08709
MOL002235	F10	P00742
MOL002235	CDK1	P06493
MOL002235	CDK2	P24941
MOL002235	CDK6	Q00534
MOL002235	CYP19A1	P11511
MOL002235	CYP1B1	Q16678
MOL002235	DAPK1	P53355
MOL002235	OPRD1	P41143
MOL002235	DPP4	P27487
MOL002235	TOP1	P11387
MOL002235	TOP2A	P11388
MOL002235	APEX1	P27695
MOL002235	MPG	P29372
MOL002235	DRD4	P21917
MOL002235	EGFR	P00533

MOL002235	HSD17B1	P14061
MOL002235	HSD17B2	P37059
MOL002235	ESR2	Q92731
MOL002235	ESRRA	P11474
MOL002235	PTK2	Q05397
MOL002235	GSK3B	P49841
MOL002235	GLO1	Q04760
MOL002235	GPR35	Q9HC97
MOL002235	HSP90AB1	P08238
MOL002235	MET	P08581
MOL002235	INSR	P06213
MOL002235	IGF1R	P08069
MOL002235	CXCR1	P25024
MOL002235	PYGL	P06737
MOL002235	CD38	P28907
MOL002235	KDM4E	B2RXH2
MOL002235	MMP13	P45452
MOL002235	MMP2	P08253
MOL002235	MMP3	P08254
MOL002235	MMP9	P14780
MOL002235	MAPT	P10636
MOL002235	MAOA	P21397
MOL002235	ABCC1	P33527
MOL002235	MPO	P05164
MOL002235	MYLK	Q15746
MOL002235	NOX4	Q9NPH5
MOL002235	NOS3	P29474
MOL002235	NUAK1	O60285
MOL002235	NCOA2	Q15596
MOL002235	ODC1	P11926
MOL002235	PPARD	Q03181
MOL002235	ABCB1	P08183
MOL002235	PLA2G1B	P04054
MOL002235	PLA2G2A	P14555
MOL002235	PIK3CG	P48736
MOL002235	PIK3R1	P27986
MOL002235	PLG	P00747
MOL002235	PTGS2	P35354
MOL002235	PKN1	Q16512
MOL002235	PTPRS	Q13332
MOL002235	AKT1	P31749
MOL002235	AURKB	Q96GD4
MOL002235	NEK2	P51955
MOL002235	NEK6	Q9HC98

MOL002235	PIM1	P11309
MOL002235	PLK1	P53350
MOL002235	SCN5A	Q14524
MOL002235	SLC22A12	Q96S37
MOL002235	TNKS	O95271
MOL002235	TNKS2	Q9H2K2
MOL002235	F2	P00734
MOL002235	PRSS1	P07477
MOL002235	TYR	P14679
MOL002235	FLT3	P36888
MOL002235	AXL	P30530
MOL002235	SRC	P12931
MOL002235	SYK	P43405
MOL002235	KDR	P35968
MOL002235	AVPR2	P30518
MOL002235	XDH	P47989
MOL002251	ALOX5	P09917
MOL002259	CHIA	Q9BZP6
MOL002259	ADORA2A	P29274
MOL002259	ADORA2B	P29275
MOL002259	ADORA3	P0DMS8
MOL002259	FUCA1	P04066
MOL002259	CA14	Q9ULX7
MOL002259	TOP2A	P11388
MOL002259	ECE1	P42892
MOL002259	EPHA2	P29317
MOL002259	EPHX2	P34913
MOL002259	SLC29A1	Q99808
MOL002259	ESR1	P03372
MOL002259	LGALS3	P17931
MOL002259	LGALS4	P56470
MOL002259	LGALS8	O00214
MOL002259	LGALS9	O00182
MOL002259	IMPDH1	P20839
MOL002259	SLC5A4	Q9NY91
MOL002259	GAA	P10253
MOL002259	MMP1	P03956
MOL002259	MMP12	P39900
MOL002259	MMP13	P45452
MOL002259	MMP7	P09237
MOL002259	MMP8	P22894
MOL002259	MAP3K9	P80192
MOL002259	MME	P08473
MOL002259	SLC5A1	P13866

MOL002259	SLC5A2	P31639
MOL002259	HRAS	P01112
MOL002259	TNNC1	P63316
MOL002259	ABL1	P00519
MOL002259	BTK	Q06187
MOL002259	TNNT2	P45379
MOL002259	TNNI3	P19429
MOL002260	BCL2	P10415
MOL002260	APP	P05067
MOL002260	PTGS1	P23219
MOL002260	DYRK1A	Q13627
MOL002260	MAPK14	Q16539
MOL002260	MAPT	P10636
MOL002268	ADA	P00813
MOL002268	AKR1B1	P15121
MOL002268	FTO	Q9C0B1
MOL002268	AMPD3	Q01432
MOL002268	BCL2	P10415
MOL002268	ACLY	P53396
MOL002268	OGA	O60502
MOL002268	CAMKK2	Q96RR4
MOL002268	CSNK2A1	P68400
MOL002268	CASP1	P29466
MOL002268	CASP2	P42575
MOL002268	CASP3	P42574
MOL002268	CASP6	P55212
MOL002268	CASP7	P55210
MOL002268	CASP8	Q14790
MOL002268	MAPK8	P45983
MOL002268	CDK2	P24941
MOL002268	CYP19A1	P11511
MOL002268	CDC25B	P30305
MOL002268	EGLN1	Q9GZT9
MOL002268	ECE1	P42892
MOL002268	ESR1	P03372
MOL002268	ESR2	Q92731
MOL002268	GRK6	P43250
MOL002268	GPR35	Q9HC97
MOL002268	HSP90AB1	P08238
MOL002268	HNF4A	P41235
MOL002268	MCL1	Q07820
MOL002268	INSR	P06213
MOL002268	IGFBP3	P17936
MOL002268	ELANE	P08246

MOL002268	LIMK1	P53667
MOL002268	LDHA	P00338
MOL002268	LDHB	P07195
MOL002268	MMP1	P03956
MOL002268	MMP13	P45452
MOL002268	MMP14	P50281
MOL002268	MMP16	P51512
MOL002268	MMP2	P08253
MOL002268	MMP8	P22894
MOL002268	MMP9	P14780
MOL002268	NOX4	Q9NPH5
MOL002268	MME	P08473
MOL002268	NCOA2	Q15596
MOL002268	PIK3CG	P48736
MOL002268	PTGS1	P23219
MOL002268	PTGS2	P35354
MOL002268	FNTA	P49354
MOL002268	PTP4A3	O75365
MOL002268	PIM1	P11309
MOL002268	ERN1	O75460
MOL002268	SLC13A5	Q86YT5
MOL002268	F2	P00734
MOL002268	FOS	P01100
MOL002268	LCK	P06239
MOL002268	FNTB	P49356
MOL002280	TOP2A	P11388
MOL002281	PDPK1	O15530
MOL002281	AKR1B1	P15121
MOL002281	GUSB	P08236
MOL002281	CA1	P00915
MOL002281	CA9	Q16790
MOL002281	CA12	O43570
MOL002281	PTGS2	P35354
MOL002281	LNPEP	Q9UIQ6
MOL002281	DYRK1B	Q9Y463
MOL002281	EGLN1	Q9GZT9
MOL002281	ESR1	P03372
MOL002281	ESR2	Q92731
MOL002281	FADS1	O60427
MOL002281	HSP90AB1	P08238
MOL002281	RET	P07949
MOL002281	MPEG1	Q2M385
MOL002281	PRKACA	P17612
MOL002281	NOS2	P35228

MOL002281	ABCB1	P08183
MOL002281	PIK3CG	P48736
MOL002281	PDE5A	O76074
MOL002281	SERPINE1	P05121
MOL002281	PTGS1	P23219
MOL002281	PTK2B	Q14289
MOL002281	RPS6KA3	P51812
MOL002281	CHEK1	O14757
MOL002281	ILK	Q13418
MOL002281	TYMS	P04818
MOL002281	TUBB1	Q9H4B7
MOL002281	TUBB3	Q13509
MOL002288	TOP2A	P11388
MOL002288	ESR1	P03372
MOL002288	SLC5A4	Q9NY91
MOL002288	SLC5A1	P13866
MOL002288	SLC5A2	P31639
MOL002293	ESR1	P03372
MOL002293	TNNC1	P63316
MOL002293	TNNT2	P45379
MOL002293	TNNI3	P19429
MOL002297	HSD11B2	P80365
MOL002297	PFKFB3	Q16875
MOL002297	BCL2L1	Q07817
MOL002297	DRD4	P21917
MOL002297	CDC25B	P30305
MOL002297	PSEN2	P49810
MOL002297	IL2	P60568
MOL002297	ACP1	P24666
MOL002297	NCOA2	Q15596
MOL002297	RBP4	P02753
MOL002297	PTAFR	P25105
MOL002297	PGR	P06401
MOL002297	PPM1B	O75688
MOL002297	PTPN1	P18031
MOL002297	PPP2R5A	Q15172
MOL002297	PPP2CA	P67775
MOL002297	PPP1CC	P36873
MOL002297	STAT3	P40763
MOL002297	S1PR1	P21453
MOL002297	S1PR3	Q99500
MOL002297	PTPN2	P17706
MOL002297	PSENEN	Q9NZ42
MOL002297	NCSTN	Q92542

MOL002297	APH1A	Q96BI3
MOL002297	PSEN1	P49768
MOL002297	APH1B	Q8WW43
MOL002303	AKR1B1	P15121
MOL002303	FTO	Q9C0B1
MOL002303	BCL2	P10415
MOL002303	ABCG2	Q9UNQ0
MOL002303	CTNNB1	P35222
MOL002303	BACE1	P56817
MOL002303	BCHE	P06276
MOL002303	CNR2	P34972
MOL002303	CSNK2A1	P68400
MOL002303	CCR5	P51681
MOL002303	PTGS1	P23219
MOL002303	PTGS2	P35354
MOL002303	CYP19A1	P11511
MOL002303	HSP90B1	P14625
MOL002303	ESR1	P03372
MOL002303	ESR2	Q92731
MOL002303	FBP1	P09467
MOL002303	HSP90AA1	P07900
MOL002303	HNF4A	P41235
MOL002303	MCL1	Q07820
MOL002303	IDH1	O75874
MOL002303	KLK1	P06870
MOL002303	KLK2	P20151
MOL002303	LDHA	P00338
MOL002303	LDHB	P07195
MOL002303	NOS2	P35228
MOL002303	RELA	Q04206
MOL002303	ABCB1	P08183
MOL002303	PDE5A	O76074
MOL002303	PTP4A3	O75365
MOL002303	PDK1	Q15118
MOL002303	PIM1	P11309
MOL002303	ERN1	O75460
MOL002303	SRC	P12931
MOL002514	PFKFB3	Q16875
MOL002514	ACHE	P22303
MOL002514	ADORA1	P30542
MOL002514	ADORA2A	P29274
MOL002514	ADORA3	P0DMS8
MOL002514	AKR1A1	P14550
MOL002514	AKR1C1	Q04828

MOL002514	AKR1C2	P52895
MOL002514	AKR1C4	P17516
MOL002514	AKR1C3	P42330
MOL002514	AKR1B1	P15121
MOL002514	ALK	Q9UM73
MOL002514	AMY1A	P04745
MOL002514	AR	P10275
MOL002514	ALOX12	P18054
MOL002514	ALOX15	P16050
MOL002514	ALOX5	P09917
MOL002514	AHR	P35869
MOL002514	ABCG2	Q9UNQ0
MOL002514	APP	P05067
MOL002514	BACE1	P56817
MOL002514	CAMK2B	Q13554
MOL002514	CA1	P00915
MOL002514	CA2	P00918
MOL002514	CA3	P07451
MOL002514	CA4	P22748
MOL002514	CA9	Q16790
MOL002514	CA5A	P35218
MOL002514	CA6	P23280
MOL002514	CA7	P43166
MOL002514	CA12	O43570
MOL002514	CA13	Q8N1Q1
MOL002514	CA14	Q9ULX7
MOL002514	CSNK2A1	P68400
MOL002514	CDK2	P24941
MOL002514	CDK1	P06493
MOL002514	CDK5R1	Q15078
MOL002514	PTGS2	P35354
MOL002514	CYP19A1	P11511
MOL002514	CYP1A2	P05177
MOL002514	CYP1B1	Q16678
MOL002514	DAPK1	P53355
MOL002514	OPRD1	P41143
MOL002514	DPP4	P27487
MOL002514	MPG	P29372
MOL002514	DRD4	P21917
MOL002514	EGFR	P00533
MOL002514	HSD17B1	P14061
MOL002514	HSD17B2	P37059
MOL002514	ESR1	P03372
MOL002514	ESR2	Q92731

MOL002514	ESRRA	P11474
MOL002514	PTK2	Q05397
MOL002514	GRK6	P43250
MOL002514	GSK3B	P49841
MOL002514	GLO1	Q04760
MOL002514	GPR35	Q9HC97
MOL002514	HSP90AB1	P08238
MOL002514	MET	P08581
MOL002514	MCL1	Q07820
MOL002514	IGF1R	P08069
MOL002514	CXCR1	P25024
MOL002514	PYGL	P06737
MOL002514	KDM4E	B2RXH2
MOL002514	MMP13	P45452
MOL002514	MMP2	P08253
MOL002514	MMP3	P08254
MOL002514	MMP9	P14780
MOL002514	MAPT	P10636
MOL002514	MAOA	P21397
MOL002514	OPRM1	P35372
MOL002514	ABCC1	P33527
MOL002514	MPO	P05164
MOL002514	NOX4	Q9NPH5
MOL002514	NAE1	Q13564
MOL002514	NOS3	P29474
MOL002514	NOS2	P35228
MOL002514	NUAK1	O60285
MOL002514	ODC1	P11926
MOL002514	ABCB1	P08183
MOL002514	PIK3CG	P48736
MOL002514	PLA2G1B	P04054
MOL002514	PIK3R1	P27986
MOL002514	PLG	P00747
MOL002514	PTGS1	P23219
MOL002514	PKN1	Q16512
MOL002514	PTPRS	Q13332
MOL002514	AKT1	P31749
MOL002514	AURKB	Q96GD4
MOL002514	NEK2	P51955
MOL002514	NEK6	Q9HC98
MOL002514	PIM1	P11309
MOL002514	PLK1	P53350
MOL002514	SLC22A12	Q96S37
MOL002514	KIT	P10721

MOL002514	TERT	O14746
MOL002514	F2	P00734
MOL002514	PRSS1	P07477
MOL002514	TYR	P14679
MOL002514	FLT3	P36888
MOL002514	AXL	P30530
MOL002514	SRC	P12931
MOL002514	SYK	P43405
MOL002514	KDR	P35968
MOL002514	AVPR2	P30518
MOL002514	XDH	P47989
MOL002514	CDK5	Q00535
MOL002714	PFKFB3	Q16875
MOL002714	ACHE	P22303
MOL002714	ADORA1	P30542
MOL002714	ADORA2A	P29274
MOL002714	ADORA3	P0DMS8
MOL002714	AKR1B10	O60218
MOL002714	AKR1B1	P15121
MOL002714	ALK	Q9UM73
MOL002714	AMY1A	P04745
MOL002714	AR	P10275
MOL002714	APOD	P05090
MOL002714	BAX	Q07812
MOL002714	BCL2	P10415
MOL002714	ALOX12	P18054
MOL002714	ALOX15	P16050
MOL002714	ALOX5	P09917
MOL002714	ARG1	P05089
MOL002714	AHR	P35869
MOL002714	ABCG2	Q9UNQ0
MOL002714	APP	P05067
MOL002714	ST6GAL1	P15907
MOL002714	BACE1	P56817
MOL002714	BCHE	P06276
MOL002714	CALM1	P62158
MOL002714	CA1	P00915
MOL002714	CA2	P00918
MOL002714	CA4	P22748
MOL002714	CA9	Q16790
MOL002714	CA6	P23280
MOL002714	CA7	P43166
MOL002714	CA12	O43570
MOL002714	CBR1	P16152

MOL002714	CSNK2A1	P68400
MOL002714	CASP3	P42574
MOL002714	CDK1	P06493
MOL002714	TP53	P04637
MOL002714	PDE3A	Q14432
MOL002714	CCNB3	Q8WWL7
MOL002714	CDK2	P24941
MOL002714	CDK5R1	Q15078
MOL002714	CDK6	Q00534
MOL002714	PTGS2	P35354
MOL002714	CFTR	P13569
MOL002714	UQCRB	P14927
MOL002714	CYP19A1	P11511
MOL002714	CYP1B1	Q16678
MOL002714	PLA2G4A	P47712
MOL002714	OPRD1	P41143
MOL002714	DPP4	P27487
MOL002714	TOP1	P11387
MOL002714	TOP2A	P11388
MOL002714	APEX1	P27695
MOL002714	PRKDC	P78527
MOL002714	EGLN1	Q9GZT9
MOL002714	EGFR	P00533
MOL002714	HSD17B1	P14061
MOL002714	HSD17B2	P37059
MOL002714	ESR1	P03372
MOL002714	ESR2	Q92731
MOL002714	ESRRA	P11474
MOL002714	FABP5	Q01469
MOL002714	FOSL1	P15407
MOL002714	FOSL2	P15408
MOL002714	GRK6	P43250
MOL002714	CCNB1	P14635
MOL002714	GSK3B	P49841
MOL002714	GLO1	Q04760
MOL002714	GPR35	Q9HC97
MOL002714	HSP90AB1	P08238
MOL002714	MET	P08581
MOL002714	HIF1A	Q16665
MOL002714	IKBKB	O14920
MOL002714	INSR	P06213
MOL002714	IGF1R	P08069
MOL002714	IGF2	P01344
MOL002714	CD38	P28907

MOL002714	KDM4E	B2RXH2
MOL002714	MAPK3	P27361
MOL002714	MMP12	P39900
MOL002714	MMP2	P08253
MOL002714	MMP9	P14780
MOL002714	MAPT	P10636
MOL002714	MAOA	P21397
MOL002714	PRKACA	P17612
MOL002714	ABCC1	P33527
MOL002714	MPO	P05164
MOL002714	MYLK	Q15746
MOL002714	SIRT1	Q96EB6
MOL002714	NOX4	Q9NPH5
MOL002714	NOX5	Q96PH1
MOL002714	NAE1	Q13564
MOL002714	TACR2	P21452
MOL002714	NTRK2	Q16620
MOL002714	NOS2	P35228
MOL002714	NFATC1	O95644
MOL002714	NCOA1	Q15788
MOL002714	NCOA2	Q15596
MOL002714	ABCB1	P08183
MOL002714	PIK3CG	P48736
MOL002714	PLA2G2A	P14555
MOL002714	PARP1	P09874
MOL002714	PTGS1	P23219
MOL002714	PTPN1	P18031
MOL002714	FOS	P01100
MOL002714	AKT1	P31749
MOL002714	PTPRS	Q13332
MOL002714	AURKB	Q96GD4
MOL002714	PIM1	P11309
MOL002714	PLK1	P53350
MOL002714	SLC22A12	Q96S37
MOL002714	KIT	P10721
MOL002714	TNKS	O95271
MOL002714	TNKS2	Q9H2K2
MOL002714	TERT	O14746
MOL002714	RELA	Q04206
MOL002714	TTR	P02766
MOL002714	PRSS1	P07477
MOL002714	TDRD7	Q8NHU6
MOL002714	TYR	P14679
MOL002714	FYN	P06241

MOL002714	LCK	P06239
MOL002714	FLT3	P36888
MOL002714	AXL	P30530
MOL002714	SRC	P12931
MOL002714	SYK	P43405
MOL002714	VEGFA	P15692
MOL002714	KDR	P35968
MOL002714	XDH	P47989
MOL002714	CDK5	Q00535
MOL002714	CCNB2	O95067
MOL002776	ACHE	P22303
MOL002776	ADORA1	P30542
MOL002776	ADRA2C	P18825
MOL002776	ALDH2	P05091
MOL002776	AKR1B1	P15121
MOL002776	ADRA2A	P08913
MOL002776	F10	P00742
MOL002776	EGFR	P00533
MOL002776	IL2	P60568
MOL002776	PTPN1	P18031
MOL002776	NOX4	Q9NPH5
MOL002776	NMUR2	Q9GZQ4
MOL002776	NQO2	P16083
MOL002776	RPS6KA3	P51812
MOL002776	TNF	P01375
MOL002776	XDH	P47989
MOL003347	HSD11B2	P80365
MOL003347	ALOX5	P09917
MOL003347	CCKBR	P32239
MOL003347	PTGS2	P35354
MOL003347	CYP19A1	P11511
MOL003347	CYP3A4	P08684
MOL003347	ICAM1	P05362
MOL003347	CXCL8	P10145
MOL003347	ITGAL	P20701
MOL003347	NR3C2	P08235
MOL003347	NOS2	P35228
MOL003347	NR1I2	O75469
MOL003347	PTGES	O14684
MOL003542	PFKFB3	Q16875
MOL003542	ADORA3	P0DMS8
MOL003542	AKR1C1	Q04828
MOL003542	AKR1C2	P52895
MOL003542	AKR1C4	P17516

MOL003542	AKR1C3	P42330
MOL003542	AKR1B1	P15121
MOL003542	ALK	Q9UM73
MOL003542	ALPL	P05186
MOL003542	AR	P10275
MOL003542	ALOX12	P18054
MOL003542	ALOX15	P16050
MOL003542	ARG1	P05089
MOL003542	AHR	P35869
MOL003542	ABCG2	Q9UNQ0
MOL003542	BACE1	P56817
MOL003542	KCNMA1	Q12791
MOL003542	CALM1	P62158
MOL003542	CA2	P00918
MOL003542	CA3	P07451
MOL003542	CA4	P22748
MOL003542	CA5A	P35218
MOL003542	CA6	P23280
MOL003542	CA7	P43166
MOL003542	CA12	O43570
MOL003542	CA13	Q8N1Q1
MOL003542	CSNK2A1	P68400
MOL003542	CCR4	P51679
MOL003542	CDK2	P24941
MOL003542	PDE3A	Q14432
MOL003542	F7	P08709
MOL003542	F10	P00742
MOL003542	CCNA2	P20248
MOL003542	CDK1	P06493
MOL003542	CCND1	P24385
MOL003542	CFTR	P13569
MOL003542	CYP19A1	P11511
MOL003542	CYP1A2	P05177
MOL003542	CYP1B1	Q16678
MOL003542	DAPK1	P53355
MOL003542	DHFR	P00374
MOL003542	DPP4	P27487
MOL003542	TOP2A	P11388
MOL003542	APEX1	P27695
MOL003542	MPG	P29372
MOL003542	EGLN1	Q9GZT9
MOL003542	HSP90B1	P14625
MOL003542	EGFR	P00533
MOL003542	HSD17B1	P14061

MOL003542	HSD17B2	P37059
MOL003542	ESR1	P03372
MOL003542	ESR2	Q92731
MOL003542	ESRRA	P11474
MOL003542	GABRA1	P14867
MOL003542	GABRA2	P47869
MOL003542	GCGR	P47871
MOL003542	PYGM	P11217
MOL003542	GSK3B	P49841
MOL003542	HSP90AB1	P08238
MOL003542	HSP90AA1	P07900
MOL003542	EP300	Q09472
MOL003542	MCL1	Q07820
MOL003542	CXCR1	P25024
MOL003542	PYGL	P06737
MOL003542	KDM4E	B2RXH2
MOL003542	MMP2	P08253
MOL003542	MMP3	P08254
MOL003542	MMP9	P14780
MOL003542	MAPT	P10636
MOL003542	MAPK14	Q16539
MOL003542	MAOA	P21397
MOL003542	ABCC1	P33527
MOL003542	MPO	P05164
MOL003542	MYLK	Q15746
MOL003542	NOX4	Q9NPH5
MOL003542	NAE1	Q13564
MOL003542	NCOA2	Q15596
MOL003542	PPARG	P37231
MOL003542	ABCB1	P08183
MOL003542	PDE10A	Q9Y233
MOL003542	PDE5A	O76074
MOL003542	PLA2G1B	P04054
MOL003542	PIK3R1	P27986
MOL003542	KCNH2	Q12809
MOL003542	PTGS1	P23219
MOL003542	PTGS2	P35354
MOL003542	FNTA	P49354
MOL003542	PTPN1	P18031
MOL003542	PIM1	P11309
MOL003542	PDK1	Q15118
MOL003542	ERBB2	P04626
MOL003542	PTPRS	Q13332
MOL003542	RXRA	P19793

MOL003542	AKT1	P31749
MOL003542	CHEK1	O14757
MOL003542	NEK2	P51955
MOL003542	NEK6	Q9HC98
MOL003542	PLK1	P53350
MOL003542	SCN5A	Q14524
MOL003542	F2	P00734
MOL003542	PRSS1	P07477
MOL003542	TYR	P14679
MOL003542	FLT3	P36888
MOL003542	SYK	P43405
MOL003542	KDR	P35968
MOL003542	AVPR2	P30518
MOL003542	XDH	P47989
MOL003542	CDK4	P11802
MOL003542	FNTB	P49356
MOL003627	ACHE	P22303
MOL003627	ADRA2B	P18089
MOL003627	AR	P10275
MOL003627	BCHE	P06276
MOL003627	CYP2C19	P33261
MOL003627	CYP2C9	P11712
MOL003627	CYP2D6	P10635
MOL003627	DRD3	P35462
MOL003627	DRD4	P21917
MOL003627	SLC6A3	Q01959
MOL003627	KCNH2	Q12809
MOL003627	IL6	P05231
MOL003627	KISS1R	Q969F8
MOL003627	CHRM1	P11229
MOL003627	CHRM3	P20309
MOL003627	CHRM4	P08173
MOL003627	CHRM5	P08912
MOL003627	CHRNA4	P43681
MOL003627	SLC6A2	P23975
MOL003627	PARP1	P09874
MOL003627	PRMT3	O60678
MOL003627	HTR1A	P08908
MOL003627	HTR1B	P28222
MOL003627	HTR1D	P28221
MOL003627	HTR3A	P46098
MOL003627	SRD5A1	P18405
MOL003627	SRD5A2	P31213
MOL003627	TNF	P01375

MOL003627	CHRNB2	P17787
MOL003648	HTR3A,	P46098
MOL003648	ADAM17	P78536
MOL003648	ADCY5	O95622
MOL003648	ADRA1B	P35368
MOL003648	ADRA1D	P25100
MOL003648	AR	P10275
MOL003648	ALOX15	P16050
MOL003648	ADRB2	P07550
MOL003648	CALM1	P62158
MOL003648	F3	P13726
MOL003648	LNPEP	Q9UIQ6
MOL003648	DAO	P14920
MOL003648	DYRK1B	Q9Y463
MOL003648	CLK1	P49759
MOL003648	HSD17B2	P37059
MOL003648	HSD17B3	P37058
MOL003648	ESRRA	P11474
MOL003648	ESRRB	O95718
MOL003648	EZR	P15311
MOL003648	HSP90AB1	P08238
MOL003648	IGHG1	P01857
MOL003648	IMPDH1	P20839
MOL003648	IMPDH2	P12268
MOL003648	RET	P07949
MOL003648	MIF	P14174
MOL003648	MAPKAPK2	P49137
MOL003648	PRKACA	P17612
MOL003648	CHRM3	P20309
MOL003648	CHRNA7	P36544
MOL003648	NCOA1	Q15788
MOL003648	PIK3CG	P48736
MOL003648	PGF	P49763
MOL003648	PARP1	P09874
MOL003648	PTGS1	P23219
MOL003648	PTGS2	P35354
MOL003648	PTPN1	P18031
MOL003648	RXRA	P19793
MOL003648	RPS6KA1	Q15418
MOL003648	RPS6KA3	P51812
MOL003648	CHEK1	O14757
MOL003648	CHEK2	O96017
MOL003648	TBK1	Q9UHD2
MOL003648	WEE1	P30291

MOL003648	ERN1	O75460
MOL003648	SCN5A	Q14524
MOL003648	SLC6A4	P31645
MOL003648	TRPM8	Q7Z2W7
MOL003648	PRSS1	P07477
MOL003648	TUBB1	Q9H4B7
MOL003648	TUBB3	Q13509
MOL003648	LCK	P06239
MOL003648	SRC	P12931
MOL003648	VEGFA	P15692
MOL003673	PFKFB3	Q16875
MOL003673	ACHE	P22303
MOL003673	ADORA1	P30542
MOL003673	ADORA2A	P29274
MOL003673	ALDH2	P05091
MOL003673	AR	P10275
MOL003673	ALOX12	P18054
MOL003673	ALOX15	P16050
MOL003673	ABCG2	Q9UNQ0
MOL003673	CALM1	P62158
MOL003673	CA4	P22748
MOL003673	CA7	P43166
MOL003673	CA12	O43570
MOL003673	CCR4	P51679
MOL003673	CDK2	P24941
MOL003673	F10	P00742
MOL003673	CCNA2	P20248
MOL003673	CCND1	P24385
MOL003673	CYP19A1	P11511
MOL003673	DPP4	P27487
MOL003673	TOP2A	P11388
MOL003673	EGLN1	Q9GZT9
MOL003673	HSP90B1	P14625
MOL003673	EGFR	P00533
MOL003673	HSD17B1	P14061
MOL003673	HSD17B2	P37059
MOL003673	ESR1	P03372
MOL003673	ESR2	Q92731
MOL003673	ESRRA	P11474
MOL003673	ESRRB	O95718
MOL003673	GSK3B	P49841
MOL003673	HSP90AA1	P07900
MOL003673	HSP90AB1	P08238
MOL003673	IL2	P60568

MOL003673	MIF	P14174
MOL003673	MGAM	O43451
MOL003673	MAPK14	Q16539
MOL003673	MAOA	P21397
MOL003673	SIRT1	Q96EB6
MOL003673	NOX4	Q9NPH5
MOL003673	NOS2	P35228
MOL003673	SLC6A2	P23975
MOL003673	RELA	Q04206
MOL003673	PPARG	P37231
MOL003673	ABCB1	P08183
MOL003673	PDE5A	O76074
MOL003673	PTGS2	P35354
MOL003673	FNTA	P49354
MOL003673	PTPN1	P18031
MOL003673	PIM1	P11309
MOL003673	AKT1	P31749
MOL003673	CHEK1	O14757
MOL003673	HTR2A	P28223
MOL003673	HTR2C	P28335
MOL003673	SCN5A	Q14524
MOL003673	F2	P00734
MOL003673	TBXAS1	P24557
MOL003673	PRSS1	P07477
MOL003673	TYR	P14679
MOL003673	XDH	P47989
MOL003673	FNTB	P49356
MOL003673	CDK4	P11802
MOL003676	CHRNB1	P11230
MOL003676	ADRA2B	P18089
MOL003676	CYP2D6	P10635
MOL003676	DRD2	P14416
MOL003676	DRD3	P35462
MOL003676	KISS1R	Q969F8
MOL003676	SLC47A1	Q96FL8
MOL003676	CHRNA4	P43681
MOL003676	CHRNA7	P36544
MOL003676	CHRNA3	P32297
MOL003676	PARP1	P09874
MOL003676	PRMT3	O60678
MOL003676	HTR2B	P41595
MOL003676	HTR3B	O95264
MOL003676	HTR3A	P46098
MOL003676	HTR7	P34969

MOL003676	SLC22A2	O15244
MOL003676	SRD5A2	P31213
MOL003676	CHRNB2	P17787
MOL003676	CHRNB4	P30926
MOL003676	CHRNA1	P02708
MOL003676	CHRNG	P07510
MOL003676	CHRND	Q07001
MOL003680	HSD11B1	P28845
MOL003680	HSD17B7	P56937
MOL003680	ADH1A	P07327
MOL003680	ADH1C	P00326
MOL003680	RNPEP	Q9H4A4
MOL003680	FAAH	O00519
MOL003680	DNPEP	Q9ULA0
MOL003680	BCHE	P06276
MOL003680	CTSB	P07858
MOL003680	CTSK	P43235
MOL003680	CTSL	P07711
MOL003680	DPP7	Q9UHL4
MOL003680	DPP4	P27487
MOL003680	DPP9	Q86TI2
MOL003680	DPP8	Q6V1X1
MOL003680	DRD1	P21728
MOL003680	SLC6A3	Q01959
MOL003680	DNM1	Q05193
MOL003680	EPHX2	P34913
MOL003680	EPHX1	P07099
MOL003680	HDAC10	Q969S8
MOL003680	HDAC11	Q96DB2
MOL003680	HDAC2	Q92769
MOL003680	HDAC3	O15379
MOL003680	NCOR2	Q9Y618
MOL003680	HDAC6	Q9UBN7
MOL003680	HDAC8	Q9BY41
MOL003680	ITGA2B	P08514
MOL003680	IL6	P05231
MOL003680	MMP8	P22894
MOL003680	GRM5	P41594
MOL003680	METAP1	P53582
MOL003680	SLC47A1	Q96FL8
MOL003680	CHRM1	P11229
MOL003680	CHRM2	P08172
MOL003680	CHRM3	P20309
MOL003680	CHRM4	P08173

MOL003680	CHRM5	P08912
MOL003680	NAAA	Q02083
MOL003680	MME	P08473
MOL003680	CHRNA4	P43681
MOL003680	CHRNA7	P36544
MOL003680	CHRNA3	P32297
MOL003680	PARP1	P09874
MOL003680	PAOX	Q6QHF9
MOL003680	PRMT3	O60678
MOL003680	HTR1B	P28222
MOL003680	HTR1D	P28221
MOL003680	HTR3B	O95264
MOL003680	HTR3A	P46098
MOL003680	SIGMAR1	Q99720
MOL003680	SLC22A2	O15244
MOL003680	SRD5A1	P18405
MOL003680	SRD5A2	P31213
MOL003680	TNF	P01375
MOL003680	CHRNB2	P17787
MOL003680	CHRNB4	P30926
MOL003680	ITGB3	P05106
MOL003758	ACHE	P22303
MOL003758	AR	P10275
MOL003758	CALM1	P62158
MOL003758	CDK2	P24941
MOL003758	F7	P08709
MOL003758	F10	P00742
MOL003758	DPP4	P27487
MOL003758	TOP2A	P11388
MOL003758	ESR1	P03372
MOL003758	ESR2	Q92731
MOL003758	GSK3B	P49841
MOL003758	HSP90AB1	P08238
MOL003758	MAPK14	Q16539
MOL003758	NOS3	P29474
MOL003758	NCOA2	Q15596
MOL003758	PPARG	P37231
MOL003758	PTGS2	P35354
MOL003758	PIM1	P11309
MOL003758	CHEK1	O14757
MOL003758	SCN5A	Q14524
MOL003758	PRSS1	P07477
MOL003856	AKR1B1	P15121
MOL003856	AR	P10275

MOL003856	ARG1	P05089
MOL003856	ABCG2	Q9UNQ0
MOL003856	CDK5R1	Q15078
MOL003856	PTGS1	P23219
MOL003856	OPRD1	P41143
MOL003856	ESR1	P03372
MOL003856	ESR2	Q92731
MOL003856	EIF2AK3	Q9NZJ5
MOL003856	GLO1	Q04760
MOL003856	HSP90AB1	P08238
MOL003856	NR1H3	Q13133
MOL003856	CD38	P28907
MOL003856	MAOA	P21397
MOL003856	PRKACA	P17612
MOL003856	ABCC1	P33527
MOL003856	SIRT1	Q96EB6
MOL003856	NOX4	Q9NPH5
MOL003856	NCOA1	Q15788
MOL003856	PDE4B	Q07343
MOL003856	PDE4D	Q08499
MOL003856	PLA2G2A	P14555
MOL003856	PLG	P00747
MOL003856	PTPRS	Q13332
MOL003856	TNKS	O95271
MOL003856	TNKS2	Q9H2K2
MOL003856	TERT	O14746
MOL003856	SHBG	P04278
MOL003856	TTR	P02766
MOL003856	PRSS1	P07477
MOL003856	XDH	P47989
MOL003856	CDK5	Q00535
MOL003857	SLC33A1	O00400
MOL003857	ADRA2A	P08913
MOL003857	FAAH	O00519
MOL003857	BACE2	Q9Y5Z0
MOL003857	CCNA2	P20248
MOL003857	PTGS1	P23219
MOL003857	CYP17A1	P05093
MOL003857	DUT	P33316
MOL003857	ESR1	P03372
MOL003857	ESR2	Q92731
MOL003857	FGFR1	P11362
MOL003857	HSP90AB1	P08238
MOL003857	KCNH2	Q12809

MOL003857	HDAC11	Q96DB2
MOL003857	HDAC4	P56524
MOL003857	HDAC5	Q9UQL6
MOL003857	HDAC7	Q8WUI4
MOL003857	HDAC9	Q9UKV0
MOL003857	IMPDH2	P12268
MOL003857	IRAK4	Q9NWZ3
MOL003857	CSF1R	P07333
MOL003857	MMP1	P03956
MOL003857	MMP2	P08253
MOL003857	MMP7	P09237
MOL003857	MMP8	P22894
MOL003857	NR3C2	P08235
MOL003857	PRKACA	P17612
MOL003857	CHRM1	P11229
MOL003857	CHRM2	P08172
MOL003857	CHRM3	P20309
MOL003857	SLC6A2	P23975
MOL003857	PPARG	P37231
MOL003857	PDE10A	Q9Y233
MOL003857	PDE4B	Q07343
MOL003857	PDE4D	Q08499
MOL003857	PIK3CA	P42336
MOL003857	PGR	P06401
MOL003857	PTPN1	P18031
MOL003857	ROCK2	O75116
MOL003857	RPS6KB1	P23443
MOL003857	AKT1	P31749
MOL003857	AURKA	O14965
MOL003857	CHEK1	O14757
MOL003857	WEE1	P30291
MOL003857	HTR6	P50406
MOL003857	HTR7	P34969
MOL003857	SCN9A	Q15858
MOL003857	PRSS1	P07477
MOL003857	AGTR1	P30556
MOL003857	ABL1	P00519
MOL003857	JAK1	P23458
MOL003857	JAK3	P52333
MOL003857	FLT1	P17948
MOL003858	HPGD	P15428
MOL003858	ADAM17	P78536
MOL003858	ADRA2A	P08913
MOL003858	AR	P10275

MOL003858	BIRC5	O15392
MOL003858	CDK2	P24941
MOL003858	ESR1	P03372
MOL003858	ESR2	Q92731
MOL003858	NR3C1	P04150
MOL003858	GSK3B	P49841
MOL003858	HSP90AB1	P08238
MOL003858	LTB4R	Q15722
MOL003858	CSF1R	P07333
MOL003858	MMP1	P03956
MOL003858	MMP7	P09237
MOL003858	GRM5	P41594
MOL003858	NR3C2	P08235
MOL003858	MAPK14	Q16539
MOL003858	PRKACA	P17612
MOL003858	NOS2	P35228
MOL003858	NCOA1	Q15788
MOL003858	PPARG	P37231
MOL003858	PIK3CG	P48736
MOL003858	PDE10A	Q9Y233
MOL003858	PIK3CA	P42336
MOL003858	PGR	P06401
MOL003858	PTGS2	P35354
MOL003858	ROCK2	O75116
MOL003858	BRAF	P15056
MOL003858	MTOR	P42345
MOL003858	SLC5A1	P13866
MOL003858	PRSS1	P07477
MOL003858	AGTR1	P30556
MOL003860	ADORA3	P0DMS8
MOL003860	AKR1B10	O60218
MOL003860	AKR1B1	P15121
MOL003860	ALOX5	P09917
MOL003860	ARG1	P05089
MOL003860	APP	P05067
MOL003860	CDK5R1	Q15078
MOL003860	PTGS1	P23219
MOL003860	ESR1	P03372
MOL003860	ESR2	Q92731
MOL003860	GSK3B	P49841
MOL003860	GLO1	Q04760
MOL003860	HSP90AB1	P08238
MOL003860	NR1H3	Q13133
MOL003860	CD38	P28907

MOL003860	PRKACA	P17612
MOL003860	ABCC1	P33527
MOL003860	SIRT1	Q96EB6
MOL003860	ABCB1	P08183
MOL003860	PDE4B	Q07343
MOL003860	PDE4D	Q08499
MOL003860	PIK3CA	P42336
MOL003860	PTPRS	Q13332
MOL003860	PIM1	P11309
MOL003860	TNKS	O95271
MOL003860	TNKS2	Q9H2K2
MOL003860	SHBG	P04278
MOL003860	TTR	P02766
MOL003860	CDK5	Q00535
MOL004004	PFKFB3	Q16875
MOL004004	ACHE	P22303
MOL004004	ADORA1	P30542
MOL004004	ADORA2A	P29274
MOL004004	AKR1A1	P14550
MOL004004	AKR1B10	O60218
MOL004004	AKR1C1	Q04828
MOL004004	AKR1C2	P52895
MOL004004	AKR1C4	P17516
MOL004004	AKR1C3	P42330
MOL004004	AKR1B1	P15121
MOL004004	ALK	Q9UM73
MOL004004	AMY1A	P04745
MOL004004	AR	P10275
MOL004004	ALOX12	P18054
MOL004004	ALOX15	P16050
MOL004004	ALOX5	P09917
MOL004004	ARG1	P05089
MOL004004	ABCG2	Q9UNQ0
MOL004004	APP	P05067
MOL004004	BACE1	P56817
MOL004004	CAMK2B	Q13554
MOL004004	CA1	P00915
MOL004004	CA2	P00918
MOL004004	CA3	P07451
MOL004004	CA4	P22748
MOL004004	CA9	Q16790
MOL004004	CA5A	P35218
MOL004004	CA6	P23280
MOL004004	CA7	P43166

MOL004004	CA12	O43570
MOL004004	CA13	Q8N1Q1
MOL004004	CA14	Q9ULX7
MOL004004	CSNK2A1	P68400
MOL004004	CDK1	P06493
MOL004004	CCNB3	Q8WWL7
MOL004004	CDK5R1	Q15078
MOL004004	CDK6	Q00534
MOL004004	PTGS2	P35354
MOL004004	CFTR	P13569
MOL004004	CYP19A1	P11511
MOL004004	CYP1B1	Q16678
MOL004004	DAPK1	P53355
MOL004004	TOP1	P11387
MOL004004	TOP2A	P11388
MOL004004	APEX1	P27695
MOL004004	DRD4	P21917
MOL004004	EGFR	P00533
MOL004004	HSD17B1	P14061
MOL004004	HSD17B2	P37059
MOL004004	ESR1	P03372
MOL004004	ESR2	Q92731
MOL004004	PTK2	Q05397
MOL004004	GRK6	P43250
MOL004004	GSK3B	P49841
MOL004004	GLO1	Q04760
MOL004004	GPR35	Q9HC97
MOL004004	HSP90AB1	P08238
MOL004004	MET	P08581
MOL004004	INSR	P06213
MOL004004	IGF1R	P08069
MOL004004	CXCR1	P25024
MOL004004	PYGL	P06737
MOL004004	CD38	P28907
MOL004004	KDM4E	B2RXH2
MOL004004	MMP12	P39900
MOL004004	MMP13	P45452
MOL004004	MMP2	P08253
MOL004004	MMP3	P08254
MOL004004	MMP9	P14780
MOL004004	MAPT	P10636
MOL004004	MAOA	P21397
MOL004004	ABCC1	P33527
MOL004004	MPO	P05164

MOL004004	MYLK	Q15746
MOL004004	NOX4	Q9NPH5
MOL004004	NUAK1	O60285
MOL004004	NCOA2	Q15596
MOL004004	ABCB1	P08183
MOL004004	PIK3CG	P48736
MOL004004	PLA2G1B	P04054
MOL004004	PIK3R1	P27986
MOL004004	PARP1	P09874
MOL004004	PTGS1	P23219
MOL004004	PKN1	Q16512
MOL004004	PTPRS	Q13332
MOL004004	AKT1	P31749
MOL004004	AURKB	Q96GD4
MOL004004	NEK2	P51955
MOL004004	NEK6	Q9HC98
MOL004004	PIM1	P11309
MOL004004	PLK1	P53350
MOL004004	TNKS	O95271
MOL004004	TNKS2	Q9H2K2
MOL004004	TERT	O14746
MOL004004	F2	P00734
MOL004004	TTR	P02766
MOL004004	FLT3	P36888
MOL004004	AXL	P30530
MOL004004	SRC	P12931
MOL004004	SYK	P43405
MOL004004	KDR	P35968
MOL004004	AVPR2	P30518
MOL004004	XDH	P47989
MOL004004	CDK5	Q00535
MOL004004	CCNB1	P14635
MOL004004	CCNB2	O95067
MOL004580	AKR1B1	P15121
MOL004580	HSP90AB1	P08238
MOL004580	PIK3CG	P48736
MOL004580	PTGS1	P23219
MOL004580	PTGS2	P35354
MOL004580	RXRA	P19793
MOL004912	ACHE	P22303
MOL004912	ADRB2	P07550
MOL004912	ALDH2	P05091
MOL004912	AR	P10275
MOL004912	ADRB1	P08588

MOL004912	CALM1	P62158
MOL004912	CA7	P43166
MOL004912	CDK2	P24941
MOL004912	F10	P00742
MOL004912	CCNA2	P20248
MOL004912	DPP4	P27487
MOL004912	ESR1	P03372
MOL004912	ESR2	Q92731
MOL004912	GCGR	P47871
MOL004912	GSK3B	P49841
MOL004912	HSP90AA1	P07900
MOL004912	HSP90AB1	P08238
MOL004912	MAPK14	Q16539
MOL004912	NOS3	P29474
MOL004912	PPARG	P37231
MOL004912	PDE10A	Q9Y233
MOL004912	PDE4D	Q08499
MOL004912	PTGS1	P23219
MOL004912	PTGS2	P35354
MOL004912	PTPN1	P18031
MOL004912	PIM1	P11309
MOL004912	RXRA	P19793
MOL004912	CHEK1	O14757
MOL004912	SCN5A	Q14524
MOL004912	F2	P00734
MOL004912	PRSS1	P07477
MOL004941	MAOB	P27338
MOL004941	ADRB2	P07550
MOL004941	DPEP1	P16444
MOL004941	CALM1	P62158
MOL004941	PKIA	P61925
MOL004941	PDE3A	Q14432
MOL004941	ESR1	P03372
MOL004941	GABRA1	P14867
MOL004941	HSP90AB1	P08238
MOL004941	PRKACA	P17612
MOL004941	PIK3CG	P48736
MOL004941	PTGS1	P23219
MOL004941	PTGS2	P35354
MOL004941	RXRA	P19793
MOL004941	SLC6A4	P31645
MOL005043	ACHE	P22303
MOL005043	AR	P10275
MOL005043	SERPINA6	P08185

MOL005043	CYP17A1	P05093
MOL005043	CYP19A1	P11511
MOL005043	CYP2C19	P33261
MOL005043	CYP51A1	Q16850
MOL005043	ESR1	P03372
MOL005043	ESR2	Q92731
MOL005043	G6PD	P11413
MOL005043	HMGCR	P04035
MOL005043	NR1H3	Q13133
MOL005043	CHRM2	P08172
MOL005043	NPC1L1	Q9UHC9
MOL005043	RORA	P35398
MOL005043	RORC	P51449
MOL005043	PGR	P06401
MOL005043	PTPN1	P18031
MOL005043	SREBF2	Q12772
MOL005043	SHBG	P04278
MOL005100	PGD	P52209
MOL005100	ACHE	P22303
MOL005100	CES1	P23141
MOL005100	ADORA1	P30542
MOL005100	ADORA3	P0DMS8
MOL005100	ADCY5	O95622
MOL005100	AKR1C3	P42330
MOL005100	AKR1B1	P15121
MOL005100	FUT7	Q11130
MOL005100	BCL2	P10415
MOL005100	NAT1	P18440
MOL005100	ABCG2	Q9UNQ0
MOL005100	APP	P05067
MOL005100	GUSB	P08236
MOL005100	BACE1	P56817
MOL005100	KCNMA1	Q12791
MOL005100	CALM1	P62158
MOL005100	CA1	P00915
MOL005100	CA2	P00918
MOL005100	CA3	P07451
MOL005100	CA4	P22748
MOL005100	CA9	Q16790
MOL005100	CA5A	P35218
MOL005100	CA6	P23280
MOL005100	CA7	P43166
MOL005100	CA12	O43570
MOL005100	CA13	Q8N1Q1

MOL005100	CBR1	P16152
MOL005100	CES2	O00748
MOL005100	ST3GAL3	Q11203
MOL005100	PTGS1	P23219
MOL005100	CYP19A1	P11511
MOL005100	CYP1B1	Q16678
MOL005100	DNMT1	P26358
MOL005100	POLB	P06746
MOL005100	TOP1	P11387
MOL005100	TOP2A	P11388
MOL005100	DYRK1B	Q9Y463
MOL005100	CLK1	P49759
MOL005100	DYRK1A	Q13627
MOL005100	EDNRA	P25101
MOL005100	HSD17B1	P14061
MOL005100	HSD17B2	P37059
MOL005100	ESR1	P03372
MOL005100	ESR2	Q92731
MOL005100	FASN	P49327
MOL005100	FFAR1	O14842
MOL005100	FUT4	P22083
MOL005100	GSK3B	P49841
MOL005100	PLA2G10	O15496
MOL005100	HSP90AB1	P08238
MOL005100	MET	P08581
MOL005100	KCNH2	Q12809
MOL005100	IGF1R	P08069
MOL005100	KLK1	P06870
MOL005100	KLK2	P20151
MOL005100	MMP12	P39900
MOL005100	MMP13	P45452
MOL005100	MMP14	P50281
MOL005100	MMP2	P08253
MOL005100	MMP3	P08254
MOL005100	MMP9	P14780
MOL005100	GRM2	Q14416
MOL005100	GRM5	P41594
MOL005100	MAOA	P21397
MOL005100	MAOB	P27338
MOL005100	PRKACA	P17612
MOL005100	ABCC1	P33527
MOL005100	MYLK	Q15746
MOL005100	CHRNA7	P36544
MOL005100	NCOA1	Q15788

MOL005100	NCOA2	Q15596
MOL005100	ODC1	P11926
MOL005100	PPARG	P37231
MOL005100	ABCB1	P08183
MOL005100	PIK3CG	P48736
MOL005100	PLA2G1B	P04054
MOL005100	PLA2G5	P39877
MOL005100	SERPINE1	P05121
MOL005100	PDGFRB	P09619
MOL005100	PARP1	P09874
MOL005100	PTGS2	P35354
MOL005100	RXRA	P19793
MOL005100	RPS6KA5	O75582
MOL005100	CHEK1	O14757
MOL005100	PIM1	P11309
MOL005100	PIM2	Q9P1W9
MOL005100	PIM3	Q86V86
MOL005100	SGK1	O00141
MOL005100	ERN1	O75460
MOL005100	STAT1	P42224
MOL005100	SCN5A	Q14524
MOL005100	SQLE	Q14534
MOL005100	TNKS	O95271
MOL005100	TNKS2	Q9H2K2
MOL005100	TAS2R31	P59538
MOL005100	TERT	O14746
MOL005100	SHBG	P04278
MOL005100	SRC	P12931
MOL005100	TDP1	Q9NUW8
MOL005100	KDR	P35968
MOL005944	HSD11B1	P28845
MOL005944	HSD17B7	P56937
MOL005944	MMP2	P08253
MOL005944	ADH1A	P07327
MOL005944	ADH1C	P00326
MOL005944	RNPEP	Q9H4A4
MOL005944	FAAH	O00519
MOL005944	DNPEP	Q9ULA0
MOL005944	BCHE	P06276
MOL005944	CASP3	P42574
MOL005944	CTSB	P07858
MOL005944	CTSK	P43235
MOL005944	CTSL	P07711
MOL005944	CD44	P16070

MOL005944	DPP7	Q9UHL4
MOL005944	DPP4	P27487
MOL005944	DPP9	Q86TI2
MOL005944	DPP8	Q6V1X1
MOL005944	DRD1	P21728
MOL005944	SLC6A3	Q01959
MOL005944	DNM1	Q05193
MOL005944	EPHX2	P34913
MOL005944	EPHX1	P07099
MOL005944	HPSE	Q9Y251
MOL005944	HDAC10	Q969S8
MOL005944	HDAC11	Q96DB2
MOL005944	HDAC2	Q92769
MOL005944	HDAC3	O15379
MOL005944	NCOR2	Q9Y618
MOL005944	HDAC6	Q9UBN7
MOL005944	HDAC8	Q9BY41
MOL005944	IER3IP1	Q9Y5U9
MOL005944	ITGA2B	P08514
MOL005944	ICAM1	P05362
MOL005944	IL6	P05231
MOL005944	MMP8	P22894
MOL005944	GRM5	P41594
MOL005944	METAP1	P53582
MOL005944	CHRM1	P11229
MOL005944	CHRM2	P08172
MOL005944	CHRM3	P20309
MOL005944	CHRM4	P08173
MOL005944	CHRM5	P08912
MOL005944	MYC	P01106
MOL005944	NAAA	Q02083
MOL005944	MME	P08473
MOL005944	CHRNA4	P43681
MOL005944	CHRNA7	P36544
MOL005944	CHRNA3	P32297
MOL005944	PARP1	P09874
MOL005944	PAOX	Q6QHF9
MOL005944	HTR1B	P28222
MOL005944	HTR1D	P28221
MOL005944	HTR3A	P46098
MOL005944	SIGMAR1	Q99720
MOL005944	SRD5A1	P18405
MOL005944	SRD5A2	P31213
MOL005944	RELA	Q04206

MOL005944	TNF	P01375
MOL005944	CHRNB2	P17787
MOL005944	CHRNB4	P30926
MOL005944	ITGB3	P05106
MOL006561	HSD11B1	P28845
MOL006561	ADRB2	P07550
MOL006561	ADRA1A	P35348
MOL006561	ADRA1D	P25100
MOL006561	FUCA1	P04066
MOL006561	RNPEP	Q9H4A4
MOL006561	AR	P10275
MOL006561	DNPEP	Q9ULA0
MOL006561	ADRB1	P08588
MOL006561	ADRB3	P13945
MOL006561	GBA	P04062
MOL006561	GBA2	Q9HCG7
MOL006561	BCHE	P06276
MOL006561	UGCG	Q16739
MOL006561	DPP4	P27487
MOL006561	DRD1	P21728
MOL006561	DRD3	P35462
MOL006561	DRD4	P21917
MOL006561	DNM1	Q05193
MOL006561	EPHX1	P07099
MOL006561	HSD17B3	P37058
MOL006561	FKBP1A	P62942
MOL006561	SLC6A9	P48067
MOL006561	PLA2G10	O15496
MOL006561	KCNH2	Q12809
MOL006561	HRH4	Q9H3N8
MOL006561	ITGA2B	P08514
MOL006561	KDM1A	O60341
MOL006561	GRM2	Q14416
MOL006561	GRM3	Q14832
MOL006561	GRM6	O15303
MOL006561	GRM8	O00222
MOL006561	MME	P08473
MOL006561	CHRNA7	P36544
MOL006561	CHRNB4	P30926
MOL006561	CHRNB3	Q05901
MOL006561	CHRNA3	P32297
MOL006561	CHRNA4	P43681
MOL006561	CHRNA5	P30532
MOL006561	SLC6A2	P23975

MOL006561	ABCB1	P08183
MOL006561	PLA2G2C	Q5R387
MOL006561	PLA2G5	P39877
MOL006561	PARP1	P09874
MOL006561	PTGER2	P43116
MOL006561	PTGER3	P43115
MOL006561	PTGER4	P35408
MOL006561	PRKCA	P17252
MOL006561	REN	P00797
MOL006561	ROCK2	O75116
MOL006561	HTR1A	P08908
MOL006561	HTR1B	P28222
MOL006561	HTR1D	P28221
MOL006561	HTR1F	P30939
MOL006561	HTR2C	P28335
MOL006561	HTR3A	P46098
MOL006561	SLC6A4	P31645
MOL006561	SRD5A2	P31213
MOL006561	TNNC1	P63316
MOL006561	JAK1	P23458
MOL006561	CHRNB2	P17787
MOL006561	ITGB3	P05106
MOL006561	TNNT2	P45379
MOL006561	TNNI3	P19429
MOL006561	CHRNA6	Q15825
MOL006561	CHRNA2	Q15822
MOL006563	HSD11B1	P28845
MOL006563	ADRA2C	P18825
MOL006563	ADRB2	P07550
MOL006563	ADRA1A	P35348
MOL006563	ADRA1B	P35368
MOL006563	ADRA2A	P08913
MOL006563	ADRA2B	P18089
MOL006563	FUCA1	P04066
MOL006563	RNPEP	Q9H4A4
MOL006563	AR	P10275
MOL006563	DNPEP	Q9ULA0
MOL006563	ADRB1	P08588
MOL006563	ADRB3	P13945
MOL006563	GBA	P04062
MOL006563	GBA2	Q9HCG7
MOL006563	BACE1	P56817
MOL006563	BCHE	P06276
MOL006563	UGCG	Q16739

MOL006563	CYP2D6	P10635
MOL006563	CYP51A1	Q16850
MOL006563	CTSC	P53634
MOL006563	DPP4	P27487
MOL006563	DRD1	P21728
MOL006563	DRD3	P35462
MOL006563	DRD4	P21917
MOL006563	DNM1	Q05193
MOL006563	EPHX1	P07099
MOL006563	HSD17B3	P37058
MOL006563	FKBP1A	P62942
MOL006563	SLC6A9	P48067
MOL006563	PLA2G10	O15496
MOL006563	KCNH2	Q12809
MOL006563	HRH1	P35367
MOL006563	HRH4	Q9H3N8
MOL006563	ITGA2B	P08514
MOL006563	GRM2	Q14416
MOL006563	GRM3	Q14832
MOL006563	GRM6	O15303
MOL006563	GRM8	O00222
MOL006563	MME	P08473
MOL006563	TACR1	P25103
MOL006563	CHRNA7	P36544
MOL006563	CHRNB4	P30926
MOL006563	CHRNB3	Q05901
MOL006563	CHRNA3	P32297
MOL006563	CHRNA4	P43681
MOL006563	CHRNA5	P30532
MOL006563	SLC6A2	P23975
MOL006563	ABCB1	P08183
MOL006563	PDE10A	Q9Y233
MOL006563	PLA2G2C	Q5R387
MOL006563	PLA2G5	P39877
MOL006563	PARP1	P09874
MOL006563	PTGER2	P43116
MOL006563	PTGER3	P43115
MOL006563	PTGER4	P35408
MOL006563	PRKCA	P17252
MOL006563	REN	P00797
MOL006563	ROCK2	O75116
MOL006563	HTR1A	P08908
MOL006563	HTR1B	P28222
MOL006563	HTR1D	P28221

MOL006563	HTR1F	P30939
MOL006563	HTR2C	P28335
MOL006563	HTR3A	P46098
MOL006563	HTR5A	P47898
MOL006563	SLC6A4	P31645
MOL006563	SCN5A	Q14524
MOL006563	SRD5A2	P31213
MOL006563	SLC18A2	Q05940
MOL006563	JAK1	P23458
MOL006563	UTS2R	Q9UKP6
MOL006563	KCNA5	P22460
MOL006563	CHRNA6	Q15825
MOL006563	CHRNB2	P17787
MOL006563	ROCK1	Q13464
MOL006563	CHRNA2	Q15822
MOL006563	ITGB3	P05106
MOL006564	HSD11B1	P28845
MOL006564	HSD17B7	P56937
MOL006564	ADH1A	P07327
MOL006564	ADH1C	P00326
MOL006564	RNPEP	Q9H4A4
MOL006564	FAAH	O00519
MOL006564	DNPEP	Q9ULA0
MOL006564	BCHE	P06276
MOL006564	CTSB	P07858
MOL006564	CTSK	P43235
MOL006564	CTSL	P07711
MOL006564	DPP7	Q9UHL4
MOL006564	DPP4	P27487
MOL006564	DPP9	Q86TI2
MOL006564	DPP8	Q6V1X1
MOL006564	DRD1	P21728
MOL006564	DRD2	P14416
MOL006564	SLC6A3	Q01959
MOL006564	DNM1	Q05193
MOL006564	EPHX2	P34913
MOL006564	EPHX1	P07099
MOL006564	HDAC10	Q969S8
MOL006564	HDAC11	Q96DB2
MOL006564	HDAC2	Q92769
MOL006564	HDAC3	O15379
MOL006564	NCOR2	Q9Y618
MOL006564	HDAC6	Q9UBN7
MOL006564	HDAC8	Q9BY41

MOL006564	ITGA2B	P08514
MOL006564	MMP8	P22894
MOL006564	GRM5	P41594
MOL006564	METAP1	P53582
MOL006564	SLC47A1	Q96FL8
MOL006564	CHRM1	P11229
MOL006564	CHRM2	P08172
MOL006564	CHRM3	P20309
MOL006564	CHRM4	P08173
MOL006564	CHRM5	P08912
MOL006564	NAAA	Q02083
MOL006564	MME	P08473
MOL006564	CHRNA4	P43681
MOL006564	CHRNA7	P36544
MOL006564	CHRNA3	P32297
MOL006564	PARP1	P09874
MOL006564	PAOX	Q6QHF9
MOL006564	PRMT3	O60678
MOL006564	HTR1B	P28222
MOL006564	HTR1D	P28221
MOL006564	HTR3B	O95264
MOL006564	HTR3A	P46098
MOL006564	SIGMAR1	Q99720
MOL006564	SLC22A2	O15244
MOL006564	SRD5A1	P18405
MOL006564	SRD5A2	P31213
MOL006564	ITGB3	P05106
MOL006564	CHRNB4	P30926
MOL006564	CHRNB2	P17787
MOL006566	ACHE	P22303
MOL006566	ADRA2B	P18089
MOL006566	CYP2D6	P10635
MOL006566	DRD1	P21728
MOL006566	KISS1R	Q969F8
MOL006566	SLC47A1	Q96FL8
MOL006566	CHRM4	P08173
MOL006566	CHRM5	P08912
MOL006566	CHRNA4	P43681
MOL006566	CHRNA3	P32297
MOL006566	OPRL1	P41146
MOL006566	SLC6A2	P23975
MOL006566	PARP1	P09874
MOL006566	PRMT3	O60678
MOL006566	HTR1B	P28222

MOL006566	HTR1D	P28221
MOL006566	HTR3B	O95264
MOL006566	HTR3A	P46098
MOL006566	SLC6A4	P31645
MOL006566	SIGMAR1	Q99720
MOL006566	SLC22A2	O15244
MOL006569	HSD11B1	P28845
MOL006569	FUCA1	P04066
MOL006569	RNPEP	Q9H4A4
MOL006569	AR	P10275
MOL006569	DNPEP	Q9ULA0
MOL006569	GBA	P04062
MOL006569	GBA2	Q9HCG7
MOL006569	BCHE	P06276
MOL006569	UGCG	Q16739
MOL006569	DPP4	P27487
MOL006569	DRD3	P35462
MOL006569	DRD4	P21917
MOL006569	DNM1	Q05193
MOL006569	EPHX1	P07099
MOL006569	HSD17B3	P37058
MOL006569	FKBP1A	P62942
MOL006569	PLA2G10	O15496
MOL006569	ITGA2B	P08514
MOL006569	LAP3	P28838
MOL006569	GRM2	Q14416
MOL006569	GRM3	Q14832
MOL006569	GRM6	O15303
MOL006569	GRM8	O00222
MOL006569	MME	P08473
MOL006569	CHRNA7	P36544
MOL006569	CHRNA3	P32297
MOL006569	CHRNA4	P43681
MOL006569	SLC6A2	P23975
MOL006569	PNMT	P11086
MOL006569	PLA2G2C	Q5R387
MOL006569	PLA2G5	P39877
MOL006569	PTGER2	P43116
MOL006569	PTGER3	P43115
MOL006569	PTGER4	P35408
MOL006569	PRKCA	P17252
MOL006569	REN	P00797
MOL006569	HTR3A	P46098
MOL006569	SLC6A4	P31645

MOL006569	SRD5A2	P31213
MOL006569	CHRNB4	P30926
MOL006569	ITGB3	P05106
MOL006569	CHRNB2	P17787
MOL006571	CHRNB1	P11230
MOL006571	ADRA2B	P18089
MOL006571	CYP2D6	P10635
MOL006571	DRD2	P14416
MOL006571	DRD3	P35462
MOL006571	SLC6A3	Q01959
MOL006571	KISS1R	Q969F8
MOL006571	CHRNA4	P43681
MOL006571	CHRNA7	P36544
MOL006571	CHRNA3	P32297
MOL006571	PARP1	P09874
MOL006571	PRMT3	O60678
MOL006571	HTR2B	P41595
MOL006571	HTR7	P34969
MOL006571	SRD5A2	P31213
MOL006571	CHRNB2	P17787
MOL006571	CHRNB4	P30926
MOL006571	CHRNA1	P02708
MOL006571	CHRNG	P07510
MOL006571	CHRND	Q07001
MOL006582	ADRB2	P07550
MOL006582	FUCA1	P04066
MOL006582	RNPEP	Q9H4A4
MOL006582	AR	P10275
MOL006582	DNPEP	Q9ULA0
MOL006582	KCNJ1	P48048
MOL006582	ADRB1	P08588
MOL006582	ADRB3	P13945
MOL006582	GBA	P04062
MOL006582	GBA2	Q9HCG7
MOL006582	NR1H4	Q96RI1
MOL006582	CNR1	P21554
MOL006582	CNR2	P34972
MOL006582	UGCG	Q16739
MOL006582	DPP4	P27487
MOL006582	DRD3	P35462
MOL006582	DRD4	P21917
MOL006582	DNM1	Q05193
MOL006582	EPHX1	P07099
MOL006582	HSD17B3	P37058

MOL006582	GRIN1	Q05586
MOL006582	PLA2G10	O15496
MOL006582	KCNH2	Q12809
MOL006582	ITGA2B	P08514
MOL006582	GRM2	Q14416
MOL006582	GRM3	Q14832
MOL006582	GRM6	O15303
MOL006582	GRM8	O00222
MOL006582	MME	P08473
MOL006582	CHRNA7	P36544
MOL006582	CHRNA3	P32297
MOL006582	CHRNA4	P43681
MOL006582	OPRL1	P41146
MOL006582	SLC6A2	P23975
MOL006582	ABCB1	P08183
MOL006582	PDE10A	Q9Y233
MOL006582	PDE11A	Q9HCR9
MOL006582	PDE5A	O76074
MOL006582	PLA2G2A	P14555
MOL006582	PLA2G2C	Q5R387
MOL006582	PLA2G5	P39877
MOL006582	PTGER2	P43116
MOL006582	PTGER3	P43115
MOL006582	PTGER4	P35408
MOL006582	PRKCA	P17252
MOL006582	PNP	P00491
MOL006582	REN	P00797
MOL006582	HTR1A	P08908
MOL006582	HTR1D	P28221
MOL006582	HTR2C	P28335
MOL006582	HTR3A	P46098
MOL006582	SLC6A4	P31645
MOL006582	MTAP	Q13126
MOL006582	SRD5A2	P31213
MOL006582	TRPV3	Q8NET8
MOL006582	TNNC1	P63316
MOL006582	KCNA5	P22460
MOL006582	TNNT2	P45379
MOL006582	TNNI3	P19429
MOL006582	GRIN2B	Q13224
MOL006582	CHRNB4	P30926
MOL006582	ITGB3	P05106
MOL006582	CHRNB2	P17787
MOL006596	HPGD	P15428

MOL006596	ALOX5AP	P20292
MOL006596	ADAM10	O14672
MOL006596	ADAM17	P78536
MOL006596	ALPL	P05186
MOL006596	AR	P10275
MOL006596	BRD4	O60885
MOL006596	KCNMA1	Q12791
MOL006596	CALM1	P62158
MOL006596	CDK2	P24941
MOL006596	F8	P00451
MOL006596	F10	P00742
MOL006596	CCNA2	P20248
MOL006596	CDK1	P06493
MOL006596	TOP1	P11387
MOL006596	TOP2A	P11388
MOL006596	HSD17B3	P37058
MOL006596	ESR1	P03372
MOL006596	ESR2	Q92731
MOL006596	EIF2AK3	Q9NZJ5
MOL006596	GSK3B	P49841
MOL006596	HDAC2	Q92769
MOL006596	FCER2	P06734
MOL006596	RET	P07949
MOL006596	PYGL	P06737
MOL006596	FKBP1A	P62942
MOL006596	MAPK3	P27361
MOL006596	MAPK1	P28482
MOL006596	MMP7	P09237
MOL006596	MMP8	P22894
MOL006596	NOS2	P35228
MOL006596	NCOA1	Q15788
MOL006596	NCOA2	Q15596
MOL006596	P2RX3	P56373
MOL006596	PDE10A	Q9Y233
MOL006596	PDE4D	Q08499
MOL006596	PLAA	Q9Y263
MOL006596	PIK3CD	O00329
MOL006596	PIK3CG	P48736
MOL006596	PTGS1	P23219
MOL006596	PTGS2	P35354
MOL006596	PTPN1	P18031
MOL006596	PIM1	P11309
MOL006596	CHEK1	O14757
MOL006596	MTOR	P42345

MOL006596	WEE1	P30291
MOL006596	SCN5A	Q14524
MOL006596	PRSS1	P07477
MOL006596	AGTR1	P30556
MOL006596	ITK	Q08881
MOL006596	SYK	P43405
MOL006596	FLT1	P17948
MOL006596	KCNA5	P22460
MOL006604	ADRA1B	P35368
MOL006604	CALM1	P62158
MOL006604	F10	P00742
MOL006604	TOP2A	P11388
MOL006604	ESR1	P03372
MOL006604	HSP90AB1	P08238
MOL006604	NOS3	P29474
MOL006604	NOS1	P29475
MOL006604	NCOA1	Q15788
MOL006604	NCOA2	Q15596
MOL006604	KCNH2	Q12809
MOL006604	PTGS2	P35354
MOL006604	SCN5A	Q14524
MOL006604	KDR	P35968
MOL006613	YWHAG	P61981
MOL006613	ADAM17	P78536
MOL006613	AKR1B1	P15121
MOL006613	AR	P10275
MOL006613	ALOX12	P18054
MOL006613	ALOX15	P16050
MOL006613	ALOX15B	O15296
MOL006613	ADRB2	P07550
MOL006613	GUSB	P08236
MOL006613	CALM1	P62158
MOL006613	CA1	P00915
MOL006613	CA2	P00918
MOL006613	CA3	P07451
MOL006613	LNPEP	Q9UIQ6
MOL006613	CYP19A1	P11511
MOL006613	DPP4	P27487
MOL006613	TOP1	P11387
MOL006613	DYRK1B	Q9Y463
MOL006613	CLK1	P49759
MOL006613	DNM1	Q05193
MOL006613	HSD17B2	P37059
MOL006613	HSD17B3	P37058

MOL006613	ESR1	P03372
MOL006613	ESR2	Q92731
MOL006613	GSK3B	P49841
MOL006613	HSP90AB1	P08238
MOL006613	MET	P08581
MOL006613	HDAC4	P56524
MOL006613	HDAC5	Q9UQL6
MOL006613	HDAC7	Q8WUI4
MOL006613	HDAC9	Q9UKV0
MOL006613	IMPDH1	P20839
MOL006613	IMPDH2	P12268
MOL006613	MMP14	P50281
MOL006613	MMP16	P51512
MOL006613	MMP2	P08253
MOL006613	MMP8	P22894
MOL006613	MMP9	P14780
MOL006613	MAP3K8	P41279
MOL006613	PRKACA	P17612
MOL006613	CHRNA7	P36544
MOL006613	NOS2	P35228
MOL006613	NCOA1	Q15788
MOL006613	PIK3CG	P48736
MOL006613	PARP1	P09874
MOL006613	PTGS1	P23219
MOL006613	PTGS2	P35354
MOL006613	PTPN1	P18031
MOL006613	RXRA	P19793
MOL006613	RPS6KA5	O75582
MOL006613	BRAF	P15056
MOL006613	CHEK1	O14757
MOL006613	PIM2	Q9P1W9
MOL006613	PIM3	Q86V86
MOL006613	SGK1	O00141
MOL006613	WEE1	P30291
MOL006613	ERN1	O75460
MOL006613	SCN5A	Q14524
MOL006613	TNKS	O95271
MOL006613	TNKS2	Q9H2K2
MOL006613	TYMS	P04818
MOL006613	TRPM8	Q7Z2W7
MOL006613	TTR	P02766
MOL006613	PRSS1	P07477
MOL006613	SRC	P12931
MOL006613	KDR	P35968

MOL006620	ADRB2	P07550
MOL006620	CALM1	P62158
MOL006620	HSP90AB1	P08238
MOL006620	PRKACA	P17612
MOL006620	PTGS1	P23219
MOL006620	PTGS2	P35354
MOL006620	RXRA	P19793
MOL006620	SCN5A	Q14524
MOL006622	ALDH2	P05091
MOL006622	IL2	P60568
MOL006622	TNF	P01375
MOL006623	ACHE	P22303
MOL006623	ADAM10	O14672
MOL006623	ADAM17	P78536
MOL006623	ADAMTS4	O75173
MOL006623	ADAMTS5	Q9UNA0
MOL006623	ADORA1	P30542
MOL006623	ADORA3	P0DMS8
MOL006623	AKR1B10	O60218
MOL006623	AKR1C3	P42330
MOL006623	AKR1B1	P15121
MOL006623	ABCG2	Q9UNQ0
MOL006623	KCNJ1	P48048
MOL006623	BACE1	P56817
MOL006623	BCHE	P06276
MOL006623	CA4	P22748
MOL006623	CA7	P43166
MOL006623	CA12	O43570
MOL006623	CBR1	P16152
MOL006623	CES2	O00748
MOL006623	CCR1	P32246
MOL006623	CDK9	P50750
MOL006623	F7	P08709
MOL006623	F10	P00742
MOL006623	CFD	P00746
MOL006623	CXCR3	P49682
MOL006623	CDK1	P06493
MOL006623	CCNB3	Q8WWL7
MOL006623	CDK2	P24941
MOL006623	CCNE2	O96020
MOL006623	CCNE1	P24864
MOL006623	CCND1	P24385
MOL006623	CDK5R1	Q15078
MOL006623	CDK7	P50613

MOL006623	PTGS1	P23219
MOL006623	CYP19A1	P11511
MOL006623	CYP1B1	Q16678
MOL006623	OPRD1	P41143
MOL006623	TOP2A	P11388
MOL006623	DUT	P33316
MOL006623	EDNRA	P25101
MOL006623	HSD17B1	P14061
MOL006623	ESR1	P03372
MOL006623	ESR2	Q92731
MOL006623	EZH2	Q15910
MOL006623	PSEN2	P49810
MOL006623	GSK3A	P49840
MOL006623	GSK3B	P49841
MOL006623	HSP90AA1	P07900
MOL006623	HSP90AB1	P08238
MOL006623	IGF1R	P08069
MOL006623	IRAK4	Q9NWZ3
MOL006623	KLK2	P20151
MOL006623	LRRK2	Q5S007
MOL006623	LIMK1	P53667
MOL006623	CD38	P28907
MOL006623	FKBP1A	P62942
MOL006623	MAPK3	P27361
MOL006623	MMP1	P03956
MOL006623	MMP12	P39900
MOL006623	MMP13	P45452
MOL006623	MMP3	P08254
MOL006623	MMP7	P09237
MOL006623	MMP8	P22894
MOL006623	MAOB	P27338
MOL006623	OPRM1	P35372
MOL006623	NOS2	P35228
MOL006623	P2RX3	P56373
MOL006623	MDM2	Q00987
MOL006623	PPARG	P37231
MOL006623	PIK3C2B	O00750
MOL006623	PDE10A	Q9Y233
MOL006623	PDE4B	Q07343
MOL006623	PLCG1	P19174
MOL006623	PIK3CA	P42336
MOL006623	PIK3CB	P42338
MOL006623	PIK3CG	P48736
MOL006623	SERPINE1	P05121

MOL006623	PARP1	P09874
MOL006623	PTGS2	P35354
MOL006623	PRKCB	P05771
MOL006623	PTPN1	P18031
MOL006623	ROS1	P08922
MOL006623	PDK1	Q15118
MOL006623	ERBB2	P04626
MOL006623	ROCK2	O75116
MOL006623	RPS6KB1	P23443
MOL006623	RPS6KA3	P51812
MOL006623	AURKA	O14965
MOL006623	CHEK1	O14757
MOL006623	MTOR	P42345
MOL006623	ACVRL1	P37023
MOL006623	WEE1	P30291
MOL006623	SLC5A2	P31639
MOL006623	SORD	Q00796
MOL006623	TAS2R31	P59538
MOL006623	SHBG	P04278
MOL006623	F2	P00734
MOL006623	ITK	Q08881
MOL006623	JAK1	P23458
MOL006623	JAK2	O60674
MOL006623	JAK3	P52333
MOL006623	KDR	P35968
MOL006623	VDR	P11473
MOL006623	P06493	CDK1
MOL006623	P14635	CCNB1
MOL006623	O95067	CCNB2
MOL006623	CCNA1	P78396
MOL006623	CCNA2	P20248
MOL006623	CDK5	Q00535
MOL006623	CCNH	P51946
MOL006623	CCNT1	O60563
MOL006623	CCNB1	P14635
MOL006623	CDK4	P11802
MOL006623	PSENEN	Q9NZ42
MOL006623	NCSTN	Q92542
MOL006623	APH1A	Q96BI3
MOL006623	PSEN1	P49768
MOL006623	APH1B	Q8WW43
MOL006626	YWHAG	P61981
MOL006626	HSD17B14	Q9BPX1
MOL006626	ACHE	P22303

MOL006626	CES1	P23141
MOL006626	ADAM17	P78536
MOL006626	ADORA1	P30542
MOL006626	ADORA2B	P29275
MOL006626	ADORA3	P0DMS8
MOL006626	AKR1B10	O60218
MOL006626	AKR1C3	P42330
MOL006626	AKR1B1	P15121
MOL006626	ANPEP	P15144
MOL006626	BCL2L1	Q07817
MOL006626	ALOX5	P09917
MOL006626	ABCG2	Q9UNQ0
MOL006626	BACE1	P56817
MOL006626	BCHE	P06276
MOL006626	PRKACA	P17612
MOL006626	CA1	P00915
MOL006626	CA2	P00918
MOL006626	CA4	P22748
MOL006626	CA7	P43166
MOL006626	CA12	O43570
MOL006626	CBR1	P16152
MOL006626	CES2	O00748
MOL006626	CTSK	P43235
MOL006626	CTSL	P07711
MOL006626	CCNE1	P24864
MOL006626	F10	P00742
MOL006626	CDK1	P06493
MOL006626	PTGS1	P23219
MOL006626	CYP19A1	P11511
MOL006626	CYP1B1	Q16678
MOL006626	QDPR	P09417
MOL006626	POLB	P06746
MOL006626	TOP2A	P11388
MOL006626	MAP2K1	Q02750
MOL006626	EPHB4	P54760
MOL006626	EPHA2	P29317
MOL006626	EPHB2	P29323
MOL006626	HSD17B1	P14061
MOL006626	HSD17B2	P37059
MOL006626	ESR1	P03372
MOL006626	ESR2	Q92731
MOL006626	GCGR	P47871
MOL006626	GSK3B	P49841
MOL006626	GRK2	P25098

MOL006626	PLA2G10	O15496
MOL006626	HSP90AB1	P08238
MOL006626	HSP90AA1	P07900
MOL006626	INSR	P06213
MOL006626	IGF1R	P08069
MOL006626	KLK2	P20151
MOL006626	KDM1A	O60341
MOL006626	MMP1	P03956
MOL006626	MMP12	P39900
MOL006626	MMP13	P45452
MOL006626	MMP15	P51511
MOL006626	MMP16	P51512
MOL006626	MMP26	Q9NRE1
MOL006626	MMP3	P08254
MOL006626	MMP8	P22894
MOL006626	MAOB	P27338
MOL006626	ABCC1	P33527
MOL006626	NOX4	Q9NPH5
MOL006626	CHRNA7	P36544
MOL006626	NOS3	P29474
MOL006626	NCOA1	Q15788
MOL006626	ODC1	P11926
MOL006626	PPARG	P37231
MOL006626	PDE7A	Q13946
MOL006626	PLA2G1B	P04054
MOL006626	PLA2G5	P39877
MOL006626	PIK3CG	P48736
MOL006626	SERPINE1	P05121
MOL006626	PTGS2	P35354
MOL006626	PTPN1	P18031
MOL006626	PDK1	Q15118
MOL006626	ERBB2	P04626
MOL006626	RXRA	P19793
MOL006626	ROCK1	Q13464
MOL006626	RPS6KA3	P51812
MOL006626	RPS6KA5	O75582
MOL006626	AURKA	O14965
MOL006626	BRAF	P15056
MOL006626	CHEK1	O14757
MOL006626	MTOR	P42345
MOL006626	PIM1	P11309
MOL006626	PIM2	Q9P1W9
MOL006626	PIM3	Q86V86
MOL006626	RAF1	P04049

MOL006626	WEE1	P30291
MOL006626	ERN1	O75460
MOL006626	SCN5A	Q14524
MOL006626	SLC5A2	P31639
MOL006626	TAS2R31	P59538
MOL006626	SHBG	P04278
MOL006626	TNF	P01375
MOL006626	BLK	P51451
MOL006626	BMX	P51813
MOL006626	CSK	P41240
MOL006626	FYN	P06241
MOL006626	LCK	P06239
MOL006626	SYK	P43405
MOL006626	YES1	P07947
MOL006626	UPP1	Q16831
MOL006626	KDR	P35968
MOL006626	CDK2	P24941
MOL006626	CDK3	Q00526
MOL006626	CCNB1	P14635
MOL006627	ACHE	P22303
MOL006627	ADRA2B	P18089
MOL006627	CYP2D6	P10635
MOL006627	DRD1	P21728
MOL006627	KISS1R	Q969F8
MOL006627	SLC47A1	Q96FL8
MOL006627	CHRM4	P08173
MOL006627	CHRM5	P08912
MOL006627	CHRNA4	P43681
MOL006627	CHRNA3	P32297
MOL006627	OPRL1	P41146
MOL006627	SLC6A2	P23975
MOL006627	PARP1	P09874
MOL006627	PRMT3	O60678
MOL006627	HTR1B	P28222
MOL006627	HTR1D	P28221
MOL006627	HTR3B	O95264
MOL006627	HTR3A	P46098
MOL006627	SLC6A4	P31645
MOL006627	SIGMAR1	Q99720
MOL006627	SLC22A2	O15244
MOL006628	HSD11B1	P28845
MOL006628	HSD17B7	P56937
MOL006628	ADH1A	P07327
MOL006628	ADH1C	P00326

MOL006628	ADRA2A	P08913
MOL006628	RNPEP	Q9H4A4
MOL006628	FAAH	O00519
MOL006628	DNPEP	Q9ULA0
MOL006628	BCHE	P06276
MOL006628	CTSB	P07858
MOL006628	CTSK	P43235
MOL006628	CTSL	P07711
MOL006628	DPP7	Q9UHL4
MOL006628	DPP4	P27487
MOL006628	DPP9	Q86TI2
MOL006628	DPP8	Q6V1X1
MOL006628	SLC6A3	Q01959
MOL006628	DNM1	Q05193
MOL006628	EPHX2	P34913
MOL006628	EPHX1	P07099
MOL006628	HDAC1	Q13547
MOL006628	HDAC10	Q969S8
MOL006628	HDAC11	Q96DB2
MOL006628	HDAC2	Q92769
MOL006628	HDAC3	O15379
MOL006628	NCOR2	Q9Y618
MOL006628	HDAC6	Q9UBN7
MOL006628	HDAC8	Q9BY41
MOL006628	ITGA2B	P08514
MOL006628	MMP8	P22894
MOL006628	GRM5	P41594
MOL006628	METAP1	P53582
MOL006628	CHRM1	P11229
MOL006628	CHRM2	P08172
MOL006628	NAAA	Q02083
MOL006628	MME	P08473
MOL006628	CHRNA4	P43681
MOL006628	CHRNA7	P36544
MOL006628	CHRNA3	P32297
MOL006628	PARP1	P09874
MOL006628	PAOX	Q6QHF9
MOL006628	REN	P00797
MOL006628	SCARB1	Q8WTV0
MOL006628	HTR1B	P28222
MOL006628	HTR1D	P28221
MOL006628	HTR2B	P41595
MOL006628	HTR3A	P46098
MOL006628	SIGMAR1	Q99720

MOL006628	SRD5A1	P18405
MOL006628	SRD5A2	P31213
MOL006628	ITGB3	P05106
MOL006628	CHRNB4	P30926
MOL006628	CHRNB2	P17787
MOL006630	ACHE	P22303
MOL006630	ADORA1	P30542
MOL006630	ADORA2A	P29274
MOL006630	AKR1A1	P14550
MOL006630	AKR1B10	O60218
MOL006630	AKR1C1	Q04828
MOL006630	AKR1C2	P52895
MOL006630	AKR1C4	P17516
MOL006630	AKR1C3	P42330
MOL006630	AKR1B1	P15121
MOL006630	ALK	Q9UM73
MOL006630	AMY1A	P04745
MOL006630	AR	P10275
MOL006630	ALOX12	P18054
MOL006630	ALOX15	P16050
MOL006630	ALOX5	P09917
MOL006630	ARG1	P05089
MOL006630	AHR	P35869
MOL006630	ABCG2	Q9UNQ0
MOL006630	APP	P05067
MOL006630	BACE1	P56817
MOL006630	CAMK2B	Q13554
MOL006630	CA1	P00915
MOL006630	CA2	P00918
MOL006630	CA3	P07451
MOL006630	CA4	P22748
MOL006630	CA9	Q16790
MOL006630	CA5A	P35218
MOL006630	CA6	P23280
MOL006630	CA7	P43166
MOL006630	CA12	O43570
MOL006630	CA13	Q8N1Q1
MOL006630	CA14	Q9ULX7
MOL006630	CSNK2A1	P68400
MOL006630	CDK1	P06493
MOL006630	CCNB3	Q8WWL7
MOL006630	CDK2	P24941
MOL006630	CDK5R1	Q15078
MOL006630	CDK6	Q00534

MOL006630	PTGS2	P35354
MOL006630	CFTR	P13569
MOL006630	CYP19A1	P11511
MOL006630	CYP1B1	Q16678
MOL006630	DAPK1	P53355
MOL006630	TOP1	P11387
MOL006630	MPG	P29372
MOL006630	DRD4	P21917
MOL006630	EGFR	P00533
MOL006630	HSD17B1	P14061
MOL006630	HSD17B2	P37059
MOL006630	ESR1	P03372
MOL006630	ESR2	Q92731
MOL006630	ESRRA	P11474
MOL006630	PTK2	Q05397
MOL006630	GRK6	P43250
MOL006630	GSK3B	P49841
MOL006630	GLO1	Q04760
MOL006630	GPR35	Q9HC97
MOL006630	HSP90AB1	P08238
MOL006630	MET	P08581
MOL006630	IGF1R	P08069
MOL006630	CXCR1	P25024
MOL006630	PYGL	P06737
MOL006630	CD38	P28907
MOL006630	MMP12	P39900
MOL006630	MMP13	P45452
MOL006630	MMP2	P08253
MOL006630	MMP3	P08254
MOL006630	MMP9	P14780
MOL006630	MAOA	P21397
MOL006630	PRKACA	P17612
MOL006630	ABCC1	P33527
MOL006630	MPO	P05164
MOL006630	NOX4	Q9NPH5
MOL006630	NAE1	Q13564
MOL006630	NUAK1	O60285
MOL006630	ABCB1	P08183
MOL006630	PLA2G1B	P04054
MOL006630	PIK3R1	P27986
MOL006630	PARP1	P09874
MOL006630	PTGS1	P23219
MOL006630	PKN1	Q16512
MOL006630	PTPRS	Q13332

MOL006630	AKT1	P31749
MOL006630	AURKB	Q96GD4
MOL006630	NEK2	P51955
MOL006630	NEK6	Q9HC98
MOL006630	PIM1	P11309
MOL006630	PLK1	P53350
MOL006630	SLC22A12	Q96S37
MOL006630	TNKS	O95271
MOL006630	TNKS2	Q9H2K2
MOL006630	TERT	O14746
MOL006630	F2	P00734
MOL006630	TTR	P02766
MOL006630	TYR	P14679
MOL006630	FLT3	P36888
MOL006630	AXL	P30530
MOL006630	SRC	P12931
MOL006630	SYK	P43405
MOL006630	KDR	P35968
MOL006630	AVPR2	P30518
MOL006630	XDH	P47989
MOL006630	CCNB1	P14635
MOL006630	CCNB2	O95067
MOL006630	CDK5	Q00535
MOL006650	ADORA1	P30542
MOL006650	ADORA3	P0DMS8
MOL006650	PTGS1	P23219
MOL006650	CYP19A1	P11511
MOL006650	TOP2A	P11388
MOL006650	EIF4A1	P60842
MOL006650	NPC1L1	Q9UHC9
MOL006652	ADORA1	P30542
MOL006652	ALDH2	P05091
MOL006652	CA14	Q9ULX7
MOL006652	PTGS1	P23219
MOL006652	PTGS2	P35354
MOL006652	SLC29A1	Q99808
MOL006652	EIF4A1	P60842
MOL006652	IL2	P60568
MOL006652	TYR	P14679
MOL006824	HSD11B1	P28845
MOL006824	HSD11B2	P80365
MOL006824	ACHE	P22303
MOL006824	SCD	O00767
MOL006824	ADORA3	P0DMS8

MOL006824	AKR1B10	O60218
MOL006824	AR	P10275
MOL006824	ALOX5	P09917
MOL006824	BCHE	P06276
MOL006824	CNR1	P21554
MOL006824	CES2	O00748
MOL006824	CD81	P60033
MOL006824	CYP17A1	P05093
MOL006824	CYP19A1	P11511
MOL006824	CYP2C19	P33261
MOL006824	CYP51A1	Q16850
MOL006824	POLB	P06746
MOL006824	CDC25A	P30304
MOL006824	CDC25B	P30305
MOL006824	ESR1	P03372
MOL006824	ESR2	Q92731
MOL006824	FABP4	P15090
MOL006824	FABP5	Q01469
MOL006824	FABP3	P05413
MOL006824	FABP1	P07148
MOL006824	HMGCR	P04035
MOL006824	ACP1	P24666
MOL006824	NR1H3	Q13133
MOL006824	MAPK3	P27361
MOL006824	CHRM2	P08172
MOL006824	NPC1L1	Q9UHC9
MOL006824	SLC6A2	P23975
MOL006824	RORA	P35398
MOL006824	RORC	P51449
MOL006824	NR1I3	Q14994
MOL006824	PPARA	Q07869
MOL006824	PPARD	Q03181
MOL006824	PPARG	P37231
MOL006824	PDE4D	Q08499
MOL006824	PLA2G1B	P04054
MOL006824	PREP	P48147
MOL006824	PTGES	O14684
MOL006824	FNTA	P49354
MOL006824	PTPN1	P18031
MOL006824	PTPN6	P29350
MOL006824	PTPN11	Q06124
MOL006824	PTPRF	P10586
MOL006824	SLC6A4	P31645
MOL006824	SQLE	Q14534

MOL006824	SREBF2	Q12772
MOL006824	PTPN2	P17706
MOL006824	TERT	O14746
MOL006824	SHBG	P04278
MOL006824	UGT2B7	P16662
MOL006824	FNTB	P49356
MOL007036	ACHE	P22303
MOL007036	ADRA1A	P35348
MOL007036	ADRA1B	P35368
MOL007036	ADRB2	P07550
MOL007036	CA2	P00918
MOL007036	TOP2A	P11388
MOL007036	IGHG1	P01857
MOL007036	CHRM1	P11229
MOL007036	CHRM3	P20309
MOL007036	OPRM1	P35372
MOL007036	NCOA2	Q15596
MOL007036	PTGS1	P23219
MOL007036	PTGS2	P35354
MOL007036	RXRA	P19793
MOL007036	SCN5A	Q14524
MOL007045	ACHE	P22303
MOL007045	ADRB2	P07550
MOL007045	OPRD1	P41143
MOL007045	DPP4	P27487
MOL007045	CHRM1	P11229
MOL007045	CHRM5	P08912
MOL007045	OPRM1	P35372
MOL007045	CHRNA7	P36544
MOL007045	NCOA1	Q15788
MOL007045	PTGS2	P35354
MOL007045	SCN5A	Q14524
MOL007045	F2	P00734
MOL007045	PRSS1	P07477
MOL007048	PTPN1	P18031
MOL007048	PTGS2	P35354
MOL007049	HSD11B1	P28845
MOL007049	PFKFB3	Q16875
MOL007049	ACHE	P22303
MOL007049	CES1	P23141
MOL007049	ADAM17	P78536
MOL007049	ADRA2A	P08913
MOL007049	ADRA2C	P18825
MOL007049	AR	P10275

MOL007049	CA2	P00918
MOL007049	CES2	O00748
MOL007049	CASP3	P42574
MOL007049	CASP7	P55210
MOL007049	TOP1	P11387
MOL007049	DRD1	P21728
MOL007049	CDC25A	P30304
MOL007049	CDC25B	P30305
MOL007049	ESR1	P03372
MOL007049	GABRA2	P47869
MOL007049	GABRB3	P28472
MOL007049	PTPN22	Q9Y2R2
MOL007049	MMP13	P45452
MOL007049	MMP8	P22894
MOL007049	MTNR1A	P48039
MOL007049	MTNR1B	P49286
MOL007049	GRM5	P41594
MOL007049	MAOB	P27338
MOL007049	CHRM1	P11229
MOL007049	CHRM2	P08172
MOL007049	CHRM3	P20309
MOL007049	CHRM4	P08173
MOL007049	CHRM5	P08912
MOL007049	NOS1	P29475
MOL007049	PPARG	P37231
MOL007049	PTGS1	P23219
MOL007049	PTGS2	P35354
MOL007049	PBRM1	Q86U86
MOL007049	PTPN1	P18031
MOL007049	SCN5A	Q14524
MOL007049	TNKS	O95271
MOL007049	TNKS2	Q9H2K2
MOL007049	SMARCA4	P51532
MOL007049	VCP	P55072
MOL007049	GABRB2	P47870
MOL007049	GABRG2	P18507
MOL007049	GABRA3	P34903
MOL007049	GABRA1	P14867
MOL007049	GABRA5	P31644
MOL007050	AR	P10275
MOL007050	CDK2	P24941
MOL007050	CCNA2	P20248
MOL007050	ESR1	P03372
MOL007050	ESR2	Q92731

MOL007050	GSK3B	P49841
MOL007050	HSP90AB1	P08238
MOL007050	MAPK14	Q16539
MOL007050	NOS2	P35228
MOL007050	PPARG	P37231
MOL007050	PIM1	P11309
MOL007050	F2	P00734
MOL007058	CES1	P23141
MOL007058	AKR1B1	P15121
MOL007058	AR	P10275
MOL007058	CES2	O00748
MOL007058	MAPK8	P45983
MOL007058	MAPK10	P53779
MOL007058	CYP11B1	P15538
MOL007058	CYP11B2	P19099
MOL007058	DPP4	P27487
MOL007058	CDC25A	P30304
MOL007058	CDC25B	P30305
MOL007058	CDC25C	P30307
MOL007058	EGFR	P00533
MOL007058	EED	O75530
MOL007058	RBBP4	Q09028
MOL007058	NR3C1	P04150
MOL007058	HRH3	Q9Y5N1
MOL007058	HRH4	Q9H3N8
MOL007058	PTPRC	P08575
MOL007058	ELANE	P08246
MOL007058	MPI	P34949
MOL007058	GRM5	P41594
MOL007058	NR3C2	P08235
MOL007058	MAOB	P27338
MOL007058	PRKACA	P17612
MOL007058	NCOA1	Q15788
MOL007058	P2RX7	Q99572
MOL007058	PIK3CG	P48736
MOL007058	PGR	P06401
MOL007058	PTGS2	P35354
MOL007058	PTPN6	P29350
MOL007058	PTPN11	Q06124
MOL007058	ERBB2	P04626
MOL007058	RXRA	P19793
MOL007058	TERT	O14746
MOL007058	F2	P00734
MOL007058	SUZ12	Q15022

MOL007058	EZH2	Q15910
MOL007058	RBBP7	Q16576
MOL007059	ACHE	P22303
MOL007059	ADRA1A	P35348
MOL007059	ADRB2	P07550
MOL007059	CA2	P00918
MOL007059	OPRD1	P41143
MOL007059	DPP4	P27487
MOL007059	DRD1	P21728
MOL007059	HSP90AB1	P08238
MOL007059	IGHG1	P01857
MOL007059	CHRM1	P11229
MOL007059	OPRM1	P35372
MOL007059	CHRNA7	P36544
MOL007059	NCOA1	Q15788
MOL007059	PTGS2	P35354
MOL007059	RXRA	P19793
MOL007059	F2	P00734
MOL007059	PRSS1	P07477
MOL007061	HSD11B1	P28845
MOL007061	HTR2A	P28223
MOL007061	ACHE	P22303
MOL007061	CES1	P23141
MOL007061	ADORA2A	P29274
MOL007061	ADORA2B	P29275
MOL007061	AKR1B1	P15121
MOL007061	ADRA1A	P35348
MOL007061	AR	P10275
MOL007061	ALOX5	P09917
MOL007061	ADRB2	P07550
MOL007061	BDKRB2	P30411
MOL007061	CA2	P00918
MOL007061	CES2	O00748
MOL007061	CYP11B1	P15538
MOL007061	CYP11B2	P19099
MOL007061	CYP19A1	P11511
MOL007061	OPRD1	P41143
MOL007061	DPP4	P27487
MOL007061	DRD1	P21728
MOL007061	SLC6A3	Q01959
MOL007061	CDC25B	P30305
MOL007061	CDC25C	P30307
MOL007061	EPHB4	P54760
MOL007061	EGFR	P00533

MOL007061	EPHX1	P07099
MOL007061	ESR2	Q92731
MOL007061	EED	O75530
MOL007061	RBBP4	Q09028
MOL007061	GABRA5	P31644
MOL007061	GABRA1	P14867
MOL007061	HSP90AB1	P08238
MOL007061	IGHG1	P01857
MOL007061	MCL1	Q07820
MOL007061	IMPDH2	P12268
MOL007061	IGF1R	P08069
MOL007061	CXCR2	P25025
MOL007061	IDH1	O75874
MOL007061	PTPRC	P08575
MOL007061	MAPK14	Q16539
MOL007061	MTNR1A	P48039
MOL007061	MTNR1B	P49286
MOL007061	MAOA	P21397
MOL007061	MAOB	P27338
MOL007061	CHRM1	P11229
MOL007061	CHRM2	P08172
MOL007061	CHRM3	P20309
MOL007061	CHRM5	P08912
MOL007061	OPRM1	P35372
MOL007061	CHRNA7	P36544
MOL007061	NCOA1	Q15788
MOL007061	NR4A1	P22736
MOL007061	HCRTR1	O43613
MOL007061	HCRTR2	O43614
MOL007061	P2RX7	Q99572
MOL007061	PRF1	P14222
MOL007061	ABCB1	P08183
MOL007061	PDE5A	O76074
MOL007061	PABPC1	P11940
MOL007061	PGR	P06401
MOL007061	PREP	P48147
MOL007061	PTGS2	P35354
MOL007061	PTPN6	P29350
MOL007061	PTPN11	Q06124
MOL007061	NQO2	P16083
MOL007061	RXRA	P19793
MOL007061	SLC6A4	P31645
MOL007061	SCN5A	Q14524
MOL007061	SCN9A	Q15858

MOL007061	TERT	O14746
MOL007061	F2	P00734
MOL007061	TLR9	Q9NR96
MOL007061	PRSS1	P07477
MOL007061	KDR	P35968
MOL007061	CACNA1C	Q13936
MOL007061	KCNA3	P22001
MOL007061	SUZ12	Q15022
MOL007061	EZH2	Q15910
MOL007061	RBBP7	Q16576
MOL007063	NR3C1	P04150
MOL007063	NR3C2	P08235
MOL007064	HSD11B1	P28845
MOL007064	ACHE	P22303
MOL007064	LYPLA2	O95372
MOL007064	ADORA1	P30542
MOL007064	AR	P10275
MOL007064	BCL2L1	Q07817
MOL007064	BRS3	P32247
MOL007064	C5AR1	P21730
MOL007064	CASR	P41180
MOL007064	CAPN1	P07384
MOL007064	CSNK1G1	Q9HCP0
MOL007064	CASP1	P29466
MOL007064	CASP3	P42574
MOL007064	CASP6	P55212
MOL007064	CASP7	P55210
MOL007064	CASP8	Q14790
MOL007064	CTSG	P08311
MOL007064	CTSK	P43235
MOL007064	CTSS	P25774
MOL007064	MAPK8	P45983
MOL007064	MAPK10	P53779
MOL007064	CCNE1	P24864
MOL007064	CDK5R1	Q15078
MOL007064	PTGS2	P35354
MOL007064	CYP17A1	P05093
MOL007064	POLA1	P09884
MOL007064	POLB	P06746
MOL007064	SLC6A3	Q01959
MOL007064	DUSP3	P51452
MOL007064	FGFR1	P11362
MOL007064	FGFR3	P22607
MOL007064	PGGT1B	P53609

MOL007064	NR3C1	P04150
MOL007064	GSTM1	P09488
MOL007064	INSR	P06213
MOL007064	IGF1R	P08069
MOL007064	IL1B	P01584
MOL007064	IARS	P41252
MOL007064	RET	P07949
MOL007064	KIF11	P52732
MOL007064	ACP1	P24666
MOL007064	MAPK14	Q16539
MOL007064	MAPKAPK2	P49137
MOL007064	GRM5	P41594
MOL007064	NR3C2	P08235
MOL007064	CHRM3	P20309
MOL007064	MUSK	O15146
MOL007064	NTRK1	P04629
MOL007064	NCOA1	Q15788
MOL007064	NCOA2	Q15596
MOL007064	HCRTR1	O43613
MOL007064	HCRTR2	O43614
MOL007064	MDM2	Q00987
MOL007064	PER2	O15055
MOL007064	PDE4D	Q08499
MOL007064	PGR	P06401
MOL007064	FNTA	P49354
MOL007064	PRKCA	P17252
MOL007064	PRKCD	Q05655
MOL007064	PRKCE	Q02156
MOL007064	PTK2B	Q14289
MOL007064	VAV1	P15498
MOL007064	ATP2A1	O14983
MOL007064	AURKA	O14965
MOL007064	AURKB	Q96GD4
MOL007064	STAT3	P40763
MOL007064	SCN9A	Q15858
MOL007064	STS	P08842
MOL007064	PCSK7	Q16549
MOL007064	PTPN2	P17706
MOL007064	TERT	O14746
MOL007064	DNTT	P04053
MOL007064	TLR9	Q9NR96
MOL007064	TRPV4	Q9HBA0
MOL007064	TTL	Q8NG68
MOL007064	JAK2	O60674

MOL007064	JAK3	P52333
MOL007064	LCK	P06239
MOL007064	FLT3	P36888
MOL007064	SYK	P43405
MOL007064	CDK2	P24941
MOL007064	FNTB	P49356
MOL007064	CDK5	Q00535
MOL007068	DPP4	P27487
MOL007068	HSP90AB1	P08238
MOL007068	IGHG1	P01857
MOL007068	NCOA1	Q15788
MOL007068	PIK3CG	P48736
MOL007068	PTGS2	P35354
MOL007068	RXRA	P19793
MOL007068	F2	P00734
MOL007068	PRSS1	P07477
MOL007069	HSD11B1	P28845
MOL007069	NUDT1	P36639
MOL007069	ACHE	P22303
MOL007069	CES1	P23141
MOL007069	ADORA2A	P29274
MOL007069	ADORA2B	P29275
MOL007069	AKR1B1	P15121
MOL007069	ALPL	P05186
MOL007069	ADRA1A	P35348
MOL007069	FAAH	O00519
MOL007069	AR	P10275
MOL007069	ALOX5	P09917
MOL007069	ADRB2	P07550
MOL007069	CAPN1	P07384
MOL007069	CA2	P00918
MOL007069	CES2	O00748
MOL007069	CASP1	P29466
MOL007069	CASP7	P55210
MOL007069	CASP8	Q14790
MOL007069	CTSK	P43235
MOL007069	CDC7	O00311
MOL007069	CDK5R1	Q15078
MOL007069	PTGS1	P23219
MOL007069	CYP17A1	P05093
MOL007069	CYP2C19	P33261
MOL007069	CYP2C9	P11712
MOL007069	CYP3A4	P08684
MOL007069	OPRD1	P41143

MOL007069	DPP4	P27487
MOL007069	DRD1	P21728
MOL007069	DRD4	P21917
MOL007069	MAP2K1	Q02750
MOL007069	CDC25A	P30304
MOL007069	CDC25B	P30305
MOL007069	CDC25C	P30307
MOL007069	EED	O75530
MOL007069	RBBP4	Q09028
MOL007069	GABRA5	P31644
MOL007069	GABRA1	P14867
MOL007069	PSEN2	P49810
MOL007069	NR3C1	P04150
MOL007069	GSK3B	P49841
MOL007069	HSP90AB1	P08238
MOL007069	HMOX1	P09601
MOL007069	HMGCR	P04035
MOL007069	ICMT	O60725
MOL007069	JAK3	P52333
MOL007069	OPRK1	P41145
MOL007069	KIF11	P52732
MOL007069	PTPRC	P08575
MOL007069	PYGL	P06737
MOL007069	CSF1R	P07333
MOL007069	MPI	P34949
MOL007069	MAPK14	Q16539
MOL007069	CHRM1	P11229
MOL007069	CHRM2	P08172
MOL007069	CHRM3	P20309
MOL007069	CHRM4	P08173
MOL007069	CHRM5	P08912
MOL007069	OPRM1	P35372
MOL007069	CHRNA7	P36544
MOL007069	NCOA1	Q15788
MOL007069	P2RX7	Q99572
MOL007069	ABCB1	P08183
MOL007069	PIK3CG	P48736
MOL007069	PDE10A	Q9Y233
MOL007069	PDE2A	O00408
MOL007069	PDE7A	Q13946
MOL007069	PIK3CA	P42336
MOL007069	PIK3CB	P42338
MOL007069	PIK3CD	O00329
MOL007069	PARP1	P09874

MOL007069	PTGS2	P35354
MOL007069	PRMT3	O60678
MOL007069	PTPN6	P29350
MOL007069	PTPN11	Q06124
MOL007069	JUN	P05412
MOL007069	AURKA	O14965
MOL007069	PIM1	P11309
MOL007069	PIM2	Q9P1W9
MOL007069	HTR1D	P28221
MOL007069	HTR6	P50406
MOL007069	SCN5A	Q14524
MOL007069	ABCC9	O60706
MOL007069	TERT	O14746
MOL007069	F2	P00734
MOL007069	JAK1	P23458
MOL007069	JAK2	O60674
MOL007069	SUZ12	Q15022
MOL007069	EZH2	Q15910
MOL007069	CDK5	Q00535
MOL007069	RBBP7	Q16576
MOL007069	PSENEN	Q9NZ42
MOL007069	NCSTN	Q92542
MOL007069	APH1A	Q96BI3
MOL007069	PSEN1	P49768
MOL007069	APH1B	Q8WW43
MOL007070	ACHE	P22303
MOL007070	CA2	P00918
MOL007070	DPP4	P27487
MOL007070	HSP90AB1	P08238
MOL007070	NCOA1	Q15788
MOL007070	PTGS2	P35354
MOL007070	F2	P00734
MOL007070	PRSS1	P07477
MOL007071	DPP4	P27487
MOL007071	NCOA1	Q15788
MOL007071	PTGS2	P35354
MOL007071	F2	P00734
MOL007071	PRSS1	P07477
MOL007077	HSD11B1	P28845
MOL007077	ADA	P00813
MOL007077	AKR1C3	P42330
MOL007077	AR	P10275
MOL007077	ST6GAL1	P15907
MOL007077	C5AR1	P21730

MOL007077	CNR2	P34972
MOL007077	CA1	P00915
MOL007077	CA2	P00918
MOL007077	CA6	P23280
MOL007077	CCR1	P32246
MOL007077	CCR5	P51681
MOL007077	CDC7	O00311
MOL007077	MAPK8	P45983
MOL007077	MAPK9	P45984
MOL007077	MAPK10	P53779
MOL007077	CDK1	P06493
MOL007077	CCNB3	Q8WWL7
MOL007077	PTGS1	P23219
MOL007077	CYP11B1	P15538
MOL007077	CYP11B2	P19099
MOL007077	CYP17A1	P05093
MOL007077	CYP19A1	P11511
MOL007077	CYP2C9	P11712
MOL007077	CYP3A4	P08684
MOL007077	OPRD1	P41143
MOL007077	POLB	P06746
MOL007077	DRD2	P14416
MOL007077	DRD4	P21917
MOL007077	EGFR	P00533
MOL007077	EPHX2	P34913
MOL007077	HSD17B2	P37059
MOL007077	ESR1	P03372
MOL007077	ESR2	Q92731
MOL007077	GABRA2	P47869
MOL007077	GABBR2	O75899
MOL007077	PSEN2	P49810
MOL007077	PGGT1B	P53609
MOL007077	GCGR	P47871
MOL007077	GSK3B	P49841
MOL007077	LIPE	Q05469
MOL007077	SLC10A2	Q12908
MOL007077	IDO1	P14902
MOL007077	ICMT	O60725
MOL007077	OPRK1	P41145
MOL007077	PYGL	P06737
MOL007077	NR1H3	Q13133
MOL007077	MAPK1	P28482
MOL007077	MAPK14	Q16539
MOL007077	MMP1	P03956

MOL007077	MMP3	P08254
MOL007077	MMP9	P14780
MOL007077	MTNR1A	P48039
MOL007077	MTNR1B	P49286
MOL007077	NR3C2	P08235
MOL007077	MAP3K11	Q16584
MOL007077	MAP3K5	Q99683
MOL007077	MAOA	P21397
MOL007077	MAOB	P27338
MOL007077	OPRM1	P35372
MOL007077	CHRM1	P11229
MOL007077	CHRM2	P08172
MOL007077	CHRM3	P20309
MOL007077	TACR1	P25103
MOL007077	TACR2	P21452
MOL007077	NPY5R	Q15761
MOL007077	PER2	O15055
MOL007077	PDE10A	Q9Y233
MOL007077	PDE2A	O00408
MOL007077	PABPC1	P11940
MOL007077	PTGS2	P35354
MOL007077	PRMT3	O60678
MOL007077	PRKCA	P17252
MOL007077	PRKCB	P05771
MOL007077	PRKCD	Q05655
MOL007077	PRKCE	Q02156
MOL007077	PRKCG	P05129
MOL007077	PRKCQ	Q04759
MOL007077	F2R	P25116
MOL007077	PTPN1	P18031
MOL007077	RASGRP1	O95267
MOL007077	PIM1	P11309
MOL007077	PIM2	Q9P1W9
MOL007077	HTR1A	P08908
MOL007077	SLC6A4	P31645
MOL007077	SCN9A	Q15858
MOL007077	S1PR1	P21453
MOL007077	FDFT1	P37268
MOL007077	SRD5A1	P18405
MOL007077	SHBG	P04278
MOL007077	TRPA1	O75762
MOL007077	TTL	Q8NG68
MOL007077	JAK1	P23458
MOL007077	JAK2	O60674

MOL007077	JAK3	P52333
MOL007077	TYK2	P29597
MOL007077	UGT2B7	P16662
MOL007077	TRPV1	Q8NER1
MOL007077	KDR	P35968
MOL007077	AVPR1A	P37288
MOL007077	KCNA5	P22460
MOL007077	PSENEN	Q9NZ42
MOL007077	NCSTN	Q92542
MOL007077	APH1A	Q96BI3
MOL007077	PSEN1	P49768
MOL007077	APH1B	Q8WW43
MOL007077	GABBR1	Q9UBS5
MOL007077	CCNB1	P14635
MOL007077	CCNB2	O95067
MOL007077	GABRB2	P47870
MOL007077	GABRG2	P18507
MOL007079	HSD11B1	P28845
MOL007079	HTR2A	P28223
MOL007079	ACHE	P22303
MOL007079	CES1	P23141
MOL007079	ADAM10	O14672
MOL007079	ADAM17	P78536
MOL007079	AKR1B1	P15121
MOL007079	FAAH	O00519
MOL007079	NAT1	P18440
MOL007079	BCL2A1	Q16548
MOL007079	ADRB2	P07550
MOL007079	BDKRB1	P46663
MOL007079	BAZ2A	Q9UIF9
MOL007079	BAZ2B	Q9UIF8
MOL007079	BRD4	O60885
MOL007079	CAPN1	P07384
MOL007079	CES2	O00748
MOL007079	CASP3	P42574
MOL007079	CTSB	P07858
MOL007079	CTSV	O60911
MOL007079	CTSG	P08311
MOL007079	CTSK	P43235
MOL007079	CTSL	P07711
MOL007079	CTSS	P25774
MOL007079	CMA1	P23946
MOL007079	MAPK8	P45983
MOL007079	MAPK10	P53779

MOL007079	CREBBP	Q92793
MOL007079	CCNB3	Q8WWL7
MOL007079	CDK2	P24941
MOL007079	CCNE2	O96020
MOL007079	CDK5R1	Q15078
MOL007079	CDK9	P50750
MOL007079	PTGS2	P35354
MOL007079	OPRD1	P41143
MOL007079	DPP4	P27487
MOL007079	RAD51	Q06609
MOL007079	TOP2A	P11388
MOL007079	DRD1	P21728
MOL007079	DRD2	P14416
MOL007079	DRD4	P21917
MOL007079	MAP2K1	Q02750
MOL007079	CDC25B	P30305
MOL007079	DUSP1	P28562
MOL007079	EPHX2	P34913
MOL007079	FAP	Q12884
MOL007079	PSEN2	P49810
MOL007079	GSTP1	P09211
MOL007079	HSP90AA1	P07900
MOL007079	HRH3	Q9Y5N1
MOL007079	HRH4	Q9H3N8
MOL007079	HDAC1	Q13547
MOL007079	HDAC2	Q92769
MOL007079	HDAC3	O15379
MOL007079	HDAC6	Q9UBN7
MOL007079	HDAC8	Q9BY41
MOL007079	IDO1	P14902
MOL007079	ICAM1	P05362
MOL007079	LRRK2	Q5S007
MOL007079	PTPRC	P08575
MOL007079	ELANE	P08246
MOL007079	PYGL	P06737
MOL007079	KDM4E	B2RXH2
MOL007079	MAPK1	P28482
MOL007079	MAPK14	Q16539
MOL007079	MMP1	P03956
MOL007079	MMP14	P50281
MOL007079	MMP2	P08253
MOL007079	MMP9	P14780
MOL007079	MTNR1A	P48039
MOL007079	MTNR1B	P49286

MOL007079	GRM1	Q13255
MOL007079	GRM5	P41594
MOL007079	MALT1	Q9UDY8
MOL007079	CHRM1	P11229
MOL007079	CHRM2	P08172
MOL007079	OPRM1	P35372
MOL007079	SIRT2	Q8IXJ6
MOL007079	CHRNA7	P36544
MOL007079	NOS2	P35228
MOL007079	NOS1	P29475
MOL007079	NCOA1	Q15788
MOL007079	HCRTR1	O43613
MOL007079	HCRTR2	O43614
MOL007079	P2RX7	Q99572
MOL007079	PDE10A	Q9Y233
MOL007079	PDE4B	Q07343
MOL007079	PDE4D	Q08499
MOL007079	PDE7A	Q13946
MOL007079	PARP1	P09874
MOL007079	PREP	P48147
MOL007079	F2R	P25116
MOL007079	PTPN6	P29350
MOL007079	PTPN11	Q06124
MOL007079	WNT3	P56703
MOL007079	NQO2	P16083
MOL007079	RPS6KA3	P51812
MOL007079	SELE	P16581
MOL007079	HTR2C	P28335
MOL007079	HTR4	Q13639
MOL007079	STAT3	P40763
MOL007079	SCN9A	Q15858
MOL007079	TGFBR1	P36897
MOL007079	F2	P00734
MOL007079	F10	P00742
MOL007079	PRSS1	P07477
MOL007079	TDP2	O95551
MOL007079	KCNA5	P22460
MOL007079	CDK5	Q00535
MOL007079	PSENEN	Q9NZ42
MOL007079	NCSTN	Q92542
MOL007079	APH1A	Q96BI3
MOL007079	PSEN1	P49768
MOL007079	APH1B	Q8WW43
MOL007079	CCNE1	P24864

MOL007079	CDK1	P06493
MOL007079	CCNB1	P14635
MOL007079	CCNB2	O95067
MOL007079	CCNA1	P78396
MOL007079	CCNA2	P20248
MOL007081	HSD11B1	P28845
MOL007081	ACHE	P22303
MOL007081	CES1	P23141
MOL007081	ADORA1	P30542
MOL007081	ADORA2A	P29274
MOL007081	ADORA3	P0DMS8
MOL007081	ADRA2C	P18825
MOL007081	AKR1B1	P15121
MOL007081	ADRA1B	P35368
MOL007081	AMPD3	Q01432
MOL007081	AR	P10275
MOL007081	ALOX5	P09917
MOL007081	APP	P05067
MOL007081	BDKRB1	P46663
MOL007081	CAPN1	P07384
MOL007081	CNR1	P21554
MOL007081	CNR2	P34972
MOL007081	CA1	P00915
MOL007081	CA2	P00918
MOL007081	CA4	P22748
MOL007081	CA6	P23280
MOL007081	CES2	O00748
MOL007081	CASP1	P29466
MOL007081	CASP3	P42574
MOL007081	CASP6	P55212
MOL007081	CASP7	P55210
MOL007081	CASP8	Q14790
MOL007081	MAPK8	P45983
MOL007081	CYP11B1	P15538
MOL007081	CYP11B2	P19099
MOL007081	CYP17A1	P05093
MOL007081	CYP19A1	P11511
MOL007081	OPRD1	P41143
MOL007081	TOP2A	P11388
MOL007081	DRD4	P21917
MOL007081	SLC6A3	Q01959
MOL007081	DUT	P33316
MOL007081	HSD17B2	P37059
MOL007081	FKBP1A	P62942

MOL007081	PSEN2	P49810
MOL007081	NR3C1	P04150
MOL007081	HSP90AB1	P08238
MOL007081	MET	P08581
MOL007081	HRH2	P25021
MOL007081	IDO1	P14902
MOL007081	MCL1	Q07820
MOL007081	IL6ST	P40189
MOL007081	CXCR2	P25025
MOL007081	OPRK1	P41145
MOL007081	LRRK2	Q5S007
MOL007081	PYGL	P06737
MOL007081	KDM4E	B2RXH2
MOL007081	PRCP	P42785
MOL007081	MAPK14	Q16539
MOL007081	MMP1	P03956
MOL007081	MMP3	P08254
MOL007081	MMP9	P14780
MOL007081	GRM5	P41594
MOL007081	NR3C2	P08235
MOL007081	MAP3K14	Q99558
MOL007081	OPRM1	P35372
MOL007081	CHRM1	P11229
MOL007081	CHRM2	P08172
MOL007081	CHRM3	P20309
MOL007081	CHRM4	P08173
MOL007081	CHRM5	P08912
MOL007081	NPY5R	Q15761
MOL007081	OPRL1	P41146
MOL007081	SLC6A2	P23975
MOL007081	NCOA1	Q15788
MOL007081	NR4A1	P22736
MOL007081	HCRTR1	O43613
MOL007081	HCRTR2	O43614
MOL007081	MDM2	Q00987
MOL007081	PER2	O15055
MOL007081	ABCB1	P08183
MOL007081	PDE10A	Q9Y233
MOL007081	PDE2A	O00408
MOL007081	PDE9A	O76083
MOL007081	PARP1	P09874
MOL007081	PGR	P06401
MOL007081	PTGES	O14684
MOL007081	PTGS2	P35354

MOL007081	PTGER1	P34995
MOL007081	ACPP	P15309
MOL007081	PSMB5	P28074
MOL007081	FNTA	P49354
MOL007081	PTK2B	Q14289
MOL007081	PTPN6	P29350
MOL007081	PTPN11	Q06124
MOL007081	PPOX	P50336
MOL007081	QRFPR	Q96P65
MOL007081	HTR2A	P28223
MOL007081	HTR7	P34969
MOL007081	SLC6A4	P31645
MOL007081	STAT3	P40763
MOL007081	SMO	Q99835
MOL007081	SCN9A	Q15858
MOL007081	ABCC9	O60706
MOL007081	TERT	O14746
MOL007081	F2	P00734
MOL007081	TRPA1	O75762
MOL007081	KCNA5	P22460
MOL007081	KCNE1	P15382
MOL007081	PSENEN	Q9NZ42
MOL007081	NCSTN	Q92542
MOL007081	APH1A	Q96BI3
MOL007081	PSEN1	P49768
MOL007081	APH1B	Q8WW43
MOL007081	KCNQ1	P51787
MOL007081	FNTB	P49356
MOL007082	HSD11B1	P28845
MOL007082	PSMB1	P20618
MOL007082	ACHE	P22303
MOL007082	CES1	P23141
MOL007082	ADORA1	P30542
MOL007082	ADORA2A	P29274
MOL007082	ADORA2B	P29275
MOL007082	ADORA3	P0DMS8
MOL007082	ADRA2C	P18825
MOL007082	AKR1B1	P15121
MOL007082	ADRA1B	P35368
MOL007082	AOC3	Q16853
MOL007082	AMPD3	Q01432
MOL007082	AR	P10275
MOL007082	ALOX12	P18054
MOL007082	ALOX5	P09917

MOL007082	APP	P05067
MOL007082	BACE1	P56817
MOL007082	BRS3	P32247
MOL007082	BDKRB1	P46663
MOL007082	CASR	P41180
MOL007082	KCNMA1	Q12791
MOL007082	CAPN1	P07384
MOL007082	CES2	O00748
MOL007082	CASP1	P29466
MOL007082	CASP3	P42574
MOL007082	CASP6	P55212
MOL007082	CASP7	P55210
MOL007082	CASP8	Q14790
MOL007082	CCKAR	P32238
MOL007082	CCKBR	P32239
MOL007082	MAPK8	P45983
MOL007082	MAPK10	P53779
MOL007082	F10	P00742
MOL007082	CDK1	P06493
MOL007082	CDK2	P24941
MOL007082	CCNE2	O96020
MOL007082	CYP17A1	P05093
MOL007082	DRD4	P21917
MOL007082	SLC6A3	Q01959
MOL007082	EDNRA	P25101
MOL007082	EDNRB	P24530
MOL007082	EGFR	P00533
MOL007082	HSD17B2	P37059
MOL007082	EIF2AK3	Q9NZJ5
MOL007082	PSEN2	P49810
MOL007082	NR3C1	P04150
MOL007082	MET	P08581
MOL007082	HRH2	P25021
MOL007082	IDO1	P14902
MOL007082	MCL1	Q07820
MOL007082	CXCR2	P25025
MOL007082	JAK3	P52333
MOL007082	OPRK1	P41145
MOL007082	LRRK2	Q5S007
MOL007082	PYGL	P06737
MOL007082	KDM4E	B2RXH2
MOL007082	MAPK14	Q16539
MOL007082	GRM5	P41594
MOL007082	NR3C2	P08235

MOL007082	OPRM1	P35372
MOL007082	CHRM1	P11229
MOL007082	CHRM2	P08172
MOL007082	CHRM3	P20309
MOL007082	CHRM4	P08173
MOL007082	CHRM5	P08912
MOL007082	SLC6A2	P23975
MOL007082	NCOA1	Q15788
MOL007082	HCRTR1	O43613
MOL007082	HCRTR2	O43614
MOL007082	ABCB1	P08183
MOL007082	FARS2	O95363
MOL007082	PIK3CG	P48736
MOL007082	PDE10A	Q9Y233
MOL007082	PDE2A	O00408
MOL007082	PARP1	P09874
MOL007082	KCNH2	Q12809
MOL007082	PGR	P06401
MOL007082	PTGES	O14684
MOL007082	PTGS1	P23219
MOL007082	PTGS2	P35354
MOL007082	ACPP	P15309
MOL007082	PSMB2	P49721
MOL007082	PSMB5	P28074
MOL007082	FNTA	P49354
MOL007082	PRKCA	P17252
MOL007082	PTPN6	P29350
MOL007082	PTPN11	Q06124
MOL007082	PPOX	P50336
MOL007082	RXRA	P19793
MOL007082	AURKA	O14965
MOL007082	BRAF	P15056
MOL007082	PIM1	P11309
MOL007082	HTR2A	P28223
MOL007082	HTR7	P34969
MOL007082	SLC6A4	P31645
MOL007082	STAT3	P40763
MOL007082	SCN5A	Q14524
MOL007082	SCN9A	Q15858
MOL007082	ABCC9	O60706
MOL007082	TERT	O14746
MOL007082	TNNC1	P63316
MOL007082	JAK1	P23458
MOL007082	JAK2	O60674

MOL007082	SYK	P43405
MOL007082	KDR	P35968
MOL007082	KCNE1	P15382
MOL007082	PSENEN	Q9NZ42
MOL007082	NCSTN	Q92542
MOL007082	APH1A	Q96BI3
MOL007082	PSEN1	P49768
MOL007082	APH1B	Q8WW43
MOL007082	KCNQ1	P51787
MOL007082	TNNT2	P45379
MOL007082	TNNI3	P19429
MOL007082	FNTB	P49356
MOL007082	CCNA1	P78396
MOL007082	CCNA2	P20248
MOL007082	CCNE1	P24864
MOL007085	HTR3A,	P46098
MOL007085	AR	P10275
MOL007085	CTSB	P07858
MOL007085	CTSK	P43235
MOL007085	CTSL	P07711
MOL007085	PTGS1	P23219
MOL007085	PTGS2	P35354
MOL007085	SLC6A3	Q01959
MOL007085	EPHX2	P34913
MOL007085	EPHX1	P07099
MOL007085	ESR1	P03372
MOL007085	ESR2	Q92731
MOL007085	GABRB3	P28472
MOL007085	GABRA2	P47869
MOL007085	GABRG2	P18507
MOL007085	GRM1	Q13255
MOL007085	CHRM1	P11229
MOL007085	CHRM2	P08172
MOL007085	CHRM5	P08912
MOL007085	HCRTR2	O43614
MOL007085	P2RX7	Q99572
MOL007085	PABPC1	P11940
MOL007085	PREP	P48147
MOL007085	PIM1	P11309
MOL007085	HTR2B	P41595
MOL007085	TRPM8	Q7Z2W7
MOL007085	GABRA3	P34903
MOL007085	GABRA1	P14867
MOL007085	GABRA5	P31644

MOL007085	GABRA6	Q16445
MOL007088	ACHE	P22303
MOL007088	CES1	P23141
MOL007088	ADORA1	P30542
MOL007088	ADORA2A	P29274
MOL007088	ADORA2B	P29275
MOL007088	ADORA3	P0DMS8
MOL007088	AKR1B1	P15121
MOL007088	ADRA1A	P35348
MOL007088	ADRA1B	P35368
MOL007088	ADRA1D	P25100
MOL007088	ADRA2A	P08913
MOL007088	APP	P05067
MOL007088	FAAH	O00519
MOL007088	BIRC5	O15392
MOL007088	BCL2L1	Q07817
MOL007088	ADRB2	P07550
MOL007088	CNR2	P34972
MOL007088	CA2	P00918
MOL007088	CES2	O00748
MOL007088	CTSK	P43235
MOL007088	CTSL	P07711
MOL007088	MAPK10	P53779
MOL007088	F13A1	P00488
MOL007088	CDK5R1	Q15078
MOL007088	PTGS1	P23219
MOL007088	PTGS2	P35354
MOL007088	CYP17A1	P05093
MOL007088	CYP19A1	P11511
MOL007088	OPRD1	P41143
MOL007088	CTSC	P53634
MOL007088	TOP1	P11387
MOL007088	TOP2A	P11388
MOL007088	DRD1	P21728
MOL007088	CDC25B	P30305
MOL007088	DUSP1	P28562
MOL007088	DUSP3	P51452
MOL007088	EDN1	P05305
MOL007088	EGFR	P00533
MOL007088	EPHX2	P34913
MOL007088	FAP	Q12884
MOL007088	PTK2	Q05397
MOL007088	CCND1	P24385
MOL007088	GABRA1	P14867

MOL007088	GSK3A	P49840
MOL007088	GSK3B	P49841
MOL007088	LIPE	Q05469
MOL007088	IDO1	P14902
MOL007088	ICAM1	P05362
MOL007088	KIF11	P52732
MOL007088	PTPRC	P08575
MOL007088	KDM4C	Q9H3R0
MOL007088	KDM4E	B2RXH2
MOL007088	CSF1R	P07333
MOL007088	MTNR1A	P48039
MOL007088	MTNR1B	P49286
MOL007088	GRM5	P41594
MOL007088	MALT1	Q9UDY8
MOL007088	CHRM1	P11229
MOL007088	CHRM2	P08172
MOL007088	CHRM3	P20309
MOL007088	CHRM4	P08173
MOL007088	CHRM5	P08912
MOL007088	OPRM1	P35372
MOL007088	NAAA	Q02083
MOL007088	SIRT2	Q8IXJ6
MOL007088	CHRNA7	P36544
MOL007088	NPY5R	Q15761
MOL007088	NCOA1	Q15788
MOL007088	NCOA2	Q15596
MOL007088	PDE4B	Q07343
MOL007088	PDE5A	O76074
MOL007088	PDE7A	Q13946
MOL007088	PIK3CA	P42336
MOL007088	PARP1	P09874
MOL007088	PGR	P06401
MOL007088	TGM2	P21980
MOL007088	PTPN1	P18031
MOL007088	PTPN6	P29350
MOL007088	PTPN11	Q06124
MOL007088	SELE	P16581
MOL007088	HTR2A	P28223
MOL007088	HTR2C	P28335
MOL007088	STAT3	P40763
MOL007088	SCN5A	Q14524
MOL007088	SCN9A	Q15858
MOL007088	TNKS2	Q9H2K2
MOL007088	TLR9	Q9NR96

MOL007088	RELA	Q04206
MOL007088	TNF	P01375
MOL007088	TYK2	P29597
MOL007088	TDP2	O95551
MOL007088	KDR	P35968
MOL007088	CDK5	Q00535
MOL007093	HSD11B1	P28845
MOL007093	ACHE	P22303
MOL007093	ADORA1	P30542
MOL007093	ADORA2A	P29274
MOL007093	ADORA2B	P29275
MOL007093	ADORA3	P0DMS8
MOL007093	ALPL	P05186
MOL007093	ADRA1B	P35368
MOL007093	AMPD3	Q01432
MOL007093	FAAH	O00519
MOL007093	AR	P10275
MOL007093	ADRB2	P07550
MOL007093	BACE1	P56817
MOL007093	BDKRB1	P46663
MOL007093	ADCY1	Q08828
MOL007093	BRD2	P25440
MOL007093	BRD3	Q15059
MOL007093	BRD4	O60885
MOL007093	CALM1	P62158
MOL007093	CNR1	P21554
MOL007093	CNR2	P34972
MOL007093	CA2	P00918
MOL007093	CASP3	P42574
MOL007093	CASP7	P55210
MOL007093	CTSD	P07339
MOL007093	CTSK	P43235
MOL007093	CTSS	P25774
MOL007093	CCR1	P32246
MOL007093	CDK2	P24941
MOL007093	F10	P00742
MOL007093	F11	P03951
MOL007093	CFD	P00746
MOL007093	CRHR1	P34998
MOL007093	CX3CR1	P49238
MOL007093	CCNA2	P20248
MOL007093	CDK1	P06493
MOL007093	CCNE2	O96020
MOL007093	CCND1	P24385

MOL007093	CDK5R1	Q15078
MOL007093	CDK5	Q00535
MOL007093	PTGS2	P35354
MOL007093	DPP4	P27487
MOL007093	TOP2A	P11388
MOL007093	PRKDC	P78527
MOL007093	DRD2	P14416
MOL007093	MAP2K1	Q02750
MOL007093	DYRK1A	Q13627
MOL007093	EGFR	P00533
MOL007093	ESR1	P03372
MOL007093	ESR2	Q92731
MOL007093	FAP	Q12884
MOL007093	FKBP1A	P62942
MOL007093	GABRA5	P31644
MOL007093	PSEN2	P49810
MOL007093	GRIN2A	Q12879
MOL007093	GRIA2	P42262
MOL007093	GSK3A	P49840
MOL007093	GSK3B	P49841
MOL007093	HSP90AB1	P08238
MOL007093	HMGCR	P04035
MOL007093	IGHG1	P01857
MOL007093	IDO1	P14902
MOL007093	CXCR2	P25025
MOL007093	KIF11	P52732
MOL007093	LRRK2	Q5S007
MOL007093	ELANE	P08246
MOL007093	PYGL	P06737
MOL007093	MAPK14	Q16539
MOL007093	MTNR1A	P48039
MOL007093	MTNR1B	P49286
MOL007093	GRM5	P41594
MOL007093	MAP3K5	Q99683
MOL007093	CHRM1	P11229
MOL007093	DDAH1	O94760
MOL007093	NTRK1	P04629
MOL007093	NOS3	P29474
MOL007093	NCOA1	Q15788
MOL007093	NCOA2	Q15596
MOL007093	P2RX3	P56373
MOL007093	P2RX7	Q99572
MOL007093	PIN1	Q13526
MOL007093	PPARG	P37231

MOL007093	PDE10A	Q9Y233
MOL007093	PDE2A	O00408
MOL007093	PDE4A	P27815
MOL007093	PDE7A	Q13946
MOL007093	PDE9A	O76083
MOL007093	PIK3CB	P42338
MOL007093	PIK3CD	O00329
MOL007093	PIK3CG	P48736
MOL007093	PARP1	P09874
MOL007093	KCNK3	O14649
MOL007093	KCNH2	Q12809
MOL007093	GPR139	Q6DWJ6
MOL007093	PREP	P48147
MOL007093	PTGS1	P23219
MOL007093	PRKCA	P17252
MOL007093	PRKCD	Q05655
MOL007093	PIM1	P11309
MOL007093	MERTK	Q12866
MOL007093	ERBB2	P04626
MOL007093	REN	P00797
MOL007093	RXRA	P19793
MOL007093	CHEK1	O14757
MOL007093	PIM2	Q9P1W9
MOL007093	HTR1A	P08908
MOL007093	HTR2A	P28223
MOL007093	HTR6	P50406
MOL007093	SCN5A	Q14524
MOL007093	SORD	Q00796
MOL007093	ABCC9	O60706
MOL007093	F2	P00734
MOL007093	TK2	O00142
MOL007093	PLAT	P00750
MOL007093	TSPO	P30536
MOL007093	PRSS1	P07477
MOL007093	TUBB1	Q9H4B7
MOL007093	JAK1	P23458
MOL007093	JAK2	O60674
MOL007093	JAK3	P52333
MOL007093	SYK	P43405
MOL007093	CCNE1	P24864
MOL007093	CDK4	P11802
MOL007093	CCNA1	P78396
MOL007093	PSENEN	Q9NZ42
MOL007093	NCSTN	Q92542

MOL007093	APH1A	Q96BI3
MOL007093	PSEN1	P49768
MOL007093	APH1B	Q8WW43
MOL007093	GRIN1	Q05586
MOL007094	ACHE	P22303
MOL007094	SOAT1	P35610
MOL007094	ADORA1	P30542
MOL007094	ADORA2A	P29274
MOL007094	ADORA2B	P29275
MOL007094	ADRA1A	P35348
MOL007094	ADRA1B	P35368
MOL007094	ADRA1D	P25100
MOL007094	ALKBH3	Q96Q83
MOL007094	ADRB2	P07550
MOL007094	CAPN1	P07384
MOL007094	CAPN2	P17655
MOL007094	CA2	P00918
MOL007094	CTSB	P07858
MOL007094	CTSK	P43235
MOL007094	CTSL	P07711
MOL007094	CTSS	P25774
MOL007094	CCNB3	Q8WWL7
MOL007094	CCNE2	O96020
MOL007094	PTGS2	P35354
MOL007094	DPP4	P27487
MOL007094	DRD1	P21728
MOL007094	EPHB4	P54760
MOL007094	ESR1	P03372
MOL007094	GABRA1	P14867
MOL007094	HSP90AB1	P08238
MOL007094	HMOX1	P09601
MOL007094	MCL1	Q07820
MOL007094	IGF1R	P08069
MOL007094	ELANE	P08246
MOL007094	MAPK14	Q16539
MOL007094	NR3C2	P08235
MOL007094	MAOA	P21397
MOL007094	MAOB	P27338
MOL007094	CHRM1	P11229
MOL007094	CHRM2	P08172
MOL007094	CHRM3	P20309
MOL007094	CHRM4	P08173
MOL007094	CHRM5	P08912
MOL007094	OPRM1	P35372

MOL007094	NTRK1	P04629
MOL007094	CHRNA7	P36544
MOL007094	CHRNA2	Q15822
MOL007094	NR4A1	P22736
MOL007094	MDM2	Q00987
MOL007094	PTGS1	P23219
MOL007094	PTK2B	Q14289
MOL007094	RXRA	P19793
MOL007094	PAK1	Q13153
MOL007094	PAK4	O96013
MOL007094	RIPK2	O43353
MOL007094	SCN5A	Q14524
MOL007094	SLC6A4	P31645
MOL007094	F2	P00734
MOL007094	KCNA3	P22001
MOL007094	CDK2	P24941
MOL007094	CCNE1	P24864
MOL007094	CDK1	P06493
MOL007094	CCNB1	P14635
MOL007094	CCNB2	O95067
MOL007098	ACHE	P22303
MOL007098	CES1	P23141
MOL007098	AKR1B10	O60218
MOL007098	AKR1B1	P15121
MOL007098	ADRA1A	P35348
MOL007098	ADRA1B	P35368
MOL007098	ADRA1D	P25100
MOL007098	AR	P10275
MOL007098	BCL2	P10415
MOL007098	BCL2L1	Q07817
MOL007098	ADRB2	P07550
MOL007098	CA2	P00918
MOL007098	CES2	O00748
MOL007098	CCR9	P51686
MOL007098	CDK2	P24941
MOL007098	OPRD1	P41143
MOL007098	DHODH	Q02127
MOL007098	TOP1	P11387
MOL007098	TOP2A	P11388
MOL007098	DRD1	P21728
MOL007098	CDC25A	P30304
MOL007098	CDC25B	P30305
MOL007098	DUSP1	P28562
MOL007098	ESR1	P03372

MOL007098	FABP4	P15090
MOL007098	FABP3	P05413
MOL007098	PTGDR2	Q9Y5Y4
MOL007098	GSK3B	P49841
MOL007098	IGHG1	P01857
MOL007098	IDO1	P14902
MOL007098	MCL1	Q07820
MOL007098	PTPRC	P08575
MOL007098	KDM4E	B2RXH2
MOL007098	MALT1	Q9UDY8
MOL007098	CHRM1	P11229
MOL007098	CHRM2	P08172
MOL007098	CHRM3	P20309
MOL007098	CHRM4	P08173
MOL007098	CHRM5	P08912
MOL007098	OPRM1	P35372
MOL007098	CHRNA7	P36544
MOL007098	NOS1	P29475
MOL007098	NCOA1	Q15788
MOL007098	NCOA2	Q15596
MOL007098	MDM2	Q00987
MOL007098	PPARG	P37231
MOL007098	PTGS1	P23219
MOL007098	PTGS2	P35354
MOL007098	PTGER1	P34995
MOL007098	PTGER3	P43115
MOL007098	PTGER4	P35408
MOL007098	PTPN1	P18031
MOL007098	PTPN6	P29350
MOL007098	PIM1	P11309
MOL007098	PTPRF	P10586
MOL007098	RXRA	P19793
MOL007098	STAT3	P40763
MOL007098	SCN5A	Q14524
MOL007098	TDP2	O95551
MOL007100	HTR2A	P28223
MOL007100	HTR3A,	P46098
MOL007100	ACHE	P22303
MOL007100	ADRA1A	P35348
MOL007100	ADRA1B	P35368
MOL007100	ADRA1D	P25100
MOL007100	AR	P10275
MOL007100	ADRB2	P07550
MOL007100	CA2	P00918

MOL007100	PDE3A	Q14432
MOL007100	CCNA2	P20248
MOL007100	DPP4	P27487
MOL007100	DRD1	P21728
MOL007100	ESR1	P03372
MOL007100	GABRA1	P14867
MOL007100	GSK3B	P49841
MOL007100	IGHG1	P01857
MOL007100	PRKACA	P17612
MOL007100	PTPN1	P18031
MOL007100	CHRM1	P11229
MOL007100	CHRM3	P20309
MOL007100	CHRM5	P08912
MOL007100	OPRM1	P35372
MOL007100	CHRNA7	P36544
MOL007100	NOS2	P35228
MOL007100	PPARG	P37231
MOL007100	PIK3CG	P48736
MOL007100	PTGS1	P23219
MOL007100	PTGS2	P35354
MOL007100	PIM1	P11309
MOL007100	RXRA	P19793
MOL007100	SCN5A	Q14524
MOL007100	SLC6A3	Q01959
MOL007100	SLC6A4	P31645
MOL007100	F2	P00734
MOL007100	PRSS1	P07477
MOL007101	HTR3A,	P46098
MOL007101	ACHE	P22303
MOL007101	CES1	P23141
MOL007101	ADAM17	P78536
MOL007101	ADORA1	P30542
MOL007101	ADORA2A	P29274
MOL007101	ADORA2B	P29275
MOL007101	ADORA3	P0DMS8
MOL007101	ALDH2	P05091
MOL007101	ALDH1A1	P00352
MOL007101	ALDH3A1	P30838
MOL007101	AKR1B1	P15121
MOL007101	ALPL	P05186
MOL007101	ADRA1A	P35348
MOL007101	ADRA1B	P35368
MOL007101	ALOX15	P16050
MOL007101	ADRB2	P07550

MOL007101	CALM1	P62158
MOL007101	CA9	Q16790
MOL007101	CA12	O43570
MOL007101	CES2	O00748
MOL007101	CASP3	P42574
MOL007101	CASP7	P55210
MOL007101	MAPK8	P45983
MOL007101	MAPK10	P53779
MOL007101	CCNE2	O96020
MOL007101	CCNE1	P24864
MOL007101	CDK5R1	Q15078
MOL007101	CDK5	Q00535
MOL007101	TOP2A	P11388
MOL007101	CDC25B	P30305
MOL007101	DUSP1	P28562
MOL007101	EGFR	P00533
MOL007101	GABRA2	P47869
MOL007101	GABRB3	P28472
MOL007101	GABRA1	P14867
MOL007101	HSP90AB1	P08238
MOL007101	GCK	P35557
MOL007101	HRH3	Q9Y5N1
MOL007101	IGHG1	P01857
MOL007101	IDO1	P14902
MOL007101	PTPRC	P08575
MOL007101	ELANE	P08246
MOL007101	KDM4E	B2RXH2
MOL007101	GRM5	P41594
MOL007101	PRKACA	P17612
MOL007101	MALT1	Q9UDY8
MOL007101	CHRM1	P11229
MOL007101	CHRM2	P08172
MOL007101	CHRM4	P08173
MOL007101	CHRM5	P08912
MOL007101	NAAA	Q02083
MOL007101	SIRT2	Q8IXJ6
MOL007101	CHRNA7	P36544
MOL007101	NCOA1	Q15788
MOL007101	NCOA2	Q15596
MOL007101	PIK3CG	P48736
MOL007101	PTGS1	P23219
MOL007101	PTGS2	P35354
MOL007101	TGM2	P21980
MOL007101	PTPN1	P18031

MOL007101	PTPN6	P29350
MOL007101	PTPN11	Q06124
MOL007101	ERBB2	P04626
MOL007101	RXRA	P19793
MOL007101	HTR1A	P08908
MOL007101	HTR2B	P41595
MOL007101	HTR2C	P28335
MOL007101	HTR5A	P47898
MOL007101	HTR7	P34969
MOL007101	STAT3	P40763
MOL007101	SCN5A	Q14524
MOL007101	TDP2	O95551
MOL007101	PLAUR	Q03405
MOL007101	KDR	P35968
MOL007101	GABRG2	P18507
MOL007101	GABRA5	P31644
MOL007101	CDK2	P24941
MOL007101	GABRA3	P34903
MOL007101	GABRB2	P47870
MOL007105	HTR2A	P28223
MOL007105	ADORA1	P30542
MOL007105	ADORA2A	P29274
MOL007105	ADRA1A	P35348
MOL007105	ADRA1B	P35368
MOL007105	ADRA1D	P25100
MOL007105	ALKBH3	Q96Q83
MOL007105	ALOX15	P16050
MOL007105	ADRB2	P07550
MOL007105	BRD4	O60885
MOL007105	CTSK	P43235
MOL007105	CDK2	P24941
MOL007105	PDE3A	Q14432
MOL007105	CCKBR	P32239
MOL007105	CREBBP	Q92793
MOL007105	CDK1	P06493
MOL007105	CCNE2	O96020
MOL007105	CCND1	P24385
MOL007105	CYP51A1	Q16850
MOL007105	OPRD1	P41143
MOL007105	DRD1	P21728
MOL007105	SLC6A3	Q01959
MOL007105	EGFR	P00533
MOL007105	ESR1	P03372
MOL007105	FADS1	O60427

MOL007105	GABRB3	P28472
MOL007105	GABRA1	P14867
MOL007105	GSK3A	P49840
MOL007105	GSK3B	P49841
MOL007105	HSP90AB1	P08238
MOL007105	HMOX1	P09601
MOL007105	HIF1A	Q16665
MOL007105	IMPDH2	P12268
MOL007105	LTA4H	P09960
MOL007105	MAPK1	P28482
MOL007105	MTNR1A	P48039
MOL007105	MTNR1B	P49286
MOL007105	GRM5	P41594
MOL007105	CHRM1	P11229
MOL007105	CHRM2	P08172
MOL007105	CHRM3	P20309
MOL007105	CHRM4	P08173
MOL007105	CHRM5	P08912
MOL007105	OPRM1	P35372
MOL007105	CHRNA7	P36544
MOL007105	NOS1	P29475
MOL007105	HCRTR1	O43613
MOL007105	HCRTR2	O43614
MOL007105	MDM2	Q00987
MOL007105	PDGFRB	P09619
MOL007105	KCNK2	O95069
MOL007105	KCNK3	O14649
MOL007105	KCNK9	Q9NPC2
MOL007105	PGR	P06401
MOL007105	PTGS1	P23219
MOL007105	PTGS2	P35354
MOL007105	PIM1	P11309
MOL007105	RXRA	P19793
MOL007105	RIPK2	O43353
MOL007105	SCN5A	Q14524
MOL007105	SLC6A4	P31645
MOL007105	SRC	P12931
MOL007105	FLT1	P17948
MOL007105	KDR	P35968
MOL007105	VDR	P11473
MOL007105	KCNA3	P22001
MOL007105	GABRG2	P18507
MOL007105	GABRA5	P31644
MOL007105	CDK4	P11802

MOL007105	CCNB1	P14635
MOL007105	CCNA1	P78396
MOL007105	CCNA2	P20248
MOL007105	CCNE1	P24864
MOL007107	ACHE	P22303
MOL007107	ADCY10	Q96PN6
MOL007107	ALK	Q9UM73
MOL007107	ADRA1A	P35348
MOL007107	ADRA1B	P35368
MOL007107	ADRA1D	P25100
MOL007107	AR	P10275
MOL007107	ALOX12	P18054
MOL007107	ALOX15	P16050
MOL007107	ALOX5	P09917
MOL007107	ADRB2	P07550
MOL007107	CNR1	P21554
MOL007107	CNR2	P34972
MOL007107	CA2	P00918
MOL007107	PTGS2	P35354
MOL007107	CYP19A1	P11511
MOL007107	CYP24A1	Q07973
MOL007107	OPRD1	P41143
MOL007107	POLA1	P09884
MOL007107	POLB	P06746
MOL007107	SLC6A3	Q01959
MOL007107	HSD17B1	P14061
MOL007107	ESR1	P03372
MOL007107	ESR2	Q92731
MOL007107	ESRRA	P11474
MOL007107	ESRRB	O95718
MOL007107	NR3C1	P04150
MOL007107	GPER1	Q99527
MOL007107	GPR55	Q9Y2T6
MOL007107	IGF1R	P08069
MOL007107	OPRK1	P41145
MOL007107	LTB4R	Q15722
MOL007107	NR1H3	Q13133
MOL007107	NR1H2	P55055
MOL007107	MC4R	P32245
MOL007107	OPRM1	P35372
MOL007107	CHRM1	P11229
MOL007107	CHRM2	P08172
MOL007107	CHRM3	P20309
MOL007107	SIRT2	Q8IXJ6

MOL007107	NR1I3	Q14994
MOL007107	PGR	P06401
MOL007107	RXRA	P19793
MOL007107	HTR1A	P08908
MOL007107	HTR1B	P28222
MOL007107	HTR2B	P41595
MOL007107	HTR6	P50406
MOL007107	SLC6A4	P31645
MOL007107	SCN5A	Q14524
MOL007107	SLC22A2	O15244
MOL007107	STS	P08842
MOL007107	SHBG	P04278
MOL007107	F2	P00734
MOL007107	TTR	P02766
MOL007107	FYN	P06241
MOL007107	SRC	P12931
MOL007107	TRPV1	Q8NER1
MOL007107	KDR	P35968
MOL007108	HSD11B1	P28845
MOL007108	ACHE	P22303
MOL007108	AKR1B1	P15121
MOL007108	ADRA1A	P35348
MOL007108	ADRA1B	P35368
MOL007108	ADRA1D	P25100
MOL007108	FAAH	O00519
MOL007108	AR	P10275
MOL007108	ABCG2	Q9UNQ0
MOL007108	ADRB2	P07550
MOL007108	CNR2	P34972
MOL007108	CA2	P00918
MOL007108	CTSK	P43235
MOL007108	CDK2	P24941
MOL007108	F10	P00742
MOL007108	PTGS1	P23219
MOL007108	PTGS2	P35354
MOL007108	DRD2	P14416
MOL007108	OPRD1	P41143
MOL007108	TOP2A	P11388
MOL007108	DRD1	P21728
MOL007108	SLC6A3	Q01959
MOL007108	EPHX2	P34913
MOL007108	ESR1	P03372
MOL007108	FAP	Q12884
MOL007108	GABRA1	P14867

MOL007108	IDO1	P14902
MOL007108	GRM5	P41594
MOL007108	CHRM1	P11229
MOL007108	CHRM2	P08172
MOL007108	CHRM3	P20309
MOL007108	CHRM4	P08173
MOL007108	CHRM5	P08912
MOL007108	OPRM1	P35372
MOL007108	SIRT2	Q8IXJ6
MOL007108	CHRNA7	P36544
MOL007108	NOS3	P29474
MOL007108	NCOA1	Q15788
MOL007108	NCOA2	Q15596
MOL007108	P2RX7	Q99572
MOL007108	PARP1	P09874
MOL007108	PGR	P06401
MOL007108	PREP	P48147
MOL007108	PSMB5	P28074
MOL007108	PIM1	P11309
MOL007108	RXRA	P19793
MOL007108	SCN5A	Q14524
MOL007108	S1PR3	Q99500
MOL007108	PRSS1	P07477
MOL007111	HSD11B1	P28845
MOL007111	ACHE	P22303
MOL007111	CES1	P23141
MOL007111	ADORA1	P30542
MOL007111	ADORA2A	P29274
MOL007111	ADORA2B	P29275
MOL007111	ADORA3	P0DMS8
MOL007111	AKR1B1	P15121
MOL007111	ADRA1A	P35348
MOL007111	AR	P10275
MOL007111	NAT1	P18440
MOL007111	ADRB2	P07550
MOL007111	CA1	P00915
MOL007111	CA2	P00918
MOL007111	CA9	Q16790
MOL007111	CA12	O43570
MOL007111	CES2	O00748
MOL007111	CDK2	P24941
MOL007111	CREBBP	Q92793
MOL007111	CCNA2	P20248
MOL007111	CCNB3	Q8WWL7

MOL007111	CCNE2	O96020
MOL007111	CYP19A1	P11511
MOL007111	OPRD1	P41143
MOL007111	DPP4	P27487
MOL007111	DRD1	P21728
MOL007111	CDC25A	P30304
MOL007111	CDC25B	P30305
MOL007111	CDC25C	P30307
MOL007111	EPHB4	P54760
MOL007111	EGFR	P00533
MOL007111	EPHX1	P07099
MOL007111	ESR1	P03372
MOL007111	ESR2	Q92731
MOL007111	EED	O75530
MOL007111	RBBP4	Q09028
MOL007111	GABRA5	P31644
MOL007111	GABRA1	P14867
MOL007111	GSK3B	P49841
MOL007111	IMPDH2	P12268
MOL007111	CXCR2	P25025
MOL007111	PTPRC	P08575
MOL007111	MPI	P34949
MOL007111	MTNR1A	P48039
MOL007111	MTNR1B	P49286
MOL007111	MAOA	P21397
MOL007111	MAOB	P27338
MOL007111	CHRM1	P11229
MOL007111	CHRM2	P08172
MOL007111	CHRM3	P20309
MOL007111	CHRM5	P08912
MOL007111	OPRM1	P35372
MOL007111	SIRT2	Q8IXJ6
MOL007111	CHRNA7	P36544
MOL007111	NAMPT	P43490
MOL007111	NOS2	P35228
MOL007111	HCRTR1	O43613
MOL007111	HCRTR2	O43614
MOL007111	P2RX7	Q99572
MOL007111	PTAFR	P25105
MOL007111	PTGS2	P35354
MOL007111	PTPN6	P29350
MOL007111	PTPN11	Q06124
MOL007111	PIM1	P11309
MOL007111	RXRA	P19793

MOL007111	CHEK1	O14757
MOL007111	SCN5A	Q14524
MOL007111	TERT	O14746
MOL007111	F2	P00734
MOL007111	KDR	P35968
MOL007111	CACNA1C	Q13936
MOL007111	KCNA3	P22001
MOL007111	SUZ12	Q15022
MOL007111	EZH2	Q15910
MOL007111	RBBP7	Q16576
MOL007111	CCNE1	P24864
MOL007111	CDK1	P06493
MOL007111	CCNB1	P14635
MOL007111	CCNB2	O95067
MOL007115	HSD11B1	P28845
MOL007115	ACHE	P22303
MOL007115	AKR1B10	O60218
MOL007115	AR	P10275
MOL007115	BCHE	P06276
MOL007115	CNR2	P34972
MOL007115	PTGS1	P23219
MOL007115	CYP17A1	P05093
MOL007115	CYP19A1	P11511
MOL007115	CYP2C19	P33261
MOL007115	POLB	P06746
MOL007115	CDC25A	P30304
MOL007115	CDC25B	P30305
MOL007115	ESR1	P03372
MOL007115	ESR2	Q92731
MOL007115	PSEN2	P49810
MOL007115	GCGR	P47871
MOL007115	SLC10A2	Q12908
MOL007115	NR1H3	Q13133
MOL007115	GRM5	P41594
MOL007115	CHRM1	P11229
MOL007115	CHRM2	P08172
MOL007115	CHRM3	P20309
MOL007115	CHRM4	P08173
MOL007115	CHRM5	P08912
MOL007115	SLC6A2	P23975
MOL007115	NCOA2	Q15596
MOL007115	NR1I3	Q14994
MOL007115	OXTR	P30559
MOL007115	PER2	O15055

MOL007115	PRKCD	Q05655
MOL007115	PTPN1	P18031
MOL007115	SLC6A4	P31645
MOL007115	SAE1	Q9UBE0
MOL007115	SHBG	P04278
MOL007115	UGT2B7	P16662
MOL007115	TRPV1	Q8NER1
MOL007115	PSENEN	Q9NZ42
MOL007115	UBA2	Q9UBT2
MOL007115	NCSTN	Q92542
MOL007115	APH1A	Q96BI3
MOL007115	PSEN1	P49768
MOL007115	APH1B	Q8WW43
MOL007118	CES1	P23141
MOL007118	AKR1B1	P15121
MOL007118	CASR	P41180
MOL007118	CES2	O00748
MOL007118	CYP17A1	P05093
MOL007118	TOP1	P11387
MOL007118	CDC25A	P30304
MOL007118	EGFR	P00533
MOL007118	HSD17B2	P37059
MOL007118	EIF2AK1	Q9BQI3
MOL007118	PSEN2	P49810
MOL007118	HRH3	Q9Y5N1
MOL007118	SLC10A2	Q12908
MOL007118	IGF1R	P08069
MOL007118	NTRK1	P04629
MOL007118	TACR1	P25103
MOL007118	NPY5R	Q15761
MOL007118	SLC6A2	P23975
MOL007118	HCRTR1	O43613
MOL007118	HCRTR2	O43614
MOL007118	P2RX7	Q99572
MOL007118	MDM2	Q00987
MOL007118	PER2	O15055
MOL007118	PARP1	P09874
MOL007118	PABPC1	P11940
MOL007118	PTK2B	Q14289
MOL007118	AURKA	O14965
MOL007118	AURKB	Q96GD4
MOL007118	PIM1	P11309
MOL007118	PIM2	Q9P1W9
MOL007118	JAK2	O60674

MOL007118	SRC	P12931
MOL007118	UGT2B7	P16662
MOL007118	KDR	P35968
MOL007118	FLT4	P35916
MOL007118	PSENEN	Q9NZ42
MOL007118	NCSTN	Q92542
MOL007118	APH1A	Q96BI3
MOL007118	PSEN1	P49768
MOL007118	APH1B	Q8WW43
MOL007119	HTR2A	P28223
MOL007119	ADRA1A	P35348
MOL007119	ADRA1B	P35368
MOL007119	AR	P10275
MOL007119	ADRB2	P07550
MOL007119	DPEP1	P16444
MOL007119	CA2	P00918
MOL007119	CDK2	P24941
MOL007119	F10	P00742
MOL007119	CCNA2	P20248
MOL007119	OPRD1	P41143
MOL007119	TOP2A	P11388
MOL007119	ESR1	P03372
MOL007119	NR3C1	P04150
MOL007119	GSK3B	P49841
MOL007119	IGHG1	P01857
MOL007119	CHRM1	P11229
MOL007119	CHRM2	P08172
MOL007119	CHRM3	P20309
MOL007119	OPRM1	P35372
MOL007119	CHRNA7	P36544
MOL007119	NCOA1	Q15788
MOL007119	NCOA2	Q15596
MOL007119	PTGS1	P23219
MOL007119	PTGS2	P35354
MOL007119	PIM1	P11309
MOL007119	RXRA	P19793
MOL007119	SCN5A	Q14524
MOL007120	ACHE	P22303
MOL007120	CA2	P00918
MOL007120	NR3C1	P04150
MOL007120	NCOA1	Q15788
MOL007120	NCOA2	Q15596
MOL007120	PGR	P06401
MOL007120	PTGS2	P35354

MOL007120	F2	P00734
MOL007121	HSD11B1	P28845
MOL007121	ACHE	P22303
MOL007121	ALOX12	P18054
MOL007121	ALOX15	P16050
MOL007121	COMT	P21964
MOL007121	DRD4	P21917
MOL007121	ESR1	P03372
MOL007121	PGGT1B	P53609
MOL007121	MTNR1A	P48039
MOL007121	MTNR1B	P49286
MOL007121	MDM2	Q00987
MOL007121	PIK3CA	P42336
MOL007121	PTGES	O14684
MOL007121	FNTA	P49354
MOL007121	FNTB	P49356
MOL007122	ACHE	P22303
MOL007122	CES1	P23141
MOL007122	ADAM17	P78536
MOL007122	AKR1C3	P42330
MOL007122	AKR1B1	P15121
MOL007122	ADRA1A	P35348
MOL007122	ADRA1B	P35368
MOL007122	ADRA1D	P25100
MOL007122	ADRA2C	P18825
MOL007122	AR	P10275
MOL007122	ADRB2	P07550
MOL007122	CA2	P00918
MOL007122	CES2	O00748
MOL007122	DRD5	P21918
MOL007122	OPRD1	P41143
MOL007122	TOP1	P11387
MOL007122	TOP2A	P11388
MOL007122	DRD1	P21728
MOL007122	CDC25A	P30304
MOL007122	CDC25B	P30305
MOL007122	ESR1	P03372
MOL007122	PTPN22	Q9Y2R2
MOL007122	LIPE	Q05469
MOL007122	PTPRC	P08575
MOL007122	MMP13	P45452
MOL007122	MMP8	P22894
MOL007122	MTNR1A	P48039
MOL007122	MTNR1B	P49286

MOL007122	GRM5	P41594
MOL007122	MAOB	P27338
MOL007122	CHRM1	P11229
MOL007122	CHRM2	P08172
MOL007122	CHRM3	P20309
MOL007122	CHRM4	P08173
MOL007122	CHRM5	P08912
MOL007122	OPRM1	P35372
MOL007122	CHRNA7	P36544
MOL007122	NOS1	P29475
MOL007122	NCOA2	Q15596
MOL007122	PIK3CA	P42336
MOL007122	PTGS1	P23219
MOL007122	PTGS2	P35354
MOL007122	PBRM1	Q86U86
MOL007122	PTPN1	P18031
MOL007122	PTPRF	P10586
MOL007122	RXRA	P19793
MOL007122	STAT3	P40763
MOL007122	SCN5A	Q14524
MOL007122	SLC6A3	Q01959
MOL007122	TNKS	O95271
MOL007122	TNKS2	Q9H2K2
MOL007122	SMARCA4	P51532
MOL007123	ACHE	P22303
MOL007123	CES1	P23141
MOL007123	ADAM17	P78536
MOL007123	AKR1C3	P42330
MOL007123	AKR1B1	P15121
MOL007123	CES2	O00748
MOL007123	TOP1	P11387
MOL007123	CDC25A	P30304
MOL007123	CDC25B	P30305
MOL007123	PTPN22	Q9Y2R2
MOL007123	LIPE	Q05469
MOL007123	PTPRC	P08575
MOL007123	MMP13	P45452
MOL007123	MMP8	P22894
MOL007123	MTNR1A	P48039
MOL007123	MTNR1B	P49286
MOL007123	GRM5	P41594
MOL007123	MAOB	P27338
MOL007123	PIK3CA	P42336
MOL007123	PBRM1	Q86U86

MOL007123	PTPN1	P18031
MOL007123	PTPRF	P10586
MOL007123	STAT3	P40763
MOL007123	TNKS	O95271
MOL007123	TNKS2	Q9H2K2
MOL007123	SMARCA4	P51532
MOL007124	ACHE	P22303
MOL007124	CES1	P23141
MOL007124	AKR1B10	O60218
MOL007124	AKR1B1	P15121
MOL007124	ADRA1A	P35348
MOL007124	ADRA1B	P35368
MOL007124	ADRA1D	P25100
MOL007124	AR	P10275
MOL007124	BCL2	P10415
MOL007124	BCL2L1	Q07817
MOL007124	ADRB2	P07550
MOL007124	CA2	P00918
MOL007124	CES2	O00748
MOL007124	CCR9	P51686
MOL007124	CDK2	P24941
MOL007124	PDE3A	Q14432
MOL007124	CCNA2	P20248
MOL007124	OPRD1	P41143
MOL007124	DHODH	Q02127
MOL007124	TOP1	P11387
MOL007124	TOP2A	P11388
MOL007124	DRD1	P21728
MOL007124	CDC25A	P30304
MOL007124	CDC25B	P30305
MOL007124	DUSP1	P28562
MOL007124	ESR1	P03372
MOL007124	FABP4	P15090
MOL007124	FABP3	P05413
MOL007124	PTGDR2	Q9Y5Y4
MOL007124	GABRA1	P14867
MOL007124	GSK3B	P49841
MOL007124	HSP90AB1	P08238
MOL007124	IDO1	P14902
MOL007124	MCL1	Q07820
MOL007124	PTPRC	P08575
MOL007124	KDM4E	B2RXH2
MOL007124	MALT1	Q9UDY8
MOL007124	CHRM1	P11229

MOL007124	CHRM2	P08172
MOL007124	CHRM3	P20309
MOL007124	CHRM4	P08173
MOL007124	OPRM1	P35372
MOL007124	CHRNA7	P36544
MOL007124	NOS1	P29475
MOL007124	MDM2	Q00987
MOL007124	PPARG	P37231
MOL007124	PTGS1	P23219
MOL007124	PTGS2	P35354
MOL007124	PTGER1	P34995
MOL007124	PTGER3	P43115
MOL007124	PTGER4	P35408
MOL007124	PTPN1	P18031
MOL007124	PTPN6	P29350
MOL007124	PIM1	P11309
MOL007124	PTPRF	P10586
MOL007124	RXRA	P19793
MOL007124	STAT3	P40763
MOL007124	SCN5A	Q14524
MOL007124	SLC6A3	Q01959
MOL007124	SLC6A4	P31645
MOL007124	TDP2	O95551
MOL007125	ACHE	P22303
MOL007125	CES1	P23141
MOL007125	AKR1B10	O60218
MOL007125	AKR1B1	P15121
MOL007125	ADRA1B	P35368
MOL007125	ADRA1D	P25100
MOL007125	AMPD1	P23109
MOL007125	AMPD2	Q01433
MOL007125	AMPD3	Q01432
MOL007125	AR	P10275
MOL007125	ADRB2	P07550
MOL007125	CA2	P00918
MOL007125	CES2	O00748
MOL007125	CCR9	P51686
MOL007125	CYP19A1	P11511
MOL007125	TOP1	P11387
MOL007125	TOP2A	P11388
MOL007125	CDC25A	P30304
MOL007125	CDC25B	P30305
MOL007125	EDNRA	P25101
MOL007125	EDNRB	P24530

MOL007125	FABP4	P15090
MOL007125	FABP5	Q01469
MOL007125	FABP3	P05413
MOL007125	HAO2	Q9NYQ3
MOL007125	HCAR2	Q8TDS4
MOL007125	IGHG1	P01857
MOL007125	MCL1	Q07820
MOL007125	KIF11	P52732
MOL007125	LDHA	P00338
MOL007125	LDHB	P07195
MOL007125	MALT1	Q9UDY8
MOL007125	CHRM1	P11229
MOL007125	CHRM3	P20309
MOL007125	OPRM1	P35372
MOL007125	CHRNA7	P36544
MOL007125	NCOA1	Q15788
MOL007125	NCOA2	Q15596
MOL007125	PPARG	P37231
MOL007125	PPARA	Q07869
MOL007125	PPARD	Q03181
MOL007125	PTGS1	P23219
MOL007125	PTGS2	P35354
MOL007125	PTGER1	P34995
MOL007125	PTGER4	P35408
MOL007125	PTPN1	P18031
MOL007125	PTPN6	P29350
MOL007125	PTPN11	Q06124
MOL007125	STAT3	P40763
MOL007125	SCN5A	Q14524
MOL007125	SLC5A2	P31639
MOL007125	STS	P08842
MOL007125	AGTR1	P30556
MOL007125	TDP2	O95551
MOL007127	ACHE	P22303
MOL007127	ADRA1A	P35348
MOL007127	ADRB2	P07550
MOL007127	CA2	P00918
MOL007127	DPP4	P27487
MOL007127	DRD1	P21728
MOL007127	GABRA1	P14867
MOL007127	HSP90AB1	P08238
MOL007127	IGHG1	P01857
MOL007127	CHRM3	P20309
MOL007127	CHRM5	P08912

MOL007127	OPRM1	P35372
MOL007127	CHRNA7	P36544
MOL007127	NCOA1	Q15788
MOL007127	PIK3CG	P48736
MOL007127	PTGS1	P23219
MOL007127	PTGS2	P35354
MOL007127	RXRA	P19793
MOL007127	SCN5A	Q14524
MOL007127	F2	P00734
MOL007130	AR	P10275
MOL007130	CALM1	P62158
MOL007130	ESR1	P03372
MOL007130	HSP90AB1	P08238
MOL007130	PTPN1	P18031
MOL007130	NOS3	P29474
MOL007130	PTGS1	P23219
MOL007130	PTGS2	P35354
MOL007130	F2	P00734
MOL007130	PRSS1	P07477
MOL007132	AR	P10275
MOL007132	CCNA2	P20248
MOL007132	DPP4	P27487
MOL007132	ESR1	P03372
MOL007132	PPARG	P37231
MOL007132	PTGS2	P35354
MOL007132	F2	P00734
MOL007132	PRSS1	P07477
MOL007141	AKR1B1	P15121
MOL007141	AMPD2	Q01433
MOL007141	AMPD3	Q01432
MOL007141	CAMKK2	Q96RR4
MOL007141	CSNK2A1	P68400
MOL007141	CASP3	P42574
MOL007141	EGFR	P00533
MOL007141	PTGDR2	Q9Y5Y4
MOL007141	GRK6	P43250
MOL007141	ITGA2B	P08514
MOL007141	ITGAV	P06756
MOL007141	ITGB5	P18084
MOL007141	SELL	P14151
MOL007141	PYGL	P06737
MOL007141	KDM3A	Q9Y4C1
MOL007141	KDM4C	Q9H3R0
MOL007141	MKNK2	Q9HBH9

MOL007141	MMP1	P03956
MOL007141	MMP2	P08253
MOL007141	MMP8	P22894
MOL007141	MMP9	P14780
MOL007141	PYGM	P11217
MOL007141	NOX4	Q9NPH5
MOL007141	MME	P08473
MOL007141	PARP1	P09874
MOL007141	PTGS2	P35354
MOL007141	SELP	P16109
MOL007141	SELE	P16581
MOL007141	CHEK1	O14757
MOL007141	PIM1	P11309
MOL007141	NEU3	Q9UQ49
MOL007141	THRA	P10827
MOL007141	THRB	P10828
MOL007141	VCP	P55072
MOL007141	TTR	P02766
MOL007141	FYN	P06241
MOL007141	LCK	P06239
MOL007141	ITGB3	P05106
MOL007141	ITGB1	P05556
MOL007142	AKR1B1	P15121
MOL007142	ABCG2	Q9UNQ0
MOL007142	APP	P05067
MOL007142	CNR1	P21554
MOL007142	CNR2	P34972
MOL007142	CA4	P22748
MOL007142	CA7	P43166
MOL007142	CA12	O43570
MOL007142	F7	P08709
MOL007142	PTGS2	P35354
MOL007142	MMP1	P03956
MOL007142	MMP12	P39900
MOL007142	MMP13	P45452
MOL007142	MMP2	P08253
MOL007142	MMP3	P08254
MOL007142	MMP9	P14780
MOL007142	PTPN1	P18031
MOL007142	ABCC1	P33527
MOL007142	ABCB1	P08183
MOL007142	PTGES	O14684
MOL007142	TTR	P02766
MOL007142	PRSS1	P07477

MOL007142	PRSS3	P35030
MOL007142	FYN	P06241
MOL007143	ACHE	P22303
MOL007143	CTSB	P07858
MOL007143	CTSK	P43235
MOL007143	CTSL	P07711
MOL007143	PTGS1	P23219
MOL007143	PTGS2	P35354
MOL007143	SLC6A3	Q01959
MOL007143	EPHX2	P34913
MOL007143	EPHX1	P07099
MOL007143	GABRB3	P28472
MOL007143	GABRA2	P47869
MOL007143	GABRG2	P18507
MOL007143	NR3C1	P04150
MOL007143	GRM1	Q13255
MOL007143	PTPN1	P18031
MOL007143	CHRM1	P11229
MOL007143	CHRM2	P08172
MOL007143	NCOA1	Q15788
MOL007143	NCOA2	Q15596
MOL007143	HCRTR2	O43614
MOL007143	P2RX7	Q99572
MOL007143	PABPC1	P11940
MOL007143	PGR	P06401
MOL007143	PREP	P48147
MOL007143	RXRA	P19793
MOL007143	HTR2B	P41595
MOL007143	TRPM8	Q7Z2W7
MOL007143	GABRA3	P34903
MOL007143	GABRA1	P14867
MOL007143	GABRA5	P31644
MOL007143	GABRA6	Q16445
MOL007145	HTR1A	P08908
MOL007145	HTR1B	P28222
MOL007145	HTR2A	P28223
MOL007145	HTR2C	P28335
MOL007145	HTR3A,	P46098
MOL007145	ACHE	P22303
MOL007145	AKR1B10	O60218
MOL007145	ADRA1A	P35348
MOL007145	ADRA1B	P35368
MOL007145	ADRA2A	P08913
MOL007145	ADRA2B	P18089

MOL007145	ALOX12	P18054
MOL007145	ALOX15	P16050
MOL007145	ADRB2	P07550
MOL007145	CA7	P43166
MOL007145	CA12	O43570
MOL007145	CA14	Q9ULX7
MOL007145	PDE3A	Q14432
MOL007145	UQCRB	P14927
MOL007145	DRD5	P21918
MOL007145	DRD2	P14416
MOL007145	OPRD1	P41143
MOL007145	DRD1	P21728
MOL007145	DUSP3	P51452
MOL007145	EPHX2	P34913
MOL007145	EPHX1	P07099
MOL007145	ESR1	P03372
MOL007145	ESR2	Q92731
MOL007145	GABRA1	P14867
MOL007145	GABRE	P78334
MOL007145	GABRG3	Q99928
MOL007145	GABRA2	P47869
MOL007145	GABRA3	P34903
MOL007145	GABRA5	P31644
MOL007145	GABRA6	Q16445
MOL007145	GSTM2	P28161
MOL007145	GSTP1	P09211
MOL007145	HDAC2	Q92769
MOL007145	HDAC4	P56524
MOL007145	HDAC8	Q9BY41
MOL007145	RET	P07949
MOL007145	CHRM1	P11229
MOL007145	CHRM2	P08172
MOL007145	CHRM3	P20309
MOL007145	CHRM4	P08173
MOL007145	CHRM5	P08912
MOL007145	OPRM1	P35372
MOL007145	CHRNA7	P36544
MOL007145	CHRNA2	Q15822
MOL007145	PTGS1	P23219
MOL007145	PTGS2	P35354
MOL007145	PRKD1	Q15139
MOL007145	AKT1	P31749
MOL007145	SCN5A	Q14524
MOL007145	SCN9A	Q15858

MOL007145	SLC6A3	Q01959
MOL007145	SLC6A2	P23975
MOL007145	SLC6A4	P31645
MOL007145	F2	P00734
MOL007145	TUBB1	Q9H4B7
MOL007149	HSD11B1	P28845
MOL007149	ACHE	P22303
MOL007149	AR	P10275
MOL007149	ALOX15	P16050
MOL007149	ALOX5	P09917
MOL007149	CASR	P41180
MOL007149	CA12	O43570
MOL007149	CCR1	P32246
MOL007149	CDK4	P11802
MOL007149	PTGS2	P35354
MOL007149	CYP19A1	P11511
MOL007149	CYP24A1	Q07973
MOL007149	CYP2D6	P10635
MOL007149	POLA1	P09884
MOL007149	ELAVL1	Q15717
MOL007149	HSD17B1	P14061
MOL007149	HSD17B2	P37059
MOL007149	ESR1	P03372
MOL007149	ESR2	Q92731
MOL007149	GABRB3	P28472
MOL007149	GABRA2	P47869
MOL007149	NR3C1	P04150
MOL007149	GPBAR1	Q8TDU6
MOL007149	HNF4A	P41235
MOL007149	KAT2B	Q92831
MOL007149	HDAC2	Q92769
MOL007149	LDLR	P01130
MOL007149	LRRK2	Q5S007
MOL007149	NR1H3	Q13133
MOL007149	NR1H2	P55055
MOL007149	MC4R	P32245
MOL007149	NR3C2	P08235
MOL007149	MAOA	P21397
MOL007149	NOX4	Q9NPH5
MOL007149	NOS2	P35228
MOL007149	PLA2G2A	P14555
MOL007149	PLA2G5	P39877
MOL007149	PRKCZ	Q05513
MOL007149	QRFPR	Q96P65

MOL007149	HTR2B	P41595
MOL007149	SLC6A4	P31645
MOL007149	CYP27A1	Q02318
MOL007149	STS	P08842
MOL007149	SAE1	Q9UBE0
MOL007149	SHBG	P04278
MOL007149	TBXA2R	P21731
MOL007149	TSPO	P30536
MOL007149	SRC	P12931
MOL007149	KDR	P35968
MOL007149	GABRG2	P18507
MOL007149	GABRA5	P31644
MOL007149	GABRA1	P14867
MOL007149	GABRA3	P34903
MOL007150	ACHE	P22303
MOL007150	CA2	P00918
MOL007150	DPP4	P27487
MOL007150	HSP90AB1	P08238
MOL007150	NCOA1	Q15788
MOL007150	PTGS2	P35354
MOL007150	F2	P00734
MOL007150	PRSS1	P07477
MOL007151	ACHE	P22303
MOL007151	CA2	P00918
MOL007151	DPP4	P27487
MOL007151	HSP90AB1	P08238
MOL007151	NCOA1	Q15788
MOL007151	PTGS2	P35354
MOL007151	F2	P00734
MOL007152	ACHE	P22303
MOL007152	CA2	P00918
MOL007152	DPP4	P27487
MOL007152	HSP90AB1	P08238
MOL007152	NCOA1	Q15788
MOL007152	PTGS2	P35354
MOL007152	F2	P00734
MOL007154	HSD11B1	P28845
MOL007154	ACHE	P22303
MOL007154	AHSA1	O95433
MOL007154	CES1	P23141
MOL007154	AKR1B1	P15121
MOL007154	ADRA1A	P35348
MOL007154	FAAH	O00519
MOL007154	AR	P10275

MOL007154	BCL2	P10415
MOL007154	ALOX5	P09917
MOL007154	ADRB2	P07550
MOL007154	BRS3	P32247
MOL007154	BRD2	P25440
MOL007154	BRD3	Q15059
MOL007154	BRD4	O60885
MOL007154	CALCR	P30988
MOL007154	CES2	O00748
MOL007154	CSNK1G1	Q9HCP0
MOL007154	CASP3	P42574
MOL007154	CTSS	P25774
MOL007154	TP53	P04637
MOL007154	CDKN1A	P38936
MOL007154	CYP19A1	P11511
MOL007154	CYP1A2	P05177
MOL007154	CYP3A4	P08684
MOL007154	OPRD1	P41143
MOL007154	DPP4	P27487
MOL007154	DRD1	P21728
MOL007154	SLC6A3	Q01959
MOL007154	CDC25A	P30304
MOL007154	CDC25B	P30305
MOL007154	CDC25C	P30307
MOL007154	EDN1	P05305
MOL007154	EDNRA	P25101
MOL007154	ECE1	P42892
MOL007154	EED	O75530
MOL007154	RBBP4	Q09028
MOL007154	FASN	P49327
MOL007154	HRH3	Q9Y5N1
MOL007154	ITGB3	P05106
MOL007154	PTPRC	P08575
MOL007154	MPI	P34949
MOL007154	MAPKAPK2	P49137
MOL007154	MMP9	P14780
MOL007154	MTNR1A	P48039
MOL007154	MTNR1B	P49286
MOL007154	GRM5	P41594
MOL007154	MAP3K8	P41279
MOL007154	MGLL	Q99685
MOL007154	CHRM1	P11229
MOL007154	CHRM2	P08172
MOL007154	CHRM3	P20309

MOL007154	CHRM4	P08173
MOL007154	CHRM5	P08912
MOL007154	OPRM1	P35372
MOL007154	MYC	P01106
MOL007154	CHRNA7	P36544
MOL007154	NFKBIA	P25963
MOL007154	NCOA1	Q15788
MOL007154	NR1I2	O75469
MOL007154	NR4A1	P22736
MOL007154	NPM1	P06748
MOL007154	HCRTR1	O43613
MOL007154	HCRTR2	O43614
MOL007154	P2RX7	Q99572
MOL007154	PDE10A	Q9Y233
MOL007154	PDE5A	O76074
MOL007154	PARP4	Q9UKK3
MOL007154	PGR	P06401
MOL007154	PTGS2	P35354
MOL007154	PTPN6	P29350
MOL007154	PTPN11	Q06124
MOL007154	FOS	P01100
MOL007154	RXRA	P19793
MOL007154	AURKB	Q96GD4
MOL007154	HTR6	P50406
MOL007154	SCN5A	Q14524
MOL007154	TERT	O14746
MOL007154	F2	P00734
MOL007154	RELA	Q04206
MOL007154	TSPO	P30536
MOL007154	KCNA5	P22460
MOL007154	SUZ12	Q15022
MOL007154	RBBP7	Q16576
MOL007154	EZH2	Q15910
MOL007155	ACHE	P22303
MOL007155	ADRA1A	P35348
MOL007155	ADRB2	P07550
MOL007155	OPRD1	P41143
MOL007155	DPP4	P27487
MOL007155	CHRM1	P11229
MOL007155	OPRM1	P35372
MOL007155	CHRNA7	P36544
MOL007155	NCOA1	Q15788
MOL007155	PTGS2	P35354
MOL007155	SCN5A	Q14524

MOL007155	F2	P00734
MOL007155	PRSS1	P07477
MOL007156	CES1	P23141
MOL007156	ADORA1	P30542
MOL007156	ADORA2A	P29274
MOL007156	ADORA2B	P29275
MOL007156	ADORA3	P0DMS8
MOL007156	AKR1B1	P15121
MOL007156	ADRA1A	P35348
MOL007156	AR	P10275
MOL007156	DPEP1	P16444
MOL007156	BRS3	P32247
MOL007156	CALM1	P62158
MOL007156	CES2	O00748
MOL007156	CDC7	O00311
MOL007156	F10	P00742
MOL007156	CYP19A1	P11511
MOL007156	CDC25A	P30304
MOL007156	CDC25B	P30305
MOL007156	CDC25C	P30307
MOL007156	EGFR	P00533
MOL007156	ESR1	P03372
MOL007156	EED	O75530
MOL007156	RBBP4	Q09028
MOL007156	HSP90AB1	P08238
MOL007156	IGHG1	P01857
MOL007156	CXCR2	P25025
MOL007156	PTPRC	P08575
MOL007156	MPI	P34949
MOL007156	MAPK14	Q16539
MOL007156	MTNR1A	P48039
MOL007156	MTNR1B	P49286
MOL007156	MAOA	P21397
MOL007156	MAOB	P27338
MOL007156	NCOA1	Q15788
MOL007156	NCOA2	Q15596
MOL007156	NR4A1	P22736
MOL007156	HCRTR2	O43614
MOL007156	P2RX7	Q99572
MOL007156	PRF1	P14222
MOL007156	PPARG	P37231
MOL007156	PTGS1	P23219
MOL007156	PTGS2	P35354
MOL007156	PTPN6	P29350

MOL007156	PTPN11	Q06124
MOL007156	NQO2	P16083
MOL007156	PIM1	P11309
MOL007156	PIM2	Q9P1W9
MOL007156	PIM3	Q86V86
MOL007156	SCN5A	Q14524
MOL007156	TERT	O14746
MOL007156	KDR	P35968
MOL007156	SUZ12	Q15022
MOL007156	RBBP7	Q16576
MOL007156	EZH2	Q15910
MOL007783	EPHX2	P34913
MOL007783	HSP90AA1	P07900
MOL007796	HSD11B1	P28845
MOL007796	HSD11B2	P80365
MOL007796	ACHE	P22303
MOL007796	AKR1C1	Q04828
MOL007796	AKR1C2	P52895
MOL007796	AKR1C3	P42330
MOL007796	FAAH	O00519
MOL007796	AR	P10275
MOL007796	EBP	Q15125
MOL007796	ALOX5	P09917
MOL007796	CNR1	P21554
MOL007796	CNR2	P34972
MOL007796	CES2	O00748
MOL007796	CTRC	Q99895
MOL007796	SERPINA6	P08185
MOL007796	PTGS2	P35354
MOL007796	CYP17A1	P05093
MOL007796	CYP19A1	P11511
MOL007796	CYP2C19	P33261
MOL007796	POLA1	P09884
MOL007796	DRD2	P14416
MOL007796	SLC6A3	Q01959
MOL007796	EPAS1	Q99814
MOL007796	HSD17B2	P37059
MOL007796	NR3C1	P04150
MOL007796	G6PD	P11413
MOL007796	GSK3B	P49841
MOL007796	HMGCR	P04035
MOL007796	NR1H3	Q13133
MOL007796	NR1H2	P55055
MOL007796	NR3C2	P08235

MOL007796	TACR2	P21452
MOL007796	NPC1L1	Q9UHC9
MOL007796	NOS2	P35228
MOL007796	SLC6A2	P23975
MOL007796	ABCB1	P08183
MOL007796	PDE4D	Q08499
MOL007796	PGR	P06401
MOL007796	PREP	P48147
MOL007796	PTGES	O14684
MOL007796	PTGER2	P43116
MOL007796	PTGIR	P43119
MOL007796	FNTA	P49354
MOL007796	PTPN1	P18031
MOL007796	HTR1A	P08908
MOL007796	SIGMAR1	Q99720
MOL007796	SRD5A2	P31213
MOL007796	SHBG	P04278
MOL007796	F2	P00734
MOL007796	TBXA2R	P21731
MOL007796	TNF	P01375
MOL007796	PRSS1	P07477
MOL007796	FNTB	P49356
MOL007799	HSD11B1	P28845
MOL007799	HSD11B2	P80365
MOL007799	ACHE	P22303
MOL007799	AKR1C1	Q04828
MOL007799	AKR1C2	P52895
MOL007799	AKR1C3	P42330
MOL007799	FAAH	O00519
MOL007799	AR	P10275
MOL007799	EBP	Q15125
MOL007799	ALOX5	P09917
MOL007799	CNR2	P34972
MOL007799	CES2	O00748
MOL007799	CTRC	Q99895
MOL007799	SERPINA6	P08185
MOL007799	PTGS2	P35354
MOL007799	CYP17A1	P05093
MOL007799	CYP19A1	P11511
MOL007799	CYP2C19	P33261
MOL007799	POLA1	P09884
MOL007799	DRD2	P14416
MOL007799	SLC6A3	Q01959
MOL007799	EPAS1	Q99814

MOL007799	HSD17B2	P37059
MOL007799	NR3C1	P04150
MOL007799	G6PD	P11413
MOL007799	GSK3B	P49841
MOL007799	HMGCR	P04035
MOL007799	OPRK1	P41145
MOL007799	NR1H3	Q13133
MOL007799	NR1H2	P55055
MOL007799	NR3C2	P08235
MOL007799	TACR2	P21452
MOL007799	NPC1L1	Q9UHC9
MOL007799	NOS2	P35228
MOL007799	SLC6A2	P23975
MOL007799	ABCB1	P08183
MOL007799	PDE4D	Q08499
MOL007799	PGR	P06401
MOL007799	PREP	P48147
MOL007799	PTGES	O14684
MOL007799	PTGER2	P43116
MOL007799	PTGIR	P43119
MOL007799	FNTA	P49354
MOL007799	PTPN1	P18031
MOL007799	HTR1A	P08908
MOL007799	SIGMAR1	Q99720
MOL007799	SRD5A2	P31213
MOL007799	PTPN2	P17706
MOL007799	SHBG	P04278
MOL007799	F2	P00734
MOL007799	TBXA2R	P21731
MOL007799	TNF	P01375
MOL007799	PRSS1	P07477
MOL007799	FNTB	P49356
MOL009653	HSD11B1	P28845
MOL009653	AR	P10275
MOL009653	BCHE	P06276
MOL009653	CNR2	P34972
MOL009653	PTGS1	P23219
MOL009653	CYP51A1	Q16850
MOL009653	CDC25A	P30304
MOL009653	HMGCR	P04035
MOL009653	NR1H3	Q13133
MOL009653	NPC1L1	Q9UHC9
MOL009653	SLC6A2	P23975
MOL009653	NR1I3	Q14994

MOL009653	PRKCA	P17252
MOL009653	PTPN1	P18031
MOL009653	UGT2B7	P16662
MOL012681	CYP27B1	O15528
MOL012681	ALOX5AP	P20292
MOL012681	ASAH1	Q13510
MOL012681	ADRA1B	P35368
MOL012681	AR	P10275
MOL012681	ADRB2	P07550
MOL012681	CALM1	P62158
MOL012681	CA2	P00918
MOL012681	PDE3A	Q14432
MOL012681	MAPK10	P53779
MOL012681	CCNE1	P24864
MOL012681	PTGS2	P35354
MOL012681	CYP24A1	Q07973
MOL012681	CYP26A1	O43174
MOL012681	CYP2C19	P33261
MOL012681	CYP2C9	P11712
MOL012681	CYP3A4	P08684
MOL012681	DCK	P27707
MOL012681	DPP4	P27487
MOL012681	EPHB4	P54760
MOL012681	FGFR1	P11362
MOL012681	PSEN2	P49810
MOL012681	QPCT	Q16769
MOL012681	HRH2	P25021
MOL012681	HRH3	Q9Y5N1
MOL012681	HRH4	Q9H3N8
MOL012681	HIF1A	Q16665
MOL012681	IGF1R	P08069
MOL012681	LTA4H	P09960
MOL012681	LIMK2	P53671
MOL012681	NR1H3	Q13133
MOL012681	CD38	P28907
MOL012681	MAPKAPK2	P49137
MOL012681	MMP14	P50281
MOL012681	MMP2	P08253
MOL012681	MMP9	P14780
MOL012681	MCHR1	Q99705
MOL012681	MTNR1A	P48039
MOL012681	MTNR1B	P49286
MOL012681	GRM5	P41594
MOL012681	NR3C2	P08235

MOL012681	NTRK3	Q16288
MOL012681	P2RX7	Q99572
MOL012681	PDE10A	Q9Y233
MOL012681	ATP4B	P51164
MOL012681	PGR	P06401
MOL012681	AKT1	P31749
MOL012681	AURKA	O14965
MOL012681	AURKB	Q96GD4
MOL012681	BRAF	P15056
MOL012681	PIM1	P11309
MOL012681	KIT	P10721
MOL012681	F2	P00734
MOL012681	SRC	P12931
MOL012681	TEK	Q02763
MOL012681	KDR	P35968
MOL012681	CDK2	P24941
MOL012681	ATP4A	P20648
MOL012681	PSENEN	Q9NZ42
MOL012681	NCSTN	Q92542
MOL012681	APH1A	Q96BI3
MOL012681	PSEN1	P49768
MOL012681	APH1B	Q8WW43
MOL012686	CALM1	P62158
MOL012686	CA2	P00918
MOL012686	HSP90AB1	P08238
MOL012686	PTGS1	P23219
MOL012686	PTGS2	P35354
MOL012686	SCN5A	Q14524
MOL012689	ACHE	P22303
MOL012689	ACACB	O00763
MOL012689	ADAM17	P78536
MOL012689	ADAM9	Q13443
MOL012689	ADORA1	P30542
MOL012689	ADORA2A	P29274
MOL012689	ADORA2B	P29275
MOL012689	ADORA3	P0DMS8
MOL012689	AKR1B10	O60218
MOL012689	ANPEP	P15144
MOL012689	AR	P10275
MOL012689	ABCG2	Q9UNQ0
MOL012689	BACE1	P56817
MOL012689	BMP1	P13497
MOL012689	BCHE	P06276
MOL012689	CALM1	P62158

MOL012689	CA2	P00918
MOL012689	CA6	P23280
MOL012689	CA13	Q8N1Q1
MOL012689	CA14	Q9ULX7
MOL012689	CTSD	P07339
MOL012689	CCNE1	P24864
MOL012689	CFD	P00746
MOL012689	CDK1	P06493
MOL012689	CDK2	P24941
MOL012689	CCNE2	O96020
MOL012689	CDK4	P11802
MOL012689	PTGS2	P35354
MOL012689	OPRD1	P41143
MOL012689	TOP2A	P11388
MOL012689	DUT	P33316
MOL012689	EPHA1	P21709
MOL012689	EPHA2	P29317
MOL012689	EPHA3	P29320
MOL012689	EPHA4	P54764
MOL012689	EPHA5	P54756
MOL012689	EPHA6	Q9UF33
MOL012689	EPHB2	P29323
MOL012689	EPHB3	P54753
MOL012689	EGFR	P00533
MOL012689	EPHX2	P34913
MOL012689	HSD17B1	P14061
MOL012689	HSD17B2	P37059
MOL012689	ESR1	P03372
MOL012689	ESR2	Q92731
MOL012689	FGFR1	P11362
MOL012689	GABRA1	P14867
MOL012689	GABRA2	P47869
MOL012689	PSEN2	P49810
MOL012689	GRK2	P25098
MOL012689	HSP90AB1	P08238
MOL012689	MET	P08581
MOL012689	HDAC8	Q9BY41
MOL012689	MCL1	Q07820
MOL012689	RET	P07949
MOL012689	MAPK3	P27361
MOL012689	MAPK1	P28482
MOL012689	MMP1	P03956
MOL012689	MMP12	P39900
MOL012689	MMP13	P45452

MOL012689	MMP14	P50281
MOL012689	MMP16	P51512
MOL012689	MMP2	P08253
MOL012689	MMP3	P08254
MOL012689	MMP7	P09237
MOL012689	MMP8	P22894
MOL012689	MMP9	P14780
MOL012689	MAOA	P21397
MOL012689	OPRM1	P35372
MOL012689	ABCC1	P33527
MOL012689	NTRK1	P04629
MOL012689	NFKB1	P19838
MOL012689	RELA	Q04206
MOL012689	NCOA2	Q15596
MOL012689	PIK3CD	O00329
MOL012689	PARP1	P09874
MOL012689	TGM2	P21980
MOL012689	PDK1	Q15118
MOL012689	RPS6KB1	P23443
MOL012689	AURKA	O14965
MOL012689	AURKB	Q96GD4
MOL012689	BRAF	P15056
MOL012689	RAF1	P04049
MOL012689	ERN1	O75460
MOL012689	SLC5A1	P13866
MOL012689	SAE1	Q9UBE0
MOL012689	THRA	P10827
MOL012689	THRB	P10828
MOL012689	BLK	P51451
MOL012689	BMX	P51813
MOL012689	PTK6	Q13882
MOL012689	BTK	Q06187
MOL012689	CSK	P41240
MOL012689	FGR	P09769
MOL012689	FYN	P06241
MOL012689	LCK	P06239
MOL012689	LYN	P07948
MOL012689	TYRO3	Q06418
MOL012689	SRC	P12931
MOL012689	SYK	P43405
MOL012689	TXK	P42681
MOL012689	YES1	P07947
MOL012689	COQ8B	Q96D53
MOL012689	UBA2	Q9UBT2

MOL012689	PSENEN	Q9NZ42
MOL012689	NCSTN	Q92542
MOL012689	APH1A	Q96BI3
MOL012689	PSEN1	P49768
MOL012689	APH1B	Q8WW43
MOL012689	CDK3	Q00526
MOL012689	CCNB1	P14635
MOL012692	HPGD	P15428
MOL012692	ACHE	P22303
MOL012692	CES1	P23141
MOL012692	SCD	O00767
MOL012692	ADORA1	P30542
MOL012692	ADORA3	P0DMS8
MOL012692	ALDH2	P05091
MOL012692	AKR1C3	P42330
MOL012692	AKR1B1	P15121
MOL012692	ALPL	P05186
MOL012692	ABCG2	Q9UNQ0
MOL012692	BACE1	P56817
MOL012692	BMP1	P13497
MOL012692	BRD4	O60885
MOL012692	BCHE	P06276
MOL012692	CAMK2D	Q13557
MOL012692	CA4	P22748
MOL012692	CA7	P43166
MOL012692	CA12	O43570
MOL012692	CBR1	P16152
MOL012692	CES2	O00748
MOL012692	COMT	P21964
MOL012692	CTSB	P07858
MOL012692	CDK2	P24941
MOL012692	CCNE2	O96020
MOL012692	CCND1	P24385
MOL012692	CDK5R1	Q15078
MOL012692	PTGS1	P23219
MOL012692	CYP19A1	P11511
MOL012692	CYP1B1	Q16678
MOL012692	DPP4	P27487
MOL012692	POLB	P06746
MOL012692	DRD1	P21728
MOL012692	DRD3	P35462
MOL012692	CDC25B	P30305
MOL012692	EGFR	P00533
MOL012692	HSD17B1	P14061

MOL012692	HSD17B3	P37058
MOL012692	ESR1	P03372
MOL012692	ESR2	Q92731
MOL012692	PSEN2	P49810
MOL012692	GCGR	P47871
MOL012692	GSK3A	P49840
MOL012692	GSK3B	P49841
MOL012692	PLA2G10	O15496
MOL012692	HSP90AA1	P07900
MOL012692	HPGDS	O60760
MOL012692	HDAC1	Q13547
MOL012692	HDAC10	Q969S8
MOL012692	HDAC11	Q96DB2
MOL012692	HDAC2	Q92769
MOL012692	HDAC3	O15379
MOL012692	NCOR2	Q9Y618
MOL012692	HDAC4	P56524
MOL012692	HDAC5	Q9UQL6
MOL012692	HDAC7	Q8WUI4
MOL012692	HDAC9	Q9UKV0
MOL012692	IKBKE	Q14164
MOL012692	KLK1	P06870
MOL012692	KLK2	P20151
MOL012692	RET	P07949
MOL012692	LTB4R	Q15722
MOL012692	MIF	P14174
MOL012692	MAPK1	P28482
MOL012692	MELK	Q14680
MOL012692	MMP12	P39900
MOL012692	MMP13	P45452
MOL012692	GRM5	P41594
MOL012692	MAOB	P27338
MOL012692	ABCC1	P33527
MOL012692	SIRT2	Q8IXJ6
MOL012692	NCOA2	Q15596
MOL012692	HCRTR1	O43613
MOL012692	PPARG	P37231
MOL012692	PLA2G1B	P04054
MOL012692	PLA2G2A	P14555
MOL012692	PLA2G5	P39877
MOL012692	PIK3CA	P42336
MOL012692	PIK3CG	P48736
MOL012692	SERPINE1	P05121
MOL012692	PLEC	Q15149

MOL012692	PTPN1	P18031
MOL012692	AURKA	O14965
MOL012692	AURKB	Q96GD4
MOL012692	BRAF	P15056
MOL012692	MTOR	P42345
MOL012692	PIM1	P11309
MOL012692	PIM2	Q9P1W9
MOL012692	PLK1	P53350
MOL012692	TAOK1	Q7L7X3
MOL012692	TAOK3	Q9H2K8
MOL012692	TBK1	Q9UHD2
MOL012692	WEE1	P30291
MOL012692	HTR3A	P46098
MOL012692	SLC5A2	P31639
MOL012692	SLC9A1	P19634
MOL012692	TAS2R31	P59538
MOL012692	SHBG	P04278
MOL012692	TYMS	P04818
MOL012692	TLR9	Q9NR96
MOL012692	LCK	P06239
MOL012692	PSENEN	Q9NZ42
MOL012692	NCSTN	Q92542
MOL012692	APH1A	Q96BI3
MOL012692	PSEN1	P49768
MOL012692	APH1B	Q8WW43
MOL012692	CCNE1	P24864
MOL012692	CDK4	P11802
MOL012692	CDK5	Q00535
MOL012714	ADORA1	P30542
MOL012714	ADORA2A	P29274
MOL012714	AKR1B1	P15121
MOL012714	ALOX5	P09917
MOL012714	ARG1	P05089
MOL012714	CA4	P22748
MOL012714	CCNB3	Q8WWL7
MOL012714	PTGS1	P23219
MOL012714	CYP1B1	Q16678
MOL012714	EPHB4	P54760
MOL012714	ESR1	P03372
MOL012714	ESR2	Q92731
MOL012714	GABRA5	P31644
MOL012714	GLO1	Q04760
MOL012714	HSP90AB1	P08238
MOL012714	NR1H3	Q13133

MOL012714	CD38	P28907
MOL012714	GRM5	P41594
MOL012714	ABCC1	P33527
MOL012714	SIRT1	Q96EB6
MOL012714	NOX4	Q9NPH5
MOL012714	PDE4B	Q07343
MOL012714	PDE4D	Q08499
MOL012714	PLA2G2A	P14555
MOL012714	PIK3CA	P42336
MOL012714	PIK3CB	P42338
MOL012714	PIK3CD	O00329
MOL012714	PI4KB	Q9UBF8
MOL012714	PLG	P00747
MOL012714	PARP1	P09874
MOL012714	PTGS2	P35354
MOL012714	PTPRS	Q13332
MOL012714	MTOR	P42345
MOL012714	PIM1	P11309
MOL012714	KIT	P10721
MOL012714	TNKS	O95271
MOL012714	TNKS2	Q9H2K2
MOL012714	TERT	O14746
MOL012714	SHBG	P04278
MOL012714	TTR	P02766
MOL012714	HCK	P08631
MOL012714	FLT3	P36888
MOL012714	XDH	P47989
MOL012714	CDK1	P06493
MOL012714	CCNB1	P14635
MOL012714	CCNB2	O95067
MOL012719	ALOX5AP	P20292
MOL012719	ADAM17	P78536
MOL012719	ADK	P55263
MOL012719	ALPL	P05186
MOL012719	PTGS1	P23219
MOL012719	MAP2K1	Q02750
MOL012719	DNM1	Q05193
MOL012719	ESR1	P03372
MOL012719	FGFR1	P11362
MOL012719	HSP90AB1	P08238
MOL012719	HSP90AA1	P07900
MOL012719	HDAC11	Q96DB2
MOL012719	HDAC4	P56524
MOL012719	HDAC5	Q9UQL6

MOL012719	HDAC7	Q8WUI4
MOL012719	HDAC9	Q9UKV0
MOL012719	HIF1A	Q16665
MOL012719	MMP1	P03956
MOL012719	MMP7	P09237
MOL012719	MMP8	P22894
MOL012719	MAP4K4	O95819
MOL012719	CHRM1	P11229
MOL012719	PLAA	Q9Y263
MOL012719	PIK3R1	P27986
MOL012719	PIK3CA	P42336
MOL012719	PIK3CD	O00329
MOL012719	AURKA	O14965
MOL012719	SCN9A	Q15858
MOL012719	SLC5A2	P31639
MOL012719	TYMP	P19971
MOL012719	PRSS1	P07477
MOL012719	FLT1	P17948
MOL012719	PIK3CB	P42338
MOL012726	CYP19A1	P11511
MOL012726	PLG	P00747
MOL012726	PTPN1	P18031
MOL012726	PRSS1	P07477
MOL012726	PRSS3	P35030
MOL012726	TRPV1	Q8NER1
MOL012735	ADORA1	P30542
MOL012735	ADORA2A	P29274
MOL012735	ADORA2B	P29275
MOL012735	ADORA3	P0DMS8
MOL012735	AKR1B1	P15121
MOL012735	CDK2	P24941
MOL012735	CCNA2	P20248
MOL012735	TOP1	P11387
MOL012735	SLC29A1	Q99808
MOL012735	ESR1	P03372
MOL012735	HSP90AB1	P08238
MOL012735	HIF1A	Q16665
MOL012735	IMPDH1	P20839
MOL012735	IMPDH2	P12268
MOL012735	IL2	P60568
MOL012735	CD38	P28907
MOL012735	PARP1	P09874
MOL012735	SLC5A1	P13866
MOL012735	SLC5A2	P31639

MOL012735	SLC28A2	O43868
MOL012735	TNF	P01375
MOL012735	PRSS1	P07477
MOL012735	XDH	P47989
MOL012753	CALM1	P62158
MOL012753	CA2	P00918
MOL012753	CDK2	P24941
MOL012753	F7	P08709
MOL012753	F10	P00742
MOL012753	TOP2A	P11388
MOL012753	ESR1	P03372
MOL012753	ESR2	Q92731
MOL012753	HSP90AB1	P08238
MOL012753	NOS2	P35228
MOL012753	NCOA1	Q15788
MOL012753	NCOA2	Q15596
MOL012753	PTGS1	P23219
MOL012753	PTGS2	P35354
MOL012753	PIM1	P11309
MOL012753	SCN5A	Q14524
MOL012753	KDR	P35968
MOL012755	YWHAG	P61981
MOL012755	PDPK1	O15530
MOL012755	ACHE	P22303
MOL012755	ADAM17	P78536
MOL012755	ADORA1	P30542
MOL012755	ADORA3	P0DMS8
MOL012755	AR	P10275
MOL012755	ABCG2	Q9UNQ0
MOL012755	GUSB	P08236
MOL012755	BACE1	P56817
MOL012755	BCHE	P06276
MOL012755	CALM1	P62158
MOL012755	CA2	P00918
MOL012755	CA4	P22748
MOL012755	CA7	P43166
MOL012755	CA12	O43570
MOL012755	CTSK	P43235
MOL012755	F10	P00742
MOL012755	CDK4	P11802
MOL012755	PTGS1	P23219
MOL012755	CYP19A1	P11511
MOL012755	CYP1B1	Q16678
MOL012755	DRD1	P21728

MOL012755	DRD2	P14416
MOL012755	DRD3	P35462
MOL012755	EPHB4	P54760
MOL012755	EGFR	P00533
MOL012755	SLC29A1	Q99808
MOL012755	HSD17B1	P14061
MOL012755	HSD17B2	P37059
MOL012755	ESR1	P03372
MOL012755	ESR2	Q92731
MOL012755	GCGR	P47871
MOL012755	GSK3B	P49841
MOL012755	HSP90AB1	P08238
MOL012755	HSP90AA1	P07900
MOL012755	HPGDS	O60760
MOL012755	KDM1A	O60341
MOL012755	MKNK2	Q9HBH9
MOL012755	MMP1	P03956
MOL012755	MAP4K4	O95819
MOL012755	MAOB	P27338
MOL012755	NOX4	Q9NPH5
MOL012755	CHRNA7	P36544
MOL012755	NOS3	P29474
MOL012755	NCOA1	Q15788
MOL012755	ODC1	P11926
MOL012755	PIK3CG	P48736
MOL012755	PLA2G1B	P04054
MOL012755	PIK3CA	P42336
MOL012755	PARP1	P09874
MOL012755	KCNH2	Q12809
MOL012755	PTGS2	P35354
MOL012755	PTPN1	P18031
MOL012755	PNP	P00491
MOL012755	PDK1	Q15118
MOL012755	RPS6KB1	P23443
MOL012755	AURKA	O14965
MOL012755	AURKB	Q96GD4
MOL012755	BRAF	P15056
MOL012755	CHEK1	O14757
MOL012755	MTOR	P42345
MOL012755	RAF1	P04049
MOL012755	SGK1	O00141
MOL012755	ERN1	O75460
MOL012755	HTR7	P34969
MOL012755	SIGMAR1	Q99720

MOL012755	SLC5A2	P31639
MOL012755	TAS2R31	P59538
MOL012755	SHBG	P04278
MOL012755	TNF	P01375
MOL012755	TRPM8	Q7Z2W7
MOL012755	UPP1	Q16831
MOL012755	KDR	P35968
MOL012760	CALM1	P62158
MOL012760	CA2	P00918
MOL012760	F10	P00742
MOL012760	TOP2A	P11388
MOL012760	ESR1	P03372
MOL012760	PTGS2	P35354
MOL012800	AR	P10275
MOL012800	CA2	P00918
MOL012800	DPP4	P27487
MOL012800	HSP90AB1	P08238
MOL012800	PRKACA	P17612
MOL012800	NOS2	P35228
MOL012800	PPARG	P37231
MOL012800	PIK3CG	P48736
MOL012800	PTGS1	P23219
MOL012800	PTGS2	P35354
