# Supplementary material for: Niaoduqing alleviates podocyte injury in high glucose model via regulating multiple targets and AGE/RAGE pathway: Network pharmacology and experimental validation
Source: Front Pharmacol. 2023 Feb 27;14:1047184. doi: 10.3389/fphar.2023.1047184 (PMC10009170; doi:10.3389/fphar.2023.1047184)
Supplement: Supplementary file 3 [file Table11.pdf]

Table S11 The result of GO and KEGG enrichment analysis to Niaoduqing

	GO Ontology	GO ID	KEGG ID
1	BP	GO:0003018	hsa04933
2	BP	GO:0008217	hsa05418
3	BP	GO:0035296	hsa04614
4	BP	GO:0097746	hsa05323
5	BP	GO:0035150	hsa05415
6	BP	GO:0043536	hsa04066
7	BP	GO:0032496	hsa04370
8	BP	GO:0002237	hsa04924
9	BP	GO:0001990	hsa04020
10	BP	GO:0009408	hsa04668
11	BP	GO:0032103	hsa04926
12	BP	GO:0003044	hsa04371
13	BP	GO:0010595	hsa05144
14	BP	GO:0043542	hsa04151
15	BP	GO:0007566	hsa05167
16	BP	GO:0043535	hsa04510
17	BP	GO:0045765	hsa05211
18	BP	GO:0019229	hsa05208
19	BP	GO:1901342	hsa05212
20	BP	GO:0010634	hsa05171
21	BP	GO:0043534	hsa05140
22	BP	GO:0009266	hsa04610
23	BP	GO:0001936	hsa05410
24	BP	GO:0010631	hsa04657
25	BP	GO:0045766	
26	BP	GO:1904018	
27	BP	GO:0090132	
28	BP	GO:0090130	
29	BP	GO:0050900	
30	BP	GO:0001935	
31	BP	GO:0007565	
32	BP	GO:0050678	
33	BP	GO:0050886	
34	BP	GO:0042310	
35	BP	GO:0002685	
36	BP	GO:1903589	
37	BP	GO:0042060	
38	BP	GO:0044706	
39	BP	GO:0048608	
40	BP	GO:0061458	
41	BP	GO:0003081	
42	BP	GO:0050920	

43	BP	GO:0050673
44	BP	GO:0010594
45	BP	GO:0003073
46	BP	GO:0031667
47	BP	GO:0001667
48	BP	GO:0001938
49	BP	GO:0001894
50	BP	GO:1902042
51	BP	GO:0033280
52	BP	GO:0090050
53	BP	GO:0010632
54	BP	GO:0046683
55	BP	GO:0001666
56	BP	GO:0060326
57	BP	GO:0060249
58	BP	GO:0050921
59	BP	GO:0036293
60	BP	GO:0140353
61	BP	GO:0019216
62	BP	GO:0014074
63	BP	GO:0045834
64	BP	GO:0071456
65	BP	GO:0001974
66	BP	GO:0070482
67	BP	GO:1903587
68	BP	GO:0036294
69	BP	GO:2001233
70	BP	GO:1902041
71	BP	GO:1903034
72	BP	GO:0031214
73	BP	GO:0110148
74	BP	GO:0018108
75	BP	GO:0001541
76	BP	GO:0018212
77	BP	GO:0007584
78	BP	GO:0048771
79	BP	GO:0002043
80	BP	GO:0071453
81	BP	GO:0002040
82	BP	GO:0050731
83	BP	GO:0032102
84	BP	GO:0030193
85	BP	GO:0050918
86	BP	GO:0050679

87	BP	GO:1900046
88	BP	GO:0034605
89	BP	GO:0050818
90	BP	GO:0097191
91	BP	GO:0097529
92	BP	GO:0002002
93	BP	GO:0002003
94	BP	GO:0008406
95	BP	GO:0009410
96	BP	GO:0006801
97	BP	GO:2001234
98	BP	GO:0001819
99	BP	GO:0045137
100	BP	GO:0006809
101	BP	GO:2000379
102	BP	GO:0030595
103	BP	GO:0090049
104	BP	GO:0042447
105	BP	GO:0043410
106	BP	GO:0072593
107	BP	GO:0046209
108	BP	GO:0008625
109	BP	GO:2001057
110	BP	GO:0046883
111	BP	GO:1903054
112	BP	GO:1903522
113	BP	GO:0050730
114	BP	GO:0033273
115	BP	GO:0002042
116	BP	GO:1903035
117	BP	GO:0008585
118	BP	GO:0048872
119	BP	GO:0045986
120	BP	GO:0090594
121	BP	GO:2001237
122	BP	GO:0007548
123	BP	GO:0046545
124	BP	GO:0098869
125	BP	GO:0001991
126	BP	GO:2000846
127	BP	GO:0048661
128	BP	GO:0001822
129	BP	GO:0010715
130	BP	GO:0043117

131	BP	GO:0046879
132	BP	GO:0033138
133	BP	GO:0072001
134	BP	GO:0035930
135	BP	GO:0071318
136	BP	GO:0009914
137	BP	GO:0002526
138	BP	GO:0043406
139	BP	GO:0010544
140	BP	GO:2000831
141	BP	GO:0002244
142	BP	GO:0046660
143	BP	GO:1990748
144	BP	GO:0032930
145	BP	GO:0002688
146	BP	GO:0038083
147	BP	GO:0097237
148	BP	GO:0062197
149	BP	GO:0001655
150	BP	GO:0048545
151	BP	GO:0019430
152	BP	GO:0030194
153	BP	GO:0035929
154	BP	GO:0045662
155	BP	GO:0045932
156	BP	GO:1900048
157	BP	GO:0016486
158	BP	GO:0032928
159	BP	GO:0050820
160	BP	GO:0061041
161	BP	GO:0002687
162	BP	GO:0003158
163	BP	GO:0003071
164	BP	GO:0050927
165	BP	GO:0071450
166	BP	GO:0071451
167	BP	GO:0140448
168	BP	GO:0046697
169	BP	GO:0050926
170	BP	GO:0001890
171	BP	GO:0033135
172	BP	GO:0046718
173	BP	GO:0010038
174	BP	GO:0002719

175	BP	GO:0007263
176	BP	GO:0031954
177	BP	GO:0050878
178	BP	GO:0010212
179	BP	GO:0000303
180	BP	GO:0010575
181	BP	GO:0061082
182	BP	GO:1990776
183	BP	GO:0032368
184	BP	GO:0045860
185	BP	GO:0044409
186	BP	GO:2001236
187	BP	GO:0098754
188	BP	GO:0000305
189	BP	GO:0070168
190	BP	GO:0006869
191	BP	GO:0002262
192	BP	GO:2000377
193	BP	GO:0110150
194	BP	GO:0003180
195	BP	GO:0001558
196	BP	GO:0001975
197	BP	GO:0033198
198	BP	GO:0043552
199	BP	GO:0001893
200	BP	GO:0034405
201	BP	GO:0035633
202	BP	GO:0045907
203	BP	GO:0052126
204	BP	GO:0048732
205	BP	GO:0044060
206	BP	GO:0090322
207	BP	GO:0043405
208	BP	GO:0002443
209	BP	GO:0048660
210	BP	GO:0003176
211	BP	GO:0090218
212	BP	GO:1905952
213	BP	GO:0006979
214	BP	GO:0010876
215	BP	GO:0022407
216	BP	GO:0010742
217	BP	GO:0090077
218	BP	GO:0048659

219	BP	GO:0003012
220	BP	GO:0002701
221	BP	GO:0009314
222	BP	GO:0010543
223	BP	GO:0045429
224	BP	GO:0071276
225	BP	GO:0023061
226	BP	GO:0033674
227	BP	GO:0042307
228	BP	GO:1904407
229	BP	GO:1905314
230	BP	GO:1901654
231	BP	GO:0071674
232	BP	GO:0046688
233	BP	GO:0071248
234	BP	GO:0031952
235	BP	GO:1904591
236	BP	GO:0045216
237	BP	GO:0071902
238	BP	GO:0016049
239	BP	GO:0051701
240	BP	GO:0042554
241	BP	GO:0043281
242	BP	GO:0071222
243	BP	GO:0061028
244	BP	GO:0050866
245	BP	GO:0042311
246	BP	GO:0043114
247	BP	GO:0043618
248	BP	GO:0002686
249	BP	GO:1903053
250	BP	GO:0007596
251	BP	GO:0042445
252	BP	GO:0014075
253	BP	GO:0030195
254	BP	GO:0030225
255	BP	GO:0038084
256	BP	GO:0060986
257	BP	GO:0002064
258	BP	GO:0071219
259	BP	GO:1900047
260	BP	GO:1904036
261	BP	GO:0000302
262	BP	GO:0007599

263	BP	GO:0050817
264	BP	GO:0045661
265	BP	GO:0016485
266	BP	GO:0002703
267	BP	GO:0071241
268	BP	GO:0046777
269	BP	GO:0043620
270	BP	GO:0050819
271	BP	GO:2000116
272	BP	GO:0003179
273	BP	GO:0031334
274	BP	GO:0010518
275	BP	GO:0048008
276	BP	GO:0010574
277	BP	GO:0003007
278	BP	GO:0071216
279	BP	GO:0033002
280	BP	GO:0043551
281	BP	GO:0048010
282	BP	GO:0090303
283	BP	GO:0042306
284	BP	GO:0090257
285	BP	GO:0097305
286	BP	GO:0051924
287	BP	GO:0007588
288	BP	GO:0010573
289	BP	GO:0045428
290	BP	GO:0046824
291	BP	GO:0060135
292	BP	GO:0022617
293	BP	GO:1904589
294	BP	GO:0009636
295	BP	GO:0001885
296	BP	GO:0006940
297	BP	GO:0080164
298	BP	GO:0003170
299	BP	GO:1905330
300	BP	GO:0072678
301	BP	GO:0006879
302	BP	GO:0010517
303	BP	GO:0050435
304	BP	GO:0046686
305	BP	GO:0051926
306	BP	GO:0002366

307	BP	GO:0060193
308	BP	GO:0002263
309	BP	GO:0002548
310	BP	GO:0043550
311	BP	GO:0051146
312	BP	GO:0015850
313	BP	GO:0033627
314	BP	GO:1903036
315	BP	GO:0035924
316	BP	GO:0034599
317	BP	GO:0097193
318	BP	GO:0044403
319	BP	GO:0051604
320	BP	GO:0043154
321	BP	GO:0061045
322	BP	GO:0001570
323	BP	GO:0019932
324	BP	GO:0048678
325	BP	GO:0018105
326	BP	GO:0019058
327	BP	GO:0045445
328	BP	GO:0008202
329	BP	GO:0055072
330	BP	GO:0097006
331	BP	GO:0071496
332	BP	GO:0034103
333	BP	GO:2000117
334	BP	GO:0060562
335	BP	GO:1900182
336	BP	GO:0048638
337	BP	GO:0051781
338	BP	GO:0046849
339	BP	GO:0062012
340	BP	GO:0060191
341	BP	GO:0018209
342	BP	GO:0002697
343	BP	GO:0007568
344	BP	GO:0051591
345	BP	GO:0002690
346	BP	GO:0030336
347	BP	GO:0001823
348	BP	GO:0002718
349	BP	GO:1901655
350	BP	GO:1904035

351	BP	GO:0070167
352	BP	GO:0002367
353	BP	GO:0002444
354	BP	GO:0110149
355	BP	GO:0019218
356	BP	GO:0071900
357	BP	GO:2000146
358	BP	GO:0051098
359	BP	GO:0050767
360	BP	GO:0030593
361	BP	GO:0051271
362	BP	GO:0019882
363	BP	GO:0046822
364	BP	GO:0002698
365	BP	GO:0003014
366	BP	GO:0006939
367	BP	GO:0061387
368	BP	GO:0032535
369	BP	GO:0042692
370	BP	GO:0050727
371	BP	GO:0040013
372	BP	GO:0046916
373	BP	GO:0072676
374	BP	GO:0045446
375	BP	GO:0051101
376	BP	GO:0030282
377	BP	GO:0010959
378	BP	GO:0051897
379	BP	GO:0001503
380	BP	GO:0043500
381	BP	GO:1904019
382	BP	GO:1990266
383	BP	GO:0030168
384	BP	GO:0043434
385	BP	GO:0016032
386	BP	GO:0055007
387	BP	GO:0071621
388	BP	GO:0006816
389	BP	GO:0048568
390	BP	GO:0034101
391	BP	GO:0043254
392	BP	GO:0052548
393	BP	GO:0002683
394	BP	GO:0007569

395	BP	GO:0045785
396	BP	GO:0051090
397	BP	GO:0001101
398	BP	GO:0051960
399	BP	GO:1900180
400	BP	GO:0045471
401	BP	GO:0055076
402	BP	GO:0045598
403	BP	GO:0008584
404	BP	GO:0032355
405	BP	GO:0046546
406	BP	GO:0050729
407	BP	GO:0062013
408	BP	GO:0052547
409	BP	GO:0043524
410	BP	GO:0042542
411	BP	GO:0051051
412	BP	GO:0035148
413	BP	GO:0051384
414	BP	GO:0097530
415	BP	GO:0007605
416	BP	GO:0008360
417	BP	GO:0050770
418	BP	GO:0006606
419	BP	GO:0034614
420	BP	GO:0042110
421	BP	GO:0035051
422	BP	GO:0051170
423	BP	GO:0043271
424	BP	GO:0090316
425	BP	GO:0051100
426	BP	GO:0002700
427	BP	GO:0050680
428	BP	GO:2001242
429	BP	GO:0002291
430	BP	GO:0002887
431	BP	GO:0009750
432	BP	GO:0019062
433	BP	GO:0030214
434	BP	GO:0031284
435	BP	GO:0031915
436	BP	GO:0042756
437	BP	GO:0044557
438	BP	GO:0045843

439	BP	GO:0048671
440	BP	GO:0051918
441	BP	GO:0060947
442	BP	GO:0071838
443	BP	GO:0090037
444	BP	GO:0097084
445	BP	GO:1901203
446	BP	GO:1901725
447	BP	GO:2000048
448	BP	GO:2000169
449	BP	GO:0046661
450	BP	GO:0043112
451	BP	GO:0031960
452	BP	GO:0006937
453	BP	GO:0046890
454	BP	GO:0048469
455	BP	GO:0051099
456	BP	GO:0001660
457	BP	GO:0006527
458	BP	GO:0031652
459	BP	GO:0033632
460	BP	GO:0033860
461	BP	GO:0035865
462	BP	GO:0042167
463	BP	GO:0043619
464	BP	GO:0046149
465	BP	GO:0048635
466	BP	GO:0060312
467	BP	GO:0071679
468	BP	GO:0106049
469	BP	GO:1901862
470	BP	GO:1902033
471	BP	GO:1902667
472	BP	GO:0001659
473	BP	GO:0050954
474	BP	GO:0051302
475	BP	GO:0007626
476	BP	GO:0010721
477	BP	GO:0008361
478	BP	GO:0006787
479	BP	GO:0033004
480	BP	GO:0033015
481	BP	GO:0042118
482	BP	GO:0043129

483	BP	GO:0043301
484	BP	GO:0044650
485	BP	GO:0045602
486	BP	GO:0060009
487	BP	GO:0072683
488	BP	GO:0090557
489	BP	GO:0150065
490	BP	GO:1903800
491	BP	GO:2000727
492	BP	GO:0051896
493	BP	GO:0030308
494	BP	GO:0002551
495	BP	GO:0007183
496	BP	GO:0014745
497	BP	GO:0031282
498	BP	GO:0031392
499	BP	GO:0032429
500	BP	GO:0061418
501	BP	GO:0061744
502	BP	GO:0006164
503	BP	GO:0002285
504	BP	GO:0022408
505	BP	GO:0010872
506	BP	GO:0030213
507	BP	GO:0031650
508	BP	GO:0035641
509	BP	GO:0038166
510	BP	GO:0047484
511	BP	GO:0048681
512	BP	GO:0048875
513	BP	GO:0050930
514	BP	GO:0051917
515	BP	GO:0060088
516	BP	GO:0060841
517	BP	GO:0097531
518	BP	GO:0031099
519	BP	GO:0072522
520	BP	GO:0032388
521	BP	GO:0010763
522	BP	GO:0044406
523	BP	GO:0048569
524	BP	GO:0140374
525	BP	GO:1903799
526	BP	GO:2001028

527	BP	GO:2001279
528	BP	GO:1901888
529	BP	GO:0017038
530	BP	GO:0002573
531	BP	GO:1901215
532	BP	GO:0002093
533	BP	GO:0002693
534	BP	GO:0003084
535	BP	GO:0010819
536	BP	GO:0017014
537	BP	GO:0018119
538	BP	GO:0033631
539	BP	GO:0034374
540	BP	GO:0035864
541	BP	GO:0045651
542	BP	GO:0070571
543	BP	GO:0090336
544	BP	GO:1901163
545	BP	GO:1901201
546	BP	GO:1902931
547	BP	GO:0043491
548	BP	GO:0043523
549	BP	GO:0031669
550	BP	GO:0009612
551	BP	GO:0070374
552	BP	GO:0003184
553	BP	GO:0010934
554	BP	GO:0010935
555	BP	GO:0016264
556	BP	GO:0060033
557	BP	GO:0060977
558	BP	GO:0061450
559	BP	GO:0090036
560	BP	GO:1901550
561	BP	GO:1902894
562	BP	GO:1903140
563	BP	GO:0009746
564	BP	GO:0030728
565	BP	GO:0031649
566	BP	GO:0034433
567	BP	GO:0034434
568	BP	GO:0034435
569	BP	GO:0043116
570	BP	GO:0043217

571	BP	GO:0048535
572	BP	GO:0050665
573	BP	GO:0060008
574	BP	GO:0060391
575	BP	GO:0060749
576	BP	GO:0061377
577	BP	GO:0090190
578	BP	GO:0097709
579	BP	GO:1903209
580	BP	GO:0010001
581	BP	GO:0034284
582	BP	GO:0050769
583	BP	GO:0033157
584	BP	GO:0045444
585	BP	GO:0071695
586	BP	GO:0010744
587	BP	GO:0031065
588	BP	GO:0031290
589	BP	GO:0045780
590	BP	GO:0046716
591	BP	GO:1901739
592	BP	GO:0048588
593	BP	GO:0060560
594	BP	GO:0002699
595	BP	GO:0048738
596	BP	GO:0003159
597	BP	GO:0006525
598	BP	GO:0010042
599	BP	GO:0032793
600	BP	GO:0048670
601	BP	GO:0061154
602	BP	GO:0071243
603	BP	GO:1903039
604	BP	GO:0060047
605	BP	GO:0002689
606	BP	GO:0003177
607	BP	GO:0008090
608	BP	GO:0035813
609	BP	GO:0060117
610	BP	GO:0071498
611	BP	GO:0090026
612	BP	GO:0090189
613	BP	GO:1905209
614	BP	GO:2000047

615	BP	GO:0031668
616	BP	GO:0051402
617	BP	GO:0031281
618	BP	GO:0045540
619	BP	GO:0060965
620	BP	GO:0061042
621	BP	GO:0106118
622	BP	GO:1901522
623	BP	GO:2000725
624	BP	GO:0045926
625	BP	GO:0003015
626	BP	GO:0010951
627	BP	GO:0009743
628	BP	GO:0034612
629	BP	GO:0009165
630	BP	GO:0002052
631	BP	GO:0010640
632	BP	GO:0032740
633	BP	GO:0033081
634	BP	GO:0035162
635	BP	GO:0035640
636	BP	GO:0035812
637	BP	GO:0036303
638	BP	GO:0045723
639	BP	GO:0051349
640	BP	GO:0071636
641	BP	GO:0090335
642	BP	GO:1903306
643	BP	GO:0090596
644	BP	GO:1901293
645	BP	GO:0051091
646	BP	GO:0002438
647	BP	GO:0010226
648	BP	GO:0010288
649	BP	GO:0030810
650	BP	GO:0036003
651	BP	GO:0044062
652	BP	GO:0045649
653	BP	GO:0048641
654	BP	GO:0060149
655	BP	GO:0060967
656	BP	GO:1900373
657	BP	GO:1903077
658	BP	GO:2000679

659	BP	GO:2001026
660	BP	GO:0010466
661	BP	GO:0010996
662	BP	GO:0035902
663	BP	GO:0042730
664	BP	GO:0090312
665	BP	GO:1901623
666	BP	GO:1903055
667	BP	GO:1903798
668	BP	GO:1904385
669	BP	GO:1904996
670	BP	GO:1905563
671	BP	GO:0050708
672	BP	GO:0001963
673	BP	GO:0002092
674	BP	GO:0006027
675	BP	GO:0006706
676	BP	GO:0048668
677	BP	GO:0070920
678	BP	GO:0071425
679	BP	GO:1904376
680	BP	GO:0051962
681	BP	GO:1903829
682	BP	GO:0010894
683	BP	GO:0032967
684	BP	GO:0034698
685	BP	GO:0045830
686	BP	GO:0048873
687	BP	GO:0060142
688	BP	GO:0031349
689	BP	GO:1902105
690	BP	GO:0021700
691	BP	GO:1903532
692	BP	GO:0001945
693	BP	GO:0002474
694	BP	GO:0002675
695	BP	GO:0009065
696	BP	GO:0010714
697	BP	GO:0010818
698	BP	GO:0032801
699	BP	GO:0044331
700	BP	GO:0051894
701	BP	GO:1903672
702	BP	GO:0022409

703	BP	GO:0022011
704	BP	GO:0032292
705	BP	GO:0048679
706	BP	GO:0060969
707	BP	GO:0071549
708	BP	GO:0090025
709	BP	GO:1902175
710	BP	GO:2000406
711	BP	GO:0034504
712	BP	GO:0060485
713	BP	GO:0008209
714	BP	GO:0010800
715	BP	GO:0034368
716	BP	GO:0034369
717	BP	GO:0043304
718	BP	GO:0045939
719	BP	GO:0045940
720	BP	GO:0051968
721	BP	GO:0060218
722	BP	GO:0061311
723	BP	GO:2000637
724	BP	GO:0010720
725	BP	GO:0001516
726	BP	GO:0030212
727	BP	GO:0031063
728	BP	GO:0033006
729	BP	GO:0043171
730	BP	GO:0045987
731	BP	GO:0046457
732	BP	GO:0050482
733	BP	GO:0060055
734	BP	GO:0060148
735	BP	GO:0060390
736	BP	GO:0070528
737	BP	GO:0070723
738	BP	GO:0090183
739	BP	GO:1900027
740	BP	GO:1903963
741	BP	GO:0006913
742	BP	GO:0030198
743	BP	GO:0042063
744	BP	GO:0051169
745	BP	GO:0043062
746	BP	GO:0007162

747	BP	GO:0051222
748	BP	GO:0006026
749	BP	GO:0007435
750	BP	GO:0010039
751	BP	GO:0010743
752	BP	GO:0014044
753	BP	GO:0019835
754	BP	GO:0034367
755	BP	GO:0035767
756	BP	GO:0048841
757	BP	GO:0090022
758	BP	GO:1900745
759	BP	GO:1901889
760	BP	GO:1905476
761	BP	GO:0045229
762	BP	GO:0002440
763	BP	GO:0010165
764	BP	GO:0045920
765	BP	GO:0046685
766	BP	GO:0060122
767	BP	GO:0070570
768	BP	GO:0150117
769	BP	GO:1902692
770	BP	GO:0022604
771	BP	GO:0070372
772	BP	GO:0051047
773	BP	GO:0001662
774	BP	GO:0002446
775	BP	GO:0007431
776	BP	GO:0016242
777	BP	GO:0034383
778	BP	GO:0051482
779	BP	GO:0090075
780	BP	GO:2000352
781	BP	GO:0001569
782	BP	GO:0002209
783	BP	GO:0042462
784	BP	GO:0045454
785	BP	GO:0090181
786	BP	GO:0097421
787	BP	GO:0098801
788	BP	GO:1905207
789	BP	GO:2000403
790	BP	GO:0007204

791	BP	GO:1901214
792	BP	GO:1904951
793	BP	GO:0043010
794	BP	GO:0002691
795	BP	GO:0003203
796	BP	GO:0010762
797	BP	GO:0030224
798	BP	GO:0036314
799	BP	GO:0045191
800	BP	GO:0007517
801	BP	GO:0016202
802	BP	GO:0032570
803	BP	GO:0032885
804	BP	GO:0048846
805	BP	GO:0071542
806	BP	GO:1902284
807	BP	GO:1905332
808	BP	GO:0050863
809	BP	GO:0070371
810	BP	GO:0035886
811	BP	GO:0042491
812	BP	GO:0042596
813	BP	GO:1903037
814	BP	GO:0032386
815	BP	GO:0045923
816	BP	GO:0071548
817	BP	GO:1901861
818	BP	GO:0002714
819	BP	GO:0002891
820	BP	GO:0010863
821	BP	GO:0014037
822	BP	GO:0030501
823	BP	GO:0045777
824	BP	GO:0051281
825	BP	GO:0150077
826	BP	GO:1904994
827	BP	GO:0048634
828	BP	GO:0071470
829	BP	GO:0071634
830	BP	GO:1900371
831	BP	GO:1902622
832	BP	GO:0006936
833	BP	GO:0043087
834	BP	GO:0002449

835	BP	GO:0007520
836	BP	GO:0030808
837	BP	GO:0032965
838	BP	GO:0033003
839	BP	GO:0033574
840	BP	GO:0042168
841	BP	GO:0045687
842	BP	GO:0045773
843	BP	GO:0071312
844	BP	GO:1900274
845	BP	GO:1902895
846	BP	GO:2000404
847	BP	GO:0045861
848	BP	GO:0051480
849	BP	GO:0045776
850	BP	GO:0045981
851	BP	GO:0071604
852	BP	GO:1900544
853	BP	GO:1904706
854	BP	GO:0002460
855	BP	GO:0001818
856	BP	GO:0009306
857	BP	GO:1901653
858	BP	GO:0031295
859	BP	GO:0032620
860	BP	GO:0032660
861	BP	GO:0032881
862	BP	GO:0045124
863	BP	GO:0097178
864	BP	GO:0150076
865	BP	GO:0035592
866	BP	GO:0070997
867	BP	GO:0051251
868	BP	GO:0003197
869	BP	GO:0008631
870	BP	GO:0031670
871	BP	GO:0032309
872	BP	GO:0035272
873	BP	GO:0035307
874	BP	GO:0045933
875	BP	GO:0048538
876	BP	GO:0060119
877	BP	GO:0071354
878	BP	GO:0085029

879	BP	GO:1904646
880	BP	GO:1903706
881	BP	GO:0071692
882	BP	GO:0007157
883	BP	GO:0031294
884	BP	GO:0035094
885	BP	GO:0043300
886	BP	GO:0050919
887	BP	GO:0055010
888	BP	GO:0060976
889	BP	GO:0071827
890	BP	GO:0001654
891	BP	GO:0007159
892	BP	GO:0001754
893	BP	GO:0007595
894	BP	GO:0010712
895	BP	GO:0010761
896	BP	GO:0010799
897	BP	GO:0035315
898	BP	GO:0045601
899	BP	GO:0045747
900	BP	GO:0045911
901	BP	GO:0046677
902	BP	GO:0101023
903	BP	GO:1900744
904	BP	GO:1905562
905	BP	GO:0150063
906	BP	GO:0002673
907	BP	GO:0033628
908	BP	GO:0043303
909	BP	GO:0045912
910	BP	GO:0090311
911	BP	GO:0051346
912	BP	GO:0030099
913	BP	GO:0048880
914	BP	GO:0002204
915	BP	GO:0002208
916	BP	GO:0002279
917	BP	GO:0006692
918	BP	GO:0006693
919	BP	GO:0006778
920	BP	GO:0006953
921	BP	GO:0009409
922	BP	GO:0010862

923	BP	GO:0018198
924	BP	GO:0030857
925	BP	GO:0034332
926	BP	GO:0042304
927	BP	GO:0042461
928	BP	GO:0045190
929	BP	GO:0045778
930	BP	GO:0060964
931	BP	GO:0070169
932	BP	GO:0070741
933	BP	GO:0072091
934	BP	GO:1904894
935	BP	GO:0014706
936	BP	GO:0045786
937	BP	GO:0002448
938	BP	GO:0002639
939	BP	GO:0008089
940	BP	GO:0050873
941	BP	GO:0060688
942	BP	GO:0071825
943	BP	GO:0110151
944	BP	GO:1902930
945	BP	GO:2000677
946	BP	GO:0006636
947	BP	GO:0010656
948	BP	GO:0032964
949	BP	GO:0045104
950	BP	GO:0046850
951	BP	GO:0051339
952	BP	GO:0071622
953	BP	GO:0010718
954	BP	GO:0031103
955	BP	GO:0035196
956	BP	GO:0043392
957	BP	GO:0045103
958	BP	GO:0048260
959	BP	GO:0060147
960	BP	GO:0072132
961	BP	GO:0006163
962	BP	GO:0031279
963	BP	GO:0034381
964	BP	GO:0060966
965	BP	GO:0071715
966	BP	GO:0010524

967	BP	GO:0031050
968	BP	GO:0070918
969	BP	GO:1902893
970	BP	GO:2000179
971	BP	GO:0060537
972	BP	GO:0000768
973	BP	GO:0003229
974	BP	GO:0038066
975	BP	GO:0061098
976	BP	GO:0061614
977	BP	GO:0140253
978	BP	GO:0002696
979	BP	GO:0001658
980	BP	GO:0002090
981	BP	GO:0002886
982	BP	GO:0010332
983	BP	GO:0031529
984	BP	GO:0043388
985	BP	GO:0045599
986	BP	GO:0046456
987	BP	GO:0055078
988	BP	GO:0071385
989	BP	GO:1904645
990	BP	GO:0006611
991	BP	GO:0006695
992	BP	GO:0006754
993	BP	GO:0006949
994	BP	GO:0016447
995	BP	GO:0033619
996	BP	GO:0051155
997	BP	GO:0061756
998	BP	GO:1902653
999	BP	GO:0072521
1000	BP	GO:0001954
1001	BP	GO:0002763
1002	BP	GO:0007405
1003	BP	GO:0042743
1004	BP	GO:2001244
1005	BP	GO:0007409
1006	BP	GO:0034329
1007	BP	GO:0050867
1008	BP	GO:0019369
1009	BP	GO:0033013
1010	BP	GO:0035306

1011	BP	GO:0051055
1012	BP	GO:0051353
1013	BP	GO:2000351
1014	BP	GO:0072594
1015	CC	GO:0045121
1016	CC	GO:0098857
1017	CC	GO:0009897
1018	CC	GO:0005901
1019	CC	GO:0044853
1020	CC	GO:0031093
1021	CC	GO:0031091
1022	CC	GO:0005925
1023	CC	GO:0030055
1024	CC	GO:0062023
1025	CC	GO:0031968
1026	CC	GO:0019867
1027	CC	GO:0016327
1028	CC	GO:0031258
1029	CC	GO:0031233
1030	CC	GO:0031045
1031	CC	GO:0005640
1032	CC	GO:0005788
1033	CC	GO:0032839
1034	CC	GO:0034774
1035	CC	GO:0060205
1036	CC	GO:0031983
1037	CC	GO:0030139
1038	CC	GO:0001772
1039	MF	GO:0005125
1040	MF	GO:0048018
1041	MF	GO:0030546
1042	MF	GO:0020037
1043	MF	GO:0046906
1044	MF	GO:0016705
1045	MF	GO:0004175
1046	MF	GO:0042056
1047	MF	GO:0008236
1048	MF	GO:0017171
1049	MF	GO:0050840
1050	MF	GO:0005126
1051	MF	GO:0001618
1052	MF	GO:0001664
1053	MF	GO:0140272
1054	MF	GO:0016209

1055	MF	GO:0004497
1056	MF	GO:0008238
1057	MF	GO:0002020
1058	MF	GO:0005178
1059	MF	GO:0008083
1060	MF	GO:0008239
1061	MF	GO:0034713
1062	MF	GO:0070008
1063	MF	GO:0008201
1064	MF	GO:0008603
1065	MF	GO:0031434
1066	MF	GO:0004252
1067	MF	GO:0031404
1068	MF	GO:0005172
1069	MF	GO:0005161
1070	MF	GO:0016805
1071	MF	GO:0005159
1072	MF	GO:0010181
1073	MF	GO:0045236
1074	MF	GO:0005539
1075	MF	GO:0016702
1076	MF	GO:0016701
1077	MF	GO:0005160
1078	MF	GO:0051019
1079	MF	GO:1901681
1080	MF	GO:0004190
1081	MF	GO:0045499
1082	MF	GO:0001968
1083	MF	GO:0070001
1084	MF	GO:0003785
1085	MF	GO:0004177
1086	MF	GO:0016712
1087	MF	GO:0004180
1088	MF	GO:0016709
1089	MF	GO:0008009
1090	MF	GO:0004601
1091	MF	GO:0016597
1092	MF	GO:0050661
1093	MF	GO:0016684
