# Supplementary material for: Niaoduqing alleviates podocyte injury in high glucose model via regulating multiple targets and AGE/RAGE pathway: Network pharmacology and experimental validation
Source: Front Pharmacol. 2023 Feb 27;14:1047184. doi: 10.3389/fphar.2023.1047184 (PMC10009170; doi:10.3389/fphar.2023.1047184)
Supplement: Supplementary file 4 [file Table1.pdf]

Table S1 All active ingredients of Niaoduqing

Components	Ingredients	MW	AlogP	Hdon	Hacc	OB (%)	Caco-2	BBB	DL	FASA-	HL
Ku Shen,Dan Shen,Che Qian Cao	MOL000006	286.25	2.07	4	6	36.16	0.19	-0.84	0.25	0.39	15.94
Sang Bai Pi,Ku Shen,Huang Qi	MOL000098	302.25	1.5	5	7	46.43	0.05	-0.77	0.28	0.38	14.4
Sang Bai Pi,Huang Qi,Bai Shao	MOL000211	456.78	6.52	2	3	55.38	0.73	0.22	0.78	0.26	8.87
Sang Bai Pi,Da Huang,Bai Shao	MOL000358	414.79	8.08	1	1	36.91	1.32	0.99	0.75	0.23	5.36
Sang Bai Pi,Huang Qi,Bai Shao	MOL000422	286.25	1.77	4	6	41.88	0.26	-0.55	0.24	0	14.74
Huang Qi,Bai Shu	MOL000033	428.82	8.54	1	1	36.23	1.45	1.09	0.78	0	5.22
Huang Qi,Fu Ling	MOL000296	414.79	8.08	1	1	36.91	1.32	0.96	0.75	0	5.35
Che Qian Cao,Bai Shao	MOL000359	414.79	8.08	1	1	36.91	1.32	0.87	0.75	0.22	5.37
Ku Shen,Huang Qi	MOL000392	268.28	2.58	1	4	69.67	0.78	0.02	0.21	0	17.04
Sang Bai Pi,Da Huang	MOL000554	484.4	-0.03	9	14	30.25	-1.96	-2.76	0.67	0.36	2.48
Dan Shen,Che Qian Cao	MOL002776	446.39	0.64	6	11	40.12	-0.85	-1.74	0.75	0.36	17.36
Bai Shu	MOL000020	312.39	2.5	0	4	62.4	0.01	-1.37	0.22	0.12	6.07
Bai Shu	MOL000021	355.44	3.21	0	5	60.31	0.33	-1.09	0.31	0.05	5.32
Bai Shu	MOL000022	356.45	3.54	1	5	63.37	0.42	-1.14	0.3	0	6.43
Bai Shu	MOL000028	426.8	7.35	1	1	39.51	1.42	1.28	0.76	0	3.83
Bai Shu	MOL000049	274.39	3.39	0	3	54.07	1.13	1.08	0.22	0	-1.31
Bai Shu	MOL000072	276.41	3.68	0	3	35.95	1.08	1.12	0.21	0	8.34
Da Huang	MOL000096	290.29	1.92	5	6	49.68	-0.03	-0.78	0.24	0.35	0.38
Huang Qi	MOL000239	314.31	2.09	2	6	50.83	0.61	-0.22	0.29	0.29	15.5
Fu Ling	MOL000273	470.76	5.41	3	4	30.93	0.01	-0.76	0.81	0	6.81
Fu Ling	MOL000275	456.78	7.03	2	3	38.71	0.52	-0.14	0.8	0	7.78
Fu Ling	MOL000276	526.83	6.1	2	5	35.11	0.03	-0.87	0.81	0	7.34
Fu Ling	MOL000279	430.74	5.15	3	3	37.96	0.28	-0.39	0.77	0	5.31
Fu Ling	MOL000280	484.79	5.72	3	4	31.07	0.05	-0.7	0.82	0	7.42
Fu Ling	MOL000282	398.74	7.18	1	1	43.51	1.32	0.91	0.72	0	5.11
Fu Ling	MOL000283	430.74	7.17	1	3	40.36	0.84	0.34	0.81	0	3.43
Fu Ling	MOL000285	482.77	5.68	2	4	38.26	0.12	-0.57	0.82	0	6.77
Fu Ling	MOL000287	470.81	7.33	2	3	38.7	0.61	-0.04	0.81	0	6.59
Fu Ling	MOL000289	528.85	6.54	2	5	33.63	0.1	-0.57	0.81	0	9.27
Fu Ling	MOL000290	498.77	5.94	3	5	30.61	-0.14	-0.93	0.76	0	8.26
Fu Ling	MOL000291	484.74	5.64	3	5	30.52	-0.08	-0.87	0.75	0	8.67
Fu Ling	MOL000292	482.77	7.11	2	4	38.15	0.32	-0.41	0.75	0	7.73
Fu Ling	MOL000300	453.75	6.35	1	3	44.17	0.38	-0.16	0.83	0.04	7.04
Huang Qi	MOL000354	316.28	1.76	4	7	49.6	0.31	-0.54	0.31	0.32	14.34
Huang Qi	MOL000371	314.36	2.89	0	5	53.74	1.18	0.63	0.48	0	9
Huang Qi	MOL000374	642.67	-0.95	9	16	41.72	-2.47	-3.62	0.69	0	2.52
Huang Qi	MOL000378	316.38	3.38	1	5	74.69	1.08	0.84	0.3	0	2.98
Huang Qi	MOL000379	462.49	0.74	4	10	36.74	-0.63	-1.5	0.92	0	13.06
Huang Qi	MOL000380	300.33	2.64	1	5	64.26	0.93	0.55	0.42	0	8.49
Huang Qi	MOL000387	418.38	2.56	0	10	31.1	0.15	-0.06	0.67	0	17.96
Huang Qi	MOL000398	316.33	2.42	2	6	109.99	0.53	0.17	0.3	0	15.51
Huang Qi	MOL000417	284.28	2.32	2	5	47.75	0.52	-0.43	0.24	0	17.1

Huang Qi	MOL000433	441.45	0.01	7	13	68.96	-1.5	-2.59	0.71	0	24.81
Huang Qi	MOL000438	302.35	3.13	2	5	67.67	0.96	0.34	0.26	0	2.9
Huang Qi	MOL000439	626.67	-0.68	8	15	49.28	-2.22	-3.36	0.62	0	0.93
Huang Qi	MOL000442	314.31	3.11	2	6	39.05	0.89	-0.04	0.48	0	7.95
Che Qian Cao	MOL000449	412.77	7.64	1	1	43.83	1.44	1	0.76	0.22	5.57
Ku Shen	MOL000456	322.38	3.46	1	4	78.2	1.09	0.39	0.73	0.33	7.56
Da Huang	MOL000471	270.25	1.67	3	5	83.38	-0.12	-1.07	0.24	0	31.49
Bai Shao	MOL000492	290.29	1.92	5	6	54.83	-0.03	-0.73	0.24	0	0.61
Dan Shen	MOL000569	322.24	1.53	6	9	61.85	-0.76	-1.52	0.26	0.43	5.29
Sang Bai Pi	MOL001004	271.26	1.93	4	5	37.99	0.31	-0.33	0.21	0.38	0.48
Ku Shen	MOL001040	272.27	2.3	3	5	42.36	0.38	-0.48	0.21	0.41	16.83
Sang Bai Pi	MOL001474	332.35	3.47	0	4	37.81	1.26	0.15	0.86	0.3	7.84
Ku Shen	MOL001484	284.28	2.44	1	5	75.18	0.89	0.4	0.54	0.3	11.72
Dan Shen	MOL001601	280.34	2.98	0	3	38.75	0.96	0.39	0.36	0.33	18.05
Dan Shen	MOL001659	412.77	7.64	1	1	43.83	1.44	1.03	0.76	0.22	5.34
Che Qian Cao	MOL001735	300.28	2.32	3	6	30.97	0.48	-0.49	0.27	0.28	16.44
Dan Shen	MOL001771	414.79	8.08	1	1	36.91	1.45	1.14	0.75	0	5.07
Bai Shao	MOL001910	470.71	3.91	2	5	64.77	0.09	-0.59	0.38	0.27	2.62
Bai Shao	MOL001918	318.35	0.79	1	6	87.59	-0.09	-0.56	0.37	0.37	7.45
Bai Shao	MOL001919	358.52	2.69	2	4	43.56	0	-0.41	0.53	0.35	4.34
Bai Shao	MOL001921	462.49	-0.57	3	10	49.12	-1.13	-1.76	0.8	0.34	7.26
Bai Shao	MOL001924	480.51	-1.28	5	11	53.87	-1.47	-1.86	0.79	0.34	13.88
Bai Shao	MOL001925	318.35	0.46	2	6	68.18	-0.34	-0.73	0.4	0.39	8.81
Bai Shao	MOL001928	318.35	0.42	2	6	66.64	-0.49	-0.88	0.33	0.41	6.54
Bai Shao	MOL001930	584.62	0.81	4	12	31.27	-0.69	-1.24	0.75	0.29	-1.85
Dan Shen	MOL001942	270.3	3.65	0	4	45.46	0.97	0.66	0.23	0.27	-1.44
Dan Shen	MOL002222	300.48	4.99	1	2	36.11	1.14	0.7	0.28	0.27	14.62
Da Huang	MOL002235	360.34	1.99	3	8	50.8	0.53	-0.26	0.41	0.22	13.94
Da Huang	MOL002251	552.96	10.9	0	1	48.64	1.97	0.84	0.61	0.32	15.73
Da Huang	MOL002259	608.6	-0.91	8	15	41.65	-2.64	-3.43	0.63	0.3	27.61
Da Huang	MOL002260	730.67	4.6	12	16	31.99	-1.61	-2.88	0.32	0.39	5.98
Da Huang	MOL002268	284.23	1.88	3	6	47.07	-0.2	-0.99	0.28	0.47	32.12
Da Huang	MOL002276	524.5	3.91	6	9	50.69	-0.74	-1.56	0.61	0.37	33.6
Da Huang	MOL002280	480.46	0.64	5	12	43.02	-1.23	-1.84	0.74	0.32	16.29
Da Huang	MOL002281	272.27	2.25	2	5	46.46	0.86	0.37	0.24	0.35	3.55
Da Huang	MOL002288	432.41	0.59	6	10	44.81	-1.12	-2	0.8	0.34	29.79
Da Huang	MOL002293	524.5	3.91	6	9	61.06	-0.7	-1.46	0.61	0.39	33.92
Da Huang	MOL002297	386.73	7.67	1	1	35.89	1.35	1.07	0.7	0.2	6.12
Da Huang	MOL002303	510.52	4.52	6	8	32.45	-0.36	-1.47	0.65	0.4	32.14
Sang Bai Pi	MOL002514	316.28	1.76	4	7	62.86	0.31	-0.5	0.3	0.32	15.19
Dan Shen	MOL002651	292.35	4.22	0	3	43.76	1.02	0.52	0.4	0.33	23.71
Che Qian Cao	MOL002714	270.25	2.33	3	5	33.52	0.63	-0.05	0.21	0.36	16.25
Ku Shen	MOL003347	536.87	8.62	1	4	44.03	0.87	0.4	0.6	0	2.15
Ku Shen	MOL003542	354.38	3.63	4	6	38.04	0.53	-0.49	0.39	0	15.37

Ku Shen	MOL003627	246.39	1.39	0	3	64.26	0.99	1	0.25	0.26	5.54
Ku Shen	MOL003648	284.28	2.44	1	5	65.83	0.91	0.36	0.54	0.3	11.73
Ku Shen	MOL003673	338.38	3.92	3	5	42.8	0.64	-0.16	0.36	0.31	17.04
Ku Shen	MOL003676	244.37	1.15	0	3	42.16	1.43	1.53	0.25	0.22	6.69
Ku Shen	MOL003680	248.41	1.42	0	3	60.07	1.13	1.14	0.25	0.18	5.57
Sang Bai Pi	MOL003758	330.31	2.03	3	7	71.55	0.55	-0.16	0.34	0.23	16.32
Sang Bai Pi	MOL003856	286.3	3.38	2	5	55.85	0.83	-0.09	0.23	0.24	5.3
Sang Bai Pi	MOL003857	310.37	5	3	4	82.13	0.87	-0.07	0.29	0.31	9.13
Sang Bai Pi	MOL003858	308.35	4.2	2	4	60.93	1.03	0.12	0.38	0.32	6.44
Sang Bai Pi	MOL003860	286.3	3.38	2	5	53.81	0.81	-0.02	0.23	0.26	4.84
Che Qian Cao	MOL004004	302.25	1.8	5	7	46.93	0.14	-0.91	0.28	0.35	14.62
Ku Shen	MOL004580	304.27	1.49	5	7	66.44	-0.34	-1.11	0.27	0.4	14.51
Sang Bai Pi	MOL004912	336.36	3.12	2	5	52.51	0.59	-0.11	0.5	0	16.09
Ku Shen	MOL004941	256.27	2.57	2	4	71.12	0.41	-0.25	0.18	0	18.09
Sang Bai Pi	MOL005043	400.76	7.63	1	1	37.58	1.32	0.94	0.71	0.23	4.43
Ku Shen	MOL005100	302.3	2.28	3	6	47.74	0.28	-0.3	0.27	0.31	16.51
Ku Shen	MOL005944	248.41	1.42	0	3	63.77	1.39	1.52	0.25	0	6.69
Ku Shen	MOL006561	264.41	0.74	1	4	35.73	0.53	0.48	0.29	0.21	3.01
Ku Shen	MOL006562	246.39	1.42	0	3	62.08	1.06	1.12	0.25	0.16	6.46
Ku Shen	MOL006563	264.41	0.45	1	4	32.04	0.61	0.15	0.29	0.19	4.55
Ku Shen	MOL006564	248.41	1.42	0	3	58.87	1.08	1.13	0.25	0.18	5.49
Ku Shen	MOL006565	248.41	1.42	0	3	68.68	1.15	1.38	0.25	0.17	6.15
Ku Shen	MOL006566	246.39	1.11	0	3	58.34	1.21	1.36	0.25	0.21	6.57
Ku Shen	MOL006568	246.39	1.39	0	3	61.57	1.39	1.45	0.25	0.24	5.9
Ku Shen	MOL006569	264.41	0.74	1	4	37.26	0.77	0.77	0.29	0.21	3.09
Ku Shen	MOL006570	262.39	0.43	1	4	35.23	0.38	-0.12	0.29	0.25	4.28
Ku Shen	MOL006571	244.37	1.15	0	3	62.01	1.16	1.13	0.24	0	4.74
Ku Shen	MOL006572	348.64	5.4	2	3	34.64	1.13	0.82	0.24	0.23	5.62
Ku Shen	MOL006573	246.39	1.39	0	3	65.34	1.06	1.11	0.25	0.24	6.24
Ku Shen	MOL006582	280.41	-0.3	2	5	40.93	0.04	-0.43	0.32	0.2	4.59
Ku Shen	MOL006583	246.39	1.42	0	3	44.43	1.11	1.13	0.25	0.16	5.22
Ku Shen	MOL006596	338.38	2.85	2	5	97.27	0.53	-0.19	0.76	0.35	6.24
Ku Shen	MOL006604	354.43	4.41	2	5	48.09	0.8	0	0.39	0.3	15.54
Ku Shen	MOL006613	286.3	2.39	2	5	47.62	0.71	0.35	0.38	0.27	8.86
Ku Shen	MOL006619	580.59	-0.87	7	14	51.39	-2.2	-3.12	0.74	0.31	13.81
Ku Shen	MOL006620	286.3	2.27	2	5	50.86	0.24	-0.27	0.24	0.37	15.78
Ku Shen	MOL006622	562.57	-0.56	6	13	42.41	-1.67	-2.25	0.76	0.29	15.73
Ku Shen	MOL006623	442.55	4.46	5	7	51.28	-0.05	-0.95	0.64	0.35	17.47
Ku Shen	MOL006626	356.4	3.89	4	6	60.97	0.33	-0.36	0.4	0.37	15.53
Ku Shen	MOL006627	246.39	1.11	0	3	62.23	1.18	1.25	0.25	0.21	6
Ku Shen	MOL006628	248.41	1.42	0	3	52.71	1.16	1.19	0.24	0.18	4.25
Ku Shen	MOL006630	286.25	2.07	4	6	54.93	0.14	-0.74	0.24	0.42	17.23
Ku Shen	MOL006649	264.41	0.67	1	4	55.42	0.6	0.68	0.28	0.19	4.94
Ku Shen	MOL006650	532.49	0.7	4	13	48.69	-1.45	-2.14	0.52	0.34	20.42

Ku Shen	MOL006652	462.44	0.95	4	11	48.53	-0.85	-1.65	0.74	0.24	18.51
Dan Shen	MOL006824	426.8	7.35	1	1	39.51	1.37	1.2	0.76	0.23	3.06
Dan Shen	MOL007036	298.41	4.38	2	3	33.77	1.19	0.8	0.29	0.29	14.91
Dan Shen	MOL007041	264.34	4.16	0	2	40.86	1.23	0.81	0.23	0.43	14.89
Dan Shen	MOL007045	310.37	3.56	1	4	44.93	0.53	0.22	0.44	0.3	23.78
Dan Shen	MOL007048	312.29	3.21	4	6	48.24	0.18	-0.89	0.31	0.4	8.87
Dan Shen	MOL007049	266.36	4.33	0	2	34.35	1.25	0.87	0.23	0.38	14.6
Dan Shen	MOL007050	356.4	3.58	2	6	62.78	0.35	-0.73	0.4	0.24	7.89
Dan Shen	MOL007051	628.64	-1.13	5	16	46.69	-1.73	-2.08	0.71	0.22	9.94
Dan Shen	MOL007058	290.28	3.36	0	4	73.44	0.54	-0.28	0.42	0.41	24.12
Dan Shen	MOL007059	294.32	3.16	1	4	32.16	0.38	-0.48	0.41	0.36	22.51
Dan Shen	MOL007061	278.32	4.26	0	3	37.07	1.03	0.46	0.36	0.36	24.33
Dan Shen	MOL007063	398.49	2.25	1	6	37.11	-0.26	-0.69	0.65	0.38	1.63
Dan Shen	MOL007064	330.46	3.18	1	4	110.32	0.34	0.22	0.44	0.32	2.17
Dan Shen	MOL007068	292.3	2.99	1	4	62.24	0.39	-0.45	0.41	0.38	24.94
Dan Shen	MOL007069	296.34	3.31	1	4	55.74	0.42	-0.3	0.4	0.32	23.7
Dan Shen	MOL007070	312.34	2.34	2	5	41.31	-0.06	-0.68	0.45	0.32	22.54
Dan Shen	MOL007071	312.34	2.07	2	5	40.31	-0.09	-0.9	0.46	0.29	22.45
Dan Shen	MOL007077	308.56	4.27	2	2	43.67	0.84	0.51	0.21	0.27	4.71
Dan Shen	MOL007079	308.35	3.83	0	4	52.47	0.57	-0.07	0.45	0.32	23.49
Dan Shen	MOL007081	354.48	2.59	1	4	57.95	0.53	0.11	0.56	0.3	4.28
Dan Shen	MOL007082	336.41	2.01	1	4	56.97	0.33	-0.01	0.52	0.34	5.15
Dan Shen	MOL007085	292.4	4.26	0	2	30.38	1.46	1.07	0.38	0.35	20.81
Dan Shen	MOL007088	296.39	3.44	0	3	52.34	0.95	0.51	0.4	0.29	17.3
Dan Shen	MOL007093	336.41	2.83	1	4	38.88	0.67	-0.15	0.55	0.35	30
Dan Shen	MOL007094	282.36	3.24	0	3	50.43	0.88	0.51	0.31	0.34	15.19
Dan Shen	MOL007098	298.41	4.32	1	3	49.4	0.85	0.24	0.29	0.3	27.17
Dan Shen	MOL007100	266.31	2.77	0	3	38.68	1.26	0.81	0.32	0.38	5.42
Dan Shen	MOL007101	278.32	2.86	0	3	45.04	0.95	0.43	0.36	0.4	18.32
Dan Shen	MOL007105	284.38	2.37	0	3	68.27	0.9	0.61	0.31	0.33	1.77
Dan Shen	MOL007107	286.5	5.98	1	1	36.07	1.63	1.54	0.25	0.25	-0.16
Dan Shen	MOL007108	296.39	3.59	0	3	54.98	0.93	0.34	0.39	0.3	31.92
Dan Shen	MOL007111	294.37	4.66	0	3	49.92	1.03	0.45	0.4	0.3	24.73
Dan Shen	MOL007115	304.57	5.5	1	1	45.04	1.28	1.16	0.2	0.28	5.81
Dan Shen	MOL007118	298.46	4.75	1	2	39.61	1.05	0.99	0.28	0.33	4.52
Dan Shen	MOL007119	312.39	3.33	1	4	49.68	0.35	-0.11	0.32	0.35	41.49
Dan Shen	MOL007120	312.39	2.14	1	4	71.03	0.62	0.03	0.44	0.28	2.91
Dan Shen	MOL007121	300.43	2.74	1	3	36.56	0.5	0.17	0.37	0.3	1.7
Dan Shen	MOL007122	282.41	4.73	0	2	38.76	1.23	0.87	0.25	0.32	14.82
Dan Shen	MOL007123	272.32	0.77	1	4	44.95	0.04	-0.25	0.24	0.35	2.24
Dan Shen	MOL007124	270.35	3.61	1	3	39.46	0.76	0.16	0.23	0.32	26.98
Dan Shen	MOL007125	314.41	3.01	2	4	52.49	0.35	-0.13	0.32	0.28	14.46
Dan Shen	MOL007127	280.29	3.21	0	4	34.72	0.5	-0.27	0.37	0.33	37.89
Dan Shen	MOL007130	314.31	2.77	4	6	64.37	0.1	-0.75	0.31	0.42	8.82

Dan Shen	MOL007132	360.34	2.69	5	8	109.38	-0.33	-1.02	0.35	0.41	2.01
Dan Shen	MOL007140	314.31	2.82	5	6	88.54	-0.09	-0.77	0.26	0.43	4.31
Dan Shen	MOL007141	340.3	2.2	4	7	45.56	-0.14	-0.97	0.61	0.45	2.4
Dan Shen	MOL007142	538.49	3.78	6	12	43.38	-0.82	-2.14	0.72	0.44	5.77
Dan Shen	MOL007143	270.4	2.88	1	2	32.43	1.13	0.77	0.23	0.3	1
Dan Shen	MOL007145	268.38	4.05	1	2	31.72	1.04	0.72	0.24	0.36	0.33
Dan Shen	MOL007149	300.48	4.99	1	2	34.49	1.08	0.63	0.28	0.29	14.56
Dan Shen	MOL007150	312.34	2.42	2	5	75.39	0.03	-0.74	0.46	0.29	23.45
Dan Shen	MOL007151	312.34	2.34	2	5	42.67	0.05	-0.63	0.45	0.33	22.25
Dan Shen	MOL007152	312.34	2.34	2	5	42.85	-0.04	-0.65	0.45	0.32	22.44
Dan Shen	MOL007154	294.37	4.66	0	3	49.89	1.05	0.7	0.4	0.31	23.56
Dan Shen	MOL007155	310.37	3.57	1	4	65.26	0.44	-0.31	0.45	0.29	23.48
Dan Shen	MOL007156	296.34	2.44	2	4	45.64	0.48	-0.28	0.3	0.38	15.21
Che Qian Cao	MOL007783	450.48	-0.41	5	10	57.5	-0.83	-0.89	0.8	0.3	13.07
Che Qian Cao	MOL007796	651.23	14.62	0	2	38.09	1.42	0.6	0.4	0.19	8.33
Che Qian Cao	MOL007799	653.25	15.06	0	2	30.91	1.46	0.61	0.4	0.19	7.77
Sang Bai Pi	MOL009653	426.8	7.59	1	1	39.73	1.42	1.04	0.79	0.23	5.01
Sang Bai Pi	MOL012681	314.37	3.33	2	6	50.84	0.62	0.08	0.26	0.3	0.78
Sang Bai Pi	MOL012686	302.3	2.16	3	6	51.72	0.11	-0.57	0.26	0.34	16.16
Sang Bai Pi	MOL012689	418.47	4.58	2	6	36.79	0.86	-0.19	0.87	0.32	15.19
Sang Bai Pi	MOL012692	422.51	4	3	6	31.09	0.43	-0.2	0.8	0.33	24.51
Sang Bai Pi	MOL012714	286.3	3.38	2	5	64.39	0.84	-0.04	0.23	0.22	6.52
Sang Bai Pi	MOL012717	566.56	-0.67	9	14	37.81	-2.26	-3.85	0.74	0	12.26
Sang Bai Pi	MOL012719	326.37	3.54	3	5	62.33	0.52	-0.34	0.44	0.3	6.8
Sang Bai Pi	MOL012726	562.6	6.95	5	8	92.19	0.35	-0.75	0.24	0.34	9.34
Sang Bai Pi	MOL012735	326.37	3.54	3	5	71.39	0.41	-0.75	0.46	0.29	3.69
Sang Bai Pi	MOL012743	552.58	-0.79	9	13	35.08	-2.28	-3.86	0.76	0.34	3.38
Sang Bai Pi	MOL012749	570.63	5.75	5	9	115.44	-0.07	-1.07	0.3	0.35	28.97
Sang Bai Pi	MOL012753	354.38	3.09	3	6	62.42	0.48	-0.18	0.54	0.35	16.79
Sang Bai Pi	MOL012755	354.38	3.09	3	6	37.5	0.42	-0.28	0.53	0.35	18.92
Sang Bai Pi	MOL012760	436.49	4.4	3	7	68.29	-0.05	-0.65	0.85	0.36	23.83
Sang Bai Pi	MOL012800	286.25	1.77	4	6	59.71	0.25	-0.37	0.24	0.4	15.5
