# Supplementary figures and images for: Niaoduqing alleviates podocyte injury in high glucose model via regulating multiple targets and AGE/RAGE pathway: Network pharmacology and experimental validation
Source: Front Pharmacol. 2023 Feb 27;14:1047184. doi: 10.3389/fphar.2023.1047184 (PMC10009170; doi:10.3389/fphar.2023.1047184)

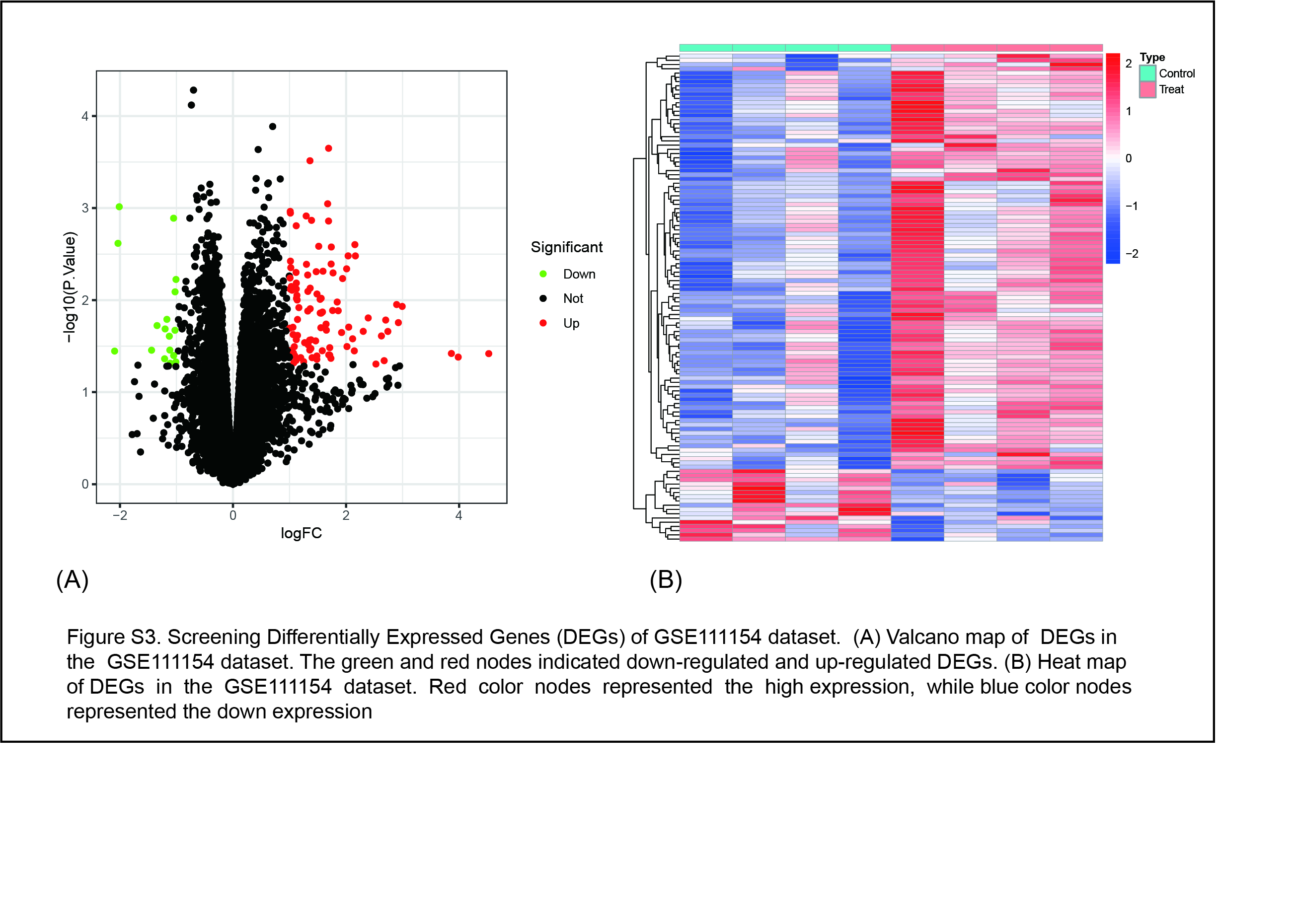

Supplement: Supplementary file 5 [file Image3.TIF]

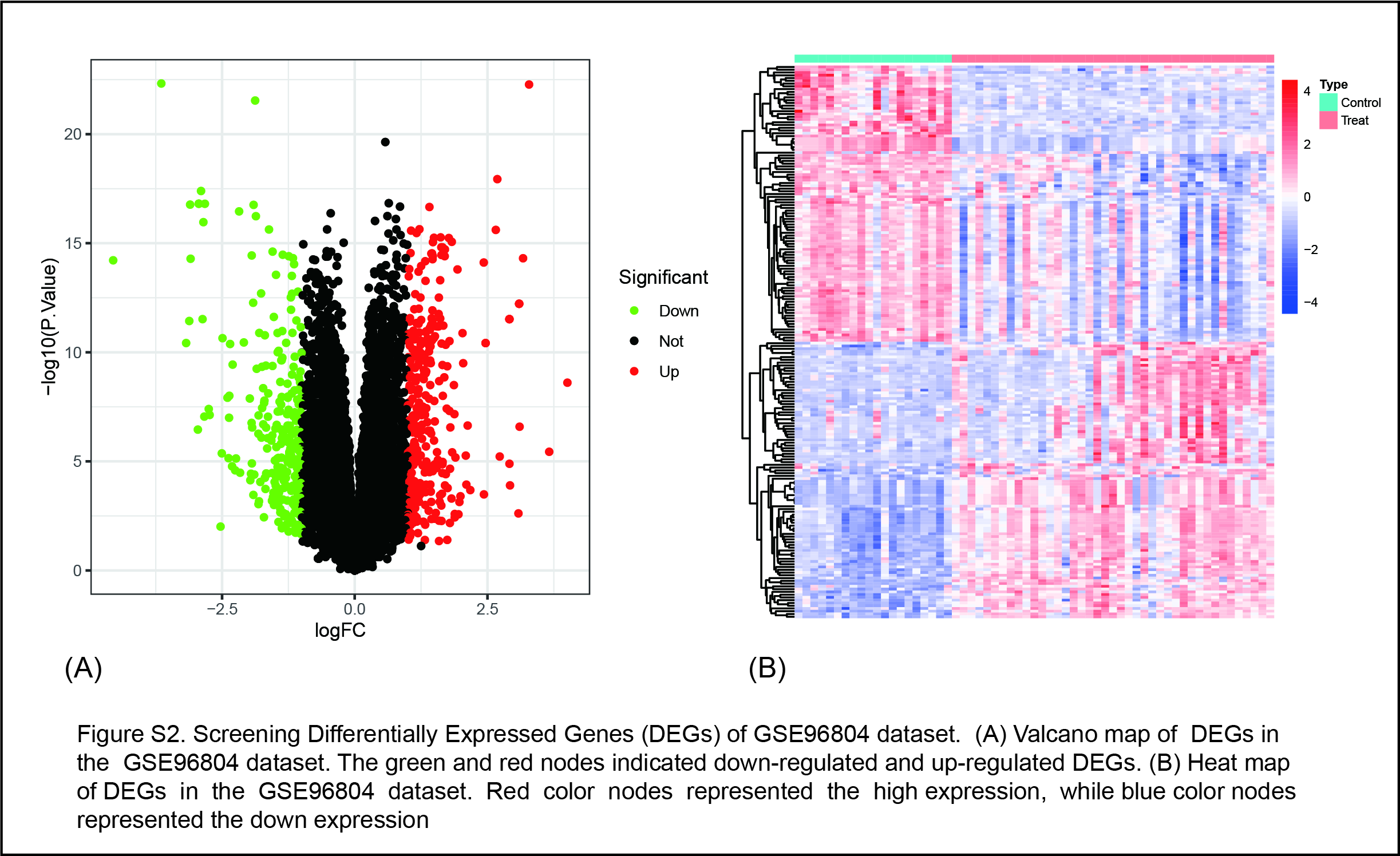

Supplement: Supplementary file 7 [file Image2.TIF]

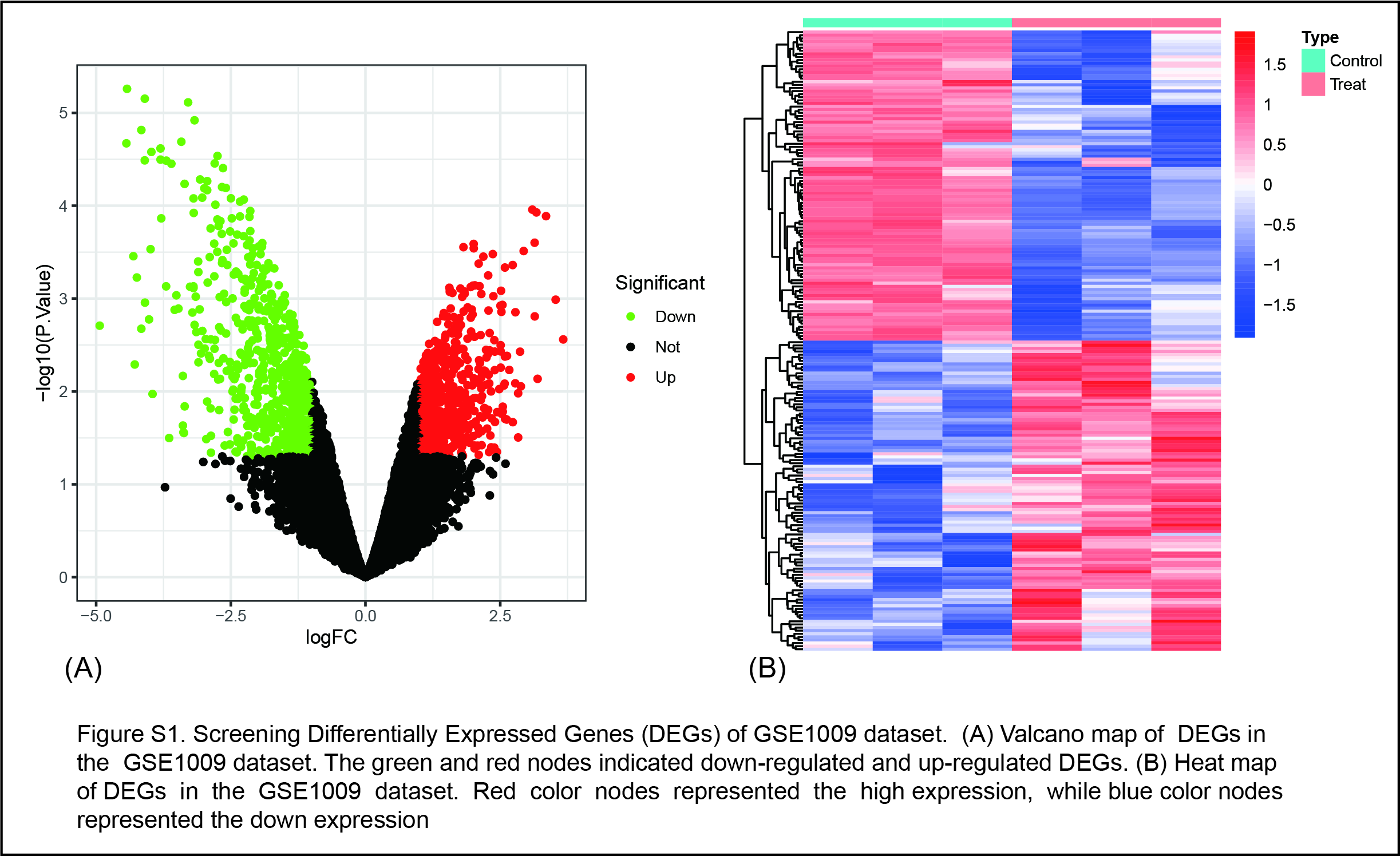

Supplement: Supplementary file 10 [file Image1.TIF]
