# Supplementary material for: Niaoduqing alleviates podocyte injury in high glucose model via regulating multiple targets and AGE/RAGE pathway: Network pharmacology and experimental validation
Source: Front Pharmacol. 2023 Feb 27;14:1047184. doi: 10.3389/fphar.2023.1047184 (PMC10009170; doi:10.3389/fphar.2023.1047184)
Supplement: Supplementary file 6 [file Table2.pdf]

Table S2 The source of predicted targets to each ingredients

Mol ID	TCMSP	Swiss Targets Prediction	Isomeric SMILES number
MOL000022	Yes	None	None
MOL000049	Yes	None	None
MOL000072	Yes	None	None
MOL000379	Yes	None	None
MOL000380	Yes	None	None
MOL000433	Yes	None	None
MOL000439	Yes	None	None
MOL000442	Yes	None	None
MOL001918	Yes	None	None
MOL002280	Yes	None	None
MOL003758	Yes	None	None
MOL004941	Yes	None	None
MOL006604	Yes	None	None
MOL006620	Yes	None	None
MOL007036	Yes	None	None
MOL007045	Yes	None	None
MOL007048	Yes	None	None
MOL007050	Yes	None	None
MOL007059	Yes	None	None
MOL007070	Yes	None	None
MOL007100	Yes	None	None
MOL007119	Yes	None	None
MOL007120	Yes	None	None
MOL007127	Yes	None	None
MOL007130	Yes	None	None
MOL007132	Yes	None	None
MOL007150	Yes	None	None
MOL007155	Yes	None	None
MOL012686	Yes	None	None
MOL012753	Yes	None	None
MOL012760	Yes	None	None
MOL012800	Yes	None	None
MOL007152	Yes	None	None
MOL007151	Yes	None	None
MOL012681	Yes	None	None
MOL007071	Yes	None	None
MOL007068	Yes	None	None
MOL007063	Yes	None	None
MOL000471	Yes	None	None
MOL004580	Yes	None	None
MOL000096	Yes	None	None

MOL000492	Yes	None	None
MOL000371	Yes	Yes	COC1=CC2=C(C=C1)[C@H]3[C@@H](CO2)C4=C(O3)C(=C(C=C4)OC)OC
MOL012714	Yes	Yes	COC1=CC2=C(C=C(O2)C3=CC(=CC(=C3)O)O)C(=C1)OC
MOL000378	Yes	Yes	COC1=CC2=C(C[C@@H](CO2)C3=C(C(=C(C=C3)OC)OC)O)C=C1
MOL000392	Yes	Yes	COC1=CC=C(C=C1)C2=COC3=C(C2=O)C=CC(=C3)O
MOL003856	Yes	Yes	COC1=CC(=CC(=C1)O)C2=CC3=CC(=C(C=C3O2)OC)O
MOL000239	Yes	Yes	COC1=CC(=C2C(=C1)OC(=C(C2=O)OC)C3=CC=C(C=C3)O)O
MOL000387	Yes	Yes	COC1=C2C(=C(C(=C1)C(=O)OC)C3=C4C(=C(C=C3C(=O)OC)OC)OCO4)OCO2
MOL001735	Yes	Yes	COC1=C(C2=C(C=C1O)OC(=CC2=O)C3=CC=C(C=C3)O)O
MOL000354	Yes	Yes	COC1=C(C=CC(=C1)C2=C(C(=O)C3=C(C=C(C=C3O2)O)O)O)O
MOL006613	Yes	Yes	COC1=C(C=C2C(=C1)C3COC4=C(C3O2)C=CC(=C4)O)O
MOL003860	Yes	Yes	COC1=C(C=C2C(=C1)C=C(O2)C3=CC(=CC(=C3)O)O)OC
MOL002514	Yes	Yes	COC1=C(C=C(C2=C1OC(=C(C2=O)O)C3=CC=C(C=C3)O)O)O
MOL005100	Yes	Yes	COC1=C(C=C(C=C1)C2CC(=O)C3=C(C=C(C=C3O2)O)O)O
MOL000417	Yes	Yes	COC1=C(C=C(C=C1)C2=COC3=C(C2=O)C=CC(=C3)O)O
MOL002235	Yes	Yes	COC1=C(C=C(C=C1)C2=C(C(=O)C3=C(C(=C(C=C3O2)OC)OC)O)O)O
MOL000033	Yes	Yes	CCC[C@@H](CC[C@@H](C)[C@H]1CC[C@@H]2[C@@]1(CC[C@H]3[C@H]2CC=C4[C@@]3(CC[C@@H](C4)O)C)C)C(C)C
MOL007108	Yes	Yes	CC1COC2=C1C(=O)C3=C(C2=O)C4=C(C=C3)C(CCC4)(C)C
MOL007079	Yes	Yes	CC1COC2=C1C(=O)C(=O)C3=C2C=CC4=C3CCCC4(C)C=O
MOL001601	Yes	Yes	CC1COC2=C1C(=O)C(=O)C3=C2C=CC4=C3CCC=C4C
MOL007093	Yes	Yes	CC1COC2=C(C1(C)CO)C(=O)C3=C(C2=O)C4=CC=CC(=C4C=C3)C
MOL007061	Yes	Yes	CC1=COC2=C1C(=O)C(=O)C3=C2C=CC4=C3CCCC4=C

MOL007069	Yes	Yes	CC1=COC2=C1C(=O)C(=O)C3=C2C=CC4=C3CCCC4(C)O
MOL007154	Yes	Yes	CC1=COC2=C1C(=O)C(=O)C3=C2C=CC4=C3CCCC4(C)C
MOL002281	Yes	Yes	CC1=CC2=CC3=CC(=CC(=C3C(=C2C(=O)O1)O)O)OC
MOL002259	Yes	Yes	CC1=CC2=C(C(=C1)OC3C(C(C(C(O3)COC4C(C(C(C(O4)CO)O)O)O)O)O)O)C(=O)C5=C(C2=O)C=C(C=C5O)OC
MOL007085	Yes	Yes	CC1=C2C=CC3=C4C2=C(C=C1)C(=O)C(=C4OC3(C)C)C(C)C
MOL007143	Yes	Yes	CC1=C2C=CC3=C4C2=C(C=C1)C(=O)C(=C4OC3(C)C)C(C)C
MOL007111	Yes	Yes	CC1=C2C=CC3=C(C2=CC=C1)C4=C(C(=CO4)C)C(=O)C3=O
MOL007156	Yes	Yes	CC1=C2C=CC3=C(C2=CC=C1)C(=O)C(=O)C(=C3O)C(C)CO
MOL007145	Yes	Yes	CC1=C(C(=O)C=C2C3=C(C=CC2=C1)C(CCC3)(C)C)O
MOL007121	Yes	Yes	CC1=C(C(=O)C=C2C(=C1)[C@@H]3C[C@@H]4[C@@]2(CCCC4(C)C)CO3)O
MOL012692	Yes	Yes	CC1(C2CCC3(C2C1C4=C(O3)C=C(C(=C4)C5CC(=O)C6=C(C=C(C=C6O5)O)O)O)C)C
MOL006596	Yes	Yes	CC1(C=CC2=C(O1)C=CC3=C2OC[C@@]4([C@H]3OC5=C4C=CC(=C5)O)O)C
MOL000456	Yes	Yes	CC1(C=CC2=C(O1)C=CC3=C2O[C@@H]4[C@H]3COC5=C4C=CC(=C5)O)C
MOL004912	Yes	Yes	CC1(C=CC2=C(O1)C=CC(=C2O)C3=COC4=C(C3=O)C=CC(=C4)O)C
MOL012755	Yes	Yes	CC1(C=CC2=C(C=CC(=C2O1)[C@@H]3CC(=O)C4=C(C=C(C=C4O3)O)O)O)C
MOL003858	Yes	Yes	CC1(C=CC2=C(C=C(C=C2O1)C3=CC4=C(O3)C=C(C=C4)O)O)C
MOL012735	Yes	Yes	CC1(C(CC2=C(O1)C=C3C(=C2)C=C(O3)C4=CC(=CC(=C4)O[C@H]5[C@@H]([C@H]([C@@H](CO5)O)O)O)O)O)C
MOL002297	Yes	Yes	CC[C@H](CC[C@@H](C)[C@H]1CC[C@@H]2[C@@]1(CC[C@H]3[C@H]2CC=C4[C@@]3(CC[C@@H](C4)O[C@H]5[C@@H]([C@H]([C@@H]([C@H](O5)CO)O)O)O)C)C)C(C)C
MOL000358	Yes	Yes	CC[C@H](CC[C@@H](C)[C@H]1CC[C@@H]2[C@@]1(CC[C@H]3[C@H]2CC=C4[C@@]3(CC[C@@H](C4)O)C)C)C(C)C

MOL000359	Yes	Yes	CC[C@H](CC[C@@H](C)[C@H]1CC[C@@H]2[C@@]1(CC[C@H]3[C@H]2CC=C4[C@@]3(CC[C@@H](C4)O)C)C)C(C)C
MOL000449	Yes	Yes	CC[C@H](/C=C/[C@@H](C)[C@H]1CC[C@@H]2[C@@]1(CC[C@H]3[C@H]2CC=C4[C@@]3(CC[C@@H](C4)O)C)C)C(C)C
MOL001771	Yes	Yes	CC[C@@H](CC[C@@H](C)[C@H]1CC[C@@H]2[C@@]1(CC[C@H]3[C@H]2CC=C4[C@@]3(CC[C@@H](C4)O)C)C)C(C)C
MOL007122	Yes	Yes	CC(C)C1=CC2=C(C3=C(C=C2)C(CCC3)(C)C)C(=O)C1=O
MOL007049	Yes	Yes	CC(C)C1=CC2=C(C3=C(C=C2)C(=C)CCC3)C(=O)C1=O
MOL007064	Yes	Yes	CC(C)C1=C[C@]23CCC4C(=CCCC4(C)C)[C@H]2OC(=O)[C@@]3(C1=O)O
MOL007098	Yes	Yes	CC(C)C1=C(C2=C(C3=C(C=C2)C(CCC3)(C)C)C(=O)C1=O)O
MOL007124	Yes	Yes	CC(C)C1=C(C2=C(C3=C(C=C2)C(CCC3)(C)C)C(=O)C1=O)O
MOL007107	Yes	Yes	CC(C)C1=C(C=C2C(=C1)CC[C@@H]3[C@@]2(CCCC3(C)C)C)O
MOL002222	Yes	Yes	CC(C)C1=C(C=C2C(=C1)C(=O)C[C@@H]3[C@@]2(CCCC3(C)C)C)O
MOL003347	Yes	Yes	CC(C)C(=O)[C@]12C(=O)C(=C([C@](C1=O)(C[C@@H]([C@@]2(C)CCC=C(C)C)CC=C(C)C)CC=C(C)C)O)CC=C(C)C
MOL003542	Yes	Yes	CC(C)/C=C/C1=C2C(=C(C=C1O)O)C(=O)C(=C(O2)C3=CC=C(C=C3)O)O
MOL012719	Yes	Yes	CC(C)(C1CC2=C(O1)C=C3C(=C2)C=C(O3)C4=CC(=CC(=C4)O)O)O
MOL001942	Yes	Yes	CC(=CCOC1=C2C=CC(=O)OC2=CC3=C1C=CO3)C
MOL006626	Yes	Yes	CC(=CCC1=C2C(=C(C=C1O)O)C(=O)C[C@H](O2)C3=C(C=C(C=C3)O)O)C
MOL003673	Yes	Yes	CC(=CCC1=C(C2=C(C=C1O)OC=C(C2=O)C3=CC=C(C=C3)O)O)C
MOL003857	Yes	Yes	CC(=CCC1=C(C=C(C=C1O)C2=CC3=C(O2)C=C(C=C3)O)O)C
MOL000275	Yes	Yes	CC(=CCC[C@H]([C@H]1CC[C@@]2([C@@]1(CCC3=C2CC[C@@H]4[C@@]3(CC[C@@H](C4(C)C)O)C)C)C)C(=O)O)C
MOL000273	Yes	Yes	CC(=CCC[C@H]([C@H]1[C@@H](C[C@@]2([C@@]1(CC=C3C2=CC[C@@H]4[C@@]3(CC[C@@H](C4(C)C)O)C)C)C)O)C(=O)O)C

MOL012689	Yes	Yes	CC(=CC1C2=C(C3=C(O1)C=C(C=C3)O)OC4=C(C2=O)C(=CC5=C4C=CC(O5)(C)C)O)C
MOL006623	Yes	Yes	CC(=C)C(CCC(C)(C)O)CC1=C2C(=C(C=C1O)O)C(=O)C[C@H](O2)C3=CC=CC=C3O
MOL000211	Yes	Yes	CC(=C)[C@@H]1CC[C@]2([C@H]1[C@H]3CC[C@@H]4[C@]5(CC[C@@H](C([C@@H]5CC[C@]4([C@@]3(CC2)C)C)(C)C)O)C)C(=O)O
MOL003627	Yes	Yes	C1C[C@H]2CN3[C@H](CC=CC3=O)[C@@H]4[C@H]2N(C1)CCC4
MOL003680	Yes	Yes	C1C[C@@H]2[C@H]3CCCN4[C@H]3[C@H](CCC4)CN2C(=O)C1
MOL005944	Yes	Yes	C1C[C@@H]2[C@H]3CCCN4[C@H]3[C@@H](CCC4)CN2C(=O)C1
MOL007141	Yes	Yes	C1C(C(=O)C2=CC3=C(C(=C(C=C3)O)O)OC4=C(C=CC1=C24)O)C(=O)O
MOL002268	Yes	Yes	C1=CC2=C(C(=C1)O)C(=O)C3=C(C2=O)C=C(C=C3O)C(=O)O
MOL002714	Yes	Yes	C1=CC=C(C=C1)C2=CC(=O)C3=C(O2)C=C(C(=C3O)O)O
MOL002776	Yes	Yes	C1=CC=C(C=C1)C2=CC(=O)C3=C(C(=C(C=C3O2)O[C@H]4[C@@H]([C@H]([C@@H]([C@H](O4)C(=O)O)O)O)O)O)O
MOL000422	Yes	Yes	C1=CC(=CC=C1C2=C(C(=O)C3=C(C=C(C=C3O2)O)O)O)O
MOL001004	Yes	Yes	C1=CC(=CC=C1C2=[O+]C3=CC(=CC(=C3C=C2O)O)O)O
MOL006630	Yes	Yes	C1=CC(=C(C=C1O)O)C2=CC(=O)C3=C(C=C(C=C3O2)O)O
MOL007142	Yes	Yes	C1=CC(=C(C=C1CC(C(=O)O)OC(=O)/C=C/C2=CC3=C(C=C2)O[C@@H]([C@@H](O3)C(=O)O)C4=CC(=C(C=C4)O)O)O)O
MOL004004	Yes	Yes	C1=CC(=C(C=C1C2=CC(=O)C3=C(O2)C=C(C(=C3O)O)O)O)O
MOL000006	Yes	Yes	C1=CC(=C(C=C1C2=CC(=O)C3=C(C=C(C=C3O2)O)O)O)O
MOL000098	Yes	Yes	C1=CC(=C(C=C1C2=C(C(=O)C3=C(C=C(C=C3O2)O)O)O)O)O
MOL000569	Yes	Yes	C1=C(C=C(C(=C1O)[O-])O)C(=O)OC2=CC(=CC(=C2O)O)C(=O)O
MOL006650	Yes	Yes	C1[C@@H]2[C@H](C3=C(O1)C=C(C=C3)O[C@H]4[C@@H]([C@H]([C@@H]([C@H](O4)COC(=O)CC(=O)O)O)O)O)OC5=CC6=C(C=C25)OCO6

MOL001484	Yes	Yes	C1[C@@H]2[C@H](C3=C(O1)C=C(C=C3)O)OC4=CC5=C(C=C24)OCO5
MOL003648	Yes	Yes	C1[C@@H]2[C@H](C3=C(O1)C=C(C=C3)O)OC4=CC5=C(C=C24)OCO5
MOL001040	Yes	Yes	C1[C@@H](OC2=CC(=CC(=C2C1=O)O)O)C3=CC=C(C=C3)O
MOL001474	Yes	Yes	C[N+]1=C2C(=C3C=CC4=C(C3=C1)OCO4)C=CC5=CC6=C(C=C52)OCO6
MOL007088	Yes	Yes	C[C@H]1COC2=C1C(=O)C(=O)C3=C2C=CC4=C3CCCC4(C)C
MOL007101	Yes	Yes	C[C@H]1COC2=C1C(=O)C(=O)C3=C2C=CC4=C(C=CC=C43)C
MOL007081	Yes	Yes	C[C@H]1COC2=C1C(=O)[C@@](C3=C2C=CC4=C3CCCC4(C)C)(CC(=O)C)O
MOL007082	Yes	Yes	C[C@H]1COC2=C1C(=O)[C@@](C3=C2C=CC4=C(C=CC=C43)C)(CC(=O)C)O
MOL007105	Yes	Yes	C[C@H]1C[C@@]2(C3=C(C4=CC=CC(=C4C=C3)C)C(=O)O2)OC1
MOL005043	Yes	Yes	C[C@H](CC[C@@H](C)C(C)C)[C@H]1CC[C@@H]2[C@@]1(CC[C@H]3[C@H]2CC=C4[C@@]3(CC[C@@H](C4)O)C)C
MOL000282	Yes	Yes	C[C@H](/C=C/[C@H](C)C(C)C)[C@H]1CC[C@@H]2[C@@]1(CC[C@H]3C2=CC[C@@H]4[C@@]3(CC[C@@H](C4)O)C)C
MOL000279	Yes	Yes	C[C@H](/C=C/[C@H](C)C(C)C)[C@H]1CC[C@@H]2[C@@]1(CC[C@H]3C2=C[C@H]([C@@]4([C@@]3(CC[C@@H](C4)O)C)O)O)C
MOL000283	Yes	Yes	C[C@H](/C=C/[C@H](C)C(C)C)[C@H]1CC[C@@H]2[C@@]1(CC[C@H]3[C@]24C=C[C@@]5([C@@]3(CC[C@@H](C5)O)C)OO4)C
MOL007115	Yes	Yes	C[C@]12CCCC([C@@H]1CCC(=C)[C@@H]2CC[C@](C)(C=C)O)(C)C
MOL007077	Yes	Yes	C[C@]12CCCC([C@@H]1CC[C@@]([C@@H]2CC[C@](C)(C=C)O)(C)O)(C)C
MOL001919	Yes	Yes	C[C@]12CC[C@@H](C([C@@H]1CC[C@@]3([C@@H]2C=CC4=C(C(=O)C(=O)[C@]43C)O)C)(C)C)O
MOL000296	Yes	Yes	C[C@]12CC[C@@H]([C@@]([C@@H]1CC[C@@]3([C@@H]2CC=C4[C@]3(CC[C@@]5([C@H]4CC(CC5)(C)C)C(=O)O)C)C)(C)CO)O
MOL001924	Yes	Yes	C[C@]12C[C@@]3([C@@H]4C[C@]1([C@@]4([C@H](O2)O3)COC(=O)C5=CC=CC=C5)O[C@H]6[C@@H]([C@H]([C@@H]([C@H](O6)CO)O)O)O)O

MOL007125	Yes	Yes	C[C@@H](CO)C1=C(C2=C(C3=C(C=C2)C(CCC3)(C)C)C(=O)C1=O)O
MOL002288	Yes	Yes	According to 2D structure
MOL007058	Yes	Yes	According to 2D structure
MOL007094	Yes	Yes	According to 2D structure
MOL000020	None	NA	None
MOL000021	None	NA	None
MOL000276	None	NA	None
MOL000280	None	NA	None
MOL000285	None	NA	None
MOL000374	None	NA	None
MOL000438	None	NA	None
MOL000554	None	NA	None
MOL001910	None	NA	None
MOL001925	None	NA	None
MOL001928	None	NA	None
MOL001930	None	NA	None
MOL002260	None	NA	None
MOL002276	None	NA	None
MOL006562	None	NA	None
MOL006565	None	NA	None
MOL006568	None	NA	None
MOL006570	None	NA	None
MOL006572	None	NA	None
MOL006573	None	NA	None
MOL006583	None	NA	None
MOL006619	None	NA	None
MOL006649	None	NA	None
MOL007051	None	NA	None
MOL007140	None	NA	None
MOL012717	None	NA	None
MOL012743	None	NA	None
MOL012749	None	NA	None
MOL000028	None	Yes	C[C@@H]1CC[C@@]2(CC[C@@]3(C(=CC[C@H]4[C@]3(CC[C@@H]5[C@@]4(CC[C@@H](C5(C)C)O)C)C)[C@@H]2[C@H]1C)C)C
MOL000287	None	Yes	CC(C)C(=C)CC[C@H]([C@H]1CC[C@@]2([C@@]1(CCC3=C2CC[C@@H]4[C@@]3(CC[C@@H](C4(C)C)O)C)C)C)C(=O)O
MOL000289	None	Yes	According to 2D structure
MOL000290	None	Yes	CC(C)C(=C)CC[C@H]([C@H]1[C@@H](C[C@@]2([C@@]1(CC=C3C2=CC[C@H]([C@]3(C)CCC(=O)O)C(=C)C)C)C)O)C(=O)O
MOL000291	None	Yes	CC(=CCC[C@H]([C@H]1[C@@H](C[C@@]

			2([C@@]1(CC=C3C2=CC[C@H]([C@]3(C)CCC(=O)O)C(=C)C)C)C)O)C(=O)O)C
MOL000292	None	Yes	CC(C)C(=C)CC[C@H]([C@H]1CC[C@@]2([C@@]1(CC=C3C2=CC[C@H]([C@]3(C)CCC(=O)O)C(=C)C)C)C)C(=O)O
MOL000300	None	Yes	CC(C)C(=C)CC[C@H]([C@H]1CC[C@@]2([C@@]1(CC=C3C2=CC[C@@H]4[C@@]3(CC[C@@H](C4(C)C)O)C)C)C)C(=O)O
MOL000398	None	Yes	C1C(C(=O)C2=CC=CC=C2O1)C3=CC=CC=C3
MOL001921	None	Yes	C[C@@]12[C@]3(C[C@@H]4[C@]3([C@H](O1)CC4=O)COC(=O)C5=CC=CC=C5)O[C@H]6[C@H](O2)[C@H]([C@@H]([C@H](O6)CO)O)O
MOL002251	None	Yes	CC1=C(C(CCC1)(C)C)/C=C/C(=C/C=C/C(=C/C=C/C=C(\C)/C=C/C=C(\C)/C2C=C3C(CCCC3(O2)C)(C)C)/C)/C
MOL002293	None	Yes	C1=CC2=C(C(=C1)O[C@H]3[C@@H]([C@H]([C@@H]([C@H](O3)CO)O)O)O)C(=O)C4=C([C@H]2[C@@H]5C6=C(C(=CC=C6)O[C@H]7[C@@H]([C@H]([C@@H]([C@H](O7)CO)O)O)O)C(=O)C8=C5C=C(C=C8O)C(=O)O)C=C(C=C4O)CO
MOL002303	None	Yes	CC1=CC2=C(C(=C1)O)C(=O)C3=C(C2C4C5=C(C(=CC=C5)O)C(=O)C6=C4C=C(C=C6O)CO)C=C(C=C3O)O
MOL003676	None	Yes	C1C[C@H]2CN3C(=O)C=CC=C3[C@@H]4[C@H]2N(C1)CCC4
MOL006561	None	Yes	C1C[C@H]2CN3[C@H](CC[C@H](C3=O)O)[C@@H]4[C@H]2N(C1)CCC4
MOL006563	None	Yes	C1C[C@@H]2[C@H]3C[C@@H](CN4[C@H]3[C@@H](CCC4)CN2C(=O)C1)O
MOL006564	None	Yes	C1C[C@@H]2[C@H]3CCCN4[C@@H]3[C@@H](CCC4)CN2C(=O)C1
MOL006566	None	Yes	C1C[C@H]2CN3[C@H](C=CCC3=O)[C@@H]4[C@H]2N(C1)CCC4
MOL006569	None	Yes	C1C[C@H]2CN3[C@H](CC[C@@H](C3=O)O)[C@@H]4[C@H]2N(C1)CCC4
MOL006571	None	Yes	C1CCN2C[C@H]3C[C@@H]([C@H]2C1)CN4C3=CC=CC4=O
MOL006582	None	Yes	According to 2D structure
MOL006622	None	Yes	COC1=CC=C(C=C1)C2=COC3=C(C2=O)C=CC(=C3)O[C@H]4C([C@H]([C@@H](C(O4)CO[C@H]5C([C@H]([C@@H](CO5)O)O)O)O)O)

			O)O
MOL006627	None	Yes	C1C[C@H]2CN3[C@H](C=CCC3=O)[C@@H]4[C@H]2N(C1)CCC4
MOL006628	None	Yes	C1CCN2C[C@@H]3C[C@H]([C@@H]2C1)CN4[C@@H]3CCCC4=O
MOL006652	None	Yes	C1[C@@H]2[C@H](C3=C(O1)C=C(C=C3)O[C@H]4[C@@H]([C@H]([C@@H]([C@H](O4)CO)O)O)O)OC5=CC6=C(C=C25)OCO6
MOL006824	None	Yes	C[C@@H]1CC[C@@]2(CC[C@@]3(C(=CC[C@H]4[C@]3(CC[C@@H]5[C@@]4(CC[C@@H](C5(C)C)O)C)C)[C@@H]2[C@H]1C)C)C
MOL007118	None	Yes	According to 2D structure
MOL007123	None	Yes	CC(C)C1=CC2=C(C3=C(C=C2)C(CCC3)(C)C)C(=O)C1=O
MOL007149	None	Yes	CC(C)C1=C(C=C2C(=C1)C(=O)C[C@@H]3[C@@]2(CCCC3(C)C)C)O
MOL007783	None	Yes	C1=CC=C(C=C1)C(=O)OCC2=C[C@H]([C@H]3[C@@H]2[C@@H](OC=C3)O[C@H]4[C@@H]([C@H]([C@@H]([C@H](O4)CO)O)O)O)O[C@H]5[C@@H]([C@H]([C@@H]([C@H](O5)CO)O)O)O
MOL007796	None	Yes	CCCCCCCCCCCCCCCC(=O)O[C@H]1CC[C@@]2([C@H]3CC[C@]4([C@H]([C@@H]3CC=C2C1)CC[C@@H]4[C@H](C)/C=C/[C@@H](CC)C(C)C)C)C
MOL007799	None	Yes	According to 2D structure
MOL009653	None	Yes	C[C@H]1[C@@H]2CC[C@H]3[C@@]4(CC[C@@H]([C@]4(CC[C@@]35[C@@]2(C5)CC[C@@H]1O)C)[C@H](C)CCC(=C)C(C)C)C
MOL012726	None	Yes	CC1=CC2C3C(C1)C4=C(C=C(C=C4)O)OC3(OC5=CC(=CC(=C25)O)C6=CC7=C(O6)C=C(C=C7)O)C8=C(C=C(C=C8)O)O
