# Supplementary material for: Niaoduqing alleviates podocyte injury in high glucose model via regulating multiple targets and AGE/RAGE pathway: Network pharmacology and experimental validation
Source: Front Pharmacol. 2023 Feb 27;14:1047184. doi: 10.3389/fphar.2023.1047184 (PMC10009170; doi:10.3389/fphar.2023.1047184)
Supplement: Supplementary file 8 [file Table9.pdf]

Table S9 The topological parameters of each nodes in compound-ingredients-therapeutic targets network

	Node	Degree Value	Betweenness Centrality	Closeness Centrality
1	PTGS2	117	18499.727	0.7589744
2	DPP4	49	3717.7412	0.43148687
3	NOS3	16	235.69447	0.3618582
4	MOL000098	11	1768.4996	0.5
5	VEGFA	8	550.50745	0.34660423
6	ICAM1	8	374.1687	0.34498835
7	HMOX1	7	327.02652	0.3498818
8	AGTR1	6	472.26212	0.31827956
9	REN	6	37.0461	0.32527474
10	FLT1	5	426.60004	0.3169165
11	MOL000006	5	370.97797	0.48051947
12	MOL000422	5	250.27715	0.47435898
13	F3	4	90.97898	0.32671082
14	MOL000378	4	222.37025	0.46835443
15	MOL007093	4	285.96643	0.4654088
16	MOL000354	3	58.24418	0.4625
17	MOL003758	3	58.24418	0.4625
18	MOL004912	3	58.24418	0.4625
19	MOL007094	3	119.20012	0.4654088
20	MOL002714	3	172.14651	0.46835443
21	MOL002514	3	58.24418	0.4625
22	MOL002235	3	58.24418	0.4625
23	MOL001484	3	192.98854	0.44311377
24	MOL003648	3	192.98854	0.44311377
25	MOL007105	3	304.51804	0.44578314
26	MOL000392	3	58.24418	0.4625
27	MOL006596	3	400.51013	0.44578314
28	MOL000289	3	481.2481	0.44578314
29	MOL007079	3	129.83095	0.4654088
30	MOL007069	3	119.20012	0.4654088
31	TGFB1	2	7.512769	0.3356009
32	MOL007068	2	39.856007	0.45962733
33	MOL007071	2	39.856007	0.45962733
34	ACE	2	34.73463	0.31157896
35	MOL007130	2	11.915721	0.43529412
36	MOL007132	2	39.856007	0.45962733
37	MOL012800	2	39.856007	0.45962733
38	MOL006604	2	11.915721	0.43529412
39	MOL006582	2	15.664773	0.30327868
40	MOL000456	2	216.63812	0.44047618
41	MOL000398	2	18.958529	0.26334518

42	MOL007058	2	39.856007	0.45962733
43	MOL003857	2	8.548148	0.24584718
44	MOL007125	2	175.32387	0.44047618
45	MOL007088	2	68.79243	0.44047618
46	MOL001040	2	91.1366	0.44047618
47	MOL000049	2	39.856007	0.45962733
48	MOL007045	2	39.856007	0.45962733
49	MOL007059	2	39.856007	0.45962733
50	MOL007070	2	39.856007	0.45962733
51	MOL007127	2	39.856007	0.45962733
52	MOL007150	2	39.856007	0.45962733
53	MOL007151	2	39.856007	0.45962733
54	MOL007152	2	39.856007	0.45962733
55	MOL007155	2	39.856007	0.45962733
56	MOL001921	2	4.4823494	0.25964913
57	MOL003673	2	39.856007	0.45962733
58	MOL003542	2	39.856007	0.45962733
59	MOL001735	2	39.856007	0.45962733
60	MOL001004	2	11.915721	0.43529412
61	MOL000417	2	39.856007	0.45962733
62	MOL000239	2	39.856007	0.45962733
63	MOL007100	2	39.856007	0.45962733
64	MOL012681	2	39.856007	0.45962733
65	MOL003858	2	175.32387	0.44047618
66	MOL012755	2	11.915721	0.43529412
67	MOL006626	2	11.915721	0.43529412
68	MOL006613	2	39.856007	0.45962733
69	MOL003347	2	68.79243	0.44047618
70	MOL006628	2	15.664773	0.30327868
71	MOL006569	2	15.664773	0.30327868
72	MOL006563	2	15.664773	0.30327868
73	MOL006561	2	15.664773	0.30327868
74	MOL007154	2	39.856007	0.45962733
75	MOL007111	2	39.856007	0.45962733
76	MOL007108	2	11.915721	0.43529412
77	MOL007061	2	39.856007	0.45962733
78	MOL005944	2	21.182518	0.30705395
79	MOL000358	2	102.4153	0.43529412
80	THBD	1	0	0.3340858
81	SOD1	1	0	0.3340858
82	MOL000022	1	0	0.43274853
83	SPP1	1	0	0.3340858
84	MOL007048	1	0	0.43274853
85	MOL000442	1	0	0.43274853

86	MOL000072	1	0	0.43274853
87	MOL000379	1	0	0.43274853
88	CXCL10	1	0	0.3340858
89	MOL012686	1	0	0.43274853
90	MOL012753	1	0	0.43274853
91	MOL012760	1	0	0.43274853
92	MOL000096	1	0	0.43274853
93	MOL000492	1	0	0.43274853
94	MOL006620	1	0	0.43274853
95	MOL000387	1	0	0.43274853
96	MOL004941	1	0	0.43274853
97	MOL007142	1	0	0.43274853
98	MOL007141	1	0	0.43274853
99	MOL002303	1	0	0.43274853
100	MOL000471	1	0	0.43274853
101	MOL000569	1	0	0.43274853
102	MOL004580	1	0	0.43274853
103	MOL002268	1	0	0.43274853
104	MOL006652	1	0	0.43274853
105	MOL012714	1	0	0.43274853
106	MOL007156	1	0	0.43274853
107	MOL001924	1	0	0.2578397
108	MOL007098	1	0	0.43274853
109	MOL006630	1	0	0.43274853
110	MOL012689	1	0	0.43274853
111	MOL007124	1	0	0.43274853
112	MOL007122	1	0	0.43274853
113	MOL006623	1	0	0.43274853
114	MOL007036	1	0	0.43274853
115	MOL007107	1	0	0.43274853
116	MOL007120	1	0	0.43274853
117	MOL007143	1	0	0.43274853
118	MOL005100	1	0	0.43274853
119	MOL004004	1	0	0.43274853
120	MOL012719	1	0	0.24104235
121	MOL000371	1	0	0.43274853
122	MOL000380	1	0	0.43274853
123	MOL001601	1	0	0.43274853
124	MOL007085	1	0	0.43274853
125	MOL007101	1	0	0.43274853
126	MOL007119	1	0	0.43274853
127	MOL007145	1	0	0.43274853
128	MOL002281	1	0	0.43274853
129	MOL012692	1	0	0.30204082

130	MOL001474	1	0	0.43274853
131	MOL007799	1	0	0.43274853
132	MOL007796	1	0	0.43274853
133	MOL007149	1	0	0.43274853
134	MOL006564	1	0	0.30204082
135	MOL000287	1	0	0.43274853
136	MOL007082	1	0	0.43274853
137	MOL007081	1	0	0.43274853
138	MOL007077	1	0	0.43274853
139	MOL007064	1	0	0.43274853
140	MOL007049	1	0	0.43274853
141	MOL003680	1	0	0.30204082
142	MOL002222	1	0	0.43274853
143	MOL001942	1	0	0.43274853
144	MOL000449	1	0	0.43274853
145	MOL000296	1	0	0.43274853
146	MOL000279	1	0	0.25694445
147	MOL000275	1	0	0.43274853
148	MOL000273	1	0	0.24183007
149	MOL000211	1	0	0.43274853
