# Supplementary material for: Niaoduqing alleviates podocyte injury in high glucose model via regulating multiple targets and AGE/RAGE pathway: Network pharmacology and experimental validation
Source: Front Pharmacol. 2023 Feb 27;14:1047184. doi: 10.3389/fphar.2023.1047184 (PMC10009170; doi:10.3389/fphar.2023.1047184)
Supplement: Supplementary file 9 [file Table6.pdf]

Table S6 The targets list of proteinuria

	Genecards	OMIM	pharmgkb	Disgenet	Overlapping
1	CUBN	CUBN	HLA-DQB1	LMX1B	CUBN
2	CLCN5	CLCN5	UGT1A9	HAVCR1	CLCN5
3	NPHS1		HLA-DRB1	TGFB1	HLA-DRB1
4	NPHS2		CYP3A4	AGTR1	ACTN4
5	AMN			NPHS1	HPSE2
6	LRP2			MYH9	PLCE1
7	WT1			COL4A5	VPS33A
8	ALB			SPP1	PSTPIP1
9	INF2			APOE	MEFV
10	ACTN4			INF2	STOX1
11	ACE			TRPC6	IRAK1
12	COL4A3			HLA-DRB1	COL4A5
13	CD2AP			NCK2	MAX
14	FLT1			DPP4	LEP
15	OCRL			PPARA	PTPN22
16	APOL1			DNASE1	ITGA3
17	COL4A5			CFH	BAZ1B
18	PAX2			SCNN1A	LMNB2
19	COL4A4			ALOX5AP	ANLN
20	B2M			MPV17	APOE
21	CORIN			NPHS2	C3
22	TRPC6			PTGS2	COX8A
23	LMX1B			REN	WDR19
24	STOX1			SOD1	LAGE3
25	PLCE1			ALB	OFD1
26	COQ8B			VEGFA	AGT
27	KIRREL1			HLA-B	COX10
28	NAGLU			CYBB	OCRL
29	PEE1			CTSL	GLA
30	FN1			RHOA	NEU1
31	CFH			IFNG	NPHS1
32	REN			NCK1	OSGEP
33	MYO1E			MUC16	FN1
34	DGKE			PAK2	HP
35	PTPRO			PAK1	ATP7B
36	PEE2			FAS	CTSB
37	PEE3			IL1RN	AMMECR1
38	APOE			AGT	DLST
39	TBC1D8B			CLCN5	SH2B1
40	ARHGDIA			HLA-DQA1	NUP85
41	NUP107			POMC	SPP1

42	AXDND1	GUSB	PTGS2
43	CTNS	NR4A1	SDHD
44	EMP2	LEP	B2M
45	MAGI2	LAMB2	NUP133
46	CRB2	CTSB	CLIP2
47	LCAT	RAB38	NOTCH2
48	NUP133	CXCL10	ERCC8
49	NOS3	C6	ACE2
50	LAMB2	SNRPD1	FASTKD2
51	ANLN	LEPR	MDH2
52	NUP160	ICAM1	VEGFA
53	NUP93	SDC1	APOL1
54	GLA	NCKAP5	TGFB1
55	NUP85	NCF2	PAX2
56	DAAM2	SORCS1	SLC35A1
57	CST3	SERPINA1	KIF1B
58	NUP205	NPPB	CLCNKB
59	TTC21B	OPTN	HLA-DQA1
60	KIRREL2	CLU	MYO1E
61	NOS1AP	ACE	SNAP29
62	APOA1	SLC9A1	FH
63	KANK2	CD55	NR3C2
64	TTC21B-AS1	MGAT5	HMOX1
65	MFF-DT	VTN	FLT1
66	VEGFA	TMEM163	GATA3
67	CTNS-AS1	ACTN4	SLC12A3
68	GFND1	TACO1	ERCC4
69	SMARCAL1	SAA1	FOXC2
70	TRIM8	PLCE1	GDNF
71	AMBP	CFB	CTNS
72	ARHGAP24	PRTN3	ALOX5AP
73	C3	COQ6	SDHAF2
74	ITGA3	PRDX1	SOD1
75	SCARB2	PAX2	CTLA4
76	CLCNKB	TPRKB	MFF-DT
77	LOC107982234	TRNE	NPHS2
78	CFHR5	PMM2	LPIN2
79	HNF4A	ATP7B	HLA-DPB1
80	WDR4	PDSS2	SLC25A11
81	COQ2	NUP107	LRP2
82	EHHADH	PRODH	CYBB
83	CFI	WDR19	TRPC6
84	COQ6	NUP133	GUSB
85	AVIL	PTPRO	MPV17

86	CFHR1	TMEM127	SAA1
87	THBD	OSGEP	NUP107
88	HMOX1	SARS2	IL1RN
89	VPS33A	RET	MAFB
90	FGF23	TBC1D8B	TFPI
91	CD79A	ANLN	CXCL10
92	RET	SDHAF2	SLC7A7
93	CRP	RFC2	KANK2
94	GATM	B2M	COA8
95	AGT	AMMECR1	LAMB2
96	AGTR1	ITGA8	HNF1B
97	ADIPOQ	COX14	GTF2I
98	RPL36A-HNRNPH2	WDR73	NCK1
99	SLC34A1	LMNB2	DGKE
100	WDR72	OFD1	LYZ
101	ZAP70	COA8	ADM
102	HNF1B	APOL1	REN
103	TGFB1	SLITRK6	TPRKB
104	MEFV	SLC25A11	HPSE
105	TNF	DGKE	LCAT
106	STAT2	SGPL1	POMC
107	ADAMTS13	MAFB	ALB
108	TSFM	MAGI2	C1QA
109	IL6	LPIN2	LIMK1
110	SGPL1	GTF2IRD1	TP53RK
111	GAPVD1	SCARB2	TBC1D8B
112	ANKFY1	SNAP29	EHHADH
113	NUP37	SLC7A7	ACSL4
114	EDN1	PSTPIP1	WDR73
115	INS	BAZ1B	SCO1
116	MMACHC	LAGE3	SDHA
117	MT-CO1	AMN	DNASE1
118	CARD14	CUBN	ZAP70
119	VPS35L	STAT4	FAN1
120	CD46	SLC12A3	C1R
121	PGF	MFF-DT	SDHB
122	ITGA8	VPS33A	COX14
123	ENG	SDHD	SARS2
124	PDSS2	SDHC	TMEM127
125	SPP1	SDHB	CD55
126	MYH9	SDHA	DPP4
127	CFHR2	VHL	NPPB
128	RBP4	HNF1B	MUC16
129	GBA	THBD	SGPL1

130	FRAS1	COQ8B	FAS
131	SPRY2	NUP85	DNASE1L3
132	NPPA	TRIM25	MGAT5
133	APOB	ZAP70	MYH9
134	CD151	WT1	NCF2
135	PLCE1-AS1	CLIP2	STAT4
136	TP53	C3	CFH
137	SYNPO	C1R	PRDX1
138	SGSH	C1QA	RHOA
139	CFHR3	SCO1	ITGA8
140	YRDC	SMARCAL1	SERPINA1
141	CCL2	GDNF	COL4A3
142	WDR73	NUP160	RET
143	GSN	FLT1	THBD
144	NOC3L	KIF1B	LEPR
145	MAGI2-AS3	FOXC2	WT1
146	GATA3	FAN1	INF2
147	PSTPIP1	FASTKD2	GSN
148	APOA2	FH	HLA-DPA1
149	UMOD	FGA	KCNE5
150	SLC12A3	STOX1	ELN
151	TF	FN1	SNRPD1
152	HGSNAT	CD2AP	MMACHC
153	RFH1	KCNE5	CD2AP
154	LAGE3	TBL2	WDR4
155	ATP6V1B1	GBA	VTN
156	CFB	GATA3	AGTR1
157	PTX3	PTPN22	GBA
158	FAN1	MMACHC	CD46
159	SERPINC1	SH2B1	HAVCR1
160	VCAM1	KANK2	TBL2
161	MTHFR	SLC37A4	SLITRK6
162	GON7	G6PC	ACE
163	ADM	ACSL4	SLC37A4
164	CTLA4	ERCC6	SORCS1
165	HP	CLCNKB	COQ6
166	VWF	COX20	RFC2
167	NEU1	ERCC8	PPARA
168	WDR19	TP53RK	COX6B1
169	SAA1	TREX1	CFI
170	OSGEP	ADAMTS13	ACKR1
171	DMP1	WDR4	COL4A4
172	PHEX	CORIN	MAGI2
173	ROBO2	SLC35A1	NUP160

174	AGTR2	COL4A3	SDC1
175	DNASE2	COL4A4	FGA
176	TP53RK	COX6B1	IFNG
177	YARS1	ERCC4	HELLPAR
178	LYZ	ELN	CLU
179	MT-TL1	EHHADH	PRODH
180	MAF	DNASE1L3	PRTN3
181	CXCL8	DLST	ICAM1
182	MMADHC	CTNS	SCARB2
183	MTMR10	CTLA4	CORIN
184	IGAN1	COX10	CTSL
185	TPRKB	COX8A	HNF4A
186	IL1B	HELLPAR	NOS3
187	SELP	GLA	PET100
188	NDUFAF6	OCRL	ERCC6
189	ENTPD5	TRNF	PTPRO
190	TMCO1	PET100	SDHC
191	C12orf57	ND6	ADAMTS13
192	C1orf122	ND4	COQ8B
193	PHEX-AS1	ND1	INSR
194	PTCHD1-AS	COX3	AMN
195	LOC107548112	COX2	HLA-B
196	ERCC6	COX1	ARHGDIA
197	SERPINE1	ND5	SCNN1A
198	ERCC4	TRNH	CFB
199	SLC37A4	TRNK	LMX1B
200	ERCC8	TRNL1	GTF2IRD1
201	IFT172	NOTCH2	F3
202	LEP	NOS3	TACO1
203	CCND1	NEU1	PDSS2
204	IL10	MYO1E	APOA1
205	STAT4	TRNW	PMM2
206	COL4A6	TRNS2	TREX1
207	PRTN3	TRNS1	SMARCAL1
208	NOS2	TRNQ	VHL
209	HPSE	TRNN	C6
210	APRT	MEFV	ADIPOQ
211	VHL	MDH2	HLA-DQB1
212	NLRP3	CD46	UGT1A9
213	GC	GSN	CYP3A4
214	TLR4	INSR	
215	ICAM1	CFI	
216	SLC2A9	APOA1	
217	FGA	HNF4A	

218	FGFR3	HMOX1
219	SDHB	HLA-DPB1
220	SDHD	HLA-DPA1
221	GPX3	GTF2I
222	PON1	IRAK1
223	APOH	ITGA3
224	PPBP	LCAT
225	F3	MAX
226	TNXB	LYZ
227	TFRC	ARHGDIA
228	SOD1	LIMK1
229	SELL	LRP2
230	IL18	HPSE
231	SELE	F3
232	NIPBL	ACE2
233	KNG1	TFPI
234	SHH	ADIPOQ
235	AFP	HP
236	SLC17A5	NR3C2
237	CGB7	HPSE2
238	MMP9	ADM
239	F2	ACKR1
240	CTSL	
241	ATP7B	
242	MAX	
243	MT-CO2	
244	MT-CO3	
245	SMARCA2	
246	KCNE5	
247	LEPR	
248	SLC22A12	
249	ZFPM2	
250	SLC12A1	
251	ELN	
252	PLA2R1	
253	MT-ATP6	
254	APOA4	
255	ACSL4	
256	SLC35A1	
257	PMM2	
258	PLIN1	
259	MAFB	
260	IRAK1	
261	HIF1A	

262	LOC106627981
263	NF1
264	ACP5
265	LPIN2
266	AMMECR1
267	CD40LG
268	SLC9A3
269	F5
270	INPP5B
271	ERCC1
272	CBLIF
273	LPA
274	VLDLR
275	SDHA
276	KIF1B
277	OFD1
278	TMEM127
279	COA8
280	MT-TS1
281	MT-TK
282	MT-TE
283	MT-TN
284	PRODH
285	LMNB2
286	SNAP29
287	PUS3
288	UTS2
289	PKD1
290	GGT1
291	HMGB1
292	CFTR
293	LIG4
294	G6PC1
295	RAD21
296	NDUFAF2
297	PAPPA
298	IGFBP3
299	TCN1
300	MTR
301	LCN2
302	ZNF592
303	PLAU
304	PTPN22
305	ANGPTL4

306	HSD11B2
307	MDH2
308	NPHP3
309	MIR146A
310	PODXL
311	SLC34A3
312	SOX17
313	ITGAM
314	FGF8
315	PECAM1
316	DCN
317	PLAT
318	FGFR1
319	ANXA5
320	TKT
321	MMP2
322	DNMT3A
323	CD81
324	BDKRB2
325	GTF2I
326	ALG1
327	TGFB3
328	PAPPA2
329	H19
330	BRD4
331	SCGB1A1
332	KISS1
333	KCNJ1
334	BSND
335	HOGA1
336	ERVW-1
337	KDR
338	LMBRD1
339	EPAS1
340	FH
341	SLC7A7
342	STX1A
343	PIGA
344	SDHC
345	DNASE1L3
346	SURF1
347	SDHAF2
348	MT-ND4
349	MT-ND6

350 CFP
351 INSM2
352 KAT6B
353 AMMEC
354 MTHFD1
355 CLCN4
356 HLA-DRB1
357 ENPEP
358 IDO1
359 CRH
360 MIR25
361 MED13L
362 DHFR
363 TCN2
364 CLCN7
365 HDAC8
366 KMT2A
367 TAF6
368 MRXSMP
369 SHBG
370 MIR214
371 DAB2
372 LRP8
373 STRA6
374 CWF19L1
375 ANKRA2
376 OR4D1
377 ANKRD37
378 GRID2IP
379 IRX2-DT
380 GARIN5B
381 IL15
382 LOC106501713
383 ITGA1
384 SLC19A2
385 UMPS
386 CD63
387 PRDX1
388 PYGL
389 VPS37D
390 CLU
391 APOC2
392 NR3C2
393 ITGAL

394	CLCN3
395	APPL1
396	CLCNKA
397	PHETA1
398	DDOST
399	KARS1
400	VPS50
401	HBEGF
402	OLR1
403	MALAT1
404	NOTCH2
405	TREX1
406	SLC2A2
407	INHA
408	OXT
409	TAC3
410	MIR210
411	LGALS13
412	GAA
413	DDAH2
414	CGB3
415	HAVCR1
416	HLA-G
417	HOTAIR
418	MME
419	ERAP2
420	PIGF
421	GCM1
422	SLC19A1
423	SLC19A3
424	TPK1
425	MTRR
426	FTCD
427	SLC46A1
428	CD320
429	MPO
430	WDR35
431	TACR3
432	LNPEP
433	PLAC1
434	CLCN1
435	ADD1
436	PLAUR
437	GPT

438	IFNA1
439	MIR15A
440	IFT140
441	NSD1
442	CSH1
443	CSH2
444	LEPQTL1
445	AGER
446	ACVR2A
447	NOSTRIN
448	MIR125B1
449	FABP1
450	GNE
451	TUG1
452	LIMK1
453	DLST
454	NCF1
455	HLA-DPB1
456	RFC2
457	FOXC2
458	SARS2
459	SH2B1
460	SLC25A11
461	GTF2IRD1
462	MLXIPL
463	MTX2
464	BAZ1B
465	EIF4H
466	HLA-DPA1
467	TBL2
468	CLIP2
469	FKBP6
470	SLITRK6
471	BCL7B
472	UNC45A
473	DNAJC30
474	GTF2IRD2
475	MT-ND5
476	BUD23
477	MT-ND1
478	METTL27
479	TMEM270
480	HELLPAR
481	RNU7-1

482	MT-TF
483	MT-TH
484	MT-TQ
485	MT-TS2
486	MT-TW
487	CCN2
488	CABIN1
489	ADAM10
490	KRAS
491	KRT5
492	SEC61A1
493	EBF3
494	MIR106B
495	MIR93
496	MIR32
497	MIR181A2
498	MIR19A
499	VUR
500	HNFJ3
501	VUR4
502	VUR5
503	VUR6
504	VUR7
505	GUSB
506	LPL
507	LIPA
508	BMP7
509	NPY
510	BCL2
511	THBS1
512	COX4I1
513	ABCD4
514	COX10
515	COX6B1
516	FASTKD2
517	COA3
518	LOC100506321
519	CDC42
520	FST
521	ADAM12
522	EPHX1
523	HLA-C
524	SERPINB2
525	IGFBP1

526	INHBB
527	IL16
528	PSG1
529	C19orf33
530	CCN1
531	MIR126
532	MIR574
533	SPRY4-IT1
534	WFDC21P
535	YWHAEP7
536	CEACAMP8
537	FLT1P1
538	NR5A1
539	CHD7
540	AGRN
541	LTC4S
542	MIF
543	HNF1A
544	ACACB
545	JAK1
546	TNS2
547	IFNG
548	FAT1
549	APOC3
550	IFIH1
551	FOXP3
552	NID1
553	KIT
554	ANOS1
555	CDKN1C
556	LDHA
557	TLR5
558	TNFRSF1B
559	HGF
560	IL4
561	HMGCR
562	BMP6
563	ANGPTL3
564	EYA1
565	TJP1
566	ACTN1
567	LAMB1
568	ACE2
569	EGF

570	SPTAN1
571	ATRIP
572	GRK2
573	ATRIP-TREX1
574	HSPG2
575	TTR
576	MTMR3
577	IL11
578	NPPB
579	TNFRSF1A
580	SMPD1
581	KL
582	PRKCB
583	NPNT
584	IL1RN
585	EPO
586	TGIF1
587	IGF1
588	KANK1
589	IL13
590	DES
591	VDR
592	SERPINA3
593	CETP
594	SLC4A1
595	HCFC1
596	LBR
597	SCO1
598	ABCG5
599	NDUFV1
600	FAM20C
601	PRMT7
602	ATP6V0A4
603	BAAT
604	NODAL
605	FOXH1
606	COX8A
607	EVC2
608	TACO1
609	CEP120
610	DYNC2H1
611	DYNC2LI1
612	HYLS1
613	TRAF3IP1

614 CFHR4
615 IFT80
616 KIAA0753
617 DYNC2I2
618 PET100
619 DYNC2I1
620 PGBD3
621 FOXC2-AS1
622 MT-TT
623 NPHP3-AS1
624 APOA1-AS
625 MT-TV
626 MT-TI
627 MT-TL2
628 NPHP3-ACAD11
629 MT-TA
630 RCCD1-AS1
631 MT-TP
632 LOC105371046
633 TRIM59-IFT80
634 ERCC8-AS1
635 SHFM3
636 ATD
637 LOC107303340
638 LOC107372315
639 WG
640 HPE1
641 HPE6
642 HPE8
643 IGAN2
644 IL17A
645 HYOU1
646 EHD1
647 MIR21
648 C4A
649 NPHP1
650 CDK20
651 ITSN1
652 ITSN2
653 SOX9
654 SRY
655 CHI3L1
656 IFNB1
657 ADD2

658	KHDRBS3
659	APCS
660	LAMP1
661	CEP164
662	C5
663	LDLR
664	THSD7A
665	CFL1
666	ITGB2
667	ENPP1
668	IL2
669	TGFBI
670	SERPINA1
671	FREM2
672	UPK3A
673	IL2RA
674	CD4
675	CD80
676	LCN1
677	PIK3C2A
678	ALOX5
679	SOD3
680	PRL
681	GDNF
682	GREM1
683	PLD3
684	EVL
685	CTSD
686	CASP8
687	F9
688	LAMP2
689	CR1
690	HLA-B
691	C4B
692	PTGDS
693	TNFSF13B
694	ICOSLG
695	KLF4
696	ANKRD1
697	TMEM234
698	NOTCH1
699	EDNRB
700	TNFSF12
701	LAMA5

702 CASP1
703 CACNA1B
704 TLN1
705 SERPINF2
706 NR0B1
707 IL7R
708 KANK4
709 SALL1
710 SIX1
711 SIX2
712 ANGPT2
713 CMIP
714 CHIT1
715 FUCA1
716 ACTN2
717 IGF2R
718 TALDO1
719 CRYAA
720 IQGAP1
721 LMNA
722 CDK5
723 MAPK1
724 SERPINB7
725 GALNS
726 IDUA
727 SEMA3A
728 SDC2
729 GALC
730 AHSG
731 SPINK1
732 CD55
733 TLR2
734 ARSA
735 PSAP
736 BLOC1S1
737 H2AC18
738 CEP290
739 CAV1
740 SERPINF1
741 FABP3
742 CD8A
743 SIX5
744 COL4A1
745 MYCN

746 ADCYAP1
747 CD40
748 PXDN
749 ARSB
750 TFEB
751 DYNC1H1
752 CXCL10
753 CXCR4
754 VTI1B
755 PLD4
756 DGKK
757 PLD5
758 HORMAD2
759 MBOAT2
760 UGCG
761 MBL2
762 PRMT1
763 DAG1
764 FTH1
765 EPHX2
766 MIR155
767 CD38
768 COL4A2
769 MX1
770 ASAH1
771 GRHPR
772 HEXA
773 GNS
774 NPC2
775 PTK2
776 SIX3
777 C16orf72
778 TIMP1
779 PPP3CA
780 PTGS2
781 TMEM176A
782 TMEM176B
783 MIR186
784 DLC1
785 PAX8
786 SMAD9
787 LAMA2
788 ST6GALNAC1
789 ELANE

790	RETN
791	BRAF
792	ALOX5AP
793	MIR29A
794	DDX58
795	M6PR
796	MUS81
797	WNT9B
798	OGA
799	SNCA
800	NPC1
801	SMAD2
802	IL5
803	LIMA1
804	STAMBP
805	HLA-A
806	SAA4
807	BGLAP
808	NR1H4
809	F2R
810	MMP10
811	SREBF1
812	TRPV5
813	KCNAB1
814	MTOR
815	FGF2
816	GNPTAB
817	VTN
818	ADA
819	TCF21
820	GLB1
821	HEXB
822	HYAL1
823	PPT1
824	CTSA
825	MAN2B1
826	SERPIND1
827	SI
828	MGAM
829	TPP1
830	CLN5
831	FUCA2
832	TSLP
833	ARSH

834	NR3C1
835	IGHE
836	CCKAR
837	NOTCH4
838	GNA12
839	STAT1
840	HSP90AA1
841	AGPAT2
842	HLA-DQB1
843	BSCL2
844	MIR17
845	FASLG
846	ATP2B1
847	COL8A2
848	NEK8
849	COL8A1
850	YWHAE
851	SMARCA1
852	C1QBP
853	TBX18
854	ITGB1
855	UCHL1
856	PLA2G7
857	KMO
858	SIRT6
859	ATG5
860	PDCD4
861	MIR152
862	LOC117600004
863	YAP1
864	NAGA
865	IGKC
866	CASP3
867	ICAM3
868	FGFR2
869	BMP4
870	PLCG1
871	GFRA1
872	EMX2
873	WNT11
874	DSTYK
875	HOXB7
876	MMP3
877	MYD88

878	PDSS1
879	COQ9
880	MPZ
881	MTMR2
882	GDAP1
883	SBF2
884	SH3TC2
885	CP
886	ABCB1
887	APELA
888	DDR1
889	PLG
890	PHRF1
891	BID
892	VEGFD
893	CD14
894	HFE
895	NAMPT
896	MYC
897	IGF2
898	WNT4
899	GNRH1
900	RAG1
901	TTC8
902	IFT74
903	WDPCP
904	BBIP1
905	GSTP1
906	IL1R1
907	SMARCA4
908	HPRT1
909	WRN
910	CD34
911	LHX1
912	RAB8A
913	FCAR
914	IL3
915	KITLG
916	TAGLN
917	TRIO
918	IRF4
919	MUC1
920	LGALS3BP
921	TSG101

922	GP2
923	NARS1
924	TLR9
925	AXL
926	BNIP3L
927	HPX
928	CSF2
929	C1GALT1C1
930	LAP3
931	CTSB
932	CSF1
933	JUN
934	CCR5
935	BAX
936	PAX6
937	RARG
938	ITGA2
939	SLIT2
940	PAX1
941	SLIT3
942	SPRY1
943	GGA3
944	CCL3
945	CELF4
946	GALNT4
947	CNTLN
948	POC1B
949	SLF2
950	PDGFA
951	IFNA2
952	SDC1
953	COQ4
954	CD59
955	MAPK14
956	HDAC1
957	PAFAH1B1
958	RENBP
959	CYLD
960	CXCL12
961	IL1A
962	COMT
963	XDH
964	CD19
965	COL1A1

966	IDS
967	PARP1
968	RRM2B
969	COL1A2
970	CYCS
971	CYP27B1
972	MVK
973	POLG
974	CYP24A1
975	VEGFC
976	B4GALNT1
977	ETFA
978	IRF3
979	HYAL2
980	MBTPS1
981	MYO5B
982	ARSG
983	CCR6
984	CDKN3
985	CLN3
986	NARS2
987	AFF2
988	MCOLN1
989	SUMF1
990	CIDEC
991	RNASE3
992	BAZ1A
993	GNPTG
994	SPAM1
995	DYM
996	EXTL2
997	HLTF
998	ARSK
999	CLN6
1000	TIPIN
1001	IFT43
1002	NAGPA
1003	SLX4
1004	VPS16
1005	ZRANB3
1006	ASTN1
1007	CERT1
1008	TMEM164
1009	RADX

1010	SNORD96B
1011	CYP11B2
1012	MET
1013	APLN
1014	EDNRA
1015	CD36
1016	FCGR3B
1017	HRAS
1018	CDK2
1019	GJA1
1020	TNNI3
1021	POMC
1022	FREM1
1023	ZMPSTE24
1024	MYOM2
1025	MEG3
1026	RAC1
1027	NES
1028	CPB2
1029	GRIN2A
1030	CACNA1A
1031	ALOX15
1032	SLC16A3
1033	RAP1B
1034	DNAH5
1035	SH3GL3
1036	SOX11
1037	XK
1038	CLMP
1039	GAS7
1040	RSU1
1041	PLXDC2
1042	RSL24D1
1043	UACA
1044	CAMKMT
1045	MDM1
1046	PIDD1
1047	UNC13C
1048	SAMD3
1049	CCDC57
1050	EVX2
1051	SMTNL2
1052	FAM151B
1053	TMEM80

1054	LNPK
1055	TRARG1
1056	GATD1
1057	CYYR1-AS1
1058	SGO1-AS1
1059	LINC02384
1060	GATD1-DT
1061	LINC01591
1062	VENTXP7
1063	LINC01996
1064	MIR4493
1065	ENSG00000255142
1066	OR7E31P
1067	ENSG00000255158
1068	IL6RP1
1069	LINC02727
1070	RPL23AP56
1071	lnc-NUP107-26
1072	ENSG00000273824
1073	HSALNG0014593
1074	HSALNG0092160
1075	PHB1P17
1076	RF00017-1082
1077	RF00017-2033
1078	RF00017-549
1079	piR-40250-003
1080	ENSG00000287830
1081	HSALNG0076416
1082	HSALNG0082020
1083	RF00017-765
1084	lnc-EMP2-5
1085	piR-32214-258
1086	piR-32214-259
1087	piR-57133-216
1088	piR-57392-001
1089	HSALNG0114029
1090	piR-41525-054
1091	piR-43105-349
1092	PCNA
1093	F2RL1
1094	FAS
1095	SMPDL3B
1096	RASA1
1097	SDCCAG8

1098 FCGR2A
1099 AVP
1100 PTPRU
1101 FBP1
1102 ABCC2
1103 CYP2C9
1104 MB
1105 NEXMIF
1106 GSTM1
1107 CD44
1108 PNPLA3
1109 PPARG
1110 AKR1B1
1111 ENO1
1112 SAA2
1113 IGHG1
1114 RAB27A
1115 ALAD
1116 MASP2
1117 ABCB7
1118 UBC
1119 ATP7A
1120 LYN
1121 SGK1
1122 PTHLH
1123 GAS6
1124 TNFRSF11A
1125 RBP1
1126 ITGB3
1127 EP300
1128 LECT2
1129 PRKAG2
1130 KCNN4
1131 HDAC9
1132 PTPN12
1133 SPTLC2
1134 TNFRSF11B
1135 WAS
1136 XIAP
1137 CD79B
1138 FANCD2
1139 FGF10
1140 NCAM1
1141 RB1

1142	SPTLC1
1143	TNFSF11
1144	AGA
1145	ALDH3A2
1146	CD27
1147	GPT2
1148	IFNAR1
1149	SLC22A6
1150	SRPK1
1151	USP1
1152	CDKN2C
1153	DKK1
1154	NKX2-5
1155	TCF3
1156	ADAM19
1157	CD209
1158	GYPC
1159	NLRP12
1160	PROK2
1161	SFRP4
1162	SORT1
1163	SRD5A1
1164	TLR6
1165	A4GALT
1166	FANCM
1167	GDF11
1168	GRIP1
1169	HS2ST1
1170	HS6ST1
1171	IGBP1
1172	IL1RL2
1173	IL9
1174	KMT2D
1175	LHX4
1176	PROKR2
1177	SAMHD1
1178	DEGS1
1179	FANCI
1180	KLRK1
1181	MEST
1182	REV1
1183	CD58
1184	HPGDS
1185	IFI30

1186	KDSR
1187	ORMDL3
1188	ACER3
1189	BST2
1190	CERS3
1191	CLEC4G
1192	CSF3
1193	DSPP
1194	ETV5
1195	GTPBP1
1196	HEY2
1197	SAFB
1198	SRSF6
1199	TLX1
1200	UBE2D4
1201	WDR48
1202	BICC1
1203	FGF20
1204	FUT4
1205	NSMF
1206	PKHD1
1207	FCHO2
1208	MEPE
1209	PSTPIP2
1210	SLC13A1
1211	AMER1
1212	DCLRE1A
1213	FOXD1
1214	SPATA5L1
1215	SSPN
1216	TBC1D23
1217	TMTC3
1218	DCLRE1B
1219	ASNSD1
1220	GREB1L
1221	PCNP
1222	SPTSSA
1223	TMBIM4
1224	YIPF2
1225	ZCCHC14
1226	CAVIN1
1227	CCDC82
1228	LARGE1
1229	RBM33

1230 H3C1
1231 FREM3
1232 SLX1B
1233 LARGE2
1234 SLX1A
1235 FAAP100
1236 FAAP24
1237 C16orf95
1238 FAAP20
1239 MIR98
1240 NRIR
1241 AZGP1
1242 BLK
1243 THY1
1244 COG2
1245 PIK3R1
1246 MMP7
1247 RAB7A
1248 PTH
1249 ATF3
1250 CANX
1251 C3AR1
1252 TLR10
1253 IL27
1254 TRA
1255 SLC2A1
1256 S100A9
1257 VKORC1
1258 CXCL16
1259 PLA2G1B
1260 CCL5
1261 CDH5
1262 AQP1
1263 ALPI
1264 DNAJB9
1265 ASS1
1266 PYGM
1267 B4GALT1
1268 GBE1
1269 MPI
1270 SERPINA7
1271 IGHM
1272 H2AC20
1273 CAT

1274	NRP1
1275	TNFSF13
1276	ERBB2
1277	CDKN2A
1278	INPPL1
1279	GRN
1280	HTR2A
1281	GRB2
1282	NPR3
1283	CTSG
1284	FURIN
1285	SHC1
1286	GDF15
1287	LOC110806262
1288	ERCC2
1289	XPA
1290	PRKCD
1291	MAP3K1
1292	CASP10
1293	GATA4
1294	IL2RG
1295	WWOX
1296	DCLRE1C
1297	TRIM32
1298	ARL6
1299	BBS2
1300	BBS4
1301	RAG2
1302	BBS10
1303	CHST14
1304	SLC35A2
1305	VAMP7
1306	MKS1
1307	BBS5
1308	BBS9
1309	LZTFL1
1310	MKKS
1311	BBS1
1312	BBS7
1313	DHX37
1314	IFT27
1315	MARS1
1316	SCAPER
1317	BBS12

1318	ZNFX1
1319	DMRT3
1320	CEP19
1321	CFAP418
1322	CYBC1
1323	RMRP
1324	LRPAP1
1325	ENAH
1326	CFD
1327	KRT7
1328	KIRREL3
1329	NCK1
1330	PIK3CD
1331	DGUOK
1332	CEP83
1333	NTRK2
1334	IDH1
1335	IDH2
1336	GNAS
1337	IL6ST
1338	CD28
1339	PDGFD
1340	UPK2
1341	H2AC16
1342	H2AC17
1343	CNDP1
1344	PLTP
1345	S100A8
1346	PDPN
1347	ATP6AP2
1348	GSTT1
1349	LIPE
1350	SIRT1
1351	ADAMTSL1
1352	CDKN1A
1353	CCR1
1354	NPHP4
1355	PDE5A
1356	ZFP36
1357	AQP2
1358	CBS
1359	FBN1
1360	ISG15
1361	IAPP

1362	ITGAV
1363	IL12A
1364	TIMP2
1365	TNFRSF12A
1366	ZHX2
1367	TNFRSF10B
1368	CALB1
1369	PMP22
1370	EMCN
1371	STAT3
1372	FGR
1373	IL4R
1374	ITGA4
1375	BCL2L1
1376	GZMB
1377	TNFRSF17
1378	COQ7
1379	KMT2C
1380	LTA
1381	COQ8A
1382	ERCC3
1383	SLC4A4
1384	AGL
1385	BCS1L
1386	POLR2A
1387	SMC1A
1388	SMC3
1389	DDB1
1390	TIMM8A
1391	POLR2L
1392	HMGN1
1393	XAB2
1394	CCL18
1395	UVSSA
1396	ELOA
1397	CALR
1398	GCK
1399	NOTCH3
1400	MECP2
1401	NFKB1
1402	WTAP
1403	CDH2
1404	VCP
1405	SMO

1406	C1R
1407	C2
1408	TUBB1
1409	COMP
1410	ENO2
1411	FAH
1412	FBLN5
1413	GP1BA
1414	GSS
1415	MYH10
1416	SERPING1
1417	UBA1
1418	YWHAZ
1419	ARF1
1420	EFEMP2
1421	HPD
1422	PEPD
1423	UBE2D3
1424	PCBD1
1425	RAC3
1426	STK24
1427	ARHGAP1
1428	LYVE1
1429	TUBB4B
1430	BST1
1431	ESD
1432	RPS27A
1433	PDLIM1
1434	SLC3A2
1435	FAT4
1436	PRG2
1437	SERPINB1
1438	CEACAM8
1439	CAND1
1440	KRT33B
1441	PCBP3
1442	PLBD2
1443	MVB12A
1444	H4C1
1445	H2AC4
1446	PGR
1447	PROM1
1448	HMCN1
1449	HAMP

1450	C1GALT1
1451	ZHX1
1452	ZHX3
1453	SCNN1G
1454	CXCR3
1455	MAPK10
1456	CD247
1457	MASP1
1458	CD163
1459	CGA
1460	CLDN16
1461	LRRC7
1462	MIR27A
1463	IGF1R
1464	FLT3
1465	HDAC4
1466	IL6R
1467	CASP9
1468	MCL1
1469	DDIT3
1470	TNFRSF13B
1471	BCL2L11
1472	CD47
1473	SSB
1474	MIR140
1475	MIR125A
1476	PTEN
1477	DPP4
1478	PRSS8
1479	APOD
1480	SIRPA
1481	IGHA1
1482	MIR378A
1483	TEK
1484	ABCA1
1485	CNDP2
1486	LRP2BP
1487	MIR192
1488	MIR196A1
1489	ANPEP
1490	G6PD
1491	CX3CL1
1492	JAK2
1493	FOS

1494	SRC
1495	NOD2
1496	NTRK1
1497	SLC6A2
1498	FCGR2B
1499	ADRB3
1500	UCP1
1501	SEMA6A
1502	IL21
1503	PF4
1504	ESM1
1505	MUC16
1506	MAL
1507	VIM
1508	ALPL
1509	ACTR2
1510	DNM3
1511	MPDU1
1512	NAT2
1513	SEMA5A
1514	ST3GAL4
1515	CARS1
1516	FRMD3
1517	SOX15
1518	ACOXL
1519	TNFSF12-TNFSF13
1520	HCG9
1521	MIR193A
1522	BTK
1523	DBH
1524	BRCA1
1525	DRD2
1526	GYS1
1527	LRRK2
1528	TOP1
1529	USP7
1530	ABCC8
1531	GLI3
1532	TLR8
1533	MAN1B1
1534	OGG1
1535	RPA1
1536	SETD2
1537	UQCRC2

1538	XPC
1539	AGXT
1540	DDB2
1541	ERCC5
1542	PHKA2
1543	GYG1
1544	NDUFA1
1545	PARP2
1546	RAB5A
1547	VIP
1548	YARS2
1549	APEX1
1550	CALCA
1551	CUL5
1552	DIAPH2
1553	GH1
1554	GP1BB
1555	POLR1A
1556	POLR1C
1557	UPB1
1558	ALG2
1559	ALG8
1560	CCBE1
1561	COX15
1562	CUL4A
1563	CUL4B
1564	MNX1
1565	NDUFB8
1566	RBX1
1567	SLC35A3
1568	SMARCA5
1569	TBX1
1570	VPS33B
1571	GBA2
1572	IFT88
1573	INPP5E
1574	RAB6A
1575	SST
1576	PLEK
1577	RAB35
1578	SLC35C1
1579	IFT122
1580	S100A12
1581	TRMU

1582	CCP110
1583	COG1
1584	DNAH8
1585	RAB3IP
1586	TCEA1
1587	OSGEPL1
1588	TCEA3
1589	TXNRD3
1590	WDR81
1591	COX14
1592	KIF24
1593	CEP89
1594	CEP97
1595	KIF19
1596	SEC22B
1597	ARMCX2
1598	POC5
1599	PRDM15
1600	FBF1
1601	KLHDC7A
1602	MT-CYB
1603	PAGR1
1604	TWNK
1605	LRRC37A2
1606	SPIN3
1607	SLC66A1
1608	PIFO
1609	RAB40AL
1610	CEP20
1611	MT-TR
1612	HDAC2
1613	SMAD3
1614	ANXA2
1615	FYN
1616	SCARB1
1617	FCGR3A
1618	HBB
1619	NDST1
1620	FABP4
1621	PRDX5
1622	PNPLA8
1623	RAB11B
1624	CD69
1625	NLRC5

1626	CASR
1627	CREB1
1628	NGF
1629	MAPK3
1630	SLC3A1
1631	HTRA1
1632	CCR4
1633	GPNMB
1634	C5AR1
1635	FCN2
1636	CHKA
1637	ASAH2
1638	TREM1
1639	GRP
1640	IL15RA
1641	PSMC6
1642	GAST
1643	TRAPPC10
1644	VSIG4
1645	ADA2
1646	MIR223
1647	LIPC
1648	TGFBR2
1649	NFE2L2
1650	BDNF
1651	LTA4H
1652	S100A4
1653	ATL1
1654	RELA
1655	TLR3
1656	TBXAS1
1657	RARA
1658	CASK
1659	KRT18
1660	SLC6A4
1661	TGM2
1662	CDKN1B
1663	FHL2
1664	GNB3
1665	GPD2
1666	NFATC1
1667	VCL
1668	PXN
1669	SP1

1670	BCAR1
1671	DROSHA
1672	WASL
1673	SH3KBP1
1674	WWTR1
1675	STOM
1676	IGES
1677	AKT1
1678	ATM
1679	CDK4
1680	CREBBP
1681	PIK3CA
1682	PTPN11
1683	TYK2
1684	AURKA
1685	CHUK
1686	NFKB2
1687	PTPRC
1688	NFKBIA
1689	PIK3R2
1690	ITGA5
1691	MAPK8
1692	MST1R
1693	NRAS
1694	RXRA
1695	STK11
1696	BAP1
1697	CCND2
1698	IKBKG
1699	MITF
1700	TYR
1701	BARD1
1702	BIRC3
1703	BIRC5
1704	CCND3
1705	PAX5
1706	SH2B3
1707	TYRP1
1708	ZIC3
1709	BIRC2
1710	CD33
1711	NKX2-1
1712	TET2
1713	XBP1

1714	APTX
1715	CFLAR
1716	KDM5C
1717	LATS1
1718	PRDM1
1719	TNFRSF10A
1720	TNFRSF21
1721	CRBN
1722	JAG2
1723	LILRB1
1724	NCOR2
1725	SFRP1
1726	TNFSF10
1727	DIS3
1728	ERF
1729	HMMR
1730	HPSE2
1731	OSM
1732	RECQL4
1733	TRIM21
1734	TRNT1
1735	COQ5
1736	IL7
1737	LIF
1738	MEOX2
1739	SFRP2
1740	SLC22A11
1741	TGFB1I1
1742	TK2
1743	TRAF5
1744	EIF1AX
1745	TET3
1746	BBC3
1747	NDUFB4
1748	SCARF1
1749	AFAP1L2
1750	ARID4A
1751	CCL4
1752	MGA
1753	MX2
1754	SHROOM3
1755	TNFRSF10C
1756	ADCK1
1757	DAZAP2

1758	RMND1
1759	SPA17
1760	UPK1A
1761	UPK1B
1762	VWA2
1763	HTRA4
1764	NSD2
1765	P2RY8
1766	BCORL1
1767	H1-4
1768	LRRC56
1769	FAM135A
1770	PTPRQ
1771	CST9
1772	CTAG1B
1773	MYEOV
1774	SFTA3
1775	UPK3B
1776	FAM170B
1777	PVT1
1778	CCDC160
1779	GAS5
1780	TP73-AS1
1781	UCA1
1782	MIR342
1783	MIRLET7E
1784	MIR335
1785	MIR99B
1786	MIRLET7B
1787	MIR561
1788	PCAT1
1789	LINC01194
1790	MIR1915
1791	ST3GAL6-AS1
1792	CCAT1
1793	TET2-AS1
1794	LAMA5-AS1
1795	LINC01514
1796	PDIA3P1
1797	PKD2
1798	ADAM17
1799	HSPB1
1800	CDH1
1801	PSMB8

1802	PDGFB
1803	FGB
1804	PGM1
1805	GPX4
1806	NEDD4L
1807	NR1H2
1808	VDAC1
1809	ANGPT1
1810	LTF
1811	TFPI
1812	TXN
1813	C9
1814	CAMK4
1815	LGALS3
1816	ARNT
1817	BSG
1818	DNASE1
1819	DYSF
1820	HBA1
1821	HBA2
1822	HLA-DQA1
1823	LBP
1824	DKK3
1825	ELAVL1
1826	SV2B
1827	SORCS1
1828	RLN2
1829	ADM2
1830	CD24
1831	CGB5
1832	FABP12
1833	PATJ
1834	MIR29B1
1835	LOC110283621
1836	EZH2
1837	GAPDH
1838	DICER1
1839	FLI1
1840	FOLR1
1841	HSPA8
1842	CEBPA
1843	CSTB
1844	HTR7
1845	KLK3

1846	MS4A1
1847	PLA2G2A
1848	ACTG2
1849	ALPP
1850	CPE
1851	MYO6
1852	PRF1
1853	SFTPB
1854	COL6A3
1855	EZR
1856	SLC6A19
1857	ACP2
1858	AICDA
1859	CD2
1860	CHMP2B
1861	COL6A1
1862	CPM
1863	GHRL
1864	GIPC1
1865	HGD
1866	IGFBP2
1867	ITIH4
1868	LAMC1
1869	MAP3K14
1870	POSTN
1871	IGFBP4
1872	LGALS1
1873	NOX4
1874	ADAM28
1875	ARHGEF7
1876	CXCL13
1877	OGN
1878	TNFRSF4
1879	SLPI
1880	GUCA2B
1881	ITLN1
1882	BCL2L14
1883	COL20A1
1884	GPRC5B
1885	KLF17
1886	WFDC2
1887	PEBP4
1888	PHB1
1889	ADPRH

1890	DDN
1891	MIR30C1
1892	MIR216A
1893	MIR638
1894	MIR198
1895	HORMAD2-AS1
1896	DEFA9P
1897	PRKCA
1898	RAF1
1899	MAP2K1
1900	MDM2
1901	BCR
1902	ESR1
1903	IKBKB
1904	ITK
1905	APP
1906	BCHE
1907	CA2
1908	DDC
1909	FLT4
1910	MMP14
1911	TERT
1912	TH
1913	ABCA3
1914	EGLN1
1915	MAOA
1916	MAPT
1917	MARK3
1918	MSH6
1919	NPR2
1920	PTCH1
1921	PTPRF
1922	SLC12A2
1923	ADRB2
1924	C1S
1925	NRXN1
1926	TBK1
1927	YWHAG
1928	ADSL
1929	COL2A1
1930	CTBP1
1931	CYP2D6
1932	DNM1L
1933	EPOR

1934 GABRA1
1935 GALK1
1936 GCH1
1937 GLI2
1938 HSPD1
1939 KCNJ11
1940 MAPK9
1941 MPL
1942 OAT
1943 PIKFYVE
1944 RUNX1
1945 SQSTM1
1946 TNNT2
1947 TRPC3
1948 WNK1
1949 ABCC4
1950 AK1
1951 AMPD1
1952 ATRX
1953 CASP4
1954 COL5A1
1955 CR2
1956 DHCR7
1957 EDN3
1958 EGLN3
1959 ETS1
1960 FEN1
1961 GCGR
1962 GLI1
1963 HADHA
1964 HTR1A
1965 HTR3A
1966 IHH
1967 LIG1
1968 LIMK2
1969 LOX
1970 MYH14
1971 MYH2
1972 NCOA3
1973 NDUFS7
1974 NOG
1975 OGDH
1976 OTC
1977 PC

1978	PDP1
1979	PDX1
1980	PINK1
1981	PKLR
1982	POLD1
1983	PRNP
1984	RUNX2
1985	RXRB
1986	RYR1
1987	SLC5A2
1988	TBX5
1989	TRPM6
1990	TRPV1
1991	TYMP
1992	YWHAB
1993	ARL3
1994	ASL
1995	BECN1
1996	BLNK
1997	BMX
1998	BTRC
1999	CALCRL
2000	CCNA2
2001	CCNH
2002	CDK7
2003	CDON
2004	CHRNA7
2005	DLL1
2006	EHMT1
2007	EHMT2
2008	FCN3
2009	GNAI3
2010	GP9
2011	HELLS
2012	KCNJ10
2013	LSS
2014	MEN1
2015	NCOA1
2016	NCOA2
2017	NDUFS1
2018	NGFR
2019	RAD23B
2020	SH2D1A
2021	SMARCB1

2022	STAG2
2023	SUOX
2024	SYNJ1
2025	SYP
2026	TEC
2027	TGFA
2028	TWIST1
2029	TXK
2030	VIPR1
2031	WARS2
2032	WNK4
2033	YWHAQ
2034	ZIC1
2035	ABHD5
2036	ACAD9
2037	ADAR
2038	ADCY10
2039	ALAS2
2040	AMD1
2041	AP2M1
2042	BCL6
2043	CHGA
2044	CS
2045	CSF2RA
2046	EGLN2
2047	EYA4
2048	FOXP2
2049	FZD9
2050	GBF1
2051	LARS2
2052	MED12
2053	MMAB
2054	NDUFS2
2055	NEK1
2056	NR4A3
2057	PDYN
2058	PLN
2059	PMPCA
2060	PNPLA2
2061	SCO2
2062	SLC7A5
2063	SMARCC2
2064	TIMP3
2065	ALDH4A1

2066	ASCL1
2067	ATP13A2
2068	CHIA
2069	COL11A2
2070	COL5A2
2071	COX5A
2072	DLX5
2073	E2F1
2074	EEF1A1
2075	EFNB2
2076	EGR1
2077	ELAC2
2078	EXO1
2079	FBXW11
2080	FMR1
2081	FOXG1
2082	GJA4
2083	GYS2
2084	HTT
2085	IGLL1
2086	ITGA9
2087	KAT6A
2088	LRPPRC
2089	MAOB
2090	MKI67
2091	MLX
2092	NDUFS4
2093	NDUFV2
2094	NEUROD1
2095	NLRC4
2096	NLRP1
2097	NR2C2
2098	PAX4
2099	PCDH19
2100	PI4KB
2101	PNMT
2102	POLL
2103	POLR2B
2104	PPP1R12A
2105	PROX1
2106	PUS1
2107	RARS2
2108	REST
2109	RNASEH2A

2110 SFTPD
2111 SLC25A24
2112 SLC4A2
2113 SLC7A11
2114 SLC7A6
2115 SSTR1
2116 STK39
2117 TFAM
2118 VIPR2
2119 WIF1
2120 XPR1
2121 XRCC5
2122 AK3
2123 ANK2
2124 ARHGEF6
2125 BPI
2126 CCL11
2127 CCNF
2128 CCS
2129 CDK13
2130 CHGB
2131 CHRD
2132 COLEC11
2133 COMMD1
2134 COPS5
2135 CUL2
2136 DARS2
2137 DCTD
2138 DGKQ
2139 EFTUD2
2140 FBL
2141 FKBP10
2142 FOXC1
2143 FOXF1
2144 FXYD2
2145 GAP43
2146 GAS1
2147 GTF2H1
2148 ICMT
2149 IGFBP6
2150 ITPKC
2151 KLHL3
2152 MPV17
2153 MTO1

2154 NDUFB3
2155 NSUN2
2156 OPLAH
2157 PLXND1
2158 PRRX1
2159 PSMC5
2160 PTF1A
2161 RAB3A
2162 RAMP2
2163 RNMT
2164 RPS13
2165 SEC31A
2166 SLC1A7
2167 SLC22A8
2168 SLC31A1
2169 SLC7A2
2170 SNAP23
2171 STAG3
2172 TBX20
2173 TBX4
2174 TFB1M
2175 TFPI2
2176 TNFRSF9
2177 TRIT1
2178 UBE3B
2179 UNC119
2180 UTS2R
2181 ZIC2
2182 ARHGAP31
2183 ATP6V1C2
2184 AUTS2
2185 BRD1
2186 BRPF1
2187 CCNC
2188 CD5
2189 CDC5L
2190 CEP55
2191 CHRDL1
2192 CNOT1
2193 CNTN4
2194 COX4I2
2195 COX5B
2196 DECR1
2197 DGCR2

2198	FCN1
2199	FLVCR1
2200	G6PC3
2201	GCG
2202	GJC2
2203	GTPBP3
2204	HHIP
2205	HIP1
2206	IARS2
2207	ICAM5
2208	INPP5A
2209	LRRC8A
2210	MAP1LC3A
2211	MATN1
2212	MCPH1
2213	MDC1
2214	MRC1
2215	MRPL44
2216	NEK3
2217	PIGT
2218	PYCARD
2219	RAB1B
2220	RAB33B
2221	RAD52
2222	RAPGEF1
2223	RING1
2224	RYR3
2225	SCARF2
2226	SFXN4
2227	SLC2A6
2228	SLC39A5
2229	SLC7A8
2230	SOX18
2231	STIL
2232	STX11
2233	TDGF1
2234	THBS3
2235	UBR5
2236	ANKRD26
2237	BOC
2238	CALB2
2239	CETN2
2240	CLDN3
2241	CLDN4

2242	DNAJB11
2243	GLRX5
2244	GTF2H4
2245	HCRT
2246	HLA-DOA
2247	HSD17B8
2248	IFT81
2249	IMPG2
2250	INPP5J
2251	ITGAE
2252	LBX1
2253	LDHD
2254	MMAA
2255	MRPL12
2256	NEDD8
2257	NEU2
2258	NPAS3
2259	PPP2R3C
2260	RAB1A
2261	RAI1
2262	RNF7
2263	SCG2
2264	SLC17A1
2265	TBCK
2266	TMSB4X
2267	USH1G
2268	VPS29
2269	VPS41
2270	WDR45
2271	WNK3
2272	AAGAB
2273	ADI1
2274	ANKK1
2275	AP1S1
2276	ASPM
2277	ATOX1
2278	ATP6V0D2
2279	C5AR2
2280	CCL17
2281	COX6C
2282	CREB5
2283	DISP1
2284	DLX6
2285	DTL

2286	FBXL4
2287	GOLM1
2288	GYG2
2289	IBTK
2290	IQSEC2
2291	KANSL1
2292	KCNJ16
2293	KCTD7
2294	KLF11
2295	LAMP3
2296	LDB1
2297	LDB2
2298	LONP2
2299	MAFG
2300	MAFK
2301	MED13
2302	MID2
2303	MSTO1
2304	MTX1
2305	NDUFB6
2306	NEIL1
2307	NEIL2
2308	NHLRC1
2309	NIF3L1
2310	NKX2-3
2311	NSUN4
2312	P2RX5
2313	PDLIM5
2314	PIGH
2315	PMM1
2316	PPME1
2317	PPP2R5A
2318	PRRT2
2319	RBMS1
2320	RNASEH2C
2321	RUNX1T1
2322	SACM1L
2323	SIM2
2324	SLC5A4
2325	SYNJ2
2326	TELO2
2327	TEX14
2328	TGIF2
2329	TMEM70

2330 UFM1
2331 USF2
2332 VGF
2333 VPREB1
2334 ZNF644
2335 ABHD11
2336 AGBL2
2337 AIF1
2338 ARSD
2339 ATP6V1G1
2340 ATP6V1G2
2341 BRD8
2342 CD52
2343 CDK5RAP1
2344 CDK5RAP3
2345 CFDP1
2346 COPS7B
2347 DCAF8
2348 DCHS1
2349 DDRGK1
2350 DDX54
2351 ESPN
2352 EYA3
2353 FMN1
2354 GPS1
2355 GTF2H3
2356 GUCY2F
2357 HESX1
2358 HNRNPH2
2359 HYDIN
2360 IFIT1
2361 IL32
2362 ING5
2363 INSM1
2364 LAT2
2365 MATN4
2366 MRRF
2367 MYBPH
2368 MYH7B
2369 NAALADL2
2370 NADK2
2371 NSUN3
2372 NSUN5
2373 PENK

2374	PMF1
2375	PNOC
2376	POLR2I
2377	POU2AF1
2378	PRDM9
2379	PRKN
2380	RALGAPA2
2381	SCUBE1
2382	SHPK
2383	SLC25A38
2384	SLC37A1
2385	SNRPF
2386	SNTB1
2387	SSBP3
2388	TSPAN2
2389	WDR62
2390	ZIC4
2391	ZNF195
2392	ACAD11
2393	AGBL1
2394	CAPN8
2395	CCDC115
2396	CCL26
2397	CENPB
2398	COX7C
2399	EAPP
2400	EMP1
2401	EOGT
2402	FBXW5
2403	FOXE3
2404	FOXL1
2405	GDF1
2406	GTF2H2
2407	INTS9
2408	ISL2
2409	KAZALD1
2410	LXN
2411	MBD6
2412	MNT
2413	MRPL18
2414	MRPS27
2415	MXD1
2416	MXD4
2417	NOP2

2418	NSUN6
2419	P3H2
2420	RBMS2
2421	RBSN
2422	RNASEH2B
2423	RNF152
2424	SLC37A3
2425	SNRPA1
2426	STX18
2427	SUGCT
2428	TANC2
2429	TAX1BP3
2430	TBC1D5
2431	TWSG1
2432	ZRSR2
2433	BCL7A
2434	EPRS1
2435	FBXW4
2436	GBA3
2437	KIAA0586
2438	TMEM230
2439	TRMT112
2440	TSSK2
2441	TTI2
2442	WDR83
2443	AGBL3
2444	AGBL4
2445	ANKLE1
2446	ARRDC4
2447	BCL7C
2448	COBL
2449	DPCD
2450	DPH7
2451	FAM167A
2452	GPR22
2453	KLHDC1
2454	KRT33A
2455	MEAF6
2456	MLN
2457	MMUT
2458	MRPL45
2459	MRPS26
2460	MT-ND2
2461	NELFA

2462	THYN1
2463	TMEM184B
2464	TMTC4
2465	UBR3
2466	UFL1
2467	WSCD1
2468	ZIC5
2469	ARMC8
2470	CALN1
2471	CCDC86
2472	CREG2
2473	FOXN4
2474	FOXR1
2475	IFNA17
2476	LIME1
2477	MGME1
2478	MT-ND3
2479	MTERF1
2480	NSUN7
2481	NXT2
2482	OR4L1
2483	PCP2
2484	PCSK1N
2485	PPP1R3D
2486	SLTM
2487	TANC1
2488	TMEM147
2489	TRMT61B
2490	WHAMM
2491	ZACN
2492	ABHD14A
2493	C16orf54
2494	DGCR6L
2495	FBRS
2496	MTERF4
2497	PPP1R3E
2498	PRSS56
2499	SNAP47
2500	STING1
2501	STX19
2502	TRIM50
2503	UBXN7
2504	ZNF506
2505	ZNF621

2506	ASPHD1
2507	ELOB
2508	RAB7B
2509	SEM1
2510	ARL13A
2511	DCAF1
2512	GALNT17
2513	GTF2IRD2B
2514	MRM2
2515	MT-ATP8
2516	NXF5
2517	RFX8
2518	CFAP36
2519	CFAP47
2520	CGAS
2521	DZANK1
2522	INTS11
2523	POM121C
2524	SPDYE1
2525	TRIM74
2526	BHLHA9
2527	C5orf64
2528	MT-ND4L
2529	MTRFR
2530	SPDYE2
2531	SPRR2F
2532	SSC4D
2533	TMEM187
2534	TRIM73
2535	C16orf92
2536	CFC1B
2537	TAFA1
2538	H2BC3
2539	DNAAF9
2540	NSUN5P2
2541	TERB2
2542	RCC1L
2543	STAG3L1
2544	STAG3L4
2545	MIR145
2546	PMF1-BGLAP
2547	RTL9
2548	SMIM23
2549	MIR142

2550 MIR9-1
2551 DGCR5
2552 HLA-DPB2
2553 NSUN5P1
2554 STAG3L2
2555 MT-RNR2
2556 MIR521-1
2557 STAG3L3
2558 ABHD11-AS1
2559 MIR450B
2560 MT-RNR1
2561 LINC00851
2562 MIR762
2563 SPDYE7P
2564 CDIPTOSP
2565 GTF2IP1
2566 IGBP1P1
2567 MIR1538
2568 GTF2IRD2P1
2569 MMP2-AS1
2570 MT-TG
2571 FAM230A
2572 MIR3978
2573 MT-TD
2574 MT-TM
2575 THRIL
2576 SPDYE14
2577 SPDYE8
2578 MIR3680-1
2579 SPDYE10
2580 SPDYE12
2581 TRL-AAG2-3
2582 GTF2IP4
2583 SPDYE13
2584 SPDYE15
2585 SPDYE9
2586 EIF4HP1
2587 IGHV4-38-2
2588 WBSCR2
2589 WBSCR23
2590 LOC106029312
2591 LOC110008580
2592 RMD1
2593 AGMX2

2594	FRTS1
2595	LOC106029311
2596	LOC106029313
2597	LOC106627982
2598	LOC108228208
2599	LOC108228209
2600	LOC110599567
2601	SBE2
2602	TKCR
2603	BMIQ16
2604	NT5E
2605	SOD2
2606	ACTA2
2607	ACY1
2608	BTD
2609	LAMB3
2610	A2M
2611	FABP5
2612	NPPC
2613	PROCR
2614	TNFAIP1
2615	BHMT
2616	SDCBP
2617	AOC1
2618	QSOX1
2619	MPP7
2620	VASN
2621	ZFYVE28
2622	EGFR
2623	CTNNB1
2624	SIGLEC1
2625	PDCD1
2626	BDKRB1
2627	EIF2AK3
2628	HSPA5
2629	RHOA
2630	AOC3
2631	ILK
2632	PPARA
2633	PON2
2634	TNFRSF6B
2635	TNIP1
2636	CES5A
2637	INSR

2638	NOS1
2639	CDH3
2640	IRF5
2641	IRS1
2642	TFAP2A
2643	HRH4
2644	WNT1
2645	CYP4A11
2646	FABP2
2647	HSPA4
2648	IL1RL1
2649	WWC1
2650	ELMO1
2651	TNFSF4
2652	CC2D2A
2653	PLVAP
2654	BANK1
2655	PXK
2656	RTN1
2657	TRB
2658	EPHB4
2659	PRKACA
2660	RAC2
2661	SPR
2662	ACAT1
2663	CAPN1
2664	DNM1
2665	DNM2
2666	F12
2667	GNAQ
2668	GSR
2669	PHGDH
2670	PPIB
2671	TUBB
2672	ACTB
2673	ACTG1
2674	ADAM9
2675	AHCY
2676	ARG1
2677	EEF2
2678	FASN
2679	FGG
2680	PGK1
2681	PRKACB

2682	PTH1R
2683	STAT5B
2684	ABCB4
2685	ACTA1
2686	AKR1C4
2687	ALDH7A1
2688	ALDOA
2689	ANXA1
2690	ATP1B1
2691	ATP6V1B2
2692	CA1
2693	CDH11
2694	CEL
2695	CTSC
2696	CTSH
2697	ENO3
2698	FLNA
2699	FLNB
2700	GNA11
2701	GPI
2702	HSP90AB1
2703	IL2RB
2704	KLKB1
2705	PGD
2706	PROS1
2707	PTPRJ
2708	RDX
2709	SSTR2
2710	TUBB3
2711	ACLY
2712	ADH5
2713	AKR1C3
2714	ALDH1A1
2715	CA4
2716	CACNA2D1
2717	CLTC
2718	COL18A1
2719	FOLH1
2720	FTL
2721	GANAB
2722	GJB2
2723	GOT2
2724	GSTM3
2725	HPGD

2726	HRG
2727	ITGB5
2728	KRT1
2729	KRT8
2730	L1CAM
2731	LDHB
2732	LIFR
2733	MSN
2734	MYH11
2735	NEDD4
2736	NME2
2737	NPY1R
2738	PFN1
2739	PNP
2740	PPIA
2741	PPP2R1A
2742	PPP2R1B
2743	PRCP
2744	PTGER4
2745	PTPRD
2746	QDPR
2747	RAB11A
2748	RALA
2749	TMPRSS2
2750	TPI1
2751	TPMT
2752	TPT1
2753	UBE2N
2754	ACAT2
2755	ALDH1A3
2756	ATIC
2757	ATP6V1A
2758	BPGM
2759	CNTN1
2760	CRYAB
2761	CSPG4
2762	CTSZ
2763	DBI
2764	DSC2
2765	EFEMP1
2766	EPS8
2767	FBLN1
2768	FRK
2769	GLO1

2770	GNAL
2771	GOT1
2772	GP6
2773	HAVCR2
2774	HSPA1A
2775	IGFALS
2776	IGFBP7
2777	ITCH
2778	KLK2
2779	LAMA3
2780	LTBP1
2781	LTBP2
2782	MDH1
2783	MINPP1
2784	MYO5A
2785	NME1
2786	NQO2
2787	P2RX4
2788	PARK7
2789	PKM
2790	PRDX2
2791	PRDX6
2792	PRKAR2A
2793	PSMA7
2794	PTPRS
2795	SERPINA6
2796	SERPINI1
2797	SFN
2798	SHMT1
2799	SLC26A2
2800	SLC9A3R1
2801	SORD
2802	STXBP2
2803	SULT2B1
2804	TUBB4A
2805	UGDH
2806	USP14
2807	WWP2
2808	XPNPEP2
2809	ACO1
2810	ALDOB
2811	ANXA11
2812	CANT1
2813	CBR1

2814	CDH13
2815	CDH15
2816	CKB
2817	CRYZ
2818	EPX
2819	GNB1
2820	GSTO1
2821	HNRNPK
2822	HPN
2823	ITM2B
2824	PEBP1
2825	PRKAR2B
2826	PTPRG
2827	QPCT
2828	RNASET2
2829	SEMA3C
2830	TAP2
2831	ACP1
2832	ACTC1
2833	AKR1A1
2834	ANXA4
2835	ARF6
2836	ATP6V0D1
2837	BGN
2838	C6
2839	C7
2840	C8B
2841	CAPN5
2842	CEACAM1
2843	CEACAM5
2844	CFL2
2845	CMPK1
2846	CNP
2847	CORO1A
2848	CRYM
2849	DCXR
2850	DDAH1
2851	GGH
2852	GNA13
2853	GNA14
2854	GPLD1
2855	GSTA3
2856	ICOS
2857	KPNB1

2858	KRT10
2859	KRT13
2860	KRT6A
2861	LCP1
2862	MAN2A1
2863	MGAT1
2864	MYOF
2865	NANS
2866	NPEPPS
2867	PAM
2868	PDCD6IP
2869	PGAM1
2870	PGAM2
2871	PLSCR1
2872	PRPH
2873	PSMA1
2874	RALB
2875	ROBO4
2876	RTN4R
2877	SELENBP1
2878	SERPINA5
2879	SLC26A4
2880	SLC2A5
2881	SMS
2882	SRI
2883	TCP1
2884	THBS4
2885	TOLLIP
2886	TOM1
2887	UBE2D2
2888	AKR1C1
2889	ALDH9A1
2890	ANXA3
2891	ANXA7
2892	ARPC3
2893	ATP6V0A1
2894	ATP8A1
2895	CAPZA1
2896	CD9
2897	CHL1
2898	CHMP4B
2899	CIB1
2900	CILP
2901	CLDN2

2902	CYFIP2
2903	DPEP1
2904	FSTL1
2905	GM2A
2906	GNB2
2907	GNB4
2908	GPD1L
2909	GSTA1
2910	IL18R1
2911	ITIH3
2912	KLF6
2913	KRT2
2914	KRT3
2915	KRT9
2916	LDHC
2917	LGMN
2918	MLPH
2919	MPST
2920	MUC5B
2921	MYO1C
2922	MYOC
2923	NAAA
2924	PABPC1
2925	PAICS
2926	PCBP1
2927	PMP2
2928	PROZ
2929	PSMA2
2930	PYGB
2931	PZP
2932	RAB10
2933	RAB2A
2934	RHCG
2935	RNASE1
2936	S100A11
2937	SERPINA4
2938	SERPINB6
2939	SERPINB8
2940	SLC44A2
2941	SPINT1
2942	SSTR5
2943	UBB
2944	UBE2V2
2945	UGP2

2946	VPS4A
2947	WASF2
2948	XPNPEP1
2949	ACTR3
2950	AKR7A2
2951	ALOX15B
2952	ANGPTL6
2953	ANO6
2954	ANXA6
2955	APEH
2956	ARPC2
2957	ART3
2958	BAIAP2
2959	C8A
2960	CAP1
2961	CAPG
2962	CAPZB
2963	CC2D1A
2964	CCT2
2965	CCT3
2966	CCT4
2967	CDH16
2968	CLIC1
2969	CLTCL1
2970	COTL1
2971	CPN2
2972	CPVL
2973	DEFA1
2974	ENTPD2
2975	FDX1
2976	FGL2
2977	FLOT1
2978	FLOT2
2979	GMPPA
2980	GPRC5A
2981	GSTA2
2982	GSTM2
2983	IL18BP
2984	ITIH1
2985	ITIH2
2986	KLK11
2987	LUM
2988	MAN1A1
2989	MUC4

2990	NAPSA
2991	NT5C
2992	NUCB1
2993	ORM1
2994	PCBP2
2995	PCDH12
2996	PCMT1
2997	PDCD10
2998	PELI1
2999	PGLYRP1
3000	PIGR
3001	PPIC
3002	PSMB2
3003	PTGER1
3004	QPRT
3005	RAB5B
3006	REG1A
3007	RHOQ
3008	RNPEP
3009	RP2
3010	RPE
3011	S100A1
3012	SERPINA10
3013	SLC44A4
3014	SLC5A8
3015	SLURP1
3016	SPPL2A
3017	ST3GAL6
3018	STEAP4
3019	STK25
3020	TAGLN2
3021	TGM4
3022	TUBB6
3023	UBA52
3024	UBE2V1
3025	VPS4B
3026	WASF3
3027	WWP1
3028	AFM
3029	ALDH1L2
3030	AMY2B
3031	AZU1
3032	BHMT2
3033	C1RL

3034	CAMP
3035	CCT6A
3036	CLEC3B
3037	CST6
3038	CYFIP1
3039	DPP7
3040	EIF6
3041	GCA
3042	GP5
3043	HPR
3044	HSPA1B
3045	LILRB4
3046	LRG1
3047	MGAT5
3048	MYL6
3049	OMD
3050	OTUB1
3051	PSCA
3052	PSME2
3053	RAB5C
3054	RHOG
3055	RNASE2
3056	S100A7
3057	SEC14L2
3058	SPON2
3059	TSPAN1
3060	ACTN3
3061	ACTR1A
3062	ACTR3B
3063	ADGRG1
3064	ALDH1L1
3065	AMY2A
3066	ARF5
3067	BAIAP2L1
3068	C8G
3069	CAB39
3070	CAPZA2
3071	CD177
3072	CDHR5
3073	CHMP2A
3074	CHMP5
3075	COL15A1
3076	CPNE1
3077	CPNE6

3078	CREG1
3079	CTDSP1
3080	DDT
3081	DEFB1
3082	DSC1
3083	EEF1G
3084	EPS8L2
3085	FAM3C
3086	FIGNL1
3087	GDI2
3088	GLG1
3089	GNG4
3090	HBZ
3091	HNRNPM
3092	KRT6C
3093	LYPLA1
3094	MAN1C1
3095	MAN2B2
3096	MAT2B
3097	MYO1D
3098	NECTIN4
3099	NID2
3100	OLFM4
3101	PCOLCE
3102	PDCD6
3103	PGK2
3104	PGLYRP2
3105	PIP
3106	PPT2
3107	RAB3D
3108	SERPINB9
3109	SERPINI2
3110	SPTBN4
3111	STK26
3112	SYT7
3113	TKFC
3114	TSPAN8
3115	TUBB8
3116	VPS25
3117	A1BG
3118	ACTR1B
3119	AKR7A3
3120	ALDOC
3121	ANGPTL2

3122	ARF3
3123	ATRN
3124	BASP1
3125	BCAS1
3126	CAPN7
3127	CD248
3128	CHMP1B
3129	CPNE3
3130	CPNE4
3131	CRISPLD2
3132	CRTAC1
3133	FLRT2
3134	GALNT7
3135	GNG12
3136	GNG7
3137	IST1
3138	ITFG1
3139	KRT71
3140	KRT74
3141	LMAN2
3142	LRRC15
3143	MYL6B
3144	MYO5C
3145	NAPA
3146	NAPB
3147	NAPRT
3148	NUTF2
3149	OSCAR
3150	PACSIN3
3151	PBLD
3152	PDLIM2
3153	PGM2
3154	PLA2G15
3155	PPP1R7
3156	RAB27B
3157	RAB32
3158	REG1B
3159	RHOJ
3160	RTN4RL1
3161	SCPEP1
3162	SECTM1
3163	SIAE
3164	SLC5A10
3165	SLC9A3R2

3166	SMPDL3A
3167	SUSD2
3168	TOM1L2
3169	TSPAN6
3170	TXNDC5
3171	VPS28
3172	VWA1
3173	ATP5F1A
3174	BDH2
3175	CDHR2
3176	CILP2
3177	CORO1B
3178	CPNE7
3179	CPQ
3180	DEFA3
3181	EHD4
3182	EPS8L1
3183	FETUB
3184	GLIPR2
3185	GLOD4
3186	GPRC5C
3187	GSTA5
3188	IL23A
3189	ISLR
3190	KRT25
3191	KRT75
3192	LDHAL6A
3193	MEGF8
3194	ORM2
3195	PABPC3
3196	PGLS
3197	PI15
3198	PSMA8
3199	RAB21
3200	RAB3B
3201	RHOF
3202	RNH1
3203	SDCBP2
3204	SNX18
3205	TRHDE
3206	TXNDC17
3207	VAT1
3208	WARS1
3209	CPNE8

3210	CRYL1
3211	CUTA
3212	FCGBP
3213	GSTT2
3214	HJV
3215	JCHAIN
3216	KIF12
3217	KRT24
3218	KRT31
3219	KRT77
3220	KRT79
3221	NECTIN2
3222	PEF1
3223	PROM2
3224	SARS1
3225	SEC14L3
3226	SYTL1
3227	TBC1D10A
3228	TMBIM1
3229	TOM1L1
3230	UEVLD
3231	VTA1
3232	ABHD14B
3233	ADSS2
3234	AMY1A
3235	ARL15
3236	B3GNT8
3237	CPNE5
3238	CPNE9
3239	DNPH1
3240	GGT6
3241	GSTT2B
3242	H2AX
3243	IL26
3244	KRT28
3245	KRT35
3246	KRT38
3247	KRT76
3248	LRRC57
3249	MELTF
3250	MMRN2
3251	MXRA8
3252	PGA5
3253	PGAM4

3254	PTTG1IP
3255	RAB2B
3256	RTN4RL2
3257	SEMG1
3258	SEMG2
3259	SH3BGRL3
3260	TMC4
3261	VPS36
3262	VPS37C
3263	WTIP
3264	ZG16B
3265	ACP3
3266	ARRDC1
3267	ATP5F1B
3268	B4GAT1
3269	BPIFB2
3270	CHTF8
3271	CPNE2
3272	FAM151A
3273	GDPD3
3274	HDHD2
3275	HID1
3276	KRT26
3277	KRT32
3278	KRT36
3279	KRT72
3280	KRT73
3281	KRT84
3282	NAXE
3283	PABPC1L
3284	PGA3
3285	PLPP1
3286	PTPA
3287	TMC5
3288	TMEM132A
3289	TTC38
3290	TTYH3
3291	VPS37B
3292	C11orf54
3293	CCN3
3294	CRISPLD1
3295	ENDOD1
3296	GGTLC2
3297	KRT27

3298	KRT37
3299	LRRN4
3300	MACROH2A1
3301	MITD1
3302	MON2
3303	AMY1C
3304	C16orf89
3305	FAM168B
3306	GGACT
3307	PALS1
3308	POTEE
3309	ADIRF
3310	DDTL
3311	H2AZ1
3312	H4-16
3313	IGHG2
3314	SELENOP
3315	VMO1
3316	ACTR3C
3317	AKR7L
3318	BROX
3319	FOLH1B
3320	H2AC11
3321	H4C2
3322	IGHG3
3323	IGHG4
3324	IGLL5
3325	NIBAN1
3326	NIBAN2
3327	PGA4
3328	AMY1B
3329	C11orf52
3330	DEFA1B
3331	H2AC1
3332	H2AC13
3333	H4C11
3334	H4C3
3335	H4C8
3336	H4C9
3337	IGHA2
3338	POTEF
3339	SMR3B
3340	H2AW
3341	H4C12

3342	H4C14
3343	H4C5
3344	MACROH2A2
3345	PABPC1L2A
3346	PPIAL4A
3347	UBE2NL
3348	DOP1B
3349	H2AC12
3350	H2AC15
3351	H2AC21
3352	H2AC6
3353	H2AC7
3354	H2AJ
3355	H2AZ2
3356	H4C13
3357	H4C4
3358	H4C6
3359	PGAP6
3360	POTEJ
3361	H2AC8
3362	HEXD
3363	IGHD
3364	IGLC1
3365	POTEI
3366	TCL6
3367	H4C15
3368	HSPA7
3369	ANXA2P2
3370	PABPC1L2B
3371	TNXA
3372	GGTLC3
3373	NME2P1
3374	GGT2P
3375	MIR200B
3376	POTEKP
3377	PPIAL4C
3378	HSP90AA2P
3379	IGKV1-5
3380	MIR106A
3381	GGT3P
3382	HSP90AB2P
3383	HSP90AB3P
3384	IGLV3-21
3385	H2AC19

3386	MIR19B1
3387	IGHV3-7
3388	IGLV1-51
3389	IGLV3-19
3390	IGKV2D-28
3391	IGKV3-20
3392	IGLV2-14
3393	IGKV1-17
3394	IGKV1D-33
3395	IGKV2-30
3396	IGKV1-39
3397	IGKV2-40
3398	IGKV3D-11
3399	RNY5
3400	TUBB8B
3401	DERPC
3402	IGKV1D-12
3403	IGHV3-9
3404	NPEPPSP1
3405	LOC440786
3406	AR
3407	ALDH2
3408	STAT6
3409	FOXO1
3410	ITGB4
3411	CYBB
3412	C1QA
3413	PITX2
3414	DAO
3415	ERAP1
3416	GAK
3417	SLC22A2
3418	ADH1B
3419	CD274
3420	VAMP1
3421	PEX19
3422	GMFB
3423	CLEC4A
3424	TPPP3
3425	MIR23A
3426	MIR195
3427	MIR30A
3428	MIR29C
3429	CYP3A4

3430	KCNH2
3431	TGFB2
3432	JAG1
3433	PCSK1
3434	EXT1
3435	GSK3B
3436	ADRB1
3437	ALDH1A2
3438	CES1
3439	MAP2K3
3440	NQO1
3441	PIK3CG
3442	SCNN1A
3443	SLC22A5
3444	SLC29A1
3445	ADORA2B
3446	CNR1
3447	HMOX2
3448	MAP2K4
3449	P2RX7
3450	SLC18A2
3451	SORL1
3452	ACADM
3453	ATP2A3
3454	C1QC
3455	CYB5R3
3456	EXT2
3457	KHK
3458	MAP2K6
3459	NR2F1
3460	PON3
3461	DRD3
3462	HDAC7
3463	HIPK2
3464	IL1RAP
3465	INVS
3466	SLC22A3
3467	CD86
3468	GADD45A
3469	GLRX
3470	KDM6B
3471	MAP2
3472	PDK2
3473	SLC22A1

3474	SLC29A4
3475	SMAD7
3476	SOCS1
3477	VEGFB
3478	ALMS1
3479	AREG
3480	ATG7
3481	C4BPA
3482	CA3
3483	CXCR1
3484	IQCB1
3485	MGP
3486	S100A6
3487	SOCS3
3488	XPNPEP3
3489	AIM2
3490	CCL21
3491	CRHBP
3492	CXCL1
3493	ECT2
3494	GLIS2
3495	GPC5
3496	IL13RA1
3497	KLF15
3498	PLIN2
3499	PLIN3
3500	RIOK2
3501	EDIL3
3502	ST8SIA4
3503	ZNF423
3504	CD5L
3505	CXCL2
3506	DCDC2
3507	FERMT2
3508	IL13RA2
3509	MYO9A
3510	RNLS
3511	RPGRIP1L
3512	ANKS6
3513	FGF21
3514	IL1F10
3515	IL33
3516	MANF
3517	MUC20

3518	NDST2
3519	RHPN1
3520	SESN2
3521	TMEM67
3522	ACKR1
3523	CSN1S1
3524	SRGAP2
3525	TMSB10
3526	CASP12
3527	MIR196A2
3528	SNHG5
3529	DLX6-AS1
3530	MIR31
3531	MIR130B
3532	MIR939
3533	KRT18P42
3534	KRT18P16
3535	ERBB4
3536	MGMT
3537	PCSK9
3538	ABCG2
3539	GLUD1
3540	PAK3
3541	PPP2CA
3542	PRKCH
3543	PROC
3544	WNT5A
3545	ESR2
3546	PLA2G4A
3547	TPO
3548	BRCA2
3549	CYP11A1
3550	RPS6KB1
3551	TNFAIP3
3552	TYMS
3553	ADORA1
3554	ADRA2A
3555	CLCN2
3556	FGF1
3557	GPX1
3558	HSP90B1
3559	IRAK4
3560	KDM6A
3561	MAP3K5

3562	NCF2
3563	PIN1
3564	PRSS1
3565	PTGS1
3566	STS
3567	TGM1
3568	TRAF3
3569	TRPV6
3570	UBE2I
3571	AQP3
3572	ARRB2
3573	BMP2
3574	CA8
3575	CDK1
3576	CISH
3577	CLDN1
3578	EFNB1
3579	FOXO3
3580	FPR2
3581	GFPT1
3582	GPC3
3583	HRH1
3584	LAMA4
3585	NTN1
3586	PPARGC1A
3587	SLC29A2
3588	SPTBN1
3589	STAR
3590	TAT
3591	TMPO
3592	TRAF6
3593	UGT1A9
3594	C1QB
3595	COL6A2
3596	COL7A1
3597	GAL
3598	GPC4
3599	MMP16
3600	NDRG1
3601	OAS1
3602	SOAT1
3603	TG
3604	UBE2D1
3605	ATP6AP1

3606	CD74
3607	CEBPB
3608	CLIC5
3609	DDX1
3610	EEA1
3611	FLII
3612	GIPR
3613	HRH2
3614	ID2
3615	IL12B
3616	MTM1
3617	NFATC4
3618	ORAI1
3619	PALLD
3620	PREX1
3621	RASSF1
3622	SLC13A3
3623	SOCS2
3624	ST6GAL1
3625	TIA1
3626	TNFRSF13C
3627	ELK1
3628	IKZF3
3629	IL21R
3630	KLF10
3631	MCAM
3632	PARVA
3633	PICK1
3634	PRDX4
3635	PTAFR
3636	SPTB
3637	TPBG
3638	VAV2
3639	ACKR3
3640	CLDN5
3641	DAAM1
3642	EGFL7
3643	ERP44
3644	GCLM
3645	GUK1
3646	IL22
3647	IQGAP2
3648	PAEP
3649	PCYT1B

3650	PDZK1
3651	RB1CC1
3652	RND3
3653	RPLP2
3654	SCN7A
3655	SORBS1
3656	SULF2
3657	TSEN2
3658	ZFYVE9
3659	BHLHE40
3660	CBLN1
3661	CXXC1
3662	EML1
3663	IRAK2
3664	JDP2
3665	LIMS1
3666	STK16
3667	VTCN1
3668	CELA3B
3669	CRLS1
3670	FN3K
3671	GPR4
3672	GSTK1
3673	KIF3B
3674	MAGI1
3675	MICA
3676	RHPN2
3677	SFXN1
3678	TMEM30A
3679	ACKR2
3680	ARHGAP35
3681	ATG10
3682	CORO2B
3683	HSPA13
3684	KCMF1
3685	LAPTM4A
3686	LAPTM5
3687	OLFM3
3688	ORAI2
3689	SDK1
3690	SNRNP70
3691	SNRPD1
3692	TAF9
3693	NCKIPSD

3694	PHLDA2
3695	VASH1
3696	AHSP
3697	EPB41L5
3698	HSD17B14
3699	LVRN
3700	RNPC3
3701	SCHIP1
3702	ACTL7A
3703	ACTL7B
3704	ADCK2
3705	CENPI
3706	FCRLB
3707	GPHA2
3708	NOMO1
3709	PSG5
3710	UBE2W
3711	ASB4
3712	H1-5
3713	IFNL3
3714	ORAI3
3715	SLC25A51
3716	STOX2
3717	YPEL1
3718	LLPH
3719	TMEM128
3720	HLA-DRB3
3721	JAML
3722	H3C14
3723	MIR204
3724	MIR34A
3725	MIR141
3726	MIR149
3727	MIR150
3728	MIRLET7C
3729	MIR124-1
3730	MIR148B
3731	MIR196B
3732	MIR199A2
3733	MIR205
3734	MIR221
3735	MIR615
3736	MIR138-1
3737	MIR199A1

3738	MIR217
3739	MIR483
3740	MIR675
3741	MIR503HG
3742	MIR518B
3743	MIR519D
3744	MIR873
3745	SNHG4
3746	LINC01619
3747	MIR18B
3748	MIR363
3749	MIR325
3750	MIR548C
3751	MIR576
3752	LOC157273
3753	MIR4767
3754	HFE-AS1
3755	MBL3P
3756	SRMP1
3757	LOC105374325
3758	MSBP1
3759	DEL22Q11.2
3760	HNP1
