# Supplementary material for: Niaoduqing alleviates podocyte injury in high glucose model via regulating multiple targets and AGE/RAGE pathway: Network pharmacology and experimental validation
Source: Front Pharmacol. 2023 Feb 27;14:1047184. doi: 10.3389/fphar.2023.1047184 (PMC10009170; doi:10.3389/fphar.2023.1047184)
Supplement: Supplementary file 11 [file Table3.pdf]

Table S3 The targets list of Niaoduqing

	Target
1	HSD11B1
2	HSD11B2
3	YWHAG
4	HPGD
5	HSD17B14
6	CYP27B1
7	PSMD3
8	PSMB1
9	HSD3B1
10	HSD3B2
11	HSD17B7
12	PDPK1
13	HTR1A
14	HTR1B
15	HTR2A
16	HTR2C
17	HTR3A,
18	ALOX5AP
19	PFKFB3
20	PGD
21	NUDT1
22	MMP2
23	HSPA5
24	CHRNB1
25	ACHE
26	ACACA
27	ACACB
28	SLC33A1
29	ASAH1
30	FGF1
31	CHIA
32	AHSA1
33	CES1
34	SOAT1
35	SCD
36	LYPLA2
37	ADAM10
38	ADAM17
39	ADAM9
40	ADAMTS4
41	ADAMTS5

42	ADORA1
43	ADORA2A
44	ADORA2B
45	ADORA3
46	ADA
47	ADK
48	ADCY10
49	ADCY2
50	ADCY5
51	ADRA2C
52	ADRB2
53	ADH1B
54	ADH1C
55	ADH1A
56	ALDH2
57	ALDH1A1
58	ALDH3A1
59	AKR1A1
60	AKR1B10
61	AKR1C1
62	AKR1C2
63	AKR1C3
64	AKR1C4
65	AKR1B1
66	ALK
67	ALPL
68	FUT7
69	ADRA1A
70	ADRA1B
71	ADRA1D
72	ADRA2A
73	ADRA2B
74	ALKBH3
75	FTO
76	FUCA1
77	MAOA
78	MAOB
79	AOC3
80	RNPEP
81	ANPEP
82	AMPD1
83	AMPD2
84	AMPD3
85	AMY1A

86	APP
87	FAAH
88	AR
89	ACE
90	EBP
91	DHCR7
92	SLPI
93	APOD
94	BAX
95	BCL2
96	BCL2L1
97	ALOX12
98	ALOX15
99	ALOX15B
100	ALOX5
101	ARG1
102	AHR
103	NAT1
104	DNPEP
105	ATP5F1E
106	ABCG2
107	ACLY
108	KCNJ1
109	AURKAIP1
110	ENPP2
111	CTNNB1
112	XIAP
113	BIRC5
114	FGF2
115	BAD
116	BCL2A1
117	BACE2
118	ADRB1
119	ADRB3
120	ST6GAL1
121	GBA
122	GBA2
123	GUSB
124	DPEP1
125	BACE1
126	OGA
127	NR1H4
128	SLC10A1
129	BRS3

130	BMP1
131	BDKRB1
132	BDKRB2
133	ADCY1
134	BAZ2A
135	BAZ2B
136	BRD2
137	BRD3
138	BRD4
139	BCHE
140	C5AR1
141	CALCRL
142	CALCR
143	CASR
144	KCNMA1
145	CALM1
146	CAPN1
147	CAPN2
148	CAMK2D
149	CAMK2B
150	CAMKK2
151	PRKACA
152	PKIA
153	CNR1
154	CNR2
155	CA1
156	CA2
157	CA3
158	CA4
159	CA9
160	CA5A
161	CA5B
162	CA6
163	CA7
164	CA12
165	CA13
166	CA14
167	CBR1
168	CES2
169	CPB1
170	CSNK1A1
171	CSNK1D
172	CSNK1G1
173	CSNK1G2

174	CSNK2A1
175	CSNK2A2
176	CASP1
177	CASP2
178	CASP3
179	CASP6
180	CASP7
181	CASP8
182	CASP9
183	CAT
184	COMT
185	CTSB
186	CTSH
187	CTSV
188	CTSD
189	CTSF
190	CTSG
191	CTSK
192	CTSL
193	CTSS
194	CAV1
195	CCR1
196	CCR4
197	CCR5
198	CCR9
199	CXCL2
200	CD40LG
201	CD44
202	CD81
203	CDC7
204	CISD1
205	CCNA2
206	CCNE1
207	CCNC
208	CDK9
209	CDK1
210	CDK2
211	CDK4
212	CDK8
213	TP53
214	UGCG
215	PRKG2
216	PDE3A
217	CCKAR

218	CCKBR
219	CMA1
220	CTRC
221	CTRB1
222	MAPK8
223	MAPK9
224	MAPK10
225	CLDN4
226	ST3GAL3
227	F7
228	F3
229	F8
230	F10
231	F11
232	F13A1
233	COL1A1
234	COL3A1
235	CFD
236	SERPINA6
237	CRHR1
238	CRP
239	CREBBP
240	CX3CR1
241	CXCR3
242	CXCL10
243	CXCL11
244	CCNT1
245	CCNB3
246	CCNE2
247	CCND3
248	CCND1
249	CDK5R1
250	CDK5
251	CDK6
252	CDK7
253	CDKN1A
254	CDKN2A
255	PTGS1
256	PTGS2
257	CYSLTR1
258	CFTR
259	LNPEP
260	UQCRB
261	CYP11B1

262	CYP11B2
263	CYP17A1
264	CYP19A1
265	CYP1A2
266	CYP1B1
267	CYP24A1
268	CYP26A1
269	CYP2C19
270	CYP2C9
271	CYP2D6
272	CYP3A4
273	CYP51A1
274	PLA2G4A
275	DRD5
276	DRD2
277	DAO
278	DCAF5
279	DAPK1
280	DAPK2
281	DAPK3
282	OPRD1
283	DCK
284	DGAT1
285	DHFR
286	DHODH
287	QDPR
288	CTSC
289	DPP7
290	DPP4
291	DPP9
292	DPP8
293	DNMT1
294	DNMT3A
295	ERCC5
296	POLA1
297	POLB
298	RAD51
299	TOP1
300	TOP2A
301	APEX1
302	MPG
303	PRKDC
304	DRD1
305	DRD3

306	DRD4
307	SLC6A3
308	DUOX2
309	DSTYK
310	MAP2K1
311	MAP2K2
312	MAP2K3
313	MAP2K4
314	CDC25A
315	CDC25B
316	CDC25C
317	CLK2
318	CLK3
319	CLK4
320	TTK
321	DUSP1
322	DUSP3
323	DYRK1B
324	CLK1
325	DYRK1A
326	DUT
327	DNM1
328	MDM2
329	EGLN1
330	ELAVL1
331	HSP90B1
332	EPAS1
333	EDNRA
334	EDNRB
335	EDN1
336	ECE1
337	EPHB4
338	EPHB6
339	EPHA1
340	EPHA2
341	EPHA3
342	EPHA4
343	EPHA5
344	EPHA6
345	EPHA7
346	EPHA8
347	EPHB1
348	EPHB2
349	EPHB3

350	EGFR
351	EPHX2
352	EPHX1
353	SLC29A1
354	SELE
355	HSD17B1
356	HSD17B2
357	HSD17B3
358	ESR1
359	ESR2
360	SULT1E1
361	ESRRA
362	ESRRB
363	ELK1
364	EIF4A1
365	EIF2AK1
366	EIF2AK3
367	EIF6
368	SLC1A3
369	XPO1
370	EED
371	EZH2
372	RBBP4
373	EZR
374	FABP4
375	FABP5
376	FABP2
377	FABP3
378	FADS1
379	FASN
380	FABP1
381	FAP
382	FGFR1
383	FGFR3
384	FKBP1A
385	FEN1
386	PTK2
387	FOSL1
388	FOSL2
389	FFAR1
390	FFAR2
391	FBP1
392	FUT4
393	PTGDR2

394	GRK6
395	GRK7
396	CCNB1
397	GABRA2
398	GABRA1
399	GABRA3
400	GABRA5
401	GABRB3
402	GABRG2
403	GABBR2
404	GABBR1
405	LGALS3
406	LGALS4
407	LGALS8
408	LGALS9
409	GABRE
410	GABRG3
411	GABRA6
412	PSEN2
413	GJA1
414	PGGT1B
415	GCGR
416	NR3C1
417	G6PD
418	GRIN2A
419	GRIN1
420	GRIA2
421	GRIK1
422	GRIK2
423	GLUL
424	QPCT
425	GSTM1
426	GSTM2
427	GSTP1
428	GLRA1
429	GLRA2
430	SLC6A9
431	PYGM
432	GSK3A
433	GSK3B
434	GLO1
435	GPBAR1
436	GPER1
437	GPR35

438	GPR55
439	GRK2
440	PLA2G10
441	HSPA8
442	HSF1
443	HSP90AB1
444	HSP90AA1
445	PTPN22
446	HPGDS
447	HMOX1
448	HPSE
449	MET
450	HNF4A
451	KCNH2
452	HK1
453	HK2
454	GCK
455	HRH1
456	HRH2
457	HRH3
458	HRH4
459	EP300
460	KAT2B
461	HDAC1
462	HDAC10
463	HDAC11
464	HDAC2
465	HDAC3
466	NCOR2
467	HDAC4
468	HDAC5
469	HDAC6
470	HDAC7
471	HDAC8
472	HDAC9
473	HMGCR
474	NKX3-1
475	HIPK4
476	LIPE
477	HAS2
478	HAO1
479	HAO2
480	HCAR2
481	HIF1A

482	IGHG1
483	SLC10A2
484	IER3IP1
485	FCER2
486	IDO1
487	MCL1
488	CHUK
489	IKBKB
490	IKBKE
491	IMPDH1
492	IMPDH2
493	INSR
494	IGFBP3
495	IGF1R
496	IGF2
497	ITGA4
498	ITGB1
499	ITGA2B
500	ITGAV
501	ITGB5
502	ITGB3
503	ITGAL
504	ICAM1
505	IFNG
506	IRF1
507	IL1A
508	IL1B
509	IRAK4
510	IL10
511	IL2
512	IL4
513	IL6
514	IL6ST
515	CXCL8
516	CXCR1
517	CXCR2
518	MMP1
519	IDH1
520	IARS
521	ICMT
522	JAK3
523	KLK1
524	KLK2
525	OPRK1

526	RET
527	KIF11
528	NUF2
529	KLF7
530	LDLR
531	LAP3
532	LRRK2
533	SELL
534	PTPRC
535	ELANE
536	LTA4H
537	LTB4R
538	LIMK1
539	LIMK2
540	LBP
541	PYGL
542	LDHA
543	LDHB
544	SLC27A1
545	SLC5A4
546	ACP1
547	NR1H3
548	NR1H2
549	CD38
550	KDM3A
551	KDM4C
552	KDM4E
553	KDM5B
554	KDM1A
555	GAA
556	PRCP
557	LYZ
558	CSF1R
559	MIF
560	MPEG1
561	MST1R
562	MGAM
563	MPI
564	MAPK3
565	MAPK1
566	MAPK14
567	MKNK2
568	MAPKAPK2
569	MKNK1

570	MELK
571	MMP10
572	MMP12
573	MMP13
574	MMP14
575	MMP15
576	MMP16
577	MMP26
578	MMP3
579	MMP7
580	MMP8
581	MMP9
582	MCHR1
583	MC4R
584	MTNR1A
585	MTNR1B
586	GRM1
587	GRM2
588	GRM3
589	GRM5
590	GRM6
591	GRM8
592	KISS1R
593	METAP1
594	METAP2
595	MARS
596	MAP2
597	MAPT
598	NR3C2
599	MAP3K11
600	MAP3K14
601	MAP3K5
602	MAP3K8
603	MAP3K9
604	MAP4K4
605	CD14
606	MGLL
607	PTPN1
608	OPRM1
609	MALT1
610	SLC47A1
611	ABCC1
612	CHRM1
613	CHRM2

614	CHRM3
615	CHRM4
616	CHRM5
617	GYS1
618	MUSK
619	MYC
620	MPO
621	MYLK
622	DDAH1
623	NLRP3
624	NAAA
625	NQO1
626	SIRT1
627	SIRT2
628	MT-ND6
629	NDUFA4
630	NOX4
631	NOX5
632	POR
633	NAE1
634	MME
635	NTRK1
636	TACR1
637	TACR2
638	NMUR2
639	CHRNA4
640	CHRNA7
641	CHRNA2
642	CHRNA3
643	CHRNB4
644	CHRNB3
645	NPY5R
646	NPBWR1
647	NTRK2
648	NCF1
649	NFKBIA
650	NAMPT
651	CHRNA5
652	NPC1L1
653	NOS3
654	NOS2
655	NOS1
656	OPRL1
657	SLC6A2

658	NTRK3
659	NUAK1
660	NFE2L2
661	NFKB1
662	RELA
663	NFATC1
664	NCOA1
665	NCOA2
666	RORA
667	RORC
668	NR1I2
669	NR1I3
670	NR4A1
671	NPM1
672	HCRTR1
673	HCRTR2
674	ODC1
675	SPP1
676	OLR1
677	OXTR
678	P2RX3
679	P2RX7
680	LGALS7
681	PIN1
682	BRPF1
683	PRF1
684	PER2
685	CASK
686	PRXC1A
687	PPARD
688	PPARG
689	PPARA
690	ABCB1
691	FARS2
692	PNMT
693	PTEN
694	PIK3CG
695	PIK3C2B
696	PIK3C2G
697	PIP5K1C
698	PIP4K2C
699	PDE10A
700	PDE11A
701	PDE2A

702	PDE3B
703	PDE4A
704	PDE4B
705	PDE4C
706	PDE4D
707	PDE5A
708	PDE7A
709	PDE8B
710	PDE9A
711	PGK1
712	PLA2G1B
713	PLA2G2A
714	PLA2G2C
715	PLA2G5
716	PLAA
717	PLCG1
718	PHKG2
719	PIK3R1
720	PIK3CA
721	PIK3CB
722	PIK3CD
723	PI4KB
724	PGF
725	RBP4
726	PLG
727	SERPINE1
728	PTAFR
729	PDGFRA
730	PDGFRB
731	PLEC
732	PARP1
733	PARP2
734	PARP4
735	PABPC1
736	PAOX
737	KCNK2
738	KCNK3
739	KCNK9
740	ATP4B
741	HERC1
742	GPR139
743	PCOLCE
744	EGF
745	PGR

746	PCNA
747	PREP
748	PTGES
749	PTGER3
750	PTGDR
751	PTGER1
752	PTGER2
753	PTGER4
754	PTGFR
755	PTGIR
756	ACPP
757	PSMB2
758	PSMB5
759	PRMT3
760	RUNX1T1
761	FNTA
762	PRKCZ
763	PRKCA
764	PRKCB
765	PRKCD
766	PRKCE
767	PRKCH
768	PRKCG
769	PRKD1
770	PRKCQ
771	PKN1
772	MDM4
773	PPM1A
774	PPM1B
775	PBRM1
776	PTK2B
777	F2R
778	TGM2
779	PTPN6
780	PTPN11
781	PTP4A3
782	FOS
783	JUN
784	WNT3
785	PIM1
786	MERTK
787	ROS1
788	VAV1
789	PPOX

790	SELP
791	PNP
792	NPEPPS
793	QRFPR
794	PDK1
795	NQO2
796	AKT1
797	RAF1
798	RASSF1
799	RASA1
800	RASGRP3
801	RASGRP1
802	ERBB2
803	ERBB3
804	PTPRF
805	PTPRS
806	RGS4
807	REN
808	RB1
809	RXRA
810	RXRB
811	ROCK2
812	ROCK1
813	RHO
814	RPS6KB1
815	RPS6KA1
816	RPS6KA2
817	RPS6KA3
818	RPS6KA4
819	RPS6KA5
820	RUNX2
821	ATP2A1
822	SCARB1
823	PPP2R5A
824	PPP2CA
825	PPP1CA
826	PPP1CC
827	AURKA
828	AURKB
829	BRAF
830	CHEK1
831	CHEK2
832	ILK
833	MTOR

834	NEK2
835	NEK6
836	PAK1
837	PAK4
838	PIM2
839	PIM3
840	PLK1
841	PLK2
842	PLK3
843	ACVRL1
844	RIPK2
845	SGK1
846	TAOK1
847	TAOK2
848	TAOK3
849	TBK1
850	WEE1
851	ERN1
852	PPP3CB
853	ATM
854	HTR1D
855	HTR1F
856	HTR2B
857	HTR3B
858	HTR3A
859	HTR4
860	HTR5A
861	HTR6
862	HTR7
863	SLC6A4
864	PON1
865	NEU3
866	SIGMAR1
867	STAT1
868	STAT3
869	MTAP
870	SMO
871	SCN5A
872	SCN9A
873	SLC5A1
874	SLC5A2
875	SLC9A1
876	SLC5A11
877	SLC28A2

878	SLC13A5
879	SLC2A4
880	SLC22A12
881	SLC22A2
882	SLC22A6
883	SSTR1
884	SSTR2
885	SSTR3
886	SSTR4
887	SSTR5
888	SHH
889	SORD
890	S1PR1
891	S1PR3
892	S1PR2
893	SQLE
894	FDFT1
895	KIT
896	SRD5A1
897	SRD5A2
898	CYP27A1
899	SREBF2
900	STS
901	PCSK7
902	ABCC9
903	SAE1
904	SOD1
905	SLC18A2
906	TNKS
907	TNKS2
908	TAS2R31
909	PTPN2
910	TERT
911	DNTT
912	SHBG
913	TGFBR1
914	F2
915	THBD
916	TBXA2R
917	TBXAS1
918	TK2
919	TYMP
920	TYMS
921	THRA

922	THRB
923	PLAT
924	TNF
925	TLR9
926	SMARCA4
927	E2F1
928	E2F2
929	TGFB1
930	HRAS
931	TRPA1
932	TRPM8
933	TRPV3
934	TRPV4
935	VCP
936	TSPO
937	TTR
938	TNNC1
939	PRSS1
940	PRSS3
941	TUBB1
942	TUBB3
943	TTL
944	TDRD7
945	DIO1
946	AGTR1
947	TYR
948	ABL1
949	BLK
950	BMX
951	PTK6
952	BTK
953	CSK
954	FGR
955	FYN
956	HCK
957	ITK
958	JAK1
959	JAK2
960	LCK
961	LYN
962	FLT3
963	TYRO3
964	AXL
965	SRC

966	SYK
967	TEK
968	TXK
969	TYK2
970	YES1
971	TDP1
972	TDP2
973	UGT2B7
974	COQ8B
975	UPP1
976	PLAUR
977	PLAU
978	UTS2R
979	TRPV1
980	VCAM1
981	VEGFA
982	FLT1
983	KDR
984	FLT4
985	AVPR1A
986	AVPR2
987	VDR
988	CACNA1C
989	CACNA1B
990	KCNA3
991	KCNA5
992	KCNE1
993	XDH
994	CCNB2
995	UBA2
996	GABRB2
997	FNTB
998	PSENEN
999	NCSTN
1000	APH1A
1001	PSEN1
1002	APH1B
1003	ITGB2
1004	CCNA1
1005	CCND2
1006	CCNH
1007	TNNT2
1008	TNNI3
1009	CHRNB2

1010	P06493
1011	P14635
1012	O95067
1013	CDK3
1014	SUZ12
1015	RBBP7
1016	KCNQ1
1017	ATP4A
1018	CHRNA1
1019	CHRNG
1020	CHRND
1021	CHRNA6
1022	GRIN2B
