# Supplementary material for: Niaoduqing alleviates podocyte injury in high glucose model via regulating multiple targets and AGE/RAGE pathway: Network pharmacology and experimental validation
Source: Front Pharmacol. 2023 Feb 27;14:1047184. doi: 10.3389/fphar.2023.1047184 (PMC10009170; doi:10.3389/fphar.2023.1047184)
Supplement: Supplementary file 12 [file Table12.pdf]

Table S12 The topological parameters of each nodes in PPI network of quercetin

	Node	Degree Value	Betweenness Centrality	Closeness Centrality
1	VEGFA	20	7.7833333	1
2	NOS3	18	6.866667	0.90909094
3	HMOX1	18	4.2833333	0.90909094
4	ICAM1	18	3.95	0.90909094
5	CXCL10	16	3.7	0.8333333
6	PTGS2	16	2.4166667	0.8333333
7	TGFB1	14	0.25	0.7692308
8	THBD	10	0.25	0.6666667
9	SPP1	14	0.25	0.7692308
10	DPP4	8	0.25	0.625
11	SOD1	8	0	0.625
