# Supplementary material for: Niaoduqing alleviates podocyte injury in high glucose model via regulating multiple targets and AGE/RAGE pathway: Network pharmacology and experimental validation
Source: Front Pharmacol. 2023 Feb 27;14:1047184. doi: 10.3389/fphar.2023.1047184 (PMC10009170; doi:10.3389/fphar.2023.1047184)
Supplement: Supplementary file 13 [file Table7.pdf]

Table S7 The targets list of podocyte injury

	GeneCards	OMIM	Combined
1	NPHS1	HLA-B	NPHS1
2	KIRREL1	TCF21	KIRREL1
3	IL6	NINJ2	IL6
4	TNF		TNF
5	NPHS2		NPHS2
6	WT1		WT1
7	PTPRO		PTPRO
8	APOE		APOE
9	SERPINE1		SERPINE1
10	CD2AP		CD2AP
11	VWF		VWF
12	CASP3		CASP3
13	CXCL8		CXCL8
14	IL1B		IL1B
15	BDNF		BDNF
16	LCN2		LCN2
17	MAPK1		MAPK1
18	NTRK1		NTRK1
19	NOS2		NOS2
20	NOS1AP		NOS1AP
21	CFH		CFH
22	F2		F2
23	PLCE1		PLCE1
24	SYNPO		SYNPO
25	MYO1E		MYO1E
26	MMP9		MMP9
27	VEGFA		VEGFA
28	HMOX1		HMOX1
29	LMX1B		LMX1B
30	PODXL		PODXL
31	TGFB1		TGFB1
32	TP53		TP53
33	IL18		IL18
34	ACTN4		ACTN4
35	MAGI2		MAGI2
36	SOD1		SOD1
37	HAVCR1		HAVCR1
38	THBD		THBD
39	TLR4		TLR4
40	DAAM2		DAAM2
41	MPO		MPO

42	S100B	S100B
43	ALB	ALB
44	ARHGDIA	ARHGDIA
45	CCL2	CCL2
46	CRP	CRP
47	ADAMTS13	ADAMTS13
48	ICAM1	ICAM1
49	AVIL	AVIL
50	SPTAN1	SPTAN1
51	ACE	ACE
52	EMP2	EMP2
53	HPRT1	HPRT1
54	CTNNB1	CTNNB1
55	STAT3	STAT3
56	NFE2L2	NFE2L2
57	INS	INS
58	HGF	HGF
59	RAC1	RAC1
60	DGKE	DGKE
61	CD46	CD46
62	AQP4	AQP4
63	TJP1	TJP1
64	GDNF	GDNF
65	TTR	TTR
66	CST3	CST3
67	TRPC6	TRPC6
68	COMT	COMT
69	UCHL1	UCHL1
70	GJA1	GJA1
71	MIR21	MIR21
72	HMGB1	HMGB1
73	EPO	EPO
74	COQ8B	COQ8B
75	IGF1	IGF1
76	EGF	EGF
77	CAT	CAT
78	HSPG2	HSPG2
79	NLRP3	NLRP3
80	SPP1	SPP1
81	EDN1	EDN1
82	CDC42	CDC42
83	PTEN	PTEN
84	EP300	EP300
85	CFI	CFI

86	NGFR	NGFR
87	WTIP	WTIP
88	F3	F3
89	KNG1	KNG1
90	IL1A	IL1A
91	NOS3	NOS3
92	ARG1	ARG1
93	PLA2R1	PLA2R1
94	FGA	FGA
95	GH1	GH1
96	MIF	MIF
97	PDPN	PDPN
98	PTGS2	PTGS2
99	HIF1A	HIF1A
100	SLC2A1	SLC2A1
101	MAPT	MAPT
102	C3	C3
103	CR1	CR1
104	SOD2	SOD2
105	PLAT	PLAT
106	PIK3C2A	PIK3C2A
107	APP	APP
108	FGF2	FGF2
109	CDK5	CDK5
110	CCND1	CCND1
111	ZAP70	ZAP70
112	AKT1	AKT1
113	MAPK8	MAPK8
114	ADIPOQ	ADIPOQ
115	SIRT1	SIRT1
116	CLIC5	CLIC5
117	EGFR	EGFR
118	HLA-DRB1	HLA-DRB1
119	FCGR2A	FCGR2A
120	DNM1	DNM1
121	NES	NES
122	KIRREL3	KIRREL3
123	FLT1	FLT1
124	BCL2	BCL2
125	MIR34A	MIR34A
126	PPARG	PPARG
127	ANLN	ANLN
128	CXCL10	CXCL10
129	REN	REN

130	IL17A	IL17A
131	PAX2	PAX2
132	RHOA	RHOA
133	BRAF	BRAF
134	CREBBP	CREBBP
135	SERPINC1	SERPINC1
136	CSPG4	CSPG4
137	APOL1	APOL1
138	ALDH5A1	ALDH5A1
139	BAX	BAX
140	MAPK14	MAPK14
141	ITGAM	ITGAM
142	AGTR1	AGTR1
143	HSPB1	HSPB1
144	CD36	CD36
145	PDGFRB	PDGFRB
146	CKB	CKB
147	JUN	JUN
148	CD55	CD55
149	DDN	DDN
150	AGER	AGER
151	HP	HP
152	YRDC	YRDC
153	FN1	FN1
154	INF2	INF2
155	NPPA	NPPA
156	TKT	TKT
157	PPP3CA	PPP3CA
158	CCR2	CCR2
159	B2M	B2M
160	CXCL12	CXCL12
161	PLG	PLG
162	VIM	VIM
163	CXCL1	CXCL1
164	ACE2	ACE2
165	TIMP1	TIMP1
166	MTOR	MTOR
167	MYLK	MYLK
168	AIF1	AIF1
169	CDH2	CDH2
170	CAV1	CAV1
171	NTRK2	NTRK2
172	BMP6	BMP6
173	SRC	SRC

174	NFKB1	NFKB1
175	TLR2	TLR2
176	VCAM1	VCAM1
177	IQGAP1	IQGAP1
178	FGB	FGB
179	COMP	COMP
180	SAA1	SAA1
181	AGT	AGT
182	CASK	CASK
183	MYC	MYC
184	GAMT	GAMT
185	RTN4	RTN4
186	WDR73	WDR73
187	BMP7	BMP7
188	MME	MME
189	NOTCH1	NOTCH1
190	ANGPTL3	ANGPTL3
191	HLA-A	HLA-A
192	TF	TF
193	CLU	CLU
194	CTLA4	CTLA4
195	ITGB1	ITGB1
196	NAGLU	NAGLU
197	CDKN1B	CDKN1B
198	IL1RN	IL1RN
199	HBB	HBB
200	NOX4	NOX4
201	MAGI2-AS3	MAGI2-AS3
202	PLAU	PLAU
203	SELE	SELE
204	KLF15	KLF15
205	ANGPT2	ANGPT2
206	NUP107	NUP107
207	LRP2	LRP2
208	HMCN1	HMCN1
209	FAS	FAS
210	FABP1	FABP1
211	FASLG	FASLG
212	HDAC4	HDAC4
213	TTC21B	TTC21B
214	PIK3CA	PIK3CA
215	CRB2	CRB2
216	TBC1D8B	TBC1D8B
217	C5	C5

218	COL5A1	COL5A1
219	CDKN1A	CDKN1A
220	NOD2	NOD2
221	ITGB3	ITGB3
222	CD14	CD14
223	HSPA1A	HSPA1A
224	MMP2	MMP2
225	SQSTM1	SQSTM1
226	CCN2	CCN2
227	TXN	TXN
228	SPTLC1	SPTLC1
229	MAPK3	MAPK3
230	CP	CP
231	FOXC2	FOXC2
232	UMOD	UMOD
233	HAVCR2	HAVCR2
234	MIR15A	MIR15A
235	ABCB1	ABCB1
236	IL13	IL13
237	GSR	GSR
238	PLAUR	PLAUR
239	ERBB4	ERBB4
240	KDR	KDR
241	ENG	ENG
242	APRT	APRT
243	ANGPT1	ANGPT1
244	GDF15	GDF15
245	FAT1	FAT1
246	DYSF	DYSF
247	ADM	ADM
248	MIR126	MIR126
249	HSPA4	HSPA4
250	ALDH3A2	ALDH3A2
251	MAFB	MAFB
252	PVALB	PVALB
253	TRIM8	TRIM8
254	LAMA2	LAMA2
255	FYN	FYN
256	SOD3	SOD3
257	SP1	SP1
258	CHI3L1	CHI3L1
259	NUP133	NUP133
260	TUBA1A	TUBA1A
261	COL4A3	COL4A3

262	APOA1	APOA1
263	PPARA	PPARA
264	TIMP2	TIMP2
265	FGG	FGG
266	MALAT1	MALAT1
267	COL4A5	COL4A5
268	PMM2	PMM2
269	WWC1	WWC1
270	AQP1	AQP1
271	CHD2	CHD2
272	MAP2K1	MAP2K1
273	CXCR4	CXCR4
274	PLEC	PLEC
275	LGALS3	LGALS3
276	YAP1	YAP1
277	PLA2G1B	PLA2G1B
278	PRKCA	PRKCA
279	SYT1	SYT1
280	SDHB	SDHB
281	TAOK1	TAOK1
282	IL2RA	IL2RA
283	ZMPSTE24	ZMPSTE24
284	MET	MET
285	CCL5	CCL5
286	GGT1	GGT1
287	GABRD	GABRD
288	KL	KL
289	ATF3	ATF3
290	SNCA	SNCA
291	CD40LG	CD40LG
292	PMP22	PMP22
293	G6PD	G6PD
294	HSPA8	HSPA8
295	CALB1	CALB1
296	CCL3	CCL3
297	CFB	CFB
298	KIF15	KIF15
299	GP6	GP6
300	FBN1	FBN1
301	COQ2	COQ2
302	SERPINA1	SERPINA1
303	RPS27A	RPS27A
304	CFHR1	CFHR1
305	PECAM1	PECAM1

306	THBS1	THBS1
307	ANXA5	ANXA5
308	PIK3CB	PIK3CB
309	CLTC	CLTC
310	SELL	SELL
311	HLA-DQA1	HLA-DQA1
312	OCRL	OCRL
313	FCGR3B	FCGR3B
314	GRN	GRN
315	IGFBP3	IGFBP3
316	PDGFB	PDGFB
317	GNB1	GNB1
318	GSK3B	GSK3B
319	KIRREL2	KIRREL2
320	PTK2	PTK2
321	CSF1	CSF1
322	PAH	PAH
323	ASXL1	ASXL1
324	WDR4	WDR4
325	CTSB	CTSB
326	C4B	C4B
327	RPL36A-HNRNPH2	RPL36A-HNRNPH2
328	KANK1	KANK1
329	VAMP2	VAMP2
330	NQO1	NQO1
331	RBP4	RBP4
332	GSTT1	GSTT1
333	ITGAV	ITGAV
334	CD44	CD44
335	PLCG1	PLCG1
336	TNFRSF1B	TNFRSF1B
337	CREB1	CREB1
338	CDK2	CDK2
339	HLA-C	HLA-C
340	EZR	EZR
341	PIK3CG	PIK3CG
342	PDGFC	PDGFC
343	IGFBP7	IGFBP7
344	GAPDH	GAPDH
345	TCF21	TCF21
346	MEG3	MEG3
347	CFTR	CFTR
348	CXCR3	CXCR3
349	MAF	MAF

350	CXCL16	CXCL16
351	CD47	CD47
352	KRAS	KRAS
353	AKR1B1	AKR1B1
354	ALDH2	ALDH2
355	FAT4	FAT4
356	HSPA5	HSPA5
357	SGPL1	SGPL1
358	TRIO	TRIO
359	LBP	LBP
360	FOXO3	FOXO3
361	TNFSF11	TNFSF11
362	HBA2	HBA2
363	CYBB	CYBB
364	CDKN1C	CDKN1C
365	MIR27A	MIR27A
366	CCNB1	CCNB1
367	HSP90AA1	HSP90AA1
368	SHH	SHH
369	GC	GC
370	SPTLC2	SPTLC2
371	TGFB2	TGFB2
372	IGF2	IGF2
373	RETN	RETN
374	FABP3	FABP3
375	GRHPR	GRHPR
376	CASP1	CASP1
377	IFI27	IFI27
378	GAL	GAL
379	STAT1	STAT1
380	ATL1	ATL1
381	ANXA1	ANXA1
382	CD59	CD59
383	CDK4	CDK4
384	MIR155	MIR155
385	SMAD3	SMAD3
386	ASH1L	ASH1L
387	LDHA	LDHA
388	TNXB	TNXB
389	NPAP1	NPAP1
390	AXDND1	AXDND1
391	PALS1	PALS1
392	PIK3R1	PIK3R1
393	CD80	CD80

394	PTGS1	PTGS1
395	CDH1	CDH1
396	DCN	DCN
397	ITGB4	ITGB4
398	S100A9	S100A9
399	CDKN3	CDKN3
400	MIR221	MIR221
401	RB1	RB1
402	ASAH1	ASAH1
403	KRT18	KRT18
404	TLR9	TLR9
405	MIR16-1	MIR16-1
406	DPP4	DPP4
407	MPZ	MPZ
408	AMBP	AMBP
409	GPX1	GPX1
410	P2RX7	P2RX7
411	MIR30A	MIR30A
412	ITSN1	ITSN1
413	GSTP1	GSTP1
414	NR3C1	NR3C1
415	NPY	NPY
416	GLA	GLA
417	CALR	CALR
418	NPR3	NPR3
419	ANPEP	ANPEP
420	FOXC1	FOXC1
421	ADAM10	ADAM10
422	MYCN	MYCN
423	LCAT	LCAT
424	HSPD1	HSPD1
425	BSG	BSG
426	ADCYAP1	ADCYAP1
427	AKR1A1	AKR1A1
428	IRAK1	IRAK1
429	ABCB11	ABCB11
430	ALDOA	ALDOA
431	ABL1	ABL1
432	FAH	FAH
433	CHKA	CHKA
434	VDR	VDR
435	DDIT3	DDIT3
436	LPL	LPL
437	MIR29B1	MIR29B1

438	DNM1L	DNM1L
439	TCN2	TCN2
440	SORD	SORD
441	BMP2	BMP2
442	COL4A4	COL4A4
443	MIR378A	MIR378A
444	PI3	PI3
445	ATP1A1	ATP1A1
446	DNASE1	DNASE1
447	PTX3	PTX3
448	JAK2	JAK2
449	SMAD4	SMAD4
450	GALC	GALC
451	CLDN5	CLDN5
452	PLCB1	PLCB1
453	HBEGF	HBEGF
454	DES	DES
455	POSTN	POSTN
456	TNFRSF11A	TNFRSF11A
457	SMPD1	SMPD1
458	NCK1	NCK1
459	SLC4A1	SLC4A1
460	DSP	DSP
461	XIST	XIST
462	SERPINF2	SERPINF2
463	TNFSF12	TNFSF12
464	SERPINA3	SERPINA3
465	PRKCB	PRKCB
466	CTSG	CTSG
467	SLC22A6	SLC22A6
468	OLR1	OLR1
469	PPARGC1A	PPARGC1A
470	COL4A2	COL4A2
471	F2R	F2R
472	ANGPTL2	ANGPTL2
473	HINT1	HINT1
474	TRAF6	TRAF6
475	MAOB	MAOB
476	MYH9	MYH9
477	MDM2	MDM2
478	ZEB2	ZEB2
479	PTPRQ	PTPRQ
480	GRB2	GRB2
481	SEMA3A	SEMA3A

482	KEAP1	KEAP1
483	DAG1	DAG1
484	ACTA1	ACTA1
485	KAT5	KAT5
486	AGRN	AGRN
487	TNFRSF11B	TNFRSF11B
488	TPP1	TPP1
489	SIRT6	SIRT6
490	PARK7	PARK7
491	PRDX6	PRDX6
492	NRAS	NRAS
493	STING1	STING1
494	GSN	GSN
495	RGMA	RGMA
496	BAD	BAD
497	CAMP	CAMP
498	AGXT	AGXT
499	H19	H19
500	ROCK1	ROCK1
501	C1S	C1S
502	SMAD2	SMAD2
503	LTF	LTF
504	RELA	RELA
505	MUC1	MUC1
506	UBE2L3	UBE2L3
507	S100A8	S100A8
508	NFATC1	NFATC1
509	C5AR1	C5AR1
510	MIR193A	MIR193A
511	MVK	MVK
512	AFP	AFP
513	HLA-G	HLA-G
514	COX5A	COX5A
515	SPOP	SPOP
516	JUP	JUP
517	CHD7	CHD7
518	APOH	APOH
519	BLVRB	BLVRB
520	MT-ATP6	MT-ATP6
521	VDAC1	VDAC1
522	MIR17	MIR17
523	PKD1	PKD1
524	SLIT2	SLIT2
525	CACNA1B	CACNA1B

526	SLC9A3	SLC9A3
527	PROCR	PROCR
528	VTN	VTN
529	TGFBR2	TGFBR2
530	MT-TL1	MT-TL1
531	CDH3	CDH3
532	PTGDS	PTGDS
533	SLPI	SLPI
534	ANGPTL4	ANGPTL4
535	E2F1	E2F1
536	TIMP3	TIMP3
537	KCNMA1	KCNMA1
538	EIF2S1	EIF2S1
539	CBS	CBS
540	S100A4	S100A4
541	SLC9A3R2	SLC9A3R2
542	FOXO1	FOXO1
543	PKD2	PKD2
544	GLUL	GLUL
545	F11	F11
546	NDRG1	NDRG1
547	CFL1	CFL1
548	CTSL	CTSL
549	AGTR2	AGTR2
550	SERPINF1	SERPINF1
551	PROC	PROC
552	TMEM47	TMEM47
553	PPBP	PPBP
554	NR3C2	NR3C2
555	EZH2	EZH2
556	SORL1	SORL1
557	NCAM1	NCAM1
558	PROM1	PROM1
559	ENO1	ENO1
560	SEMA3E	SEMA3E
561	KIAA0319L	KIAA0319L
562	RARA	RARA
563	SREBF1	SREBF1
564	ADAM17	ADAM17
565	CDH5	CDH5
566	CNP	CNP
567	CTSD	CTSD
568	NEU1	NEU1
569	TUG1	TUG1

570	RELN	RELN
571	TFF3	TFF3
572	CRABP2	CRABP2
573	MIR25	MIR25
574	ALDH9A1	ALDH9A1
575	PRKCD	PRKCD
576	PCNA	PCNA
577	MIR182	MIR182
578	EPCAM	EPCAM
579	RNASE3	RNASE3
580	MMP7	MMP7
581	NOTCH2	NOTCH2
582	EDNRA	EDNRA
583	TGFA	TGFA
584	HDAC9	HDAC9
585	GARS1	GARS1
586	IGFBP1	IGFBP1
587	RIPK3	RIPK3
588	AQP2	AQP2
589	IGFBP2	IGFBP2
590	H2BC8	H2BC8
591	DDAH1	DDAH1
592	ABCA1	ABCA1
593	FKBP1A	FKBP1A
594	CAPN1	CAPN1
595	RIPK1	RIPK1
596	RHOD	RHOD
597	LYN	LYN
598	C1QB	C1QB
599	MIR29A	MIR29A
600	CUBN	CUBN
601	TNIP1	TNIP1
602	ENPEP	ENPEP
603	TEK	TEK
604	GFER	GFER
605	EDNRB	EDNRB
606	GYPA	GYPA
607	ALDOB	ALDOB
608	PNP	PNP
609	MIR10A	MIR10A
610	COL18A1	COL18A1
611	MMP10	MMP10
612	GRM5	GRM5
613	CD34	CD34

614	SOX2-OT	SOX2-OT
615	ACTB	ACTB
616	ACTR2	ACTR2
617	XBP1	XBP1
618	VEGFC	VEGFC
619	ESR2	ESR2
620	PON1	PON1
621	ITGB8	ITGB8
622	IL6ST	IL6ST
623	NRP1	NRP1
624	FTH1	FTH1
625	POLG	POLG
626	TGFBR1	TGFBR1
627	CSTB	CSTB
628	PROS1	PROS1
629	COL17A1	COL17A1
630	IRS1	IRS1
631	CXCR1	CXCR1
632	RPL5	RPL5
633	FGFBP1	FGFBP1
634	NUP93	NUP93
635	CYP4A11	CYP4A11
636	MYO1D	MYO1D
637	AOC1	AOC1
638	KLF6	KLF6
639	CDKN2A	CDKN2A
640	SMARCAL1	SMARCAL1
641	MASP2	MASP2
642	GALK1	GALK1
643	CD63	CD63
644	CMIP	CMIP
645	GALNS	GALNS
646	HADHB	HADHB
647	SCARB2	SCARB2
648	FLNB	FLNB
649	GRM1	GRM1
650	ADD1	ADD1
651	COL12A1	COL12A1
652	CD40	CD40
653	NUF2	NUF2
654	CEBPA	CEBPA
655	NFASC	NFASC
656	CCN1	CCN1
657	ACVR2A	ACVR2A

658	FLNA	FLNA
659	MST1	MST1
660	BCL2L11	BCL2L11
661	ITGA3	ITGA3
662	NR1H4	NR1H4
663	PFN2	PFN2
664	PGF	PGF
665	HDAC6	HDAC6
666	FCGR3A	FCGR3A
667	SLC9A1	SLC9A1
668	ANXA2	ANXA2
669	ZFP36	ZFP36
670	NR1H2	NR1H2
671	BMP4	BMP4
672	LRG1	LRG1
673	RET	RET
674	FZD4	FZD4
675	ITGB6	ITGB6
676	CRYAB	CRYAB
677	ACTN3	ACTN3
678	SPINK1	SPINK1
679	ELAVL1	ELAVL1
680	NT5E	NT5E
681	KRT10	KRT10
682	TNFRSF12A	TNFRSF12A
683	SERPINA7	SERPINA7
684	FTL	FTL
685	ILK	ILK
686	PRNP	PRNP
687	COL8A1	COL8A1
688	ACTC1	ACTC1
689	UBA5	UBA5
690	STIM1	STIM1
691	TAGLN	TAGLN
692	CS	CS
693	LAMA5	LAMA5
694	CLTRN	CLTRN
695	APOD	APOD
696	PRDX5	PRDX5
697	FGFR2	FGFR2
698	MYOC	MYOC
699	EPHX2	EPHX2
700	CD24	CD24
701	PTGER4	PTGER4

702	KRT16	KRT16
703	LAMB1	LAMB1
704	MIR423	MIR423
705	MERTK	MERTK
706	UACA	UACA
707	CYLD	CYLD
708	ACTA2	ACTA2
709	CD209	CD209
710	HRAS	HRAS
711	GAS6	GAS6
712	NOD1	NOD1
713	ATF6	ATF6
714	VASP	VASP
715	ACP1	ACP1
716	ACTR3	ACTR3
717	TNFSF10	TNFSF10
718	IFNB1	IFNB1
719	MGAM	MGAM
720	TGFBI	TGFBI
721	CASR	CASR
722	FERMT2	FERMT2
723	CPOX	CPOX
724	PTPN3	PTPN3
725	ABAT	ABAT
726	ADD3	ADD3
727	HDAC1	HDAC1
728	MC1R	MC1R
729	MPDU1	MPDU1
730	DEGS1	DEGS1
731	DLC1	DLC1
732	GLRX	GLRX
733	FLT4	FLT4
734	PLD3	PLD3
735	SERPINI1	SERPINI1
736	SOCS1	SOCS1
737	RAB7A	RAB7A
738	BLOC1S1	BLOC1S1
739	ARHGAP24	ARHGAP24
740	TOLLIP	TOLLIP
741	TYRO3	TYRO3
742	PVT1	PVT1
743	PAK1	PAK1
744	AKR1D1	AKR1D1
745	MIR17HG	MIR17HG

746	MYOM2	MYOM2
747	TPMT	TPMT
748	MAP2K4	MAP2K4
749	COLEC12	COLEC12
750	F2RL1	F2RL1
751	ETFA	ETFA
752	ACP5	ACP5
753	TOR1A	TOR1A
754	PPIA	PPIA
755	CTTN	CTTN
756	EEF2	EEF2
757	CRK	CRK
758	NID1	NID1
759	PRSS23	PRSS23
760	ST6GAL1	ST6GAL1
761	FGF1	FGF1
762	UBC	UBC
763	NPC2	NPC2
764	CBL	CBL
765	VCP	VCP
766	MIR30D	MIR30D
767	KNL1	KNL1
768	SLCO2A1	SLCO2A1
769	ITGB5	ITGB5
770	VCL	VCL
771	PSAP	PSAP
772	TSHB	TSHB
773	KLF4	KLF4
774	WNT1	WNT1
775	GNAS	GNAS
776	NDUFA13	NDUFA13
777	SNHG4	SNHG4
778	CCNA2	CCNA2
779	BHMT	BHMT
780	SERPINA4	SERPINA4
781	TXNIP	TXNIP
782	CLEC3B	CLEC3B
783	GPX4	GPX4
784	IL18BP	IL18BP
785	KIT	KIT
786	PICK1	PICK1
787	GOT2	GOT2
788	H2BC6	H2BC6
789	TGFBR3	TGFBR3

790	ARF6	ARF6
791	LGALS1	LGALS1
792	MIR217	MIR217
793	VPS4B	VPS4B
794	HBA1	HBA1
795	LAGE3	LAGE3
796	HDAC3	HDAC3
797	VNN1	VNN1
798	KLK1	KLK1
799	SHBG	SHBG
800	IFNGR1	IFNGR1
801	PDE5A	PDE5A
802	GPNMB	GPNMB
803	MIR26A1	MIR26A1
804	FGFR1	FGFR1
805	ANKFY1	ANKFY1
806	DDR1	DDR1
807	C1QA	C1QA
808	RCAN1	RCAN1
809	RRAS	RRAS
810	TREH	TREH
811	TPPP3	TPPP3
812	TGIF1	TGIF1
813	COL14A1	COL14A1
814	STUB1	STUB1
815	VHL	VHL
816	SHC1	SHC1
817	ICOSLG	ICOSLG
818	SIRPA	SIRPA
819	SERPINB1	SERPINB1
820	UFSP2	UFSP2
821	GUSB	GUSB
822	SPHK1	SPHK1
823	ACACA	ACACA
824	ARSA	ARSA
825	EPAS1	EPAS1
826	HOTAIR	HOTAIR
827	PTPA	PTPA
828	RPL15	RPL15
829	RPS2	RPS2
830	PHPT1	PHPT1
831	ACP3	ACP3
832	AGO2	AGO2
833	SDC4	SDC4

834	CRKL	CRKL
835	SMAD7	SMAD7
836	TOMM40	TOMM40
837	BTD	BTD
838	BGN	BGN
839	ALDH1A1	ALDH1A1
840	SESN2	SESN2
841	HPX	HPX
842	MIR874	MIR874
843	MRAS	MRAS
844	NDUFB9	NDUFB9
845	NDUFB10	NDUFB10
846	CDK5RAP2	CDK5RAP2
847	NDUFB3	NDUFB3
848	IGHG1	IGHG1
849	GAREM2	GAREM2
850	SLC44A4	SLC44A4
851	THY1	THY1
852	KRT1	KRT1
853	GREM1	GREM1
854	CAPN2	CAPN2
855	SLC12A1	SLC12A1
856	LAMA1	LAMA1
857	UGT2B7	UGT2B7
858	DDAH2	DDAH2
859	NEBL	NEBL
860	MMP14	MMP14
861	CRYAA	CRYAA
862	MIR9-1	MIR9-1
863	TLR7	TLR7
864	SLC6A19	SLC6A19
865	APOC3	APOC3
866	TPI1	TPI1
867	BPI	BPI
868	TRPC1	TRPC1
869	PTAFR	PTAFR
870	NCF2	NCF2
871	ALDH1A2	ALDH1A2
872	SLK	SLK
873	NEDD4L	NEDD4L
874	TRPC3	TRPC3
875	DKC1	DKC1
876	MIR200C	MIR200C
877	NFIB	NFIB

878	S100A1	S100A1
879	PRKAA2	PRKAA2
880	TCF3	TCF3
881	MIR135B	MIR135B
882	IDH1	IDH1
883	GLO1	GLO1
884	PTHLH	PTHLH
885	SLC6A6	SLC6A6
886	ARRB2	ARRB2
887	ITIH4	ITIH4
888	MIR150	MIR150
889	TNFRSF10B	TNFRSF10B
890	CCT5	CCT5
891	PFN1	PFN1
892	IGF1R	IGF1R
893	PTGER2	PTGER2
894	ADGRG1	ADGRG1
895	LUM	LUM
896	CLDN1	CLDN1
897	TMPRSS2	TMPRSS2
898	HTRA2	HTRA2
899	LYZ	LYZ
900	DDC	DDC
901	PGK1	PGK1
902	GSTA1	GSTA1
903	CPQ	CPQ
904	JAK1	JAK1
905	WTAP	WTAP
906	ACTG1	ACTG1
907	SPTBN1	SPTBN1
908	MIR192	MIR192
909	C3AR1	C3AR1
910	APOC2	APOC2
911	F2RL2	F2RL2
912	MIR135A1	MIR135A1
913	XPC	XPC
914	MLC1	MLC1
915	ITGA5	ITGA5
916	RRM2B	RRM2B
917	ALDH16A1	ALDH16A1
918	PTPN6	PTPN6
919	GABRB1	GABRB1
920	P2RY2	P2RY2
921	SRGAP2	SRGAP2

922	A2M	A2M
923	RAP1A	RAP1A
924	SLC22A2	SLC22A2
925	SH3KBP1	SH3KBP1
926	ALPL	ALPL
927	TLN1	TLN1
928	DOCK4	DOCK4
929	CETP	CETP
930	BID	BID
931	HLA-H	HLA-H
932	IL1RL1	IL1RL1
933	RHOC	RHOC
934	LCK	LCK
935	TUBA1B	TUBA1B
936	CAMK4	CAMK4
937	ITLN1	ITLN1
938	AIM2	AIM2
939	SLC44A2	SLC44A2
940	FHL2	FHL2
941	TRAP1	TRAP1
942	MAP2K3	MAP2K3
943	CD5L	CD5L
944	MIOX	MIOX
945	RPS6KB1	RPS6KB1
946	TFF2	TFF2
947	NUPR1	NUPR1
948	MIR10B	MIR10B
949	UPK3A	UPK3A
950	ICAM2	ICAM2
951	COX4I1	COX4I1
952	PTPRS	PTPRS
953	SLC12A3	SLC12A3
954	UFM1	UFM1
955	CDH13	CDH13
956	CEACAM5	CEACAM5
957	ATP5F1A	ATP5F1A
958	CALML4	CALML4
959	GPX3	GPX3
960	CACNA2D1	CACNA2D1
961	LCN1	LCN1
962	ALDH7A1	ALDH7A1
963	PNPO	PNPO
964	NFU1	NFU1
965	YY1	YY1

966	FOLR1	FOLR1
967	ATP6AP2	ATP6AP2
968	DSG1	DSG1
969	EEF1A1	EEF1A1
970	CANT1	CANT1
971	MFGE8	MFGE8
972	CD33	CD33
973	GPX2	GPX2
974	ORM1	ORM1
975	TFG	TFG
976	KLF2	KLF2
977	HSP90B1	HSP90B1
978	LAMC1	LAMC1
979	VASN	VASN
980	RARG	RARG
981	UBB	UBB
982	CD27	CD27
983	OPTN	OPTN
984	ST14	ST14
985	PRDX3	PRDX3
986	PRDX1	PRDX1
987	RPS19	RPS19
988	AHCY	AHCY
989	LAMB2	LAMB2
990	NID2	NID2
991	ALG10B	ALG10B
992	TMEM63C	TMEM63C
993	VSIG4	VSIG4
994	AKT2	AKT2
995	GBA	GBA
996	DDR2	DDR2
997	TMCO1	TMCO1
998	HMMR	HMMR
999	ARSB	ARSB
1000	HDAC2	HDAC2
1001	GDA	GDA
1002	FCN2	FCN2
1003	POLG2	POLG2
1004	BMPR2	BMPR2
1005	RAB6A	RAB6A
1006	PIGR	PIGR
1007	SPINT1	SPINT1
1008	SERPINH1	SERPINH1
1009	DCDC2	DCDC2

1010	LTBP4	LTBP4
1011	SORT1	SORT1
1012	ANG	ANG
1013	TMX2	TMX2
1014	MIR128-1	MIR128-1
1015	SPARCL1	SPARCL1
1016	GAA	GAA
1017	LAP3	LAP3
1018	COL6A3	COL6A3
1019	CNR2	CNR2
1020	CCN3	CCN3
1021	FBLN5	FBLN5
1022	KLF5	KLF5
1023	WAS	WAS
1024	ACTN2	ACTN2
1025	MEST	MEST
1026	CANX	CANX
1027	SLC25A3	SLC25A3
1028	DST	DST
1029	SLC5A1	SLC5A1
1030	CD151	CD151
1031	CREG1	CREG1
1032	PRSS8	PRSS8
1033	COQ8A	COQ8A
1034	APOM	APOM
1035	F11R	F11R
1036	AXL	AXL
1037	MKI67	MKI67
1038	MDH2	MDH2
1039	GSS	GSS
1040	UGT1A6	UGT1A6
1041	ATRN	ATRN
1042	NRP2	NRP2
1043	TMEM106B	TMEM106B
1044	POC1B	POC1B
1045	LIMS1	LIMS1
1046	LRRC7	LRRC7
1047	PALLD	PALLD
1048	HIP1	HIP1
1049	CHRDL1	CHRDL1
1050	KDM6A	KDM6A
1051	CALCRL	CALCRL
1052	ADD2	ADD2
1053	MYH2	MYH2

1054	GNAQ	GNAQ
1055	PSMB8	PSMB8
1056	DYNLL1	DYNLL1
1057	ALAD	ALAD
1058	CERS3	CERS3
1059	GNB2	GNB2
1060	PTGER1	PTGER1
1061	DEFB1	DEFB1
1062	ATP1A2	ATP1A2
1063	NUP205	NUP205
1064	DPP3	DPP3
1065	NEDD4	NEDD4
1066	DLX6-AS1	DLX6-AS1
1067	FYB1	FYB1
1068	IL10RB	IL10RB
1069	MIR138-1	MIR138-1
1070	CLDN7	CLDN7
1071	PDIA3	PDIA3
1072	CLMP	CLMP
1073	SKP2	SKP2
1074	HOGA1	HOGA1
1075	CENPF	CENPF
1076	BCL2L2	BCL2L2
1077	AADAC	AADAC
1078	ITGA2	ITGA2
1079	CDK5R1	CDK5R1
1080	FOXA1	FOXA1
1081	PTH1R	PTH1R
1082	MIR186	MIR186
1083	RYR2	RYR2
1084	SMPD2	SMPD2
1085	GOT1	GOT1
1086	SERPINA6	SERPINA6
1087	HSPE1	HSPE1
1088	HSD3B2	HSD3B2
1089	MTM1	MTM1
1090	NPPC	NPPC
1091	KMO	KMO
1092	PKM	PKM
1093	PRKCH	PRKCH
1094	JAG1	JAG1
1095	RPS6KA6	RPS6KA6
1096	LYRM4	LYRM4
1097	FGGY	FGGY

1098	PDGFD	PDGFD
1099	APOC1	APOC1
1100	GATM	GATM
1101	COL6A1	COL6A1
1102	VIL1	VIL1
1103	FGL2	FGL2
1104	MFAP4	MFAP4
1105	CCNE1	CCNE1
1106	PARVA	PARVA
1107	PHB1	PHB1
1108	H4C1	H4C1
1109	FBLN2	FBLN2
1110	MSRB3	MSRB3
1111	MYO1C	MYO1C
1112	MIR128-2	MIR128-2
1113	S100A13	S100A13
1114	GINS1	GINS1
1115	PXN	PXN
1116	KRIT1	KRIT1
1117	DBN1	DBN1
1118	CAPN5	CAPN5
1119	SRSF1	SRSF1
1120	DPT	DPT
1121	CBR1	CBR1
1122	CD74	CD74
1123	FABP5	FABP5
1124	CFD	CFD
1125	PHGDH	PHGDH
1126	MTHFD1	MTHFD1
1127	PEBP1	PEBP1
1128	FOSL2	FOSL2
1129	FIS1	FIS1
1130	PARD3B	PARD3B
1131	H2BC12	H2BC12
1132	S100A7	S100A7
1133	H1-5	H1-5
1134	DNAJB4	DNAJB4
1135	PODN	PODN
1136	OSGEP	OSGEP
1137	SERPIND1	SERPIND1
1138	RAN	RAN
1139	CST5	CST5
1140	BUB1B	BUB1B
1141	BUB1	BUB1

1142	CRABP1	CRABP1
1143	TSEN15	TSEN15
1144	LY6E	LY6E
1145	UPB1	UPB1
1146	QDPR	QDPR
1147	H4-16	H4-16
1148	KDSR	KDSR
1149	PRKCI	PRKCI
1150	RAB3A	RAB3A
1151	CTSH	CTSH
1152	ACVR1B	ACVR1B
1153	FGF9	FGF9
1154	S100A11	S100A11
1155	S100A6	S100A6
1156	TGM1	TGM1
1157	IDUA	IDUA
1158	LRP1	LRP1
1159	RAB11B	RAB11B
1160	PDLIM5	PDLIM5
1161	PVR	PVR
1162	CALM2	CALM2
1163	ROBO4	ROBO4
1164	COL15A1	COL15A1
1165	NAXE	NAXE
1166	CPE	CPE
1167	SMS	SMS
1168	KRT2	KRT2
1169	CLDN2	CLDN2
1170	EIF6	EIF6
1171	RPL23	RPL23
1172	GPR180	GPR180
1173	DICER1	DICER1
1174	CA4	CA4
1175	HRG	HRG
1176	ECM1	ECM1
1177	PATJ	PATJ
1178	ITGA1	ITGA1
1179	CHMP4B	CHMP4B
1180	ACMSD	ACMSD
1181	TINAGL1	TINAGL1
1182	CASC2	CASC2
1183	SMPDL3B	SMPDL3B
1184	HGD	HGD
1185	ATP6V1B2	ATP6V1B2

1186	UBA1	UBA1
1187	GSDMD	GSDMD
1188	AQP5	AQP5
1189	C1QC	C1QC
1190	GPRC5A	GPRC5A
1191	PKP1	PKP1
1192	TUBB2B	TUBB2B
1193	CLCF1	CLCF1
1194	TIAL1	TIAL1
1195	NLRP4	NLRP4
1196	INPPL1	INPPL1
1197	ATOH8	ATOH8
1198	SPON2	SPON2
1199	FBLN1	FBLN1
1200	ALDH6A1	ALDH6A1
1201	PAPPA2	PAPPA2
1202	TERF2IP	TERF2IP
1203	NOTCH4	NOTCH4
1204	GANAB	GANAB
1205	CD81	CD81
1206	CCR1	CCR1
1207	FURIN	FURIN
1208	ATP2B1	ATP2B1
1209	CELF4	CELF4
1210	PPP3CC	PPP3CC
1211	SERPINA5	SERPINA5
1212	LILRB4	LILRB4
1213	NEDD8	NEDD8
1214	S100P	S100P
1215	PHLPP1	PHLPP1
1216	KIF12	KIF12
1217	GIPR	GIPR
1218	MIR135A2	MIR135A2
1219	CCR7	CCR7
1220	TP53RK	TP53RK
1221	GNA11	GNA11
1222	NCSTN	NCSTN
1223	CIB1	CIB1
1224	RHOF	RHOF
1225	RAP1GAP	RAP1GAP
1226	DAAM1	DAAM1
1227	VEGFB	VEGFB
1228	DSG3	DSG3
1229	PCK1	PCK1

1230	SHMT1	SHMT1
1231	COTL1	COTL1
1232	TOM1L2	TOM1L2
1233	COPS8	COPS8
1234	RARS1	RARS1
1235	MARCKS	MARCKS
1236	BLZF1	BLZF1
1237	SEMA3C	SEMA3C
1238	SERPINB4	SERPINB4
1239	PTPRU	PTPRU
1240	MEP1A	MEP1A
1241	SLC38A1	SLC38A1
1242	C1QTNF1	C1QTNF1
1243	PGD	PGD
1244	MAP4	MAP4
1245	QPRT	QPRT
1246	EFEMP2	EFEMP2
1247	TRIM32	TRIM32
1248	AHSG	AHSG
1249	IRS2	IRS2
1250	CPS1	CPS1
1251	PAM	PAM
1252	DPEP1	DPEP1
1253	AQP9	AQP9
1254	FH	FH
1255	ACY1	ACY1
1256	CD28	CD28
1257	ASPA	ASPA
1258	NASP	NASP
1259	TMEM174	TMEM174
1260	SELENOW	SELENOW
1261	KDM6B	KDM6B
1262	GUK1	GUK1
1263	AKR7A3	AKR7A3
1264	ENPP6	ENPP6
1265	SLC5A10	SLC5A10
1266	SPTBN5	SPTBN5
1267	HINT3	HINT3
1268	ISOC1	ISOC1
1269	TMED7	TMED7
1270	DAB2	DAB2
1271	YES1	YES1
1272	ENPP1	ENPP1
1273	ATP6V1A	ATP6V1A

1274	LRRC55	LRRC55
1275	TRAPPC10	TRAPPC10
1276	HSPA9	HSPA9
1277	ST3GAL4	ST3GAL4
1278	CASP14	CASP14
1279	CST6	CST6
1280	PRSS1	PRSS1
1281	STC1	STC1
1282	F12	F12
1283	AKR1C4	AKR1C4
1284	HELLS	HELLS
1285	RPL18	RPL18
1286	UBE2H	UBE2H
1287	NCAPG	NCAPG
1288	VTCN1	VTCN1
1289	TMTC1	TMTC1
1290	PCLAF	PCLAF
1291	PALS2	PALS2
1292	PRKACA	PRKACA
1293	GPD2	GPD2
1294	SOSTDC1	SOSTDC1
1295	PSMC6	PSMC6
1296	GOLM1	GOLM1
1297	SEMA3B	SEMA3B
1298	TGM3	TGM3
1299	HYAL1	HYAL1
1300	REST	REST
1301	LAIR1	LAIR1
1302	WNT9A	WNT9A
1303	SERPING1	SERPING1
1304	EGFL6	EGFL6
1305	PHB2	PHB2
1306	SNX12	SNX12
1307	TPRKB	TPRKB
1308	FHIT	FHIT
1309	UGCG	UGCG
1310	ZYX	ZYX
1311	ACTG2	ACTG2
1312	SLC2A4	SLC2A4
1313	SUCLG1	SUCLG1
1314	BST1	BST1
1315	AP1S1	AP1S1
1316	PCDH11X	PCDH11X
1317	H3C1	H3C1

1318	H4C2	H4C2
1319	H4C11	H4C11
1320	H4C3	H4C3
1321	H4C8	H4C8
1322	H4C9	H4C9
1323	H4C12	H4C12
1324	H4C14	H4C14
1325	H4C5	H4C5
1326	H4C13	H4C13
1327	H4C4	H4C4
1328	H4C6	H4C6
1329	H4C15	H4C15
1330	PRKAB1	PRKAB1
1331	MIR200A	MIR200A
1332	FLT3LG	FLT3LG
1333	CCL21	CCL21
1334	DNAJC3	DNAJC3
1335	PLPBP	PLPBP
1336	ANXA6	ANXA6
1337	MTAP	MTAP
1338	CNTN1	CNTN1
1339	UQCRC2	UQCRC2
1340	HDAC11	HDAC11
1341	CRNN	CRNN
1342	GAS1	GAS1
1343	UFC1	UFC1
1344	RTN3	RTN3
1345	PDLIM1	PDLIM1
1346	SPAG5-AS1	SPAG5-AS1
1347	PTGFR	PTGFR
1348	SMAD1	SMAD1
1349	EGR2	EGR2
1350	CA1	CA1
1351	PSAT1	PSAT1
1352	APCS	APCS
1353	CDSN	CDSN
1354	SRSF2	SRSF2
1355	A2ML1	A2ML1
1356	VANGL2	VANGL2
1357	LINC01619	LINC01619
1358	PPIB	PPIB
1359	LDHB	LDHB
1360	APOA4	APOA4
1361	PCMT1	PCMT1

1362	CLIC4	CLIC4
1363	OSCAR	OSCAR
1364	LOXL2	LOXL2
1365	MAD2L2	MAD2L2
1366	EDA2R	EDA2R
1367	SNAI1	SNAI1
1368	MYL2	MYL2
1369	HTRA1	HTRA1
1370	RHPN1	RHPN1
1371	MIR29C	MIR29C
1372	PLSCR1	PLSCR1
1373	FGL1	FGL1
1374	ARRB1	ARRB1
1375	STK24	STK24
1376	HSPA14	HSPA14
1377	EIF4A1	EIF4A1
1378	KHK	KHK
1379	GSTM2	GSTM2
1380	CAB39	CAB39
1381	EXTL2	EXTL2
1382	RAB22A	RAB22A
1383	PTPN14	PTPN14
1384	ACER3	ACER3
1385	EFNB1	EFNB1
1386	EFNA1	EFNA1
1387	LGALS8	LGALS8
1388	PPIC	PPIC
1389	MIR30C1	MIR30C1
1390	MXRA8	MXRA8
1391	LYVE1	LYVE1
1392	ACADM	ACADM
1393	LAMP1	LAMP1
1394	PCSK1N	PCSK1N
1395	ACVR1	ACVR1
1396	PCDH12	PCDH12
1397	GNPTG	GNPTG
1398	HPN	HPN
1399	RAP1B	RAP1B
1400	COCH	COCH
1401	CTSV	CTSV
1402	DAD1	DAD1
1403	NACA	NACA
1404	SUSD2	SUSD2
1405	AMY1A	AMY1A

1406	NPTX1	NPTX1
1407	RARRES1	RARRES1
1408	PLXNA1	PLXNA1
1409	THRA	THRA
1410	NSF	NSF
1411	CADM1	CADM1
1412	HNRNPD	HNRNPD
1413	EPHB4	EPHB4
1414	GAK	GAK
1415	TST	TST
1416	RPL11	RPL11
1417	PEPD	PEPD
1418	SKP1	SKP1
1419	GLB1	GLB1
1420	HPGD	HPGD
1421	CLDN3	CLDN3
1422	USP14	USP14
1423	GM2A	GM2A
1424	NUP85	NUP85
1425	MIP	MIP
1426	APOA2	APOA2
1427	NLRC5	NLRC5
1428	IGF2R	IGF2R
1429	FGF4	FGF4
1430	SDCBP	SDCBP
1431	ACSL4	ACSL4
1432	IFITM3	IFITM3
1433	ERP29	ERP29
1434	REG1A	REG1A
1435	ATP7A	ATP7A
1436	SLC29A4	SLC29A4
1437	CD7	CD7
1438	DSC1	DSC1
1439	VTI1B	VTI1B
1440	STOM	STOM
1441	PTPRF	PTPRF
1442	SCP2	SCP2
1443	SFRP1	SFRP1
1444	NUP37	NUP37
1445	MIR769	MIR769
1446	KPNA2	KPNA2
1447	MAN2B1	MAN2B1
1448	EFEMP1	EFEMP1
1449	PDCD6IP	PDCD6IP

1450	MIR34C	MIR34C
1451	EEF1G	EEF1G
1452	ACADSB	ACADSB
1453	RPS13	RPS13
1454	HLA-DMA	HLA-DMA
1455	CPZ	CPZ
1456	LOC105374325	LOC105374325
1457	PLVAP	PLVAP
1458	CD9	CD9
1459	ECH1	ECH1
1460	CTSC	CTSC
1461	RPL7A	RPL7A
1462	GAPVD1	GAPVD1
1463	CSRP1	CSRP1
1464	ANXA7	ANXA7
1465	ACTN1	ACTN1
1466	RTN4RL2	RTN4RL2
1467	ATP5F1B	ATP5F1B
1468	C9	C9
1469	LAMP2	LAMP2
1470	SPINK5	SPINK5
1471	ANXA3	ANXA3
1472	PPP1CA	PPP1CA
1473	UGT1A9	UGT1A9
1474	MYDGF	MYDGF
1475	ASS1	ASS1
1476	S100A10	S100A10
1477	SNX3	SNX3
1478	ALDH1L1	ALDH1L1
1479	NDRG2	NDRG2
1480	UPK1B	UPK1B
1481	BMPR1B	BMPR1B
1482	SYVN1	SYVN1
1483	SPAG5	SPAG5
1484	PFKM	PFKM
1485	NME1	NME1
1486	STX4	STX4
1487	SIRPB1	SIRPB1
1488	RAPGEF2	RAPGEF2
1489	CTDSP1	CTDSP1
1490	DPYS	DPYS
1491	NEGR1	NEGR1
1492	RNASE7	RNASE7
1493	PARD3	PARD3

1494	MIR500A	MIR500A
1495	ETFB	ETFB
1496	GPC3	GPC3
1497	ATP6V0A1	ATP6V0A1
1498	LGALS3BP	LGALS3BP
1499	RPLP0	RPLP0
1500	MTMR3	MTMR3
1501	PLD4	PLD4
1502	DGKK	DGKK
1503	PLD5	PLD5
1504	HORMAD2	HORMAD2
1505	MBOAT2	MBOAT2
1506	GREM2	GREM2
1507	TRPC5	TRPC5
1508	YWHAE	YWHAE
1509	CHMP5	CHMP5
1510	ANKRD1	ANKRD1
1511	SV2B	SV2B
1512	B4GALT1	B4GALT1
1513	GIPC1	GIPC1
1514	SLURP1	SLURP1
1515	IGKC	IGKC
1516	CTNND1	CTNND1
1517	CYB5A	CYB5A
1518	GNS	GNS
1519	TFAM	TFAM
1520	BLVRA	BLVRA
1521	KRT9	KRT9
1522	LIPA	LIPA
1523	UBE2N	UBE2N
1524	PRSS3	PRSS3
1525	TSG101	TSG101
1526	KRT86	KRT86
1527	CISD1	CISD1
1528	EVPL	EVPL
1529	SCIN	SCIN
1530	GPRC5B	GPRC5B
1531	SRPK1	SRPK1
1532	USF1	USF1
1533	GPI	GPI
1534	HPD	HPD
1535	ITM2B	ITM2B
1536	PPIL1	PPIL1
1537	PDCD10	PDCD10

1538	SPG11	SPG11
1539	ACOT13	ACOT13
1540	CKMT1A	CKMT1A
1541	UPK2	UPK2
1542	MIR615	MIR615
1543	FADD	FADD
1544	MIR383	MIR383
1545	FLOT2	FLOT2
1546	CHMP2A	CHMP2A
1547	KAT2B	KAT2B
1548	MLXIPL	MLXIPL
1549	GCKR	GCKR
1550	NUP160	NUP160
1551	GGH	GGH
1552	PTPRJ	PTPRJ
1553	TALDO1	TALDO1
1554	TACSTD2	TACSTD2
1555	GSTO1	GSTO1
1556	RNASET2	RNASET2
1557	RPS14	RPS14
1558	BANF1	BANF1
1559	RPL22	RPL22
1560	RPL7	RPL7
1561	RPS26	RPS26
1562	CD276	CD276
1563	FLOT1	FLOT1
1564	MCF2	MCF2
1565	H3C4	H3C4
1566	MDH1	MDH1
1567	AZGP1	AZGP1
1568	AKR1B10	AKR1B10
1569	PLA2G15	PLA2G15
1570	RHEB	RHEB
1571	TAC3	TAC3
1572	ARHGAP1	ARHGAP1
1573	B3GAT3	B3GAT3
1574	DNAJC5	DNAJC5
1575	PSMD14	PSMD14
1576	AGRP	AGRP
1577	PRPS2	PRPS2
1578	ARF4	ARF4
1579	ARL1	ARL1
1580	CAPZB	CAPZB
1581	HNRNPDL	HNRNPDL

1582	INPP5A	INPP5A
1583	SH3GL3	SH3GL3
1584	KIF3A	KIF3A
1585	FXR2	FXR2
1586	ARF3	ARF3
1587	ENOPH1	ENOPH1
1588	FCRL4	FCRL4
1589	SLC25A25	SLC25A25
1590	NAA50	NAA50
1591	H1-4	H1-4
1592	IFT20	IFT20
1593	DNAJC14	DNAJC14
1594	MVB12A	MVB12A
1595	CYS1	CYS1
1596	KPRP	KPRP
1597	ARHGEF7	ARHGEF7
1598	HEXA	HEXA
1599	EIF5A	EIF5A
1600	ESD	ESD
1601	PTP4A2	PTP4A2
1602	SLC9A2	SLC9A2
1603	DDT	DDT
1604	CKMT1B	CKMT1B
1605	CDC42SE2	CDC42SE2
1606	LATS1	LATS1
1607	PLCD1	PLCD1
1608	AGA	AGA
1609	STXBP2	STXBP2
1610	CSTA	CSTA
1611	IGLL1	IGLL1
1612	ARPC1B	ARPC1B
1613	SLC39A4	SLC39A4
1614	XPNPEP3	XPNPEP3
1615	POGLUT1	POGLUT1
1616	TKFC	TKFC
1617	IGHM	IGHM
1618	VPS37D	VPS37D
1619	IQGAP2	IQGAP2
1620	F2RL3	F2RL3
1621	EXT1	EXT1
1622	PSMA5	PSMA5
1623	PRG2	PRG2
1624	SDC3	SDC3
1625	CYB5R3	CYB5R3

1626	PDHB	PDHB
1627	ERLIN2	ERLIN2
1628	CYGB	CYGB
1629	CXADR	CXADR
1630	RACK1	RACK1
1631	DPYSL5	DPYSL5
1632	ADPRH	ADPRH
1633	B4GALNT1	B4GALNT1
1634	GON7	GON7
1635	ATXN2	ATXN2
1636	HEXB	HEXB
1637	ATP1B1	ATP1B1
1638	PPP2CB	PPP2CB
1639	ATP6V1E1	ATP6V1E1
1640	GPD1	GPD1
1641	PSMA2	PSMA2
1642	HNRNPC	HNRNPC
1643	SERPINB3	SERPINB3
1644	TMPRSS11D	TMPRSS11D
1645	CAPS	CAPS
1646	DSTN	DSTN
1647	MTPN	MTPN
1648	ADIPOR2	ADIPOR2
1649	FFAR2	FFAR2
1650	FOSL1	FOSL1
1651	ARNT	ARNT
1652	SMPD3	SMPD3
1653	NPNT	NPNT
1654	DLST	DLST
1655	ECHS1	ECHS1
1656	GK	GK
1657	HLA-DRA	HLA-DRA
1658	DPP6	DPP6
1659	IGFALS	IGFALS
1660	BLMH	BLMH
1661	PCBD1	PCBD1
1662	PSMB1	PSMB1
1663	QPCT	QPCT
1664	C7	C7
1665	GMPPB	GMPPB
1666	MAN2A1	MAN2A1
1667	SLC13A2	SLC13A2
1668	SNRPB	SNRPB
1669	HIBCH	HIBCH

1670	NCS1	NCS1
1671	CLDN11	CLDN11
1672	DECR1	DECR1
1673	PCDH15	PCDH15
1674	PHIP	PHIP
1675	RAB33B	RAB33B
1676	SNRPE	SNRPE
1677	STX3	STX3
1678	TUBGCP6	TUBGCP6
1679	FREM2	FREM2
1680	RAB18	RAB18
1681	ACYP1	ACYP1
1682	ARPC4	ARPC4
1683	HAO2	HAO2
1684	HSPA12A	HSPA12A
1685	PTER	PTER
1686	SYNGR2	SYNGR2
1687	COL5A3	COL5A3
1688	MPHOSPH8	MPHOSPH8
1689	UPK1A	UPK1A
1690	SCHIP1	SCHIP1
1691	SNRPD3	SNRPD3
1692	MELTF	MELTF
1693	MESP2	MESP2
1694	HIKESHI	HIKESHI
1695	ATP5MG	ATP5MG
1696	C1orf68	C1orf68
1697	UPK3B	UPK3B
1698	HIF1AN	HIF1AN
1699	HHIP	HHIP
1700	WWTR1	WWTR1
1701	ATN1	ATN1
1702	DACH1	DACH1
1703	NRF1	NRF1
1704	HMGCS2	HMGCS2
1705	PFKP	PFKP
1706	HSPA1B	HSPA1B
1707	PTGIR	PTGIR
1708	CALM1	CALM1
1709	LGALS9	LGALS9
1710	ALG13	ALG13
1711	SLC9A3R1	SLC9A3R1
1712	CTSF	CTSF
1713	ANTXR1	ANTXR1

1714	CHP1	CHP1
1715	SZT2	SZT2
1716	DERL2	DERL2
1717	FIBP	FIBP
1718	RARRES2	RARRES2
1719	COL22A1	COL22A1
1720	MAP2K6	MAP2K6
1721	DNM3	DNM3
1722	UBE2D2	UBE2D2
1723	AK1	AK1
1724	ACAA2	ACAA2
1725	GNA13	GNA13
1726	ADH6	ADH6
1727	CD300A	CD300A
1728	CAPZA2	CAPZA2
1729	H2BC4	H2BC4
1730	H2BC10	H2BC10
1731	H2BC7	H2BC7
1732	PGRMC1	PGRMC1
1733	VAV2	VAV2
1734	NAIP	NAIP
1735	MIR770	MIR770
1736	SRP14	SRP14
1737	VEGFD	VEGFD
1738	SNX9	SNX9
1739	CEL	CEL
1740	PRCP	PRCP
1741	SLC4A4	SLC4A4
1742	SLC3A2	SLC3A2
1743	TAGLN2	TAGLN2
1744	CAPNS1	CAPNS1
1745	CLEC1B	CLEC1B
1746	PRKAG2	PRKAG2
1747	ORMDL3	ORMDL3
1748	SPTSSA	SPTSSA
1749	MARCHF5	MARCHF5
1750	KAT8	KAT8
1751	CPM	CPM
1752	SLC5A2	SLC5A2
1753	ACO1	ACO1
1754	MYOF	MYOF
1755	AKR1C1	AKR1C1
1756	COMMD1	COMMD1
1757	MSRA	MSRA

1758	RENBP	RENBP
1759	SEMA5A	SEMA5A
1760	GLG1	GLG1
1761	OLFM4	OLFM4
1762	CD248	CD248
1763	NCK2	NCK2
1764	NOX5	NOX5
1765	MIR26A2	MIR26A2
1766	MIR548C	MIR548C
1767	CLCN5	CLCN5
1768	SMAD6	SMAD6
1769	DGAT2	DGAT2
1770	SLC13A1	SLC13A1
1771	LRPAP1	LRPAP1
1772	RHPN2	RHPN2
1773	RNF166	RNF166
1774	LTBP2	LTBP2
1775	ANXA4	ANXA4
1776	RPS3	RPS3
1777	A4GALT	A4GALT
1778	DNAJB1	DNAJB1
1779	FTCD	FTCD
1780	IGFBP6	IGFBP6
1781	BST2	BST2
1782	PDCD6	PDCD6
1783	TSPAN15	TSPAN15
1784	SCPEP1	SCPEP1
1785	ACACB	ACACB
1786	EEF1D	EEF1D
1787	UBE2I	UBE2I
1788	NAT8	NAT8
1789	ACVR2B	ACVR2B
1790	AQP3	AQP3
1791	SP3	SP3
1792	CA2	CA2
1793	CDH11	CDH11
1794	PRKAG1	PRKAG1
1795	SLC3A1	SLC3A1
1796	GSTM3	GSTM3
1797	CTSA	CTSA
1798	DBI	DBI
1799	RPSA	RPSA
1800	PTGES3	PTGES3
1801	PGAM1	PGAM1

1802	SRI	SRI
1803	VDAC3	VDAC3
1804	CTSE	CTSE
1805	GNG2	GNG2
1806	KLF10	KLF10
1807	NCL	NCL
1808	PROZ	PROZ
1809	RNASE1	RNASE1
1810	RPS15A	RPS15A
1811	SNAP23	SNAP23
1812	BCAM	BCAM
1813	ESAM	ESAM
1814	GLYAT	GLYAT
1815	MOCS2	MOCS2
1816	PTP4A1	PTP4A1
1817	RND3	RND3
1818	RPS7	RPS7
1819	SH3GLB1	SH3GLB1
1820	SLC7A8	SLC7A8
1821	TMED10	TMED10
1822	MYL6	MYL6
1823	OTUB1	OTUB1
1824	PTMA	PTMA
1825	RNASE2	RNASE2
1826	TCTN3	TCTN3
1827	GSTK1	GSTK1
1828	PLA1A	PLA1A
1829	STIM2	STIM2
1830	DCD	DCD
1831	ST13	ST13
1832	VWA1	VWA1
1833	MUC13	MUC13
1834	PCYOX1	PCYOX1
1835	S100A16	S100A16
1836	SPRR1B	SPRR1B
1837	CCL24	CCL24
1838	PHLDA3	PHLDA3
1839	PLPP3	PLPP3
1840	SERPINB12	SERPINB12
1841	ATP5PO	ATP5PO
1842	MXRA5	MXRA5
1843	ATP5F1C	ATP5F1C
1844	IGHA1	IGHA1
1845	MUC19	MUC19

1846	CCN5	CCN5
1847	ASPN	ASPN
1848	BBOX1	BBOX1
1849	GNA12	GNA12
1850	LMAN2	LMAN2
1851	JAML	JAML
1852	FERMT3	FERMT3
1853	AQP7	AQP7
1854	CNDP1	CNDP1
1855	SPR	SPR
1856	ACAT1	ACAT1
1857	FBP1	FBP1
1858	PRKACB	PRKACB
1859	FUCA1	FUCA1
1860	SLC25A1	SLC25A1
1861	GNAI2	GNAI2
1862	PSMA7	PSMA7
1863	SGSH	SGSH
1864	ANXA11	ANXA11
1865	GPC4	GPC4
1866	PSMB5	PSMB5
1867	RUVBL1	RUVBL1
1868	DCXR	DCXR
1869	RPS20	RPS20
1870	SLC25A5	SLC25A5
1871	SLC26A4	SLC26A4
1872	SLC2A5	SLC2A5
1873	VAV3	VAV3
1874	VPS35	VPS35
1875	ARHGDIB	ARHGDIB
1876	ARL6	ARL6
1877	CALM3	CALM3
1878	CCT7	CCT7
1879	GALE	GALE
1880	PAICS	PAICS
1881	PCBP1	PCBP1
1882	RAB2A	RAB2A
1883	RPS9	RPS9
1884	SERPINB5	SERPINB5
1885	CAP1	CAP1
1886	CCT4	CCT4
1887	CLIC1	CLIC1
1888	FUCA2	FUCA2
1889	ITIH1	ITIH1

1890	KPNA4	KPNA4
1891	MGST3	MGST3
1892	MTCH2	MTCH2
1893	PCBP2	PCBP2
1894	PPA1	PPA1
1895	RPL12	RPL12
1896	TP53I3	TP53I3
1897	AFM	AFM
1898	CCT6A	CCT6A
1899	CCT8	CCT8
1900	DNASE2	DNASE2
1901	TSPAN1	TSPAN1
1902	TXNL1	TXNL1
1903	AGMAT	AGMAT
1904	C8G	C8G
1905	CD320	CD320
1906	EPB41L2	EPB41L2
1907	GDI2	GDI2
1908	KIF3B	KIF3B
1909	LAMTOR3	LAMTOR3
1910	NECTIN4	NECTIN4
1911	PDIA6	PDIA6
1912	QSOX1	QSOX1
1913	RAB9A	RAB9A
1914	RPS28	RPS28
1915	RPS4X	RPS4X
1916	RPS8	RPS8
1917	TRIM23	TRIM23
1918	ACTR1B	ACTR1B
1919	AKR1E2	AKR1E2
1920	ARPC5	ARPC5
1921	EDF1	EDF1
1922	FLRT2	FLRT2
1923	LRRC15	LRRC15
1924	PIP4K2C	PIP4K2C
1925	SEC11A	SEC11A
1926	SIAE	SIAE
1927	SLC30A7	SLC30A7
1928	SPRR3	SPRR3
1929	VPS28	VPS28
1930	CHMP3	CHMP3
1931	EPDR1	EPDR1
1932	FBLN7	FBLN7
1933	ISLR	ISLR

1934	RAB21	RAB21
1935	STX12	STX12
1936	STX2	STX2
1937	TBC1D15	TBC1D15
1938	TRHDE	TRHDE
1939	HRNR	HRNR
1940	JCHAIN	JCHAIN
1941	CD300E	CD300E
1942	SRP9	SRP9
1943	LAMTOR1	LAMTOR1
1944	PLPP1	PLPP1
1945	TEX264	TEX264
1946	TMEM33	TMEM33
1947	MACROH2A1	MACROH2A1
1948	MUC21	MUC21
1949	TMEM192	TMEM192
1950	C1QTNF9B	C1QTNF9B
1951	RIDA	RIDA
1952	CSK	CSK
1953	RBBP7	RBBP7
1954	DUSP26	DUSP26
1955	P2RY6	P2RY6
1956	HDAC7	HDAC7
1957	ICOS	ICOS
1958	VAC14	VAC14
1959	ADIPOR1	ADIPOR1
1960	MS4A1	MS4A1
1961	FOSB	FOSB
1962	ALG1	ALG1
1963	SULF2	SULF2
1964	EDN2	EDN2
1965	PROX1	PROX1
1966	RNASEH2C	RNASEH2C
1967	CSRP3	CSRP3
1968	SLC6A13	SLC6A13
1969	JAK3	JAK3
1970	SLC2A3	SLC2A3
1971	MYH10	MYH10
1972	GABRR2	GABRR2
1973	SPATA5L1	SPATA5L1
1974	BHLHE40	BHLHE40
1975	WNT2	WNT2
1976	TIE1	TIE1
1977	AZIN2	AZIN2

1978	GPC5	GPC5
1979	CNSN	CNSN
1980	ALMS1	ALMS1
1981	CDK9	CDK9
1982	CREBZF	CREBZF
1983	FGFR4	FGFR4
1984	SMURF1	SMURF1
1985	CCDC47	CCDC47
1986	UTRN	UTRN
1987	SEMA3F	SEMA3F
1988	IFT172	IFT172
1989	SSTR1	SSTR1
1990	DDOST	DDOST
1991	SHROOM3	SHROOM3
1992	FMN1	FMN1
1993	OTUD7B	OTUD7B
1994	SOX11	SOX11
1995	COQ7	COQ7
1996	SETDB1	SETDB1
1997	GRK6	GRK6
1998	TRIB3	TRIB3
1999	TBX2	TBX2
2000	MIR1207	MIR1207
2001	NUP188	NUP188
2002	DSC2	DSC2
2003	PDSS2	PDSS2
2004	AMOTL1	AMOTL1
2005	ANKS6	ANKS6
2006	KRBOX4	KRBOX4
2007	MAGT1	MAGT1
2008	SLC35C1	SLC35C1
2009	STT3B	STT3B
2010	EMC1	EMC1
2011	CD2	CD2
2012	SSTR2	SSTR2
2013	PREX1	PREX1
2014	WDR37	WDR37
2015	SLC34A1	SLC34A1
2016	PRUNE1	PRUNE1
2017	MFF-DT	MFF-DT
2018	KTN1	KTN1
2019	IGHE	IGHE
2020	SRMP1	SRMP1
2021	SELENOI	SELENOI

2022	MAPK7	MAPK7
2023	SLC6A12	SLC6A12
2024	CERS2	CERS2
2025	DAB1	DAB1
2026	IPO7	IPO7
2027	CARM1	CARM1
2028	SLC7A9	SLC7A9
2029	CCNI	CCNI
2030	GGA3	GGA3
2031	PRKCSH	PRKCSH
2032	GP2	GP2
2033	GNE	GNE
2034	MIR939	MIR939
2035	AQP8	AQP8
2036	BCAS3	BCAS3
2037	RIN3	RIN3
2038	SYPL2	SYPL2
2039	DROSHA	DROSHA
2040	SCARA5	SCARA5
2041	ACOX1	ACOX1
2042	METAP2	METAP2
2043	UBE2G2	UBE2G2
2044	EMC6	EMC6
2045	TNFAIP8	TNFAIP8
2046	DNAJB2	DNAJB2
2047	WDR72	WDR72
2048	FUT8	FUT8
2049	SEL1L	SEL1L
2050	NCLN	NCLN
2051	CLEC2B	CLEC2B
2052	TMEM147	TMEM147
2053	SPNS2	SPNS2
2054	CAPZA1	CAPZA1
2055	SYNPO2	SYNPO2
2056	MCC	MCC
2057	RAD51D	RAD51D
2058	ZIC5	ZIC5
2059	SKOR1	SKOR1
2060	TRPS1	TRPS1
2061	ALCAM	ALCAM
2062	ULK1	ULK1
2063	RAMP2	RAMP2
2064	AQP10	AQP10
2065	DDX1	DDX1

2066	TLE4	TLE4
2067	POU4F2	POU4F2
2068	PTH2R	PTH2R
2069	BCAR1	BCAR1
2070	VAMP1	VAMP1
2071	SLC28A2	SLC28A2
2072	ARHGEF6	ARHGEF6
2073		HLA-B
2074		NINJ2
