# Supplementary material for: Niaoduqing alleviates podocyte injury in high glucose model via regulating multiple targets and AGE/RAGE pathway: Network pharmacology and experimental validation
Source: Front Pharmacol. 2023 Feb 27;14:1047184. doi: 10.3389/fphar.2023.1047184 (PMC10009170; doi:10.3389/fphar.2023.1047184)
Supplement: Supplementary file 14 [file Table13.pdf]

Table S13 The result of GO and KEGG enrichment analysis to quercetin

	GO Ontology	GO ID	KEGG ID
1	BP	GO:0043536	hsa04933
2	BP	GO:0009408	hsa05418
3	BP	GO:0003018	hsa04370
4	BP	GO:0043542	hsa05323
5	BP	GO:0010595	hsa04066
6	BP	GO:0043535	hsa04668
7	BP	GO:0032496	hsa04926
8	BP	GO:0007566	hsa05167
9	BP	GO:0010631	hsa05144
10	BP	GO:0090132	hsa05208
11	BP	GO:0002237	hsa05211
12	BP	GO:0090130	hsa05212
13	BP	GO:0010634	hsa05140
14	BP	GO:0043534	hsa04657
15	BP	GO:0009266	hsa05206
16	BP	GO:0007565	hsa04064
17	BP	GO:0002685	hsa04620
18	BP	GO:0044706	hsa05165
19	BP	GO:0031667	hsa04151
20	BP	GO:0001667	
21	BP	GO:0010594	
22	BP	GO:0001894	
23	BP	GO:0010632	
24	BP	GO:0001666	
25	BP	GO:0060249	
26	BP	GO:0036293	
27	BP	GO:1902042	
28	BP	GO:0070482	
29	BP	GO:0035296	
30	BP	GO:0097746	
31	BP	GO:0033280	
32	BP	GO:0090050	
33	BP	GO:0035150	
34	BP	GO:2001233	
35	BP	GO:0050900	
36	BP	GO:0071456	
37	BP	GO:0036294	
38	BP	GO:0031214	
39	BP	GO:0110148	
40	BP	GO:0007584	
41	BP	GO:0032102	
42	BP	GO:0042060	

43	BP	GO:0048608
44	BP	GO:0071453
45	BP	GO:0032103
46	BP	GO:0061458
47	BP	GO:1902041
48	BP	GO:0008217
49	BP	GO:0001541
50	BP	GO:0097191
51	BP	GO:0050920
52	BP	GO:2001234
53	BP	GO:0034605
54	BP	GO:0006801
55	BP	GO:0006809
56	BP	GO:0090049
57	BP	GO:0046209
58	BP	GO:0008625
59	BP	GO:2001057
60	BP	GO:0048872
61	BP	GO:0033273
62	BP	GO:0002042
63	BP	GO:1903035
64	BP	GO:0008585
65	BP	GO:2001237
66	BP	GO:0046545
67	BP	GO:0098869
68	BP	GO:1903054
69	BP	GO:0048661
70	BP	GO:0033138
71	BP	GO:0062197
72	BP	GO:0048545
73	BP	GO:0045765
74	BP	GO:0045986
75	BP	GO:0090594
76	BP	GO:1901342
77	BP	GO:0046660
78	BP	GO:1990748
79	BP	GO:0010715
80	BP	GO:0043117
81	BP	GO:0002688
82	BP	GO:0097237
83	BP	GO:0010038
84	BP	GO:0071318
85	BP	GO:0046683
86	BP	GO:0010544

87	BP	GO:0002687
88	BP	GO:0003158
89	BP	GO:0032930
90	BP	GO:0050921
91	BP	GO:0001890
92	BP	GO:0033135
93	BP	GO:1903589
94	BP	GO:0010212
95	BP	GO:0014074
96	BP	GO:0019430
97	BP	GO:0045662
98	BP	GO:0045932
99	BP	GO:2001236
100	BP	GO:0098754
101	BP	GO:0048732
102	BP	GO:0032928
103	BP	GO:0002262
104	BP	GO:0006979
105	BP	GO:0022407
106	BP	GO:0071450
107	BP	GO:0071451
108	BP	GO:0003012
109	BP	GO:0009314
110	BP	GO:0046697
111	BP	GO:0009410
112	BP	GO:1903034
113	BP	GO:0002719
114	BP	GO:0007263
115	BP	GO:0001819
116	BP	GO:0000303
117	BP	GO:0010575
118	BP	GO:0061082
119	BP	GO:0043410
120	BP	GO:0048771
121	BP	GO:0000305
122	BP	GO:0070168
123	BP	GO:0048660
124	BP	GO:0045766
125	BP	GO:1904018
126	BP	GO:0110150
127	BP	GO:0048659
128	BP	GO:0002040
129	BP	GO:0003180
130	BP	GO:0001975

131	BP	GO:0033198
132	BP	GO:0050731
133	BP	GO:1901654
134	BP	GO:0071248
135	BP	GO:0001893
136	BP	GO:0034405
137	BP	GO:0035633
138	BP	GO:0045907
139	BP	GO:0090322
140	BP	GO:0003176
141	BP	GO:0071222
142	BP	GO:0050866
143	BP	GO:0002701
144	BP	GO:0010543
145	BP	GO:0045429
146	BP	GO:0071276
147	BP	GO:0002064
148	BP	GO:0097529
149	BP	GO:0008406
150	BP	GO:0071219
151	BP	GO:0000302
152	BP	GO:0042307
153	BP	GO:1904407
154	BP	GO:1905314
155	BP	GO:0002703
156	BP	GO:0071241
157	BP	GO:0046688
158	BP	GO:0045137
159	BP	GO:1904591
160	BP	GO:0030595
161	BP	GO:0042554
162	BP	GO:0031334
163	BP	GO:0072593
164	BP	GO:0001974
165	BP	GO:0061028
166	BP	GO:0042311
167	BP	GO:0043114
168	BP	GO:0043618
169	BP	GO:1903587
170	BP	GO:0003007
171	BP	GO:0071216
172	BP	GO:0002686
173	BP	GO:1903053
174	BP	GO:0033002

175	BP	GO:0014075
176	BP	GO:0030195
177	BP	GO:0030225
178	BP	GO:0090257
179	BP	GO:0097305
180	BP	GO:1900047
181	BP	GO:1904036
182	BP	GO:0051924
183	BP	GO:0045661
184	BP	GO:0009636
185	BP	GO:0050730
186	BP	GO:0043620
187	BP	GO:0050819
188	BP	GO:0002043
189	BP	GO:0003179
190	BP	GO:0002366
191	BP	GO:0007548
192	BP	GO:0002263
193	BP	GO:0010574
194	BP	GO:0051146
195	BP	GO:0034599
196	BP	GO:0097193
197	BP	GO:0042306
198	BP	GO:0010573
199	BP	GO:0045428
200	BP	GO:0046824
201	BP	GO:0060135
202	BP	GO:0022617
203	BP	GO:1904589
204	BP	GO:0001885
205	BP	GO:0006940
206	BP	GO:0080164
207	BP	GO:0003170
208	BP	GO:1905330
209	BP	GO:0019229
210	BP	GO:0030193
211	BP	GO:0050918
212	BP	GO:0072678
213	BP	GO:0060326
214	BP	GO:0006879
215	BP	GO:0046686
216	BP	GO:0051926
217	BP	GO:1900046
218	BP	GO:0018105

219	BP	GO:0071496
220	BP	GO:0050818
221	BP	GO:0060562
222	BP	GO:0033627
223	BP	GO:0048638
224	BP	GO:0019216
225	BP	GO:0062012
226	BP	GO:0018209
227	BP	GO:0002697
228	BP	GO:0007568
229	BP	GO:2000379
230	BP	GO:0030336
231	BP	GO:0043154
232	BP	GO:0061045
233	BP	GO:0001570
234	BP	GO:2000146
235	BP	GO:0048678
236	BP	GO:0042310
237	BP	GO:0045445
238	BP	GO:0050767
239	BP	GO:0055072
240	BP	GO:0051271
241	BP	GO:0034103
242	BP	GO:2000117
243	BP	GO:1900182
244	BP	GO:0018108
245	BP	GO:0018212
246	BP	GO:0051781
247	BP	GO:0050878
248	BP	GO:0050678
249	BP	GO:0046849
250	BP	GO:0032535
251	BP	GO:0042692
252	BP	GO:0040013
253	BP	GO:0002690
254	BP	GO:0002718
255	BP	GO:1901655
256	BP	GO:1904035
257	BP	GO:0070167
258	BP	GO:0002367
259	BP	GO:0010959
260	BP	GO:0110149
261	BP	GO:0001503
262	BP	GO:0001558

263	BP	GO:0030593
264	BP	GO:0006816
265	BP	GO:0046822
266	BP	GO:0048568
267	BP	GO:0043254
268	BP	GO:0002683
269	BP	GO:0045785
270	BP	GO:0050673
271	BP	GO:0002698
272	BP	GO:0006939
273	BP	GO:0061387
274	BP	GO:0002443
275	BP	GO:0051090
276	BP	GO:0001938
277	BP	GO:0002526
278	BP	GO:0043406
279	BP	GO:0051960
280	BP	GO:0046916
281	BP	GO:0072676
282	BP	GO:0045446
283	BP	GO:0051101
284	BP	GO:0030282
285	BP	GO:0043500
286	BP	GO:1904019
287	BP	GO:1990266
288	BP	GO:0051051
289	BP	GO:0030168
290	BP	GO:0055007
291	BP	GO:0071621
292	BP	GO:0016049
293	BP	GO:0042110
294	BP	GO:0034101
295	BP	GO:0007569
296	BP	GO:0061041
297	BP	GO:0001101
298	BP	GO:1900180
299	BP	GO:0045471
300	BP	GO:0055076
301	BP	GO:0045598
302	BP	GO:0032355
303	BP	GO:0062013
304	BP	GO:0046718
305	BP	GO:0043524
306	BP	GO:0042542

307	BP	GO:0035148
308	BP	GO:0051384
309	BP	GO:0097530
310	BP	GO:0045834
311	BP	GO:0044409
312	BP	GO:0007605
313	BP	GO:0008360
314	BP	GO:0050770
315	BP	GO:0006606
316	BP	GO:0034614
317	BP	GO:0035051
318	BP	GO:2000377
319	BP	GO:0051170
320	BP	GO:0043271
321	BP	GO:0090316
322	BP	GO:0002700
323	BP	GO:2001242
324	BP	GO:0043112
325	BP	GO:0031960
326	BP	GO:0006937
327	BP	GO:0046890
328	BP	GO:0001659
329	BP	GO:0050954
330	BP	GO:0052126
331	BP	GO:0043405
332	BP	GO:0051302
333	BP	GO:0001936
334	BP	GO:0007626
335	BP	GO:0010721
336	BP	GO:0008361
337	BP	GO:0030308
338	BP	GO:0006164
339	BP	GO:0001935
340	BP	GO:0002285
341	BP	GO:0022408
342	BP	GO:0071674
343	BP	GO:0031099
344	BP	GO:0045216
345	BP	GO:0071902
346	BP	GO:0072522
347	BP	GO:0002291
348	BP	GO:0002887
349	BP	GO:0009750
350	BP	GO:0019062

351	BP	GO:0030214
352	BP	GO:0031284
353	BP	GO:0031915
354	BP	GO:0044557
355	BP	GO:0045843
356	BP	GO:0048671
357	BP	GO:0051918
358	BP	GO:0060947
359	BP	GO:0090037
360	BP	GO:0097084
361	BP	GO:1901203
362	BP	GO:1901725
363	BP	GO:2000048
364	BP	GO:0032388
365	BP	GO:0051701
366	BP	GO:0017038
367	BP	GO:0050679
368	BP	GO:0002573
369	BP	GO:1901215
370	BP	GO:0043281
371	BP	GO:0001660
372	BP	GO:0006527
373	BP	GO:0031652
374	BP	GO:0033632
375	BP	GO:0035865
376	BP	GO:0042167
377	BP	GO:0042447
378	BP	GO:0043619
379	BP	GO:0046149
380	BP	GO:0048635
381	BP	GO:0060312
382	BP	GO:0071679
383	BP	GO:0106049
384	BP	GO:1901862
385	BP	GO:1902667
386	BP	GO:0043523
387	BP	GO:0031669
388	BP	GO:0009612
389	BP	GO:0007596
390	BP	GO:0070374
391	BP	GO:0009746
392	BP	GO:0006787
393	BP	GO:0033004
394	BP	GO:0033015

395	BP	GO:0042118
396	BP	GO:0043129
397	BP	GO:0043301
398	BP	GO:0044650
399	BP	GO:0045602
400	BP	GO:0060009
401	BP	GO:0072683
402	BP	GO:0090557
403	BP	GO:0150065
404	BP	GO:1903800
405	BP	GO:2000727
406	BP	GO:0007599
407	BP	GO:0050817
408	BP	GO:0010001
409	BP	GO:0034284
410	BP	GO:0050769
411	BP	GO:0002551
412	BP	GO:0007183
413	BP	GO:0014745
414	BP	GO:0031282
415	BP	GO:0031392
416	BP	GO:0061418
417	BP	GO:0061744
418	BP	GO:0033157
419	BP	GO:0045444
420	BP	GO:0048588
421	BP	GO:0060560
422	BP	GO:0002699
423	BP	GO:2000116
424	BP	GO:0048738
425	BP	GO:0030213
426	BP	GO:0031650
427	BP	GO:0035641
428	BP	GO:0047484
429	BP	GO:0048681
430	BP	GO:0048875
431	BP	GO:0050930
432	BP	GO:0051917
433	BP	GO:0060088
434	BP	GO:0060841
435	BP	GO:0097531
436	BP	GO:1903039
437	BP	GO:0031668
438	BP	GO:0051402

439	BP	GO:0010763
440	BP	GO:0044406
441	BP	GO:0048569
442	BP	GO:0140374
443	BP	GO:1903799
444	BP	GO:2001028
445	BP	GO:2001279
446	BP	GO:0045926
447	BP	GO:0046883
448	BP	GO:0010951
449	BP	GO:0009743
450	BP	GO:0034612
451	BP	GO:0009165
452	BP	GO:0002093
453	BP	GO:0002693
454	BP	GO:0010819
455	BP	GO:0033631
456	BP	GO:0035864
457	BP	GO:0045651
458	BP	GO:0070571
459	BP	GO:0090336
460	BP	GO:1901163
461	BP	GO:1901201
462	BP	GO:1902931
463	BP	GO:0090596
464	BP	GO:1901293
465	BP	GO:1903522
466	BP	GO:0051091
467	BP	GO:0010466
468	BP	GO:0003184
469	BP	GO:0010934
470	BP	GO:0010935
471	BP	GO:0060977
472	BP	GO:0061450
473	BP	GO:0090036
474	BP	GO:1901550
475	BP	GO:1902894
476	BP	GO:1903140
477	BP	GO:0050708
478	BP	GO:0030728
479	BP	GO:0031649
480	BP	GO:0043116
481	BP	GO:0043217
482	BP	GO:0048535

483	BP	GO:0050665
484	BP	GO:0060008
485	BP	GO:0060391
486	BP	GO:0060749
487	BP	GO:0061377
488	BP	GO:0090190
489	BP	GO:0097709
490	BP	GO:1903209
491	BP	GO:0051962
492	BP	GO:1903829
493	BP	GO:0031065
494	BP	GO:0031290
495	BP	GO:0045780
496	BP	GO:0046716
497	BP	GO:1901739
498	BP	GO:1902105
499	BP	GO:1903532
500	BP	GO:0022409
501	BP	GO:0003159
502	BP	GO:0006525
503	BP	GO:0010042
504	BP	GO:0032793
505	BP	GO:0048670
506	BP	GO:0061154
507	BP	GO:0071243
508	BP	GO:0034504
509	BP	GO:0044403
510	BP	GO:0060485
511	BP	GO:0002689
512	BP	GO:0003177
513	BP	GO:0008090
514	BP	GO:0038083
515	BP	GO:0060117
516	BP	GO:0071498
517	BP	GO:0090026
518	BP	GO:0090189
519	BP	GO:1905209
520	BP	GO:2000047
521	BP	GO:0046879
522	BP	GO:0010720
523	BP	GO:0031281
524	BP	GO:0045540
525	BP	GO:0060965
526	BP	GO:0061042

527	BP	GO:0106118
528	BP	GO:1901522
529	BP	GO:2000725
530	BP	GO:0006913
531	BP	GO:0030198
532	BP	GO:0042063
533	BP	GO:0051169
534	BP	GO:0043062
535	BP	GO:0007162
536	BP	GO:0051222
537	BP	GO:0045229
538	BP	GO:0009914
539	BP	GO:0002052
540	BP	GO:0030194
541	BP	GO:0032740
542	BP	GO:0033081
543	BP	GO:0035162
544	BP	GO:0035640
545	BP	GO:0036303
546	BP	GO:0045723
547	BP	GO:0051349
548	BP	GO:0071636
549	BP	GO:0090335
550	BP	GO:1900048
551	BP	GO:1903306
552	BP	GO:0002440
553	BP	GO:0022604
554	BP	GO:0070372
555	BP	GO:0051047
556	BP	GO:0019932
557	BP	GO:0002438
558	BP	GO:0010226
559	BP	GO:0010288
560	BP	GO:0030810
561	BP	GO:0036003
562	BP	GO:0045649
563	BP	GO:0048641
564	BP	GO:0050820
565	BP	GO:0060149
566	BP	GO:0060967
567	BP	GO:1900373
568	BP	GO:1903077
569	BP	GO:2000679
570	BP	GO:2001026

571	BP	GO:0019058
572	BP	GO:0008202
573	BP	GO:1901214
574	BP	GO:1904951
575	BP	GO:0010996
576	BP	GO:0042730
577	BP	GO:0050927
578	BP	GO:0090312
579	BP	GO:1901623
580	BP	GO:1903055
581	BP	GO:1903798
582	BP	GO:1904996
583	BP	GO:0007517
584	BP	GO:0001963
585	BP	GO:0002092
586	BP	GO:0006027
587	BP	GO:0006706
588	BP	GO:0048668
589	BP	GO:0050926
590	BP	GO:0070920
591	BP	GO:1904376
592	BP	GO:0050863
593	BP	GO:0070371
594	BP	GO:0010894
595	BP	GO:0031954
596	BP	GO:0032967
597	BP	GO:0034698
598	BP	GO:0045830
599	BP	GO:0048873
600	BP	GO:0060142
601	BP	GO:1903037
602	BP	GO:0032386
603	BP	GO:0001945
604	BP	GO:0002675
605	BP	GO:0009065
606	BP	GO:0010714
607	BP	GO:0010818
608	BP	GO:0032801
609	BP	GO:0044331
610	BP	GO:0051894
611	BP	GO:1903672
612	BP	GO:1990776
613	BP	GO:0006936
614	BP	GO:0022011

615	BP	GO:0032292
616	BP	GO:0048679
617	BP	GO:0060969
618	BP	GO:0071549
619	BP	GO:0090025
620	BP	GO:1902175
621	BP	GO:2000406
622	BP	GO:0043087
623	BP	GO:0002449
624	BP	GO:0045861
625	BP	GO:0008209
626	BP	GO:0010800
627	BP	GO:0043304
628	BP	GO:0045939
629	BP	GO:0051968
630	BP	GO:0061311
631	BP	GO:2000637
632	BP	GO:0002460
633	BP	GO:0001818
634	BP	GO:0009306
635	BP	GO:0071900
636	BP	GO:0001516
637	BP	GO:0030212
638	BP	GO:0031063
639	BP	GO:0033006
640	BP	GO:0045987
641	BP	GO:0046457
642	BP	GO:0060055
643	BP	GO:0060148
644	BP	GO:0060390
645	BP	GO:0070528
646	BP	GO:0070723
647	BP	GO:0090183
648	BP	GO:1900027
649	BP	GO:0035592
650	BP	GO:0070997
651	BP	GO:0051251
652	BP	GO:0051098
653	BP	GO:0006026
654	BP	GO:0007435
655	BP	GO:0010039
656	BP	GO:0014044
657	BP	GO:0019835
658	BP	GO:0035767

659	BP	GO:0048841
660	BP	GO:0090022
661	BP	GO:1900745
662	BP	GO:1905476
663	BP	GO:1903706
664	BP	GO:0071692
665	BP	GO:0007159
666	BP	GO:0010165
667	BP	GO:0043552
668	BP	GO:0045920
669	BP	GO:0046685
670	BP	GO:0060122
671	BP	GO:0070570
672	BP	GO:0150117
673	BP	GO:1902692
674	BP	GO:0001662
675	BP	GO:0007431
676	BP	GO:0016242
677	BP	GO:0034383
678	BP	GO:0090075
679	BP	GO:2000352
680	BP	GO:0051346
681	BP	GO:0030099
682	BP	GO:0001569
683	BP	GO:0002209
684	BP	GO:0042462
685	BP	GO:0045454
686	BP	GO:0090181
687	BP	GO:0097421
688	BP	GO:1905207
689	BP	GO:2000403
690	BP	GO:0014706
691	BP	GO:0045786
692	BP	GO:0045860
693	BP	GO:0050727
694	BP	GO:0002691
695	BP	GO:0003203
696	BP	GO:0010762
697	BP	GO:0030224
698	BP	GO:0036314
699	BP	GO:0045191
700	BP	GO:0001990
701	BP	GO:0016202
702	BP	GO:0032570

703	BP	GO:0032885
704	BP	GO:0048846
705	BP	GO:0071542
706	BP	GO:0090218
707	BP	GO:1902284
708	BP	GO:1905332
709	BP	GO:0006163
710	BP	GO:0010742
711	BP	GO:0035886
712	BP	GO:0042491
713	BP	GO:0042596
714	BP	GO:0090077
715	BP	GO:0060537
716	BP	GO:0045923
717	BP	GO:0071548
718	BP	GO:1901861
719	BP	GO:0002696
720	BP	GO:0002714
721	BP	GO:0002891
722	BP	GO:0014037
723	BP	GO:0030501
724	BP	GO:0051281
725	BP	GO:0150077
726	BP	GO:1904994
727	BP	GO:0043434
728	BP	GO:0016032
729	BP	GO:0072521
730	BP	GO:0048634
731	BP	GO:0071470
732	BP	GO:0071634
733	BP	GO:1900371
734	BP	GO:1902622
735	BP	GO:0007409
736	BP	GO:0050867
737	BP	GO:0072594
738	BP	GO:0007520
739	BP	GO:0030808
740	BP	GO:0032965
741	BP	GO:0033003
742	BP	GO:0033574
743	BP	GO:0042168
744	BP	GO:0045687
745	BP	GO:0045773
746	BP	GO:0071312

747	BP	GO:1902895
748	BP	GO:2000404
749	BP	GO:1903131
750	BP	GO:0031952
751	BP	GO:0045776
752	BP	GO:0045981
753	BP	GO:0071604
754	BP	GO:0140353
755	BP	GO:1900544
756	BP	GO:1904706
757	BP	GO:0052548
758	BP	GO:0031295
759	BP	GO:0032620
760	BP	GO:0032660
761	BP	GO:0032881
762	BP	GO:0045124
763	BP	GO:0097178
764	BP	GO:0150076
765	BP	GO:0003197
766	BP	GO:0008631
767	BP	GO:0031670
768	BP	GO:0035272
769	BP	GO:0035307
770	BP	GO:0045933
771	BP	GO:0048538
772	BP	GO:0060119
773	BP	GO:0071354
774	BP	GO:0085029
775	BP	GO:1904646
776	BP	GO:0022411
777	BP	GO:0007157
778	BP	GO:0031294
779	BP	GO:0035094
780	BP	GO:0043300
781	BP	GO:0050919
782	BP	GO:0055010
783	BP	GO:0060976
784	BP	GO:0010975
785	BP	GO:0001754
786	BP	GO:0003044
787	BP	GO:0007595
788	BP	GO:0010712
789	BP	GO:0010761
790	BP	GO:0010799

791	BP	GO:0035315
792	BP	GO:0045601
793	BP	GO:0045747
794	BP	GO:0045911
795	BP	GO:0046677
796	BP	GO:1900744
797	BP	GO:0002673
798	BP	GO:0033628
799	BP	GO:0043303
800	BP	GO:0045912
801	BP	GO:0090311
802	BP	GO:0002204
803	BP	GO:0002208
804	BP	GO:0002279
805	BP	GO:0006692
806	BP	GO:0006693
807	BP	GO:0006778
808	BP	GO:0006953
809	BP	GO:0009409
810	BP	GO:0010862
811	BP	GO:0030857
812	BP	GO:0034332
813	BP	GO:0038084
814	BP	GO:0042304
815	BP	GO:0042461
816	BP	GO:0045190
817	BP	GO:0045778
818	BP	GO:0060964
819	BP	GO:0070169
820	BP	GO:0070741
821	BP	GO:1904894
822	BP	GO:0052547
823	BP	GO:0023061
824	BP	GO:0002448
825	BP	GO:0002639
826	BP	GO:0008089
827	BP	GO:0050873
828	BP	GO:0060688
829	BP	GO:0110151
830	BP	GO:1902930
831	BP	GO:2000677
832	BP	GO:0033674
833	BP	GO:0061564
834	BP	GO:0006636

835	BP	GO:0010656
836	BP	GO:0032964
837	BP	GO:0045104
838	BP	GO:0046850
839	BP	GO:0051339
840	BP	GO:0071622
841	BP	GO:0010718
842	BP	GO:0031103
843	BP	GO:0035196
844	BP	GO:0043392
845	BP	GO:0045103
846	BP	GO:0048260
847	BP	GO:0060147
848	BP	GO:0072132
849	BP	GO:0031279
850	BP	GO:0034381
851	BP	GO:0060966
852	BP	GO:0010524
853	BP	GO:0031050
854	BP	GO:0070918
855	BP	GO:1902893
856	BP	GO:2000179
857	BP	GO:0000768
858	BP	GO:0003229
859	BP	GO:0038066
860	BP	GO:0061614
861	BP	GO:0140253
862	BP	GO:0009117
863	BP	GO:0001658
864	BP	GO:0002090
865	BP	GO:0002886
866	BP	GO:0010332
867	BP	GO:0031529
868	BP	GO:0043388
869	BP	GO:0045599
870	BP	GO:0046456
871	BP	GO:0048008
872	BP	GO:0071385
873	BP	GO:1904645
874	BP	GO:1901361
875	BP	GO:0006611
876	BP	GO:0006695
877	BP	GO:0006754
878	BP	GO:0006949

879	BP	GO:0016447
880	BP	GO:0033619
881	BP	GO:0051155
882	BP	GO:0061756
883	BP	GO:1902653
884	BP	GO:0006753
885	BP	GO:0001954
886	BP	GO:0002763
887	BP	GO:0007405
888	BP	GO:0042743
889	BP	GO:2001244
890	BP	GO:0019369
891	BP	GO:0033013
892	BP	GO:0035306
893	BP	GO:0043551
894	BP	GO:0048010
895	BP	GO:0051055
896	BP	GO:0090303
897	BP	GO:2000351
898	BP	GO:0002712
899	BP	GO:0002889
900	BP	GO:0031102
901	BP	GO:0031663
902	BP	GO:0055008
903	BP	GO:1903670
904	BP	GO:0040014
905	BP	GO:0071384
906	BP	GO:2000401
907	BP	GO:0007588
908	BP	GO:0045576
909	BP	GO:0060113
910	BP	GO:0060393
911	BP	GO:0060675
912	BP	GO:0002704
913	BP	GO:0016239
914	BP	GO:0072171
915	BP	GO:0006749
916	BP	GO:0016126
917	BP	GO:0046530
918	BP	GO:0048247
919	BP	GO:0050771
920	BP	GO:0070542
921	BP	GO:0098930
922	BP	GO:1903409

923	BP	GO:0045453
924	BP	GO:0060389
925	BP	GO:0071677
926	BP	GO:0072577
927	BP	GO:0002562
928	BP	GO:0014015
929	BP	GO:0016444
930	BP	GO:0045600
931	BP	GO:0050766
932	BP	GO:0050922
933	BP	GO:0051893
934	BP	GO:0090109
935	BP	GO:0016445
936	BP	GO:0042490
937	BP	GO:0009206
938	BP	GO:0009145
939	BP	GO:0019226
940	BP	GO:0050805
941	BP	GO:0050810
942	BP	GO:0051966
943	BP	GO:0002381
944	BP	GO:0002548
945	BP	GO:0045123
946	BP	GO:0060415
947	BP	GO:0003208
948	BP	GO:0032722
949	BP	GO:0033555
950	BP	GO:0043550
951	BP	GO:0072078
952	BP	GO:0150116
953	BP	GO:0000271
954	BP	GO:1903036
955	BP	GO:0002637
956	BP	GO:0035924
957	BP	GO:0043299
958	BP	GO:0051145
959	BP	GO:0072088
960	BP	GO:1904427
961	BP	GO:0002437
962	BP	GO:0003151
963	BP	GO:0009201
964	BP	GO:0048844
965	BP	GO:0051881
966	BP	GO:1903201

967	BP	GO:0006024
968	BP	GO:0008088
969	BP	GO:0033077
970	BP	GO:0043627
971	BP	GO:0048662
972	BP	GO:0061333
973	BP	GO:0031100
974	BP	GO:0045685
975	BP	GO:0072028
976	BP	GO:0002200
977	BP	GO:0032418
978	BP	GO:0048644
979	BP	GO:0009064
980	BP	GO:0030500
981	BP	GO:0043537
982	BP	GO:0001895
983	BP	GO:0006023
984	BP	GO:0050772
985	BP	GO:0051279
986	BP	GO:0061844
987	BP	GO:0007422
988	BP	GO:0009791
989	BP	GO:0071229
990	BP	GO:0051149
991	BP	GO:0060968
992	BP	GO:0071260
993	BP	GO:2000243
994	BP	GO:0001910
995	BP	GO:0002312
996	BP	GO:0009205
997	BP	GO:0016575
998	BP	GO:0046889
999	BP	GO:0060395
1000	BP	GO:0050886
1001	BP	GO:0006970
1002	BP	GO:0010660
1003	BP	GO:0032370
1004	BP	GO:0042440
1005	BP	GO:0098586
1006	BP	GO:1900542
1007	BP	GO:0009142
1008	BP	GO:0010507
1009	BP	GO:0055013
1010	BP	GO:0097006

1011	BP	GO:0006140
1012	BP	GO:0022406
1013	BP	GO:1903510
1014	BP	GO:0048041
1015	BP	GO:1901606
1016	BP	GO:2000177
1017	CC	GO:0045121
1018	CC	GO:0098857
1019	CC	GO:0005901
1020	CC	GO:0044853
1021	CC	GO:0031093
1022	CC	GO:0031091
1023	CC	GO:0009897
1024	CC	GO:0031968
1025	CC	GO:0019867
1026	CC	GO:0016327
1027	CC	GO:0031258
1028	CC	GO:0005788
1029	CC	GO:0034774
1030	CC	GO:0060205
1031	CC	GO:0031983
1032	CC	GO:0030139
1033	CC	GO:0031045
1034	CC	GO:0005640
1035	CC	GO:0032839
1036	CC	GO:0005925
1037	CC	GO:0030055
1038	CC	GO:0062023
1039	CC	GO:0001772
1040	CC	GO:0005774
1041	CC	GO:0005911
1042	CC	GO:0005637
1043	CC	GO:1904115
1044	CC	GO:0005758
1045	MF	GO:0048018
1046	MF	GO:0030546
1047	MF	GO:0005125
1048	MF	GO:0020037
1049	MF	GO:0046906
1050	MF	GO:0016705
1051	MF	GO:0042056
1052	MF	GO:0005126
1053	MF	GO:0050840
1054	MF	GO:0001618

1055	MF	GO:0140272
1056	MF	GO:0016209
1057	MF	GO:0004497
1058	MF	GO:0005178
1059	MF	GO:0008083
1060	MF	GO:0008201
1061	MF	GO:0008239
1062	MF	GO:0034713
1063	MF	GO:0008603
1064	MF	GO:0005539
1065	MF	GO:0005172
1066	MF	GO:0005161
1067	MF	GO:0010181
1068	MF	GO:1901681
1069	MF	GO:0045236
1070	MF	GO:0016702
1071	MF	GO:0016701
1072	MF	GO:0005160
1073	MF	GO:0045499
1074	MF	GO:0001968
1075	MF	GO:0003785
1076	MF	GO:0004177
1077	MF	GO:0016712
1078	MF	GO:0016709
1079	MF	GO:0008009
1080	MF	GO:0004601
1081	MF	GO:0016597
1082	MF	GO:0050661
1083	MF	GO:0016684
1084	MF	GO:0005507
1085	MF	GO:0042379
1086	MF	GO:0050660
