# Supplementary material for: Niaoduqing alleviates podocyte injury in high glucose model via regulating multiple targets and AGE/RAGE pathway: Network pharmacology and experimental validation
Source: Front Pharmacol. 2023 Feb 27;14:1047184. doi: 10.3389/fphar.2023.1047184 (PMC10009170; doi:10.3389/fphar.2023.1047184)
Supplement: Supplementary file 15 [file Table8.pdf]

Table S8 The list of potential therapeutic targets

	Target
1	SPP1
2	PTGS2
3	VEGFA
4	TGFB1
5	HMOX1
6	FLT1
7	SOD1
8	CXCL10
9	REN
10	DPP4
11	THBD
12	AGTR1
13	ACE
14	ICAM1
15	NOS3
16	F3
