# Supplementary material for: Niaoduqing alleviates podocyte injury in high glucose model via regulating multiple targets and AGE/RAGE pathway: Network pharmacology and experimental validation
Source: Front Pharmacol. 2023 Feb 27;14:1047184. doi: 10.3389/fphar.2023.1047184 (PMC10009170; doi:10.3389/fphar.2023.1047184)
Supplement: Supplementary file 16 [file Table10.pdf]

Table S10 The topological parameters of each nodes in PPI network of Niaoduqing

| | Node | Degree Value | Betweenness Centrality | Closeness Centrality |
|---|---|---|---|---|
| 1 | VEGFA | 30 | 17.083334 | 1 |
| 2 | ICAM1 | 28 | 10.25 | 0.9375 |
| 3 | NOS3 | 28 | 14.330953 | 0.9375 |
| 4 | PTGS2 | 26 | 9.930952 | 0.88235295 |
| 5 | ACE | 24 | 5.759524 | 0.8333333 |
| 6 | HMOX1 | 22 | 5.904762 | 0.7894737 |
| 7 | TGFB1 | 22 | 3.0738096 | 0.7894737 |
| 8 | REN | 22 | 3.5738096 | 0.7894737 |
| 9 | SPP1 | 22 | 3.0738096 | 0.7894737 |
| 10 | F3 | 16 | 1.6904762 | 0.6818182 |
| 11 | AGTR1 | 16 | 0 | 0.6818182 |
| 12 | CXCL10 | 16 | 2.6857142 | 0.6818182 |
| 13 | THBD | 14 | 1.2857143 | 0.65217394 |
| 14 | FLT1 | 14 | 0.5714286 | 0.65217394 |
| 15 | DPP4 | 12 | 0.78571427 | 0.625 |
| 16 | SOD1 | 8 | 0 | 0.5769231 |
